# A Unique Set of the *Burkholderia* Collagen-Like Proteins Provides Insight into Pathogenesis, Genome Evolution and Niche Adaptation, and Infection Detection

**DOI:** 10.1371/journal.pone.0137578

**Published:** 2015-09-10

**Authors:** Beth A. Bachert, Soo J. Choi, Anna K. Snyder, Rita V. M. Rio, Brandon C. Durney, Lisa A. Holland, Kei Amemiya, Susan L. Welkos, Joel A. Bozue, Christopher K. Cote, Rita Berisio, Slawomir Lukomski

**Affiliations:** 1 Department of Microbiology, Immunology and Cell Biology, West Virginia University, Morgantown, West Virginia, United States of America; 2 Department of Biology, West Virginia University, Morgantown, West Virginia, United States of America; 3 Department of Chemistry, West Virginia University, Morgantown, West Virginia, United States of America; 4 Bacteriology Division, The United States Army of Medical Research Institute of Infectious Diseases, Fort Detrick, Frederick, Maryland, United States of America; 5 Institute of Biostructures and Bioimaging, National Research Council, Naples, Italy; University of Toledo School of Medicine, UNITED STATES

## Abstract

*Burkholderia pseudomallei* and *Burkholderia mallei*, classified as category B priority pathogens, are significant human and animal pathogens that are highly infectious and broad-spectrum antibiotic resistant. Currently, the pathogenicity mechanisms utilized by *Burkholderia* are not fully understood, and correct diagnosis of *B*. *pseudomallei* and *B*. *mallei* infection remains a challenge due to limited detection methods. Here, we provide a comprehensive analysis of a set of 13 novel *Burkholderia* collagen-like proteins (Bucl) that were identified among *B*. *pseudomallei* and *B*. *mallei* select agents. We infer that several Bucl proteins participate in pathogenesis based on their noncollagenous domains that are associated with the components of a type III secretion apparatus and membrane transport systems. Homology modeling of the outer membrane efflux domain of Bucl8 points to a role in multi-drug resistance. We determined that *bucl* genes are widespread in *B*. *pseudomallei* and *B*. *mallei*; Fischer’s exact test and Cramer’s V^2^ values indicate that the majority of *bucl* genes are highly associated with these pathogenic species versus nonpathogenic *B*. *thailandensis*. We designed a *bucl*-based quantitative PCR assay which was able to detect *B*. *pseudomallei* infection in a mouse with a detection limit of 50 CFU. Finally, chromosomal mapping and phylogenetic analysis of *bucl* loci revealed considerable genomic plasticity and adaptation of *Burkholderia* spp. to host and environmental niches. In this study, we identified a large set of phylogenetically unrelated *bucl* genes commonly found in *Burkholderia* select agents, encoding predicted pathogenicity factors, detection targets, and vaccine candidates.

## Introduction

Collagen structure is formed by three polypeptide chains of continuous repetitive Gly-Xaa-Yaa (GXY) sequence, each adopting left handed polyproline II type helices that combined form a right-handed superhelix [1]. It is a universal structure that is broadly found among members of all three domains of life. It is the most abundant protein in mammals where it harbors important structural functions in the extracellular matrix and in support of cell adhesion, differentiation and growth [2, 3]. The prokaryotic collagen was identified and studied more recently, and has similar GXY sequence and triple helical structure [4–8]. In mammalian collagens, proline (Pro) in the Y position is hydroxylated post-translationally and resulting Hyp (hydroxyproline) residues confer the maximum stability to the triple helix. As bacteria lack the prolyl hydroxylase required for these residues, bacterial collagens must be stabilized by other mechanisms, including increased proline content and electrostatic interactions between amino acid side chains [9–12]. Several bacterial collagen-like proteins have been shown to form stable triple helices, including streptococcal collagen-like proteins 1 and 2 of *Streptococcus pyogenes* [4, 13], rCL_Cp_ from *Clostridium perfringens* [14], and BclA of *Bacillus anthracis* [15, 16]. Bacterial collagen-like proteins are found in species that are pathogenic to humans and animals [5–8, 16–22]. They are often surface-exposed and participate in important pathogenesis processes, including adherence and biofilm formation, host colonization and immune evasion [6, 7, 18, 19, 23–30]. Several collagen-like genes have been evaluated as biomarkers for pathogen detection by targeting their conserved non-collagenous regions [31, 32] and for strain fingerprinting by targeting highly polymorphic repetitive collagen-like sequences [32–35].

The *Burkholderia* species are ubiquitous in the environment but also include animal and plant pathogens. A group of 17 closely related species, designated *B*. *cepacia* complex organisms, cause pulmonary infections primarily in patients with cystic fibrosis [36]. Two other species, *Burkholderia pseudomallei* and *Burkholderia mallei*, are significant human and animal pathogens in endemic regions and also represent biowarfare threats. These bacteria have been classified as category B priority pathogens, in part due to their high infectivity, an intrinsic broad-spectrum antibiotic resistance, and previous use as biological weapons during wartime [37]. *B*. *pseudomallei* is a soil saprophyte endemic to southeastern Asia and northern Australia, which causes melioidosis in humans. Melioidosis has a variety of clinical outcomes, from localized skin infection to pneumonia and acute septicemia, as well as chronic illness with abscess formation in major organs [38]. As 50% of patients with septicemic melioidosis die within 48 hours, rapid diagnosis is crucial to patient survival [39]. *B*. *pseudomallei* has a large genome of about 7.2 Mb, which undergoes frequent horizontal gene transfer as evidenced by multiple genomic islands that differ between strains [40]. *B*. *mallei* is a closely related bacterium with a smaller genome, ~5.8 Mb [41]. It is the causative agent of glanders in horses and other animals that can be transmitted to humans. It has been demonstrated by multi-locus sequence typing analysis that *B*. *mallei* is a clonal derivative of *B*. *pseudomallei* [42], which has undergone significant genomic reduction and rearrangement during host-adaptation [41]. Consequently, *B*. *mallei* is unable to survive outside the host. *B*. *mallei* was one of the first microbes to be weaponized during World War I to infect livestock and humans [37]. A third closely related organism, *B*. *thailandensis*, is considered non-pathogenic for humans [43]. *B*. *thailandensis* is also a soil saprophyte with a large genome of ~6.7 Mb, which is endemic to geographical regions coinciding with *B*. *pseudomallei* [43, 44]; therefore, it is necessary to differentiate between the two species.

In this study, we identified and characterized an unexpectedly large set of 13 distinct *Burkholderia* collagen-like (*bucl*/Bucl) genes and proteins that are conserved in pathogenic *B*. *pseudomallei* and *B*. *mallei* species. We report the widespread presence of *bucl* genes in *B*. *pseudomallei* and *B*. *mallei* assessed by bioinformatics and analytical PCR, explore their phylogenetic relationships, infer important pathogenicity traits and antibiotic resistance mechanisms associated with Bucl proteins, and demonstrate the use of *bucl* genes as detection markers for these select agents in an animal model of infection.

## Results

### Identification of *Burkholderia* collagen-like (*bucl*) genes

An increasing number of collagen-like proteins have recently been identified and studied in a variety of bacterial species, including Gram-positive pathogenic group A [5–8, 17], B (SL, unpublished data), C [20, 45] streptococci and pneumococci [18], bacilli and clostridia [16, 21, 32], as well as Gram-negative respiratory pathogen *Legionella pneumophila* ([19]; SL, unpublished data). Here, we assessed the presence and distribution of the collagen-like proteins among *Burkholderia* species in the Pfam collagen family database (PF01391). We identified a total of 85 sequences among the members of the *Burkholderiaceae* family, with 77 of these sequences designated *Burkholderia* collagen-like (Bucl) proteins, among various species of the *Burkholderia* genus. We next focused on 59 protein sequences found in three closely related species of *Burkholderia*, *B*. *pseudomallei* (Bp), *B*. *mallei* (Bm), and *B*. *thailandensis* (Bt) that we initially categorized into 16 (Bucl1-16) protein types, based on domain organization and GXY-repeat types in their collagen-like (CL) regions; subsequent refinement eliminated three Bucl types, resulting in 13 Bucl proteins 1, 2, 3, 4, 5, 6, 7, 8, 10, 13, 14, 15, and 16. To assess their distribution, nucleotide sequences of these 13 *bucl* genes were used as independent queries to BLASTn-search the NCBI nonredundant database. Though we observed collagen-like sequences in other *Burkholderia* species, this set of 13 *bucl* genes and proteins were unique to Bp, Bm, and Bt species.

### Identification of *bucl* genes in Bp K96243, proof of principle

**Fig 1:**
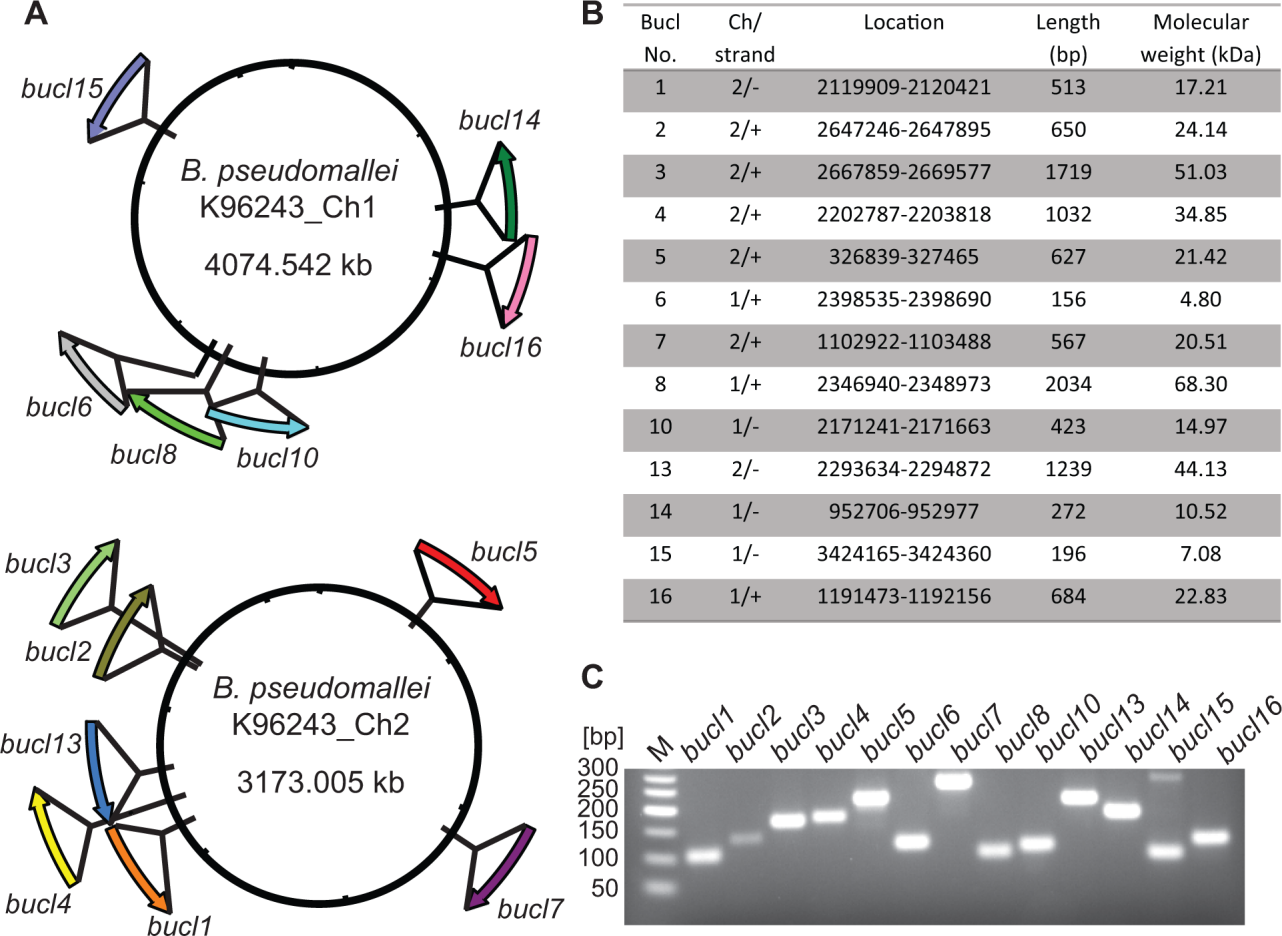
Identification and characterization of *bucl* genes in *B*. *pseudomallei* reference strain K96243. (A) Schematic representation of *bucl* distribution. Relative position and orientation of each *bucl* gene is shown; six *bucl* genes are present on chromosome one and seven on chromosome two. (B) Summary table of *bucl* distribution. *bucl* location, orientation, and length are mapped to the genome of Bp K96243. Molecular weight of each Bucl protein encoded by each *bucl* allele is shown. (C) PCR amplification of 13 *bucl* genes from Bp K96243. Primers were designed targeting the non-collagenous conserved regions, and PCR conditions were established for all *bucl* amplicons at a uniform annealing temperature of 64°C. Amplicon sizes; *bucl1*, 123 bp; *bucl2* 133 bp; *bucl3*, 166 bp; *bucl4*, 176 bp; *bucl5*, 216 bp; *bucl6*, 115 bp; *bucl7*, 264 bp; *bucl8*, 96 bp; *bucl10*, 109 bp; *bucl13*, 212 bp; *bucl14*, 178 bp; *bucl15*, 95 bp; and *bucl16*, 123 bp; M, 50-bp DNA size marker.

**Fig 2:**
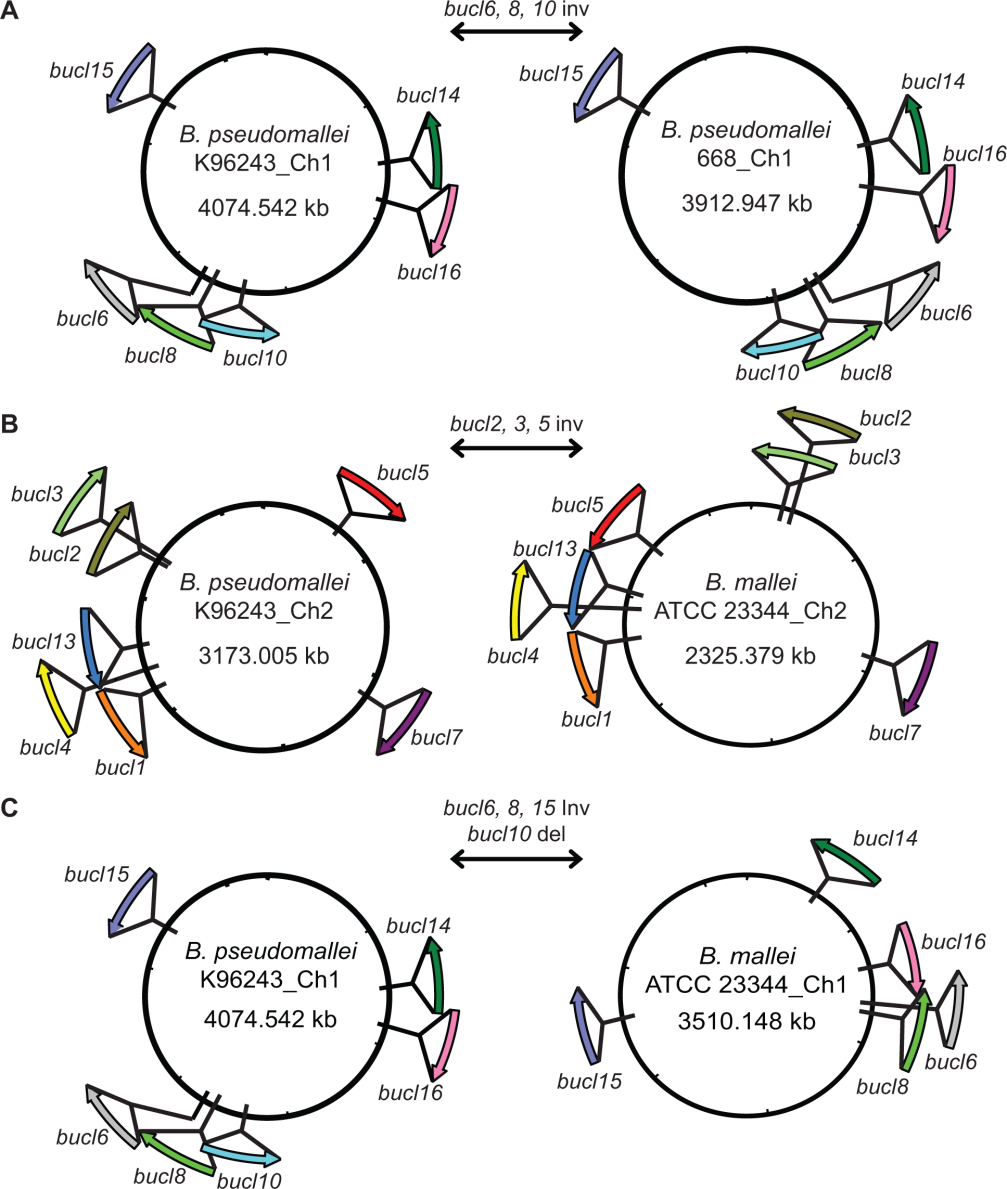
Chromosomal rearrangements and deletions involving *bucl* loci. Relative positions and orientations of each *bucl* gene was rendered from the NCBI database, and used for chromosomal mapping. (A) Intraspecies chromosomal inversion (inv) between *B*. *pseudomallei* strains K96243 and 668 involving the region encoding *bucl* genes 6, 8, and 10. (B) Interspecies chromosomal inversion between Bp K96243 and Bm ATCC 23344 involving the region encoding *bucl* genes 2, 3, and 5 on chromosome 2. (C) Interspecies chromosomal inversion involving *bucl* genes 6, 8, and 15, and deletion of *bucl*10 between Bp K96243 and Bm ATCC 23344 on chromosome 1. Ch, chromosome.

A BLAST search of *bucl* alleles from various strains against the genome sequence of the reference strain Bp K96243 revealed that all 13 *bucl* genes were present and were distributed around both chromosomes (Fig 1A). Six *bucl* genes were localized on chromosome one and seven *bucl* genes on chromosome two, and were found on both plus and minus strands (Fig 1A and 1B). The presence of each *bucl* gene in Bp K96243 genome was confirmed by PCR with primers targeting the noncollagenous regions (Fig 1C). Mapping of *bucl* genes in additional seven Bp and four Bm fully sequenced genomes revealed significant intra- and inter-species genomic rearrangements involving *bucl* loci (Fig 2). For example, the region encoding *bucl* genes 6, 8, and 10 in Bp 668 was inverted compared to Bp K96243 genome (Fig 2A). Additionally, we observed both rearrangements (Fig 2B and 2C) and deletions of *bucl* loci (Fig 2C) in Bm genomes, compared to Bp, which is consistent with Bm-genomic plasticity as well as the evolution of Bm from Bp through genome reduction [41, 46]. To further characterize the genomic organization of these strains, organizational patterns (OP) of *bucl* biomarkers were assigned according to their position and orientation on each chromosome (Table 1). In aggregate, chromosomal rearrangements occur more frequently on chromosome one (six distinct organizational patterns were observed for both Bp and Bm strains analyzed) compared to chromosome two (three organizational patterns observed). While only one major organizational pattern on chromosome 1, Ch1 OPII, was found exclusively among Bp strains, major organizational pattern on chromosome 2, Ch2 OPI, was found in both Bp and Bm genomes. All observed rearrangements were intrachromosomal in both species, indicating no exchange of genetic material involving *bucl* markers occurred between the chromosomes. In summary, consistent with bioinformatic data, we here confirmed by PCR the presence of all 13 *bucl* genes in Bp K96243. We also captured significant genomic plasticity of the Bp and Bm species by employing *bucl* markers.

**Table 1:** Assessment of genomic plasticity of *B*. *pseudomallei* and *B*. *mallei* using biomarkers.

		*bucl*s on Chromosome 1				*bucl*s on Chromosome 2	
OP^a^	Strains	plus strand	minus strand	Ch2 OP	Strains	plus strand	minus strand
Ch1 OPI	BpK9624	6, 8, 16	10, 14, 15	Ch2 OPI	BpK9624, Bp668, Bp1026b, Bp1106a, Bp1710b, BpBP006, Bp305, Bp146, Bp511, Bp520, Bp20B16, Bp78, Bm10229, Bm10247, BmSAVP1	2, 3, 4, 5, 7	1, 13
Ch1 OPII	Bp668, Bp1026b, Bp1106a, Bp1710b, BpBP006, Bp305, Bp79, Bp146, Bp511, Bp520, Bp20B16, Bp78	10, 16	6, 8, 14, 15	Ch2 OPII	Bp79	2, 3, 4, 5	1, 7, 13
Ch1 OPIII	Bm23344	15, 16	6, 8, 14	Ch2 OPIII	Bm23344	4, 7	1, 2, 3, 5, 13
Ch1 OPIV	Bm10229	6, 8, 14, 15, 16	10				
Ch1 OPV	Bm10247	10, 15	6, 8, 14, 16				
Ch1 OPVI	BmSAVP1	15	6, 8, 10, 14, 16				

^a^ Organizational patterns (OP) of *bucl* genes were assigned to each chromosome, Ch1 and Ch2, according to position and orientation. OPs were labeled I-VI for chromosome 1, and I-III for chromosome 2. *bucl* position on the plus or minus strand is shown corresponding to each OP.

### Characterization of Bucl proteins

Overall characteristics of Bucl proteins were examined in a set of geographically diverse *Burkholderia* strains sequenced, including 13 Bp, 11 Bm, and 9 Bt strains (Table 2, Table 3). All 13 Bucl proteins identified contained a collagen-like region (CL) flanked by noncollagenous N- and C-terminal regions. The noncollagenous regions were conserved among all three species within each Bucl with sporadic length variations observed (Table 2). As expected, the CL regions of the same Bucl varied significantly in length between strains due to differing numbers of GXY repeats. For example, Bucl3 varied from 38 repeats to 63 repeats in different strains of Bp, Bm, and Bt (Table 2). The triplet usage was unique to each Bucl across species and usually one or two GXY-repeat types dominated each CL region. For example, Bucl1 and Bucl8 contained exclusively GAN and GAS repeats, respectively, while Bucl3 contained predominantly GTS repeats and Bucl10 had predominantly GIH triplets.

**Table 2:** Characterization of Bucl proteins in *Burkholderia* ^a^.

	No. of amino acids			Collagen-like region (CL)		Structural predictions		
Bucl No.	Total	N-terminus	C-terminus	No. of GXY repeats	GXY type	Putative domains	^b^SS	^c^TM
Bucl1	152–197	42	80–89	7–22	**GAN**	N/A	No	Yes
Bucl2	171–228	141	21	3–19	**GEV.** GEA	N/A	No	No
Bucl3	551–640	44	372–405	38–63	**GTS.** GSS	Talin-1	Yes	Yes
Bucl4	297–379	271	9–18	7–30	**GVS. GAS**	Bac_export_1	Yes	Yes, CL region
Bucl5	168–230	35–41	114	7–25	**GLE.** GPE. GLD. GFD	N/A	No	No
Bucl6	40–88	1	27	4–20	GAL. **GAS. GAA.** GAE	N/A	No	Yes, CL region
Bucl7	188–212	134	36	5–14	**GLS. GSS. GAS.** GVA	N/A	No	Yes, CL region
Bucl8	608–677	522	74	4–24	**GAS**	OEP	Yes	Yes, CL region
Bucl10	92–155	2–8	63	8–25	**GIH.** GMH. GMR	N/A	No	No
Bucl13	385–433	24	318	12–25	**GIR.** GVR. GSG. GGS.	SBP_bac_3	No	Yes
Bucl14	83–191	11	69	2–37	**GWC. GRC. GRR.** GRH	N/A	No	Yes, CL region
Bucl15	56–91	21–69	4	5–14	GVL. GAL. GML. **GAT. GAI.** GAA	N/A	Yes	Yes, CL region
Bucl16	227–307	65–67	148	4–30	GFG. GVD. **GFD. GAF**	N/A	No	Yes, CL region

^a^ Characteristics of Bucl proteins are shown based on analysis of completed genomes of 13 Bp, 11 Bm, and 9 Bt strains (see Table 3). The total protein length and length of protein sequences that are amino- and carboxyl-terminal to CL regions in each Bucl protein is shown as amino acid number, whereas the length of each CL region, which varies between strains, is expressed as the number of GXY repeats. Predominant GXY repeats are represented in bold text. Putative domains in the noncollagenous regions of each Bucl are shown: Talin-1 domain; Bac_export_1, bacterial export protein family 1; OEP, outer membrane efflux protein; and SBP_bac_3, bacterial extracellular solute-binding protein family 3.

^b^ SS; Signal sequence predictions are based on hidden Markov model predictions in the SignalP 3.0 server.

^c^ TM; Transmembrane domain predictions were made using TMPred.

**Table 3:** *Burkholderia* strains used in this study^a^.

Species	Abbreviation	Strain	Isolate information	
*B*. *pseudomallei*	BpK9624^b^	K96243	female diabetic patient- Khon Kaen hospital, Northeast Thailand	1996
*B*. *pseudomallei*	Bp1710b^b^	1710b	relapse of same patient infected with 1710a, blood culture, Northeast Thailand, Sappasithiprasong hospital	1999
*B*. *pseudomallei*	Bp305^b^	MSHR305	brain sample, fatal encephalomyelitis, Australia, Royal Darwin hospital	1994
*B*. *pseudomallei*	Bp1026b^b^	1026b	blood culture from 29-year old female rice farmer with diabetes milletus, Northeast Thailand, Sappasithiprasong hospital	1993
*B*. *pseudomallei*	BpBP006	BPC006	Blood from patient with Type I diabetes and multiple abscesses, China, Baoting Town, Hainan	2008
*B*. *pseudomallei*	Bp1106a^b^	1106a	female rice farmer, Northeast Thailand, Sappasithiprasong hospital	1993
*B*. *pseudomallei*	Bp79	NCTC 13179	skin ulcer, Australia	2014
*B*. *pseudomallei*	Bp668^b^	668	blood culture from 53-year old male patient with severe melioidosis encephalomyelitis, Darwin Australia	1995
*B*. *pseudomallei*	Bp146	MSHR146	goat udder, Australia	1992
*B*. *pseudomallei*	Bp511	MSHR511	throat of goat, Australia	1997
*B*. *pseudomallei*	Bp520	MSHR520	human blood culture, Australia	1998
*B*. *pseudomallei*	Bp20B16	NAU20B-16	soil, Australia	2006
*B*. *pseudomallei*	Bp78	NCTC 13178	human post-mortem brain, Australia	N/A
				
*B*. *mallei*	BmSAVP1	SAVP1	pathogenic strain which became avirulent after passage through 6 equids, originally caused disease in a mule in India	
*B*. *mallei*	Bm10229	NCTC 10229	Europe	
*B*. *mallei*	Bm10247	NCTC 10247	Europe	
*B*. *mallei*	Bm23344^b^	ATCC 23344	human post-mortem knee fluid, skin pustules and blood, Burma	1944
*B*. *mallei*	Bm21280^b^	2002721280	Iran	1952
*B*. *mallei*	BmA188	A188	>8 passages	
*B*. *mallei*	BmA193	A193	Pasteur Institute, France	1964
*B*. *mallei*	Bm10399^b^	ATCC 10399	horse lung, Southern China	1949
*B*. *mallei*	BmPRL20	PRL-20	blood of a gelding from the Lahore Polo Club, Lahore, Pakistan	2005
*B*. *mallei*	Bm11	strain_11	human, Turkey	1949
*B*. *mallei*	Bm6	strain_6	human	1950
				
*B*. *thailandensis*	BtE264	E264	rice field soil sample, Central Thailand	
*B*. *thailandensis*	Bt121	MSMB121	soil, Australia	2007
*B*. *thailandensis*	BtH0587	H0587	human pleural wound, LA, United States	1997
*B*. *thailandensis*	BtE444	E444	soil, Thailand	2002
*B*. *thailandensis*	Bt43	MSMB43	bore water source in Darwin, Australia; first isolate of *B*. *thailandensis* in Australia	
*B*. *thailandensis*	BtTXDOH	TXDOH	United States	
*B*. *thailandensis*	Bt21723	2002721723	Human, CDC	2010
*B*. *thailandensis*	Bt4	4		
*B*. *thailandensis*	BtE555	E555		

^a^ 13 Bp, 11 Bm, and 9 Bt strains listed in this table were used for analysis of Bucl characteristics (Table 2), in part for distribution assessment (Table 4), and for phylogenetic analyses. Strain abbreviations listed in this table are used in all figures.

^b^ Strains which were also tested by PCR for *bucl* distribution.

**Fig 3:**
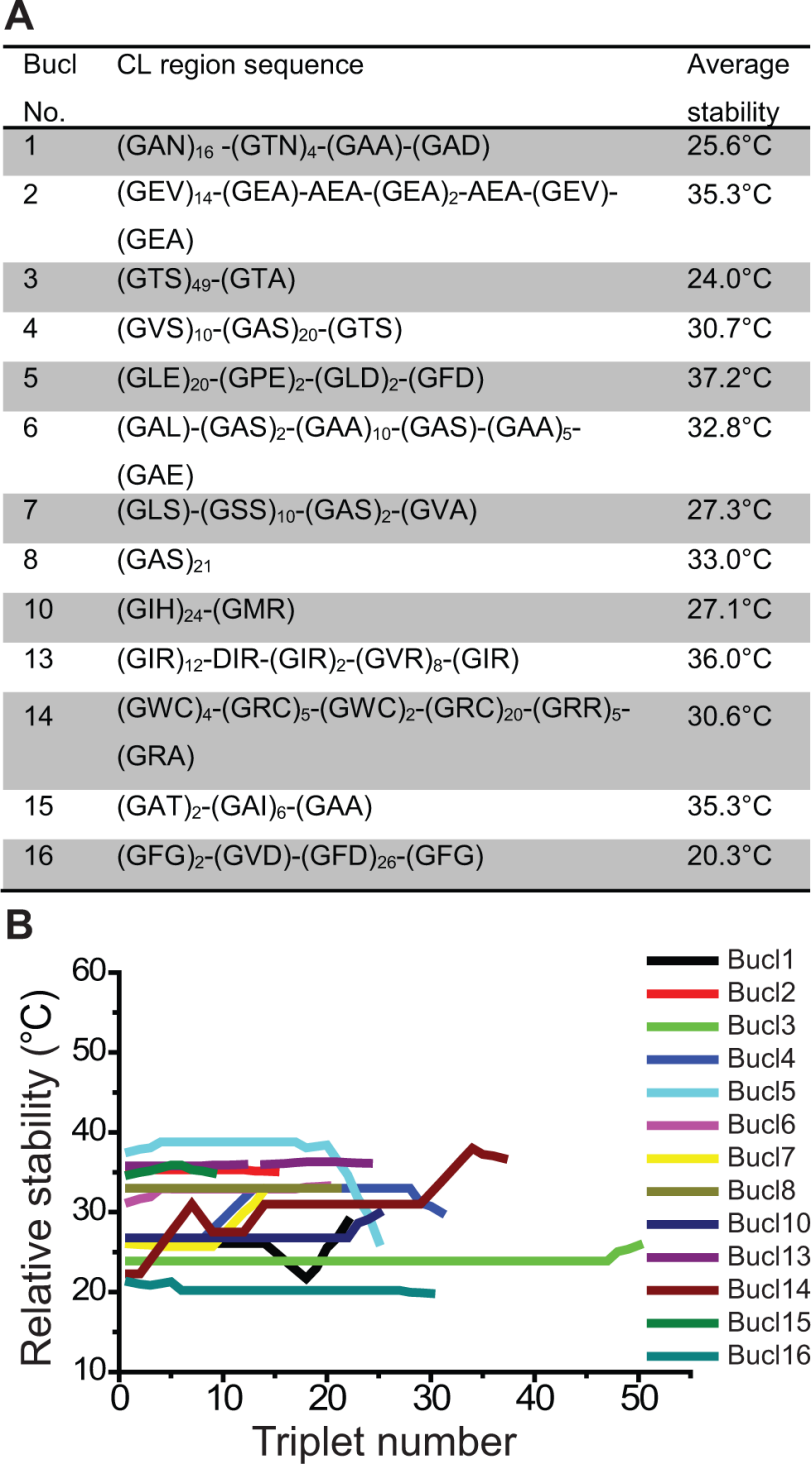
Thermal stability of the Bucl collagen regions. (A) The CL region sequences, representative of all 13 Bucl proteins, plotted in B) are shown with averaged stability values calculated for the entire CL region. (B) Triple helix thermal stability plot. Amino acid sequences for Bucl-CL regions shown in A) were used to model thermal stability with an algorithm developed by Persikov et al. 2005. Relative thermal stability is shown as the melting temperature for each GXY triplet along each Bucl-CL region.

In order to assess whether the Bucl proteins will form collagen-like triple helices, stability predictions were performed on representative Bucl-CL amino acid sequences. GXY repeat number in Bucl proteins varies from 2 in Bucl14 to 63 in Bucl3 (Table 2). Stability of the predicted collagenous regions of each Bucl was computed using an approach derived from host-guest peptide studies [47]. Examination of the stability profiles shows highest stabilities for Bucl2, Bucl5, Bucl13, and Bucl15, with predicted melting temperatures ranging between 35–38°C, while all other Bucl proteins had melting temperatures between 20–35°C (Fig 3). Transmembrane regions were predicted in CL domains of Bucl proteins 4, 6, 7, 8, 14, 15, and 16, whose stability ranks low (Fig 3). Hydrophobic interactions occurring in a membrane environment likely stabilize these triple helices.

### Structural Predictions

**Fig 4:**
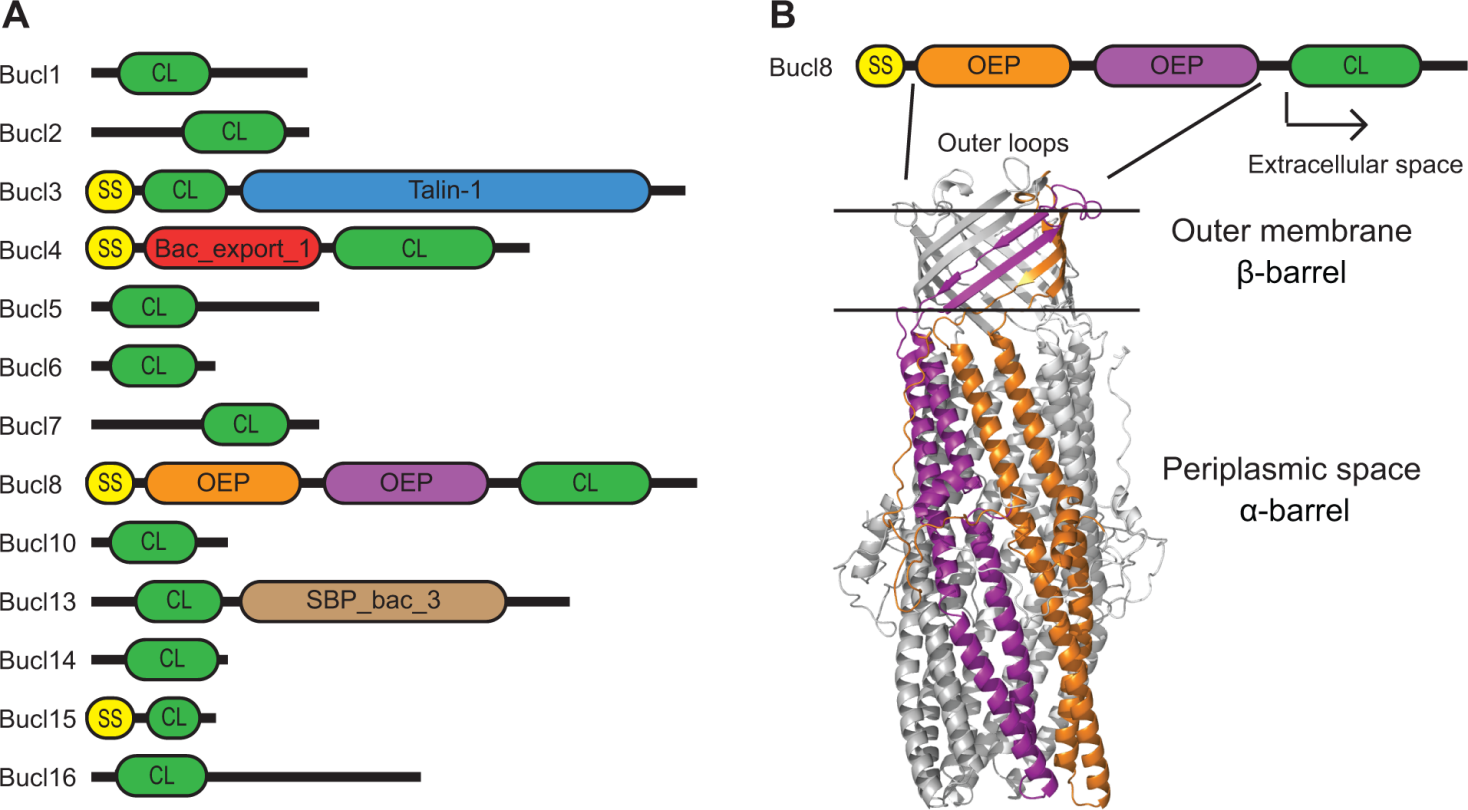
Characterization of *Burkholderia* collagen-like proteins. (A) Architecture of Bucl proteins identified in collagen Pfam data base (not to scale). Proteins were categorized into 13 distinct Bucl types based on sequence similarities and domain organization. Predicted domains in each Bucl are shown: SS, signal sequence; CL, collagen-like domain; Talin-1 domain; Bac_export_1, bacterial export protein family 1; OEP, Outer Membrane Efflux Protein; and SBP_bac_3, bacterial extracellular solute-binding protein family 3. (B) Cellular organization of Bucl8 and homology modelling of the OEP domains. Bucl8 protein schematic is shown above homology model of OEP domains generated with MODELLER. Three monomers, each containing two OEP domains, assemble to form a homotrimer. Shown from top to bottom are the cell-surface exposed loops, the β-barrel spanning the outer membrane and the α-barrel spanning the periplasmic space, corresponding to the predicted OEP domains. The two OEP domains from a single monomer are highlighted in orange and purple, and the remaining monomers are colored gray. Following the OEP domains, the CL region is predicted to be partially extracellular with an additional C-terminal non-collagenous domain.

In addition to the CL region, four Bucl proteins were predicted to contain putative domains proven to participate in pathogenesis in other bacterial species (Table 2, Fig 4A). Bucl3 contains a putative Talin-1 domain; Talin-1 is a cytoskeletal protein that binds and activates integrins in mammals and talin-1-integrin interaction links the cytoskeleton with the extracellular matrix, allowing cell adhesion and migration [48–50]. Bucl4 contained a Bac_export_1 domain (Bacterial export proteins, family 1; PF01311) found in members of type III secretion protein family, including the SpaR of *Shigella* and *Salmonella*, and the YscT of *Yersinia*. These proteins form the inner-membrane part of the needle complex, which transports bacterial effector proteins to afflict host cells. Bucl8 contained the OEP domain; the members of outer membrane efflux protein family (PF02321) form channels that allow export of various compounds, including anti-microbial agents, in Gram-negative bacteria across the outer membrane [51]. Bucl13 contained a SBP_bac_3 domain (Bacterial extracellular solute-binding proteins, family 3; PF00497), which is found in periplasmic proteins that bind specific solutes within the periplasmic space and are often associated with ABC-type transporters [52].

Signal sequences were predicted in Bucl proteins 3, 4, 8, and 15, additionally supporting extracellular location for Bucl4 and Bucl8 (Table 2). Most Bucl proteins had transmembrane regions, interestingly, often associated with the CL regions (Table 2).

### Modeling of the OEP domains in Bucl8

The OEP domains found in Bucl8 are inferred in the formation of an efflux pump, thus, contributing to multi-drug resistance of Bp and Bm species [53]. Two tandem OEP domains were predicted with high confidence (E-values 7x10^-22^ and 4.5x10^-18^). The Bucl8 is also predicted to be a lipoprotein with an amino-terminal lipid-binding cysteine residue and a transmembrane region predicted with TMpred [54].

HMM search in the PDB database using Bucl8-OEP region as a query identified closest similarity (E-value = 6.6x10^-53^) to the drug discharge outer-membrane lipoprotein OprM of *P*. *aeruginosa* [55, 56]. Using OprM structure as a template (pdb code 3d5k, sequence identity 27%), the model of Bucl8 was generated with MODELLER 9 v.9 [57].

The OEP domains of Bucl8 form a trimeric structure containing the characteristic α-barrel, which spans the periplasmic space, and the β-barrel, which spans the outer membrane (Fig 4B). In OprM, the β-barrel is known to anchor the protein to the outer membrane, and also contains a series of surface exposed loops that are involved in constriction of the β-barrel pore, thereby preventing influx of xenobiotics at the resting state [56, 58]. The α-barrel contains an arrangement of twelve short helices and six long helices that form a bundle which is constricted at both ends but contains a bulge in the middle that can accommodate antibiotics. Twisting of the helices to loosen the pores forms a funnel-channel structure allowing for the active transport of antibiotics across the outer membrane outside of the bacterial cell [56].

The *bucl8* gene was found in all Bp and Bm strains tested by PCR and bioinformatics (Table 4), signifying the potential importance of Bucl8-efflux pump in the survival and pathogenesis of these species. Interestingly, all Bt strains analyzed contained DNA sequence homologous to the OEP-domain of Bucl8 in Bp and Bm but lacked the sequence corresponding to the Bucl8-collagenous domain; thus, it could not be recognized as a true Bucl. Additionally, a single nucleotide insertion at position 52, directly preceding the OEP-encoding region, was found, causing a frameshift mutation, which resulted in an altered amino acid downstream sequence.

**Table 4:** Distribution of all *bucl* genes in *Burkholderia spp*. as assessed by bioinformatics and PCR amplification^a^.

	*bucl* No.												
StrainAbbreviation	1	2	3	4	5	6	7	8	10	13	14	15	16
BpBP006^b^	+	+	+	+	+	+	+	+	+	+	+	+	+
Bp79^b^	+	+	+	+	+	+	+	+	+	+	+	+	+
Bp146^b^	+	+	+	+	+	+	+	+	+	+	+	+	+
Bp511^b^	+	+	+	+	+	+	+	+	+	+	+	+	+
Bp520^b^	+	+	+	+	+	+	+	+	+	+	+	+	+
Bp20B16^b^	+	+	+	+	+	+	+	+	+	+	+	+	+
Bp78^b^	+	+	+	+	+	+	+	+	+	+	+	+	+
Bp1026b^b^	+	+	+	+	+	+	+	+	+	+	+	+	+
BpE203	+	+	+	+	+	+	+	+	+	+	+	+	+
Bp4845	+	+	+	+	+	+	+	+	+	+	+	+	+
Bp1152	+	+	+	+	+	+	+	+	+	+	+	+	+
Bp1992	+	+	+	+	+	+	+	+	+	+	+	+	+
BpE8	+	+	+	+	+	+	+	+	+	+	+	+	+
Bp423	+	+	+	+	+	+	+	+	+	+	+	+	+
Bp6068	+	+	+	+	+	+	+	+	+	+	+	+	+
BpS13	+	+	+	+	+	+	+	+	+	+	+	+	+
Bp1710a	+	+	+	+	+	+	+	+	+	+	+	+	+
Bp1710b^b^	+	+	+	+	+	+	+	+	+	+	+	+	+
BpK9624^b^	+	+	+	+	+	+	+	+	+	+	+	+	+
Bp1106b	+	+	+	+	+	+	+	+	+	+	+	+	+
BpCh3	-	NT	NT	-	+	+	+	+	NT	+	+	+	+
Bp121	+	+	+	+	+	+	+	+	+	+	+	+	+
Bp1112	+	+	+	+	+	+	+	+	+	+	+	+	+
Bp305^b^	+	+	+	+	+	+	+	+	+	+	+	+	+
Bp668^b^	+	+	+	+	+	+	+	+	+	+	+	+	+
Bp406e	+	+	+	+	+	+	+	+	+	+	+	+	+
Bp1106a^b^	+	+	+	+	+	+	+	+	+	+	+	+	+
Bp5855	+	+	+	+	+	+	+	+	+	+	+	+	+
Bp5848	+	+	+	+	+	+	+	+	+	+	+	+	+
Bp5858	+	+	+	+	+	+	+	+	+	+	+	+	+
Bp0134a	+	+	+	+	+	+	+	+	+	+	+	+	+
Bp0303a	+	+	+	+	+	+	+	+	+	+	+	+	+
Bm10229^b^	+	-	+	+	+	+	+	+	+	+	+	+	+
Bm10247^b^	+	+	+	+	+	+	+	+	+	+	+	+	+
BmA188^b^	+	-	-	+	+	+	+	+	+	+	+	+	+
BmA193^b^	+	-	-	+	+	+	-	+	+	+	+	+	+
Bm10399^b^	+	+	+	+	+	+	+	+	+	+	+	+	+
BmPRL20^b^	+	-	+	+	+	+	+	+	+	+	+	+	+
Bm6^b^	+	-	+	-	+	+	+	+	+	+	+	+	+
Bm11^b^	+	+	+	+	+	+	+	+	+	+	+	+	+
BmSAVP1^b^	-	+	+	-	+	+	-	+	+	+	+	+	+
BmGB3	+	-	-	-	+	+	+	+	+	+	+	+	+
BmGB4	+	+	+	-	+	+	+	+	+	+	+	+	+
BmISU	+	+	+	+	+	+	+	+	-	+	+	+	+
BmTurk1^c^	+	+	+	+	+	+	+	+	+	+	+	+	+
Bm234	+	+	+	+	+	+	+	+	-	+	+	+	+
Bm235	+	+	+	+	+	+	+	+	-	+	+	+	+
BmHI533	+	+	+	+	+	+	+	+	-	+	+	+	+
BmGB11	+	-	+	+	+	+	+	+	+	+	+	+	+
BmNBL7	+	+	+	+	+	+	+	+	-	+	+	+	+
BmGB8	+	+	+	+	+	+	+	+	-	+	+	+	+
Bm23344^b^	+	+	+	+	+	+	+	+	-	+	+	+	+
BmTurk2^c^	+	+	+	-	+	+	+	+	+	+	+	+	+
BmFMH	+	+	+	+	+	+	+	+	-	+	+	+	+
Bm21280^b^	+	+	+	+	+	+	+	+	-	+	+	-	+
Bm85567	+	+	+	+	+	+	+	+	+	+	+	+	+
Bm2700C	+	-	+	+	+	+	+	+	+	+	+	+	+
BmCh7	+	+	+	+	+	+	+	+	-	+	+	+	+
BmCh5	+	+	+	+	+	+	+	+	+	+	+	+	+
Bm10230	+	-	+	+	+	+	+	+	+	+	+	+	+
BmGB8	+	+	+	+	+	+	+	+	-	+	+	+	+
Bt21723^b^	-	-	+	+	-	-	-	-	-	-	-	-	-
BtH0587^b^	-	-	+	+	-	-	-	-	-	-	-	-	-
BtE444^b^	-	-	+	+	-	-	-	-	-	-	-	-	-
Bt121^b^	-	-	+	+	-	-	-	-	-	-	-	-	-
BtE555^b^	-	-	+	+	-	-	-	-	-	-	-	-	-
Bt43^b^	-	-	+	+	-	-	-	-	-	-	-	-	-
Bt4^b^	-	-	+	+	-	-	-	-	-	-	-	-	-
BtTXDOH^b^	-	-	+	+	-	-	-	-	-	-	-	-	-
BtE264^b^	-	-	+	+	-	-	-	-	-	-	-	-	-
BtDW503	-	-	+	+	-	-	-	-	-	-	-	-	-
BtE421	-	-	+	+	-	-	-	-	-	-	-	-	-
BtE426	-	-	+	+	-	-	-	-	-	-	-	-	-
**Fisher p-value**	**<0.0001**	**<0.0001**	**0.581**	**0.353**	**<0.0001**	**<0.0001**	**<0.0001**	**<0.0001**	**<0.0001**	**<0.0001**	**<0.0001**	**<0.0001**	**<0.0001**
**Cramer’s V** ^**2**^	**0.829**	**0.486**	**0.009**	**0.018**	**1**	**1**	**0.829**	**1**	**0.426**	**1**	**1**	**0.908**	**1**
Bc706	-	-	-	-	NT	NT	NT	NT	-	NT	NT	NT	NT
Bc709	-	-	-	-	-	-	-	-	-	-	-	-	-
Bc710	-	-	-	-	-	-	-	-	-	-	-	-	-
Bce6656	-	-	-	-	-	-	-	-	-	-	-	-	-
BceBC7	-	-	-	-	-	-	-	-	-	-	-	-	-
BceK562	-	-	-	-	-	-	-	-	-	-	-	-	-
BceJ2315	-	-	-	-	-	-	-	-	-	-	-	-	-
Bce103a2	-	-	-	-	NT	NT	-	NT	-	NT	-	NT	-
BmvCF2	-	-	-	-	-	-	-	-	-	-	-	-	-
BmvCGD1	-	-	-	-	-	-	-	-	-	-	-	-	-
BmvCGD2	-	-	-	-	-	-	-	-	-	-	-	-	-
BmvCF1	-	-	-	-	-	-	-	-	-	-	-	-	-
Bmv17616	-	-	-	-	-	-	-	-	-	-	-	-	-
Bmv13010	-	-	-	-	-	-	-	-	-	-	-	-	-

^a^ Presence or absence of *bucl* amplicons are indicated by + or –, respectively. NT, not tested (not sufficient amount of gDNA available). Association of *bucl* presence with pathogenic Bp and Bm species, compared to nonpathogenic Bt strains, was assessed using the Fisher Exact Probability Test and Cramer’s V^2^ analysis.

^b^ Strains for which *bucl* presence was determined by bioinformatics.

^c^ Smaller-sized amplicons observed for *bucl2* amplicon (S4A Fig).

### Phylogenetic analyses of *bucl* genes

To better understand the relationship of *bucl* genes among *Burkholderia* spp., parsimony and model-based phylogenetic analyses were performed. All 13 *bucl* sequences, originally identified in collagen Pfam database, were BLASTn-searched against completed genomes of Bp, Bm, and Bt, and each *bucl* sequence was downloaded. The 13 *bucl* genes demonstrate no sequence similarity, indicating these are non-homologous genes, whereas alleles encoding the same *bucl* gene were orthologous across species. Nucleotide sequence alignments were generated for each *bucl* gene present in 13 Bp and 11 Bm strains; analysis of *bucl3* and *bucl4* also included 9 Bt strains (S1 data set). Pairwise alignments of each *bucl* among the different strains revealed that percent identities ranged from 42%-100%, with the average percent identity for each *bucl* ranging from 76.5–94.9% (S2 data set). In general, the non-collagenous regions of *bucl* genes were conserved, while the CL regions showed significant length polymorphisms. Consequently, the *bucl1* phylogeny based on non-CL region sequence produced a star pattern, while the *bucl1* phylogeny generated based on the entire *bucl1* sequence showed more extensive branching patterns, most of which were supported by Bayesian Posterior Probability values and several of which were also supported by maximum parsimony bootstrap values (S1 Fig). The CL region of *bucl1* encodes a single GAN-repeat type, therefore, the only difference between *bucl1* alleles from different strains represented in this tree arises from different GAN-repeat numbers. Since this is a common feature of all *bucl*s, and incorporation of these regions would likely lead to long branch attraction, only the non-CL regions were used in further analyses. Multiple sequence alignments of *bucl* genes 2, 5, 6, 7, 10, 13, 14, 15 and 16 showed highly conserved nucleotide sequence, similar to *bucl1*, therefore phylogenetic analysis was not performed.

**Fig 5:**
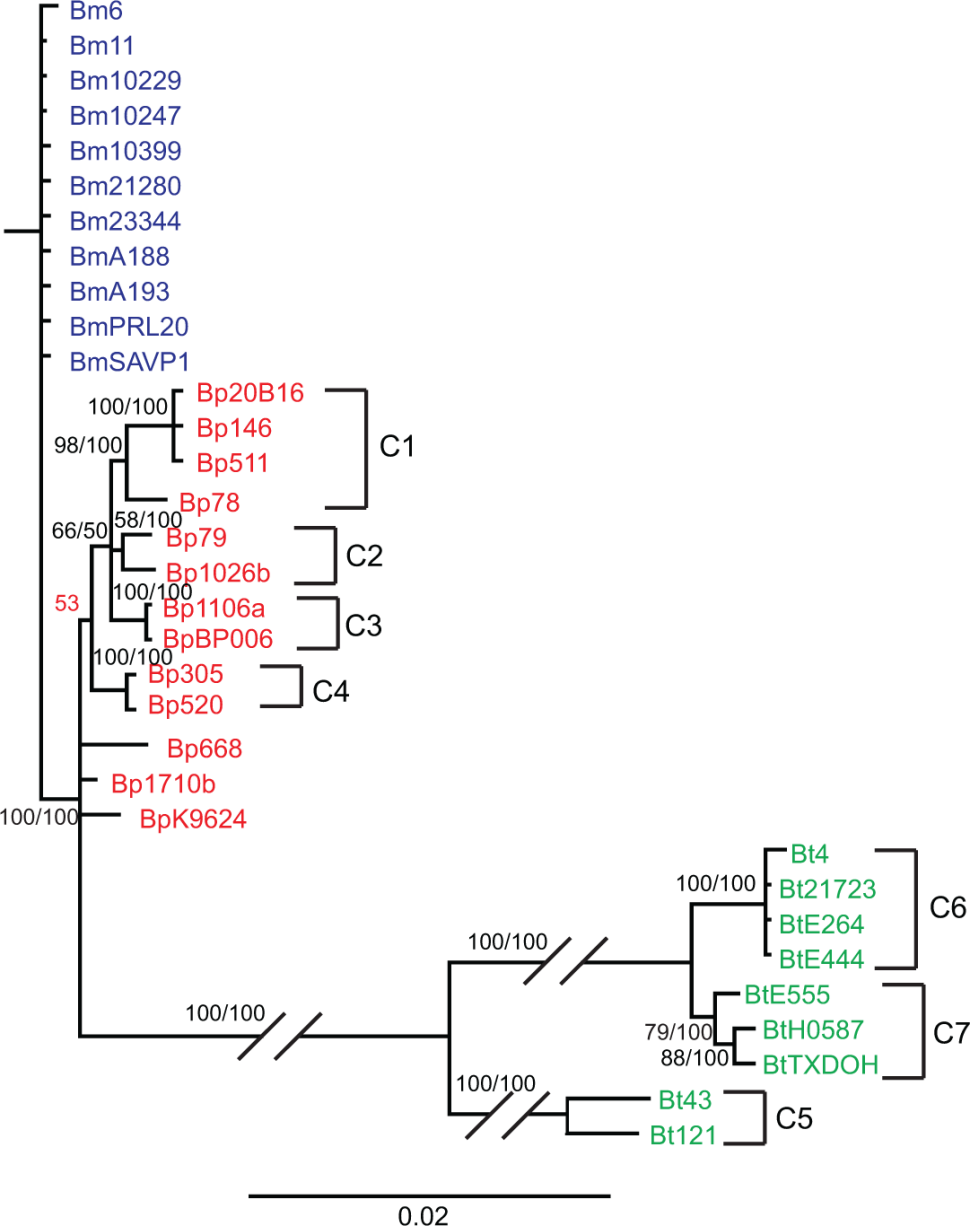
Phylogenetic analysis of *B*. *pseudomallei*, *B*. *mallei*, and *B*. *thailandensis* strains by *bucl*-locus typing. Bayesian analysis was performed on concatenated nucleotide sequences of the non-collagenous regions of *bucl3*, *bucl4*, and *bucl8* present in a set of 13 *B*. *pseudomallei*, 11 *B*. *mallei*, and 9 *B*. *thailandensis* strains (as shown in Table 3). Support values for each branch are shown as posterior probability from Bayesian analysis and bootstrap values from maximum parsimony analysis, respectively (PP/MP). Posterior probability value which was not supported by maximum parsimony analysis is shown in red. Phylogenetic Clusters 1–4 (C1-C4) correlated with geographic location of *B*. *pseudomallei* strains, whereas Clusters 5–7 (C5-C7) contained *B*. *thailandensis* strains that made up a separate branch from *B*. *pseudomallei* and *B*. *mallei* strains. Scale bar is representative of evolutionary distance in substitutions per nucleotide.

**Fig 6:**
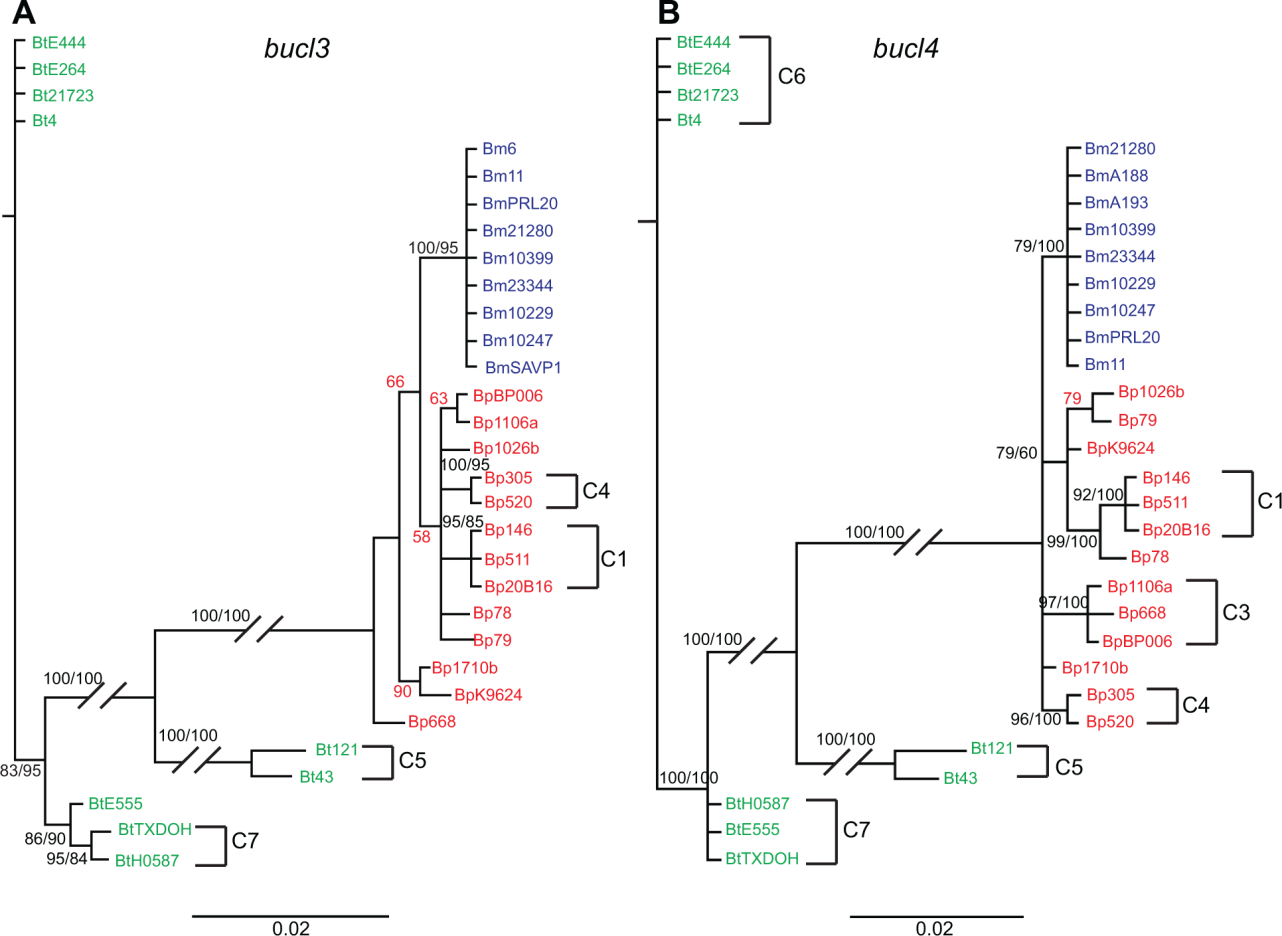
Phylogenetic analysis of *B*. *pseudomallei*, *B*. *mallei*, and *B*. *thailandensis* strains using individual *bucl3* and *bucl4* genes. Bayesian analysis was performed on nucleotide sequences of non-collagenous regions of a set of *Burkholderia* strains described in Table 3. Support values for each branch are shown as posterior probability from Bayesian analysis and bootstrap values from maximum parsimony analysis, respectively (PP/MP). Posterior probability values not supported by parsimony analysis are shown in red. Scale bar is representative of evolutionary distance in substitutions per nucleotide. Several clusters of strains corresponding to those observed in the concatenated analysis, C1-C7 in Fig 5, were also observed in the individual trees.

Phylogenetic trees were generated for individual and concatenated *bucl3*, *bucl4*, and *bucl8*, as these genes were present in all three species and contained the most informative characters. We included the OEP-encoding sequence of *bucl8* from Bt strains in this analysis, despite the lack of CL-encoding sequence and conserved frameshift mutation, because of significant sequence similarity to *bucl8*-OEP sequences shared with Bp and Bm. The phylogeny generated from concatenated sequences showed similar associations as phylogenies for the individual genes, although usually with higher statistical support. All analyses showed Bp and Bm strains were more closely related to each other than to Bt strains, which formed a main separate branch (Figs 5 and 6, S2 Fig). This observation is consistent with the hypothesis that the pathogenic Bp and Bm strains diversified from Bt [42, 59–61]. On the concatenated tree, Bm strains formed a single clade without further resolution that was strongly supported by both Bayesian posterior probability (PP, 100) and maximum parsimony (MP, 100) bootstrap values (Fig 5). This observation indicates either inadequate time for the diversification of Bm strains or purifying selection for the retention of nucleotide identity due to importance in adapting to its host pathogen niche [41]. In contrast, Bp strains exhibited higher diversification as shown by the presence of multiple clades. Four supported clusters were observed, two of which, Cluster 1 and Cluster 4, showed geographical associations as these strains were all Australian isolates. Cluster 1 (PP 98, MP 100) contained Bp strains 20B16, MSHR146, MSHR511, and NCTC 13178, all isolates obtained from Australia. Cluster 2 (PP 58, MP 100) contained Bp strains NCTC13179 and 1026b, isolated from human infections in Australia and Thailand, respectively. Cluster 3 (PP 100, MP 100) contained Bp strains 1106a and BPC006, obtained from northeast Thailand and China, respectively. Finally, Cluster 4 (PP 100, MP 100) contained Bp strains MSHR305 and MSHR520, which are both human infection isolates from Australia. Clusters 1 and 4 were also supported by trees based on individual *bucl3*, *bucl4*, and *bucl8* genes, although strain NCTC 13178 as part of Cluster 1 was only supported by the tree based on *bucl4* (Fig 6, S2 Fig). Similar to Bp, Bt strains showed significant diversification as evidenced by the formation of three supported clusters in the concatenated tree. These clusters were numbered consecutively Cluster 5, Cluster 6, and Cluster 7 (Fig 5). Clusters 5 and 7 were supported by individual *bucl3* and *bucl4* phylogenies (Fig 6), while only Cluster 5 was supported by *bucl8* phylogeny (S2 Fig). Analysis performed using amino acid sequences of Bucl proteins generated phylogenetic trees with similar patterns, though the support values were lower (S3 Fig), indicating many of the nucleotide changes were synonymous.

Overall, most *bucl* genes were highly conserved among Bp and Bm with most of the variation occurring in the CL region due to differing numbers of GXY repeats. Variation in non-CL regions of *bucl3*, *bucl4*, and *bucl8* revealed divergence between Bt and select agents Bp and Bm, as well as diversification among Bt strains. Bp and Bm appear more closely related, but only Bp strains showed diversification across the *bucl* loci by the formation of multiple distinct clades with strong statistical support.

### Assessment of *bucl* distribution across *Burkholderia* spp.

In order to assess the distribution of *bucl* genes across *Burkholderia*, nucleotide BLAST searches were performed using *bucl*-gene sequences from the reference strain Bp K96243, as queries against completed genomes of 13 Bp, 11 Bm, and 9 Bt strains. All 13 *bucl* genes were present in all Bp genomes, while the majority of *bucl* genes were maintained within Bm genomes (Table 4). Up to three *bucl* genes were missing in 8 Bm genomes, which is consistent with the reduced genetic material in this species [41, 46]. In contrast, only complete open reading frames of *bucl3* and *bucl4* were present in Bt genomes, presumably encoding a lipoprotein with a putative Talin-1 domain and a type III secretion inner membrane protein (Table 2, Fig 4A), respectively.

**Fig 7:**
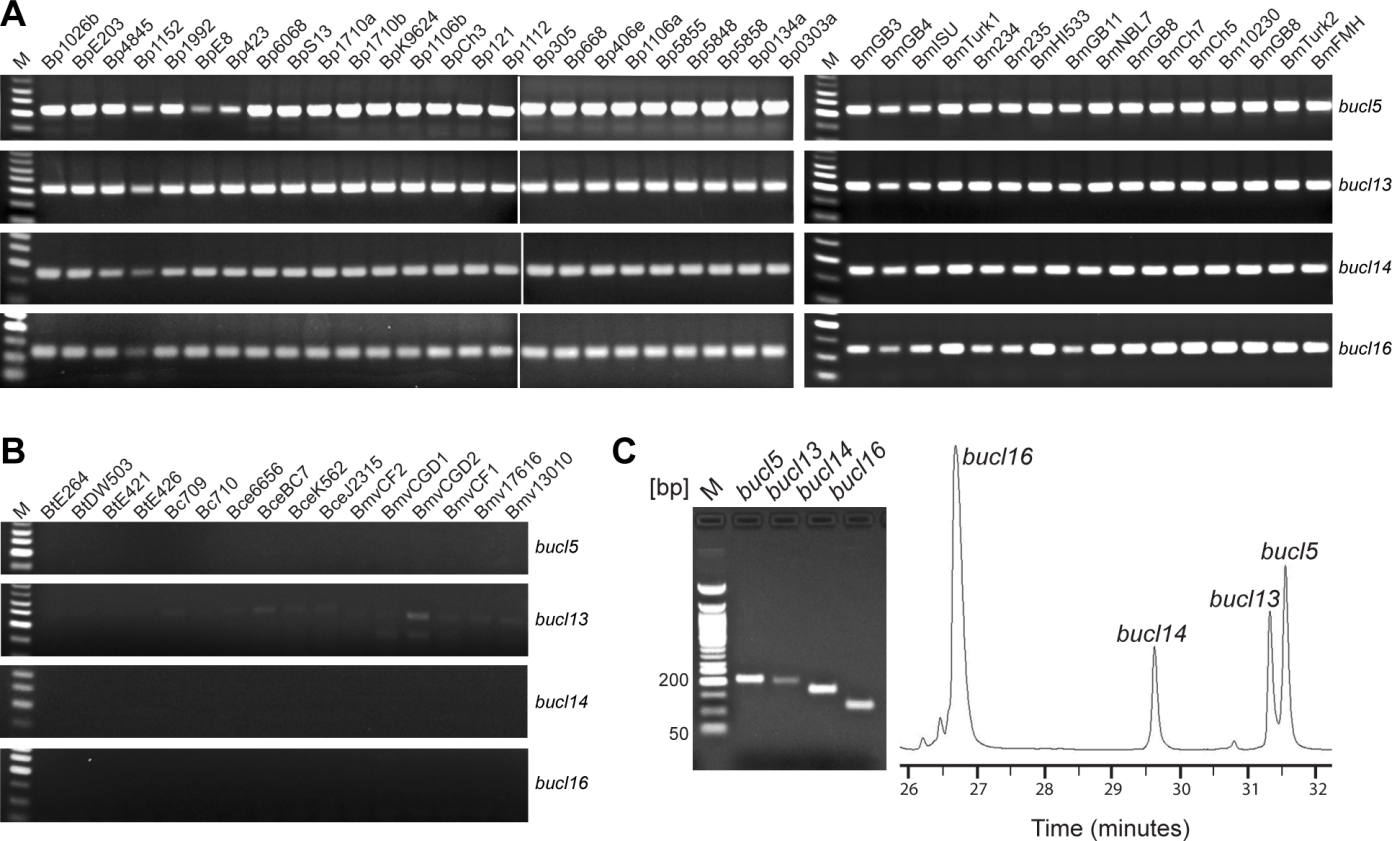
Distribution of *bucl* genes among *Burkholderia* spp. select agents by PCR. Presence of *bucl* genes was assessed by PCR on (A) a collection of genomic DNA from 25 *B*. *pseudomallei* and 16 *B*. *mallei* strains, as well as (B) in control strains of *B*. *thailandensis*, *B*. *cepacia*, *B*. *cenocepacia*, and *B*. *multivorans*; selected *bucl* genes *5*, *13*, *14*, and *16* are shown. (C) Detection and separation of selected *bucl* amplicons generated from the *B*. *pseudomallei* reference strain K96243 by traditional 2% agarose gel electrophoresis (left) or by capillary gel electrophoresis (right). Electropherogram generated by capillary gel electrophoresis with phospholipid nanogel matrix shows separation of amplicons over time. Amplicon sizes: *bucl5*, 216 bp; *bucl13*, 214 bp; *bucl14*, 178 bp; and *bucl16*, 123 bp. M, 50-bp DNA ladder. PCR data shown in Panel A for 25 Bp strains come from two merged gel images.

In addition to bioinformatic data, we tested distribution of *bucl* genes by standard PCR in a collection of genomic DNA from 25 Bp and 20 Bm strains, as well as the DNA from non-select agent controls 4 Bt, 3 *B*. *cepacia* (Bc), 5 *B*. *cenocepacia* (Bce), and 6 *B*. *multivorans* (Bmv) strains (Table 5, Table 6). Consistent with bioinformatic data, virtually all 25 Bp strains were found to contain all 13 *bucl* genes, with the exception of strain China 3 (BpCh3) which was missing *bucl1* and *bucl4* (Table 4, Fig 7, S4 Fig). Almost all Bm strains tested (15 out of 20) were lacking up to three *bucl* genes, in agreement with bioinformatic results. We calculated *bucl* frequencies as the proportion of Bp and Bm strains positive for each *bucl*, as tested by both PCR and bioinformatics. High frequencies were observed for *bucl3*, *bucl4*, *bucl7*, and *bucl15* (0.90–0.98), while lower frequencies were observed for *bucl2* (0.85) and *bucl10* (0.82). The *bucl2* and *bucl10* genes were most frequently absent from Bm strains, missing in about one-third of strains analyzed, indicating these genes are nonessential for Bm survival in mammalian host. Finally, all Bt strains contained only *bucl3* and *bucl4*, while no amplification of these two *bucl* genes was obtained for other control *Burkholderia* spp. (Table 4, Fig 7, S4 Fig).

**Table 5:** Primers and probe used for *bucl* amplification^a^.

*bucl*#	Primer name	Primer sequence 5'-3'	Amplicon size
*bucl1*	Bucl1_4F	GTGGCGCTGGCGCATCGTGAACGGC	103 bp
* *	Bucl1_4R	CTTCGTCGGTTGCGTGTCGTCCGTTGC	
*bucl2*	Bucl2_1F	CGGCGTGCGACGGAA	133 bp
* *	Bucl2_1R	GCCCACTTCGCGATTCTTC	
*bucl3*	Bucl3_2F	CTGCTCGGCGGCCTGTCGGGTTCGG	166 bp
* *	Bucl3_2R	CGGGCGCGGTCGTCGTCGA	
*bucl4*	Bucl4_2F_ext	GACGAATTCATCCGCTTCATCGTG	176 bp
* *	Bucl4_2R_ext2	CCGCTGCGCATCGGGCCTTTCA	
*bucl5*	Bucl5_2F	AACTCGACGAACTCAACGCGAATCGAC	216 bp
* *	Bucl5_2R	GCGCGCCGTTCTTTCTAGCGCTGC	
*bucl6*	Bucl6_CL flank_F	AGGAGCGGCGCTTGCCGGGCG	115 bp^b^
* *	Bucl6_Clflank_2R	GAACGGCGACGGTCCGACGCAGC	
*bucl7*	Bucl7_2F	ATGGACACGACCACGCAGGACGGG	264 bp
* *	Bucl7_2R	CCAATGAACGGCCCGCGTCGCTTTC	
*bucl8*	Bucl8_2F	GCAGCTCGATTCGTGGAT	243 bp
* *	Bucl8_2R	AGGTGGTACGACAGGCTCAG	
	Bucl8_3F	CTACGCGCTCCTCGACATCGCGC	96 bp
	Bucl8_3R	TGCGTGCCGATGCCCGCGCGCA	
*bucl10*	Bucl10_1F	GCATGCGTTGGACACGA	109 bp
* *	Bucl10_1R	GCAACGTCGTCATCTCGTC	
*bucl13*	Bucl13_2F	GTTCGATTTCACGACGTACCGGCTCG	212 bp
* *	Bucl13_2R	CGTCGTCGTCGAAGTACAGCACGTC	
*bucl14*	Bucl14_1F	TCGGCACATCTGTCGCCGCGAACC	178 bp
* *	Bucl14_1R	CGTATGGCCGCCGTGTCGATCGG	
*bucl15*	Bucl15_1F	GATCGCTCGACGCGCCCGRCGTGC	95 bp^b^
* *	Bucl15_1R	CTAAAACCGCCGGCGYGCCGCGC	
*bucl16*	Bucl16_2F	CCGGCAGCACCGACTCGAGCGTGCG	123 bp
* *	Bucl16_2R	CGTCGTTCGMGCTCGCCGATCGCTCG	
	^c^Bucl16_5'FAM_3'IBQ	TCTGCA+CG+G+CG+GTG+AGCCGCTTCA	

^a^ Primers were designed to generate conserved amplicons within the non-collagenous region of each *bucl* gene. Primers for *bucl6* and *bucl15* were designed flanking the collagenous region, which varies in size among strains (S4B Fig). Primers Bucl8_3F/3R were used to generate amplicon from Bp K96243 reference strain, whereas primers Bucl8_2F/2R selectively amplify products from Bp and Bm gDNA and were used in large-scale PCR screening (S4B Fig).

^b^ Amplicon sizes expected for the Bp K96243 reference strain.

^c^ LNA probe for *bucl16* detection; + symbols precede LNA bases.

**Table 6:** Genomic DNA collection.

Species	Abbreviation	Strain	Isolate information			Source of DNA
			Alternative designations	Source of isolate	Year	
*B*. *pseudomallei*	Bp1026b	1026B		blood culture from 29-year old female rice farmer with diabetes milletus, Northeast Thailand, Sappasithiprasong hospital	1993	USAMRIID^a^
*B*. *pseudomallei*	BpE203	E203		Soil sample from Roi Et, Thailand	1997	USAMRIID
*B*. *pseudomallei*	Bp4845	NCTC4845	(S. 397, NRRL B-1112, CCEB 472)	Monkey, Singapore	1935	USAMRIID
*B*. *pseudomallei*	Bp1152	STW-115-2		water, Thailand	1965	USAMRIID
*B*. *pseudomallei*	Bp1992	STW-199-2		water, Thailand	1965	USAMRIID
*B*. *pseudomallei*	BpE8	E8		Soil sample obtained on road to Trakan Phuet Phon District, Ubon Ratchathani Thailand		USAMRIID
*B*. *pseudomallei*	Bp423	423		Blood culture, Cambodia	2008	USAMRIID
*B*. *pseudomallei*	Bp6068	Pasteur 6068	2002721763	Vietnam		BEI Resources^b^
*B*. *pseudomallei*	Bp13	S13		muicodal strain, environmental isolate, Singapore		BEI Resources
*B*. *pseudomallei*	Bp1710a	1710a		blood culture of 52-year old male rice farmer with diabetes milletus, Northeast Thailand	1996	BEI Resources
*B*. *pseudomallei*	Bp1710b	1710b		relapse of same patient infected with 1710a, blood culture, Northeast Thailand, Sappasithiprasong hospital	1999	BEI Resources
*B*. *pseudomallei*	BpK9624	K96243		female diabetic patient- Khon Kaen hospital, Northeast Thailand	1996	BEI Resources
*B*. *pseudomallei*	Bp1106b	1106b		relapse of same patient infected with 1106a- female rice farmer, pus aspirated from liver abscess, Northeast Thailand, Sappasithiprasong hospital	1996	BEI Resources
*B*. *pseudomallei*	BpCh3	China 3		septicemia of American soldier, Burma		BEI Resources
*B*. *pseudomallei*	Bp121	NBL 121	strain 286, MP-S	chronic melioidosis case, infection acquired while living in Far East, Louisiana, United States	1953	BEI Resources
*B*. *pseudomallei*	Bp1112	NRRL B-1112	strain S 397, CCEB 472	naturally infected lab monkey, Singapore	1935	BEI Resources
*B*. *pseudomallei*	Bp305	MSHR305		brain sample, fatal encephalomyelitis, Australia, Royal Darwin hospital	1994	USAMRIID
*B*. *pseudomallei*	Bp668	MSHR668		blood culture from 53-year old male patient with severe melioidosis encephalomyelitis, Darwin Australia	1995	USAMRIID
*B*. *pseudomallei*	Bp406e	406e		disseminated melioidosis patient, toe swab, Ubon Ratchathani province, Northeast Thailand	1988	USAMRIID
*B*. *pseudomallei*	Bp1106a	1106a		female rice farmer, Northeast Thailand, Sappasithiprasong hospital	1993	USAMRIID
*B*. *pseudomallei*	Bp5855	MSHR5855		Australia	2011	USAMRIID
*B*. *pseudomallei*	Bp5848	MSHR5848		inhalational melioidosis, Australia	2011	USAMRIID
*B*. *pseudomallei*	Bp5858	MSHR5858				USAMRIID
*B*. *pseudomallei*	Bp0134a	HBPUB 10134a		sputum, Thailand, Mahidol University	2010	USAMRIID
*B*. *pseudomallei*	Bp0303a	HBPUB 10303a		sputum, Thailand, Mahidol University	2011	USAMRIID
*B*. *mallei*	BmGB3	GB3	2002734306, 2002734311, strain A, NCTC120	Lister Institute, London	1920	USAMRIID
*B*. *mallei*	BmGB4	GB4	M4, 2002734304, strain 6, NCTC10248	human, Ankara, Turkey	1950	USAMRIID
*B*. *mallei*	BmISU	ISU		Iowa State University		USAMRIID
*B*. *mallei*	BmTurk1	Turkey 1	2000031065, #1 Turkey	Turkey, isolated by Dr. Linda Schlater	2003	USAMRIID
*B*. *mallei*	Bm234	KC234	2002721273, 3783	human, Burma- isolated via CA Gleisser Army Medical School	1956	USAMRIID
*B*. *mallei*	Bm235	KC235	2002721274	Fort Detrick, Maryland, United States	1956	USAMRIID
*B*. *mallei*	BmHI533	HI533	2000031304, 2000031281	human liver abscess drainage, Maryland, United States	2000	USAMRIID
*B*. *mallei*	BmGB11	GB11	NCTC 10245, 2002721275, China 5, ATCC10399	horse lung, Southern China	1949	USAMRIID
*B*. *mallei*	BmNBL7	NBL 7	China 7	Prep of B mallei China 7 derived from ATCC23344 via passage through several individuals		BEI Resources
*B*. *mallei*	BmGB8	GB8 horse 4		derivative of ATCC23344 passaged through horse and isolated from the lung as a single colony, Manitoba, Canada		BEI Resources
*B*. *mallei*	Bm23344	ATCC 23344		human post-mortem knee fluid, skin pustules and blood, Burma	1944	BEI Resources
*B*. *mallei*	BmTurk2	Turkey 2	T2	Turkey		BEI Resources
*B*. *mallei*	BmFMH	FMH		derivative of ATCC23344 passaged through human, laboratory acquired infection- blood	2000	USAMRIID
*B*. *mallei*	Bm21280	2002721280	KC1092, 52–236	Iran	1952	BEI Resources
*B*. *mallei*	Bm86567	86–567	India86-567-2, 2000031064	mule, East India		BEI Resources
*B*. *mallei*	Bm2700C	SR092700C				BEI Resources
*B*. *mallei*	BmCh7	China 7	NBL7	preparation produced directly from ATCC 23344	1942	BEI Resources
*B*. *mallei*	BmCh5	China 5	MM-A, NBL4	lung and nose of infected horse, Kweiyang, China	1942	BEI Resources
*B*. *mallei*	Bm10230	NCTC 10230	strain Ivan	horse with glanders, Hungary	1961	BEI Resources
*B*. *mallei*	BmGB8**	GB8 (atcc23344)		Laboratory passage of ATCC 23344 in mouse	1997	USAMRIID
*B*. *thailandensis*	BtE264	E264		rice field soil sample, Central Thailand		BEI Resources
*B*. *thailandensis*	BtDW503	DW503		Derived from E264; (ΔamrR-oprA) (Kms Gms Sms); rpsL (Smr), Central Thailand		BEI Resources
*B*. *thailandensis*	BtE421	E421		rice field soil sample from Ubon Ratchathani province, Northeast Thailand	2001	BEI Resources
*B*. *thailandensis*	BtE426	E426		rice field soil sample from Ubon Ratchathani province, Northeast Thailand	2001	BEI Resources
*B*. *cepacia*	Bc706	DD-706				BEI Resources
*B*. *cepacia*	Bc709	DD-709				BEI Resources
*B*. *cepacia*	Bc710	DD-710				BEI Resources
*B*. *cenocepacia*	Bce6656	LMG16656		sputum of cystic fibrosis patient, Edinburgh, United Kingdom	1989	BEI Resources
*B*. *cenocepacia*	BceBC7	BC7		sputum from 15-year old patient with "cepacia syndrome", Canada		Emory U^c^
*B*. *cenocepacia*	BceK562	K56-2		less antibiotic resistant derivative of BC7, Canada		Emory U
*B*. *cenocepacia*	BceJ2315	J2315		sputum from cystic fibrosis patient, Edinburgh, United Kingdom	1989	Emory U
*B*. *cenocepacia*	Bce103a2	DD-707				BEI Resources
*B*. *multivorans*	BmvCF2	CF2		sputum from cystic fibrosis patient, NIH Clinical Center		Emory U
*B*. *multivorans*	BmvCGD1	CGD1		sputum from chronic granulomatous disease patient, NIH Clinical Center		Emory U
*B*. *multivorans*	BmvCGD2	CGD2		blood from chronic granulomatous disease patient, NIH Clinical Center		Emory U
*B*. *multivorans*	BmvCF1	CF1		sputum from cystic fibrosis patient, Belgium		Emory U
*B*. *multivorans*	Bmv17616	ATCC 17616		soil sample, United States		Emory U
*B*. *multivorans*	Bmv13010	LMG13010	CCUG 34080, Lauwers Cepa 002, CIP 105495, DSM 13243, NCTC 13007	sputum of cystic fibrosis patient, Belgium	1992	BEI Resources

^a^ USAMRIID; United States Army Medical Research Institute of Infectious Disease.

^b^ BEI Resources; NIH Biodefense and Emerging Infections Research Resources Repository, NIAID, NIH.

^c^ Emory U; Dr. Joanna Goldberg, Emory University School of Medicine, Atlanta, GA.

We next evaluated the association of *bucl* presence with pathogenicity among Bp, Bm, and Bt strains. The Fisher Exact Probability Test and Cramer’s V analysis were performed on the number of *bucl* genes present and absent among two groups: 1) pathogenic Bp and Bm strains and 2) nonpathogenic Bt strains. The Fisher test provides a measure of the statistical significance between two groups, and Cramer’s V squared (V^2^) is a value, which measures the degree of association between two variables on a scale of zero (no association) to one (perfect association). The Fisher test showed significant differences between group 1 and 2 for all *bucl* genes, except for *bucl3* and *bucl4*, indicating the presence of collagen-like genes is significantly associated with pathogenic *B*. *pseudomallei* and *B*. *mallei* species as compared with non-pathogenic *B*. *thailandensis* (Table 4). Further calculation of Cramer’s V^2^ showed perfect association (V^2^ = 1) for *bucl* genes *5*, *6*, *8*, *13*, *14*, and *16* that were present in all Bp and Bm strains, while absent in all Bt strains. High V^2^ values were calculated for *bucl1* (V^2^ = 0.829), *bucl7* (V^2^ = 0.829), and *bucl15* (V^2^ = 0.908), indicating positive association with these *bucl* genes with pathogenic Bp and Bm, as compared with nonpathogenic Bt lacking them. The remaining *bucl* genes, 2, 3, 4, and 10, had little or no association with pathogenic Bp and Bm compared to Bt (V^2^<0.5). Hence, our statistical analyses strongly infer association between the presence of the majority of Bucl proteins and pathogenicity.

### Detection of *Burkholderia* select agents by analytical PCR

Four conserved amplicons generated from *bucl* genes that were uniformly found in all Bp and Bm strains, but were absent in Bt, Bc, Bce, and Bmv strains, were assessed for select agent detection by standard agarose gel electrophoresis and capillary gel electrophoresis: *bucl5* (216 bp), *bucl13* (212 bp), *bucl14* (178 bp), and *bucl16* (123 bp) (Fig 7C). Size-identification of *bucl*-based amplicons by capillary gel electrophoresis was performed in a 10% phospholipid nanogel, allowing near single base pair resolution [62], including *bucl5* and *bucl13* amplicons that differ by 4 bp. Sizing of the target DNA fragments was accomplished by linear regression analysis for DNA size (in bp) versus migration time. The *bucl* gene amplicon sizes were calculated using the linear fit obtained for the migration times of internal standards with lengths of 100 bp and 250 bp, and the standard deviation calculated from 5 replicate measurements. The bias is calculated as the difference between the true fragment size and the measured size. Sizing results are reported as follows for n = 5 separations: [gene name (true size): calculated size ± standard deviation, percent relative size bias defined as bias divided by the true size]; *bucl5* (216 bp): 218 ± 2 bp, 0.9%; *bucl13* (212 bp): 215 ± 1 bp, 1%; *bucl14* (178 bp): 181 ± 1 bp, 2%; *bucl16* (123 bp): 120 ± 1 bp, 2%.

### Detection of *Burkholderia* select agents by quantitative PCR

**Fig 8:**
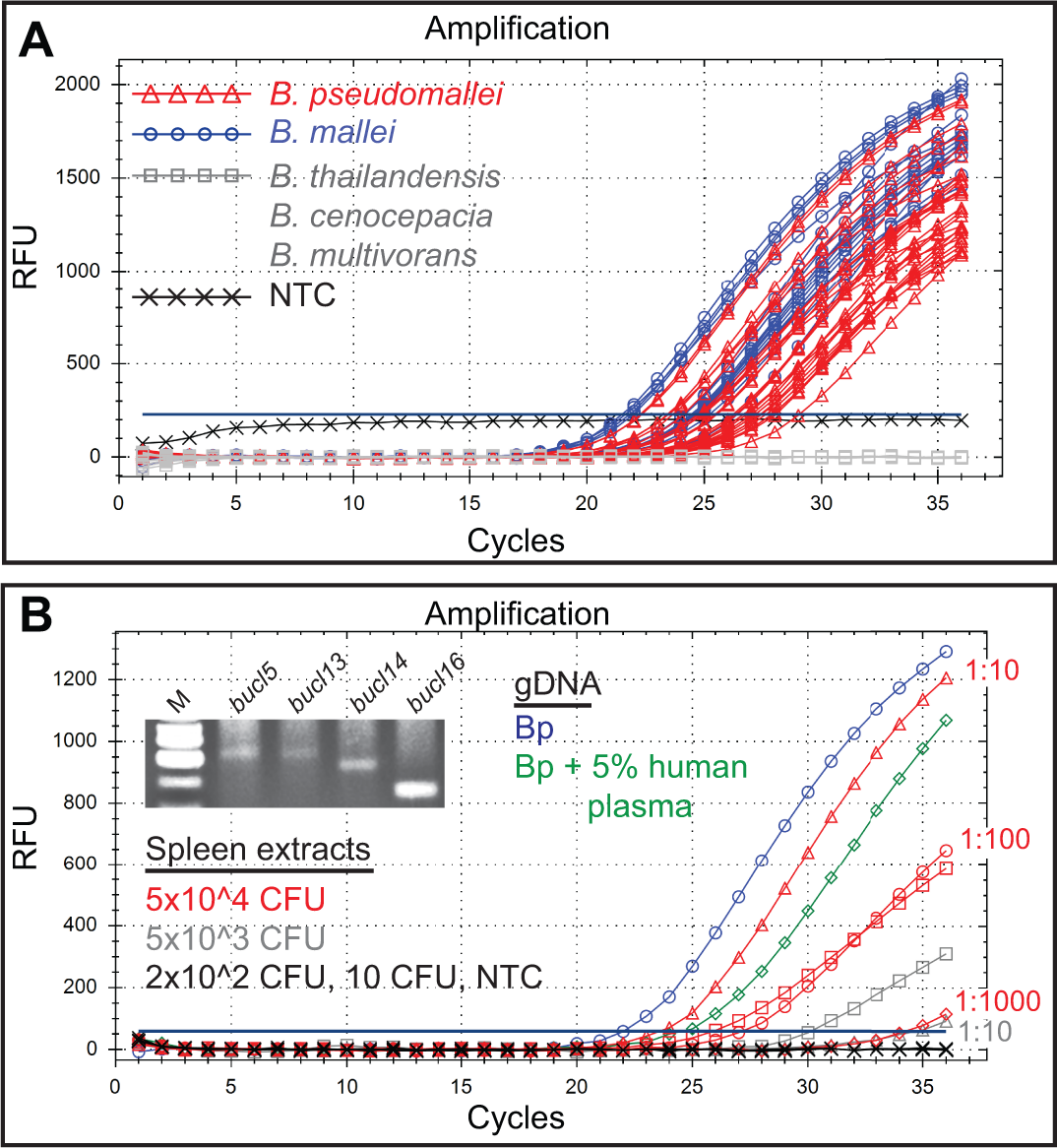
Detection of *B*. *pseudomallei* and *B*. *mallei* by qPCR. (A) Real-time qPCR detection of *bucl16*-gene target. Genomic DNA of 25 *B*. *pseudomallei* (red) and 15 *B*. *mallei* strains (blue), and control DNA from 4 *B*. *thailandensis*, 4 *B*. *cenocepacia*, and 6 *B*. *multivorans* strains (gray). (B) qPCR detection of *bucl16* target in the presence of human plasma and in spleen extracts from infected mice. 25 ng of gDNA from Bp K96243 was used as a positive control (blue line). Amplification of *bucl16* in qPCR reaction spiked with 5% human plasma is shown (green line). Mice were infected with Bp HBPUB10134a and CFU counts used in each qPCR reaction were based on plating spleen extracts on blood agar. Positive amplification is shown for spleen samples with 5x10^4^ CFU (red lines: square, undiluted; triangle, 1:10 dilution; circle, 1:100 dilution; diamond, 1:1000 dilution) and 5x10^3^ CFU (gray lines: square, undiluted; triangle, 1:10 dilution), while no amplification was obtained for crude spleen samples with original 2x10^2^ CFU and 10 CFU per reaction and no template control, NTC (black lines). Inset; amplification of *bucl* markers *5*, *13*, *14*, and *16* by standard PCR using crude spleen samples containing 5x10^4^ CFU per reaction. M, 50-bp DNA ladder.

Identification of molecular targets for *Burkholderia* select agents is challenging due to the high genomic plasticity reported in these organisms that include significant genomic rearrangements and deletions. PCR assays developed for *Burkholderia* detection include BurkDiff, a dual-probe assay able to detect and differentiate Bp and Bm [63, 64], and the TTS1 assay targeting *orf2* of type three secretion system I, detecting Bp only [64–66]. Here, we developed a qPCR assay for the detection of Bp and Bm based on *bucl16* target. A locked nucleic acid hydrolysis probe specific for *bucl16* gave robust amplification using DNA of Bp K96243 (Cq = 21.85±1.37). This probe was then tested against the genomic DNA collection, providing amplification of all Bp and all Bm strains, with no amplification from non-select agent controls including Bt, Bce, and Bmv, as well as a no DNA template control (Fig 8A). 30 ng of DNA was used for each strain and Cq values ranged from 23.42–29.05.

We next tested the *bucl16*-based qPCR assay towards detection of an infection with *Burkholderia* select agents by employing samples spiked with human plasma, and with samples obtained from experimental animals. PCR reactions performed with 30 ng Bp K96243 DNA and spiked with 5% human plasma produced positive amplification with average Cq = 24.28±2.14 (Fig 8B), whereas reactions spiked with 10% and 20% human plasma produced averaged Cq = 25.89±1.76 and Cq = 27.96±1.82, respectively.

Next, Bp strain HBPUB10134a was used for the detection of *Burkholderia* infection *in vivo*. Our recent studies have shown that Bp HBPUB10134a was the most virulent in the intraperitoneal infection model among a panel of 11 Bp strains, with an LD_50_ of 10 CFU at day 21 post-infection [67]. Following the injection, mice presented common clinical manifestations, including abscess and pyrogranuloma formation in the spleen and liver, and in some cases lesions and inflammation in the eyes and tail. A common pathological observation was the loss of rear limb function occurring between 6 and 30 days post-infection, associated with the pyrogranulomatous inflammation in the skin, skeletal muscle, bone, and peripheral nerves in the hind limbs. Here, mice that were injected intraperitoneally, were euthanized and sampled after 3, 7, or 14 days postinfection. Homogenized spleen samples were plated on blood agar to assess bacterial loads and 1 μL samples of irradiated sterile spleen extracts were used directly in qPCR reactions. Four samples, with original bacterial loads of 5x10^7^, 5x10^6^, 2x10^5^, and 10^3^ CFU/ mL, thus, presumably corresponding to 5x10^4^, 5x10^3^, 2x10^2^, and 10 CFU per 1 μL added to each qPCR reaction, respectively, were tested using our *bucl16*-based assay. When crude spleen extracts were used in qPCR, positive detection was obtained for 5x10^3^ CFU and 5x10^4^ CFU samples with averaged Cq values of 29.49±1.67 and 26.39±1.71, respectively (Fig 8B). Importantly, we observed that 1:10 dilution of the sample containing 5x10^4^ CFU/ μL, resulted in improved amplification, as evidenced by lower Cq value (23.32±0.42), while 1:100 dilution resulted in similar amplification as undiluted crude sample (Cq = 27.23±1.10) (Fig 8B, red curves). Further 1:1000 dilution of spleen extract provided detection level as low as 50 CFU per reaction with a Cq value of 32.63 ±1.57. On the other hand, 1:10 dilution of the sample originally containing 5x10^3^ CFU/ μL resulted in poorer amplification (Cq = 32.66±2.46) than crude undiluted sample (gray curves). We think that crude spleen extracts contained varying levels of inhibitors that differentially affected amplifications in these two samples. Finally, in addition to *bucl16*, *bucl* genes *5*, *6*, *8*, *13*, and *14* that were found in all Bp and Bm strains are similarly good candidate markers for the development of diagnostic qPCR assays.

## Discussion

Traditionally, collagen has been associated with multicellular animals, although, the number of collagen-like proteins identified in bacterial genomes has recently increased with 2554 sequences currently (search on 04/12/15) deposited in the Pfam collagen data base. The distribution of these collagen-like proteins is not uniform, however; they are absent in some bacteria and are overrepresented in other species. Here, we identified and characterized a group of 13 discrete collagen-like proteins in *Burkholderia*, referred to as Bucl, which are largely found in the pathogenic Bp and Bm species. Furthermore, we found that *bucl* genes provided important clues on the genomic plasticity and evolution of *Burkholderia* select agents. We observed Bucl proteins contained domains that are known to be involved in pathogenesis and antibiotic resistance, including an outer membrane efflux protein which we modelled. Finally, we utilized *bucl* genes as detection targets and successfully detected Bp infection in a mouse model.

### Characterization of Bucl-CL Region

Collagen-like sequences, embedding the typical repetition of triplets of the type Gly-X-Y [2, 68–70] have been identified in all Bucl sequences. We observed that for each Bucl, one or two GXY types predominated the CL region. This limited variation in GXY content resembles that seen in Bcl proteins of *Bacillus anthracis* [32] but is in contrast to Scl proteins of *Streptococcus pyogenes*, whose GXY sequence varies significantly within the CL region [13]. Typical of prokaryotic collagens, these sequences do not contain triple-helix-stabilizing hydroxyprolines, since bacteria lack the prolyl-hydroxylase enzyme necessary for post-translational modification of Pro to Hyp. The highest triple helix stabilities were predicted for Bucl2, Bucl5, Bucl13 and Bucl15, within the range of 35–38°, which is similar to that of previously studied bacterial collagens as well as human collagen [11, 13, 71, 72]. Similar to the CL regions of other prokaryotic proteins, like Scls from *S*. *pyogenes* [7, 8], the CL regions of these proteins share the common characteristics of possessing charged residues GEX, GLE and GXR triplets, respectively (Fig 3, Table 2). Indeed, ion pairs play a major role in stabilizing the triple helix, with an enthalpic stabilization, which likely involves interactions of polar groups with an ordered hydration network [9, 10, 12]. Additionally, specific GXY triplets were found to have favorable enthalpy values, corresponding to increased hydrogen bonding potential, including GPE [71], which is a common GXY triplet in the Bucl5 CL region. These regions are likely to be of biological importance in establishing interactions with charged counterparts. Interestingly, bacterial collagens have been shown to have relatively high proline content, 20% in *S*. *pyogenes* and up to 40% in *B*. *anthracis* [11], especially in the X position [73], whereas Bucl proteins lack Pro residues; only Bucl5 contains GPE repeats, likely contributing to its predicted high stability. Other Bucl proteins with lower thermal stability may rely on the hydrophobic membrane environment for triple helix stabilization, as those were predicted to have transmembrane regions, especially within the CL regions. Stability predictions shown here were computed using long CL sequences, whereas some Bucl variants had short CL regions, which may not form triple helices. This is substantiated by the fact that few triplets may also exist in other folds e.g., G5 domain, whose structure presents a pseudo-triple helix [74]. In summary, while overall characteristics of the Bucl proteins we identified were similar to previously described bacterial collagen-like proteins, i.e., presence of collagenous and non-collagenous domains and length variation in collagen region, the GXY content observed in Bucls was unique and likely impacts the structural stability of the Bucl-CL triple helix.

### Characterization of Bucl non-collagenous domains and their inferred roles in *Burkholderia* pathogenesis

It has been observed that collagen-like proteins are often surface associated. Indeed, among 53 bacterial and viral collagen-like proteins analyzed in an initial genome-based study, 16 were annotated as cell-wall attached or membrane associated [73]. Additionally, surface expression of collagen-like proteins including Scls of *S*. *pyogenes*, PclA of *S*. *pneuomonia*, and Lcl of *L*. *pneumophila*, has been demonstrated experimentally [7, 8, 18, 19]. Structural predictions performed for Bucl proteins revealed that their majority, 10 out of 13, have transmembrane regions, supporting the location of Bucl proteins in the inner or outer membrane of *Burkholderia* spp. Moreover, four of these proteins were predicted to contain both signal sequences and transmembrane domains, further supporting surface association. Further non-collagenous features include well-conserved domains (in Bucl3, Bucl4, Bucl8 and Bucl13), which are inferred in pathogenesis.

Bucl3 was predicted to have a Talin-1 domain. Talin-1 in eukaryotes is known to bind and activate integrins as well as link the cell cytoskeleton to the extracellular matrix [50]. Cell-to-cell invasion by *Burkholderia* is largely achieved by the disruption of the host cytoskeletal network, as well as the fusion of host cells resulting in the formation of multinucleated giant cells, mediated mainly by type III and type VI secretion systems [75, 76]. The intra- and inter-cellular spread is facilitated by the formation of actin tails which propel the bacterial cells. Thus far, several type III secretion effector proteins are known to be involved with host actin polymerization allowing cell invasion, including BimA, BopE, and BipD [43]. The putative Talin-1 domain found in Bucl3 may also be involved in interactions with host actin that allow for cell invasion or the formation of actin tails during infection.

The Bucl4 protein is putative inner membrane protein part of the type III secretion T3SS-2 system [77]. There are three known type III secretion gene clusters (T3SS-1, T3SS-2, and T3SS-3) distributed among Bp, Bm, and Bt species. T3SS-1 is specific to Bp while T3SS-2 and 3 are found ubiquitously in all three species [78]. The T3SS-3 is known to be important for virulence in Bp [44, 75, 79], as mutants deficient in the T3SS-3 have reduced replication in host cells, and are unable to escape endocytic vacuoles, and to form membrane protrusions and actin tails [80]. The other two secretion systems are less well characterized, and the role of the T3SS-2 secretion system in pathogenesis is not known. The unique association of a collagenous domain with Bac_export_1 domain in this inner membrane protein of T3SS-2 has not been previously acknowledged.

Bucl13 contains the SBP_bac_3 domain, and is predicted to be a bacterial periplasmic solute binding protein. Binding of the solute causes a conformational change, which allows interaction of the solute with inner membrane proteins and subsequent transport of the solute into the cell. Family 3 solute-binding proteins are known to bind polar amino acids and opines [52], therefore Bucl13 is likely associated with amino acid transport; interestingly, Bucl13 is present in all Bp and Bm strains tested, while it is absent in non-pathogenic Bt. Bucl13 was also predicted to have a collagenous domain with a relatively high thermal stability, possibly contributing to its function.

Of particular interest is the Bucl8 protein, which was found to contain two tandem outer membrane efflux protein (OEP) domains that are known to contribute to the multidrug resistant phenotype of Bp and Bm species. These organisms are intrinsically resistant to multiple antibiotics including aminoglycosides, macrolides, and β-lactams [53, 81]. The outer membrane protein is an integral component of a tripartite Resistance-Nodulation-Division (RND) efflux pump that also requires an accessory protein in the periplasm and an inner membrane transport protein. It is known that there are 10 RND efflux pumps annotated in the Bp K96243 strain, many of which have not been explored [40]. Currently, only three of these systems, BpeAB-OprB, AmrAB-OprA, and BpeEF-OprC, have been investigated for their roles in multidrug resistance [82–84]. Interestingly, Bucl8 was found to be present in all Bp and Bm strains and absent in the non-pathogenic Bt, suggesting selective pressure for the Bucl8-OEP in human or animal infection. We homology-modeled the Bucl8-OEP region based on OprM protein of *Pseudomonas aeruginosa* and observed a trimeric arrangement forming an outer membrane-spanning β-barrel and periplasmic α-barrel. The presence of the CL domain in Bucl8 is an unexpected observation, as collagenous regions have not been reported as part of efflux pump systems. On the other hand, the trimeric arrangement of Bucl8 is consistent with the formation of a collagen triple helix. TMPred predicted a transmembrane region, albeit with lower score, for a part of the collagen-like region (amino acids 539–557) indicating the CL region folds back across the membrane. The CL region is then predicted to extend into the extracellular space, projecting the carboxyl-terminal region from the cell surface. The triple-helical CL region may have a number of functions: i) to project the C-terminal region, which may serve as a surface adhesin, ii) to stabilize the trimeric arrangement of the OEP, and iii) may assist in blocking the β-barrel pore at the resting state, thus, preventing entry of xenobiotics into the cell. Ongoing studies will determine the potential role of Bucl8-OEP in drug resistance, Bp and Bm pathogenesis, as well as a potential as vaccine candidate.

### Bucl phylogeny

The presence of 13 collagen-like genes in Bp and Bm genomes poses the question how have these unique sequences been acquired in *Burkholderia*? The GXY repeats found in bacterial collagens may have arisen through mechanisms including *de novo* spontaneous mutation and subsequent triplet repeat expansion independent within each gene, or by horizontal gene transfer. It has been initially suggested that collagen sequences are acquired by horizontal transfer from eukaryotes to prokaryotes based on the lack of collagen sequences in ancestral archaeal genomes and relatively few sequences identified in bacterial genomes [73]. However, current collagen Pfam contains 2,554 bacterial collagen sequences, as well as 14 archaeal. A recent study, focused on bacterial molecular mimics of host proteins, proposed that collagen-like sequences found in pathogens evolved independently to mimic human host proteins [85]. The uniformity of GXY content within each Bucl indicated they are likely to have evolved from the accumulation of repeats within each gene, resulting in diverse Bucl proteins that share the GXY motif but with different GXY composition. Additionally, gene-enrichment analysis showed that collagen-like proteins were related to extracellular matrix mimicry and cell adhesion, supporting the evolution of repetitive sequences in virulence factors. Our phylogenetic analyses show that 13 collagen-like genes observed in numerous Bp and Bm genomes are unrelated to each other, which supports their independent acquisition, as well as selective adaptation of their collagen-like sequences in the host environment. This is further supported by the lack of collagen-like proteins (11 out of 13) in the closely related environmental species of Bt, indicating these sequences were acquired after divergence of Bp and Bm from Bt. Since Bucl proteins are unrelated and encoded in various locations in the genome, within-gene expansion of GXY-repeat motifs may point to convergent evolution of collagenous sequences to fulfill a similar function.

Phylogenetic trees based on three *bucl* loci showed Bt strains formed a distinct separate branch from Bp and Bm strains. This is consistent with previous studies based on phylogenetic analyses of seven MLST loci [42] and over 11,000 SNPs [60] which showed Bp and Bt isolates were resolved into two groups that were supported in 100% of bootstrap replicates. We also observed Bm strains share high sequence similarity, while Bp strains exhibited more intraspecies diversity, forming more extensive clusters that often corresponded to geographical associations. Previous phylogeographic reconstruction of *Burkholderia* strains based on over 14,000 SNPs showed that Bp and Bm strains formed separate clusters. The same study also showed Bp strains were significantly divided between those originating from Australia and Asia [60], in agreement with our observation that Australian isolates formed distinct clusters in *bucl*-based phylogenetic trees.

### Bucl distribution

Both bioinformatic and PCR analyses showed that the majority of *bucl* genes are unique to Bp and Bm strains, with the exception of *bucl3* and *bucl4*. This observation may indicate these two genes are selected for in the environment of Bp and Bt, as several genomes of host-adapted Bm, lack these genes. The absence of most *bucl*s from Bt is a surprising observation since its genome is overall similar to that of Bp [59], which may suggest either the acquisition of *bucl*s in Bp or the loss of *bucl*s in Bt after divergence of the two species. Both Bp and Bt species have large genomes of approximately 7.2 and 6.7 Mb, respectively, divided between two chromosomes. Comparative genomics showed that Bp and Bt genomes share a large number of conserved genes involved in both core and accessory functions, while genes associated with virulence in Bp have increased diversity [59]. Interestingly, *bucl3* and *bucl4*, encoding proteins potentially involved in pathogenesis, are found in the avirulent Bt. It has been shown that 71% of virulence-related genes in Bp are conserved in Bt with similarities of over 80%, including type III secretion gene clusters [59]. Amino acid differences in virulence proteins present in both species may confer functional differences impacting virulence in Bp vs. Bt. Lastly, the presence of *bucl3* and *bucl4* alone in Bt was not sufficient to cause pathogenesis, a new biological trait acquired by Bp and Bm after the acquisition of additional virulence factors, including additional Bucls. A prominent feature of *Burkholderia* genomes is the presence of multiple horizontally acquired genomic islands that differ between Bp and Bt [40, 59]. These genomic islands are associated with survival in the soil environment and are absent in Bm genomes, possibly explaining why Bm cannot persist in the environment [46]. The presence of most *bucl* biomarkers in both Bp and Bm genomes indicates they are not located within genomic islands but are rather a part of the core genome.

Previously, it has been reported that Bm is a clonal derivative of Bp, which has evolved to adapt to the host environment. Multilocus sequence typing analyses show that, in contrast to Bp, Bm strains are genetically homogenous, while relatively few new genes are being identified, as additional genomes are sequenced [46]. However, the variable portion of the genome, though not acquiring new genetic information, is continuing to alter via expansion of IS elements and chromosomal rearrangements. Our phylogenetic analysis of *bucl* genes within Bm supports the genetic homogeneity among Bm strains, and mapping of *bucl* markers showed considerable chromosomal rearrangements occurring between Bm strains.

Different collagen-like proteins, unrelated to 13 Bucls characterized here, were also present in other *Burkholderia* species. We noticed these collagen-like proteins found in *B*. *cepacia*, *B*. *cenocepacia*, *B*. *multivorans*, *B*. *ambifaria*, *B*. *glumae*, *B*. *gladioli*, and *B*. *xenovorans*, contained GTS repeats within the CL region, similar to Bucl3. However, outside of the CL region, sequence identity was very low, therefore these proteins were not included in the Bucl3 group. Given the importance of *Burkholderia* species as human pathogens and part of the *Burkholderia cepacia* complex (*B*. *cepacia*, *B*. *cenocepacia*, *B*. *multivorans*, and *B*. *ambifaria*), plant pathogens (*B*. *glumae* and *B*. *gladioli*), and plant symbionts (*B*. *xenovorans*), investigation of these collagen-like proteins is an interesting area for further study.

### *bucl*-based infection detection

Bp and Bm are reported to have fatality rates up to 80% and 95%, respectively [86, 87], making early diagnosis and treatment critical for patient survival. Currently, culture-based identification of *Burkholderia* select agents remains the gold standard for diagnosis [86, 88]. Highly variable genomes present a challenge in finding reliable genetic targets that are not subjected to chromosomal deletion, especially for *B*. *mallei*. Although a few laboratory-developed qPCR tests have been reported, there are no FDA-approved assays for the detection of *Burkholderia* select agents. The TTS1 assay [65, 66] detects specifically Bp, and the BurkDiff assay [63] detects both organisms and differentiates them based on a SNP-associated shift of approximately 1 ΔCt [64]. Here, we assessed *bucl* markers as detection targets. Standard PCR performed on a large collection of gDNA yielded specific amplicons for *bucl5*, *13*, *14*, and *16* from Bp and Bm but not from the non-select agent controls. This PCR test with 4 *bucl* targets detected Bp infection in laboratory animals using spleen extracts as a specimen. Separation of these conserved amplicons by capillary electrophoresis in a phospholipid nanogel matrix allowed for size-based identification, similarly to previously described identification of *Aspergillus* spp. [31] and *Streptococcus pyogenes* [35]. Precise microfluidic separation could be used for strain fingerprinting based on multiplexed amplicons generated with primers flanking the repetitive CL region, as previously tested with *B*. *anthracis* strains [32]. Our achieved resolution <9 bp along a wide range of amplicon sizes will allow to differentiate two strains that differ by a single GXY repeat. We further developed a qPCR assay for *bucl16*, which detects both Bp and Bm; it was tested with purified genomic DNA templates, gDNA spiked with 5% human plasma, and spleen extracts from infected mice. The *bucl16* assay was able to detect as low as 50 CFU per reaction in diluted spleen samples; however, it should be noted that sample-to-sample variation was observed. The specimen type will also affect detection outcome. For example, sputum and pus typically contain high bacterial loads (10^2^−10^9^ CFU/ mL) [89], whereas blood of 45% of patients with septicemic melioidosis had less than 1 CFU/ mL bacteria in the blood [90], which presents a sensitivity challenge, even for highly performing qPCR assays. In as much as current work was focused on a single assay, which would simultaneously detect both select agents similarly to BurkDiff assay, the ongoing research is focused on the development of a probe-based qPCR assay targeting nucleotide polymorphisms identified in *bucl3* and *bucl4* genes. In summary, selected *bucl* genes represent promising detection targets as they are both specific to and ubiquitously found in Bp and Bm strains.

## Materials and Methods

### Ethics statement

#### Animal Studies

Animal research at the United States Army Medical Research Institute of Infectious Diseases (USAMRIID) was conducted under an animal use protocol approved by the USAMRIID Institutional Animal Care and Use Committee (approved by the USAMRIID-IACUC) in compliance with the Animal Welfare Act, PHS Policy, and other Federal statutes and regulations relating to animals and experiments involving animals. The facility where this research was conducted is accredited by the Association for Assessment and Accreditation of Laboratory Animal Care International (AAALAC) and adheres to principles stated in the Guide for the Care and Use of Laboratory Animals, National Research Council, 2011. Tissue samples used in this study were generated in a previously published work [67]. Briefly, challenged mice were observed at least daily for 14 days for clinical signs of illness. Humane endpoints were used during all studies, and mice were humanely euthanized when moribund, according to an endpoint score sheet. Animals were scored on a scale of 0–11: 0–2 = no significant clinical signs; 3–7 = significant clinical symptoms; such as subdued behavior, hunched appearance, absence of grooming, and impacted hind limb function and hind limb paralysis (increased monitoring was warranted and mice were checked at least twice per day); 8–11 = distress. Those animals receiving a score of 8–11 were humanely euthanized by CO_2_ exposure using compressed CO_2_ gas followed by cervical dislocation. The mice that were serially sampled were deeply anesthetized and then euthanized by exsanguination followed by cervical dislocation. However, even with multiple observations per day, some animals died as a direct result of the infection in between observation periods.

#### Human plasma collection

Anonymized human plasma samples were utilized in quantitative PCR experiments. Plasma samples were obtained from an already-existing collection, which was established by the corresponding author (SL; IRB Protocol Number: 1308076685). Collection of human blood of healthy adults was performed in accordance with the Human Research Protections Policy at West Virginia University. This study was approved by the Institutional Review Board at West Virginia University (IORG0000194) and written informed consent was obtained from all participants.

### Bioinformatic analyses

*Burkholderia* collagen-like proteins, designated Bucl, were identified by searching the Sanger Institute Pfam collagen database (PF01391). Bucl proteins found in *B*. *pseudomallei*, *B*. *mallei*, and *B*. *thailandensis* were categorized into 13 Bucl-protein types based on similar domain organization and primary sequence similarity. Next, nucleotide BLASTn search was performed using each of 13 *bucl*-gene sequence as a query against the NCBI Nucleotide collection (nr/nt) database, as well as whole genome shotgun contigs (wgs) database, to determine *bucl* distribution in completed *Burkholderia* spp. genomes. DNA analyses were performed using the Lasergene Core Suite v. 12 (DNASTAR, Inc., Madison, WI).

### Protein structure prediction and modeling

Domain organization of Bucl proteins was adapted from the Pfam collagen database [91] and verified independently using the Fugue 2.0 Server [92], which additionally identified the putative Talin-1 domain within Bucl3. Presence of a signal peptide was predicted with the hidden Markov model component of the SignalP 3.0 Server (http://www.cbs.dtu.dk/services/SignalP-3.0/) [93–95]. The presence of transmembrane domains was predicted with TMpred [96].

When possible, as in the case of Bucl8, a 3D model was generated by homology modeling. Best template was identified by employing profile hidden Markov models (profile HMMs) and the program HMMer [97]. Once the best template was identified (pdb code 3d5k, sequence identity 27%, residues 51–516), the model of Bucl8 outer membrane efflux protein (OEP) domains was generated using MODELLER 9V9 [57]. Stereo-chemical quality of the model was improved by energy minimization using GROMACS [98].

Thermal stability along the predicted triple helices of Bucl-collagen domains was assessed with an algorithm developed by Persikov *et al*. 2005 [47]. With this approach, a stability coefficient is assigned for every GXY triplet and averaged over a window of 5 tripeptide units. The averaged relative stability values are plotted against the tripeptide number in the collagen sequence.

### Phylogenetic analyses

Both individual (with and without the collagen-like domains) and concatenated nucleotide sequences were aligned with ClustalV in the Megalign module in DNASTAR Lasergene software, and verified manually. Maximum parsimony analyses were performed with 1000 bootstrap replicates using MEGA 6.06 [99], with the Tree-Bisection Reconnection heuristic search and 200 max trees saved. The evolutionary models used for each data-set were determined by MrModelTest 2.3 [100] with the Akaike Information Criterion (AIC). Bayesian analyses were performed within MrBayes 3.1.2 [101] implementing six Markov chains, 1000000 generations, with trees sampled every 100 iterations. Posterior probabilities were calculated using the last 20% of saved trees (burnin = 8000). Cutoff values for significance were assigned 95 for Bayesian analysis and 70 for maximum parsimony analysis. All phylogenetic trees were constructed using the majority rule consensus. Trees were viewed in FIGTREE v1.3.1 (http://tree.bio.ed.ac.uk/software/figtree/). Phylogenies were constructed based on single *bucl* genes as well as concatenated *bucl* genes.

### *bucl* distribution among *Burkholderia* species

*bucl* distribution was assessed in a broad collection of *Burkholderia* strains using genomic DNA (Table 2) obtained from: (i) NIH Biodefense and Emerging Infections Research Resources Repository, NIAID, NIH, (ii) Dr. Christopher Cote (The United States Army Medical Research Institute of Infectious Disease), and (iii) Dr. Joanna Goldberg (Emory University). The total collection consisted of DNA from 25 *B*. *pseudomallei* and 20 *B*. *mallei* strains, and non-select agent control DNA from 4 *B*. *thailandensis*, 3 *B*. *cepacia*, 5 *B*. *cenocepacia*, and 6 *B*. *multivorans* strains. Analytical PCR was performed with primers targeting conserved non-collagenous regions of *bucl* alleles present in the reference strain *B*. *pseudomallei* K96243. PCR buffer (10 mM Tris-HCl, 1.5 mM MgCl_2_, 50 mM KCl, pH 8.3) included 0.2 μM primers, 0.2 mM dNTP’s, and 1.5 M betaine (Sigma-Aldrich, St. Louis, MO) to ameliorate amplification problems associated with high GC content (~68%) of *Burkholderia* genomes [41]. A temperature gradient of 50–65°C was tested for each primer pair and gDNA of *B*. *pseudomallei* K96243 harboring all 13 *bucl* genes as a template; uniform amplification conditions were established for all *bucl* genes at an annealing temperature of 64°C. Amplification was performed with an in-house Taq polymerase as follows: 95°C, 5 min-[95°C 30 sec, 64°C 30 sec, 72°C 45 sec] x30 cycles- 72°C, 10 min. 40 ng of template DNA was used for screening genomic DNA collection and reactions were carried out on a Bio-Rad S1000 thermal cycler. Resultant PCR products were analyzed on a 2% agarose gel with a 50-bp ladder DNA standard (New England Biolabs Inc., Boston, MA). Gels were imaged using the Eagle Eye II (Stratagene, La Jolla, CA), and FOTO/ Analyst Investigator/ Eclipse gel documentation workstation (Fotodyne, Harland, WI).

### qPCR amplification of *bucl* targets

Testing of selected *bucl* amplicons by real-time PCR with SYBR green intercalating dye was performed to assess potential candidates for probe-based detection of *B*. *pseudomallei* and *B*. *mallei* species. Reactions were carried out with SsoAdvanced SYBR Green Supermix (Bio-Rad, Hercules, CA), 0.5 μM concentration of each primer and 25 ng of gDNA from strain *B*. *pseudomallei* K96243 as a template in a total volume of 20 μL. Amplification curves were obtained with the following program: 95°C, 3 min-[95°C 5 sec, 64°C 10 sec]x35 cycles. qPCR was performed using a Bio-Rad CFX96 instrument and data analyzed with the CFX Manager software Version 3.0. PrimeTime qPCR probe was developed for the *bucl16* gene, which yielded robust amplification in 5’ nuclease qPCR assays, to detect *B*. *pseudomallei* and *B*. *mallei* species. Locked nucleic acid (LNA) *bucl16*-based probe (Table 4) contained a 5’-FAM fluorophore and a 3’-Iowa Black fluorescent quencher. Reactions were carried out using SsoAdvanced Universal Probes supermix (Bio-Rad), 0.5 μM primers, 0.2 μM concentration of probe and 25 ng of gDNA template in a total volume of 20 μL. Amplification curves were obtained with the following program: 95°C 3 min-[95°C 5 sec, 64°C 10 sec]x35 cycles.

### Capillary gel electrophoresis

Reagents for separation of DNA by capillary gel electrophoresis included the nanogel matrix composed of the phospholipids dimyristoyl-*sn*-glycero-3-phosphocholine (DMPC) and 1,2-dihexanoyl-*sn*-glycero-3-phosphocholine (DHPC) (Avanti Polar Lipids, Alabaster, AL), 3-(N-morpholino)-propanesulfonic acid (MOPS) (Alfa Aesar, Ward Hill, MA) buffer, and SYBR green 1 (Life Technologies, Grand Island, NY). The phospholipid pseudogel was prepared at a molar ratio of [DMPC]/[DHPC] = 2.5 at 10% wt/vol in an aqueous solution of 100 mM MOPS buffer (pH 7) in order to generate the nanogel separation matrix. Intercalating dye was incorporated into the nanogel at 1x concentration to enable fluorescent DNA detection. The 50-bp DNA ladder (New England BioLabs, Ipswich, MA) was used as a molecular size marker.

Separations were performed on a Beckman Coulter P/ACE MDQ system equipped with a laser-induced fluorescence detection module and a 3 mW air-cooled argon ion laser (λ_ex_ = 488 nm and λ_em_ = 520 nm). The fused silica capillary was conditioned prior to electrophoresis separation of DNA using previously described rinsing [62] and coating [102] procedures. The capillary was filled with liquid nanogel solution a temperature below 24°C (19–21°C); then the temperature was increased to 30°C in order to form the sieving gel for accurate sizing separations of PCR amplicons. DNA samples were electrokinetically injected under reverse polarity as previously described [103]. Data collection and analysis were performed with 32 Karat Software version 5.0 (Beckman Coulter). Sizing was accomplished by co-injecting the *bucl5*, *13*, *14*, and *16* amplicons with two internal standards of known length that bracketed the size of the DNA targets. Internal standards of 100 bp and 250 bp were used to create a linear fit for DNA size (in bp) versus migration time. The resulting slope and intercept were then used to calculate the size (length in bp) of the *bucl* gene targets based on their migration times. The reported values for the calculated DNA size and standard deviation (in bp) are an average for *n* = 5 consecutive separations.

### Detection of *B*. *pseudomallei* gDNA in infected mice and human plasma using *bucl* markers

BALB/c mice (female 7–10 weeks of age at time of challenge-National Cancer Institute, NCI-Frederick, MD) were injected by the intraperitoneal (i.p.) route. Mice were infected with a dose equivalent to approximately 6 times the LD_50_ of *B*. *pseudomallei* HBPUB10134a (LD_50_ is 10 CFU) [67]. At various time points after infection mice were euthanized by exsanguination under deep anesthesia and spleens were harvested. Spleens were weighed and homogenized in RPMI 1640 medium (Life Technology, Grand Island, NY). Bacterial load in the freshly prepared spleen extracts was determined by plating serial dilutions on sheep blood agar (ThermoScientific Remel Products, KS). Plates were incubated at 37°C for two days before determining CFU counts. The spleen extracts were irradiated and confirmed sterile before use in PCR assays, and were stored at -70°C. Standard PCR for *bucl* genes *5*, *13*, *14*, and *16*, and probe-based qPCR for *bucl16* were performed as described above using 1 μL of DNA-containing spleen specimen. Additionally, qPCR reactions were performed with 30 ng *B*. *pseudomallei* K96243 gDNA spiked with 5% human plasma collected in EDTA tubes to test the feasibility of the assay on clinical samples containing plasma. qPCR experiments were performed in triplicate and Cq values were averaged.

### Statistical analyses

Statistical significance of *bucl* presence in pathogenic *B*. *pseudomallei* and *B*. *mallei* strains vs. nonpathogenic *B*. *thailandensis* was performed using the Fisher Exact Probability Test, followed by calculation of Cramer’s V squared.

## Disclaimers

Research was conducted under an IACUC approved protocol in compliance with the Animal Welfare Act, PHS Policy, and other federal statutes and regulations relating to animals and experiments involving animals. The facility where this research was conducted is accredited by the Association for Assessment and Accreditation of Laboratory Animal Care, International and adheres to principles stated in the 8^th^ Edition of the Guide for the Care and Use of Laboratory Animals, National Research Council, 2011.

Opinions, interpretations, conclusions, and recommendations are those of the authors and are not necessarily endorsed by the U. S. Army.

## Supporting Information

S1 Dataset Nucleotide sequences for all *bucl* genes. Nucleotide sequences were used to generate phylogenetic trees shown in Figs 5 and 6, S1, S2 and S3 Figs.
(DOCX)

S2 Dataset Percent identity between strains for each bucl gene. Pairwise nucleotide sequence alignments were generated using the ClustalW algorithm in the DNAStar Megalign software and used to calculate percent identities and divergence. Each table contains percent identities (right side of black squares) and divergence values (left side of black squares) for each pairwise alignment.
(PDF)

S1 Fig Phylogenetic analyses of *bucl1* in *B*. *pseudomallei* and B. *mallei* strains. Nucleotide sequences encoding (A) the noncollagenous domain and (B) entire gene of *bucl1* alleles were used. Support values for each branch are shown as posterior probability from Bayesian analysis and bootstrap values from maximum parsimony analysis, respectively (PP/MP). Scale bar is representative of evolutionary distance in substitutions per nucleotide.
(JPG)

S2 Fig Phylogenetic analysis of *bucl8* among *Burkholderia* strains. Bayesian analysis was performed on nucleotide sequences of *bucl8* non-collagenous regions of a set of *Burkholderia* strains described in Table 3. Support values for each branch are shown as posterior probability from Bayesian analysis. Several clusters of strains, C1, C4, and C5, corresponding to those observed in the concatenated analysis were also observed. Scale bar is representative of evolutionary distance in substitutions per nucleotide.
(JPG)

S3 Fig Phylogenetic analysis of Bucl3 and Bucl4 amino acid sequences among *Burkholderia* strains. Bayesian analysis was performed on amino acid sequences of (A) Bucl3 and (B) Bucl4 non-collagenous regions of a set of *Burkholderia* strains described in Table 3. Support values for each branch are shown as posterior probability from Bayesian analysis and bootstrap values from maximum parsimony analysis, respectively (PP/MP). Posterior probability value, which was not supported by maximum parsimony analysis is shown in red. Scale bar is representative of evolutionary distance in substitutions per nucleotide.
(JPG)

S4 Fig Distribution of *bucl* genes among *Burkholderia* spp. select agents by PCR. Presence of (A) *bucl* genes *2*, *3*, and *10* and (B) *bucl* genes *6*, *7*, *8*, and *15*, was assessed by PCR on a collection of genomic DNA from *B*. *pseudomallei* and *B*. *mallei* select agents (top panels), as well as in control strains of *B*. *thailandensis*, *B*. *cepacia*, *B*. *cenocepacia*, and *B*. *multivorans* (bottom panels). Amplicon sizes based on Bp K96243: In A) *bucl2*, 133 bp; *bucl3*, 166 bp; and *bucl10*, 109 bp; In B) *bucl6*, 115 bp; *bucl7*, 264 bp; *bucl8*, 243 bp; and *bucl15*, 95 bp.M, 50-bp DNA ladder. PCR data shown in panels A and B for 25 Bp strains come from two merged gel images.
(JPG)
