# Supplementary material for: A Unique Set of the Burkholderia Collagen-Like Proteins Provides Insight into Pathogenesis, Genome Evolution and Niche Adaptation, and Infection Detection
Source: PLoS One. 2015 Sep 10;10(9):e0137578. doi: 10.1371/journal.pone.0137578 (PMC4565658; doi:10.1371/journal.pone.0137578)
Supplement: S1 Dataset — Nucleotide sequences were used to generate phylogenetic trees shown in Figs 5 and 6, S1, S2 and S3 Figs. (DOCX) [file pone.0137578.s001.docx]

>Bucl1_Bps_K96243.seq Reverse Complement DNA Sequence Untitled Seq #5(1,513)

GTGCGCGGCAGCTTGCACGCCGCGCCTGGGCGCCGCCGCGGCCGCGGGCGCGCGCATCGAATGCGGCGGCGCGCAGGTT

GCGGCGTTGCGGCGCTTGACGTTGGAGGGGGCTGCCGGGATTCTGACGGCGCGAACGGCGCGAACGGCGCGAACGGCGC

GAACGGCGCGAACGGCGCGAACGGCGCGAACGGCGCGAACGGCACGAACGGCACGAACGGCACGAACGGCGCGGCTGGA

GCCGACACCGGCCGCGGCGGTTCGGTGGCGCTGGCGCATCGTGAACGGCGCGCGGCGGCGGGTGGCGGTGCGATCTTCG

CGACAGCGGCGACAGGGCCGAGCAACGGACGACACGCAACCGACGAAGGCAACCGACGGCGGCAACCGACGAAGGCGCG

CGACGCATGCCGCGCACCATCGCCGCGCGAGCGGCGCGGCGACGATGGTTTCGAATCACCGGCGCGCCGGATGCGGCGA

GGCCGGCGCCGCAACGGCGCGTCGCGGCCGTTGCGGTGA

>A4LPM8_BURPS_Bucl1_Bps_305.seq Reverse Complement DNA Sequence Untitled Seq #2(1,558)

gtgcgcggcggcttgcacgccgcgcctgggcgccgctgcggccgcgggcgcgcgcatcgactgcggcggcgcgcaggtt

gcggcgttgcggcgcttgacgttggagggggctgccgggattctgacggcgcgaacggcgcgaacggcgcgaacggcgc

gaacggcgcgaacggcgcgaacggcgcgaacggcgcgaacggcgcgaacggcacggacggcacgaacggcacgaacggc

acgaacggcacgaacggcacgaacggcacgaacggcgcggctggagccgacaccggccgcggcggttcggtggcgctgg

cgcatcgtgaacggcgcgcggcggcgggtggcggtgcgatcttcgcgacagcggcgacagggccgagcaacggacgaca

cgcaaccgacgaaggcaaccgacggcggcaaccgacgaaggcgcgcgacgcatgccgcgcaccatcgccgcgcgagcgg

cgcggcgacgatggtttcgaatcaccggcgcgccggatgcggcgaggccggcgccgcaacggcgcgtcgcggccgttgc

ggtga

>A3P738_BURP0_Bucl1_Bps_1106a.seq Reverse Complement DNA Sequence Untitled Seq #9(1,558)

GTGCGCGGCGGCTTGCACGCCGCGCCTGGGCGCCGCCGCGGCCGCGGGCGCGCGCATCGAATGCGGCGGCGCGCAGGTT

GCGGCGTTGCGGCGCTTGACGTTGGAGGGGGCTGCCGGGATTCTGACGGCGCGAACGGCGCGAACGGCGCGAACGGCGC

GAACGGCGCGAACGGCGCGAACGGCGCGAACGGCGCGAACGGCGCGAACGGCGCGAACGGCGCGAACGGCGCGAACGGC

GCGAACGGCGCGAACGGCGCGAACGGCGCGAACGGCACGGCTGGAGCCGACACCGGCCGCGGCGGTTCGGTGGCGCTGG

CGCATCGTGAACGGCGCGCGGCGGCGGGTGGCGGTGCGATCTTCGCGACAGCGGCGACAGGGCCGAGCAACGGACGACA

CGCAACCGACGAAGGCAACCGACGGCGGCAACCGACGAAGGCGCGCGACGCATGCCGCGCACCATCGCCGCGCGAGCGG

CGCGGCGACGATGGTTTCGAATCACCGGCGCGCCGGATGCGGCGAGGCCGGCGCCGCAACGGCGCGTCGCGGCCGTTGC

GGTGA

>C4I6P3_BURPS_Bucl1_Bps_MSHR346.seq Reverse Complement DNA Sequence Untitled Seq #1(1,531)

GTGCGCGGCGGCTTGCACGCCGCGCCTGGGCGCCGCCGCGGCCGCGGGCGCGCGCATCGAATGCGGCGGCGCGCAGGTT

GCGGCGTTGCGGCGCTTGACGTTGGAGGGGGCTGCCGGGATTCTGACGGCGCGAACGGCGCGAACGGCGCGAACGGCGC

GAACGGCGCGAACGGCGCGAACGGCGCGAACGGCACGGACGGCACGGACGGCACGAACGGCACGAACGGCACGGACGGC

ACGAACGGCACGAACGGCACGAACGGCGCGGCTGGAGCCGACACCGGCCGCGGCGGTTCGGTGGCGCTGGCGCATCGTG

AACGGCGCGCGGCGGCGGGTGGCGGTGCGATCTTCGCGACAGCGGCGACAGGGCCGAGCAACGGACGACACGCAACCGA

CGAAGGCGCGCGACGCATGCCGCGCACCATCGCCGCGCGAGCGGCGCGGCGACGATGGTTTCGAATCACCGGCGCGCCG

GATGCGGCGAGGCCGGCGCCGCAACGGCGCGTCGCGGCCGTTGCGGTGA

>Bucl1_Bps_1026b.seq Reverse Complement DNA Sequence Untitled Seq #1(1,540)

GTGCGCGGCAGCTTGCACGCCGCGCCTGGGCGCCGCCGCGGCCGCGGGCGCGCGCATCGAATGCGGCGGCGCGCAGGTT

GCGGCGTTGCGGCGCTTGACGTTGGAGGGGGCTGCCGGGATTCTGACGGCGCGAACGGCGCGAACGGCGCGAACGGCGC

GAACGGCGCGAACGGCGCGAACGGCGCGAACGGCGCGAACGGCGCGAACGGCGCGAACGGCGCGAACGGCACGAACGGC

ACGAACGGCACGAACGGCGCGGCTGGAGCCGACACCGGCCGCGGCGGTTCGGTGGCGCTGGCGCATCGTGAACGGCGCG

CGGCGGCGGGTGGCGGTGCGATCTTCGCGACAGCGGCGACAGGGCCGAGCAACGGACGACACGCAACCGACGAAGGCAA

CCGACGGCGGCAACCGACGAAGGCGCGCGACGCATGCCGCGCACCATCGCCGCGCGAGCGGCGCGGCGACGATGGTTTC

GAATCACCGGCGCGCCGGATGCGGCGAGGCCGGCGCCGCAACGGCGCGTCGCGGCCGTTGCGGTGA

>Bucl1_Bps_1710b.seq Reverse Complement DNA Sequence Untitled Seq #17(1,504)

gtgcgcggcggcttgcacgccgcgcctgggcgccgccgcggccgcgggcgcgcgcatcgaatgcggcggcgcgcaggtt

gcggcgttgcggcgcttgacgttggagggggctgccgggattctgacggcgcgaacggcgcgaacggcgcgaacggcgc

gaacggcgcgaacggcgcgaacggcgcgaacggcgcgaacggcgcgaacggcacgaacggcgcggctggagccgacacc

ggccgcggcggttcggtggcgctggcgcatcgtgaacggcgcgcggcggcgggtggcggtgcgatcttcgcgacagcgg

cgacagggccgagcaacggacgacacgcaaccgacgaaggcaaccgacggcggcaaccgacgaaggcgcgcgacgcatg

ccgcgcaccatcgccgcgcgagcggcgcggcgacgatggtttcgaatcaccggcgcgccggatgcggcgaggccggcgc

cgcaacggcgcgtcgcggccgttgcggtga

>Bucl1_Bps_BPC006.seq Reverse Complement DNA Sequence Untitled Seq #32(1,556)

gtgcgcggcggcttgcacgccgcgcctgggcgccgccgcggccgcgggcgcgcgcatcgaatgcggcggcgcgcaggtt

gcggcgttgcggcgcttgacgttggagggggctgccgggattctgacggcgcgaacggcgcgaacggcgcgaacggcgc

gaacggccgacggcgcgaacggcgcgaacggcgcgaacggcgcgaacggcgcgaacggcgcgaacggcgcgaacggcgc

gaacggcgcgaacggcgcgaacggcgcgaacggcacggctggagccgacaccggccgcggcggttcggtggcgctggcg

catcgtgaacggcgcgcggcggcgggtggcggtgcgatcttcgcgacagcggcgacagggccgagcaacggacgacacg

caaccgacgaaggcaaccgacggcggcaaccgacgaaggcgcgcgacgcatgccgcgcaccatcgccgcgcgagcggcg

cggcgacgatggtttcgaatcaccggcgcgccggatgcggcgaggccggcgccgcaacggcgcgtcgcggccgttgcgg

tga

>Bucl1_Bps_668.seq Reverse Complement DNA Sequence Untitled Seq #12(1,459)

GTGCGCGGCGGCTTGCACGCCGCGCCTGGGCGCCGCTGCGGCCGCGGGCGCGCGCATCGGATGCGGCGGCGCGCAGGTT

GCGGCGTTGCGGCGCTTGACGTTGGAGGGGGCTGCCGGGATTCTGACGGCGCGAACGGCGCGAACGGCGCGAACGGCGC

GAACGGCGCGAACGGCGCGAACGGCGCGAACGGCGCGGCTGGAGCCGACACCGGCCGCGGCGGTTCGGTGGCGCTGGCG

CATCGTGAACGGCGCGCGGCGGCGGGTGGCGGTGCGATCTTCGCGACAGCGGCGACAGGGCCGAGCAACGGACGACACG

CAACCGACGAAGGCGCGCGACGCATGCCGCGCACCATCGCCGCGCGAGCGGCGCGGCGACGATGGTTTCGAATCACCGG

CGCGCCGGATGCGGCGAGGCCGGCGCCGCAACGGCGCGTCGCTGCCGCTGCGGTGA

>Bucl1_Bps_NCTC_13179.seq Created: Thursday, January 02, 2014 2:29 PM

gtgcgcggcggcttgcacgccgcgcctgggcgccgccgcggccgcgggcgcgcgcatcgaatgcggcggcgcgcaggtt

gcggcgttgcggcgcttgacgttggagggggctgccgggattctgacggcgcgaacggcgcgaacggcgcgaacggcgc

gaacggcgcgaacggcgcgaacggcacggctggagccgacaccggccgcggcggttcggtggcgctggcgcatcgtgaa

cggcgcgcggcggcgggtggcggtgcgatcttcgcgacagcggcgacagggccgagcaacggacgacacgcaaccgacg

aaggcgcgcgacgcatgccgcgcaccatcgccgcgcgagcggcgcggcgacgatggtttcgaatcaccggcgcgccgga

tgcggcgaggccggcgccgcaacggcgcgtcgcggccgttgcgggga

>Bucl1_Bps_MSHR146.seq Reverse Complement DNA Sequence Untitled Seq #11(1,459)

GTGCGCGGCGGCTTGCACGCCGCGCCTGGGCGCCGCCGCGGCCGCAGGCGCGCGCATCGAATGCGGCGGCGCGCAGGTT

GCGGCGTTGCGGCGCTTGACGTTGGAGGGGGCTGCCGGGATTCTGACGGCGCGAACGGCGCGAACGGCGCGAACGGCGC

GAACGGCGCGAACGGCGCGGCTGGAGCCGACACCGGCCGCGGCGGTTCGGTGGCGCTGGCGCATCGTGAACGGCGCGCG

GCGGCGGGTGGCGGTGCGATCTTCGCGACAGCGGCGACAGGGCCGAGCAACGGACGACACGCAACCGACGAAGGCAACC

GACGGCGGCAACCGACGAAGGCGCGCGACGCATGCCGCGCACCATCGCCGCGCGAGCGGCGCGGCGACGATGGTTTCGA

ATCACCGGCGCGCCGGATGCGGCGAGGCCGGCGCCGCAACGGCGCGTCGCGGCCGTTGCGGTGA

>Bucl1_Bps_MSHR511.seq Created: Wednesday, March 19, 2014 11:12 AM

GTGCGCGGCGGCTTGCACGCCGCGCCTGGGCGCCGCCGCGGCCGCAGGCGCGCGCATCGAATGCGGCGGCGCGCAGGTT

GCGGCGTTGCGGCGCTTGACGTTGGAGGGGGCTGCCGGGATTCTGACGGCGCGAACGGCGCGAACGGCGCGAACGGCGC

GAACGGCGCGAACGGCGCGAACGGCGCGAACGGCGCGAACGGCGCGGCTGGAGCCGACACCGGCCGCGGCGGTTCGGTG

GCGCTGGCGCATCGTGAACGGCGCGCGGCGGCGGGTGGCGGTGCGATCTTCGCGACAGCGGCGACAGGGCCGAGCAACG

GACGACACGCAACCGACGAAGGCAACCGACGGCGGCAACCGACGAAGGCGCGCGACGCATGCCGCGCACCATCGCCGCG

CGAGCGGCGCGGCGACGATGGTTTCGAATCACCGGCGCGCCGGATGCGGCGAGGCCGGCGCCGCAACGGCGCGTCGCGG

CCGTTGCGGTGA

>Bucl1_Bps_MSHR520.seq Reverse Complement DNA Sequence Untitled Seq #4(1,540)

GTGCGCGGCGGCTTGCACGCCGCGCCTGGGCGCCGCTGCGGCCGCGGGCGCGCGCATCGACTGCGGCGGCGCGCAGGTT

GCGGCGTTGCGGCGCTTGACGTTGGAGGGGGCTGCCGGGATTCTGACGGCGCGAACGGCGCGAACGGCGCGAACGGCGC

GAACGGCGCGAACGGCGCGAACGGCGCGAACGGCACGGACGGCACGAACGGCACGAACGGCACGAACGGCACGAACGGC

ACGAACGGCACGAACGGCGCGGCTGGAGCCGACACCGGCCGCGGCGGTTCGGTGGCGCTGGCGCATCGTGAACGGCGCG

CGGCGGCGGGTGGCGGTGCGATCTTCGCGACAGCGGCGACAGGGCCGAGCAACGGACGACACGCAACCGACGAAGGCAA

CCGACGGCGGCAACCGACGAAGGCGCGCGACGCATGCCGCGCACCATCGCCGCGCGAGCGGCGCGGCGACGATGGTTTC

GAATCACCGGCGCGCCGGATGCGGCGAGGCCGGCGCCGCAACGGCGCGTCGCGGCCGTTGCGGTGA

>Bucl1_Bps_NAU20B-16.seq Created: Wednesday, March 19, 2014 11:18 AM

GTGCGCGGCGGCTTGCACGCCGCGCCTGGGCGCCGCCGCGGCCGCAGGCGCGCGCATCGAATGCGGCGGCGCGCAGGTT

GCGGCGTTGCGGCGCTTGACGTTGAAGGGGGCTGCCGGGATTCTGACGGCGCGAACGGCGCGAACGGCGCGAACGGCGC

GAACGGCGCGAACGGCGCGAACGGCGCGGCTGGAGCCGACACCGGCCGCGGCGGTTCGGTGGCGCTGGCGCATCGTGAA

CGGCGCGCGGCGGCGGGTGGCGGTGCGATCTTCGCGACAGCGGCGACAGGGCCGAGCAACGGACGACACGCAACCGACG

AAGGCAACCGACGGCGGCAACCGACGAAGGCGCGCGACGCATGCCGCGCACCATCGCCGCGCGAGCGGCGCGGCGACGA

TGGTTTCGAATCACCGGCGCGCCGGATGCGGCGAGGCCGGCGCCGCAACGGCGCGTCGCGGCCGTTGCGGTGA

>Bucl1_Bps_NCTC_13178.seq Reverse Complement DNA Sequence Untitled Seq #1(1,504)

GTGCGCGGCGGCTTGCACGCCGCGCCTGGGCGCCGCCGCAGCCGCGGGCGCGCGCATCGAATGCGGCGGCGCGCAGGTT

GCGGCGTTGCGGCGCTTGACGTTGGAGGGGGCTGCCGGGATTCTGACGGCGCGAACGGCGCGAACGGCGCGAACGGCGC

GAACGGCGCGAACGGCGCGAACGGCGCGAACGGCGCGAACGGCGCGAACGGCGCGAACGGCACGGCTGGAGCCGACACC

GGCCGCGGCGGTTCGGTGGCGCTGGCGCATCGTGAACGGCGCGCGGCGGCGGGTGGCGGTGCGATCTTCGCGACAGCGG

CGACAGGGCCGAGCAACGGACGACACGCAACCGACGAAGGCAACCGACGGCGGCAACCGACGAAGGCGCGCGACGCATG

CCGCGCACCATCGCCGCGCGAGCGGCGCGGCGACGATGGTTTCGAATCACCGGCGCGCCGGATGCGGCGAGGCCGGCGC

CGCAACGGCGCGTCGCGGCCGTTGCGGTGA

>Bucl1_Bm_2000031281.seq Created: Sunday, March 16, 2014 6:28 PM

GTGCGCGGCGGCTTGCACGCCGCGCCTGGGCGCCGCCGCGGCCGCGGGCGCGCGCATCGAATGCGGCGGCGCGCAGGTT

GCGGCGTTGCGGCGCTTGACGTTGGAGGGGGCTGCCGGGATTCTGACGGCGCGAACGGCGCGAACGGCGCGAACGGCGC

GAACGGCGCGAACGGCGCGAACGGCGCGAACGGCGCGAACGGCGCGAACGGCGCGAACGGCGCGAACGGCGCGAACGGC

GCGAACGGCGCGAACGGCACGAACGGCACGAACGGCACGAACGGCGCGGCTGGAGCCGACACCGGCCGCGGCGGTTCGG

TGGCGCTGGCGCATCGTGAACGGCGCGCGGCGGCGGGTGGCGGTGCGATCTTCGCGACAGCGGCGACAGGGCCGAGCAA

CGGACGACACGCAACCGACGAAGGCAACCGACGGCGGCAACCGACGAAGGCGCGCGACGCATGCCGCGCACCATCGCCG

CGCGAGCGGCGCGGCGACGATGGTTTCGAATCACCGGCGCGCCGGATGCGGCGAGGCCGGCGCCGCAACGGCGCGTCGC

GGCCGTTGCGGTGA

>Bucl1_Bm_2002721280.seq Reverse Complement DNA Sequence Untitled Seq #9(1,594)

GTGCGCGGCGGCTTGCACGCCGCGCCTGGGCGCCGCCGCGGCCGCGGGCGCGCGCATCGAATGCGGCGGCGCGCAGGTT

GCGGCGTTGCGGCGCTTGACGTTGGAGGGGGCTGCCGGGATTCTGACGGCGCGAACGGCGCGAACGGCGCGAACGGCGC

GAACGGCGCGAACGGCGCGAACGGCGCGAACGGCGCGAACGGCGCGAACGGCGCGAACGGCGCGAACGGCGCGAACGGC

GCGAACGGCGCGAACGGCGCGAACGGCGCGAACGGCACGAACGGCACGAACGGCACGAACGGCACGAACGGCGCGGCTG

GAGCCGACACCGGCCGCGGCGGTTCGGTGGCGCTGGCGCATCGTGAACGGCGCGCGGCGGCGGGTGGCGGTGCGATCTT

CGCGACAGCGGCGACAGGGCCGAGCAACGGACGACACGCAACCGACGAAGGCAACCGACGGCGGCAACCGACGAAGGCG

CGCGACGCATGCCGCGCACCATCGCCGCGCGAGCGGCGCGGCGACGATGGTTTCGAATCACCGGCGCGCCGGATGCGGC

GAGGCCGGCGCCGCAACGGCGCGTCGCGGCCGTTGCGGTGA

>Bucl1_Bm_A188.seq Created: Sunday, March 16, 2014 7:35 PM

GTGCGCGGCGGCTTGCACGCCGCGCCTGGGCGCCGCCGCGGCCGCGGGCGCGCGCATCGAATGCGGCGGCGCGCAGGTT

GCGGCGTTGCGGCGCTTGACGTTGGAGGGGGCTGCCGGGATTCTGACGGCGCGAACGGCGCGAACGGCGCGAACGGCGC

GAACGGCGCGAACGGCGCGAACGGCGCGAACGGCGCGAACGGCGCGAACGGCGCGAACGGCGCGAACGGCGCGAACGGC

GCGAACGGCACGAACGGCACGAACGGCACGAACGGCACGAACGGCGCGGCTGGAGCCGACACCGGCCGCGGCGGTTCGG

TGGCGCTGGCGCATCGTGAACGGCGCGCGGCGGCGGGTGGCGGTGCGATCTTCGCGACAGCGGCGACAGGGCCGAGCAA

CGGACGACACGCAACCGACGAAGGCAACCGACGGCGGCAACCGACGAAGGCGCGCGACGCATGCCGCGCACCATCGCCG

CGCGAGCGGCGCGGCGACGATGGTTTCGAATCACCGGCGCGCCGGATGCGGCGAGGCCGGCGCCGCAACGGCGCGTCGC

GGCCGTTGCGGTGA

>Bucl1_Bm_A193.seq Created: Sunday, March 16, 2014 7:28 PM

GTGCGCGGCGGCTTGCACGCCGCGCCTGGGCGCCGCCGCGGCCGCGGGCGCGCGCATCGAATGCGGCGGCGCGCAGGTT

GCGGCGTTGCGGCGCTTGACGTTGGAGGGGGCTGCCGGGATTCTGACGGCGCGAACGGCGCGAACGGCGCGAACGGCGC

GAACGGCGCGAACGGCGCGAACGGCGCGAACGGCGCGAACGGCGCGAACGGCGCGAACGGCGCGAACGGCGCGAACGGC

ACGAACGGCACGAACGGCACGAACGGCACGAACGGCGCGGCTGGAGCCGACACCGGCCGCGGCGGTTCGGTGGCGCTGG

CGCATCGTGAACGGCGCGCGGCGGCGGGTGGCGGTGCGATCTTCGCGACAGCGGCGACAGGGCCGAGCAACGGACGACA

CGCAACCGACGAAGGCAACCGACGGCGGCAACCGACGAAGGCGCGCGACGCATGCCGCGCACCATCGCCGCGCGAGCGG

CGCGGCGACGATGGTTTCGAATCACCGGCGCGCCGGATGCGGCGAGGCCGGCGCCGCAACGGCGCGTCGCGGCCGTTGC

GGTGA

>Bucl1_Bm_China_7.seq Created: Sunday, March 16, 2014 6:38 PM

GTGCGCGGCGGCTTGCACGCCGCGCCTGGGCGCCGCCGCGGCCGCGGGCGCGCGCATCGAATGCGGCGGCGCGCAGGTT

GCGGCGTTGCGGCGCTTGACGTTGGAGGGGGCTGCCGGGATTCTGACGGCGCGAACGGCGCGAACGGCGCGAACGGCGC

GAACGGCGCGAACGGCGCGAACGGCGCGAACGGCGCGAACGGCGCGAACGGCGCGAACGGCGCGAACGGCGCGAACGGC

GCGAACGGCGCGAACGGCACGAACGGCACGAACGGCACGAACGGCGCGGCTGGAGCCGACACCGGCCGCGGCGGTTCGG

TGGCGCTGGCGCATCGTGAACGGCGCGCGGCGGCGGGTGGCGGTGCGATCTTCGCGACAGCGGCGACAGGGCCGAGCAA

CGGACGACACGCAACCGACGAAGGCAACCGACGGCGGCAACCGACGAAGGCGCGCGACGCATGCCGCGCACCATCGCCG

CGCGAGCGGCGCGGCGACGATGGTTTCGAATCACCGGCGCGCCGGATGCGGCGAGGCCGGCGCCGCAACGGCGCGTCGC

GGCCGTTGCGGTGA

>Bucl1_Bm_PRL-20.seq Reverse Complement DNA Sequence Untitled Seq #7(1,549)

GTGCGCGGCGGCTTGCACGCCGCGCCTGGGCGCCGCCGCGGCCGCGGGCGCGCGCATCGAATGCGGCGGCGCGCAGGTT

GCGGCGTTGCGGCGCTTGACGTTGGAGGGGGCTGCCGGGATTCTGACGGCGCGAACGGCGCGAACGGCGCGAACGGCGC

GAACGGCGCGAACGGCGCGAACGGCGCGAACGGCGCGAACGGCGCGAACGGCGCGAACGGCGCGAACGGCACGAACGGC

ACGAACGGCACGAACGGCACGAACGGCGCGGCTGGAGCCGACACCGGCCGCGGCGGTTCGGTGGCGCTGGCGCATCGTG

AACGGCGCGCGGCGGCGGGTGGCGGTGCGATCTTCGCGACAGCGGCGACAGGGCCGAGCAACGGACGACACGCAACCGA

CGAAGGCAACCGACGGCGGCAACCGACGAAGGCGCGCGACGCATGCCGCGCACCATCGCCGCGCGAGCGGCGCGGCGAC

GATGGTTTCGAATCACCGGCGCGCCGGATGCGGCGAGGCCGGCGCCGCAACGGCGCGTCGCGGCCGTTGCGGTGA

>Bucl1_Bm_strain_6.seq Created: Sunday, March 16, 2014 7:40 PM

GTGCGCGGCGGCTTGCACGCCGCGCCTGGGCGCCGCCGCGGCCGCGGGCGCGCGCATCGAATGCGGCGGCGCGCAGGTT

GCGGCGTTGCGGCGCTTGACGTTGGAGGGGGCTGCCGGGATTCTGACGGCGCGAACGGCGCGAACGGCGCGAACGGCGC

GAACGGCGCGAACGGCGCGAACGGCGCGAACGGCGCGAACGGCGCGAACGGCGCGAACGGCGCGAACGGCGCGAACGGC

GCGAACGGCGCGAACGGCGCGAACGGCACGAACGGCACGAACGGCGCGGCTGGAGCCGACACCGGCCGCGGCGGTTCGG

TGGCGCTGGCGCATCGTGAACGGCGCGCGGCGGCGGGTGGCGGTGCGATCTTCGCGACAGCGGCGACAGGGCCGAGCAA

CGGACGACACGCAACCGACGAAGGCAACCGACGGCGGCAACCGACGAAGGCGCGCGACGCATGCCGCGCACCATCGCCG

CGCGAGCGGCGCGGCGACGATGGTTTCGAATCACCGGCGCGCCGGATGCGGCGAGGCCGGCGCCGCAACGGCGCGTCGC

GGCCGTTGCGGTGA

>Bucl1_Bm_strain_11.seq Created: Sunday, March 16, 2014 8:05 PM

GTGCGCGGCGGCTTGCACGCCGCGCCTGGGCGCCGCCGCGGCCGCGGGCGCGCGCATCGAATGCGGCGGCGCGCAGGTT

GCGGCGTTGCGGCGCTTGACGTTGGAGGGGGCTGCCGGGATTCTGACGGCGCGAACGGCGCGAACGGCGCGAACGGCGC

GAACGGCGCGAACGGCGCGAACGGCGCGAACGGCGCGAACGGCGCGAACGGCGCGAACGGCGCGAACGGCGCGAACGGC

GCGAACGGCGCGAACGGCACGAACGGCACGAACGGCACGAACGGCACGAACGGCACGAACGGCGCGGCTGGAGCCGACA

CCGGCCGCGGCGGTTCGGTGGCGCTGGCGCATCGTGAACGGCGCGCGGCGGCGGGTGGCGGTGCGATCTTCGCGACAGC

GGCGACAGGGCCGAGCAACGGACGACACGCAACCGACGAAGGCAACCGACGGCGGCAACCGACGAAGGCGCGCGACGCA

TGCCGCGCACCATCGCCGCGCGAGCGGCGCGGCGACGATGGTTTCGAATCACCGGCGCGCCGGATGCGGCGAGGCCGGC

GCCGCAACGGCGCGTCGCGGCCGTTGCGGTGA

>A2S1L2_BURM9_Bucl1_Bm_NCTC_10229.seq Created: Monday, February 04, 2013 11:24 AM

GTGCGCGGCGGCTTGCACGCCGCGCCTGGGCGCCGCCGCGGCCGCGGGCGCGCGCATCGAATGCGGCGGCGCGCAGGTT

GCGGCGTTGCGGCGCTTGACGTTGGAGGGGGCTGCCGGGATTCTGACGGCGCGAACGGCGCGAACGGCGCGAACGGCGC

GAACGGCGCGAACGGCGCGAACGGCGCGAACGGCACGAACGGCACGAACGGCACGAACGGCACGAACGGCACGAACGGC

GCGGCTGGAGCCGACACCGGCCGCGGCGGTTCGGTGGCGCTGGCGCATCGTGAACGGCGCGCGGCGGCGGGTGGCGGTG

CGATCTTCGCGACAGCGGCGACAGGGCCGAGCAACGGACGACACGCAACCGACGAAGGCAACCGACGGCGGCAACCGAC

GAAGGCGCGCGACGCATGCCGCGCACCATCGCCGCGCGAGCGGCGCGGCGACGATGGTTTCGAATCACCGGCGCGCCGG

ATGCGGCGAGGCCGGCGCCGCAACGGCGCGTCGCGGCCGTTGCGGTGA

>A3MCC9_BURM7_Bucl1_Bm_NCTC_10247.seq Created: Monday, February 04, 2013 12:16 PM

GTGCGCGGCGGCTTGCACGCCGCGCCTGGGCGCCGCCGCGGCCGCGGGCGCGCGCATCGAATGCGGCGGCGCGCAGGTT

GCGGCGTTGCGGCGCTTGACGTTGGAGGGGGCTGCCGGGATTCTGACGGCGCGAACGGCGCGAACGGCGCGAACGGCGC

GAACGGCGCGAACGGCGCGAACGGCGCGAACGGCGCGAACGGCGCGAACGGCGCGAACGGCGCGAACGGCGCGAACGGC

GCGAACGGCGCGAACGGCACGAACGGCACGAACGGCACGAACGGCACGAACGGCACGAACGGCGCGGCTGGAGCCGACA

CCGGCCGCGGCGGTTCGGTGGCGCTGGCGCATCGTGAACGGCGCGCGGCGGCGGGTGGCGGTGCGATCTTCGCGACAGC

GGCGACAGGGCCGAGCAACGGACGACACGCAACCGACGAAGGCAACCGACGGCGGCAACCGACGAAGGCGCGCGACGCA

TGCCGCGCACCATCGCCGCGCGAGCGGCGCGGCGACGATGGTTTCGAATCACCGGCGCGCCGGATGCGGCGAGGCCGGC

GCCGCAACGGCGCGTCGCGGCCGTTGCGGTGA

>A5J478_BURMA_Bucl1_Bm_FMH.seq Reverse Complement DNA Sequence Untitled Seq #15(1,567)

GTGCGCGGCGGCTTGCACGCCGCGCCTGGGCGCCGCCGCGGCCGCGGGCGCGCGCATCGAATGCGGCGGCGCGCAGGTT

GCGGCGTTGCGGCGCTTGACGTTGGAGGGGGCTGCCGGGATTCTGACGGCGCGAACGGCGCGAACGGCGCGAACGGCGC

GAACGGCGCGAACGGCGCGAACGGCGCGAACGGCGCGAACGGCGCGAACGGCGCGAACGGCGCGAACGGCGCGAACGGC

GCGAACGGCGCGAACGGCACGAACGGCACGAACGGCACGAACGGCGCGGCTGGAGCCGACACCGGCCGCGGCGGTTCGG

TGGCGCTGGCGCATCGTGAACGGCGCGCGGCGGCGGGTGGCGGTGCGATCTTCGCGACAGCGGCGACAGGGCCGAGCAA

CGGACGACACGCAACCGACGAAGGCAACCGACGGCGGCAACCGACGAAGGCGCGCGACGCATGCCGCGCACCATCGCCG

CGCGAGCGGCGCGGCGACGATGGTTTCGAATCACCGGCGCGCCGGATGCGGCGAGGCCGGCGCCGCAACGGCGCGTCGC

GGCCGTTGCGGTGA

>A5XL93_BURMA_Bucl1_Bm_JHU.seq Reverse Complement DNA Sequence Untitled Seq #22(1,567)

GTGCGCGGCGGCTTGCACGCCGCGCCTGGGCGCCGCCGCGGCCGCGGGCGCGCGCATCGAATGCGGCGGCGCGCAGGTT

GCGGCGTTGCGGCGCTTGACGTTGGAGGGGGCTGCCGGGATTCTGACGGCGCGAACGGCGCGAACGGCGCGAACGGCGC

GAACGGCGCGAACGGCGCGAACGGCGCGAACGGCGCGAACGGCGCGAACGGCGCGAACGGCGCGAACGGCGCGAACGGC

GCGAACGGCGCGAACGGCACGAACGGCACGAACGGCACGAACGGCGCGGCTGGAGCCGACACCGGCCGCGGCGGTTCGG

TGGCGCTGGCGCATCGTGAACGGCGCGCGGCGGCGGGTGGCGGTGCGATCTTCGCGACAGCGGCGACAGGGCCGAGCAA

CGGACGACACGCAACCGACGAAGGCAACCGACGGCGGCAACCGACGAAGGCGCGCGACGCATGCCGCGCACCATCGCCG

CGCGAGCGGCGCGGCGACGATGGTTTCGAATCACCGGCGCGCCGGATGCGGCGAGGCCGGCGCCGCAACGGCGCGTCGC

GGCCGTTGCGGTGA

>A9K4Y8_BURMA_Bucl1_Bm_ATCC_10399.seq Created: Monday, February 04, 2013 11:54 AM

GTGCGCGGCGGCTTGCACGCCGCGCCTGGGCGCCGCCGCGGCCGCGGGCGCGCGCATCGAATGCGGCGGCGCGCAGGTT

GCGGCGTTGCGGCGCTTGACGTTGGAGGGGGCTGCCGGGATTCTGACGGCGCGAACGGCGCGAACGGCGCGAACGGCGC

GAACGGCGCGAACGGCGCGAACGGCGCGAACGGCGCGAACGGCGCGAACGGCGCGAACGGCGCGAACGGCACGAACGGC

ACGAACGGCACGAACGGCGCGGCTGGAGCCGACACCGGCCGCGGCGGTTCGGTGGCGCTGGCGCATCGTGAACGGCGCG

CGGCGGCGGGTGGCGGTGCGATCTTCGCGACAGCGGCGACAGGGCCGAGCAACGGACGACACGCAACCGACGAAGGCAA

CCGACGGCGGCAACCGACGAAGGCGCGCGACGCATGCCGCGCACCATCGCCGCGCGAGCGGCGCGGCGACGATGGTTTC

GAATCACCGGCGCGCCGGATGCGGCGAGGCCGGCGCCGCAACGGCGCGTCGCGGCCGTTGCGGTGA

>C4AZF6_BURMA_Bucl1_Bm_GB8_horse_4.seq Created: Thursday, May 22, 2014 9:40 AM

GTGCGCGGCGGCTTGCACGCCGCGCCTGGGCGCCGCCGCGGCCGCGGGCGCGCGCATCGAATGCGGCGGCGCGCAGGTT

GCGGCGTTGCGGCGCTTGACGTTGGAGGGGGCTGCCGGGATTCTGACGGCGCGAACGGCGCGAACGGCGCGAACGGCGC

GAACGGCGCGAACGGCGCGAACGGCGCGAACGGCGCGAACGGCGCGAACGGCGCGAACGGCGCGAACGGCGCGAACGGC

GCGAACGGCGCGAACGGCACGAACGGCACGAACGGCACGAACGGCGCGGCTGGAGCCGACACCGGCCGCGGCGGTTCGG

TGGCGCTGGCGCATCGTGAACGGCGCGCGGCGGCGGGTGGCGGTGCGATCTTCGCGACAGCGGCGACAGGGCCGAGCAA

CGGACGACACGCAACCGACGAAGGCAACCGACGGCGGCAACCGACGAAGGCGCGCGACGCATGCCGCGCACCATCGCCG

CGCGAGCGGCGCGGCGACGATGGTTTCGAATCACCGGCGCGCCGGATGCGGCGAGGCCGGCGCCGCAACGGCGCGTCGC

GGCCGTTGCGGTGA

>Q62AX7_BURMA_Bucl1_Bm_ATCC_23344.seq Created: Friday, January 03, 2014 12:15 PM

gtgcgcggcggcttgcacgccgcgcctgggcgccgccgcggccgcgggcgcgcgcatcgaatgcggcggcgcgcaggtt

gcggcgttgcggcgcttgacgttggagggggctgccgggattctgacggcgcgaacggcgcgaacggcgcgaacggcgc

gaacggcgcgaacggcgcgaacggcgcgaacggcgcgaacggcgcgaacggcgcgaacggcgcgaacggcgcgaacggc

gcgaacggcgcgaacggcacgaacggcacgaacggcacgaacggcgcggctggagccgacaccggccgcggcggttcgg

tggcgctggcgcatcgtgaacggcgcgcggcggcgggtggcggtgcgatcttcgcgacagcggcgacagggccgagcaa

cggacgacacgcaaccgacgaaggcaaccgacggcggcaaccgacgaaggcgcgcgacgcatgccgcgcaccatcgccg

cgcgagcggcgcggcgacgatggtttcgaatcaccggcgcgccggatgcggcgaggccggcgccgcaacggcgcgtcgc

ggccgttgcggtga

>Bucl2_Bps_K96243.seq Created: Monday, January 20, 2014 4:54 PM

ttgagcgcagcatcggatttcatcgtcgtctcctcgccgaccggtttcgataaacggccgcccgcggtcaagcgcgcgc

ggcggcgcatgcaaaccagcatcgcgcattacgcggcgcggaccttgatgcgcgtcaacgaagggaaaatgacaaacga

atgcgcggcctggcgttcgcgcgacgtcgacagccgaggcgacgcggagaaaggcaggccgcgatcggcccggcggccg

tccggccgccgccgaaccggcgggaagcgacgtcaacgaaaacggccgcacgaagcggcgtgcgacggaaagctcgcgg

gccgtgccgcgaaaagcgcgacggcgggaacgacgagcggcacggcggacggcacggcgaggatcgcggcgaaaaatgc

gtcggaatgcagaagaatcgcgaagtgggcgaagtgggcgaagtgggcgaagtgggcgaagtgggcgaagtgggcgaag

tgggcgaagtgggcgaagcagccgaagcgggcgaagcagccgaagcgggcgaagcagccgaagcgggcgaagcaggcga

agcaggcgaagcaggcgaagcaggcgaagcaggcggaatacgcgtcggagcgcaccgcgcgcgccccgacggcgccgcg

ccgcgtcacgccagatag

>Bucl2_Bps_MSHR305.seq Created: Thursday, January 02, 2014 11:35 AM

ttgagcgcagcatcggatttcatcgtcgtctcctcgccgaccggtttcgataaacggccgcccgcggtcaagcgcgcgc

ggcggcgcatgcaaaccagcatcgcgcattacgcggcgcggaccttgatgcgcgtcaacgaagggaaaatgacagacga

atgcgcggcctggcgttcgcgcgacgtcgacggccgaggcgacgcggagaaaggcaggctgcgatcggcccggcggccg

tccggccgccgccgaaccggcgggaagcgacgtcaacgaaaacgggccgcacgaagcggcgtgcgacggaaagctcgcg

ggccgtgccgcgaaaagcgcgacggcgggaacgacgagcggcacggcggacggcacggcgaggatcgcggcgaaaaatg

cgtcggaatgcagaagaatcgcgaagtgggcgaagcagccgaagcagccgaagcagccgaagcagccgaagcagccgaa

gcgggcgaagcgggcgaagtgggcgaagcagccgaagcgggcgaagcgggcgaagcaggcggaatacgcgtcggagcgc

accgcgcgcgccccgacggcgccgcgccgcgtcacgccagatag

>Bucl2_Bps_1106a.seq Created: Tuesday, December 31, 2013 10:07 AM

ttgagcgcagcatcggatttcatcgtcgtctcctcgccgaccggtttcgataaacggccgcccgcggtcaagcgcgcgc

ggcggcgcatgcaaaccagcatcgcgcattacgcggcgcggaccttgatgcgcgtcaacgaagggaaaatgacagacga

atgcgcggcctggcgttcgcgcgacgtcgacggccgaggcgacgcggagaaaggcaggccgcgatcggcccggcggccg

tccggccgccgccgaaccggcgggaagcgacgtcaacgaaaacggccgcacgaagcggcgtgcgacggaaagctcgcgg

gccgtgccgcgaaaagcgcgacggcgggaacgacgagcggcacggcggacggcacggcgaggatcgcggcgaaaaatgc

gtcggaatgcagaagaatcgcgaagtgggcgaagtgggcgaagtgggcgaagtgggcgaagtgggcgaagtgggcgaag

tgggcgaagtgggcgaagcgggcgaagcagccgaagcgggcgaagcaggcgaagcaggcgaagcaggcggaatacgcgt

cggagcgcaccgcgcgcgccccgacggcgccgcgccgcgtcacgccagatag

>Bucl2_Bps_1026b.seq Created: Saturday, November 23, 2013 3:34 PM

TTGAGCGCAGCATCGGATTTCATCGTCGTCTCCTCGCCGACCGGCTTCGATAAACGGCCGCCCGCGGTCAAGCGCGCGC

GGCGGCGCATGCAAACCAGCATCGCGCATTACGCGGCGCGGACCTTGATGCGCGTCAACGAAGGGAAAATGACAAACGA

ATGCGCGGCCTGGCGTTCGCGCGACGTCGACAGCCGAGGCGACGCGGAGAAAGGCAGGCCGCGATCGGCCCGGCGGCCG

TCCGGCCGCCGCCGAACCGGCGGGAAGCGACGTCAACGAAAACGGCCGCACGAAGCGGCGTGCGACGGAAAGCTCGCGG

GCCGTGCCGCGAAAAGCGCGACGGCGGGAACGACGAGCGGCACGGCGGACGGCACGGCGAGGATCGCGGCGAAAAATGC

GTCGGAATGCAGAAGAATCGCGAAGTGGGCGAAGTGGGCGAAGTGGGCGAAGTGGGCGAAGTGGGCGAAGTGGGCGAAG

TGGGCGAAGTGGGCGAAGTGGGCGAAGCAGCCGAAGCGGGCGAAGCAGCCGAAGCGGGCGAAGCAGCCGAAGCGGGCGA

AGCAGGCGAAGCAGGCGAAGCAGGCGAAGCAGGCGAAGCAGGCGGAATACGCGTCGGAGCGCACCGCGCGCGCCCCGAC

GGCGCCGCGCCGCGTCACGCCAGATAG

>Bucl2_Bps_1710b.seq Created: Tuesday, December 31, 2013 12:27 PM

ttgagcgcagcatcggatttcatcgtcgtctcctcgccgaccggtttcgataaacggccgcccgcggtcaagcgcgcgc

ggcggcgcatgcaaaccagcatcgcgcattacgcggcgcggaccttgatgcgcgtcaacgaagggaaaatgacagacga

atgcgcggcctggcgttcgcgcgacgtcgacggccgaggcgacgcggagaaaggcaggccgcgatcggcccggcggccg

tccggccgccgccgaaccggcgggaagcgacgtcaacgaaaacgggccgcacgaagcggcgtgcgacggaaagctcgcg

ggccgtgccgcgaaaagcgcgacggcgggaacgacgagcggcacggcggacggcacggcgaggatcgcggcgaaaaatg

cgtcggaatgcagaagaatcgcgaagtgggcgaagtgggcgaagtgggcgaagtgggcgaagtgggcgaagtgggcgaa

gtgggcgaagtgggcggagtgggcgaagtgggcgaagcagccgcagcgggcgaagcagccgaagcagccgaagcgggcg

gaatacgcgtcggagcgcaccgcgcgcgccccgacggcgccgcgccgcgtcacgccagatag

>Bucl2_Bps_BPC006.seq Created: Tuesday, December 31, 2013 2:56 PM

ttgagcgcagcatcggatttcatcgtcgtctcctcgccgaccggtttcgataaacggccgcccgcggtcaagcgcgcgc

ggcggcgcatgcaaaccagcatcgcgcattacgcggcgcggaccttgatgcgcgtcaacgaagggaaaatgacagacga

atgcgcggcctggcgttcgcgcgacgtcgacagccgaggcgacgcggagaaaggcaggccgcgatcggcccggcggccg

tccggccgccgccgaaccggcgggaagcgacgtcaacgaaaacggccgcacgaagcggcgtgcgacggaaagctcgcgg

gccgtgccgcgaaaagcgcgacggcgggaacgacgagcggcacggcggacggcacggcgaggatcgcggcgaaaaatgc

gtcggaatgcagaagaatcgcgaagtgggcgaagtgggcgaagtgggcgaagtgggcgaagtgggcgaagtgggcgaag

tgggcgaagtgggcgaagcgggcgaagcagccgaagcggcgaagcaggcgaagcaggcgaagcaggcggaatacgcgtc

ggagcgcaccgcgcgcgccccgacggcgccgcgccgcgtcacgccagatag

>A3NN74_BURP6_Bucl2_Bp_668.seq Created: Tuesday, October 29, 2013 5:33 PM

TTGAGCGCAGCATCGGATTTCATCGTCGTCTCCTCGCCGACCGGTTTCGATAAACGGCCGCCCGCGGTCAAGCGCGCGC

GGCGGCGCATGCAAACCAGCATCGCGCATTACGCGGCGCGGACCTTGATGCGCGTCAACGAAGGGAAAATGACAAACGA

ATGCGCGGCCTGGCGTTCGCGCGACGTCGACAGCCGAGGCGACGCGGAGAAAGGCAGGCCGCGATCGGCCCGGCGGCCG

TCCGGCCGCCGCCGAACCGGCGGGAAGCGACGTCAACGAAAACGGGCCGCACGAAGCGGCGTGCGACGGAAAGCTCGCG

GGCCGTGCCGCGAAAAGCGCGACGGCGGGAACGACGAGCGGCACGGCGGACGGCACGGCGAGGATCGCGGCGAAAAATG

CGTCGGAATGCAGAAGAATCGCGAAGTGGGTGAAGTGGGTGAAGTGGGTGAAGTGGGTGAAGTGGGTGAAGTGGGTGAA

GTGGGTGAAGTGGGTGAAGTGGGTGAAGTGGGTGAAGTGGGTGAAGTGGGCGAAGTGGGCGAAGTGGGCGAAGTGGGCG

AAGCAGCCGAAGCAGGCGAAGCGGGCGAAGCAGCCGAAGCGGGCGAAGTGGGCGAAGCAGCCGAAGCGGGCGGAATACG

CGTCGGAGCGCACCGCGCGCGCCCCGACGGCGCCGCGCCGCGTCACGCCAGATAG

>Bucl2_Bps_NCTC_13179.seq Created: Thursday, January 02, 2014 3:27 PM

ttgagcgcagcatcggatttcatcgtcgtctcctcgccgaccggtttcgataaacggccgcccgcggtcaagcgcgcgc

ggcggcgcatgcaaaccagcatcgcgcattacgcggcgcggaccttgatgcgcgtcaacgaagggaaaatgacagacga

atgcgcggcctggcgttcgcgcgacgtcgacggccgaggcgacgcggagaaaggcaggctgcgatcggcccggcggccg

tccggccgccgccgaaccggcgggaagcgacgtcaacgaaaacgggccgcacgaagcggcgtgcgacggaaagctcgcg

gaccgtgccgcgaaaagcgcgacggcgggaacgacgagcggcacggcggacggcacggcgaggatcgcggcgaaaaatg

cgtcggaatgcagaagaatcgcgaagtgggcgaagtgggcgaagtgggcgaagtgggcggaatacgcgtcggagcgcac

cgcgcgcgccccgacggcgccgcgccgcgtcacgccagatag

>Bucl2_Bps_MSHR146.seq Created: Wednesday, March 19, 2014 12:14 PM

TTGAGCGCAGCATCGGATTTCATCGTCGTCTCCTCGCCGACCGGTTTCGATAAACGGCCGCCCGCGGTCAAGCGCGCGC

GGCGGCGCATGCAAACCAGCATCGCGCATTACGCGGCGCGGACCTTGATGCGCGTCAACGAAGGGAAAATGACAAACGA

ATGCGCGGCCTGGCGTTCGCGCGACGTCGACGGCCGAGGCGACGCGGAGAAAGGCAGGCCGCGATCGGCCCGGCGGCCG

TCCGGCCGCCGCCGAACCGGCGGGAAGCAACGTCAACGAAAACGGGCCGCACGAAGCGGCGTGCGACGGAAAGCTCGCG

AGCCGTGCCGCGAAAAGCGCGACGGCGGGAACGACGAGCGGCACGGCGGACGGCACGGCGAGGATCGCGGCGAAAAATG

CGTCGGAATGCAGAAGAATCGCGAAGTGGGCGAAGTGGGCGAAGTGGGCGAAGTGGGCGAAGTGGGCGAAGTGGGCGAA

GTGGGCGAAGCAGCCGAAGCGGGCGAAGCAGCCGAAGCGGGCGAAGCGGGCGAAGCAGCCGAAGCGGGCGAAGCAGCCG

AAGCGGGCGAAGCGGGCGGAATACGCGTCGGAGCGCACCGCGCGCGCCCCGACGGCGCCGCGCCGCGTCACGCCAGATA

G

>Bucl2_Bps_MSHR511.seq Reverse Complement DNA Sequence Untitled Seq #14(1,642)

TTGAGCGCAGCATCGGATTTCATCGTCGTCTCCTCGCCGACCGGTTTCGATAAACGGCCGCCCGCGGTCAAGCGCGCGC

GGCGGCGCATGCAAACCAGCATCGCGCATTACGCGGCGCGGACCTTGATGCGCGTCAACGAAGGGAAAATGACAAACGA

ATGCGCGGCCTGGCGTTCGCGCGACGTCGACGGCCGAGGCGACGCGGAGAAAGGCAGGCCGCGATCGGCCCGGCGGCCG

TCCGGCCGCCGCCGAACCGGCGGGAAGCAACGTCAACGAAAACGGGCCGCACGAAGCGGCGTGCGACGGAAAGCTCGCG

AGCCGTGCCGCGAAAAGCGCGACGGCGGGAACGACGAGCGGCACGGCGGACGGCACGGCGAGGATCGCGGCGAAAAATG

CGTCGGAATGCAGAAGAATCGCGAAGTGGGCGAAGTGGGCGAAGTGGGCGAAGTGGGCGAAGTGGGCGAAGTGGGCGAA

GTGGGCGAAGTGGGCGAAGCAGCCGAAGCGGGCGAAGCAGCCGAAGCGGGCGAAGCGGGCGAAGCAGCCGAAGCGGGCG

AAGCAGCCGAAGCGGGCGAAGCGGGCGGAATACGCGTCGGAGCGCACCGCGCGCGCCCCGACGGCGCCGCGCCGCGTCA

CGCCAGATAG

>Bucl2_Bps_MSHR520.seq Created: Wednesday, March 19, 2014 12:23 PM

TTGAGCGCAGCATCGGATTTCATCGTCGTCTCCTCGCCGACCGGTTTCGATAAACGGCCGCCCGCGGTCAAGCGCGCGC

GGCGGCGCATGCAAACCAGCATCGCGCATTACGCGGCGCGGACCTTGATGCGCGTCAACGAAGGGAAAATGACAGACGA

ATGCGCGGCCTGGCGTTCGCGCGACGTCGACGGCCGAGGCGACGCGGAGAAAGGCAGGCTGCGATCGGCCCGGCGGCCG

TCCGGCCGCCGCCGAACCGGCGGGAAGCGACGTCAACGAAAACGGGCCGCACGAAGCGGCGTGCGACGGAAAGCTCGCG

GGCCGTGCCGCGAAAAGCGCGACGGCGGGAACGACGAGCGGCACGGCGGACGGCACGGCGAGGATCGCGGCGAAAAATG

CGTCGGAATGCAGAAGAATCGCGAAGTGGGCGAAGCAGCCGAAGCAGCCGAAGCAGCCGAAGCAGCCGAAGCAGCCGAA

GCGGGCGAAGCGGGCGAAGTGGGCGAAGCAGCCGAAGCGGGCGAAGCGGGCGAAGCAGGCGGAATACGCGTCGGAGCGC

ACCGCGCGCGCCCCGACGGCGCCGCGCCGCGTCACGCCAGATAG

>Bucl2_Bps_NAU20B-16.seq Reverse Complement DNA Sequence Untitled Seq #18(1,642)

TTGAGCGCAGCATCGGATTTCATCGTCGTCTCCTCGCCGACCGGTTTCGATAAACGGCCGCCCGCGGTCAAGCGCGCGC

GGCGGCGCATGCAAACCAGCATCGCGCATTACGCGGCGCGGACCTTGATGCGCGTCAACGAAGGGAAAATGACAAACGA

ATGCGCGGCCTGGCGTTCGCGCGACGTCGACGGCCGAGGCGACGCGGAGAAAGGCAGGCCGCGATCGGCCCGGCGGCCG

TCCGGCCGCCGCCGAACCGGCGGGAAGCAACGTCAACGAAAACGGGCCGCACGAAGCGGCGTGCGACGGAAAGCTCGCG

AGCCGTGCCGCGAAAAGCGCGACGGCGGGAACGACGAGCGGCACGGCGGACGGCACGGCGAGGATCGCGGCGAAAAATG

CGTCGGAATGCAGAAGAATCGCGAAGTGGGCGAAGTGGGCGAAGTGGGCGAAGTGGGCGAAGTGGGCGAAGTGGGCGAA

GTGGGCGAAGTGGGCGAAGCAGCCGAAGCGGGCGAAGCAGCCGAAGCGGGCGAAGCGGGCGAAGCAGCCGAAGCGGGCG

AAGCAGCCGAAGCGGGCGAAGCGGGCGGAATACGCGTCGGAGCGCACCGCGCGCGCCCCGACGGCGCCGCGCCGCGTCA

CGCCAGATAG

>Bucl2_Bps_NCTC_13178.seq Created: Wednesday, March 19, 2014 12:32 PM

TTGAGCGCAGCATCGGATTTCATCGTCGTCTCCTCGCCGACCGGCTTCGATAAACGGCCGCCCGCGGTCAAGCGCGCGC

GGCGGCGCATGCAAACCAGCATCGCGCATTACGCGGCGCGGACCTTGATGCGCGTCAACGAAGGGAAAATGACAGACGA

ATGCGCGGCCTGGCGTTCGCGCGACGTCGACGGCCGAGGCGACGCGGAGAAAGGCAGGCCGCGATCGGCCCGGCGGCCG

TCCGGCCGCCGCCGAACCGGCGGGAAGCGACGTCAACGAAAACGGGCCGCACGAAGCGGCGTGCGACGGAAAGCTCGCG

GGCCGTGCCGCGAAAAGCGCGACGGCGGGAACGACGAGCGGCACGGCGGACGGCACGGCGAGGATCGCGGCGAAAAATG

CGTCGGAATGCAGAAGAATCGCGAAGCGGGCGAAGCGGGCGAAGCGGGCGAAGCAGCCGAAGCAGCCGAAGCAGCCGAA

GCAGCCGAAGCAGCCGAAGCGGGCGAAGCGGGCGAAGCGGGCGGAATACGCGTCGGAGCGCACCGCGCGCGCCCCGACG

GCGCCGCGCCGCGTCACGCCAGATAG

>B7CQL7_BURPS_Bucl2_Bps_576.seq Created: Monday, January 20, 2014 4:46 PM

TTGAGCGCAGCATCGGATTTCATCGTCGTCTCCTCGCCGACCGGTTTCGATAAACGGCCGCCCGCGGTCAAGCGCGCGC

GGCGGCGCATGCAAACCAGCATCGCGCATTACGCGGCGCGGACCTTGATGCGCGTCAACGAAGGGAAAATGACAGACGA

ATGCGCGGCCTGGCGTTCGCGCGACGTCGACGGCCGAGGCGACGCGGAGAAAGGCAGGCCGCGATCGGCCCGGCGGCCG

TCCGGCCGCCGCCGAACCGGCGGGAAGCGACGTCAACGAAAACGGGCCGCACGAAGCGGCGTGCGACGGAAAGCTCGCG

GGCCGTGCCGCGAAAAGCGCGACGGCGGGAACGACGAGCGGCACGGCGGACGGCACGGCGAGGATCGCGGCGAAAAATG

CGTCGGAATGCAGAAGAATCGCGAAGTGGGCGAAGTGGGCGAAGTGGGCGAAGTGGGCGAAGTGGGCGAAGTGGGCGAA

GCAGCCGAAGCGGGCGAAGCAGCCGAAGCGGGCGAAGCGGGCGAAGCAGCCGAAGCAGCCGAAGCGGGCGAAGCAGGCG

AAGCAGGCGAAGCAGGCGAAGCAGGCGAAGCAGGCGAAGCAGGCGAAGCAGGCGGAATACGCGTCGGAGCGCACCGCGC

GCGCCCCGACGGCGCCGCGCCGCGTCACGCCAGATAG

>C0Y6X0_BURPS_Bucl2_Bps_Pakistan_9.seq Created: Monday, January 20, 2014 4:51 PM

TTGAGCGCAGCATCGGATTTCATCGTCGTCTCCTCGCCGACCGGTTTCGATAAACGGCCGCCCGCGGTCAAGCGCGCGC

GGCGGCGCATGCAAACCAGCATCGCGCATTACGCGGCGCGGACCTTGATGCGCGTCAACGAAGGGAAAATGACAGACGA

ATGCGCGGCCTGGCGTTCGCGCGACGTCGACGGCCGAGGCGACGCGGAGAAAGGCAGGCCGCGATCGGCCCGGCGGCCG

TCCGGCCGCCGCCGAACCGGCGGGAAGCGACGTCAACGAAAACGGGCCGCACGAAGCGGCGTGCGACGGAAAGCTCGCG

GGCCGTGCCGCGAAAAGCGCGACGGCGGGAACGACGAGCGGCACGGCGGACGGCACGGCGAGGATCGCGGCGAAAAATG

CGTCGGAATGCAGAAGAATCGCGAAGTGGGCGAAGTGGGCGAAGTGGGCGAAGTGGGCGAAGTGGGCGAAGTGGGCGAA

GTGGGCGAAGTGGGCGAAGTGGGCGAAGTGGGCGAAGTGGGCGAAGTGGGCGAAGCAGCCGAAGCGGGCGAAGCAGCCG

AAGCAGCCGAAGCGGGCGAAGCAGGCGAAGCAGGCGAAGCAGGCGAAGCAGGCGAAGCAGGCGGAATACGCGTCGGAGC

GCACCGCGCGCGCCCCGACGGCGCCGCGCCGCGTCACGCCAGATAG

>A8EN62_BURPS_Bucl2_Bp_406e.seq Reverse Complement DNA Sequence Untitled Seq #4(1,660)

TTGAGCGCAGCATCGGATTTCATCGTCGTCTCCTCGCCGACCGGTTTCGATAAACGGCCGCCCGCGGTCAAGCGCGCGC

GGCGGCGCATGCAAACCAGCATCGCGCATTACGCGGCGCGGACCTTGATGCGCGTCAACGAAGGGAAAATGACAGACGA

ATGCGCGGCCTGGCGTTCGCGCGACGTCGACGGCCGAGGCGACGCGGAGAAAGGCAGGCCGCGATCGGCCCGGCGGCCG

TCCGGCCGCCGCCGAACCGGCGGGAAGCGACGTCAACGAAAACGGGCCGCACGAAGCGGCGTGCGACGGAAAGCTCGCG

GGCCGTGCCGCGAAAAGCGCGACGGCGGGAACGACGAGCGGCACGGCGGACGGCACGGCGAGGATCGCGGCGAAAAATG

CGTCGGAATGCAGAAGAATCGCGAAGTGGGCGAAGTGGGCGAAGTGGGCGAAGTGGGCGAAGTGGGCGAAGTGGGCGAA

GTGGGCGAAGTGGGCGAAGTGGGCGAAGCGGGCGAAGCAGCCGAAGCGGGCGAAGCGGGCGAAGCGGGCGAAGCGGGCG

AAGCGGGCGAAGCGGGCGAAGCGGGCGAAGCGGGCGAAGCAGGCGGAATACGCGTCGGAGCGCACCGCGCGCGCCCCGA

CGGCGCCGCGCCGCGTCACGCCAGATAG

>Bucl2_Bm_2000031281.seq Created: Saturday, March 22, 2014 7:13 PM

TTGAGCGCAGCATCGGATTTCATCGTCGTCTCCTCGCCGACCGGCTTCGATAAACGGCCGCCCGCGGTCAAGCGCGCGC

GGCGGCGCATGCAAACCAGCATCGCGCATTACGCGGTGCGGACCTTGATGCGCGTCAACGAAGGGAAAATGACAGACGA

ATGCGCGGCCTGGCGTTCGCGCGACGTCGACGGCCGAGGCGACGCGGAGAAAGGCAGGCCGCGATCGGCCCGGCGGCCG

TCCGGCCGCCGCCGAACCGGCGGGAAGCGACGTCAACGAAAACGGGCCGCACGAAGCGGCGTGCGACGGAAAGCTCGCG

GGCCGTGCCGCGAAAAGCGCGACGGCGGGAACGACGAGCGGCACGGCGGACGGCACGGCGAGGATCGCGGCGAAAAATG

CGTCGGAATGCAGAAGAATCGCGAAGTGGGCGAAGTGGGCGAAGTGGGCGAAGTGGGCGAAGCAGCCGCAGCGGGCGAA

GCAGCCGAAGCAGCCGAAGCGGGCGGAATACGCGTCGGAGCGCACCGCGCGCGCCCCGACGGCGCCGCGCCGCGTCACG

CCAGATAG

>Bucl2_Bm_2002721280.seq Created: Saturday, March 22, 2014 7:29 PM

TTGAGCGCAGCATCGGATTTCATCGTCGTCTCCTCGCCGACCGGCTTCGATAAACGGCCGCCCGCGGTCAAGCGCGCGC

GGCGGCGCATGCAAACCAGCATCGCGCATTACGCGGTGCGGACCTTGATGCGCGTCAACGAAGGGAAAATGACAGACGA

ATGCGCGGCCTGGCGTTCGCGCGACGTCGACGGCCGAGGCGACGCGGAGAAAGGCAGGCCGCGATCGGCCCGGCGGCCG

TCCGGCCGCCGCCGAACCGGCGGGAAGCGACGTCAACGAAAACGGGCCGCACGAAGCGGCGTGCGACGGAAAGCTCGCG

GGCCGTGCCGCGAAAAGCGCGACGGCGGGAACGACGAGCGGCACGGCGGACGGCACGGCGAGGATCGCGGCGAAAAATG

CGTCGGAATGCAGAAGAATCGCGAAGTGGGCGAAGTGGGCGAAGTGGGCGAAGTGGGCGAAGCAGCCGCAGCGGGCGAA

GCAGCCGAAGCAGCCGAAGCGGGCGGAATACGCGTCGGAGCGCACCGCGCGCGCCCCGACGGCGCCGCGCCGCGTCACG

CCAGATAG

>Bucl2_Bm_China_7.seq Created: Saturday, March 22, 2014 7:18 PM

TTGAGCGCAGCATCGGATTTCATCGTCGTCTCCTCGCCGACCGGCTTCGATAAACGGCCGCCCGCGGTCAAGCGCGCGC

GGCGGCGCATGCAAACCAGCATCGCGCATTACGCGGTGCGGACCTTGATGCGCGTCAACGAAGGGAAAATGACAGACGA

ATGCGCGGCCTGGCGTTCGCGCGACGTCGACGGCCGAGGCGACGCGGAGAAAGGCAGGCCGCGATCGGCCCGGCGGCCG

TCCGGCCGCCGCCGAACCGGCGGGAAGCGACGTCAACGAAAACGGGCCGCACGAAGCGGCGTGCGACGGAAAGCTCGCG

GGCCGTGCCGCGAAAAGCGCGACGGCGGGAACGACGAGCGGCACGGCGGACGGCACGGCGAGGATCGCGGCGAAAAATG

CGTCGGAATGCAGAAGAATCGCGAAGTGGGCGAAGTGGGCGAAGTGGGCGAAGTGGGCGAAGCAGCCGCAGCGGGCGAA

GCAGCCGAAGCAGCCGAAGCGGGCGGAATACGCGTCGGAGCGCACCGCGCGCGCCCCGACGGCGCCGCGCCGCGTCACG

CCAGATAG

>Bucl2_Bm_strain_11.seq Reverse Complement DNA Sequence Untitled Seq #24(1,552)

TTGAGCGCAGCATCGGATTTCATCGTCGTCTCCTCGCCGACCGGCTTCGATAAACGGCCGCCCGCGGTCAAGCGCGCGC

GGCGGCGCATGCAAACCAGCATCGCGCATTACGCGGTGCGGACCTTGATGCGCGTCAACGAAGGGAAAATGACAGACGA

ATGCGCGGCCTGGCGTTCGCGCGACGTCGACGGCCGAGGCGACGCGGAGAAAGGCAGGCCGCGATCGGCCCGGCGGCCG

TCCGGCCGCCGCCGAACCGGCGGGAAGCGACGTCAACGAAAACGGGCCGCACGAAGCGGCGTGCGACGGAAAGCTCGCG

GGCCGTGCCGCGAAAAGCGCGACGGCGGGAACGACGAGCGGCACGGCGGACGGCACGGCGAGGATCGCGGCGAAAAATG

CGTCGGAATGCAGAAGAATCGCGAAGTGGGCGAAGTGGGCGAAGTGGGCGAAGCAGCCGCAGCGGGCGAAGCAGCCGAA

GCAGCCGAAGCGGGCGGAATACGCGTCGGAGCGCACCGCGCGCGCCCCGACGGCGCCGCGCCGCGTCACGCCAGATAG

>Bucl2_Bm_NCTC_10247.seq Created: Friday, January 03, 2014 5:44 PM

ttgagcgcagcatcggatttcatcgtcgtctcctcgccgaccggcttcgataaacggccgcccgcggtcaagcgcgcgc

ggcggcgcatgcaaaccagcatcgcgcattacgcggtgcggaccttgatgcgcgtcaacgaagggaaaatgacagacga

atgcgcggcctggcgttcgcgcgacgtcgacggccgaggcgacgcggagaaaggcaggccgcgatcggcccggcggccg

tccggccgccgccgaaccggcgggaagcgacgtcaacgaaaacgggccgcacgaagcggcgtgcgacggaaagctcgcg

ggccgtgccgcgaaaagcgcgacggcgggaacgacgagcggcacggcggacggcacggcgaggatcgcggcgaaaaatg

cgtcggaatgcagaagaatcgcgaagtgggcgaagtgggcgaagtgggcgaagcagccgcagcgggcgaagcagccgaa

gcagccgaagcgggcggaatacgcgtcggagcgcaccgcgcgcgccccgacggcgccgcgccgcgtcacgccagatag

>Bucl2_Bm_FMH.seq Reverse Complement DNA Sequence Untitled Seq #18(1,561)

TTGAGCGCAGCATCGGATTTCATCGTCGTCTCCTCGCCGACCGGCTTCGATAAACGGCCGCCCGCGGTCAAGCGCGCGC

GGCGGCGCATGCAAACCAGCATCGCGCATTACGCGGTGCGGACCTTGATGCGCGTCAACGAAGGGAAAATGACAGACGA

ATGCGCGGCCTGGCGTTCGCGCGACGTCGACGGCCGAGGCGACGCGGAGAAAGGCAGGCCGCGATCGGCCCGGCGGCCG

TCCGGCCGCCGCCGAACCGGCGGGAAGCGACGTCAACGAAAACGGGCCGCACGAAGCGGCGTGCGACGGAAAGCTCGCG

GGCCGTGCCGCGAAAAGCGCGACGGCGGGAACGACGAGCGGCACGGCGGACGGCACGGCGAGGATCGCGGCGAAAAATG

CGTCGGAATGCAGAAGAATCGCGAAGTGGGCGAAGTGGGCGAAGTGGGCGAAGTGGGCGAAGCAGCCGCAGCGGGCGAA

GCAGCCGAAGCAGCCGAAGCGGGCGGAATACGCGTCGGAGCGCACCGCGCGCGCCCCGACGGCGCCGCGCCGCGTCACG

CCAGATAG

>Bucl2_Bm_JHU.seq Reverse Complement DNA Sequence Untitled Seq #15(1,561)

TTGAGCGCAGCATCGGATTTCATCGTCGTCTCCTCGCCGACCGGCTTCGATAAACGGCCGCCCGCGGTCAAGCGCGCGC

GGCGGCGCATGCAAACCAGCATCGCGCATTACGCGGTGCGGACCTTGATGCGCGTCAACGAAGGGAAAATGACAGACGA

ATGCGCGGCCTGGCGTTCGCGCGACGTCGACGGCCGAGGCGACGCGGAGAAAGGCAGGCCGCGATCGGCCCGGCGGCCG

TCCGGCCGCCGCCGAACCGGCGGGAAGCGACGTCAACGAAAACGGGCCGCACGAAGCGGCGTGCGACGGAAAGCTCGCG

GGCCGTGCCGCGAAAAGCGCGACGGCGGGAACGACGAGCGGCACGGCGGACGGCACGGCGAGGATCGCGGCGAAAAATG

CGTCGGAATGCAGAAGAATCGCGAAGTGGGCGAAGTGGGCGAAGTGGGCGAAGTGGGCGAAGCAGCCGCAGCGGGCGAA

GCAGCCGAAGCAGCCGAAGCGGGCGGAATACGCGTCGGAGCGCACCGCGCGCGCCCCGACGGCGCCGCGCCGCGTCACG

CCAGATAG

>Bucl2_Bm_ATCC_10399.seq Reverse Complement DNA Sequence Untitled Seq #17(1,588)

TTGAGCGCAGCATCGGATTTCATCGTCGTCTCCTCGCCGACCGGCTTCGATAAACGGCCGCCCGCGGTCAAGCGCGCGC

GGCGGCGCATGCAAACCAGCATCGCGCATTACGCGGTGCGGACCTTGATGCGCGTCAACGAAGGGAAAATGACAGACGA

ATGCGCGGCCTGGCGTTCGCGCGACGTCGACGGCCGAGGCGACGCGGAGAAAGGCAGGCCGCGATCGGCCCGGCGGCCG

TCCGGCCGCCGCCGAACCGGCGGGAAGCGACGTCAACGAAAACGGGCCGCACGAAGCGGCGTGCGACGGAAAGCTCGCG

GGCCGTGCCGCGAAAAGCGCGACGGCGGGAACGACGAGCGGCACGGCGGACGGCACGGCGAGGATCGCGGCGAAAAATG

CGTCGGAATGCAGAAGAATCGCGAAGTGGGCGAAGTGGGCGAAGTGGGCGAAGTGGGCGAAGTGGGCGAAGTGGGCGAA

GTGGGCGAAGCAGCCGCAGCGGGCGAAGCAGCCGAAGCAGCCGAAGCGGGCGGAATACGCGTCGGAGCGCACCGCGCGC

GCCCCGACGGCGCCGCGCCGCGTCACGCCAGATAG

>Bucl2_Bm_GB8_horse_4.seq Reverse Complement DNA Sequence Untitled Seq #21(1,561)

TTGAGCGCAGCATCGGATTTCATCGTCGTCTCCTCGCCGACCGGCTTCGATAAACGGCCGCCCGCGGTCAAGCGCGCGC

GGCGGCGCATGCAAACCAGCATCGCGCATTACGCGGTGCGGACCTTGATGCGCGTCAACGAAGGGAAAATGACAGACGA

ATGCGCGGCCTGGCGTTCGCGCGACGTCGACGGCCGAGGCGACGCGGAGAAAGGCAGGCCGCGATCGGCCCGGCGGCCG

TCCGGCCGCCGCCGAACCGGCGGGAAGCGACGTCAACGAAAACGGGCCGCACGAAGCGGCGTGCGACGGAAAGCTCGCG

GGCCGTGCCGCGAAAAGCGCGACGGCGGGAACGACGAGCGGCACGGCGGACGGCACGGCGAGGATCGCGGCGAAAAATG

CGTCGGAATGCAGAAGAATCGCGAAGTGGGCGAAGTGGGCGAAGTGGGCGAAGTGGGCGAAGCAGCCGCAGCGGGCGAA

GCAGCCGAAGCAGCCGAAGCGGGCGGAATACGCGTCGGAGCGCACCGCGCGCGCCCCGACGGCGCCGCGCCGCGTCACG

CCAGATAG

>Bucl2_Bm_ATCC_23344.seq Created: Friday, January 03, 2014 1:21 PM

ttgagcgcagcatcggatttcatcgtcgtctcctcgccgaccggcttcgataaacggccgcccgcggtcaagcgcgcgc

ggcggcgcatgcaaaccagcatcgcgcattacgcggtgcggaccttgatgcgcgtcaacgaagggaaaatgacagacga

atgcgcggcctggcgttcgcgcgacgtcgacggccgaggcgacgcggagaaaggcaggccgcgatcggcccggcggccg

tccggccgccgccgaaccggcgggaagcgacgtcaacgaaaacgggccgcacgaagcggcgtgcgacggaaagctcgcg

ggccgtgccgcgaaaagcgcgacggcgggaacgacgagcggcacggcggacggcacggcgaggatcgcggcgaaaaatg

cgtcggaatgcagaagaatcgcgaagtgggcgaagtgggcgaagtgggcgaagtgggcgaagcagccgcagcgggcgaa

gcagccgaagcagccgaagcgggcggaatacgcgtcggagcgcaccgcgcgcgccccgacggcgccgcgccgcgtcacg

ccagatag

>Bucl2_Bm_SAVP1.seq Created: Saturday, January 04, 2014 12:40 PM

ttgagcgcagcatcggatttcatcgtcgtctcctcgccgaccggcttcgataaacggccgcccgcggtcaagcgcgcgc

ggcggcgcatgcaaaccagcatcgcgcattacgcggtgcggaccttgatgcgcgtcaacgaagggaaaatgacagacga

atgcgcggcctggcgttcgcgcgacgtcgacggccgaggcgacgcggagaaaggcaggccgcgatcggcccggcggccg

tccggccgccgccgaaccggcgggaagcgacgtcaacgaaaacgggccgcacgaagcggcgtgcgacggaaagctcgcg

ggccgtgccgcgaaaagcgcgacggcgggaacgacgagcggcacggcggacggcacggcgaggatcgcggcgaaaaatg

cgtcggaatgcagaagaatcgcgaagtgggcgaagtgggcgaagtgggcgaagcagccgcagcgggcgaagcagccgaa

gcagccgaagcgggcggaatacgcgtcggagcgcaccgcgcgcgccccgacggcgccgcgccgcgtcacgccagatag

>Bucl3_Bps_1026b.seq Created: Wednesday, November 20, 2013 11:10 AM

ATGCAAAAACTGTTCAATAAATCGGCCATCGCAATCGGCATTTCCACCCTTCTCGTTCTGTCCGGCTGCGGCTCGGTCG

ACGGCCCGTCGCTGTCGGCCAGCGGCGTCAAGCCCGCCACCGTCGGCAATACGGGGACTTCGGGGACGTCCGGCACTTC

CGGTACGTCGGGTACTTCCGGCACGTCCGGCACCTCCGGTACGTCTGGCACGTCCGGTACGTCTGGCACCTCCGGCACC

TCCGGCACGTCGGGTACTTCCGGTACGTCTGGCACCTCCGGCACGTCTGGCACCTCCGGTACGTCCGGCACCTCCGGCA

CGTCTGGCACCTCCGGTACGTCCGGCACCTCCGGCACCTCCGGCACGTCTGGCACGTCTGGCACCTCCGGCACGTCGGG

TACTTCCGGCACGTCGGGCACCTCCGGTACGTCGGGTACTTCCGGTACGTCCGGCACCTCTGGCACGTCCGGCACCTCC

GGTACGTCGGGTACCTCCGGCACGTCAGGCACTTCCGGTACGTCGGGCACGTCCGGCACCGCCTCCTCCCCGCTCGGCA

ACATCCTCGCGCAAACCGGCAACCTGATCACCGCGACGGGCACCGTCGTCTCCGCCACCGGCAACCAAATCTCCGGCAC

GTCGGTGCCGGGCGTGAACGGCGCGACGACGACGAACCTCGGCAACGCCGTCAGCTCGCTCGGCAACGGCGTCCAGACG

CTCGGCAACGGCACAGCCGCCGGCCTCGGCACGCTCGGCACGTCGACGAATCCGCTCGGCCCGACGCTCACGTCGACCT

CCGGCGTCGTCTCGAACGTCGGCAACGCGGTCTCGTCGCTCGGCGGCGTCGTGACGAGCATCGGCACCGGCCCGCTCGC

GCCGCTCGCGCCCGTCACGTCGCCGCTCGGCGGCGCGGTCGGCACGCTCGGCAGCACCGTCACGCAAGTCGGCTCCGGC

CTGAACAACGTGCTCACGAGCGCGCCCGTCCAGCAACTCGAAACCGGCGTCAGCTCGATCATCAACCCGATCTCGAACG

TCGTTTCCGGCACGACGCAGACGATCGGCACCGCCACCGGGCTCGGCGCACCCGTCAATAATCTGCTGACCACCGTCGG

CAATGGGCTGAACGCGGCCGGCGCGAAGGTATCGGGCTCGACGAACAACCAGGTGGTGCAGGCGGTCGGCGGCGTCGTC

AGCCAGCTCGGCAACACGGTCACGAGCGTCGGCGGTCTGCTGACGGGCGGCACCACCAACCCGCTCGCACCGATCACCG

GCGCGCTGTCCGGCGGCGCCACCAACCCGCTCGCGCCGATCACCGGCATCGCCGGTTCGCTGAGCGGCACGCTCGGCGC

CAACGCGGGCCTGAACGCAGGCGCGACGCAACCCGGCACGACGACGAACCCGCTCGCCCCCGTCACCGGCCTGCTCGGC

GGCCTGTCGGGTTCGGTGGGCGGCAACGCGGCGGCCGGCCTCACCGCCGTGCTCGCGCCCATCACGAATGTCGTCGGCT

CGCTGACCGCCGGCGTGAACGGCACCGTCGCCGCCCCGTCGCCCGCTCCGACCACCGGCTCGACGACGACCGCGCCCGC

CACGGGTGTGGTCGGCAGCCTGACGGGCGGCACGACCGCGAGCGGCACGTCGACGACGAACCTGCTCGCGCCCGTGACC

AACCTGATCGGCGGGCTGCTCGGCGGCGTGTCGGGCAAGTAG

>Bucl3_Bt_E264.seq Reverse Complement DNA Sequence Untitled Seq #16(1,1806)

ATGCAAAAACTGTTCAATAAATCGGCCATCGCAATCGGCATTTCCACCCTTCTCGCTCTGTCCGGCTGCGGCTCGGTCG

ACGGCCCGTCGCTGTCGGCCGGCGGCGTCAAGCCCGCCACCGTCGGCAATTCGGGGACTTCGGGGACCTCCGGTACATC

GGGCACTTCCGGCACGTCGGGTTCGGGCGGCACGTCCGGATCGTCGGGTTCGGGGACTTCGGGCACGTCGGGGACTTCG

GGCACCTCGGGGACCTCCGGTACGTCGGGCACTTCCGGTACGTCGGGCACTTCCGGCACCTCGGGGACTTCCGGTACCT

CGGGCACCTCCGGCACCTCGGGGACTTCCGGCACCTCGGGCACTTCCGGTACGTCGGGCACTTCCGGCACCTCGGGGAC

TTCCGGCACCTCGGGGACTTCCGGTACGTCGGGCACCTCCGGTACGTCGGGCACCTCCGGTACGTCGGGCACCTCCGGC

ACGTCGGGGACCTCCGGTACGTCGGGCACCTCCGGTACGTCGGGCACCTCCGGTACGTCGGGCACCTCCGGTACGTCGG

GCACCTCCGGTACGTCGGGCACCTCCGGTACGTCGGGCACCTCCGGTACGTCGGGCACCTCCGGTACGTCGGGCACCTC

CGGCACCGCCTCCTCCCCGCTCGGCAATATCCTCGCGCAGACCGGCAACCTGATCACCGCGACGGGCACCGTCGTCTCC

GCCACCGGCAACCAGATCTCTGGGACGTCGGTGCCGGGCGTCAACAGCGCGACGACCACGAACCTCGGCAACGCCGTCA

GCTCGCTCGGCAACGGCGTCCAGACGCTCGGCAACGGCACCGCCGCCGGCCTCGGCACGCTCGGCACGTCGACGAACCC

GCTCGGGCCGACGCTCACGTCGACATCGGGCGTCGTATCGAACGTCGGCAATGCGGTTTCGTCGCTCGGCGGCGTCGTG

ACGAGCATCGGCACCGGCCCGCTCGCGCCGCTCGCCCCCGTCACGTCGCCGCTCGGCGGCGCGGTCGGCACGCTCGGCA

GCACCGTCACGCAGGTCGGCTCAGGCCTGAACAACGTGCTCACGAGCGCGCCCATCCAGCAGCTCGAGACGGGCGTCAG

CTCGATCATCAATCCGATCACGAACGCCGTCTCCGGCACGACGCAGACGATCGGCACCGCCACCGGCCTCGGCGCGCCG

GTCAACAACCTGCTGACCACCGTCGGCAACGGGCTGAACGCGGCCGGCACGAAGGTGTCGGGCTCGACGAACAACCAGG

TGGTCCAGGCGGTCGGCGGCGTCGTCAGCCAGCTCGGCAACACGGTCACGAGCGTCGGCGGCCTGCTGACGGGCGGCAC

GACCAATCCGCTTGCGCCGATCACCGGCGTGCTGTCCGGCGGCTCGACCAACCCGCTCGCGCCGATCACCGGCGTCGCG

GGCTCGCTGAGCGGCACGCTCGGCGCCAACGCGGGCCTCACGGCGGGCACGACGCAGCCGGGCGCGACGACGAATCCGC

TCGCGCCCGTCACCGGCCTGCTCGGCGGCCTGTCGGGTTCGGCGGGCGGCAACGCGACGGCGGGCCTGACCGCCGCGCT

CGCGCCCGTCACGAATCTCGTCGGCTCGCTGACCGCCGGCATGACCGGCGCCGTCGCGGCAACGCCGCCCGCCACGACC

GCCGGCTCGACGACGACCGCGCCCGCCACGGGCGTCGTCGGCAGCCTGACCGGCGGCGCGACCGCGAGCGGCACGTCGA

CGACGAACCTGCTCGCGCCCGTGACCAACCTGATCGGCGGGCTGCTCGGCAGCGTGTCGGGCAAGTAG

>Bucl3_Bt_MSMB121.seq Reverse Complement DNA Sequence Untitled Seq #29(1,1800)

ATGCAAAAACTGTTCAATCAATCGGCCATCGCAATCGGCGTTTCCGCACTCCTCGTCCTGTCGGGCTGCGGCTCGGTCG

ACGGCCCGTCGTCGCCGTCCAGCGGCGTCAAGCCCACTACCGTCGGCAATACGGGAACGTCGGGCACGTCCGGCACCTC

GGGTTCGGGCGGCACGTCCGGATCGTCGGGTTCGGGCACTTCCGGGACTTCCGGGACTTCCGGGACTTCCGGGACTTCG

GGCACTTCGGGCACTTCGGGTACTTCGGGTACTTCGGGTACTTCGGGTACTTCGGGTACTTCGGGTACTTCGGGTACTT

CGGGTACTTCGGGTACTTCGGGTACTTCGGGTACTTCGGGTACTTCGGGTACTTCGGGTACTTCGGGTACTTCGGGTAC

TTCGGGTACTTCGGGCACTTCCGGCACTTCCGGCACTTCCGGCACTTCGGGCACTTCGGGCACTTCGGGCACTTCCGGC

ACTTCGGGCACTTCCGGCACTTCGGGCACTTCGGGCACTTCGGGCACTTCGGGCACTTCGGGCACTTCGGGCACTTCGG

GCACTTCGGGCACTTCCGGCACTTCGGGCACTTCGGGCACTTCGGGCACTTCCGGCACTTCCGGCACCTCCGGCACCGC

CTCCTCCCCGCTCGGCAACATCCTCTCGCAGACCGGCAACCTGATCACCGCGACGGGCACCGTCGTCTCCGCCACCGGC

AACCAGATCTCCGGCACGTCGGTGCCGGGCGTCAACGGCGCGACGACCACGAACCTCGGCAACGCCGTCAGCTCGCTCG

GCAACGGCGTCCAGACGCTCGGCAACGGCACCGTCGCCGGCCTCGGCACGCTCGGCACGTCGACGAACCCGCTCGGCCC

GACGCTCACGTCGACGTCCGGCGTCGTATCGGACGTCGGCAACGCGGTCTCGTCGCTCGGCAGCGTCGTGACGAGCATC

GGCACCGGCCCCCTCGCGCCGCTCGCGCCCGTCACGTCGCCGCTCGGCGGCGTGGTCGGCACGCTCGGCAGCACCGTCA

CGCAGGTCGGCTCCGGCCTGAACAACGTGCTCACGAGCGCGCCCGTCCAGCAGCTCGAAACCGGCGTCAGCTCGATCAT

CAACCCGATCACGAACGCCGTATCCGGCACGACGCAGACGATCGGCACCGCGACCGGACTCGGCGCGCCCGTCAACAAT

CTGCTGGCCACCGTCGGCAACGGGCTGAACGCGGCCGGCGCGAAGGTGTCGGGCTCGACGAACAACCAGGTGGTCCAGG

CGGTCGGCGGCGTCGTCAGCCAGCTCGGCAACACGGTCACGAGCGTCGGCGGCCTGCTGACGGGCGGCACGACCAATCC

GCTCGCGCCGATCACCGGCGTGCTGTCCGGCGGCTCGACCAACCCGCTTGCGCCGATCACGGGCGCCGTCGGCTCGCTT

AGCGCCACGCTCGGCGTCAACGCGGGTCTCACGGCGGGCGCGACGCAGCCGGCGGGCACGGCGACGAATCCGCTCGCGC

CCGTCACCGGCCTGCTCGGCGGCCTGTCGGGTTCGGCGGGCGGCAATGCGACGGCCGGCCTGAGCGCCGTGCTCGCGCC

CGTCACGAACCTCGTCGGCTCGCTGAGCGCCGGCGTGCACGGCACCGTCGCGGCCACGCCGCCCGCGACGGCCACCGGT

TCGACGACGACCGCGCCCGTCACGGGCGTCGTCGGCAGCCTGACCGGCAGCACGACCGCGAGCGGCTCGTCGACGACGA

ACCTGCTCTCGCCCGTGACCAACCTGCTCGGCGGGCTGCTCGGCGGCGTGTCGGGCAAGTAG

>Q3JJL4_BURP1_Bucl3_Bps_1710b.seq Created: Thursday, December 22, 2011 2:35 PM

ATGCAAAAACTGTTCAATAAATCGGCCATCGCAATCGGCATTTCCACCCTTCTCGTTCTGTCCGGCTGCGGCTCGGTCG

ACGGCCCGTCGCTGTCGGCCAGCGGCGTCAAGCCCGCCACCGTCGGCAATACGGGGACTTCGGGGACGTCCGGCACTTC

CGGTACGTCGGGTACTTCCGGCACGTCCGGCACCTCCGGTACGTCAGGTACTTCCGGCACGTCCGGCACCTCCGGTACG

TCCGGCACCTCCGGTACGTCTGGCACGTCCGGTACGTCTGGCACCTCCGGCACCTCCGGCACGTCGGGTACTTCCGGTA

CGTCTGGCACCTCCGGCACGTCTGGCACCTCCGGTACGTCCGGCACCTCCGGCACGTCTGGCACCTCCGGTACGTCCGG

CACCTCCGGCACCTCCGGCACGTCTGGCACGTCCGGTACGTCTGGCACCTCCGGCACGTCGGGTACTTCCGGCACGTCG

GGCACCTCCGGTACGTCGGGTACTTCCGGTACGTCCGGTACCTCTGGCACGTCAGGCACCTCCGGTACGTCGGGTACCT

CCGGCACGTCAGGCACTTCCGGTACGTCGGGCACGTCCGGCACCGCCTCCTCCCCGCTCGGCAACATCCTCGCGCAAAC

CGGCAACCTGATCACCGCGACGGGCACCGTCGTCTCCGCCACCGGCAACCAAATCTCCGGCACGTCGGTGCCGGGCGTG

AACGGCGCGACGACGACGAACCTCGGCAACGCCGTCAGCTCGCTCGGCAACGGCGTCCAGACGCTCGGCAACGGCACAG

CCGCCGGCCTCGGCACGCTCGGCACGTCGACGAATCCGCTCGGCCCGACGCTCACGTCGACCTCCGGCGTCGTCTCGAA

CGTCGGCAACGCGGTCTCGTCGCTCGGCGGCGTCGTGACGAGCATCGGCACCGGCCCGCTCGCGCCGCTCGCGCCCGTC

ACGTCGCCGCTCGGCGGCGCGGTCGGCACGCTCGGCAGCACCGTCACGCAAGTCGGCTCCGGCCTGAACAACGTGCTCA

CGAGCGCCCCCGTCCAGCAGCTCGAAACCGGCGTCAGCTCGATCATCAACCCGATCTCGAACGTCGTTTCCGGCACGAC

GCAGACGATCGGCACCGCCACCGGGCTCGGCGCACCCGTCAATAATCTGCTGACCACCGTCGGCAATGGGCTGAACGCG

GCCGGCGCGAAGGTGTCGGGCTCGACGAACAACCAGGTGGTGCAGGCGGTCGGCGGCGTCGTCAGCCAGCTCGGCAACA

CGGTCACGAGCGTCGGCGGTCTGCTGACGGGCGGCACCACCAACCCGCTCGCACCGATCACCGGCGCGCTGTCCGGCGG

CGCCACCAACCCGCTCGCGCCGATCACCGGCATCGCCGGTTCGCTGAGCGGCACGCTCGGCGCCAACGCGGGCCTGAAC

GCAGGCGCGACGCAACCCGGCACGACGACGAACCCGCTCGCCCCCGTCACCGGCCTGCTCGGCGGCCTGTCGGGTTCGG

CGGGCGGCAACGCGGCGGCCGGCCTCACCGCCGTGCTCGCGCCCATCACGAATGTCGTCGGCTCGCTGACCGCCGGCGT

GAACGGCACCGTCGCCGCCCCGTCGCCCGCCCCGACCACCGGCTCGACGACGACCGCGCCCGCCACGGGTGTGGTCGGC

AGCCTGACGGGCGGCACGACCGCGAGCGGCACGTCGACGACGAACCTGCTCGCGCCCGTGACCAACCTGATCGGCGGGC

TGCTCGGCGGCGTGTCGGGCAAGTAG

>Q62EE4_BURMA_Bucl3_ATCC_23344.seq Reverse Complement DNA Sequence Untitled Seq #3(1,1755)

ATGCAAAAACTGTTCAATAAATCGGCCATCGCAATCGGCATTTCCACCCTTCTCGTTCTGTCCGGCTGCGGCTCGGTCG

ACGGCCCGTCGCTGTCGGCCAGCGGCGTCAAGCCCGCCACCGTCGGCAATACGGGGACTTCGGGGACGTCCGGCACTTC

CGGTACGTCGGGTACTTCCGGCACGTCCGGCACCTCCGGTACGTCAGGTACTTCCGGCACGTCCGGTACGTCCGGCACC

TCCGGTACGTCTGGCACGTCCGGTACGTCTGGCACCTCCGGCACCTCCGGCACGTCGGGTACTTCCGGTACGTCTGGCA

CCTCCGGCACGTCTGGCACCTCCGGTACGTCCGGTACGTCCGGCACCTCCGGCACGTCTGGCACCTCCGGTACGTCCGG

CACGTCTGGCACGTCTGGCACGTCTGGCACGTCCGGCACGTCTGGCACGTCTGGCACGTCTGGCACGTCCGGTACGTCT

GGCACCTCCGGCACGTCGGGTACTTCCGGCACGTCGGGCACCTCCGGTACGTCGGGTACTTCCGGTACGTCCGGCACCT

CCGGCACCTCCGGCACCTCCGGCACCTCCGGTACGTCGGGTACCTCCGGCACGTCAGGCACTTCCGGTACGTCGGGCAC

GTCCGGCACCGCCTCCTCCCCGCTCGGCAACATCCTCGCGCAAACCGGCAACCTGATCACCGCGACGGGCACCGTCGTC

TCCGCCACCGGCAACCAAATCTCCGGCACGTCGGTGCCGGGCGTGAACGGCGCGACGACGACGAACCTCGGCAACGCCG

TCAGCTCGCTCGGCAACGGCGTCCAGACGCTCGGCAACGGCACAGCCGCCGGCCTCGGCACGCTCGGCACGTCGACGAA

TCCGCTCGGCCCGACGCTCACGTCGACCTCCGGCGTCGTCTCGAACGTCGGCAACGCGGTCTCGTCGCTCGGCGGCGTC

GTGACGAGCATCGGCACCGGCCCGCTCGCGCCCGTCACGTCGCCGCTCGGCGGCGCGGTCGGCACGCTCGGCAGCACCG

TCACGCAAGTCGGCTCCGGCCTGAACAACGTGCTCACGAGCGCGCCCGTCCAGCAGCTCGAAACCGGCGTCAGCTCGAT

CATCAATCCGATCTCGAACGTCGTTTCCGGCACGACGCAGACGATCGGCACCGCCACCGGGCTCGGCGCACCCGTCAAT

AATCTGCTGACCACCGTCGGCAATGGGCTGAACGCGGCCGGCGCGAAGGTATCGGGCTCGACGAACAACCAGGTGGTGC

AGGCGGTCGGCGGCGTCGTCAGCCAGCTCGGCAACACGGTCACGAGCGTCGGCGGTCTGCTGACGGGCGGCACCACCAA

CCCGCTCGCACCGATCACCGGCATCGCCGGTTCGCTGAGCGGCACGCTCGGCGCCAACGCGGGCCTGAACGCAGGCGCG

ACGCAACCCGGCACGACGACGAACCCGCTCGCCCCCGTCACCGGCCTGCTCGGCGGCCTGTCGGGTTCGGCGGGCGGCA

ACGCGGCGGCCGGCCTCACCGCCGTGCTCGCGCCCATCACGAATGTCGTCGGCTCGCTGACCGCCGGCGTGAACGGCAC

CGTCGCCGCCCCGTCGCCCGCCCCGACCACCGGCTCGACGACGACCGCGCCCGCCACGGGTGTGGTCGGCAGCCTGACG

GGCGGCACGACCGCGAGCGGCACGTCGACGACGAACCTGCTCGCGCCCGTGACCAACCTGATCGGCGGGCTGCTCGGCG

GCGTGTCGGGCAAGTAG

>Q63IU2_BURPS_Bucl3_Bps_K96243.seq Created: Monday, April 15, 2013 2:25 PM

ATGCAAAAACTGTTCAATAAATCGGCCATCGCAATCGGCATTTCCACCCTTCTCGTTCTGTCCGGCTGCGGCTCGGTCG

ACGGCCCGTCGCTGTCGGCCAGCGGCGTCAAGCCCGCCACCGTCGGCAATACGGGGACTTCGGGGACGTCCGGCACTTC

CGGTACGTCGGGTACTTCCGGCACGTCCGGCACCTCCGGTACGTCAGGTACTTCCGGCACGTCCGGCACCTCCGGTACG

TCCGGCACCTCCGGTACGTCTGGCACGTCCGGTACGTCTGGCACCTCCGGCACCTCCGGCACGTCGGGTACTTCCGGTA

CGTCTGGCACCTCCGGCACGTCTGGCACCTCCGGTACGTCCGGCACCTCCGGCACGTCTGGCACCTCCGGTACGTCCGG

CACCTCCGGCACCTCCGGCACGTCTGGCACGTCCGGTACGTCTGGCACCTCCGGCACGTCGGGTACTTCCGGCACGTCG

GGCACCTCCGGTACGTCGGGTACTTCCGGTACGTCCGGTACCTCTGGCACGTCAGGCACCTCCGGTACGTCGGGTACCT

CCGGCACGTCAGGCACTTCCGGTACGTCGGGCACGTCCGGCACCGCCTCCTCCCCGCTCGGCAACATCCTCGCGCAAAC

CGGCAACCTGATCACCGCGACGGGCACCGTCGTCTCCGCCACCGGCAACCAAATCTCCGGCACGTCGGTGCCGGGCGTG

AACGGCGCGACGACGACGAACCTCGGCAACGCCGTCAGCTCGCTCGGCAACGGCGTCCAGACGCTCGGCAACGGCACAG

CCGCCGGCCTCGGCACGCTCGGCACGTCGACGAATCCGCTCGGCCCGACGCTCACGTCGACCTCCGGCGTCGTCTCGAA

CGTCGGCAACGCGGTCTCGTCGCTCGGCGGCGTCGTGACGAGCATCGGCACCGGCCCGCTCGCGCCGCTCGCGCCCGTC

ACGTCGCCGCTCGGCGGCGCGGTCGGCACGCTCGGCAGCACCGTCACGCAAGTCGGCTCCGGCCTGAACAACGTGCTCA

CGAGCGCCCCCGTCCAGCAGCTCGAAACCGGCGTCAGCTCGATCATCAACCCGATCTCGAACGTCGTTTCCGGCACGAC

GCAGACGATCGGCACCGCCACCGGGCTCGGCGCACCCGTCAATAATCTGCTGACCACCGTCGGCAATGGGCTGAACGCG

GCCGGCGCGAAGGTGTCGGGCTCGACGAACAACCAGGTGGTGCAGGCGGTCGGCGGCGTCGTCAGCCAGCTCGGCAACA

CGGTCACGAGCGTCGGCGGTCTGCTGACGGGCGGCACCACCAACCCGCTCGCGCCGATCACCGGCATCGCCGGTTCGCT

GAGCGGCACGCTCGGCGCCAACGCGGGCCTGAACGCAGGCGCGACGCAACCCGGCACGACGACGAACCCGCTCGCCCCC

GTCACCGGCCTGCTCGGCGGCCTGTCGGGTTCGGCGGGCGGCAACGCGGCGGCCGGCCTCACCGCCGTGCTCGCGCCCG

TCACGAATGTCGTCGGCTCGCTGACCGCCGGCGTGAACGGCACCGTCGCCGCCCCGTCGCCCGCCCCGACCACCGGCTC

GACGACGACCGCGCCCGCCACGGGTGTGGTCGGCAGCCTGACGGGCGGCACGACCGCGAGCGGCACGTCGACGACGAAC

CTGCTCGCGCCCGTGACCAACCTGATCGGCGGGCTGCTCGGCGGCGTGTCGGGCAAGTAG

>Bucl3_Bps_MSHR_305.seq Created: Wednesday, November 20, 2013 11:04 AM

ATGCAAAAACTGTTCAATAAATCGGCCATCGCAATCGGCATTTCCACCCTTCTCGTTCTGTCCGGCTGCGGCTCGGTCG

ACGGCCCGTCGCTGTCGGCCAGCGGCGTCAAGCCCGCCACCGTCGGCAATACGGGGACTTCGGGGACTTCGGGGACGTC

CGGCACTTCCGGTACGTCGGGCACGTCCGGCACCTCCGGTACGTCAGGTACTTCCGGCACGTCCGGCACCTCCGGTACG

TCCGGCACCTCCGGTACGTCTGGCACGTCCGGTACGTCTGGCACGTCCGGTACGTCTGGCACCTCCGGCACCTCCGGCA

CGTCGGGTACTTCCGGTACGTCTGGCACCTCCGGCACGTCTGGCACCTCCGGTACGTCCGGCACCTCCGGCACGTCTGG

CACCTCCGGTACGTCTGGCACGTCTGGCACGTCCGGTACGTCTGGCACCTCCGGCACGTCGGGTACTTCCGGCACGTCG

GGCACCTCCGGTACGTCGGGTACTTCCGGTACGTCCGGCACCTCTGGCACGTCCGGCACCTCCGGTACGTCGGGTACCT

CCGGCACGTCAGGCACTTCCGGTACGTCGGGCACGTCCGGCACCGCCTCCTCCCCGCTCGGCAACATCCTCGCGCAAAC

CGGCAACCTGATCACCGCGACGGGCACCGTCGTCTCCGCCACCGGCAACCAAATCTCCGGCACGTCGGTGCCGGGCGTG

AACGGCGCGACGACGACGAACCTCGGCAACGCCGTCAGCTCGCTCGGCAACGGCGTCCAGACGCTCGGCAACGGCACAG

CCGCCGGCCTCGGCACGCTCGGCACGTCGACGAATCCGCTCGGCCCGACGCTCACGTCGACCTCCGGCGTCGTCTCGAA

CGTCGGCAACGCGGTCTCGTCGCTCGGCGGCGTCGTGACGAGCATCGGCACCGGCCCGCTCGCGCCGCTCGCGCCCGTC

ACGTCGCCGCTCGGCGGCGCGGTCGGCACGCTCGGCAGCACCGTCACGCAAGTCGGCTCCGGCCTGAGCAACGTGCTCA

CGAGCGCGCCCGTCCAGCAGCTCGAAACCGGCGTCAGCTCGATCATCAACCCGATCTCGAACGTCGTTTCCGGCACGAC

GCAGACGATCGGCACCGCCACCGGGCTCGGCGCACCCGTCAATAATCTGCTGACCACCGTCGGCAATGGGCTGAACGCG

GCCGGCGCGAAGGTATCGGGCTCGACGAACAACCAGGTGGTGCAGGCGGTCGGCGGCGTCGTCAGCCAGCTCGGCAACA

CGGTCACGAGCGTCGGCGGTCTGCTGACGGGCGGCACCACCAACCCGCTCGCACCGATCACCGGCGCGCTGTCCGGCGG

CGCCACCAACCCGCTCGCGCCGATCACCGGCATCGCCGGTTCGCTGAGCGGCACGCTCGGCGCCAACGCGGGCCTGAAC

GCAGGCGCGACGCAACCCGGCACGACGACGAACCCGCTCGCCCCCGTCACCGGCCTGCTCGGCGGCCTGTCGGGTTCGG

CGGGCGGCAACGCGGCGGCCGGCCTCACCGCCGTGCTCGCGCCCATCACGAATGTCGTCGGCTCGCTGACCGCCGGCGT

GAACGGCACCGTCGCCGCCCCGTCGCCCGCTCCGACCACTGGCTCGACGACGACCGCGCCCGCCACGGGTGTGGTCGGC

AGCCTGACGGGCGGCACGACCGCGAGCGGCACGTCGACGACGAACCTGCTCGCGCCCGTGACCAACCTGATCGGCGGGC

TGCTCGGCGGCGTGTCGGGCAAGTAG

>Bucl3_Bps_668.seq Created: Monday, December 30, 2013 12:09 PM

atgcaaaaactgttcaataaatcggccatcgcaatcggcatttccacccttctcgttctgtccggctgcggctcggtcg

acggcccgtcgctgtcggccagcggcgtcaagcccgccaccgtcggcaatacggggacttcggggacgtccggtacgtc

gggtacttccggcacgtccggtacgtctggcacctccggtacgtctggcacctccggcacgtctggcacctccggcacg

tctggcacctccggcacgtctggcacctccggtacgtctggcacgtccggcacgtcgggtacttccggtacgtctggca

cctccggtacgtccggcacctccggcacctccggcacgtcgggcacgtcgggtacttccggcacgtcgggcacctccgg

tacgtctggcacgtctggcacgtcgggtacgtcgggtacttccggtacgtccggcacctccggcacgtcgggtacttcc

ggcacgtctggcacctccggcacgtcgggtacttccggcacgtccggcacctccggtacgtccggcacctccggtacgt

cgggtacttccggtacgtccggcacctctggcacgtccggcacctccggtacgtcaggtacttccggcacgtcaggcac

ttccggtacgtcgggcacgtccggcaccgcctcctccccgctcggcaacatcctcgcgcaaaccggcaacctgatcacc

gcgacgggcaccgtcgtctccgccaccggcaaccaaatctccggcacgtcggtgccgggcgtgaacggcgcgacgacga

cgaacctcggcaacgccgtcagctcgctcggcaacggcgtccagacgctcggcaacggcacagccgccggcctcggcac

gctcggcacgtcgacgaatccgctcggcccgacgctcacgtcgacctccggcgtcgtctcgaacgtcggcaacgcggtc

tcgtcgctcggcggcgtcgtgacgagcatcggcaccggcccgctcgcgccgctcgcgcccgtcacgtcgccgctcggcg

gcgcggtcggcacgctcggcagcaccgtcacgcaagtcggctccggcctgaacaacgtgctcacgagcgcgcccgtcca

gcagctcgaaaccggcgtcagctcgatcatcaacccgatctcaaacgtcgtttccggcacgacgcagacgatcggcacc

gccaccgggctcggcgcacccgtcaataatctgctgaccaccgtcggcaatgggctgaacgcggccggcgcgaaggtgt

cgggctcgacgaacaaccaggtggtgcaggcggtcggcggcgtcgtcagccagctcggcaacacggtcacgagcgtcgg

cggtctgctgacgggcggcaccaccaacccgctcgcaccgatcaccggcgcgctgtccggcggctccaccaacccgctc

gcgccgatcaccggcgcgctgtccggcggttcgaccaacccgctcgcgccgatcaccggcatcgccggttcgctgagcg

gcacgctcggcgccaacgcgggcctgaacgcaggcgcgacgcaacccggcacgacgacgaacccgctcgcccccgtcac

cggcctgctcggcggcctgtcgggttcggcgggcggcaacgcggcggccggcctcaccgccgtgctcgcgcccatcacg

aatgtcgtcggctcgctgaccgccggcgtgaacggcaccgtcgccgccccgtcgcccgccccgaccaccggctcgacga

cgaccgcgcccgccacgggtgtggtcggcagcctgacgggcggcacgaccgcgagcggcacgtcgacgacgaacctgct

cgcgcccgtgaccaacctgatcggcgggctgctcggcggcgtgtcgggcaagtaa

>Bucl3_Bps_1106a.seq Created: Tuesday, December 31, 2013 9:20 AM

atgcaaaaactgttcaataaatcggccatcgcaatcggcatttccacccttctcgttctgtccggctgcggctcggtcg

acggcccgtcgctgtcggccagcggcgtcaagcccgccaccgtcggcaatacggggacttcggggacgtccggcacttc

cggtacgtcgggcacttccggcacttccggcacttccggcacttccggcacttccggcacttccggcacttccggcact

tccggcacttccggcacttccggcacttccggcacttccggcacttccggcacttccggcacgtccggcacgtccggta

cgtcaggtacttccggcacgtccggcacctccggtacgtccggcacctccggtacgtctggcacgtccggtacgtctgg

cacctccggcacctccggcacgtcgggtacttccggtacgtctggcacctccggcacgtctggcacctccggcacgtct

ggcacctccggtacgtccggcacgtctggcacgtctggcacgtccggtacgtctggcacgtccggcacgtcgggtactt

ccggcacgtcgggcacctccggtacgtcgggtacttccggtacgtccggcacctccggcacgtccggcacctccggtac

gtcgggtacctccggcacgtcaggcacttccggtacgtcgggcacgtccggcaccgcctcctccccgctcggcaacatc

ctcgcgcaaaccggcaacctgatcaccgcgacgggcaccgtcgtctccgccaccggcaaccaaatctccggcacgtcgg

tgccgggcgtgaacggcgcgacgacgacgaacctcggcaacgccgtcagctcgctcggcaacggcgtccagacgctcgg

caacggcacagccgccggcctcggcacgctcggcacgtcgacgaatccgctcggcccgacgctcacgtcgacctccggc

gtcgtctcgaacgtcggcaacgcggtctcgtcgctcggcggcgtcgtgacgagcatcggcaccggcccgctcgcgccgc

tcgcgcccgtcacgtcgccgctcggcggcgcggtcggcacgctcggcagcaccgtcacgcaagtcggctccggcctgaa

caacgtgctcacgagcgcgcccgtccagcaactcgaaaccggcgtcagctcgatcatcaacccgatctcgaacgtcgtt

tccggcacgacgcagacgatcggcaccgccaccgggctcggcgcacccgtcaataatctgctgaccaccgtcggcaatg

ggctgaacgcggccggcgcgaaggtatcgggctcgacgaacaaccaggtggtgcaggcggtcggcggcgtcgtcagcca

gctcggcaacacggtcacgagcgtcggcggtctgctgacgggcggcaccaccaacccgctcgcaccgatcaccggcgcg

ctgtccggcggcgccaccaacccgctcgcgccgatcaccggcatcgccggttcgctgagcggcacgctcggcgccaacg

cgggcctgaacgcaggcgcgacgcaacccggcacgacgacgaacccgctcgcccccgtcaccggcctgctcggcggcct

gtcgggttcggcgggcggcaacgcggcggccggcctcaccgccgtgctcgcgcccgtcacgaatgtcgtcggctcgctg

accgccggcgtgaacggcaccgtcgccgccccgtcgcccgctccgaccaccggctcgacgacgaccgcgcccgccacgg

gtgtggtcggcagcctgacgggcggcacgaccgcgagcggcacgtcgacgacgaacctgctcgcgcccgtgaccaacct

gatcggcgggctgctcggcggcgtgtcgggcaagtag

>Bucl3_BPC006.seq Created: Tuesday, December 31, 2013 2:34 PM

atgcaaaaactgttcaataaatcggccatcgcaatcggcatttccacccttctcgttctgtccggctgcggctcggtcg

acggcccgtcgctgtcggccagcggcgtcaagcccgccaccgtcggcaatacggggacttcggggacgtccggcacttc

cggtacgtcgggcacttccggcacttccggcacttccggcacttccggcacttccggcacttccggcacttccggcact

tccggcacttccggcacttccggcacttccggcacttccggcacttccggcacttccggcacgtccggcacgtccggta

cgtcaggtacttccggcacgtccggcacctccggtacgtccggcacctccggtacgtctggcacgtccggtacgtctgg

cacctccggcacctccggcacgtcgggtacttccggtacgtctggcacctccggcacgtctggcacctccggcacgtct

ggcacctccggtacgtccggcacgtctggcacgtctggcacgtccggtacgtctggcacgtccggcacgtcgggtactt

ccggcacgtcgggcacctccggtacgtcgggtacttccggtacgtccggcacctccggcacgtccggcacctccggtac

gtcgggtacctccggcacgtcaggcacttccggtacgtcgggcacgtccggcaccgcctcctccccgctcggcaacatc

ctcgcgcaaaccggcaacctgatcaccgcgacgggcaccgtcgtctccgccaccggcaaccaaatctccggcacgtcgg

tgccgggcgtgaacggcgcgacgacgacgaacctcggcaacgccgtcagctcgctcggcaacggcgtccagacgctcgg

caacggcacagccgccggcctcggcacgctcggcacgtcgacgaatccgctcggcccgacgctcacgtcgacctccggc

gtcgtctcgaacgtcggcaacgcggtctcgtcgctcggcggcgtcgtgacgagcatcggcaccggcccgctcgcgccgc

tcgcgcccgtcacgtcgccgctcggcggcgcggtcggcacgctcggcagcaccgtcacgcaagtcggctccggcctgaa

caacgtgctcacgagcgcgcccgtccagcaactcgaaaccggcgtcagctcgatcatcaacccgatctcgaacgtcgtt

tccggcacgacgcagacgatcggcaccgccaccgggctcggcgcacccgtcaataatctgctgaccaccgtcggcaatg

ggctgaacgcggccggcgcgaaggtatcgggctcgacgaacaaccaggtggtgcaggcggtcggcggcgtcgtcagcca

gctcggcaacacggtcacgagcgtcggcggtctgctgacgggcggcaccaccaacccgctcgcaccgatcaccggcgcg

ctgtccggcggcgccaccaacccgctcgcgccgatcaccggcatcgccggttcgctgagcggcacgctcggcgccaacg

cgggcctgaacgcaggcgcgacgcaacccggcacgacgacgaacccgctcgcccccgtcaccggcctgctcggcggcct

gtcgggttcggcgggcggcaacgcggcggccggcctcaccgccgtgctcgcgcccgtcacgaatgtcgtcggctcgctg

accgccggcgtgaacggcaccgtcgccgccccgtcgcccgctccgaccaccggctcgacgacgaccgcgcccgccacgg

gtgtggtcggcagcctgacgggcggcacgaccgcgagcggcacgtcgacgacgaacctgctcgcgcccgtgaccaacct

gatcggcgggctgctcggcggcgtgtcgggcaagtag

>Bucl3_Bps_NCTC_13179.seq Created: Thursday, January 02, 2014 2:43 PM

atgcaaaaactgttcaataaatcggccatcgcaatcggcatttccacccttctcgttctgtccggctgcggctcggtcg

acggcccgtcgctgtcggccagcggcgtcaagcccgccaccgtcggcaatacggggacttcggggacttcggggacgtc

cggcacttccggtacgtcgggtacttccggcacgtccggcacgtccggcacctccggtacgtcaggtacttccggcacg

tccggcacctccggcacgtccggcacctccggtacgtccggcacctccggtacgtccggcacctccggtacgtccggca

cctccggtacgtccggcacctccggtacgtctggcacgtccggtacgtctggcacctccggcacctccggcacgtcggg

tacttccggcacgtctggcacctccggcacgtctggcacctccggtacgtccggcacctccggcacgtcgggcacgtcg

ggcacgtctggcacgtccggtacgtctggcacctccggcacgtcgggtacttccggcacgtcgggcacctccggtacgt

cgggtacttccggtacgtccggcacctctggcacgtcgggtacctccggcacgtcaggcacttccggtacgtcgggcac

ttccggtacgtcgggcacgtccggcaccgcctcctccccgctcggcaacatcctcgcgcaaaccggcaacctgatcacc

gcgacgggcaccgtcgtctccgccaccggcaaccaaatctccggcacgtcggtgccgggcgtgaacggcgcgacgacga

cgaacctcggcaacgccgtcagctcgctcggcaacggcgtccagacgctcggcaacggcacagccgccggcctcggcac

gctcggcacgtcgacgaatccgctcggcccgacgctcacgtcgacctccggcgtcgtctcgaacgtcggcaacgcggtc

tcgtcgctcggcggcgtcgtgacgagcatcggcaccggcccgctcgcgccgctcgcgcccgtcacgtcgccgctcggcg

gcgcggtcggcacgctcggcagcaccgtcacgcaagtcggctccagcctgaacaacgtgctcacgagcgcgcccgtcca

gcagctcgaaaccggcgtcagctcgatcatcaacccgatctcgaacgtcgtttccggcacgacgcagacgatcggcacc

gccaccgggctcggcgcacccgtcaataatctgctgaccaccgtcggcaatgggctgaacgcggccggcgcgaaggtat

cgggctcgacgaacaaccaggtggtgcaggcggtcggcggcgtcgtcagccagctcggcaacacggtcacgagcgtcgg

cggtctgctgacgggcggcaccaccaacccgctcgcaccgatcaccggcgcgctgtccggcggcgccaccaacccgctc

gcgccgatcaccggcatcgccggttcgctgagcggcacgctcggcgccaacgcgggcctgaacgcaggcgcgacgcaac

ccggcacgacgacgaacccgctcgcccccgtcaccggcctgctcggcggcctgtcgggttcggcgggcggcaacgccgc

ggccggcctcaccgccgtgctcgcgcccgtcacgaatgtcgtcggctcgctgaccgccggcgtgaacggcaccgtcgcc

gccccgtcgcccgctccgaccaccggctcgacgacgaccgcgcccgccacgggtgtggtcggcagcctgacgggcggca

cgaccgcgagcggcacgtcgacgacgaacctgctcgcgcccgtgaccaacctgatcggcgggctgctcggcggcgtgtc

gggcaagtag

>Bucl3_Bm_NCTC_10229.seq Created: Friday, January 03, 2014 3:03 PM

atgcaaaaactgttcaataaatcggccatcgcaatcggcatttccacccttctcgttctgtccggctgcggctcggtcg

acggcccgtcgctgtcggccagcggcgtcaagcccgccaccgtcggcaatacggggacttcggggacgtccggcacttc

cggtacgtcgggtacttccggcacgtccggcacctccggtacgtcaggtacttccggcacgtccggtacgtccggcacc

tccggtacgtctggcacgtccggtacgtctggcacctccggcacctccggcacgtcgggtacttccggtacgtctggca

cctccggcacgtctggcacctccggtacgtccggtacgtccggcacctccggcacgtctggcacctccggtacgtccgg

cacgtctggcacgtctggcacgtccggcacgtctggcacgtctggcacgtctggcacgtccggtacgtctggcacctcc

ggcacgtcgggtacttccggcacgtcgggcacctccggtacgtcgggtacttccggtacgtccggcacctccggcacct

ccggcacctccggtacgtcgggtacctccggcacgtcaggcacttccggtacgtcgggcacgtccggcaccgcctcctc

cccgctcggcaacatcctcgcgcaaaccggcaacctgatcaccgcgacgggcaccgtcgtctccgccaccggcaaccaa

atctccggcacgtcggtgccgggcgtgaacggcgcgacgacgacgaacctcggcaacgccgtcagctcgctcggcaacg

gcgtccagacgctcggcaacggcacagccgccggcctcggcacgctcggcacgtcgacgaatccgctcggcccgacgct

cacgtcgacctccggcgtcgtctcgaacgtcggcaacgcggtctcgtcgctcggcggcgtcgtgacgagcatcggcacc

ggcccgctcgcgcccgtcacgtcgccgctcggcggcgcggtcggcacgctcggcagcaccgtcacgcaagtcggctccg

gcctgaacaacgtgctcacgagcgcgcccgtccagcagctcgaaaccggcgtcagctcgatcatcaatccgatctcgaa

cgtcgtttccggcacgacgcagacgatcggcaccgccaccgggctcggcgcacccgtcaataatctgctgaccaccgtc

ggcaatgggctgaacgcggccggcgcgaaggtatcgggctcgacgaacaaccaggtggtgcaggcggtcggcggcgtcg

tcagccagctcggcaacacggtcacgagcgtcggcggtctgctgacgggcggcaccaccaacccgctcgcaccgatcac

cggcatcgccggttcgctgagcggcacgctcggcgccaacgcgggcctgaacgcaggcgcgacgcaacccggcacgacg

acgaacccgctcgcccccgtcaccggcctgctcggcggcctgtcgggttcggcgggcggcaacgcggcggccggcctca

ccgccgtgctcgcgcccatcacgaatgtcgtcggctcgctgaccgccggcgtgaacggcaccgtcgccgccccgtcgcc

cgccccgaccaccggctcgacgacgaccgcgcccgccacgggtgtggtcggcagcctgacgggcggcacgaccgcgagc

ggcacgtcgacgacgaacctgctcgcgcccgtgaccaacctgatcggcgggctgctcggcggcgtgtcgggcaagtag

>Bucl3_Bm_NCTC_10247.seq Created: Friday, January 03, 2014 5:24 PM

atgcaaaaactgttcaataaatcggccatcgcaatcggcatttccacccttctcgttctgtccggctgcggctcggtcg

acggcccgtcgctgtcggccagcggcgtcaagcccgccaccgtcggcaatacggggacttcggggacgtccggcacttc

cggtacgtcgggtacttccggcacgtccggcacctccggtacgtcaggtacttccggcacgtccggtacgtccggcacc

tccggtacgtctggcacgtccggtacgtctggcacctccggcacctccggcacgtcgggtacttccggtacgtctggca

cctccggcacgtctggcacctccggtacgtccggtacgtccggcacctccggcacgtctggcacctccggtacgtccgg

cacgtctggcacgtctggcacgtccggcacgtctggcacgtctggcacgtctggcacgtccggtacgtctggcacctcc

ggcacgtcgggtacttccggcacgtcgggcacctccggtacgtcgggtacttccggtacgtccggcacctccggcacct

ccggcacctccggtacgtcgggtacctccggcacgtcaggcacttccggtacgtcgggcacgtccggcaccgcctcctc

cccgctcggcaacatcctcgcgcaaaccggcaacctgatcaccgcgacgggcaccgtcgtctccgccaccggcaaccaa

atctccggcacgtcggtgccgggcgtgaacggcgcgacgacgacgaacctcggcaacgccgtcagctcgctcggcaacg

gcgtccagacgctcggcaacggcacagccgccggcctcggcacgctcggcacgtcgacgaatccgctcggcccgacgct

cacgtcgacctccggcgtcgtctcgaacgtcggcaacgcggtctcgtcgctcggcggcgtcgtgacgagcatcggcacc

ggcccgctcgcgcccgtcacgtcgccgctcggcggcgcggtcggcacgctcggcagcaccgtcacgcaagtcggctccg

gcctgaacaacgtgctcacgagcgcgcccgtccagcagctcgaaaccggcgtcagctcgatcatcaatccgatctcgaa

cgtcgtttccggcacgacgcagacgatcggcaccgccaccgggctcggcgcacccgtcaataatctgctgaccaccgtc

ggcaatgggctgaacgcggccggcgcgaaggtatcgggctcgacgaacaaccaggtggtgcaggcggtcggcggcgtcg

tcagccagctcggcaacacggtcacgagcgtcggcggtctgctgacgggcggcaccaccaacccgctcgcaccgatcac

cggcatcgccggttcgctgagcggcacgctcggcgccaacgcgggcctgaacgcaggcgcgacgcaacccggcacgacg

acgaacccgctcgcccccgtcaccggcctgctcggcggcctgtcgggttcggcgggcggcaacgcggcggccggcctca

ccgccgtgctcgcgcccatcacgaatgtcgtcggctcgctgaccgccggcgtgaacggcaccgtcgccgccccgtcgcc

cgccccgaccaccggctcgacgacgaccgcgcccgccacgggtgtggtcggcagcctgacgggcggcacgaccgcgagc

ggcacgtcgacgacgaacctgctcgcgcccgtgaccaacctgatcggcgggctgctcggcggcgtgtcgggcaagtag

>Bucl3_Bm_SAVP1.seq Created: Saturday, January 04, 2014 12:18 PM

atgcaaaaactgttcaataaatcggccatcgcaatcggcatttccacccttctcgttctgtccggctgcggctcggtcg

acggcccgtcgctgtcggccagcggcgtcaagcccgccaccgtcggcaatacggggacttcggggacgtccggcacttc

cggtacgtcgggtacttccggcacgtccggcacctccggtacgtcaggtacttccggcacgtccggtacgtccggcacc

tccggtacgtctggcacgtccggtacgtctggcacctccggcacctccggcacgtcgggtacttccggtacgtctggca

cctccggcacgtctggcacctccggtacgtccggtacgtccggcacctccggcacgtctggcacctccggtacgtccgg

cacgtctggcacgtctggcacgtctggcacgtctggcacgtctggcacgtctggcacgtctggcacgtccggtacgtct

ggcacctccggcacgtcgggtacttccggcacgtcgggcacctccggtacgtcgggtacttccggtacgtccggcacct

ccggcacctccggcacctccggtacgtcgggtacctccggcacgtcaggcacttccggtacgtcgggcacgtccggcac

cgcctcctccccgctcggcaacatcctcgcgcaaaccggcaacctgatcaccgcgacgggcaccgtcgtctccgccacc

ggcaaccaaatctccggcacgtcggtgccgggcgtgaacggcgcgacgacgacgaacctcggcaacgccgtcagctcgc

tcggcaacggcgtccagacgctcggcaacggcacagccgccggcctcggcacgctcggcacgtcgacgaatccgctcgg

cccgacgctcacgtcgacctccggcgtcgtctcgaacgtcggcaacgcggtctcgtcgctcggcggcgtcgtgacgagc

atcggcaccggcccgctcgcgcccgtcacgtcgccgctcggcggcgcggtcggcacgctcggcagcaccgtcacgcaag

tcggctccggcctgaacaacgtgctcacgagcgcgcccgtccagcagctcgaaaccggcgtcagctcgatcatcaatcc

gatctcgaacgtcgtttccggcacgacgcagacgatcggcaccgccaccgggctcggcgcacccgtcaataatctgctg

accaccgtcggcaatgggctgaacgcggccggcgcgaaggtatcgggctcgacgaacaaccaggtggtgcaggcggtcg

gcggcgtcgtcagccagctcggcaacacggtcacgagcgtcggcggtctgctgacgggcggcaccaccaacccgctcgc

accgatcaccggcatcgccggttcgctgagcggcacgctcggcgccaacgcgggcctgaacgcaggcgcgacgcaaccc

ggcacgacgacgaacccgctcgcccccgtcaccggcctgctcggcggcctgtcgggttcggcgggcggcaacgcggcgg

ccggcctcaccgccgtgctcgcgcccatcacgaatgtcgtcggctcgctgaccgccggcgtgaacggcaccgtcgccgc

cccgtcgcccgccccgaccaccggctcgacgacgaccgcgcccgccacgggtgtggtcggcagcctgacgggcggcacg

accgcgagcggcacgtcgacgacgaacctgctcgcgcccgtgaccaacctgatcggcgggctgctcggcggcgtgtcgg

gcaagtag

>Bucl3_Bps_MSHR146.seq Created: Wednesday, March 19, 2014 2:16 PM

ATGCAAAAACTGTTCAATAAATCGGCCATCGCAATCGGCATTTCCACCCTTCTCGTTCTGTCCGGCTGCGGCTCGGTCG

ACGGCCCGTCGCTGTCGGCCAGCGGCGTCAAGCCCGCCACCGTCGGCAATACGGGGACTTCGGGGACGTCCGGCACTTC

CGGTACGTCCGGCACTTCCGGTACGTCCGGCACTTCCGGCACTTCCGGCACGTCCGGCACCTCCGGTACGTCAGGTACT

TCCGGCACGTCCGGCACCTCCGGTACGTCTGGCACGTCCGGTACGTCTGGCACGTCCGGTACGTCTGGTACGTCTGGCA

CGTCCGGCACCTCCGGCACGTCGGGTACTTCCGGTACGTCTGGCACCTCCGGCACGTCTGGCACCTCCGGTACGTCCGG

CACCTCCGGCACGTCTGGCACCTCCGGTACGTCCGGCACGTCTGGCACGTCCGGCACGTCTGGCACGTCCGGTACGTCT

GGCACCTCCGGCACGTCGGGTACTTCCGGCACGTCGGGCACCTCCGGTACGTCGGGTACTTCCGGTACGTCCGGCACCT

CTGGCACGTCCGGCACCTCCGGTACGTCGGGTACCTCCGGCACGTCAGGCACTTCCGGTACGTCGGGCACGTCCGGCAC

CGCCTCCTCCCCGCTCGGCAACATCCTCGCGCAAACCGGCAACCTGATCACCGCGACGGGCACCGTCGTCTCCGCCACC

GGCAACCAAATCTCCGGCACGTCGGTGCCGGGCGTGAACGGCGCGACGACGACGAACCTCGGCAACGCCGTCAGCTCGC

TCGGCAACGGTGTCCAGACGCTCGGCAACGGCACAGCCGCCGGCCTCGGCACGCTCGGCACGTCGACGAATCCGCTCGG

CCCGACGCTCACGTCGACCTCCGGCGTCGTCTCGAACGTCGGCAACGCGGTCTCGTCGCTCGGCGGCGTCGTGACGAGC

ATCGGCACCGGCCCGCTCGCGCCGCTCGCGCCCGTCACGTCGCCGCTCGGCGGCGCGGTCGGCACGCTCGGCAGCACCG

TCACGCAAGTCGGCTCCGGCCTGAACAACGTGCTCACGAGCGCGCCCGTCCAGCAGCTCGAAACCGGCGTCAGCTCGAT

CATCAACCCGATCTCGAACGTCGTTTCCGGCACGACGCAGACGATCGGCACCGCCACCGGGCTCGGCGCACCCGTCAAT

AATCTGCTGACCACCGTCGGCAATGGGCTGAACGCGGCCGGCGCGAAGGTGTCGGGCTCGACGAACAACCAGGTGGTGC

AGGCGGTCGGCGGCGTCGTCAGCCAGCTCGGCAACACGGTCACGAGCGTCGGCGGTCTGCTGACGGGCGGCACCACCAA

CCCGCTCGCACCGATCACCGGCGCGCTGTCCGGCGGCGCCACCAACCCGCTCGCGCCGATCACCGGCATCGCCGGTTCG

CTGAGCGGCACGCTCGGCGCCAACGCGGGCCTGAACGCAGGCGCGACGCAACCCGGCACGACGACGAACCCGCTCGCCC

CCGTCACCGGCCTGCTCGGCGGCCTGTCGGGTTCGGCGGGCGGCAACGCGGCGGCCGGCCTCACCGCCGTGCTCGCGCC

CGTCACGAATGTCGTCGGCTCGCTGACCGCCGGCGTGAACGGCACCGTCGCCGCCCCGTCGCCCGCTCCGACCACCGGC

TCGACGACGACCGCGCCCGCCACGGGTGTGGTCGGCAGCCTGACGGGCGGCACGACCGCGAGCGGCACGTCGACGACGA

ACCTGCTCGCGCCCGTGACCAACCTGATCGGCGGGCTGCTCGGCGGCGTGTCGGGCAAGTAG

>Bucl3_Bps_MSHR511.seq Reverse Complement DNA Sequence Untitled Seq #5(1,1800)

ATGCAAAAACTGTTCAATAAATCGGCCATCGCAATCGGCATTTCCACCCTTCTCGTTCTGTCCGGCTGCGGCTCGGTCG

ACGGCCCGTCGCTGTCGGCCAGCGGCGTCAAGCCCGCCACCGTCGGCAATACGGGGACTTCGGGGACGTCCGGCACTTC

CGGTACGTCCGGCACTTCCGGTACGTCCGGCACTTCCGGCACTTCCGGCACGTCCGGCACCTCCGGTACGTCAGGTACT

TCCGGCACGTCCGGCACCTCCGGTACGTCTGGCACGTCCGGTACGTCTGGCACGTCCGGTACGTCTGGTACGTCTGGCA

CGTCCGGCACCTCCGGCACGTCGGGTACTTCCGGTACGTCTGGCACCTCCGGCACGTCTGGCACCTCCGGTACGTCCGG

CACCTCCGGCACGTCTGGCACCTCCGGTACGTCCGGCACGTCTGGCACGTCCGGCACGTCTGGCACGTCCGGTACGTCT

GGCACCTCCGGCACGTCGGGTACTTCCGGCACGTCGGGCACCTCCGGTACGTCGGGTACTTCCGGTACGTCCGGCACCT

CTGGCACGTCCGGCACCTCCGGTACGTCGGGTACCTCCGGCACGTCAGGCACTTCCGGTACGTCGGGCACGTCCGGCAC

CGCCTCCTCCCCGCTCGGCAACATCCTCGCGCAAACCGGCAACCTGATCACCGCGACGGGCACCGTCGTCTCCGCCACC

GGCAACCAAATCTCCGGCACGTCGGTGCCGGGCGTGAACGGCGCGACGACGACGAACCTCGGCAACGCCGTCAGCTCGC

TCGGCAACGGTGTCCAGACGCTCGGCAACGGCACAGCCGCCGGCCTCGGCACGCTCGGCACGTCGACGAATCCGCTCGG

CCCGACGCTCACGTCGACCTCCGGCGTCGTCTCGAACGTCGGCAACGCGGTCTCGTCGCTCGGCGGCGTCGTGACGAGC

ATCGGCACCGGCCCGCTCGCGCCGCTCGCGCCCGTCACGTCGCCGCTCGGCGGCGCGGTCGGCACGCTCGGCAGCACCG

TCACGCAAGTCGGCTCCGGCCTGAACAACGTGCTCACGAGCGCGCCCGTCCAGCAGCTCGAAACCGGCGTCAGCTCGAT

CATCAACCCGATCTCGAACGTCGTTTCCGGCACGACGCAGACGATCGGCACCGCCACCGGGCTCGGCGCACCCGTCAAT

AATCTGCTGACCACCGTCGGCAATGGGCTGAACGCGGCCGGCGCGAAGGTGTCGGGCTCGACGAACAACCAGGTGGTGC

AGGCGGTCGGCGGCGTCGTCAGCCAGCTCGGCAACACGGTCACGAGCGTCGGCGGTCTGCTGACGGGCGGCACCACCAA

CCCGCTCGCACCGATCACCGGCGCGCTGTCCGGCGGCGCCACCAACCCGCTCGCGCCGATCACCGGCATCGCCGGTTCG

CTGAGCGGCACGCTCGGCGCCAACGCGGGCCTGAACGCAGGCGCGACGCAACCCGGCACGACGACGAACCCGCTCGCCC

CCGTCACCGGCCTGCTCGGCGGCCTGTCGGGTTCGGCGGGCGGCAACGCGGCGGCCGGCCTCACCGCCGTGCTCGCGCC

CGTCACGAATGTCGTCGGCTCGCTGACCGCCGGCGTGAACGGCACCGTCGCCGCCCCGTCGCCCGCTCCGACCACCGGC

TCGACGACGACCGCGCCCGCCACGGGTGTGGTCGGCAGCCTGACGGGCGGCACGACCGCGAGCGGCACGTCGACGACGA

ACCTGCTCGCGCCCGTGACCAACCTGATCGGCGGGCTGCTCGGCGGCGTGTCGGGCAAGTAG

>Bucl3_Bps_MSHR520.seq Created: Wednesday, March 19, 2014 1:33 PM

ATGCAAAAACTGTTCAATAAATCGGCCATCGCAATCGGCATTTCCACCCTTCTCGTTCTGTCCGGCTGCGGCTCGGTCG

ACGGCCCGTCGCTGTCGGCCAGCGGCGTCAAGCCCGCCACCGTCGGCAATACGGGGACTTCGGGGACTTCGGGGACGTC

CGGCACTTCCGGTACGTCGGGCACGTCCGGCACCTCCGGTACGTCAGGTACTTCCGGCACGTCCGGCACCTCCGGTACG

TCCGGCACCTCCGGTACGTCTGGCACGTCCGGTACGTCTGGCACGTCCGGTACGTCTGGCACCTCCGGCACCTCCGGCA

CGTCGGGTACTTCCGGTACGTCTGGCACCTCCGGCACGTCTGGCACCTCCGGTACGTCCGGCACCTCCGGCACGTCTGG

CACCTCCGGTACGTCTGGCACGTCTGGCACGTCCGGTACGTCTGGCACCTCCGGCACGTCGGGTACTTCCGGCACGTCG

GGCACCTCCGGTACGTCGGGTACTTCCGGTACGTCCGGCACCTCTGGCACGTCCGGCACCTCCGGTACGTCGGGTACCT

CCGGCACGTCAGGCACTTCCGGTACGTCGGGCACGTCCGGCACCGCCTCCTCCCCGCTCGGCAACATCCTCGCGCAAAC

CGGCAACCTGATCACCGCGACGGGCACCGTCGTCTCCGCCACCGGCAACCAAATCTCCGGCACGTCGGTGCCGGGCGTG

AACGGCGCGACGACGACGAACCTCGGCAACGCCGTCAGCTCGCTCGGCAACGGCGTCCAGACGCTCGGCAACGGCACAG

CCGCCGGCCTCGGCACGCTCGGCACGTCGACGAATCCGCTCGGCCCGACGCTCACGTCGACCTCCGGCGTCGTCTCGAA

CGTCGGCAACGCGGTCTCGTCGCTCGGCGGCGTCGTGACGAGCATCGGCACCGGCCCGCTCGCGCCGCTCGCGCCCGTC

ACGTCGCCGCTCGGCGGCGCGGTCGGCACGCTCGGCAGCACCGTCACGCAAGTCGGCTCCGGCCTGAGCAACGTGCTCA

CGAGCGCGCCCGTCCAGCAGCTCGAAACCGGCGTCAGCTCGATCATCAACCCGATCTCGAACGTCGTTTCCGGCACGAC

GCAGACGATCGGCACCGCCACCGGGCTCGGCGCACCCGTCAATAATCTGCTGACCACCGTCGGCAATGGGCTGAACGCG

GCCGGCGCGAAGGTATCGGGCTCGACGAACAACCAGGTGGTGCAGGCGGTCGGCGGCGTCGTCAGCCAGCTCGGCAACA

CGGTCACGAGCGTCGGCGGTCTGCTGACGGGCGGCACCACCAACCCGCTCGCACCGATCACCGGCGCGCTGTCCGGCGG

CGCCACCAACCCGCTCGCGCCGATCACCGGCATCGCCGGTTCGCTGAGCGGCACGCTCGGCGCCAACGCGGGCCTGAAC

GCAGGCGCGACGCAACCCGGCACGACGACGAACCCGCTCGCCCCCGTCACCGGCCTGCTCGGCGGCCTGTCGGGTTCGG

CGGGCGGCAACGCGGCGGCCGGCCTCACCGCCGTGCTCGCGCCCATCACGAATGTCGTCGGCTCGCTGACCGCCGGCGT

GAACGGCACCGTCGCCGCCCCGTCGCCCGCTCCGACCACTGGCTCGACGACGACCGCGCCCGCCACGGGTGTGGTCGGC

AGCCTGACGGGCGGCACGACCGCGAGCGGCACGTCGACGACGAACCTGCTCGCGCCCGTGACCAACCTGATCGGCGGGC

TGCTCGGCGGCGTGTCGGGCAAGTAG

>Bucl3_Bps_NAU20B-16.seq Reverse Complement DNA Sequence Untitled Seq #8(1,1800)

ATGCAAAAACTGTTCAATAAATCGGCCATCGCAATCGGCATTTCCACCCTTCTCGTTCTGTCCGGCTGCGGCTCGGTCG

ACGGCCCGTCGCTGTCGGCCAGCGGCGTCAAGCCCGCCACCGTCGGCAATACGGGGACTTCGGGGACGTCCGGCACTTC

CGGTACGTCCGGCACTTCCGGTACGTCCGGCACTTCCGGCACTTCCGGCACGTCCGGCACCTCCGGTACGTCAGGTACT

TCCGGCACGTCCGGCACCTCCGGTACGTCTGGCACGTCCGGTACGTCTGGCACGTCCGGTACGTCTGGTACGTCTGGCA

CGTCCGGCACCTCCGGCACGTCGGGTACTTCCGGTACGTCTGGCACCTCCGGCACGTCTGGCACCTCCGGTACGTCCGG

CACCTCCGGCACGTCTGGCACCTCCGGTACGTCCGGCACGTCTGGCACGTCCGGCACGTCTGGCACGTCCGGTACGTCT

GGCACCTCCGGCACGTCGGGTACTTCCGGCACGTCGGGCACCTCCGGTACGTCGGGTACTTCCGGTACGTCCGGCACCT

CTGGCACGTCCGGCACCTCCGGTACGTCGGGTACCTCCGGCACGTCAGGCACTTCCGGTACGTCGGGCACGTCCGGCAC

CGCCTCCTCCCCGCTCGGCAACATCCTCGCGCAAACCGGCAACCTGATCACCGCGACGGGCACCGTCGTCTCCGCCACC

GGCAACCAAATCTCCGGCACGTCGGTGCCGGGCGTGAACGGCGCGACGACGACGAACCTCGGCAACGCCGTCAGCTCGC

TCGGCAACGGTGTCCAGACGCTCGGCAACGGCACAGCCGCCGGCCTCGGCACGCTCGGCACGTCGACGAATCCGCTCGG

CCCGACGCTCACGTCGACCTCCGGCGTCGTCTCGAACGTCGGCAACGCGGTCTCGTCGCTCGGCGGCGTCGTGACGAGC

ATCGGCACCGGCCCGCTCGCGCCGCTCGCGCCCGTCACGTCGCCGCTCGGCGGCGCGGTCGGCACGCTCGGCAGCACCG

TCACGCAAGTCGGCTCCGGCCTGAACAACGTGCTCACGAGCGCGCCCGTCCAGCAGCTCGAAACCGGCGTCAGCTCGAT

CATCAACCCGATCTCGAACGTCGTTTCCGGCACGACGCAGACGATCGGCACCGCCACCGGGCTCGGCGCACCCGTCAAT

AATCTGCTGACCACCGTCGGCAATGGGCTGAACGCGGCCGGCGCGAAGGTGTCGGGCTCGACGAACAACCAGGTGGTGC

AGGCGGTCGGCGGCGTCGTCAGCCAGCTCGGCAACACGGTCACGAGCGTCGGCGGTCTGCTGACGGGCGGCACCACCAA

CCCGCTCGCACCGATCACCGGCGCGCTGTCCGGCGGCGCCACCAACCCGCTCGCGCCGATCACCGGCATCGCCGGTTCG

CTGAGCGGCACGCTCGGCGCCAACGCGGGCCTGAACGCAGGCGCGACGCAACCCGGCACGACGACGAACCCGCTCGCCC

CCGTCACCGGCCTGCTCGGCGGCCTGTCGGGTTCGGCGGGCGGCAACGCGGCGGCCGGCCTCACCGCCGTGCTCGCGCC

CGTCACGAATGTCGTCGGCTCGCTGACCGCCGGCGTGAACGGCACCGTCGCCGCCCCGTCGCCCGCTCCGACCACCGGC

TCGACGACGACCGCGCCCGCCACGGGTGTGGTCGGCAGCCTGACGGGCGGCACGACCGCGAGCGGCACGTCGACGACGA

ACCTGCTCGCGCCCGTGACCAACCTGATCGGCGGGCTGCTCGGCGGCGTGTCGGGCAAGTAG

>Bucl3_Bps_NCTC_13178.seq Created: Wednesday, March 19, 2014 2:41 PM

ATGCAAAAACTGTTCAATAAATCGGCCATCGCAATCGGCATTTCCACCCTTCTCGTTCTGTCCGGCTGCGGCTCGGTCG

ACGGCCCGTCGCTGTCGGCCAGCGGCGTCAAGCCCGCCACCGTCGGCAATACGGGGACTTCGGGGACGTCCGGCACTTC

CGGCACTTCCGGCACTTCCGGCACTTCCGGCACTTCCGGCACTTCCGGCACGTCCGGTACGTCAGGTACTTCCGGCACG

TCCGGCACCTCCGGTACGTCCGGCACCTCCGGTACGTCTGGCACGTCCGGTACGTCTGGCACCTCCGGCACCTCCGGCA

CGTCGGGTACTTCCGGCACGTCTGGCACCTCCGGTACGTCCGGCACCTCCGGCACGTCTGGCACCTCCGGTACGTCCGG

TACGTCCGGTACGTCTGGCACGTCCGGCACGTCCGGTACGTCTGGCACCTCCGGCACGTCGGGTACTTCCGGCACGTCG

GGTACTTCCGGTACGTCCGGCACCTCTGGCACGTCCGGCACCTCCGGTACGTCGGGTACCTCCGGCACGTCAGGCACTT

CCGGTACGTCGGGCACGTCCGGCACCGCCTCCTCCCCGCTCGGCAACATCCTCGCGCAAACCGGCAACCTGATCACCGC

GACGGGCACCGTCGTCTCCGCCACCGGCAACCAAATCTCCGGCACGTCGGTGCCGGGCGTGAACGGCGCGACGACGACG

AACCTCGGCAACGCCGTCAGCTCGCTCGGCAACGGCGTCCAGACGCTCGGCAACGGCACAGCCGCCGGCCTCGGCACGC

TCGGCACGTCGACGAATCCGCTCGGCCCGACGCTCACGTCGACCTCCGGCGTCGTCTCGAACGTCGGCAACGCGGTCTC

GTCGCTCGGCGGCGTCGTGACGAGCATCGGCACCGGCCCGCTCGCGCCGCTCGCGCCCGTCACGTCGCCGCTCGGCGGC

GCGGTCGGCACGCTCGGCAGCACCGTCACGCAAGTCGGCTCCGGCCTGAACAACGTGCTCACGAGCGCGCCCGTCCAGC

AGCTCGAAACCGGCGTCAGCTCGATCATCAATCCGATCTCGAACGTCGTTTCCGGCACGACGCAGACGATCGGCACCGC

CACCGGGCTCGGCGCACCCGTCAATAATCTGCTGACCACCGTCGGCAATGGGCTGAACGCGGCTGGCGCGAAGGTATCG

GGCTCGACGAACAACCAGGTGGTGCAGGCGGTCGGCGGCGTCGTCAGCCAGCTCGGCAACACGGTCACGAGCGTCGGCG

GTCTGCTGACGGGCGGCACCACCAACCCGCTCGCACCGATCACCGGCGCGCTGTCCGGCGGCGCCACCAACCCGCTCGC

GCCGATCACCGGCATCGCCGGTTCGCTGAGCGGCACGCTCGGCGCCAACGCGGGCCTGAACGCAGGCGCGACGCAACCC

GGCACGACGACGAACCCGCTCGCCCCCGTCACCGGCCTGCTCGGCGGCCTGTCGGGTTCGGCGGGCGGCAACGCGGCGG

CCGGCCTCACCGCCGTGCTCGCGCCCGTCACGAATGTCGTCGGCTCGCTGACCGCCGGCGTGAACGGCACCGTCGCCGC

CCCGTCGCCCGCTCCGACCACCGGCTCGACGACGACCGCGCCCGCCACGGGTGTGGTCGGCAGCCTGACGGGCGGCACG

ACCGCGAGCGGCACGTCGACGACGAACCTGCTCGCGCCCGTGACCAACCTGATCGGCGGGCTGCTCGGCGGCGTGTCGG

GCAAGTAG

>Bucl3_Bm_2000031281.seq Reverse Complement DNA Sequence Untitled Seq #41(1,1755)

ATGCAAAAACTGTTCAATAAATCGGCCATCGCAATCGGCATTTCCACCCTTCTCGTTCTGTCCGGCTGCGGCTCGGTCG

ACGGCCCGTCGCTGTCGGCCAGCGGCGTCAAGCCCGCCACCGTCGGCAATACGGGGACTTCGGGGACGTCCGGCACTTC

CGGTACGTCGGGTACTTCCGGCACGTCCGGCACCTCCGGTACGTCAGGTACTTCCGGCACGTCCGGTACGTCCGGCACC

TCCGGTACGTCTGGCACGTCCGGTACGTCTGGCACCTCCGGCACCTCCGGCACGTCGGGTACTTCCGGTACGTCTGGCA

CCTCCGGCACGTCTGGCACCTCCGGTACGTCCGGTACGTCCGGCACCTCCGGCACGTCTGGCACCTCCGGTACGTCCGG

CACGTCTGGCACGTCTGGCACGTCTGGCACGTCCGGCACGTCTGGCACGTCTGGCACGTCTGGCACGTCCGGTACGTCT

GGCACCTCCGGCACGTCGGGTACTTCCGGCACGTCGGGCACCTCCGGTACGTCGGGTACTTCCGGTACGTCCGGCACCT

CCGGCACCTCCGGCACCTCCGGCACCTCCGGTACGTCGGGTACCTCCGGCACGTCAGGCACTTCCGGTACGTCGGGCAC

GTCCGGCACCGCCTCCTCCCCGCTCGGCAACATCCTCGCGCAAACCGGCAACCTGATCACCGCGACGGGCACCGTCGTC

TCCGCCACCGGCAACCAAATCTCCGGCACGTCGGTGCCGGGCGTGAACGGCGCGACGACGACGAACCTCGGCAACGCCG

TCAGCTCGCTCGGCAACGGCGTCCAGACGCTCGGCAACGGCACAGCCGCCGGCCTCGGCACGCTCGGCACGTCGACGAA

TCCGCTCGGCCCGACGCTCACGTCGACCTCCGGCGTCGTCTCGAACGTCGGCAACGCGGTCTCGTCGCTCGGCGGCGTC

GTGACGAGCATCGGCACCGGCCCGCTCGCGCCCGTCACGTCGCCGCTCGGCGGCGCGGTCGGCACGCTCGGCAGCACCG

TCACGCAAGTCGGCTCCGGCCTGAACAACGTGCTCACGAGCGCGCCCGTCCAGCAGCTCGAAACCGGCGTCAGCTCGAT

CATCAATCCGATCTCGAACGTCGTTTCCGGCACGACGCAGACGATCGGCACCGCCACCGGGCTCGGCGCACCCGTCAAT

AATCTGCTGACCACCGTCGGCAATGGGCTGAACGCGGCCGGCGCGAAGGTATCGGGCTCGACGAACAACCAGGTGGTGC

AGGCGGTCGGCGGCGTCGTCAGCCAGCTCGGCAACACGGTCACGAGCGTCGGCGGTCTGCTGACGGGCGGCACCACCAA

CCCGCTCGCACCGATCACCGGCATCGCCGGTTCGCTGAGCGGCACGCTCGGCGCCAACGCGGGCCTGAACGCAGGCGCG

ACGCAACCCGGCACGACGACGAACCCGCTCGCCCCCGTCACCGGCCTGCTCGGCGGCCTGTCGGGTTCGGCGGGCGGCA

ACGCGGCGGCCGGCCTCACCGCCGTGCTCGCGCCCATCACGAATGTCGTCGGCTCGCTGACCGCCGGCGTGAACGGCAC

CGTCGCCGCCCCGTCGCCCGCCCCGACCACCGGCTCGACGACGACCGCGCCCGCCACGGGTGTGGTCGGCAGCCTGACG

GGCGGCACGACCGCGAGCGGCACGTCGACGACGAACCTGCTCGCGCCCGTGACCAACCTGATCGGCGGGCTGCTCGGCG

GCGTGTCGGGCAAGTAG

>Bucl3_Bm_2002721280.seq Created: Sunday, March 23, 2014 5:32 PM

ATGCAAAAACTGTTCAATAAATCGGCCATCGCAATCGGCATTTCCACCCTTCTCGTTCTGTCCGGCTGCGGCTCGGTCG

ACGGCCCGTCGCTGTCGGCCAGCGGCGTCAAGCCCGCCACCGTCGGCAATACGGGGACTTCGGGGACGTCCGGCACTTC

CGGTACGTCGGGTACTTCCGGCACGTCCGGCACCTCCGGTACGTCTGGCACGTCCGGTACGTCTGGCACCTCCGGCACC

TCCGGCACGTCGGGTACTTCCGGTACGTCTGGCACCTCCGGCACGTCTGGCACCTCCGGTACGTCCGGTACGTCCGGCA

CCTCCGGCACGTCTGGCACCTCCGGTACGTCCGGCACGTCTGGCACGTCTGGCACGTCTGGCACGTCCGGCACGTCTGG

CACGTCTGGCACGTCTGGCACGTCCGGTACGTCTGGCACCTCCGGCACGTCGGGTACTTCCGGCACGTCGGGCACCTCC

GGTACGTCGGGTACTTCCGGTACGTCCGGCACCTCCGGCACCTCCGGCACCTCCGGTACGTCGGGTACCTCCGGCACGT

CAGGCACTTCCGGTACGTCGGGCACGTCCGGCACCGCCTCCTCCCCGCTCGGCAACATCCTCGCGCAAACCGGCAACCT

GATCACCGCGACGGGCACCGTCGTCTCCGCCACCGGCAACCAAATCTCCGGCACGTCGGTGCCGGGCGTGAACGGCGCG

ACGACGACGAACCTCGGCAACGCCGTCAGCTCGCTCGGCAACGGCGTCCAGACGCTCGGCAACGGCACAGCCGCCGGCC

TCGGCACGCTCGGCACGTCGACGAATCCGCTCGGCCCGACGCTCACGTCGACCTCCGGCGTCGTCTCGAACGTCGGCAA

CGCGGTCTCGTCGCTCGGCGGCGTCGTGACGAGCATCGGCACCGGCCCGCTCGCGCCCGTCACGTCGCCGCTCGGCGGC

GCGGTCGGCACGCTCGGCAGCACCGTCACGCAAGTCGGCTCCGGCCTGAACAACGTGCTCACGAGCGCGCCCGTCCAGC

AGCTCGAAACCGGCGTCAGCTCGATCATCAATCCGATCTCGAACGTCGTTTCCGGCACGACGCAGACGATCGGCACCGC

CACCGGGCTCGGCGCACCCGTCAATAATCTGCTGACCACCGTCGGCAATGGGCTGAACGCGGCCGGCGCGAAGGTATCG

GGCTCGACGAACAACCAGGTGGTGCAGGCGGTCGGCGGCGTCGTCAGCCAGCTCGGCAACACGGTCACGAGCGTCGGCG

GTCTGCTGACGGGCGGCACCACCAACCCGCTCGCACCGATCACCGGCATCGCCGGTTCGCTGAGCGGCACGCTCGGCGC

CAACGCGGGCCTGAACGCAGGCGCGACGCAACCCGGCACGACGACGAACCCGCTCGCCCCCGTCACCGGCCTGCTCGGC

GGCCTGTCGGGTTCGGCGGGCGGCAACGCGGCGGCCGGCCTCACCGCCGTGCTCGCGCCCATCACGAATGTCGTCGGCT

CGCTGACCGCCGGCGTGAACGGCACCGTCGCCGCCCCGTCGCCCGCCCCGACCACCGGCTCGACGACGACCGCGCCCGC

CACGGGTGTGGTCGGCAGCCTGACGGGCGGCACGACCGCGAGCGGCACGTCGACGACGAACCTGCTCGCGCCCGTGACC

AACCTGATCGGCGGGCTGCTCGGCGGCGTGTCGGGCAAGTAG

>Bucl3_Bm_China_7.seq Created: Sunday, March 23, 2014 6:50 PM

ATGCAAAAACTGTTCAATAAATCGGCCATCGCAATCGGCATTTCCACCCTTCTCGTTCTGTCCGGCTGCGGCTCGGTCG

ACGGCCCGTCGCTGTCGGCCAGCGGCGTCAAGCCCGCCACCGTCGGCAATACGGGGACTTCGGGGACGTCCGGCACTTC

CGGTACGTCGGGTACTTCCGGCACGTCCGGCACCTCCGGTACGTCAGGTACTTCCGGCACGTCCGGTACGTCCGGCACC

TCCGGTACGTCTGGCACGTCCGGTACGTCTGGCACCTCCGGCACCTCCGGCACGTCGGGTACTTCCGGTACGTCTGGCA

CCTCCGGCACGTCTGGCACCTCCGGTACGTCCGGTACGTCCGGCACCTCCGGCACGTCTGGCACCTCCGGTACGTCCGG

CACGTCTGGCACGTCTGGCACGTCTGGCACGTCCGGCACGTCTGGCACGTCTGGCACGTCTGGCACGTCCGGTACGTCT

GGCACCTCCGGCACGTCGGGTACTTCCGGCACGTCGGGCACCTCCGGTACGTCGGGTACTTCCGGTACGTCCGGCACCT

CCGGCACCTCCGGCACCTCCGGCACCTCCGGTACGTCGGGTACCTCCGGCACGTCAGGCACTTCCGGTACGTCGGGCAC

GTCCGGCACCGCCTCCTCCCCGCTCGGCAACATCCTCGCGCAAACCGGCAACCTGATCACCGCGACGGGCACCGTCGTC

TCCGCCACCGGCAACCAAATCTCCGGCACGTCGGTGCCGGGCGTGAACGGCGCGACGACGACGAACCTCGGCAACGCCG

TCAGCTCGCTCGGCAACGGCGTCCAGACGCTCGGCAACGGCACAGCCGCCGGCCTCGGCACGCTCGGCACGTCGACGAA

TCCGCTCGGCCCGACGCTCACGTCGACCTCCGGCGTCGTCTCGAACGTCGGCAACGCGGTCTCGTCGCTCGGCGGCGTC

GTGACGAGCATCGGCACCGGCCCGCTCGCGCCCGTCACGTCGCCGCTCGGCGGCGCGGTCGGCACGCTCGGCAGCACCG

TCACGCAAGTCGGCTCCGGCCTGAACAACGTGCTCACGAGCGCGCCCGTCCAGCAGCTCGAAACCGGCGTCAGCTCGAT

CATCAATCCGATCTCGAACGTCGTTTCCGGCACGACGCAGACGATCGGCACCGCCACCGGGCTCGGCGCACCCGTCAAT

AATCTGCTGACCACCGTCGGCAATGGGCTGAACGCGGCCGGCGCGAAGGTATCGGGCTCGACGAACAACCAGGTGGTGC

AGGCGGTCGGCGGCGTCGTCAGCCAGCTCGGCAACACGGTCACGAGCGTCGGCGGTCTGCTGACGGGCGGCACCACCAA

CCCGCTCGCACCGATCACCGGCATCGCCGGTTCGCTGAGCGGCACGCTCGGCGCCAACGCGGGCCTGAACGCAGGCGCG

ACGCAACCCGGCACGACGACGAACCCGCTCGCCCCCGTCACCGGCCTGCTCGGCGGCCTGTCGGGTTCGGCGGGCGGCA

ACGCGGCGGCCGGCCTCACCGCCGTGCTCGCGCCCATCACGAATGTCGTCGGCTCGCTGACCGCCGGCGTGAACGGCAC

CGTCGCCGCCCCGTCGCCCGCCCCGACCACCGGCTCGACGACGACCGCGCCCGCCACGGGTGTGGTCGGCAGCCTGACG

GGCGGCACGACCGCGAGCGGCACGTCGACGACGAACCTGCTCGCGCCCGTGACCAACCTGATCGGCGGGCTGCTCGGCG

GCGTGTCGGGCAAGTAG

>Bucl3_Bm_PRL-20.seq Reverse Complement DNA Sequence Untitled Seq #55(1,1773)

ATGCAAAAACTGTTCAATAAATCGGCCATCGCAATCGGCATTTCCACCCTTCTCGTTCTGTCCGGCTGCGGCTCGGTCG

ACGGCCCGTCGCTGTCGGCCAGCGGCGTCAAGCCCGCCACCGTCGGCAATACGGGGACTTCGGGGACGTCCGGCACTTC

CGGTACGTCGGGTACTTCCGGCACGTCCGGCACCTCCGGTACGTCAGGTACTTCCGGCACGTCCGGTACGTCCGGCACC

TCCGGTACGTCTGGCACGTCCGGTACGTCTGGCACCTCCGGCACCTCCGGCACGTCGGGTACTTCCGGTACGTCTGGCA

CCTCCGGCACGTCTGGCACCTCCGGTACGTCCGGTACGTCCGGCACCTCCGGCACGTCTGGCACCTCCGGTACGTCCGG

CACGTCTGGCACGTCTGGCACGTCTGGCACGTCTGGCACGTCTGGCACGTCCGGCACGTCTGGCACGTCTGGCACGTCT

GGCACGTCTGGCACGTCCGGTACGTCTGGCACCTCCGGCACGTCGGGTACTTCCGGCACGTCGGGCACCTCCGGTACGT

CGGGTACTTCCGGTACGTCCGGCACCTCCGGCACCTCCGGCACCTCCGGTACGTCGGGTACCTCCGGCACGTCAGGCAC

TTCCGGTACGTCGGGCACGTCCGGCACCGCCTCCTCCCCGCTCGGCAACATCCTCGCGCAAACCGGCAACCTGATCACC

GCGACGGGCACCGTCGTCTCCGCCACCGGCAACCAAATCTCCGGCACGTCGGTGCCGGGCGTGAACGGCGCGACGACGA

CGAACCTCGGCAACGCCGTCAGCTCGCTCGGCAACGGCGTCCAGACGCTCGGCAACGGCACAGCCGCCGGCCTCGGCAC

GCTCGGCACGTCGACGAATCCGCTCGGCCCGACGCTCACGTCGACCTCCGGCGTCGTCTCGAACGTCGGCAACGCGGTC

TCGTCGCTCGGCGGCGTCGTGACGAGCATCGGCACCGGCCCGCTCGCGCCCGTCACGTCGCCGCTCGGCGGCGCGGTCG

GCACGCTCGGCAGCACCGTCACGCAAGTCGGCTCCGGCCTGAACAACGTGCTCACGAGCGCGCCCGTCCAGCAGCTCGA

AACCGGCGTCAGCTCGATCATCAATCCGATCTCGAACGTCGTTTCCGGCACGACGCAGACGATCGGCACCGCCACCGGG

CTCGGCGCACCCGTCAATAATCTGCTGACCACCGTCGGCAATGGGCTGAACGCGGCCGGCGCGAAGGTATCGGGCTCGA

CGAACAACCAGGTGGTGCAGGCGGTCGGCGGCGTCGTCAGCCAGCTCGGCAACACGGTCACGAGCGTCGGCGGTCTGCT

GACGGGCGGCACCACCAACCCGCTCGCACCGATCACCGGCATCGCCGGTTCGCTGAGCGGCACGCTCGGCGCCAACGCG

GGCCTGAACGCAGGCGCGACGCAACCCGGCACGACGACGAACCCGCTCGCCCCCGTCACCGGCCTGCTCGGCGGCCTGT

CGGGTTCGGCGGGCGGCAACGCGGCGGCCGGCCTCACCGCCGTGCTCGCGCCCATCACGAATGTCGTCGGCTCGCTGAC

CGCCGGCGTGAACGGCACCGTCGCCGCCCCGTCGCCCGCCCCGACCACCGGCTCGACGACGACCGCGCCCGCCACGGGT

GTGGTCGGCAGCCTGACGGGCGGCACGACCGCGAGCGGCACGTCGACGACGAACCTGCTCGCGCCCGTGACCAACCTGA

TCGGCGGGCTGCTCGGCGGCGTGTCGGGCAAGTAG

>Bucl3_Bm_strain_6.seq Created: Sunday, March 23, 2014 5:30 PM

ATGCAAAAACTGTTCAATAAATCGGCCATCGCAATCGGCATTTCCACCCTTCTCGTTCTGTCCGGCTGCGGCTCGGTCG

ACGGCCCGTCGCTGTCGGCCAGCGGCGTCAAGCCCGCCACCGTCGGCAATACGGGGACTTCGGGGACGTCCGGCACTTC

CGGTACGTCGGGTACTTCCGGCACGTCCGGCACCTCCGGTACGTCAGGTACTTCCGGCACGTCCGGTACGTCCGGCACC

TCCGGTACGTCTGGCACGTCCGGTACGTCTGGCACCTCCGGCACCTCCGGCACGTCGGGTACTTCCGGTACGTCTGGCA

CCTCCGGCACGTCTGGCACCTCCGGTACGTCCGGTACGTCCGGCACCTCCGGCACGTCTGGCACCTCCGGTACGTCCGG

CACGTCTGGCACGTCTGGCACGTCTGGCACGTCCGGTACGTCTGGCACCTCCGGCACGTCGGGTACTTCCGGCACGTCG

GGCACCTCCGGTACGTCGGGTACTTCCGGTACGTCCGGCACCTCCGGCACCTCCGGTACGTCGGGTACCTCCGGCACGT

CAGGCACTTCCGGTACGTCGGGCACGTCCGGCACCGCCTCCTCCCCGCTCGGCAACATCCTCGCGCAAACCGGCAACCT

GATCACCGCGACGGGCACCGTCGTCTCCGCCACCGGCAACCAAATCTCCGGCACGTCGGTGCCGGGCGTGAACGGCGCG

ACGACGACGAACCTCGGCAACGCCGTCAGCTCGCTCGGCAACGGCGTCCAGACGCTCGGCAACGGCACAGCCGCCGGCC

TCGGCACGCTCGGCACGTCGACGAATCCGCTCGGCCCGACGCTCACGTCGACCTCCGGCGTCGTCTCGAACGTCGGCAA

CGCGGTCTCGTCGCTCGGCGGCGTCGTGACGAGCATCGGCACCGGCCCGCTCGCGCCCGTCACGTCGCCGCTCGGCGGC

GCGGTCGGCACGCTCGGCAGCACCGTCACGCAAGTCGGCTCCGGCCTGAACAACGTGCTCACGAGCGCGCCCGTCCAGC

AGCTCGAAACCGGCGTCAGCTCGATCATCAATCCGATCTCGAACGTCGTTTCCGGCACGACGCAGACGATCGGCACCGC

CACCGGGCTCGGCGCACCCGTCAATAATCTGCTGACCACCGTCGGCAATGGGCTGAACGCGGCCGGCGCGAAGGTATCG

GGCTCGACGAACAACCAGGTGGTGCAGGCGGTCGGCGGCGTCGTCAGCCAGCTCGGCAACACGGTCACGAGCGTCGGCG

GTCTGCTGACGGGCGGCACCACCAACCCGCTCGCACCGATCACCGGCATCGCCGGTTCGCTGAGCGGCACGCTCGGCGC

CAACGCGGGCCTGAACGCAGGCGCGACGCAACCCGGCACGACGACGAACCCGCTCGCCCCCGTCACCGGCCTGCTCGGC

GGCCTGTCGGGTTCGGCGGGCGGCAACGCGGCGGCCGGCCTCACCGCCGTGCTCGCGCCCATCACGAATGTCGTCGGCT

CGCTGACCGCCGGCGTGAACGGCACCGTCGCCGCCCCGTCGCCCGCCCCGACCACCGGCTCGACGACGACCGCGCCCGC

CACGGGTGTGGTCGGCAGCCTGACGGGCGGCACGACCGCGAGCGGCACGTCGACGACGAACCTGCTCGCGCCCGTGACC

AACCTGATCGGCGGGCTGCTCGGCGGCGTGTCGGGCAAGTAG

>Bucl3_Bm_strain_11.seq Created: Sunday, March 23, 2014 5:34 PM

ATGCAAAAACTGTTCAATAAATCGGCCATCGCAATCGGCATTTCCACCCTTCTCGTTCTGTCCGGCTGCGGCTCGGTCG

ACGGCCCGTCGCTGTCGGCCAGCGGCGTCAAGCCCGCCACCGTCGGCAATACGGGGACTTCGGGGACGTCCGGCACTTC

CGGTACGTCGGGTACTTCCGGCACGTCCGGCACCTCCGGTACGTCAGGTACTTCCGGCACGTCCGGTACGTCCGGCACC

TCCGGTACGTCTGGCACGTCCGGTACGTCTGGCACCTCCGGCACCTCCGGCACGTCGGGTACTTCCGGTACGTCTGGCA

CCTCCGGCACGTCTGGCACCTCCGGTACGTCCGGTACGTCCGGCACCTCCGGCACGTCTGGCACCTCCGGTACGTCCGG

CACGTCTGGCACGTCTGGCACGTCCGGCACGTCTGGCACGTCTGGCACGTCTGGCACGTCCGGTACGTCTGGCACCTCC

GGCACGTCGGGTACTTCCGGCACGTCGGGCACCTCCGGTACGTCGGGTACTTCCGGTACGTCCGGCACCTCCGGCACCT

CCGGCACCTCCGGTACGTCGGGTACCTCCGGCACGTCAGGCACTTCCGGTACGTCGGGCACGTCCGGCACCGCCTCCTC

CCCGCTCGGCAACATCCTCGCGCAAACCGGCAACCTGATCACCGCGACGGGCACCGTCGTCTCCGCCACCGGCAACCAA

ATCTCCGGCACGTCGGTGCCGGGCGTGAACGGCGCGACGACGACGAACCTCGGCAACGCCGTCAGCTCGCTCGGCAACG

GCGTCCAGACGCTCGGCAACGGCACAGCCGCCGGCCTCGGCACGCTCGGCACGTCGACGAATCCGCTCGGCCCGACGCT

CACGTCGACCTCCGGCGTCGTCTCGAACGTCGGCAACGCGGTCTCGTCGCTCGGCGGCGTCGTGACGAGCATCGGCACC

GGCCCGCTCGCGCCCGTCACGTCGCCGCTCGGCGGCGCGGTCGGCACGCTCGGCAGCACCGTCACGCAAGTCGGCTCCG

GCCTGAACAACGTGCTCACGAGCGCGCCCGTCCAGCAGCTCGAAACCGGCGTCAGCTCGATCATCAATCCGATCTCGAA

CGTCGTTTCCGGCACGACGCAGACGATCGGCACCGCCACCGGGCTCGGCGCACCCGTCAATAATCTGCTGACCACCGTC

GGCAATGGGCTGAACGCGGCCGGCGCGAAGGTATCGGGCTCGACGAACAACCAGGTGGTGCAGGCGGTCGGCGGCGTCG

TCAGCCAGCTCGGCAACACGGTCACGAGCGTCGGCGGTCTGCTGACGGGCGGCACCACCAACCCGCTCGCACCGATCAC

CGGCATCGCCGGTTCGCTGAGCGGCACGCTCGGCGCCAACGCGGGCCTGAACGCAGGCGCGACGCAACCCGGCACGACG

ACGAACCCGCTCGCCCCCGTCACCGGCCTGCTCGGCGGCCTGTCGGGTTCGGCGGGCGGCAACGCGGCGGCCGGCCTCA

CCGCCGTGCTCGCGCCCATCACGAATGTCGTCGGCTCGCTGACCGCCGGCGTGAACGGCACCGTCGCCGCCCCGTCGCC

CGCCCCGACCACCGGCTCGACGACGACCGCGCCCGCCACGGGTGTGGTCGGCAGCCTGACGGGCGGCACGACCGCGAGC

GGCACGTCGACGACGAACCTGCTCGCGCCCGTGACCAACCTGATCGGCGGGCTGCTCGGCGGCGTGTCGGGCAAGTAG

>Bucl3_Bm_FMH.seq Reverse Complement DNA Sequence Untitled Seq #48(1,1755)

ATGCAAAAACTGTTCAATAAATCGGCCATCGCAATCGGCATTTCCACCCTTCTCGTTCTGTCCGGCTGCGGCTCGGTCG

ACGGCCCGTCGCTGTCGGCCAGCGGCGTCAAGCCCGCCACCGTCGGCAATACGGGGACTTCGGGGACGTCCGGCACTTC

CGGTACGTCGGGTACTTCCGGCACGTCCGGCACCTCCGGTACGTCAGGTACTTCCGGCACGTCCGGTACGTCCGGCACC

TCCGGTACGTCTGGCACGTCCGGTACGTCTGGCACCTCCGGCACCTCCGGCACGTCGGGTACTTCCGGTACGTCTGGCA

CCTCCGGCACGTCTGGCACCTCCGGTACGTCCGGTACGTCCGGCACCTCCGGCACGTCTGGCACCTCCGGTACGTCCGG

CACGTCTGGCACGTCTGGCACGTCTGGCACGTCCGGCACGTCTGGCACGTCTGGCACGTCTGGCACGTCCGGTACGTCT

GGCACCTCCGGCACGTCGGGTACTTCCGGCACGTCGGGCACCTCCGGTACGTCGGGTACTTCCGGTACGTCCGGCACCT

CCGGCACCTCCGGCACCTCCGGCACCTCCGGTACGTCGGGTACCTCCGGCACGTCAGGCACTTCCGGTACGTCGGGCAC

GTCCGGCACCGCCTCCTCCCCGCTCGGCAACATCCTCGCGCAAACCGGCAACCTGATCACCGCGACGGGCACCGTCGTC

TCCGCCACCGGCAACCAAATCTCCGGCACGTCGGTGCCGGGCGTGAACGGCGCGACGACGACGAACCTCGGCAACGCCG

TCAGCTCGCTCGGCAACGGCGTCCAGACGCTCGGCAACGGCACAGCCGCCGGCCTCGGCACGCTCGGCACGTCGACGAA

TCCGCTCGGCCCGACGCTCACGTCGACCTCCGGCGTCGTCTCGAACGTCGGCAACGCGGTCTCGTCGCTCGGCGGCGTC

GTGACGAGCATCGGCACCGGCCCGCTCGCGCCCGTCACGTCGCCGCTCGGCGGCGCGGTCGGCACGCTCGGCAGCACCG

TCACGCAAGTCGGCTCCGGCCTGAACAACGTGCTCACGAGCGCGCCCGTCCAGCAGCTCGAAACCGGCGTCAGCTCGAT

CATCAATCCGATCTCGAACGTCGTTTCCGGCACGACGCAGACGATCGGCACCGCCACCGGGCTCGGCGCACCCGTCAAT

AATCTGCTGACCACCGTCGGCAATGGGCTGAACGCGGCCGGCGCGAAGGTATCGGGCTCGACGAACAACCAGGTGGTGC

AGGCGGTCGGCGGCGTCGTCAGCCAGCTCGGCAACACGGTCACGAGCGTCGGCGGTCTGCTGACGGGCGGCACCACCAA

CCCGCTCGCACCGATCACCGGCATCGCCGGTTCGCTGAGCGGCACGCTCGGCGCCAACGCGGGCCTGAACGCAGGCGCG

ACGCAACCCGGCACGACGACGAACCCGCTCGCCCCCGTCACCGGCCTGCTCGGCGGCCTGTCGGGTTCGGCGGGCGGCA

ACGCGGCGGCCGGCCTCACCGCCGTGCTCGCGCCCATCACGAATGTCGTCGGCTCGCTGACCGCCGGCGTGAACGGCAC

CGTCGCCGCCCCGTCGCCCGCCCCGACCACCGGCTCGACGACGACCGCGCCCGCCACGGGTGTGGTCGGCAGCCTGACG

GGCGGCACGACCGCGAGCGGCACGTCGACGACGAACCTGCTCGCGCCCGTGACCAACCTGATCGGCGGGCTGCTCGGCG

GCGTGTCGGGCAAGTAG

>Bucl3_Bm_JHU.seq Reverse Complement DNA Sequence Untitled Seq #46(1,1754)

ATGCAAAAACTGTTCAATAAATCGGCCATCGCAATCGGCATTTCCACCCTTCTCGTTCTGTCCGGCTGCGGCTCGGTCG

ACGGCCCGTCGCTGTCGGCCAGCGGCGTCAAGCCCGCCACCGTCGGCAATACGGGGACTTCGGGGACGTCCGGCACTTC

CGGTACGTCGGGTACTTCCGGCACGTCCGGCACCTCCGGTACGTCAGGTACTTCCGGCACGTCCGGTACGTCCGGCACC

TCCGGTACGTCTGGCACGTCCGGTACGTCTGGCACCTCCGGCACCTCCGGCACGTCGGGTACTTCCGGTACGTCTGGCA

CCTCCGGCACGTCTGGCACCTCCGGTACGTCCGGTACGTCCGGCACCTCCGGCACGTCTGGCACCTCCGGTACGTCCGG

CACGTCTGGCACGTCTGGCACGTCTGGCACGTCCGGCACGTCTGGCACGTCTGGCACGTCTGGCACGTCCGGTACGTCT

GGCACCTCCGGCACGTCGGGTACTTCCGGCACGTCGGGCACCTCCGGTACGTCGGGTACTTCCGGTACGTCCGGCACCT

CCGGCACCTCCGGCACCTCCGGCACCTCCGGTACGTCGGGTACCTCCGGCACGTCAGGCACTTCCGGTACGTCGGGCAC

GTCCGGCACCGCCTCCTCCCCGCTCGGCAACATCCTCGCGCAAACCGGCAACCTGATCACCGCGACGGGCACCGTCGTC

TCCGCCACCGGCAACCAAATCTCCGGCACGTCGGTGCCGGGCGTGAACGGCGCGACGACGACGAACCTCGGCAACGCCG

TCAGCTCGCTCGGCAACGGCGTCCAGACGCTCGGCAACGGCACAGCCGCCGGCCTCGGCACGCTCGGCACGTCGACGAA

TCCGCTCGGCCCGACGCTCACGTCGACCTCCGGCGTCGTCTCGAACGTCGGCAACGCGGTCTCGTCGCTCGGCGGCGTC

GTGACGAGCATCGGCACCGGCCCGCTCGCGCCCGTCACGTCGCCGCTCGGCGGCGCGGTCGGCACGCTCGGCAGCACCG

TCACGCAAGTCGGCTCCGGCCTGAACAACGTGCTCACGAGCGCGCCCGTCCAGCAGCTCGAAACCGGCGTCAGCTCGAT

CATCAATCCGATCTCGAACGTCGTTTCCGGCACGACGCAGACGATCGGCACCGCCACCGGGCTCGGCGCACCCGTCAAT

AATCTGCTGACCACCGTCGGCAATGGGCTGAACGCGGCCGGCGCGAAGGTATCGGGCTCGACGAACAACCAGGTGGTGC

AGGCGGTCGGCGGCGTCGTCAGCCAGCTCGGCAACACGGTCACGAGCGTCGGCGGTCTGCTGACGGGCGGCACCACCAA

CCCGCTCGCACCGATCACCGGCATCGCCGGTTCGCTGAGCGGCACGCTCGGCGCCAACGCGGGCCTGAACGCAGGCGCG

ACGCAACCCGGCACGACGACGAACCCGCTCGCCCCCGTCACCGGCCTGCTCGGCGGCCTGTCGGGTTCGGCGGGCGGCA

ACGCGGCGGCCGGCCTCACCGCCGTGCTCGCGCCCATCACGAATGTCGTCGGCTCGCTGACCGCCGGCGTGAACGGCAC

CGTCGCCGCCCCGTCGCCCGCCCCGACCACCGGCTCGACGACGACCGCGCCCGCCACGGGTGTGGTCGGCAGCCTGACG

GGCGGCACGACCGCGAGCGGCACGTCGACGACGAACCTGCTCGCGCCCGTGACCAACCTGATCGGCGGGCTGCTCGGCG

GCGTGTCGGGCAAGTAG

>Bucl3_Bm_ATCC_10399.seq Reverse Complement DNA Sequence Untitled Seq #37(1,1746)

ATGCAAAAACTGTTCAATAAATCGGCCATCGCAATCGGCATTTCCACCCTTCTCGTTCTGTCCGGCTGCGGCTCGGTCG

ACGGCCCGTCGCTGTCGGCCAGCGGCGTCAAGCCCGCCACCGTCGGCAATACGGGGACTTCGGGGACGTCCGGCACTTC

CGGTACGTCGGGTACTTCCGGCACGTCCGGCACCTCCGGTACGTCAGGTACTTCCGGCACGTCCGGTACGTCCGGCACC

TCCGGTACGTCTGGCACGTCCGGTACGTCTGGCACCTCCGGCACCTCCGGCACGTCGGGTACTTCCGGTACGTCTGGCA

CCTCCGGCACGTCTGGCACCTCCGGTACGTCCGGTACGTCCGGCACCTCCGGCACGTCTGGCACCTCCGGTACGTCCGG

CACGTCTGGCACGTCTGGCACGTCTGGCACGTCCGGCACGTCTGGCACGTCTGGCACGTCTGGCACGTCCGGTACGTCT

GGCACCTCCGGCACGTCGGGTACTTCCGGCACGTCGGGCACCTCCGGTACGTCGGGTACTTCCGGTACGTCCGGCACCT

CCGGCACCTCCGGCACCTCCGGTACGTCGGGTACCTCCGGCACGTCAGGCACTTCCGGTACGTCGGGCACGTCCGGCAC

CGCCTCCTCCCCGCTCGGCAACATCCTCGCGCAAACCGGCAACCTGATCACCGCGACGGGCACCGTCGTCTCCGCCACC

GGCAACCAAATCTCCGGCACGTCGGTGCCGGGCGTGAACGGCGCGACGACGACGAACCTCGGCAACGCCGTCAGCTCGC

TCGGCAACGGCGTCCAGACGCTCGGCAACGGCACAGCCGCCGGCCTCGGCACGCTCGGCACGTCGACGAATCCGCTCGG

CCCGACGCTCACGTCGACCTCCGGCGTCGTCTCGAACGTCGGCAACGCGGTCTCGTCGCTCGGCGGCGTCGTGACGAGC

ATCGGCACCGGCCCGCTCGCGCCCGTCACGTCGCCGCTCGGCGGCGCGGTCGGCACGCTCGGCAGCACCGTCACGCAAG

TCGGCTCCGGCCTGAACAACGTGCTCACGAGCGCGCCCGTCCAGCAGCTCGAAACCGGCGTCAGCTCGATCATCAATCC

GATCTCGAACGTCGTTTCCGGCACGACGCAGACGATCGGCACCGCCACCGGGCTCGGCGCACCCGTCAATAATCTGCTG

ACCACCGTCGGCAATGGGCTGAACGCGGCCGGCGCGAAGGTATCGGGCTCGACGAACAACCAGGTGGTGCAGGCGGTCG

GCGGCGTCGTCAGCCAGCTCGGCAACACGGTCACGAGCGTCGGCGGTCTGCTGACGGGCGGCACCACCAACCCGCTCGC

ACCGATCACCGGCATCGCCGGTTCGCTGAGCGGCACGCTCGGCGCCAACGCGGGCCTGAACGCAGGCGCGACGCAACCC

GGCACGACGACGAACCCGCTCGCCCCCGTCACCGGCCTGCTCGGCGGCCTGTCGGGTTCGGCGGGCGGCAACGCGGCGG

CCGGCCTCACCGCCGTGCTCGCGCCCATCACGAATGTCGTCGGCTCGCTGACCGCCGGCGTGAACGGCACCGTCGCCGC

CCCGTCGCCCGCCCCGACCACCGGCTCGACGACGACCGCGCCCGCCACGGGTGTGGTCGGCAGCCTGACGGGCGGCACG

ACCGCGAGCGGCACGTCGACGACGAACCTGCTCGCGCCCGTGACCAACCTGATCGGCGGGCTGCTCGGCGGCGTGTCGG

GCAAGTAG

>Bucl3_Bm_GB8_horse_4.seq Reverse Complement DNA Sequence Untitled Seq #51(1,1755)

ATGCAAAAACTGTTCAATAAATCGGCCATCGCAATCGGCATTTCCACCCTTCTCGTTCTGTCCGGCTGCGGCTCGGTCG

ACGGCCCGTCGCTGTCGGCCAGCGGCGTCAAGCCCGCCACCGTCGGCAATACGGGGACTTCGGGGACGTCCGGCACTTC

CGGTACGTCGGGTACTTCCGGCACGTCCGGCACCTCCGGTACGTCAGGTACTTCCGGCACGTCCGGTACGTCCGGCACC

TCCGGTACGTCTGGCACGTCCGGTACGTCTGGCACCTCCGGCACCTCCGGCACGTCGGGTACTTCCGGTACGTCTGGCA

CCTCCGGCACGTCTGGCACCTCCGGTACGTCCGGTACGTCCGGCACCTCCGGCACGTCTGGCACCTCCGGTACGTCCGG

CACGTCTGGCACGTCTGGCACGTCTGGCACGTCCGGCACGTCTGGCACGTCTGGCACGTCTGGCACGTCCGGTACGTCT

GGCACCTCCGGCACGTCGGGTACTTCCGGCACGTCGGGCACCTCCGGTACGTCGGGTACTTCCGGTACGTCCGGCACCT

CCGGCACCTCCGGCACCTCCGGCACCTCCGGTACGTCGGGTACCTCCGGCACGTCAGGCACTTCCGGTACGTCGGGCAC

GTCCGGCACCGCCTCCTCCCCGCTCGGCAACATCCTCGCGCAAACCGGCAACCTGATCACCGCGACGGGCACCGTCGTC

TCCGCCACCGGCAACCAAATCTCCGGCACGTCGGTGCCGGGCGTGAACGGCGCGACGACGACGAACCTCGGCAACGCCG

TCAGCTCGCTCGGCAACGGCGTCCAGACGCTCGGCAACGGCACAGCCGCCGGCCTCGGCACGCTCGGCACGTCGACGAA

TCCGCTCGGCCCGACGCTCACGTCGACCTCCGGCGTCGTCTCGAACGTCGGCAACGCGGTCTCGTCGCTCGGCGGCGTC

GTGACGAGCATCGGCACCGGCCCGCTCGCGCCCGTCACGTCGCCGCTCGGCGGCGCGGTCGGCACGCTCGGCAGCACCG

TCACGCAAGTCGGCTCCGGCCTGAACAACGTGCTCACGAGCGCGCCCGTCCAGCAGCTCGAAACCGGCGTCAGCTCGAT

CATCAATCCGATCTCGAACGTCGTTTCCGGCACGACGCAGACGATCGGCACCGCCACCGGGCTCGGCGCACCCGTCAAT

AATCTGCTGACCACCGTCGGCAATGGGCTGAACGCGGCCGGCGCGAAGGTATCGGGCTCGACGAACAACCAGGTGGTGC

AGGCGGTCGGCGGCGTCGTCAGCCAGCTCGGCAACACGGTCACGAGCGTCGGCGGTCTGCTGACGGGCGGCACCACCAA

CCCGCTCGCACCGATCACCGGCATCGCCGGTTCGCTGAGCGGCACGCTCGGCGCCAACGCGGGCCTGAACGCAGGCGCG

ACGCAACCCGGCACGACGACGAACCCGCTCGCCCCCGTCACCGGCCTGCTCGGCGGCCTGTCGGGTTCGGCGGGCGGCA

ACGCGGCGGCCGGCCTCACCGCCGTGCTCGCGCCCATCACGAATGTCGTCGGCTCGCTGACCGCCGGCGTGAACGGCAC

CGTCGCCGCCCCGTCGCCCGCCCCGACCACCGGCTCGACGACGACCGCGCCCGCCACGGGTGTGGTCGGCAGCCTGACG

GGCGGCACGACCGCGAGCGGCACGTCGACGACGAACCTGCTCGCGCCCGTGACCAACCTGATCGGCGGGCTGCTCGGCG

GCGTGTCGGGCAAGTAG

>Bucl3_Bt_2002721723.seq Reverse Complement DNA Sequence Untitled Seq #30(1,1806)

ATGCAAAAACTGTTCAATAAATCGGCCATCGCAATCGGCATTTCCACCCTTCTCGCTCTGTCCGGCTGCGGCTCGGTCG

ACGGCCCGTCGCTGTCGGCCGGCGGCGTCAAGCCCGCCACCGTCGGCAATTCGGGGACTTCGGGGACCTCCGGTACATC

GGGCACTTCCGGCACGTCGGGTTCGGGCGGCACGTCCGGATCGTCGGGTTCGGGGACTTCGGGCACGTCGGGGACTTCG

GGCACCTCGGGGACCTCCGGTACGTCGGGCACTTCCGGTACGTCGGGCACTTCCGGCACCTCGGGGACTTCCGGTACCT

CGGGCACCTCCGGCACCTCGGGGACTTCCGGCACCTCGGGCACTTCCGGTACGTCGGGCACTTCCGGCACCTCGGGGAC

TTCCGGCACCTCGGGGACTTCCGGTACGTCGGGCACCTCCGGTACGTCGGGCACCTCCGGTACGTCGGGCACCTCCGGC

ACGTCGGGGACCTCCGGTACGTCGGGCACCTCCGGTACGTCGGGCACCTCCGGTACGTCGGGCACCTCCGGTACGTCGG

GCACCTCCGGTACGTCGGGCACCTCCGGTACGTCGGGCACCTCCGGTACGTCGGGCACCTCCGGTACGTCGGGCACCTC

CGGCACCGCCTCCTCCCCGCTCGGCAATATCCTCGCGCAGACCGGCAACCTGATCACCGCGACGGGCACCGTCGTCTCC

GCCACCGGCAACCAGATCTCTGGGACGTCGGTGCCGGGCGTCAACAGCGCGACGACCACGAACCTCGGCAACGCCGTCA

GCTCGCTCGGCAACGGCGTCCAGACGCTCGGCAACGGCACCGCCGCCGGCCTCGGCACGCTCGGCACGTCGACGAACCC

GCTCGGGCCGACGCTCACGTCGACATCGGGCGTCGTATCGAACGTCGGCAATGCGGTTTCGTCGCTCGGCGGCGTCGTG

ACGAGCATCGGCACCGGCCCGCTCGCGCCGCTCGCCCCCGTCACGTCGCCGCTCGGCGGCGCGGTCGGCACGCTCGGCA

GCACCGTCACGCAGGTCGGCTCAGGCCTGAACAACGTGCTCACGAGCGCGCCCATCCAGCAGCTCGAGACGGGCGTCAG

CTCGATCATCAATCCGATCACGAACGCCGTCTCCGGCACGACGCAGACGATCGGCACCGCCACCGGCCTCGGCGCGCCG

GTCAACAACCTGCTGACCACCGTCGGCAACGGGCTGAACGCGGCCGGCACGAAGGTGTCGGGCTCGACGAACAACCAGG

TGGTCCAGGCGGTCGGCGGCGTCGTCAGCCAGCTCGGCAACACGGTCACGAGCGTCGGCGGCCTGCTGACGGGCGGCAC

GACCAATCCGCTTGCGCCGATCACCGGCGTGCTGTCCGGCGGCTCGACCAACCCGCTCGCGCCGATCACCGGCGTCGCG

GGCTCGCTGAGCGGCACGCTCGGCGCCAACGCGGGCCTCACGGCGGGCACGACGCAGCCGGGCGCGACGACGAATCCGC

TCGCGCCCGTCACCGGCCTGCTCGGCGGCCTGTCGGGTTCGGCGGGCGGCAACGCGACGGCGGGCCTGACCGCCGCGCT

CGCGCCCGTCACGAATCTCGTCGGCTCGCTGACCGCCGGCATGACCGGCGCCGTCGCGGCAACGCCGCCCGCCACGACC

GCCGGCTCGACGACGACCGCGCCCGCCACGGGCGTCGTCGGCAGCCTGACCGGCGGCGCGACCGCGAGCGGCACGTCGA

CGACGAACCTGCTCGCGCCCGTGACCAACCTGATCGGCGGGCTGCTCGGCAGCGTGTCGGGCAAGTAG

>Bucl3_Bt_E444.seq Reverse Complement DNA Sequence Untitled Seq #27(1,1806)

ATGCAAAAACTGTTCAATAAATCGGCCATCGCAATCGGCATTTCCACCCTTCTCGCTCTGTCCGGCTGCGGCTCGGTCG

ACGGCCCGTCGCTGTCGGCCGGCGGCGTCAAGCCCGCCACCGTCGGCAATTCGGGGACTTCGGGGACCTCCGGTACATC

GGGCACTTCCGGCACGTCGGGTTCGGGCGGCACGTCCGGATCGTCGGGTTCGGGGACTTCGGGCACGTCGGGGACTTCG

GGCACCTCGGGGACCTCCGGTACGTCGGGCACTTCCGGTACGTCGGGCACTTCCGGCACCTCGGGGACTTCCGGTACCT

CGGGCACCTCCGGCACCTCGGGGACTTCCGGCACCTCGGGCACTTCCGGTACGTCGGGCACTTCCGGCACCTCGGGGAC

TTCCGGCACCTCGGGGACTTCCGGTACGTCGGGCACCTCCGGTACGTCGGGCACCTCCGGTACGTCGGGCACCTCCGGC

ACGTCGGGGACCTCCGGTACGTCGGGCACCTCCGGTACGTCGGGCACCTCCGGTACGTCGGGGACCTCCGGTACGTCGG

GCACCTCCGGTACGTCGGGCACCTCCGGTACGTCGGGCACCTCCGGTACGTCGGGCACCTCCGGTACGTCGGGCACCTC

CGGCACCGCCTCCTCCCCGCTCGGCAATATCCTCGCGCAGACCGGCAACCTGATCACCGCGACGGGCACCGTCGTCTCC

GCCACCGGCAACCAGATCTCTGGGACGTCGGTGCCGGGCGTCAACAGCGCGACGACCACGAACCTCGGCAACGCCGTCA

GCTCGCTCGGCAACGGCGTCCAGACGCTCGGCAACGGCACCGCCGCCGGCCTCGGCACGCTCGGCACGTCGACGAACCC

GCTCGGGCCGACGCTCACGTCGACATCGGGCGTCGTATCGAACGTCGGCAATGCGGTTTCGTCGCTCGGCGGCGTCGTG

ACGAGCATCGGCACCGGCCCGCTCGCGCCGCTCGCCCCCGTCACGTCGCCGCTCGGCGGCGCGGTCGGCACGCTCGGCA

GCACCGTCACGCAGGTCGGCTCAGGCCTGAACAACGTGCTCACGAGCGCGCCCATCCAGCAGCTCGAGACGGGCGTCAG

CTCGATCATCAATCCGATCACGAACGCCGTCTCCGGCACGACGCAGACGATCGGCACCGCCACCGGCCTCGGCGCGCCG

GTCAACAACCTGCTGACCACCGTCGGCAACGGGCTGAACGCGGCCGGCACGAAGGTGTCGGGCTCGACGAACAACCAGG

TGGTCCAGGCGGTCGGCGGCGTCGTCAGCCAGCTCGGCAACACGGTCACGAGCGTCGGCGGCCTGCTGACGGGCGGCAC

GACCAATCCGCTTGCGCCGATCACCGGCGTGCTGTCCGGCGGCTCGACCAACCCGCTCGCGCCGATCACCGGCGTCGCG

GGCTCGCTGAGCGGCACGCTCGGCGCCAACGCGGGCCTCACGGCGGGCACGACGCAGCCGGGCGCGACGACGAATCCGC

TCGCGCCCGTCACCGGCCTGCTCGGCGGCCTGTCGGGTTCGGCGGGCGGCAACGCGACGGCGGGCCTGACCGCCGCGCT

CGCGCCCGTCACGAATCTCGTCGGCTCGCTGACCGCCGGCATGACCGGCGCCGTCGCGGCAACGCCGCCCGCCACGACC

GCCGGCTCGACGACGACCGCGCCCGCCACGGGCGTCGTCGGCAGCCTGACCGGCGGCGCGACCGCGAGCGGCACGTCGA

CGACGAACCTGCTCGCGCCCGTGACCAACCTGATCGGCGGGCTGCTCGGCAGCGTGTCGGGCAAGTAG

>Bucl3_Bt_E555.seq Reverse Complement DNA Sequence Untitled Seq #11(1,1770)

ATGCAAAAACTGTTCAATAAATCGGCCATCGCAATCGGCATTTCCACCCTTCTCGCTCTGTCCGGCTGCGGCTCGGTCG

ACGGCCCGTCGCTGTCGGCCGGCGGCGTCAAGCCCGCCACCGTCGGCAATTCGGGGACTTCGGGGACCTCCGGTACATC

GGGCACTTCCGGCACGTCGGGTTCGGGCGGCACGTCCGGATCGTCCGGATCGTCCGGATCGTCCGGATCGTCCGGATCG

TCGGGTTCGGGGACGTCGGGGACTTCGGGGACTTCGGGGACTTCGGGGACCTCGGGGACCTCGGGGACCTCGGGTACGT

CGGGCACTTCCGGTACGTCGGGGACTTCCGGCACCTCGGGGACTTCCGGTACCTCGGGCACCTCCGGCACGTCGGGTAC

TTCCGGCACCTCGGGGACTTCCGGCACCTCGGGTACCTCCGGTACGTCGGGGACTTCCGGCACCTCGGGGACTTCCGGT

ACCTCGGGCACCTCCGGCACCTCGGGGACTTCCGGTACGTCGGGCACCTCCGGTACGTCGGGTACATCCGGCACGTCGG

GGACCTCCGGTACGTCGGGCACCTCCGGTACGTCGGGCACCTCCGGCACCGCCTCCTCCCCGCTCGGCAATATCCTCGC

GCAGACCGGCAACCTGATCACCGCGACGGGCACCGTCGTCTCCGCCACCGGCAACCAGATCTCCGGGACGTCGGTGCCG

GGCGTCAACAGCGCGACGACCACGAACCTCGGCAACGCCGTCAGCTCGCTCGGCAACGGCGTCCAGACGCTCGGCAACG

GCACCGCCGCCGGCCTCGGCACGCTCGGCACGTCGACGAACCCGCTCGGGCCGACGCTCACGTCGACATCGGGCGTCGT

ATCGAACGTCGGCAATGCGGTTTCGTCGCTCGGCGGCGTCGTGACGAGCATCGGCACCGGCCCGCTCGCGCCGCTCGCC

CCCGTCACGTCGCCGCTCGGCGGCGCGGTCGGCACGCTCGGCAGCACCGTCACGCAGGTCGGCTCAGGCCTGAACAACG

TGCTCACGAGCGCGCCCATCCAGCAGCTCGAGACGGGCGTCAGCTCGATCATCAATCCGATCACGAACGCCGTCTCCGG

CACGACGCAGACGATCGGCACCGCCACCGGCCTCGGCGCGCCGGTCAACAACCTGCTGACCACCGTCGGCAACGGGCTG

AACGCGGCCGGCACGAAGGTGTCGGGCTCGACGAACAACCAGGTGGTCCAGGCGGTCGGCGGCGTCGTCAGCCAGCTCG

GCAACACGGTCACGAGCGTCGGCGGCCTGCTGACGGGCGGCACGACCAATCCGCTTGCGCCGATCACCGGCGTGCTGTC

CGGCGGCTCGACCAACCCGCTCGCCCCGATCACCGGCGTCGCGGGCTCGCTGAGCGGCACGCTCGGCGCCAACGCGGGC

CTCACGGCGGGCACGACGCAGCCGGGCACGACGACGAATCCGCTCGCGCCCGTCACCGGCCTGCTCGGCGGCCTGTCGG

GTTCGGCGGGCGGCAACGCGACGGCGGGCCTGACCGCCGCGCTCGCGCCCGTCACGAATCTCGTCGGCTCGCTGACCGC

CGGCATGACCGGCGCCGTCGCGGCAACGCCGCCCGCCACGACCGCCGGCTCGACGACGACCGCGCCCGCCACGGGCGTC

GTCGGCAGCCTGACCGGCGGCGCGACCGCGAGCGGCACGTCGACGACGAACCTGCTCGCGCCCGTGACCAACCTGATCG

GCGGGCTGCTCGGCAGCGTGTCGGGCAAGTAG

>Bucl3_Bt_H0587.seq Reverse Complement DNA Sequence Untitled Seq #33(1,1833)

ATGCAAAAACTGTTCAATAAATCGGCCATCGCAATCGGCATTTCCACCCTTCTCGCTCTGTCCGGCTGCGGCTCGGTCG

ACGGCCCGTCGCTGTCGGCCGGCGGCGTCAAGCCCGCCACCGTCGGCAATTCGGGGACTTCGGGGACCTCCGGTACATC

GGGCACTTCCGGCACGTCGGGTTCGGGCGGCACGTCCGGATCGTCCGGATCGTCCGGATCGTCGGGTTCGGGGACGTCG

GGGACGTCGGGGACTTCGGGGACCTCGGGGACCTCGGGGACCTCGGGCACTTCCGGTACGTCGGGCACTTCCGGTACGT

CGGGGACTTCCGGCACCTCGGGGACTTCCGGTACCTCGGGCACCTCCGGCACGTCGGGTACTTCCGGCACCTCGGGGAC

TTCCGGCACCTCGGGTACCTCCGGTACGTCGGGGACTTCCGGTACCTCGGGCACCTCCGGCACCTCGGGGACTTCCGGT

ACGTCGGGCACCTCCGGTACGTCGGGTACATCCGGCACGTCGGGGACCTCCGGTACGTCGGGCACCTCCGGCACGTCGG

GGACCTCCGGTACGTCGGGCACCTCCGGTACGTCGGGGACGTCCGGTACGTCGGGCACCTCCGGCACCGCCTCCTCCCC

GCTCGGCAATATCCTCGCGCAGACCGGCAACCTGATCACCGCGACGGGCACCGTCGTCTCCGCCACCGGCAACCAGATC

TCCGGGACGTCGGTGCCGGGCGTCAACAGCGCGACGACCACGAACCTCGGCAACGCCGTCAGCTCGCTCGGCAACGGCG

TCCAGACGCTCGGCAACGGCACCGCCGCCGGCCTCGGCACGCTCGGCACGTCGACGAACCCGCTCGGGCCAACGCTCAC

GTCGACATCGGGCGTCGTATCGAACGTCGGCAATGCGGTTTCGTCGCTCGGCGGCGTCGTGACGAGCATCGGCACCGGC

CCGCTCGCGCCGCTCGCCCCCGTCACGTCGCCACTCGGCGGCGCGGTCGGCACGCTCGGCAGCACCGTCACGCAGGTCG

GCTCAGGCCTGAACAACGTGCTCACGAGCGCGCCCATCCAGCAGCTCGAGACGGGCGTCAGCTCGATCATCAATCCGAT

CACGAACGCCGTCTCCGGCACGACGCAGACGATCGGCACCGCCACCGGCCTCGGCGCGCCGGTCAACAACCTGCTGACC

ACCGTCGGCAACGGGCTGAACGCGGCCGGCACGAAGGTGTCGGGCTCGACGAACAACCAGGTGGTCCAGGCGGTCGGCG

GCGTCGTCAGCCAGCTCGGCAACACGGTCACGAGCGTCGGCGGCCTGCTGACGGGCGGCACGACCAATCCGCTTGCGCC

GATCACCGGCGTGCTGTCCGGCGGCTCGACCAACCCGCTCGCCCCGATCACCGGCGTGCTGTCCGGCGGCTCGACCAAC

CCGCTCGCCCCGATCACCGGCGTCGCGGGCTCGCTGAGCGGCACGCTCGGCGCCAACGCGGGCCTCACGGCGGGCACGA

CGCAGCCGGGCACGACGACGAATCCGCTCGCGCCCGTCACCGGCCTGCTCGGCGGCCTGTCGGGTTCGGCGGGCGGCAA

CGCGACGGCGGGCCTGACCGCCGCGCTCGCGCCCGTCACGAATCTCGTCGGCTCGCTGACCGCCGGCATGACCGGCGCC

GTCGCGGCAACGCCGCCCGCCACGACCGCCGGCTCGACGACGACCGCGCCCGCCACGGGCGTCGTCGGCAGCCTGACCG

GCGGCGCGACCGCGAGCGGCACGTCGACGACGAACCTGCTCGCGCCCGTGACCAACCTGATCGGCGGGCTGCTCGGCAG

CGTGTCGGGCAAGTAG

>Bucl3_Bt_MSMB43.seq Created: Monday, March 24, 2014 11:42 AM

ATGCAAAAACTGTTCAATCAATCTGCCATCGCAATCGGCGTTTCCGCACTCCTCGTCCTGTCGGGCTGCGGCTCGGTCG

ACGGCCCGTCGTCGCCGTCCAGCGGCGTCAAGCCCACTACCGTCGGCAATACGGGAACGTCGGGCACGTCCGGCACCTC

GGGTTCGGGCGGCACGTCCGGATCGTCGGGTTCGGGCACTTCCGGGACTTCCGGGACTTCCGGGACTTCGGGCACTTCG

GGCACTTCGGGTACTTCGGGTACTTCGGGTACTTCGGGTACTTCGGGTACTTCGGGTACTTCGGGTACTTCGGGTACTT

CGGGTACTTCGGGTACTTCGGGTACTTCGGGCACTTCCGGCACTTCCGGCACTTCGGGCACTTCGGGCACTTCGGGTAC

TTCGGGCACTTCCGGCACTTCCGGCACTTCGGGCACTTCGGGCACTTCGGGCACTTCGGGCACTTCCGGCACTTCCGGC

ACCTCCGGCACCGCCTCCTCCCCGCTCGGCAACATCCTCTCGCAGACCGGCAACCTGATCACCGCGACGGGCACCGTCG

TCTCCGCCACCGGCAACCAGATCTCCGGCACGTCGGTGCCGGGCGTCAACGGCGCGACGACCACGAACCTCGGCAACGC

CGTCAGCTCGCTCGGCAACGGCGTCCAGACGCTCGGCAACGGCACCGTCGCCGGCCTCGGCACGCTCGGCACGTCGACG

AACCCGCTCGGCCCGACGCTCACGTCGACGTCCGGCGTCGTATCGAACGTCGGCAACGCGGTCTCGTCGCTCGGCCGCG

TCGTGACAAGCATCGGCACCGGCCCCCTCGCGCCGCTCGCGCCCGTCACGTCGCCGCTCGGCGGCGTGGTCGGCACGCT

CGGCAGCACCGTCACGCAGGTCGGCTCCGGCCTGAACAACGTGCTCACGAGCGCGCCCGTCCAGCAGCTCGAAACCGGC

GTCAGCTCGATCATCAACCCGATCACGAACGCCGTATCCGGCACGACGCAGACGATCGGCACCGCGACCGGACTCGGCG

CGCCCGTCAACAATCTGCTGGCCACCGTCGGCAACGGGCTGAACGCGGCCGGCGCGAAGGTGTCGGGCTCGACGAACAA

CCAGGTGGTCCACGCGGTCGGCGGCGTCGTCAGCCAGCTCGGCAACACGGTCACGAGCGTCGGCGGCCTGCTGACGGGC

GGCACGACCAATCCGCTCGCGCCGATCACCGGCGTGCTGTCCGGCGGCTCGACCAACCCGCTCGCGCCGATCACGGGCG

CCGTCGGCTCGCTTAGCGCCACGCTCGGCGTCAACGCGGGCCTCACGGCGGGCGCGACGCAGCCGGCGGGCACGGCGAC

GAATCCGCTCGCGCCCGTCACCGGCCTGCTCGGCGGCCTGTCGGGTTCGGCGGGCGGCAATGCGACGGCCGGCCTGAGC

GCCGTGCTCGCGCCCGTCACGAACCTCGTCGGCTCGCTGAGCGCCGGCGTGCACGGCACCGTCGCGGCCACGCCGCCCG

CGACGGCCACCGGTTCGACGACGACCGCGCCCGTCACGGGCGTCGTCGGCAGCCTGACCGGCAGCACGACCGCGAGCGG

CTCGTCGACGACGAACCTGCTCGCGCCCGTGACCAACCTGCTCGGCGGGCTGCTCGGCGGCGTGTCGGGCAAGTAG

>Bucl3_Bt_TXDOH.seq Reverse Complement DNA Sequence Untitled Seq #19(1,1923)

ATGCAAAAACTGTTCAATAAATCGGCCATCGCAATCGGCATTTCCACCCTTCTCGCTCTGTCCGGCTGCGGCTCGGTCG

ACGGCCCGTCGCTGTCGGCCGGCGGCGTCAAGCCCGCCACCGTCGGCAATTCGGGGACTTCGGGGACCTCCGGTACATC

GGGCACTTCCGGCACGTCGGGTTCGGGCGGCACGTCCGGATCGTCCGGATCGTCCGGATCGTCCGGATCGTCCGGATCG

TCCGGATCGTCCGGATCGTCGGGTTCGGGGACGTCGGGGACGTCGGGGACGTCGGGGACGTCGGGGACGTCGGGGACGT

CGGGGACTTCGGGGACTTCGGGGACTTCGGGGACTTCGGGGACCTCGGGGACCTCGGGGACCTCGGGTACGTCGGGCAC

TTCCGGTACGTCGGGGACTTCCGGCACCTCGGGGACTTCCGGTACCTCGGGCACCTCCGGCACGTCGGGTACTTCCGGC

ACCTCGGGGACTTCCGGCACCTCGGGTACCTCCGGTACGTCGGGGACTTCCGGCACCTCGGGGACTTCCGGTACCTCGG

GCACCTCCGGCACCTCGGGGACTTCCGGTACGTCGGGCACCTCCGGTACGTCGGGTACATCCGGCACGTCGGGGACCTC

CGGTACGTCGGGGACCTCCGGTACGTCGGGCACCTCCGGTACGTCGGGCACCTCCGGTACGTCGGGCACCTCCGGCACC

GCCTCCTCCCCGCTCGGCAATATCCTCGCGCAGACCGGCAACCTGATCACCGCGACGGGCACTGTCGTCTCCGCCACCG

GCAACCAGATCTCCGGGACGTCGGTGCCGGGCGTCAACAGCGCGACGACCACGAACCTCGGCAACGCCGTCAGCTCGCT

CGGCAACGGCGTCCAGACGCTCGGCAACGGCACCGCCGCCGGCCTCGGCACGCTCGGCACGTCGACGAACCCGCTCGGG

CCAACGCTCACGTCGACATCGGGCGTCGTATCGAACGTCGGCAATGCGGTTTCGTCGCTCGGCGGCGTCGTGACGAGCA

TCGGCACCGGCCCGCTCGCGCCGCTCGCCCCCGTCACGTCGCCGCTCGGCGGCGCGGTCGGCACGCTCGGCAGCACCGT

CACGCAGGTCGGCTCAGGCCTGAACAACGTGCTCACGAGCGCGCCCATCCAGCAGCTCGAGACGGGCGTCAGCTCGATC

ATCAATCCGATCACGAACGCCGTCTCCGGCACGACGCAGACGATCGGCACCGCCACCGGCCTCGGCGCGCCGGTCAACA

ACCTGCTGACCACCGTCGGCAACGGGCTGAACGCGGCCGGCACGAAGGTGTCGGGCTCGACGAACAACCAGGTGGTCCA

GGCGGTCGGCGGCGTCGTCAGCCAGCTCGGCAACACGGTCACGAGCGTCGGCGGCCTGCTGACGGGCGGCACGACCAAT

CCGCTTGCGCCGATCACCGGCGTGCTGTCCGGCGGCTCGACCAACCCGCTCGCCCCGATCACCGGCGTGCTGTCCGGCG

GCTCGACCAACCCGCTCGCCCCGATCACCGGCGTCGCGGGCTCGCTGAGCGGCACGCTCGGCGCCAACGCGGGCCTCAC

GGCGGGCACGACGCAGCCGGGCACGACGACGAATCCGCTCGCGCCCGTCACCGGCCTGCTCGGCGGCCTGTCGGGTTCG

GCGGGCGGCAACGCGACGGCGGGCCTGACCGCCGCGCTCGCGCCCGTCACGAATCTCGTCGGCTCGCTGACCGCCGGCA

TGACCGGCGCCGTCGCGGCAACGCCGCCCGCCACGACCGCCGGCTCGACGACGACCGCGCCCGCCACGGGCGTCGTCGG

CAGCCTGACCGGCGGCGCGACCGCGAGCGGCACGTCGACGACGAACCTGCTCGCGCCCGTGACCAACCTGATCGGCGGG

CTGCTCGGCAGCGTGTCGGGCAAGTAG

>Bucl3_Bt_4.seq Reverse Complement DNA Sequence Untitled Seq #14(1,1824)

ATGCAAAAACTGTTCAATAAATCGGCCATCGCAATCGGCATTTCCACCCTTCTCGCTCTGTCCGGCTGCGGCTCGGTCG

ACGGCCCGTCGCTGTCGGCCGGCGGCGTCAAGCCCGCCACCGTCGGCAATTCGGGGACTTCGGGGACCTCCGGTACATC

GGGCACTTCCGGCACGTCGGGTTCGGGCGGCACGTCCGGATCGTCGGGTTCGGGGACTTCGGGCACGTCGGGGACTTCG

GGCACCTCGGGGACCTCCGGTACGTCGGGCACTTCCGGTACGTCGGGCACTTCCGGCACCTCGGGGACTTCCGGTACCT

CGGGCACCTCCGGCACCTCGGGGACTTCCGGCACCTCGGGCACTTCCGGTACGTCGGGCACTTCCGGCACCTCGGGGAC

TTCCGGCACCTCGGGGACTTCCGGTACGTCGGGCACCTCCGGTACGTCGGGCACCTCCGGTACGTCGGGCACCTCCGGC

ACGTCGGGGACCTCCGGTACGTCGGGCACCTCCGGTACGTCGGGCACCTCCGGTACGTCGGGGACCTCCGGTACGTCGG

GCACCTCCGGTACGTCGGGCACCTCCGGTACGTCGGGCACCTCCGGTACGTCGGGCACCTCCGGTACGTCGGGCACCTC

CGGTACGTCGGGCACCTCCGGCACCGCCTCCTCCCCGCTCGGCAATATCCTCGCGCAGACCGGCAACCTGATCACCGCG

ACGGGCACCGTCGTCTCCGCCACCGGCAACCAGATCTCTGGGACGTCGGTGCCGGGCGTCAACAGCGCGACGACCACGA

ACCTCGGCAACGCCGTCAGCTCGCTCGGCAACGGCGTCCAGACGCTCGGCAACGGCACCGCCGCCGGCCTCGGCACGCT

CGGCACGTCGACGAACCCGCTCGGGCCGACGCTCACGTCGACATCGGGCGTCGTATCGAACGTCGGCAATGCGGTTTCG

TCGCTCGGCGGCGTCGTGACGAGCATCGGCACCGGCCCGCTCGCGCCGCTCGCCCCCGTCACGTCGCCGCTCGGCGGCG

CGGTCGGCACGCTCGGCAGCACCGTCACGCAGGTCGGCTCAGGCCTGAACAACGTGCTCACGAGCGCGCCCATCCAGCA

GCTCGAGACGGGCGTCAGCTCGATCATCAATCCGATCACGAACGCCGTCTCCGGCACGACGCAGACGATCGGCACCGCC

ACCGGCCTCGGCGCGCCGGTCAACAACCTGCTGACCACCGTCGGCAACGGGCTGAACGCGGCCGGCACGAAGGTGTCGG

GCTCGACGAACAACCAGGTGGTCCAGGCGGTCGGCGGCGTCGTCAGCCAGCTCGGCAACACGGTCACGAGCGTCGGCGG

CCTGCTGACGGGCGGCACGACCAATCCGCTTGCGCCGATCACCGGCGTGCTGTCCGGCGGCTCGACCAACCCGCTCGCG

CCGATCACCGGCGTCGCGGGCTCGCTGAGCGGCACGCTCGGCGCCAACGCGGGCCTCACGGCGGGCACGACGCAGCCGG

GCGCGACGACGAATCCGCTCGCGCCCGTCACCGGCCTGCTCGGCGGCCTGTCGGGTTCGGCGGGCGGCAACGCGACGGC

GGGCCTGACCGCCGCGCTCGCGCCCGTCACGAATCTCGTCGGCTCGCTGACCGCCGGCATGACCGGCGCCGTCGCGGCA

ACGCCGCCCGCCACGACCGCCGGCTCGACGACGACCGCGCCCGCCACGGGCGTCGTCGGCAGCCTGACCGGCGGCGCGA

CCGCGAGCGGCACGTCGACGACGAACCTGCTCGCGCCCGTGACCAACCTGATCGGCGGGCTGCTCGGCAGCGTGTCGGG

CAAGTAG

>Q63JT5_BURPS_Bps_K96243.seq Created: Tuesday, February 12, 2013 4:55 PM

ATGACGACCTACGACATCTTTGCGCTGCAGCACCTGGGCGACGAATTCATCCGCTTCATCGTGCTGCTCGCGCTGTCGA

GCGAGCGGCTGCTCGTGCTGATGGCGATCCTGCCGGCGACCGCCGACAACGTGATGAAAGGCCCGATGCGCAGCGGCGC

GGCGGCGGTGTGGTGCCTGTTCGTCGCGGGCGGACAGCAGGCGCTGATTCCGCAGCTCCACGGCGCGTTCCTGATGATC

GCCTGCGTGAAGGAGGCCGTGATCGGGCTCGTGCTCGGCATCGCGGCATCGACGGTATTCTGGGCCGCCGAGGCGATCG

GCACGTATCTCGACGACGTCGCCGGCTTCAACAACGTGCAGATGCAGAACCCGAGCTCGGGCGCGCAGACCTCGCTGAT

GGCGACGTTGTTCGGGCAACTGACGATCGCGTCGTTCTGGCTGCTGGGCGGCATGACGTTCCTGCTCGGCGCGCTGTAC

GAATCGTTCGCCTGGTGGCCGCTCGGATCGTTCGATCCGGTGCCCGCGCCGCTGCTCGAGATGTTCGCGCAGGCGCGCA

TCGACATGCTGATGAACATGATCGCGCGGATCGCGACGCCGATGATGGCGATGCTGTTGCTCGTCGACCTGGGCCTCGC

GTTCGTCGCGCGATCGGCGCAGAAACTCGATCTGATGTCGGTCAGCCAGCCGCTGAAAGGCGCGATCGCGGTGCTGATC

GTCGCGGTGCTGGCGGGGAATTTCGTGAGCGAGATGCGCGGGCAGATTTCGCTCTCGGGACTGGCGCAGCAGATCGGGC

GACTCGCGCGGCAGCCGGCGGGTGGGGTTTCGGGGGTTTCGGGGGTTTCGGGGGTTTCGGGGGTTTCGGGGGTTTCGGG

GGTTTCGGGGGTTTCGGGGGTTTCGGGCGCTTCGGGCGCTTCGGGTGCTTCGGGTGCTTCGGGTGCTTCGGGTGCTTCG

GGTGCTTCGGGTGCTTCGGGTGCTTCGGGTACGTCAAGCACTTCGAGCACTTGGGACGCCACCAGCGTCACAACCGTCC

GCTGA

>Bucl4_Bps_MSHR_305.seq Created: Friday, November 22, 2013 6:28 PM

ATGACGACCTACGACATCTTTGCGCTGCAGCACCTGGGCGACGAATTCATCCGCTTCATCGTGCTGCTCGCGCTGTCGA

GCGAGCGGCTGCTCGTGCTGATGGCGATCCTGCCGGCGACCGCCGACAACGTGATGAAAGGCCCGATGCGCAGCGGCGC

GGCGGCGGTGTGGTGCCTGTTCGTCGCGGGCGGACAGCAGGCGCTGATTCCGCAGCTCCACGGCGCGTTCCTGATGATC

GCCTGCGCGAAGGAGGCCGTGATCGGGCTCGTGCTCGGCATCGCGGCATCGACGGTATTCTGGGCCGCCGAGGCGATCG

GCACGTATCTCGACGACGTCGCCGGCTTCAACAACGTGCAGATGCAGAACCCGAGCTCGGGCGCGCAGACCTCGCTGAT

GGCGACGCTGTTCGGGCAACTGACGATCGCGTCGTTCTGGCTGCTGGGCGGCATGACGTTCCTGCTCGGCGCGCTGTAC

GAATCGTTCGCCTGGTGGCCGCTCGGATCGTTCGATCCGGTGCCCGCGCCGCTGCTCGAGATGTTCGCGCAGGCGCGCA

TCGACATGCTGATGAACATGATCGCGCGGATCGCGACGCCGATGATGGCGATGCTGTTGCTCGTCGACCTGGGCCTCGC

GTTCGTCGCGCGATCGGCGCAGAAACTCGATCTGATGTCGGTCAGCCAGCCGCTGAAAGGCGCGATCGCGGTGCTGATC

GTCGCGGTGCTGGCGGGGAATTTCGTGAGCGAGATGCGCGGGCAGATTTCGCTCTCGGGACTGGCGCAGCAGATCGGGC

GACTCGCGCGGCAGCCGGCGGGTGGGGTTTCGGGGGTTTCGGGGGTTTCGGGCGCTTCGGGCGCTTCGGGCGCTTCGGG

TGCTTCGGGTACGTCAAGCACTTCGAGCACTTGGGACGCCACCAGCGTCACAACCGTCCGCTGA

>Bucl4_Bps_1106a.seq Created: Friday, November 22, 2013 5:46 PM

ATGACGACCTACGACATCTTTGCGCTGCAGCACCTGGGCGACGAATTCATCCGCTTCATCGTGCTGCTCGCGCTGTCGA

GCGAGCGGCTGCTCGTGCTGATGGCGATCCTGCCGGCGACCGCCGACAACGTGATGAAAGGCCCGATGCGCAGCGGCGC

GGCGGCGGTGTGGTGCCTGTTCGTCGCGGGCGGACAGCAGGCGCTGATTCCGCAGCTCCACGGCGCGTTCCTGATGATC

GCCTGCGTGAAGGAGGCCGTGATCGGGCTCGTGCTCGGCATCGCAGCATCGACGGTATTCTGGGCCGCCGAGGCGATCG

GCACGTATCTCGACGACGTCGCCGGCTTCAACAACGTGCAGATGCAGAACCCGAGCTCGGGCGCGCAGACCTCGCTGAT

GGCGACGCTGTTCGGGCAACTGACGATCGCATCGTTCTGGCTGCTGGGCGGCATGACGTTCCTGCTCGGCGCGCTGTAC

GAATCGTTCGCCTGGTGGCCGCTCGGATCGTTCGATCCGGTGCCCGCGCCGCTGCTCGAGATGTTCGCGCAGGCGCGCA

TCGACATGCTGATGAACATGATCGCGCGGATCGCGACGCCGATGATGGCGATGCTGTTGCTCGTCGACCTGGGCCTCGC

GTTCGTCGCGCGATCGGCGCAGAAACTCGATCTGATGTCGGTCAGCCAGCCGCTGAAAGGCGCGATCGCGGTGCTGATC

GTCGCGGTGCTGGCGGGGAATTTCGTGAGCGAGATGCGCGGGCAGATTTCGCTCTCGGGACTGGCGCAGCAGATCGGGC

GACTCGCACGGCAGCCGGCGGGTGGGGTTTCGGGGGTTTCGGGGGTTTCGGGGGTTTCGGGGGTTTCGGGGGTTTCGGG

TGCTTCGGGTGCTTCGGGTGCTTCGGGTGCTTCGGGCGCTTCGGGTACGTCAAGCACTTCGAGCACTTGGGACGCCACC

AGCGTCACAACCGTCCGCTGA

>Bucl4_Bps_1026b.seq Created: Friday, November 22, 2013 6:25 PM

ATGACGACCTACGACATCTTTGCGCTGCAGCACCTGGGCGACGAATTCATCCGCTTCATCGTGCTGCTCGCGCTGTCGA

GCGAGCGGCTGCTCGTGCTGATGGCGATCCTGCCGGCGACCGCCGACAACGTGATGAAAGGCCCGATGCGCAGCGGCGC

GGCGGCGGTGTGGTGCCTGTTCGTCGCGGGCGGACAGCAGGCGCTGATTCCGCAGCTCCACGGCGCGTTCCTGATGATC

GCCTGCGTGAAGGAGGCCGTGATCGGGCTCGTGCTCGGCATCGCGGCATCGACGGTATTCTGGGCCGCCGAGGCGATCG

GCACGTATCTCGACGACGTCGCCGGCTTCAACAACGTGCAGATGCAGAACCCGAGCTCGGGCGCCCAGACCTCGCTGAT

GGCGACGTTGTTCGGGCAACTGACGATCGCGTCGTTCTGGCTGCTGGGCGGCATGACGTTCCTGCTCGGCGCGCTGTAC

GAATCGTTCGCCTGGTGGCCGCTCGGATCGTTCGATCCGGTGCCCGCGCCGCTGCTCGAGATGTTCGCGCAGGCGCGCA

TCGACATGCTGATGAACATGATCGCGCGGATCGCGACGCCGATGATGGCGATGCTGTTGCTCGTCGACCTGGGCCTCGC

GTTCGTCGCGCGATCGGCGCAGAAACTCGATCTGATGTCGGTCAGCCAGCCGCTGAAAGGCGCGATCGCGGTGCTGATC

GTCGCGGTGCTGGCGGGGAATTTCGTGAGCGAGATGCGCGGGCAGATTTCGCTCTCGGGACTGGCGCAGCAGATCGGGC

GACTCGCACGGCAGCCGGCGGGTGGGGTTTCGGGGGTTTCGGGGGTTTCGGGGGTTTCGGGGGTTTCGGGTGCTTCGGG

TGCTTCGGGTGCTTCGGGTGCTTCGGGTACGTCAAGCACTTCGAGCACTTGGGACGCCACCAGCGTCACAACCGTCCGC

TGA

>Bucl4_Bps_1710b.seq Created: Friday, November 22, 2013 5:38 PM

ATGACGACCTACGACATCTTTGCGCTGCAGCACCTGGGCGACGAATTCATCCGCTTCATCGTGCTGCTCGCGCTGTCGA

GCGAGCGGCTGCTCGTGCTGATGGCGATCCTGCCGGCGACCGCCGACAACGTGATGAAAGGCCCGATGCGCAGCGGCGC

GGCGGCGGTGTGGTGCCTGTTCGTCGCGGGCGGACAGCAGGCGCTGATTCCGCAGCTCCACGGCGCGTTCCTGATGATC

GCCTGCGTGAAGGAGGCCGTGATCGGGCTCGTGCTCGGCATCGCGGCATCGACGGTATTCTGGGCCGCCGAGGCGATCG

GCACGTATCTCGACGACGTCGCCGGCTTCAACAACGTGCAGATGCAGAACCCGAGCTCGGGCGCGCAGACCTCGCTGAT

GGCGACGCTGTTCGGGCAACTGACGATCGCGTCGTTCTGGCTGCTGGGCGGCATGACGTTCCTGCTCGGCGCGCTGTAC

GAATCGTTCGCCTGGTGGCCGCTCGGATCGTTCGATCCGGTGCCCGCGCCGCTGCTCGAGATGTTCGCGCAGGCGCGCA

TCGACATGCTGATGAACATGATCGCGCGGATCGCGACGCCGATGATGGCGATGCTGTTGCTCGTCGACCTGGGCCTCGC

GTTCGTCGCGCGATCGGCGCAGAAACTCGATCTGATGTCGGTCAGCCAGCCGCTGAAAGGCGCGATCGCGGTGCTGATC

GTCGCGGTGCTGGCGGGGAATTTCGTGAGCGAGATGCGCGGGCAGATTTCGCTCTCGGGACTGGCGCAGCAGATCGGGC

GACTCGCGCGGCAGCCGGCGGGTGGGGTTTCGGGGGTTTCGGGGGTTTCGGGGGTTTCGGGGGTTTCGGGGGTTTCGGG

GGTTTCGGGGGTTTCGGGGGTTTCGGGGGTTTCGGGGGTTTCGGGGGTTTCGGGTGCTTCGGGCGCTTCGGGTACGTCA

AGCACTTCGAGCCCTTGGGACGCCACCAGCGTCACAACCGTCCGCTGA

>Bucl4_Bps_BPC006.seq Created: Friday, November 22, 2013 5:41 PM

ATGACGACCTACGACATCTTTGCGCTGCAGCACCTGGGCGACGAATTCATCCGCTTCATCGTGCTGCTCGCGCTGTCGA

GCGAGCGGCTGCTCGTGCTGATGGCGATCCTGCCGGCGACCGCCGACAACGTGATGAAAGGCCCGATGCGCAGCGGCGC

GGCGGCGGTGTGGTGCCTGTTCGTCGCGGGCGGACAGCAGGCGCTGATTCCGCAGCTCCACGGCGCGTTCCTGATGATC

GCCTGCGTGAAGGAGGCCGTGATCGGGCTCGTGCTCGGCATCGCAGCATCGACGGTATTCTGGGCCGCCGAGGCGATCG

GCACGTATCTCGACGACGTCGCCGGCTTCAACAACGTGCAGATGCAGAACCCGAGCTCGGGCGCGCAGACCTCGCTGAT

GGCGACGCTGTTCGGGCAACTGACGATCGCATCGTTCTGGCTGCTGGGCGGCATGACGTTCCTGCTCGGCGCGCTGTAC

GAATCGTTCGCCTGGTGGCCGCTCGGATCGTTCGATCCGGTGCCCGCGCCGCTGCTCGAGATGTTCGCGCAGGCGCGCA

TCGACATGCTGATGAACATGATCGCGCGGATCGCGACGCCGATGATGGCGATGCTGTTGCTCGTCGACCTGGGCCTCGC

GTTCGTCGCGCGATCGGCGCAGAAACTCGATCTGATGTCGGTCAGCCAGCCGCTGAAAGGCGCGATCGCGGTGCTGATC

GTCGCGGTGCTGGCGGGGAATTTCGTGAGCGAGATGCGCGGGCAGATTTCGCTCTCGGGACTGGCGCAGCAGATCGGGC

GACTCGCACGGCAGCCGGCGGGTGGGGTTTCGGGGGTTTCGGGGGTTTCGGGGGTTTCGGGGGTTTCGGGGGTTTCGGG

TGCTTCGGGTGCTTCGGGTGCTTCGGGTGCTTCGGGCGCTTCGGGTACGTCAAGCACTTCGAGCACTTGGGACGCCACC

AGCGTCACAACCGTCCGCTGA

>Bucl4_Bps_668.seq Created: Friday, November 22, 2013 6:11 PM

ATGACGACCTACGACATCTTTGCGCTGCAGCACCTGGGCGACGAATTCATCCGCTTCATCGTGCTGCTCGCGCTGTCGA

GCGAGCGGCTGCTCGTGCTGATGGCGATCCTGCCGGCGACCGCCGACAACGTGATGAAAGGCCCGATGCGCAGCGGCGC

GGCGGCGGTGTGGTGCCTGTTCGTCGCGGGCGGACAGCAGGCGCTGATTCCGCAGCTCCACGGCGCGTTCCTGATGATC

GCCTGCGTGAAGGAGGCCGTGATCGGGCTCGTGCTCGGCATCGCCGCATCGACGGTATTCTGGGCCGCCGAGGCGATCG

GCACGTATCTCGACGACGTCGCCGGCTTCAACAACGTGCAGATGCAGAACCCGAGCTCGGGCGCGCAGACCTCGCTGAT

GGCGACGCTGTTCGGGCAACTGACGATCGCATCGTTCTGGCTGCTGGGCGGCATGACGTTCCTGCTCGGCGCGCTGTAC

GAATCGTTCGCCTGGTGGCCGCTCGGATCGTTCGATCCGGTGCCCGCGCCGCTGCTCGAGATGTTCGCGCAGGCGCGCA

TCGACATGCTGATGAACATGATCGCGCGGATCGCGACGCCGATGATGGCGATGCTGTTGCTCGTCGACCTGGGCCTCGC

GTTCGTCGCGCGATCGGCGCAGAAACTCGATCTGATGTCGGTCAGCCAGCCGCTGAAAGGCGCGATCGCGGTGCTGATC

GTCGCGGTGCTGGCGGGGAATTTCGTGAGCGAGATGCGCGGGCAGATTTCGCTCTCGGGACTGGCGCAGCAGATCGGGC

GACTCGCACGGCAGCCGGCGGGTGGGGTTTCGGGGGTTTCGGGGGTTTCGGGGGTTTCGGGGGTTTCGGGGGTTTCGGG

GGTTTCGGGGGTTTCGGGTGCTTCGGGTGCTTCGGGCGCTTCGGGTACGTCAAGCACTTCGAGCACTTGGGACGCCACC

AGCGTCACAACCGTCCGCTGA

>Bucl4_Bps_NCTC_13179.seq Created: Friday, November 22, 2013 1:53 PM

ATGACGACCTACGACATCTTTGCGCTGCAGCACCTGGGCGACGAATTCATCCGCTTCATCGTGCTGCTCGCGCTGTCGA

GCGAGCGGCTGCTCGTGCTGATGGCGATCCTGCCGGCGACCGCCGACAACGTGATGAAAGGCCCGATGCGCAGCGGCGC

GGCGGCGGTGTGGTGCCTGTTCGTCGCGGGCGGACAGCAGGCGCTGATTCCGCAGCTCCACGGCGCGTTCCTGATGATC

GCCTGCGTGAAGGAGGCCGTGATCGGGCTCGTGCTCGGCATCGCGGCATCGACGGTATTCTGGGCCGCCGAGGCGATCG

GCACGTATCTCGACGACGTCGCCGGCTTCAACAACGTGCAGATGCAGAACCCGAGCTCGGGCGCCCAGACCTCGCTGAT

GGCGACGTTGTTCGGGCAACTGACGATCGCGTCGTTCTGGCTGCTGGGCGGCATGACGTTCCTGCTCGGCGCGCTGTAC

GAATCGTTCGCCTGGTGGCCGCTCGGATCGTTCGATCCGGTGCCCGCGCCGCTGCTCGAGATGTTCGCGCAGGCGCGCA

TCGACATGCTGATGAACATGATCGCGCGGATCGCGACGCCGATGATGGCGATGCTGTTGCTCGTCGACCTGGGCCTCGC

GTTCGTCGCGCGATCGGCGCAGAAACTCGATCTGATGTCGGTCAGCCAGCCGCTGAAAGGCGCGATCGCGGTGCTGATC

GTCGCGGTGCTGGCGGGGAATTTCGTGAGCGAGATGCGCGGGCAGATTTCGCTCTCGGGACTGGCGCAGCAGATCGGGC

GACTCGCGCGGCAGCCGGCGGGTGGGGTTTCGGGGGTTTCGGGGGTTTCGGGGGTTTCGGGTGCTTCGGGTGCTTCGGG

CGCTTCGGGTGCTTCGGGTGCTTCGGGTGCTTCGGGCGCTTCGGGTGCTTCGGGCGCTTCGGGCGCTTCGGGTACGTCA

AGCACTTCGAGCCCTTGGGACGCCACCAGCGTCACAACCGTCCGCTGA

>Bucl4_Bps_MSHR146.seq Created: Wednesday, March 19, 2014 4:14 PM

ATGACGACCTACGACATCTTTGCGCTGCAGCACCTGGGCGACGAATTCATCCGCTTCATCGTGCTGCTCGCGCTGTCGA

GCGAGCGGCTGCTCGTGCTGATGGCGATCCTGCCGGCGACCGCCGACAACGTGATGAAAGGCCCGATGCGCAGCGGCGC

GGCGGCGGTGTGGTGCCTGTTCGTCGCGGGCGGACAGCAGGCGCTGATTCCGCAGCTCCACGGCGCGTTCCTGATGATC

GCCTGCGTGAAGGAGGCCGTGATCGGGCTCGTGCTCGGCATCGCGGCATCGACGGTATTCTGGGCCGCCGAGGCGATCG

GCACGTATCTCGACGACGTCGCCGGCTTCAACAACGTGCAGATGCAGAACCCGAGCTCGGGCGCGCAGACCTCGCTAAT

GGCGACGTTGTTCGGGCAACTGACGATCGCGTCGTTCTGGCTGCTGGGCGGCATGACGTTCCTGCTCGGCGCGCTGTAC

GAATCCTTCGCCTGGTGGCCGCTCGGATCGTTCGATCCGGTGCCCGCGCCGCTGCTCGAGATGTTCGCGCAGGCGCGCA

TCGACATGCTGATGAACATGATCGCGCGGATCGCGACGCCGATGATGGCGATGCTGTTGCTCGTCGACCTGGGCCTCGC

GTTCGTCGCGCGATCGGCGCAGAAACTCGATCTGATGTCGGTCAGCCAGCCGCTGAAAGGCGCGATCGCGGTGCTGATC

GTCGCGGTGCTGGCGGGGAATTTCGTGAGCGAGATGCGCGGGCAGATTTCGCTCTCGGGACTGGCGCAGCAGATCGGGC

GACTCGCACGGCAGCCGGCGGGTGGGGTTTCGGGGGTTTCGGGGGTTTCGGGGGTTTCGGGTGCTTCGGGTCCTTCGGG

TGCTTCGGGTGCTTCGGGTGCTTCGGGTGCTTCGGGTGCTTCGGGTGCTTCGGGTGCTTCGGGTGCTTCGGGTGCTTCG

GGTGCTTCGGGTGCTTCGGGTGCTTCGGGTGCTTCGGGCGCTTCGGGTGCTTCGGGTGCTTCGGGTACGTCAAGCACTT

CGAGCACTTGGGACGCCACCAGCGTCACAACCGTCCGCTGA

>Bucl4_Bps_MSHR511.seq Reverse Complement DNA Sequence Untitled Seq #24(1,1140)

ATGACGACCTACGACATCTTTGCGCTGCAGCACCTGGGCGACGAATTCATCCGCTTCATCGTGCTGCTCGCGCTGTCGA

GCGAGCGGCTGCTCGTGCTGATGGCGATCCTGCCGGCGACCGCCGACAACGTGATGAAAGGCCCGATGCGCAGCGGCGC

GGCGGCGGTGTGGTGCCTGTTCGTCGCGGGCGGACAGCAGGCGCTGATTCCGCAGCTCCACGGCGCGTTCCTGATGATC

GCCTGCGTGAAGGAGGCCGTGATCGGGCTCGTGCTCGGCATCGCGGCATCGACGGTATTCTGGGCCGCCGAGGCGATCG

GCACGTATCTCGACGACGTCGCCGGCTTCAACAACGTGCAGATGCAGAACCCGAGCTCGGGCGCGCAGACCTCGCTAAT

GGCGACGTTGTTCGGGCAACTGACGATCGCGTCGTTCTGGCTGCTGGGCGGCATGACGTTCCTGCTCGGCGCGCTGTAC

GAATCCTTCGCCTGGTGGCCGCTCGGATCGTTCGATCCGGTGCCCGCGCCGCTGCTCGAGATGTTCGCGCAGGCGCGCA

TCGACATGCTGATGAACATGATCGCGCGGATCGCGACGCCGATGATGGCGATGCTGTTGCTCGTCGACCTGGGCCTCGC

GTTCGTCGCGCGATCGGCGCAGAAACTCGATCTGATGTCGGTCAGCCAGCCGCTGAAAGGCGCGATCGCGGTGCTGATC

GTCGCGGTGCTGGCGGGGAATTTCGTGAGCGAGATGCGCGGGCAGATTTCGCTCTCGGGACTGGCGCAGCAGATCGGGC

GACTCGCACGGCAGCCGGCGGGTGGGGTTTCGGGGGTTTCGGGGGTTTCGGGGGTTTCGGGGGTTTCGGGGGTTTCGGG

GGTTTCGGGGGTTTCGGGGGTTTCGGGGGTTTCGGGTGCTTCGGGTGCTTCGGGTGCTTCGGGTGCTTCGGGTGCTTCG

GGTGCTTCGGGTGCTTCGGGTGCTTCGGGTGCTTCGGGTGCTTCGGGTGCTTCGGGTGCTTCGGGTGCTTCGGGTGCTT

CGGGTGCTTCGGGTGCTTCGGGTGCTTCGGGCGCTTCGGGTGCTTCGGGTGCTTCGGGTACGTCAAGCACTTCGAGCAC

TTGGGACGCCACCAGCGTCACAACCGTCCGCTGA

>Bucl4_Bps_MSHR520.seq Created: Wednesday, March 19, 2014 4:18 PM

ATGACGACCTACGACATCTTTGCGCTGCAGCACCTGGGCGACGAATTCATCCGCTTCATCGTGCTGCTCGCGCTGTCGA

GCGAGCGGCTGCTCGTGCTGATGGCGATCCTGCCGGCGACCGCCGACAACGTGATGAAAGGCCCGATGCGCAGCGGCGC

GGCGGCGGTGTGGTGCCTGTTCGTCGCGGGCGGACAGCAGGCGCTGATTCCGCAGCTCCACGGCGCGTTCCTGATGATC

GCCTGCGCGAAGGAGGCCGTGATCGGGCTCGTGCTCGGCATCGCGGCATCGACGGTATTCTGGGCCGCCGAGGCGATCG

GCACGTATCTCGACGACGTCGCCGGCTTCAACAACGTGCAGATGCAGAACCCGAGCTCGGGCGCGCAGACCTCGCTGAT

GGCGACGCTGTTCGGGCAACTGACGATCGCGTCGTTCTGGCTGCTGGGCGGCATGACGTTCCTGCTCGGCGCGCTGTAC

GAATCGTTCGCCTGGTGGCCGCTCGGATCGTTCGATCCGGTGCCCGCGCCGCTGCTCGAGATGTTCGCGCAGGCGCGCA

TCGACATGCTGATGAACATGATCGCGCGGATCGCGACGCCGATGATGGCGATGCTGTTGCTCGTCGACCTGGGCCTCGC

GTTCGTCGCGCGATCGGCGCAGAAACTCGATCTGATGTCGGTCAGCCAGCCGCTGAAAGGCGCGATCGCGGTGCTGATC

GTCGCGGTGCTGGCGGGGAATTTCGTGAGCGAGATGCGCGGGCAGATTTCGCTCTCGGGACTGGCGCAGCAGATCGGGC

GACTCGCGCGGCAGCCGGCGGGTGGGGTTTCGGGGGTTTCGGGGGTTTCGGGCGCTTCGGGCGCTTCGGGCGCTTCGGG

TGCTTCGGGTACGTCAAGCACTTCGAGCACTTGGGACGCCACCAGCGTCACAACCGTCCGCTGA

>Bucl4_Bps_NAU20B-16.seq Reverse Complement DNA Sequence Untitled Seq #27(1,1126)

ATGACGACCTACGACATCTTTGCGCTGCAGCACCTGGGCGACGAATTCATCCGCTTCATCGTGCTGCTCGCGCTGTCGA

GCGAGCGGCTGCTCGTGCTGATGGCGATCCTGCCGGCGACCGCCGACAACGTGATGAAAGGCCCGATGCGCAGCGGCGC

GGCGGCGGTGTGGTGCCTGTTCGTCGCGGGCGGACAGCAGGCGCTGATTCCGCAGCTCCACGGCGCGTTCCTGATGATC

GCCTGCGTGAAGGAGGCCGTGATCGGGCTCGTGCTCGGCATCGCGGCATCGACGGTATTCTGGGCCGCCGAGGCGATCG

GCACGTATCTCGACGACGTCGCCGGCTTCAACAACGTGCAGATGCAGAACCCGAGCTCGGGCGCGCAGACCTCGCTAAT

GGCGACGTTGTTCGGGCAACTGACGATCGCGTCGTTCTGGCTGCTGGGCGGCATGACGTTCCTGCTCGGCGCGCTGTAC

GAATCCTTCGCCTGGTGGCCGCTCGGATCGTTCGATCCGGTGCCCGCGCCGCTGCTCGAGATGTTCGCGCAGGCGCGCA

TCGACATGCTGATGAACATGATCGCGCGGATCGCGACGCCGATGATGGCGATGCTGTTGCTCGTCGACCTGGGCCTCGC

GTTCGTCGCGCGATCGGCGCAGAAACTCGATCTGATGTCGGTCAGCCAGCCGCTGAAAGGCGCGATCGCGGTGCTGATC

GTCGCGGTGCTGGCGGGGAATTTCGTGAGCGAGATGCGCGGGCAGATTTCGCTCTCGGGACTGGCGCAGCAGATCGGGC

GACTCGCACGGCAGCCGGCGGGTGGGGTTTCGGGGGTTTCGGGGGTTTCGGGGGTTTCGGGGGTTTCGGGGGTTTCGGG

GGTTTCGGGGTTCGGGGTTCGGTGCTTCGGGTGCTTCGGGTGCTTCGGGTGCTTCGGGTGCTTCGGGTGCTTCGGGTGC

TTCGGGTGCTTCGGGTGCTTCGGGTGCTTCGGGTGCTTCGGGTGCTTCGGGTGCTTCGGGTGCTTCGGGTGCTTCGGGT

GCTTCGGGTGCTTCGGGCGCTTCGGGTGCTTCGGGTGCTTCGGGTACGTCAAGCACTTCGAGCACTTGGGACGCCACCA

GCGTCACAACCGTCCGCTGA

>Bucl4_Bps_NCTC_13178.seq Created: Wednesday, March 19, 2014 3:49 PM

ATGACGACCTACGACATCTTTGCGCTGCAGCACCTGGGCGACGAATTCATCCGCTTCATCGTGCTGCTCGCGCTGTCGA

GCGAGCGGCTGCTCGTGCTGATGGCGATCCTGCCGGCGACCGCCGACAACGTGATGAAAGGCCCGATGCGCAGCGGCGC

GGCGGCGGTGTGGTGCCTGTTCGTCGCGGGCGGACAGCAGGCGCTGATTCCGCAGCTCCACGGCGCGTTCCTGATGATC

GCCTGCGTGAAGGAGGCCGTGATCGGGCTCGTGCTCGGCATCGCGGCATCGACGGTATTCTGGGCCGCCGAGGCGATCG

GCACGTATCTCGACGACGTCGCCGGCTTCAACAACGTGCAGATGCAGAACCCGAGCTCGGGCGCGCAGACCTCGCTGAT

GGCGACGTTGTTCGGGCAACTGACGATCGCGTCGTTCTGGCTGCTGGGCGGCATGACGTTCCTGCTCGGCGCGCTGTAC

GAATCCTTCGCCTGGTGGCCGCTCGGATCGTTCGATCCGGTGCCCGCGCCGCTGCTCGAGATGTTCGCGCAGGCGCGCA

TCGACATGCTGATGAACATGATCGCGCGGATCGCGACGCCGATGATGGCGATGCTGTTGCTCGTCGACCTGGGCCTCGC

GTTCGTCGCGCGATCGGCGCAGAAACTCGATCTGATGTCGGTCAGCCAGCCGCTGAAAGGCGCGATCGCGGTGCTGATC

GTCGCGGTGCTGGCGGGGAATTTCGTGAACGAGATGCGCGGGCAGATTTCGCTCTCGGGACTGGCGCAGCAGATCGGGC

GACTCGCACGGCAGCCGGCGGGTGGGGTTTCGGGGGTTTCGGGGGTTTCGGGGGTTTCGGGGGTTTCGGGGGTTTCGGG

GGTTTCGGGGGTTTCGGGCGCTTCGGGCGCTTCGGGCGCTTCGGGTGCTTCGGGTGCTTCGGGTGCTTCGGGTGCTTCG

GGTGCTTCGGGCGCTTCGGGCGCTTCGGGCGCTTCGGGTACGTCAAGCACTTCGAGCACTTGGGACGCCACCAGCGTCA

CAACCGTCCGCTGA

>B1H7D0_BURPS_Bps_S13.seq Created: Wednesday, April 10, 2013 2:33 PM

ATGACGACCTACGACATCTTTGCGCTGCAGCACCTGGGCGACGAATTCATCCGCTTCATCGTGCTGCTCGCGCTGTCGA

GCGAGCGGCTGCTCGTGCTGATGGCGATCCTGCCGGCGACCGCCGACAACGTGATGAAAGGCCCGATGCGCAGCGGCGC

GGCGGCGGTGTGGTGCCTGTTCGTCGCGGGCGGACAGCAGGCGCTGATTCCGCAGCTCCACGGCGCGTTCCTGATGATC

GCCTGCGTGAAGGAGGCCGTGATCGGGCTCGTGCTCGGCATCGCGGCATCGACGGTATTCTGGGCCGCCGAGGCGATCG

GCACGTATCTCGACGACGTCGCCGGCTTCAACAACGTGCAGATGCAGAACCCGAGCTCGGGCGCCCAGACCTCGCTGAT

GGCGACGTTGTTCGGGCAACTGACGATCGCGTCGTTCTGGCTGCTGGGCGGCATGACGTTCCTGCTCGGCGCGCTGTAC

GAATCGTTCGCCTGGTGGCCGCTCGGATCGTTCGATCCGGTGCCCGCGCCGCTGCTCGAGATGTTCGCGCAGGCGCGCA

TCGACATGCTGATGAACATGATCGCGCGGATCGCGACGCCGATGATGGCGATGCTGTTGCTCGTCGACCTGGGCCTCGC

GTTCGTCGCGCGATCGGCGCAGAAACTCGATCTGATGTCGGTCAGCCAGCCGCTGAAAGGCGCGATCGCGGTGCTGATC

GTCGCGGTGCTGGCGGGGAATTTCGTGAGCGAGATGCGCGGGCAGATTTCGCTCTCGGGACTGGCGCAGCAGATCGGGC

GACTCGCACGGCAGCCGGCGGGTGGGGTTTCGGGGGTTTCGGGGGTTTCGGGGGTTTCGGGGGTTTCGGGGGTTTCGGG

GGTTTCGGGGGTTTCGGGTGCTTCGGGTGCTTCGGGTGCTTCGGGTGCTTCGGGTGCTTCGGGTGCTTCGGGTGCTTCG

GGTGCTTCGGGTGCTTCGGGTGCTTCGGGTGCTTCGGGTGCTTCGGGTGCTTCGGGTGCTTCGGGCGCTTCGGGTACGT

CAAGCACTTCGAGCACTTGGGACGCCACCAGCGTCACAACCGTCCGCTGA

>B2HC63_BURPS_Bps_1655.seq Reverse Complement DNA Sequence Untitled Seq #5(1,1113)

ATGACGACCTACGACATCTTTGCGCTGCAGCACCTGGGCGACGAATTCATCCGCTTCATCGTGCTGCTCGCGCTGTCGA

GCGAGCGGCTGCTCGTGCTGATGGCGATCCTGCCGGCGACCGCCGACAACGTGATGAAAGGCCCGATGCGCAGCGGCGC

GGCGGCGGTGTGGTGCCTGTTCGTCGCGGGCGGACAGCAGGCGCTGATTCCGCAGCTCCACGGCGCGTTCCTGATGATC

GCCTGCGTGAAGGAGGCCGTGATCGGGCTCGTGCTCGGCATCGCGGCATCGACGGTATTCTGGGCCGCCGAGGCGATCG

GCACGTATCTCGACGACGTCGCCGGCTTCAACAACGTGCAGATGCAGAACCCGAGCTCGGGCGCGCAGACCTCGCTAAT

GGCGACGTTGTTCGGGCAACTGACGATCGCGTCGTTCTGGCTGCTGGGCGGCATGACGTTCCTGCTCGGCGCGCTGTAC

GAATCCTTCGCCTGGTGGCCGCTCGGATCGTTCGATCCGGTGCCCGCGCCGCTGCTCGAGATGTTCGCGCAGGCGCGCA

TCGACATGCTGATGAACATGATCGCGCGGATCGCGACGCCGATGATGGCGATGCTGTTGCTCGTCGACCTGGGCCTCGC

GTTCGTCGCGCGATCGGCGCAGAAACTCGATCTGATGTCGGTCAGCCAGCCGCTGAAAGGCGCGATCGCGGTGCTGATC

GTCGCGGTGCTGGCGGGGAATTTCGTGAGCGAGATGCGCGGGCAGATTTCGCTCTCGGGACTGGCGCAGCAGATCGGGC

GACTCGCACGGCAGCCGGCGGGTGGGGTTTCGGGGGTTTCGGGGGTTTCGGGGGTTTCGGGGGTTTCGGGGGTTTCGGG

TGCTTCGGGTGCTTCGGGTGCTTCGGGTGCTTCGGGTGCTTCGGGTGCTTCGGGTGCTTCGGGTGCTTCGGGTGCTTCG

GGTGCTTCGGGTGCTTCGGGTGCTTCGGGTGCTTCGGGTGCTTCGGGTGCTTCGGGTGCTTCGGGTGCTTCGGGTGCTT

CGGGCGCTTCGGGTGCTTCGGGTGCTTCGGGTACGTCAAGCACTTCGAGCACTTGGGACGCCACCAGCGTCACAACCGT

CCGCTGA

>Bucl4_Bm_2000031281.seq Created: Monday, March 24, 2014 5:17 PM

ATGACGACCTACGACATCTTTGCGCTGCAGCACCTGGGCGACGAATTCATCCGCTTCATCGTGCTGCTCGCGCTGTCGA

GCGAGCGGCTGCTCGTGCTGATGGCGATCCTGCCGGCGACCGCCGACAACGTGATGAAAGGCCCGATGCGCAGCGGCGC

GGCGGCGGTGTGGTGCCTGTTCGTCGCGGGCGGACAGCAGGCGCTGATTCCGCAGCTCCACGGCGCGTTCCTGATGATC

GCCTGCGTGAAGGAGGCCGTGATCGGGCTCGTGCTCGGCATCGCGGCATCGACGGTATTCTGGGCCGCCGAGGCGATCG

GCACGTATCTCGACGACGTCGCCGGCTTCAACAACGTGCAGATGCAGAACCCGAGCTCGGGCGCGCAGACCTCGCTGAT

GGCGACGCTGTTCGGGCAACTGACGATCGCGTCGTTCTGGCTGCTGGGCGGCATGACGTTCCTGCTCGGCGCGCTGTAC

GAATCGTTCGCCTGGTGGCCGTTCGGATCGTTCGATCCGGTGCCCGCGCCGCTGCTCGAGATGTTCGCGCAGGCGCGCA

TCGACATGCTGATGAACATGATCGCGCGGATCGCGACGCCGATGATGGCGATGCTGTTGCTCGTCGACCTGGGCCTCGC

GTTCGTCGCGCGATCGGCGCAGAAACTCGATCTGATGTCGGTCAGCCAGCCGCTGAAAGGCGCGATCGCGGTGCTGATC

GTCGCGGTGCTGGCGGGGAATTTCGTGAGCGAGATGCGCGGGCAGATTTCGCTCTCGGGACTGGCGCAGCAGATCGGGC

GACTCGCGCGGCAGCCGGCGGGTGGGGTTTCGGGGGTTTCGGGGGTTTCGGGGGTTTCGGGGGTTTCGGGGGTTTCGGG

TGCTTCGGGTGCTTCGGGTGCTTCGGGCGCTTCGGGCGCTTCGGGCGCTTCGGGTGCTTCGGGCGCTTCGGGCGCTTCG

GGTACGTCAAGCACTTCGAGCACTTGGGACGCCACCAGCGTCACAACCGTCCGCTGA

>A5TN55_BURMA_Bm_2002721280.seq Created: Tuesday, February 12, 2013 5:14 PM

ATGACGACCTACGACATCTTTGCGCTGCAGCACCTGGGCGACGAATTCATCCGCTTCATCGTGCTGCTCGCGCTGTCGA

GCGAGCGGCTGCTCGTGCTGATGGCGATCCTGCCGGCGACCGCCGACAACGTGATGAAAGGCCCGATGCGCAGCGGCGC

GGCGGCGGTGTGGTGCCTGTTCGTCGCGGGCGGACAGCAGGCGCTGATTCCGCAGCTCCACGGCGCGTTCCTGATGATC

GCCTGCGTGAAGGAGGCCGTGATCGGGCTCGTGCTCGGCATCGCGGCATCGACGGTATTCTGGGCCGCCGAGGCGATCG

GCACGTATCTCGACGACGTCGCCGGCTTCAACAACGTGCAGATGCAGAACCCGAGCTCGGGCGCGCAGACCTCGCTGAT

GGCGACGCTGTTCGGGCAACTGACGATCGCGTCGTTCTGGCTGCTGGGCGGCATGACGTTCCTGCTCGGCGCGCTGTAC

GAATCGTTCGCCTGGTGGCCGTTCGGATCGTTCGATCCGGTGCCCGCGCCGCTGCTCGAGATGTTCGCGCAGGCGCGCA

TCGACATGCTGATGAACATGATCGCGCGGATCGCGACGCCGATGATGGCGATGCTGTTGCTCGTCGACCTGGGCCTCGC

GTTCGTCGCGCGATCGGCGCAGAAACTCGATCTGATGTCGGTCAGCCAGCCGCTGAAAGGCGCGATCGCGGTGCTGATC

GTCGCGGTGCTGGCGGGGAATTTCGTGAGCGAGATGCGCGGGCAGATTTCGCTCTCGGGACTGGCGCAGCAGATCGGGC

GACTCGCGCGGCAGCCGGCGGGTGGGGTTTCGGGGGTTTCGGGGGTTTCGGGGGTTTCGGGGGTTTCGGGGGTTTCGGG

TGCTTCGGGTGCTTCGGGCGCTTCGGGCGCTTCGGGCGCTTCGGGCGCTTCGGGCGCTTCGGGCGCTTCGGGCGCTTCG

GGCGCTTCGGGTACGTCAAGCACTTCGAGCACTTGGGACGCCACCAGCGTCACAACCGTCCGCTGA

>Bucl4_Bm_A188.seq Reverse Complement DNA Sequence Untitled Seq #14(1,1041)

ATGACGACCTACGACATCTTTGCGCTGCAGCACCTGGGCGACGAATTCATCCGCTTCATCGTGCTGCTCGCGCTGTCGA

GCGAGCGGCTGCTCGTGCTGATGGCGATCCTGCCGGCGACCGCCGACAACGTGATGAAAGGCCCGATGCGCAGCGGCGC

GGCGGCGGTGTGGTGCCTGTTCGTCGCGGGCGGACAGCAGGCGCTGATTCCGCAGCTCCACGGCGCGTTCCTGATGATC

GCCTGCGTGAAGGAGGCCGTGATCGGGCTCGTGCTCGGCATCGCGGCATCGACGGTATTCTGGGCCGCCGAGGCGATCG

GCACGTATCTCGACGACGTCGCCGGCTTCAACAACGTGCAGATGCAGAACCCGAGCTCGGGCGCGCAGACCTCGCTGAT

GGCGACGCTGTTCGGGCAACTGACGATCGCGTCGTTCTGGCTGCTGGGCGGCATGACGTTCCTGCTCGGCGCGCTGTAC

GAATCGTTCGCCTGGTGGCCGTTCGGATCGTTCGATCCGGTGCCCGCGCCGCTGCTCGAGATGTTCGCGCAGGCGCGCA

TCGACATGCTGATGAACATGATCGCGCGGATCGCGACGCCGATGATGGCGATGCTGTTGCTCGTCGACCTGGGCCTCGC

GTTCGTCGCGCGATCGGCGCAGAAACTCGATCTGATGTCGGTCAGCCAGCCGCTGAAAGGCGCGATCGCGGTGCTGATC

GTCGCGGTGCTGGCGGGGAATTTCGTGAGCGAGATGCGCGGGCAGATTTCGCTCTCGGGACTGGCGCAGCAGATCGGGC

GACTCGCGCGGCAGCCGGCGGGTGGGGTTTCGGGGGTTTCGGGGGTTTCGGGGGTTTCGGGGGTTTCGGGGGTTTCGGG

GGTTTCGGGGGTTTCGGGGGTTTCGGGGGTTTCGGGTGCTTCGGGTGCTTCGGGTGCTTCGGGCGCTTCGGGCGCTTCG

GGCGCTTCGGGTGCTTCGGGCGCTTCGGGCGCTTCGGGTACGTCAAGCACTTCGAGCACTTGGGACGCCACCAGCGTCA

CAACCGTCCGCTGA

>Bucl4_Bm_A193.seq Reverse Complement DNA Sequence Untitled Seq #25(1,1059)

ATGACGACCTACGACATCTTTGCGCTGCAGCACCTGGGCGACGAATTCATCCGCTTCATCGTGCTGCTCGCGCTGTCGA

GCGAGCGGCTGCTCGTGCTGATGGCGATCCTGCCGGCGACCGCCGACAACGTGATGAAAGGCCCGATGCGCAGCGGCGC

GGCGGCGGTGTGGTGCCTGTTCGTCGCGGGCGGACAGCAGGCGCTGATTCCGCAGCTCCACGGCGCGTTCCTGATGATC

GCCTGCGTGAAGGAGGCCGTGATCGGGCTCGTGCTCGGCATCGCGGCATCGACGGTATTCTGGGCCGCCGAGGCGATCG

GCACGTATCTCGACGACGTCGCCGGCTTCAACAACGTGCAGATGCAGAACCCGAGCTCGGGCGCGCAGACCTCGCTGAT

GGCGACGCTGTTCGGGCAACTGACGATCGCGTCGTTCTGGCTGCTGGGCGGCATGACGTTCCTGCTCGGCGCGCTGTAC

GAATCGTTCGCCTGGTGGCCGTTCGGATCGTTCGATCCGGTGCCCGCGCCGCTGCTCGAGATGTTCGCGCAGGCGCGCA

TCGACATGCTGATGAACATGATCGCGCGGATCGCGACGCCGATGATGGCGATGCTGTTGCTCGTCGACCTGGGCCTCGC

GTTCGTCGCGCGATCGGCGCAGAAACTCGATCTGATGTCGGTCAGCCAGCCGCTGAAAGGCGCGATCGCGGTGCTGATC

GTCGCGGTGCTGGCGGGGAATTTCGTGAGCGAGATGCGCGGGCAGATTTCGCTCTCGGGACTGGCGCAGCAGATCGGGC

GACTCGCGCGGCAGCCGGCGGGTGGGGTTTCGGGGGTTTCGGGGGTTTCGGGGGTTTCGGGGGTTTCGGGGGTTTCGGG

GGTTTCGGGGGTTTCGGGGGTTTCGGGGGTTTCGGGGGTTTCGGGTGCTTCGGGTGCTTCGGGTGCTTCGGGCGCTTCG

GGCGCTTCGGGCGCTTCGGGCGCTTCGGGTGCTTCGGGCGCTTCGGGCGCTTCGGGTACGTCAAGCACTTCGAGCACTT

GGGACGCCACCAGCGTCACAACCGTCCGCTGA

>Bucl4_Bm_China_7.seq Reverse Complement DNA Sequence Untitled Seq #14(1,1005)

ATGACGACCTACGACATCTTTGCGCTGCAGCACCTGGGCGACGAATTCATCCGCTTCATCGTGCTGCTCGCGCTGTCGA

GCGAGCGGCTGCTCGTGCTGATGGCGATCCTGCCGGCGACCGCCGACAACGTGATGAAAGGCCCGATGCGCAGCGGCGC

GGCGGCGGTGTGGTGCCTGTTCGTCGCGGGCGGACAGCAGGCGCTGATTCCGCAGCTCCACGGCGCGTTCCTGATGATC

GCCTGCGTGAAGGAGGCCGTGATCGGGCTCGTGCTCGGCATCGCGGCATCGACGGTATTCTGGGCCGCCGAGGCGATCG

GCACGTATCTCGACGACGTCGCCGGCTTCAACAACGTGCAGATGCAGAACCCGAGCTCGGGCGCGCAGACCTCGCTGAT

GGCGACGCTGTTCGGGCAACTGACGATCGCGTCGTTCTGGCTGCTGGGCGGCATGACGTTCCTGCTCGGCGCGCTGTAC

GAATCGTTCGCCTGGTGGCCGTTCGGATCGTTCGATCCGGTGCCCGCGCCGCTGCTCGAGATGTTCGCGCAGGCGCGCA

TCGACATGCTGATGAACATGATCGCGCGGATCGCGACGCCGATGATGGCGATGCTGTTGCTCGTCGACCTGGGCCTCGC

GTTCGTCGCGCGATCGGCGCAGAAACTCGATCTGATGTCGGTCAGCCAGCCGCTGAAAGGCGCGATCGCGGTGCTGATC

GTCGCGGTGCTGGCGGGGAATTTCGTGAGCGAGATGCGCGGGCAGATTTCGCTCTCGGGACTGGCGCAGCAGATCGGGC

GACTCGCGCGGCAGCCGGCGGGTGGGGTTTCGGGGGTTTCGGGGGTTTCGGGGGTTTCGGGGGTTTCGGGGGTTTCGGG

TGCTTCGGGTGCTTCGGGTGCTTCGGGCGCTTCGGGCGCTTCGGGCGCTTCGGGTGCTTCGGGCGCTTCGGGCGCTTCG

GGTACGTCAAGCACTTCGAGCACTTGGGACGCCACCAGCGTCACAACCGTCCGCTGA

>Bucl4_Bm_PRL-20.seq Created: Monday, March 24, 2014 4:04 PM

ATGACGACCTACGACATCTTTGCGCTGCAGCACCTGGGCGACGAATTCATCCGCTTCATCGTGCTGCTCGCGCTGTCGA

GCGAGCGGCTGCTCGTGCTGATGGCGATCCTGCCGGCGACCGCCGACAACGTGATGAAAGGCCCGATGCGCAGCGGCGC

GGCGGCGGTGTGGTGCCTGTTCGTCGCGGGCGGACAGCAGGCGCTGATTCCGCAGCTCCACGGCGCGTTCCTGATGATC

GCCTGCGTGAAGGAGGCCGTGATCGGGCTCGTGCTCGGCATCGCGGCATCGACGGTATTCTGGGCCGCCGAGGCGATCG

GCACGTATCTCGACGACGTCGCCGGCTTCAACAACGTGCAGATGCAGAACCCGAGCTCGGGCGCGCAGACCTCGCTGAT

GGCGACGCTGTTCGGGCAACTGACGATCGCGTCGTTCTGGCTGCTGGGCGGCATGACGTTCCTGCTCGGCGCGCTGTAC

GAATCGTTCGCCTGGTGGCCGTTCGGATCGTTCGATCCGGTGCCCGCGCCGCTGCTCGAGATGTTCGCGCAGGCGCGCA

TCGACATGCTGATGAACATGATCGCGCGGATCGCGACGCCGATGATGGCGATGCTGTTGCTCGTCGACCTGGGCCTCGC

GTTCGTCGCGCGATCGGCGCAGAAACTCGATCTGATGTCGGTCAGCCAGCCGCTGAAAGGCGCGATCGCGGTGCTGATC

GTCGCGGTGCTGGCGGGGAATTTCGTGAGCGAGATGCGCGGGCAGATTTCGCTCTCGGGACTGGCGCAGCAGATCGGGC

GACTCGCGCGGCAGCCGGCGGGTGGGGTTTCGGGGGTTTCGGGGGTTTCGGGGGTTTCGGGGGTTTCGGGGGTTTCGGG

GGTTTCGGGGGTTTCGGGGGTTTCGGGGGTTTCGGGGGTTTCGGGTGCTTCGGGTGCTTCGGGCGCTTCGGGCGCTTCG

GGTGCTTCGGGCGCTTCGGGCGCTTCGGGTACGTCAAGCACTTCGAGCACTTGGGACGCCACCAGCGTCACAACCGTCC

GCTGA

>Bucl4_Bm_strain_11.seq Reverse Complement DNA Sequence Untitled Seq #4(1,1032)

ATGACGACCTACGACATCTTTGCGCTGCAGCACCTGGGCGACGAATTCATCCGCTTCATCGTGCTGCTCGCGCTGTCGA

GCGAGCGGCTGCTCGTGCTGATGGCGATCCTGCCGGCGACCGCCGACAACGTGATGAAAGGCCCGATGCGCAGCGGCGC

GGCGGCGGTGTGGTGCCTGTTCGTCGCGGGCGGACAGCAGGCGCTGATTCCGCAGCTCCACGGCGCGTTCCTGATGATC

GCCTGCGTGAAGGAGGCCGTGATCGGGCTCGTGCTCGGCATCGCGGCATCGACGGTATTCTGGGCCGCCGAGGCGATCG

GCACGTATCTCGACGACGTCGCCGGCTTCAACAACGTGCAGATGCAGAACCCGAGCTCGGGCGCGCAGACCTCGCTGAT

GGCGACGCTGTTCGGGCAACTGACGATCGCGTCGTTCTGGCTGCTGGGCGGCATGACGTTCCTGCTCGGCGCGCTGTAC

GAATCGTTCGCCTGGTGGCCGTTCGGATCGTTCGATCCGGTGCCCGCGCCGCTGCTCGAGATGTTCGCGCAGGCGCGCA

TCGACATGCTGATGAACATGATCGCGCGGATCGCGACGCCGATGATGGCGATGCTGTTGCTCGTCGACCTGGGCCTCGC

GTTCGTCGCGCGATCGGCGCAGAAACTCGATCTGATGTCGGTCAGCCAGCCGCTGAAAGGCGCGATCGCGGTGCTGATC

GTCGCGGTGCTGGCGGGGAATTTCGTGAGCGAGATGCGCGGGCAGATTTCGCTCTCGGGACTGGCGCAGCAGATCGGGC

GACTCGCGCGGCAGCCGGCGGGTGGGGTTTCGGGGGTTTCGGGGGTTTCGGGGGTTTCGGGGGTTTCGGGGGTTTCGGG

GGTTTCGGGGGTTTCGGGGGTTTCGGGTGCTTCGGGTGCTTCGGGTGCTTCGGGCGCTTCGGGCGCTTCGGGCGCTTCG

GGCGCTTCGGGCGCTTCGGGCGCTTCGGGTACGTCAAGCACTTCGAGCACTTGGGACGCCACCAGCGTCACAACCGTCC

GCTGA

>A2S1C1_BURM9_Bm_NCTC_10229.seq Reverse Complement DNA Sequence Untitled Seq #1(1,987)

ATGACGACCTACGACATCTTTGCGCTGCAGCACCTGGGCGACGAATTCATCCGCTTCATCGTGCTGCTCGCGCTGTCGA

GCGAGCGGCTGCTCGTGCTGATGGCGATCCTGCCGGCGACCGCCGACAACGTGATGAAAGGCCCGATGCGCAGCGGCGC

GGCGGCGGTGTGGTGCCTGTTCGTCGCGGGCGGACAGCAGGCGCTGATTCCGCAGCTCCACGGCGCGTTCCTGATGATC

GCCTGCGTGAAGGAGGCCGTGATCGGGCTCGTGCTCGGCATCGCGGCATCGACGGTATTCTGGGCCGCCGAGGCGATCG

GCACGTATCTCGACGACGTCGCCGGCTTCAACAACGTGCAGATGCAGAACCCGAGCTCGGGCGCGCAGACCTCGCTGAT

GGCGACGCTGTTCGGGCAACTGACGATCGCGTCGTTCTGGCTGCTGGGCGGCATGACGTTCCTGCTCGGCGCGCTGTAC

GAATCGTTCGCCTGGTGGCCGTTCGGATCGTTCGATCCGGTGCCCGCGCCGCTGCTCGAGATGTTCGCGCAGGCGCGCA

TCGACATGCTGATGAACATGATCGCGCGGATCGCGACGCCGATGATGGCGATGCTGTTGCTCGTCGACCTGGGCCTCGC

GTTCGTCGCGCGATCGGCGCAGAAACTCGATCTGATGTCGGTCAGCCAGCCGCTGAAAGGCGCGATCGCGGTGCTGATC

GTCGCGGTGCTGGCGGGGAATTTCGTGAGCGAGATGCGCGGGCAGATTTCGCTCTCGGGACTGGCGCAGCAGATCGGGC

GACTCGCGCGGCAGCCGGCGGGTGGGGTTTCGGGGGTTTCGGGGGTTTCGGGGGTTTCGGGTGCTTCGGGTGCTTCGGG

TGCTTCGGGCGCTTCGGGCGCTTCGGGCGCTTCGGGCGCTTCGGGCGCTTCGGGCGCTTCGGGTACGTCAAGCACTTCG

AGCACTTGGGACGCCACCAGCGTCACAACCGTCCGCTGA

>Bucl4_Bm_NCTC_10247.seq Reverse Complement DNA Sequence Untitled Seq #12(1,1095)

ATGACGACCTACGACATCTTTGCGCTGCAGCACCTGGGCGACGAATTCATCCGCTTCATCGTGCTGCTCGCGCTGTCGA

GCGAGCGGCTGCTCGTGCTGATGGCGATCCTGCCGGCGACCGCCGACAACGTGATGAAAGGCCCGATGCGCAGCGGCGC

GGCGGCGGTGTGGTGCCTGTTCGTCGCGGGCGGACAGCAGGCGCTGATTCCGCAGCTCCACGGCGCGTTCCTGATGATC

GCCTGCGTGAAGGAGGCCGTGATCGGGCTCGTGCTCGGCATCGCGGCATCGACGGTATTCTGGGCCGCCGAGGCGATCG

GCACGTATCTCGACGACGTCGCCGGCTTCAACAACGTGCAGATGCAGAACCCGAGCTCGGGCGCGCAGACCTCGCTGAT

GGCGACGCTGTTCGGGCAACTGACGATCGCGTCGTTCTGGCTGCTGGGCGGCATGACGTTCCTGCTCGGCGCGCTGTAC

GAATCGTTCGCCTGGTGGCCGTTCGGATCGTTCGATCCGGTGCCCGCGCCGCTGCTCGAGATGTTCGCGCAGGCGCGCA

TCGACATGCTGATGAACATGATCGCGCGGATCGCGACGCCGATGATGGCGATGCTGTTGCTCGTCGACCTGGGCCTCGC

GTTCGTCGCGCGATCGGCGCAGAAACTCGATCTGATGTCGGTCAGCCAGCCGCTGAAAGGCGCGATCGCGGTGCTGATC

GTCGCGGTGCTGGCGGGGAATTTCGTGAGCGAGATGCGCGGGCAGATTTCGCTCTCGGGACTGGCGCAGCAGATCGGGC

GACTCGCGCGGCAGCCGGCGGGTGGGGTTTCGGGGGTTTCGGGGGTTTCGGGGGTTTCGGGGGTTTCGGGGGTTTCGGG

GGTTTCGGGGGTTTCGGGGGTTTCGGGGGTTTCGGGGGTTTCGGGGGTTTCGGGGGTTTCGGGGGTTTCGGGGGTTTCG

GGGGTTTCGGGTGCTTCGGGTGCTTCGGGTGCTTCGGGCGCTTCGGGCGCTTCGGGCGCTTCGGGCGCTTCGGGCGCTT

CGGGCGCTTCGGGTACGTCAAGCACTTCGAGCACTTGGGACGCCACCAGCGTCACAACCGTCCGCTGA

>Bucl4_Bm_FMH.seq Created: Monday, March 24, 2014 5:31 PM

ATGACGACCTACGACATCTTTGCGCTGCAGCACCTGGGCGACGAATTCATCCGCTTCATCGTGCTGCTCGCGCTGTCGA

GCGAGCGGCTGCTCGTGCTGATGGCGATCCTGCCGGCGACCGCCGACAACGTGATGAAAGGCCCGATGCGCAGCGGCGC

GGCGGCGGTGTGGTGCCTGTTCGTCGCGGGCGGACAGCAGGCGCTGATTCCGCAGCTCCACGGCGCGTTCCTGATGATC

GCCTGCGTGAAGGAGGCCGTGATCGGGCTCGTGCTCGGCATCGCGGCATCGACGGTATTCTGGGCCGCCGAGGCGATCG

GCACGTATCTCGACGACGTCGCCGGCTTCAACAACGTGCAGATGCAGAACCCGAGCTCGGGCGCGCAGACCTCGCTGAT

GGCGACGCTGTTCGGGCAACTGACGATCGCGTCGTTCTGGCTGCTGGGCGGCATGACGTTCCTGCTCGGCGCGCTGTAC

GAATCGTTCGCCTGGTGGCCGTTCGGATCGTTCGATCCGGTGCCCGCGCCGCTGCTCGAGATGTTCGCGCAGGCGCGCA

TCGACATGCTGATGAACATGATCGCGCGGATCGCGACGCCGATGATGGCGATGCTGTTGCTCGTCGACCTGGGCCTCGC

GTTCGTCGCGCGATCGGCGCAGAAACTCGATCTGATGTCGGTCAGCCAGCCGCTGAAAGGCGCGATCGCGGTGCTGATC

GTCGCGGTGCTGGCGGGGAATTTCGTGAGCGAGATGCGCGGGCAGATTTCGCTCTCGGGACTGGCGCAGCAGATCGGGC

GACTCGCGCGGCAGCCGGCGGGTGGGGTTTCGGGGGTTTCGGGGGTTTCGGGGGTTTCGGGGGTTTCGGGGGTTTCGGG

TGCTTCGGGTGCTTCGGGTGCTTCGGGCGCTTCGGGCGCTTCGGGCGCTTCGGGTGCTTCGGGCGCTTCGGGCGCTTCG

GGTACGTCAAGCACTTCGAGCACTTGGGACGCCACCAGCGTCACAACCGTCCGCTGA

>A5XLG9_BURMA_Bm_JHU.seq Created: Wednesday, April 10, 2013 3:08 PM

ATGACGACCTACGACATCTTTGCGCTGCAGCACCTGGGCGACGAATTCATCCGCTTCATCGTGCTGCTCGCGCTGTCGA

GCGAGCGGCTGCTCGTGCTGATGGCGATCCTGCCGGCGACCGCCGACAACGTGATGAAAGGCCCGATGCGCAGCGGCGC

GGCGGCGGTGTGGTGCCTGTTCGTCGCGGGCGGACAGCAGGCGCTGATTCCGCAGCTCCACGGCGCGTTCCTGATGATC

GCCTGCGTGAAGGAGGCCGTGATCGGGCTCGTGCTCGGCATCGCGGCATCGACGGTATTCTGGGCCGCCGAGGCGATCG

GCACGTATCTCGACGACGTCGCCGGCTTCAACAACGTGCAGATGCAGAACCCGAGCTCGGGCGCGCAGACCTCGCTGAT

GGCGACGCTGTTCGGGCAACTGACGATCGCGTCGTTCTGGCTGCTGGGCGGCATGACGTTCCTGCTCGGCGCGCTGTAC

GAATCGTTCGCCTGGTGGCCGTTCGGATCGTTCGATCCGGTGCCCGCGCCGCTGCTCGAGATGTTCGCGCAGGCGCGCA

TCGACATGCTGATGAACATGATCGCGCGGATCGCGACGCCGATGATGGCGATGCTGTTGCTCGTCGACCTGGGCCTCGC

GTTCGTCGCGCGATCGGCGCAGAAACTCGATCTGATGTCGGTCAGCCAGCCGCTGAAAGGCGCGATCGCGGTGCTGATC

GTCGCGGTGCTGGCGGGGAATTTCGTGAGCGAGATGCGCGGGCAGATTTCGCTCTCGGGACTGGCGCAGCAGATCGGGC

GACTCGCGCGGCAGCCGGCGGGTGGGGTTTCGGGGGTTTCGGGGGTTTCGGGGGTTTCGGGGGTTTCGGGGGTTTCGGG

TGCTTCGGGTGCTTCGGGTGCTTCGGGCGCTTCGGGCGCTTCGGGCGCTTCGGGTGCTTCGGGCGCTTCGGGCGCTTCG

GGTACGTCAAGCACTTCGAGCACTTGGGACGCCACCAGCGTCACAACCGTCCGCTGA

>A9K4R2_BURMA_Bm_ATCC_10399.seq Reverse Complement DNA Sequence Untitled Seq #2(1,1023)

ATGACGACCTACGACATCTTTGCGCTGCAGCACCTGGGCGACGAATTCATCCGCTTCATCGTGCTGCTCGCGCTGTCGA

GCGAGCGGCTGCTCGTGCTGATGGCGATCCTGCCGGCGACCGCCGACAACGTGATGAAAGGCCCGATGCGCAGCGGCGC

GGCGGCGGTGTGGTGCCTGTTCGTCGCGGGCGGACAGCAGGCGCTGATTCCGCAGCTCCACGGCGCGTTCCTGATGATC

GCCTGCGTGAAGGAGGCCGTGATCGGGCTCGTGCTCGGCATCGCGGCATCGACGGTATTCTGGGCCGCCGAGGCGATCG

GCACGTATCTCGACGACGTCGCCGGCTTCAACAACGTGCAGATGCAGAACCCGAGCTCGGGCGCGCAGACCTCGCTGAT

GGCGACGCTGTTCGGGCAACTGACGATCGCGTCGTTCTGGCTGCTGGGCGGCATGACGTTCCTGCTCGGCGCGCTGTAC

GAATCGTTCGCCTGGTGGCCGTTCGGATCGTTCGATCCGGTGCCCGCGCCGCTGCTCGAGATGTTCGCGCAGGCGCGCA

TCGACATGCTGATGAACATGATCGCGCGGATCGCGACGCCGATGATGGCGATGCTGTTGCTCGTCGACCTGGGCCTCGC

GTTCGTCGCGCGATCGGCGCAGAAACTCGATCTGATGTCGGTCAGCCAGCCGCTGAAAGGCGCGATCGCGGTGCTGATC

GTCGCGGTGCTGGCGGGGAATTTCGTGAGCGAGATGCGCGGGCAGATTTCGCTCTCGGGACTGGCGCAGCAGATCGGGC

GACTCGCGCGGCAGCCGGCGGGTGGGGTTTCGGGGGTTTCGGGGGTTTCGGGGGTTTCGGGGGTTTCGGGGGTTTCGGG

GGTTTCGGGGGTTTCGGGTGCTTCGGGTGCTTCGGGTGCTTCGGGCGCTTCGGGCGCTTCGGGCGCTTCGGGTGCTTCG

GGCGCTTCGGGCGCTTCGGGTACGTCAAGCACTTCGAGCACTTGGGACGCCACCAGCGTCACAACCGTCCGCTGA

>C4AZL9_BURMA_Bm_GB8_horse_4.seq Created: Wednesday, April 10, 2013 3:59 PM

ATGACGACCTACGACATCTTTGCGCTGCAGCACCTGGGCGACGAATTCATCCGCTTCATCGTGCTGCTCGCGCTGTCGA

GCGAGCGGCTGCTCGTGCTGATGGCGATCCTGCCGGCGACCGCCGACAACGTGATGAAAGGCCCGATGCGCAGCGGCGC

GGCGGCGGTGTGGTGCCTGTTCGTCGCGGGCGGACAGCAGGCGCTGATTCCGCAGCTCCACGGCGCGTTCCTGATGATC

GCCTGCGTGAAGGAGGCCGTGATCGGGCTCGTGCTCGGCATCGCGGCATCGACGGTATTCTGGGCCGCCGAGGCGATCG

GCACGTATCTCGACGACGTCGCCGGCTTCAACAACGTGCAGATGCAGAACCCGAGCTCGGGCGCGCAGACCTCGCTGAT

GGCGACGCTGTTCGGGCAACTGACGATCGCGTCGTTCTGGCTGCTGGGCGGCATGACGTTCCTGCTCGGCGCGCTGTAC

GAATCGTTCGCCTGGTGGCCGTTCGGATCGTTCGATCCGGTGCCCGCGCCGCTGCTCGAGATGTTCGCGCAGGCGCGCA

TCGACATGCTGATGAACATGATCGCGCGGATCGCGACGCCGATGATGGCGATGCTGTTGCTCGTCGACCTGGGCCTCGC

GTTCGTCGCGCGATCGGCGCAGAAACTCGATCTGATGTCGGTCAGCCAGCCGCTGAAAGGCGCGATCGCGGTGCTGATC

GTCGCGGTGCTGGCGGGGAATTTCGTGAGCGAGATGCGCGGGCAGATTTCGCTCTCGGGACTGGCGCAGCAGATCGGGC

GACTCGCGCGGCAGCCGGCGGGTGGGGTTTCGGGGGTTTCGGGGGTTTCGGGGGTTTCGGGGGTTTCGGGGGTTTCGGG

TGCTTCGGGTGCTTCGGGTGCTTCGGGCGCTTCGGGCGCTTCGGGCGCTTCGGGTGCTTCGGGCGCTTCGGGCGCTTCG

GGTACGTCAAGCACTTCGAGCACTTGGGACGCCACCAGCGTCACAACCGTCCGCTGA

>Q62AR6_BURMA_Bm_ATCC_23344.seq Created: Wednesday, April 10, 2013 4:39 PM

ATGACGACCTACGACATCTTTGCGCTGCAGCACCTGGGCGACGAATTCATCCGCTTCATCGTGCTGCTCGCGCTGTCGA

GCGAGCGGCTGCTCGTGCTGATGGCGATCCTGCCGGCGACCGCCGACAACGTGATGAAAGGCCCGATGCGCAGCGGCGC

GGCGGCGGTGTGGTGCCTGTTCGTCGCGGGCGGACAGCAGGCGCTGATTCCGCAGCTCCACGGCGCGTTCCTGATGATC

GCCTGCGTGAAGGAGGCCGTGATCGGGCTCGTGCTCGGCATCGCGGCATCGACGGTATTCTGGGCCGCCGAGGCGATCG

GCACGTATCTCGACGACGTCGCCGGCTTCAACAACGTGCAGATGCAGAACCCGAGCTCGGGCGCGCAGACCTCGCTGAT

GGCGACGCTGTTCGGGCAACTGACGATCGCGTCGTTCTGGCTGCTGGGCGGCATGACGTTCCTGCTCGGCGCGCTGTAC

GAATCGTTCGCCTGGTGGCCGTTCGGATCGTTCGATCCGGTGCCCGCGCCGCTGCTCGAGATGTTCGCGCAGGCGCGCA

TCGACATGCTGATGAACATGATCGCGCGGATCGCGACGCCGATGATGGCGATGCTGTTGCTCGTCGACCTGGGCCTCGC

GTTCGTCGCGCGATCGGCGCAGAAACTCGATCTGATGTCGGTCAGCCAGCCGCTGAAAGGCGCGATCGCGGTGCTGATC

GTCGCGGTGCTGGCGGGGAATTTCGTGAGCGAGATGCGCGGGCAGATTTCGCTCTCGGGACTGGCGCAGCAGATCGGGC

GACTCGCGCGGCAGCCGGCGGGTGGGGTTTCGGGGGTTTCGGGGGTTTCGGGGGTTTCGGGGGTTTCGGGGGTTTCGGG

TGCTTCGGGTGCTTCGGGTGCTTCGGGCGCTTCGGGCGCTTCGGGCGCTTCGGGTGCTTCGGGCGCTTCGGGCGCTTCG

GGTACGTCAAGCACTTCGAGCACTTGGGACGCCACCAGCGTCACAACCGTCCGCTGA

>Bucl4_Bt_E264.seq Reverse Complement DNA Sequence Untitled Seq #1(1,987)

ATGAACACCTACGACATCTTTGCGCTTCAGCACCTGGGCGACGAATTCATCGGCTTCATCGTGCTGCTCGCGCTGTCGA

GCGAGCGGCTGCTCGTGATCATGTCGATCCTGCCCGCGACCGCCGACACCGTGCTGAAAGGCCCGATGCGCAGCGGCGC

GGCGGCGCTCTGGTGCCTGTTCGTCGCGTGCGGGCAGCAGGCGCTGATTCCGCAGCTTCACGGCGCGTTTCTCGTGATC

GCATGCTTGAAGGAGGCCGTGATCGGGCTTGTGATCGGCATTGCGGCGTCGACGGTGTTCTGGGCCGCCGAGGCGATCG

GCACGTATGTCGACGACGTCGCCGGCTTCAACAACCTGCAGATGCAGAATCCGAGTTCGGGCACGCAGACGTCGCTGAT

GGCGACGCTGTTCGGGCAACTGACGATTGCGTCGTTCTGGCTGCTGGGCGGCATGACGTTCCTGCTCGGCGCGCTGTAC

GAATCGTACGCATGGTGGCCGCTCGGCTCGTTCGATCCGGTTCCCGCGCCGCTCCTCGAGATGTTCGCGCAGGCGCGGC

TCGACATGCTGATGAACATGGTCGCGCGGATCGCGACGCCGATGATGACGATGCTGGTGCTCGTCGACCTGGGCCTCGC

GTTCGTCGCGCGATCGGCGCAGAAGCTCGATCTGATGTCGGTCAGCCAGCCGCTCAAGGGCGCGATCGCGGTGCTGATC

GTCGCGGTGCTGGCGGGCAATTTCGTGAACGAGATGCGCGGGCAGATTTCGCTCGCGGAACTGGCGCAGCAGGTCAGAC

GGCTTGCGGAGCGGCCGGGACGCGATGGGAAGACGGATGGCGGCGGCGGGGCTTCGGGGGCTTCAGGCGTTTCGGGCGT

TTCGGGCGTTTCGGGCGTTTCGGGCGTTTCGGGCGTTTCGGGCGTTTCGGGCGTTTCGGGCGTTTCGGGCGTTTCGGGC

GTTTCGGGCGTTTCGGGCGCGACCAGTGTCACATCCATCCGCTGA

>Bucl4_Bt_MSMB121.seq Created: Friday, November 22, 2013 6:32 PM

ATGAACACCAGCGACATCTTTGCGCTGCAGCACCTGGGCGACGAACTCATCGGCTTCATCGTGCTGCTCGCGCTGTCGA

GCGAGCGGCTGCTCGTGATCATGTCGATCCTGCCCGCGACCGCCGACACCGTGATGAAAGGCCCGATGCGCAGCGGCGC

GGCGGCGGTGTGGTGCCTGTTCATCGCGTGCGGGCAGCAGGCGCTGATTCCGCAGCTTCACGGCGCGTTTCTCGTGATC

GCCTGCTTGAAGGAGGCCGTGATCGGGCTCGTGCTCGGCATCGCCGCGTCGACGGTGTTCTGGGCCGCAGAGGCGATCG

GCACGTATGTCGACGACGTCGCCGGCTTCAACAACGTGCAGATGCAGAACCCGAGCTCGGGCACGCAGACATCGCTGAT

GGCGACGCTGTTCGGGCAACTGACGATCGCGTCGTTCTGGCTGCTGGGCGGCATGACGTTCCTGCTCGGCGCGCTGGTC

GAATCGTATGCATGGTGGCCGCTCGGGTCGTTCGATCCGGTTCCCGCGCCGCTGCTCGAAGCATTCGCGCAAGCGCGGA

TCGACATGCTGATGAACATGATCGCGCGGATCGCGACGCCGATGATGGCGATGCTGGTGCTCGTCGACCTGGGCCTCGC

GTTCGTCGCGCGGTCGGCGCAGAAGCTCGATCTGATGTCGGTCGGCCAGCCGCTCAAGGGCGCGATCGCAGTGCTGATG

GTCGCGGTGCTGGCGGGGAATTTCGTGACCGAGATGCGCGGGCAGATTTCGCTTGCGGAGCTGGCGCAGCAGGTCCGGC

GGCTCGCGCAGCGGCCGGGGGGCGATGGGAAGACGGATCGAGGGGGCCGCGCTTCGGGGGCTTCGGGGGCTTCGGGGGC

GACCAGCGTCACATCCGTTCGCTGA

>Bucl4_Bt_2002721723.seq Reverse Complement DNA Sequence Untitled Seq #13(1,993)

ATGAACACCTACGACATCTTTGCGCTTCAGCACCTGGGCGACGAATTCATCGGCTTCATCGTGCTGCTCGCGCTGTCGA

GCGAGCGGCTGCTCGTGATCATGTCGATCCTGCCCGCGACCGCCGACACCGTGCTGAAAGGCCCGATGCGCAGCGGCGC

GGCGGCGCTCTGGTGCCTGTTCGTCGCGTGCGGGCAGCAGGCGCTGATTCCGCAGCTTCACGGCGCGTTTCTCGTGATC

GCATGCTTGAAGGAGGCCGTGATCGGGCTTGTGATCGGCATTGCGGCGTCGACGGTGTTCTGGGCCGCCGAGGCGATCG

GCACGTATGTCGACGACGTCGCCGGCTTCAACAACCTGCAGATGCAGAATCCGAGTTCGGGCACGCAGACGTCGCTGAT

GGCGACGCTGTTCGGGCAACTGACGATTGCGTCGTTCTGGCTGCTGGGCGGCATGACGTTCCTGCTCGGCGCGCTGTAC

GAATCGTACGCATGGTGGCCGCTCGGCTCGTTCGATCCGGTTCCCGCGCCGCTCCTCGAGATGTTCGCGCAGGCGCGGC

TCGACATGCTGATGAACATGGTCGCGCGGATCGCGACGCCGATGATGACGATGCTGGTGCTCGTCGACCTGGGCCTCGC

GTTCGTCGCGCGATCGGCGCAGAAGCTCGATCTGATGTCGGTCAGCCAGCCGCTCAAGGGCGCGATCGCGGTGCTGATC

GTCGCGGTGCTGGCGGGCAATTTCGTGAACGAGATGCGCGGGCAGATTTCGCTCGCGGAACTGGCGCAGCAGGTCAGAC

GGCTTGCGGAGCGGCCGGGACGCGATGGGAAGACGGATGGCGGCGGCGGGGCTTCGGGGGCTTCAGGCGTTTCGGGCGT

TTCGGGCGTTTCGGGCGTTTCGGGCGTTTCGGGCGTTTCGGGCGTTTCGGGCGTTTCGGGCGTTTCGGGCGTTTCGGGC

GTTTCGGGCGTTTCGGGCGCGACCAGTGTCACATCCATCCGCTGA

>Bucl4_Bt_E444.seq Reverse Complement DNA Sequence Untitled Seq #19(1,975)

ATGAACACCTACGACATCTTTGCGCTTCAGCACCTGGGCGACGAATTCATCGGCTTCATCGTGCTGCTCGCGCTGTCGA

GCGAGCGGCTGCTCGTGATCATGTCGATCCTGCCCGCGACCGCCGACACCGTGCTGAAAGGCCCGATGCGCAGCGGCGC

GGCGGCGCTCTGGTGCCTGTTCGTCGCGTGCGGGCAGCAGGCGCTGATTCCGCAGCTTCACGGCGCGTTTCTCGTGATC

GCATGCTTGAAGGAGGCCGTGATCGGGCTTGTGATCGGCATTGCGGCGTCGACGGTGTTCTGGGCCGCCGAGGCGATCG

GCACGTATGTCGACGACGTCGCCGGCTTCAACAACCTGCAGATGCAGAATCCGAGTTCGGGCACGCAGACGTCGCTGAT

GGCGACGCTGTTCGGGCAACTGACGATTGCGTCGTTCTGGCTGCTGGGCGGCATGACGTTCCTGCTCGGCGCGCTGTAC

GAATCGTACGCATGGTGGCCGCTCGGCTCGTTCGATCCGGTTCCCGCGCCGCTCCTCGAGATGTTCGCGCAGGCGCGGC

TCGACATGCTGATGAACATGGTCGCGCGGATCGCGACGCCGATGATGACGATGCTGGTGCTCGTCGACCTGGGCCTCGC

GTTCGTCGCGCGATCGGCGCAGAAGCTCGATCTGATGTCGGTCAGCCAGCCGCTCAAGGGCGCGATCGCGGTGCTGATC

GTCGCGGTGCTGGCGGGCAATTTCGTGAACGAGATGCGCGGGCAGATTTCGCTCGCGGAACTGGCGCAGCAGGTCAGAC

GGCTTGCGGAGCGGCCGGGACGCGATGGGAAGACGGATGGCGGCGGCGGGGCTTCGGGGGCTTCAGGCGTTTCGGGCGT

TTCGGGCGTTTCGGGCGTTTCGGGCGTTTCGGGCGTTTCGGGCGTTTCGGGCGTTTCGGGCGTTTCGGGCGTTTCGGGC

GCGACCAGTGTCACATCCATCCGCTGA

>Bucl4_Bt_H0587.seq Reverse Complement DNA Sequence Untitled Seq #16(1,948)

ATGAACACCTACGACATCTTTGCGCTGCAGCACCTGGGCGACGAATTCATCGGCTTCATCGTGCTGCTCGCGCTGTCGA

GCGAGCGGCTGCTCGTGATCATGTCGATCCTGCCCGCGACCGCCGACACCGTGCTGAAAGGCCCGATGCGCAGCGGCGC

GGCGGCGCTCTGGTGCCTGTTCGTCGCGTGCGGGCAGCAGGCGCTGATTCCGCAGCTTCACGGCGCGTTTCTCGTGATC

GCATGCTTGAAGGAGGCCGTGATCGGGCTCGTGATCGGCATTGCGGCGTCGACGGTGTTCTGGGCCGCCGAGGCGATCG

GCACGTATGTCGACGACGTCGCCGGCTTCAACAACCTGCAGATGCAGAATCCGAGTTCGGGCACGCAGACGTCGCTGAT

GGCGACGCTGTTCGGGCAACTGACGATCGCGTCGTTCTGGCTGCTGGGCGGCATGACGTTCCTGCTCGGCGCGCTGTAC

GAATCGTACGCATGGTGGCCGCTCGGCTCGTTCGATCCGGTTCCCGCGCCGCTCCTCGAGATGTTCGCGCAGGCGCGGC

TCGACATGCTGATGAACATGGTCGCGCGGATCGCGACGCCGATGATGACGATGCTGGTGCTCGTCGACCTGGGCCTCGC

GTTCGTCGCGCGATCGGCGCAGAAGCTCGATCTGATGTCGGTCAGCCAGCCGCTCAAGGGCGCGATCGCGGTGCTGATC

GTCGCGGTGCTGGCGGGCAATTTCGTGAACGAGATGCGCGGGCAGATTTCGCTCGCGGAACTGGCGCAGCAGGTCAGAC

GGCTTGCGGAGCGGCCGGGACGCGATGGGAAGACGGATGGCGGCGGCGGGGCTTCGGGGGCTTCGGGGGCTTCGGGCGT

TTCGGGCGTTTCGGGCGTTTCGGGCGTTTCGGGCGTTTCGGGCGTTTCGGGCGCGACCAGCGTCACATCCATCCGCTGA

>Bucl5_Bps_K96243.seq Created: Sunday, November 24, 2013 10:19 AM

ATGGGCTATGGGTGGACTATCGATGGGTTTTCGATGAGGCTCTCGATGGGTTCGGGTTCGGGTTCGATGAACTCGATGA

GTCAGATGAGTTCGATGGACTTGAGACTCGAAGGACTCGAAGGACTCGAAGGACTCGAAGGACCCGAAGGACTCGAAGG

ACCCGAAGGACCCGAAGGACCCGAAGGACCCGAAGGACTCGAAGGACTCGAAGGACTCGAAGGACTCGAAGGACTCGAA

GGACTCGAAGGACTCGAAGGACTCGATGGATTCGATGGATTCGACGAACTCGACGAACTCAACGCGAATCGACCACGCC

GCTCGCGAAGCGGCGCCCACGCTCGCGTTCGGCATCGGGGCGGCGAGCGTGCCGATGCGGCGGCACGCTCGCCGCGCGC

GGACGTCGTCGGCCTGTCCGGCAGGTCGGCGCCGGAGGCGCGTGCGCAACGTCATCCTCCCAGCGCGCCGCGCCGCTTC

CAGAGCAGCGCTAGAAAGAACGGCGCGCCGATCAGCGCGGTCAGGATGCCGAGCGGAATCTCGGCCGGCGCGGCGAGCG

TGCGCGCGGCGAGATCGGCGGCCACCGCGAGCAGCGCGCCGAAAAGCGCCGCGCCCGGCATCACGATGCGCTGA

>Bucl5_Bps_MSHR305.seq Created: Sunday, November 24, 2013 10:43 AM

ATGGGCTATGGGTGGACTATCGATGGGTTTTCGATGAGGCTCTCGATGGGTTCGGGTTCGGGTTCGGGTTCGGGGTCGA

TGAACTCGATGAGTCAGATGAGTTCGATGGACTTGAGACTCGAAGGACTCGAAGGACTCGAAGGACTCGAAGGACTCGA

AGGACCCGAAGGACTCGAAGGACTCGAAGGACCCGAAGGACCCGAAGGACCCGAAGGACCCGAAGGACTCGAAGGACTC

GATGGATTCGATGAACTCGACGAACTCAACGCGAATCGACCACGCCGCTCGCGAAGCGGCGCCCACGCTCGCGTTCGGC

ATCGCGGCGGCGAGCGTGCCGATGCGGCGGCACGCTCGCCGCGCGCGAACGTCGTCGGCCTGTCCGGCAGGTCGGCGCC

GGAGGCGCGTGCGCAACGTCATCCTCCCAGCGCGCCGCGCCGCTTCCATAGCAGCGCTAGAAAGAACGGCGCGCCGATC

AGCGCGGTCAGGATGCCGAGCGGAATCTCGGCCGGCGCGGCGAGCGTGCGCGCGGCGAGATCGGCGGCCACCGCGAGCA

GCGCGCCGAAAAGCGCCGCGCCCGGCATCACGATGCGCTGA

>Bucl5_Bps_1106a.seq Created: Tuesday, December 31, 2013 10:23 AM

atgggctatgggtggactatcgatgggttttcgatgaggctctcgatgggttcgggttcgggttcgatgaactcgatga

gtcagatgagttcgatggacttgagactcgaaggactcgaaggactcgaaggacccgaaggacccgaaggacccgaagg

acccgaaggacccgaaggactcgatggactcgatggactcgatggactcgatggattcgatgaactcgacgaactcaac

gcgaatcgaccacgccgctcgcgaaacggcgcccacgctcgcgttcggcatcgcggcggcgagcgtgccgatgcggcgg

cacgctcgccgcgcgcgaacgtcgtcggcctgtccggcaggtcggcgccgaaggcgcgtgcgcaacgtcatcctcccag

cgcgccgcgccgcttccatagcagcgctagaaagaacggcgcgccgatcagcgcggtcaggatgccgagcggaatctcg

gccggcgcggcgagcgtgcgcgcggcgagatcggcggccaccgcgagcagcgcgccgaaaagcgccgcgcccggcatca

cgatgcgctga

>Bucl5_Bps_1026b.seq Created: Sunday, November 24, 2013 10:34 AM

ATGGACTATGGGTGGACTATCGATGGGTTTTCGATGAGGCTCTCGATGGGTTCGGGTTCGATGAACTCGATGAACTCGA

TGAGTCAGATGAGTTCGATGGACTTGAGACTCGAAGGACTCGAAGGACTCGAAGGACTCGAAGGACTCGAAGGACTCGA

AGGACTCGAAGGACTCGAAGGACTCGAAGGACCCGAAGGACCCGAAGGACCCGAAGGACTCGAAGGACTCGAAGGACCC

GAAGGACTCGAAGGACTCGAAGGACCCGAAGGACTCGAAGGACTCGAAGGACTCGATGGATTCGATGAACTCGACGAAC

TCAACGCGAATCGACCACGCCGCTCGCGAAGCGGCGCCCACGCTCGCGTTCGGCATCGCGGCGGCGAGCGTGCCGATGC

GGCGGCACGCTCGCCGCGCGCGAACGTCGTCGGCCTGTCCGGCAGGTCGGCGCCGAAGGCGCGTGCGCAACGTCATCCT

CCCAGCGCGCCGCGCCGCTTCCATAGCAGCGCTAGAAAGAACGGCGCGCCGATCAGCGCGGTCAGGATGCCGAGCGGAA

TCTCGGCCGGCGCGGCGAGCGTGCGCGCGGCGAGATCGGCGGCCACCGCGAGCAGCGCGCCGAAAAGCGCCGCGCCCGG

CATCACGATGCGCTGA

>Q3JHM0_BURP1_Bucl5_Bp_1710b.seq Created: Tuesday, October 29, 2013 6:37 PM

ATGGGCTATGGGTGGACTATCGATGGGTTTTCGATGAGGCTCTCGATGGGTTCGGGTTCGGGTTCGATGAACTCGATGA

GTCAGATGAGTTCGATGGACTTGAGACTCGAAGGACTCGAAGGACTCGAAGGACTCGAAGGACTCGAAGGACTCGAAGG

ACTCGAAGGACCCGAAGGACTCGAAGGACCCGAAGGACCCGAAGGACCCGAAGGACTCGAAGGACCCGAAGGACTCGAA

GGACTCGAAGGACTCGAAGGACTCGATGGATTCGATGGATTCGACGAACTCGACGAACTCAACGCGAATCGACCACGCC

GCTCGCGAAGCGGCGCCCACGCTCGCGTTCGGCATCGGGGCGGCGAGCGTGCCGATGCGGCGGCACGCTCGCCGCGCGC

GGACGTCGTCGGCCTGTCCGGCAGGTCGGCGCCGGAGGCGCGTGCGCAACGTCATCCTCCCAGCGCGCCGCGCCGCTTC

CAGAGCAGCGCTAGAAAGAACGGCGCGCCGATCAGCGCGGTCAGGATGCCGAGCGGAATCTCGGCCGGCGCGGCGAGCG

TGCGCGCGGCGAGATCGGCGGCCACCGCGAGCAGCGCGCCGAAAAGCGCCGCGCCCGGCATCACGATGCGCTGA

>Bucl5_Bps_BPC006.seq Created: Tuesday, December 31, 2013 3:04 PM

atgggctatgggtggactatcgatgggttttcgatgaggctctcgatgggttcgggttcgggttcgggttatgaactcg

atgaactcgatgagtcagatgagttcgatggacttgagactcgaaggactcgaaggactcgaaggacccgaaggacccg

aaggacccgaaggacccgaaggacccgaaggactcgatggactcgatggactcgatggactcgatggattcgatgaact

cgacgaactcaacgcgaatcgaccacgccgctcgcgaaacggcgcccacgctcgcgttcggcatcgcggcggcgagcgt

gccgatgcggcggcacgctcgccgcgcgcgaacgtcgtcggcctgtccggcaggtcggcgccgaaggcgcgtgcgcaac

gtcatcctcccagcgcgccgcgccgcttccatagcagcgctagaaagaacggcgcgccgatcagcgcggtcaggatgcc

gagcggaatctcggccggcgcggcgagcgtgcgcgcggcgagatcggcggccaccgcgagcagcgcgccgaaaagcgcc

gcgcccggcatcacgatgcgctga

>Bucl5_Bps_668.seq Created: Monday, January 20, 2014 5:35 PM

atgggctatgggtggactatcgatgggttttcgatgaggctctcgatgggttcgggttcgggttcgatgaactcgatga

actcgatgagtcagatgagttcgatggacttgagactcgaaggacccgaaggacccgaaggacccgaaggacccgaagg

acccgaaggacccgaaggacccgaaggactcgatggactcgatggactcgaaggactcgatggattcgacgaactcgac

gaactcaacgcgaatcgaccacgccgctcgcgaagcggcgcccacgctcgcgttcggcatcggggcggcgagcgtgccg

atgcggcggcacgctcgccgcgcgcggacgtcgtcggcctgtccggcaggtcggcgccggaggcgcgtgcgcaacgtca

tcctcccagcgcgccgcgccgcttccatagcagcgctagaaagaacggcgcgccgatcagcgcggtcaggatgccgagc

ggaatctcggccggcgcggcgagcgtgcgcgcggcgagatcggcggccaccgcgagcagcgcgccgaaaagcgccgcgc

ccggcatcacgatgcgctga

>Bucl5_Bps_NCTC_13179.seq Created: Friday, January 03, 2014 9:36 AM

atgggctatgggtggactatcgatgggttttcgatgaggctctcgatgggttcgggttcgggttcgatgaactcgatga

gtcagatgagttcgatggacttgagactcgaaggactcgaaggactcgaaggactcgaaggactcgaaggactcgaagg

actcgaaggactcgatggattcgatgaactcgacgaactcaacgcgaatcgaccacgccgctcgcgaaacggcgcccac

gctcgcgttcggcatcgcggcggcgagcgtgccgatgcggcggcacgctcgccgcgcgcggacatcgtcggcctgtccg

gcaggtcggcgccggaggcgcgtgcgcaacgtcatcctcccagcgcgccgcgccgcttccagagcagcgctagaaagaa

cggcgcgccgatcagcgcggtcaggatgccgagcggaatctcggccggcgcggcgagcgtgcgcgcggcgagcgcgccg

aagagcgccgcgcccggcatcacgatgcgctga

>Bucl5_Bps_MSHR146.seq Created: Wednesday, March 19, 2014 4:38 PM

ATGGGCTATGGGTGGACTATCGATGGGTTTTCGATGAGGCTCTCGATGGGTTCGGGTTCGATGAACTCGATGAGTCAGA

TGAGTTCGATGGACTTGAGACTCGAAGGACTCGAAGGACTCGAAGGACTCGAAGGACCCGAAGGACTCGAAGGACCCGA

AGGACTCGAAGGACCCGATGGACTCGATGGATTCGATGAACTCGACGAACTCAACGCGAATCGACCACGCCGCTCGCGA

AACGGCGCCCACGCTCGCGTTCGGCATCGCGGCGGCGAGCGTGCCGATGCGGCGGCACGCTCGCCGCGCGCGGACATCG

TCGGCCTGTCCGGCAGGTCGGCGCCGGAGGCGCGTGCGCAACGTCATCCTCCCAGCGCGCCGCGCCGCTTCCAGAGCAG

CGCTAGAAAGAACGGCGCGCCGATCAGCGCGGTCAGGATGCCGAGCGGAATCTCGGCCGGCGCGGCGAGCGTGCGCGCG

GCGAGATCGGCGGCCACCGCGAGCAGCGCGCCGAAGAGCGCCGCGCCCGGCATCACGATGCGCTGA

>Bucl5_Bps_MSHR511.seq Reverse Complement DNA Sequence Untitled Seq #5(1,540)

ATGGGCTATGGGTGGACTATCGATGGGTTTTCGATGAGGCTCTCGATGGGTTCGGGTTCGATGAACTCGATGAGTCAGA

TGAGTTCGATGGACTTGAGACTCGAAGGACTCGAAGGACTCGAAGGACTCGAAGGACCCGAAGGACTCGAAGGACCCGA

AGGACTCGAAGGACCCGATGGACTCGATGGATTCGATGAACTCGACGAACTCAACGCGAATCGACCACGCCGCTCGCGA

AACGGCGCCCACGCTCGCGTTCGGCATCGCGGCGGCGAGCGTGCCGATGCGGCGGCACGCTCGCCGCGCGCGGACATCG

TCGGCCTGTCCGGCAGGTCGGCGCCGGAGGCGCGTGCGCAACGTCATCCTCCCAGCGCGCCGCGCCGCTTCCAGAGCAG

CGCTAGAAAGAACGGCGCGCCGATCAGCGCGGTCAGGATGCCGAGCGGAATCTCGGCCGGCGCGGCGAGCGTGCGCGCG

GCGAGATCGGCGGCCACCGCGAGCAGCGCGCCGAAGAGCGCCGCGCCCGGCATCACGATGCGCTGA

>Bucl5_Bps_MSHR520.seq Created: Wednesday, March 19, 2014 4:21 PM

ATGGGCTATGGGTGGACTATCGATGGGTTTTCGATGAGGCTCTCGATGGGTTCGGGTTCGGGTTCGGGTTCGGGGTCGA

TGAACTCGATGAGTCAGATGAGTTCGATGGACTTGAGACTCGAAGGACTCGAAGGACTCGAAGGACTCGAAGGACTCGA

AGGACCCGAAGGACTCGAAGGACTCGAAGGACCCGAAGGACCCGAAGGACCCGAAGGACCCGAAGGACTCGAAGGACTC

GATGGATTCGATGAACTCGACGAACTCAACGCGAATCGACCACGCCGCTCGCGAAGCGGCGCCCACGCTCGCGTTCGGC

ATCGCGGCGGCGAGCGTGCCGATGCGGCGGCACGCTCGCCGCGCGCGAACGTCGTCGGCCTGTCCGGCAGGTCGGCGCC

GGAGGCGCGTGCGCAACGTCATCCTCCCAGCGCGCCGCGCCGCTTCCATAGCAGCGCTAGAAAGAACGGCGCGCCGATC

AGCGCGGTCAGGATGCCGAGCGGAATCTCGGCCGGCGCGGCGAGCGTGCGCGCGGCGAGATCGGCGGCCACCGCGAGCA

GCGCGCCGAAAAGCGCCGCGCCCGGCATCACGATGCGCTGA

>Bucl5_Bps_NAU20B-16.seq Reverse Complement DNA Sequence Untitled Seq #8(1,540)

ATGGGCTATGGGTGGACTATCGATGGGTTTTCGATGAGGCTCTCGATGGGTTCGGGTTCGATGAACTCGATGAGTCAGA

TGAGTTCGATGGACTTGAGACTCGAAGGACTCGAAGGACTCGAAGGACTCGAAGGACCCGAAGGACTCGAAGGACCCGA

AGGACTCGAAGGACCCGATGGACTCGATGGATTCGATGAACTCGACGAACTCAACGCGAATCGACCACGCCGCTCGCGA

AACGGCGCCCACGCTCGCGTTCGGCATCGCGGCGGCGAGCGTGCCGATGCGGCGGCACGCTCGCCGCGCGCGGACATCG

TCGGCCTGTCCGGCAGGTCGGCGCCGGAGGCGCGTGCGCAACGTCATCCTCCCAGCGCGCCGCGCCGCTTCCAGAGCAG

CGCTAGAAAGAACGGCGCGCCGATCAGCGCGGTCAGGATGCCGAGCGGAATCTCGGCCGGCGCGGCGAGCGTGCGCGCG

GCGAGATCGGCGGCCACCGCGAGCAGCGCGCCGAAGAGCGCCGCGCCCGGCATCACGATGCGCTGA

>Bucl5_Bps_NCTC_13178.seq Created: Wednesday, March 19, 2014 4:57 PM

ATGGGCTATGGGTGGACTATCGATGGGTTTTCGATGAGGCTCTCGATGGGTTCGGGTTCGATGAACTCGATGAACTCGA

TGAGTCAGATGAGTTCGATGGACTTGAGACTCGAAGGACCCGAAGGACTCGATGGACTCGATGGACTCGATGGACTCGA

AGGACTCGATGGATTCGATGAACTCGACGAACTCAACGCGAATCGACCACGCCGCTCGCGAAACGGCGCCCACGCTCGC

GTTCGGCATCGCGGCGGCGAGCGTGCCGATGCGGCGGCACGCTCGCCGCGCGCGAACGTCGTCGGCCTGTCCGGCAGGT

CGGCGCCGAAGGCGCGTGCGCAACGTCATCCTCCCAGCGCGCCGCGCCGCTTCCATAGCAGCGCTAGAAAGAACGGCGC

GCCGATCAGCGCGGTCAGGATGCCGAGCGGAATCTCGGCCGGCGCGGCGAGCGTGCGCGCGGCGAGATCGGCGGCCACC

GCGAGCAGCGCGCCGAAAAGCGCCGCGCCCGGCATCACGATGCGCTGA

>Bucl5_Bm_2000031281.seq Created: Tuesday, March 25, 2014 10:39 AM

ATGGGCTATGGGTGGACTATCGATGGGTTTTCGATGAGGCTCTCGATGGGTTCGGGTTCGGGTTCGGGTTCGATGAACT

CGATGAGTCAGATGAGTTCGATGGACTTGAGACTCGAAGGACTCGAAGGACTCGAAGGACTCGAAGGACTCGAAGGACT

CGAAGGACTCGAAGGATTCGAAGGACTCGAAGGACCCGAAGGACCCGAAGGACTCGATGGACTCGATGGATTCGATGAA

CTCGACGAACTCAACGCGAATCGACCACGCCGCTCGCGAAACGGCGCCCACGCTCGCGTTCGGCATCGCGGCGGCGAGC

GTGCCGATGCGGCGGCACGCTCGCCGCGCGCGAACGTCGTCGGCCTGTCCGGCAGGTCGGCGCCGAAGGCGCGTGCGCA

ACGTCATCCTCCCAGCGCGCCGCGCCGCTTCCAGAGCAGCGCTAGAAAGAACGGCGCGCCGATCAGCGCGGTCAGGATG

CCGAGCGGAATCTCGGCCGGCGCGGCGAGCGTGCGCGCGGCGAGATCGGCGGCCACCGCGAGCAGCGCGCCGAAAAGCG

CCGCGCCCGGTATCACGATGCGCTGA

>Bucl5_Bm_2002721280.seq Reverse Complement DNA Sequence Untitled Seq #78(1,567)

ATGGGCTATGGGTGGACTATCGATGGGTTTTCGATGAGGCTCTCGATGGGTTCGGGTTCGGGTTCGGGTTCGGGTTCGA

TGAACTCGATGAGTCAGATGAGTTCGATGGACTTGAGACTCGAAGGACTCGAAGGACTCGAAGGACTCGAAGGACTCGA

AGGACTCGAAGGACTCGAAGGACCCGAAGGACCCGAAGGACTCGATGGACTCGATGGATTCGATGAACTCGACGAACTC

AACGCGAATCGACCACGCCGCTCGCGAAACGGCGCCCACGCTCGCGTTCGGCATCGCGGCGGCGAGCGTGCCGATGCGG

CGGCACGCTCGCCGCGCGCGAACGTCGTCGGCCTGTCCGGCAGGTCGGCGCCGAAGGCGCGTGCGCAACGTCATCCTCC

CAGCGCGCCGCGCCGCTTCCAGAGCAGCGCTAGAAAGAACGGCGCGCCGATCAGCGCGGTCAGGATGCCGAGCGGAATC

TCGGCCGGCGCGGCGAGCGTGCGCGCGGCGAGATCGGCGGCCACCGCGAGCAGCGCGCCGAAAAGCGCCGCGCCCGGTA

TCACGATGCGCTGA

>Bucl5_Bm_A188.seq Created: Tuesday, March 25, 2014 10:31 AM

ATGGGCTATGGGTGGACTATCGATGGGTTTTCGATGAGGCTCTCGATGGGTTCGGGTTCGGGTTCGGGTTCGATGAACT

CGATGAGTCAGATGAGTTCGATGGACTTGAGACTCGAAGGACTCGAAGGACTCGAAGGACTCGAAGGACTCGAAGGACT

CGAAGGACTCGAAGGACTCGAAGGACTCGAAGGACTCGAAGGACCCGAAGGACTCGATGGACTCGATGGATTCGATGAA

CTCGACGAACTCAACGCGAATCGACCACGCCGCTCGCGAAACGGCGCCCACGCTCGCGTTCGGCATCGCGGCGGCGAGC

GTGCCGATGCGGCGGCACGCTCGCCGCGCGCGAACGTCGTCGGCCTGTCCGGCAGGTCGGCGCCGAAGGCGCGTGCGCA

ACGTCATCCTCCCAGCGCGCCGCGCCGCTTCCAGAGCAGCGCTAGAAAGAACGGCGCGCCGATCAGCGCGGTCAGGATG

CCGAGCGGAATCTCGGCCGGCGCGGCGAGCGTGCGCGCGGCGAGATCGGCGGCCACCGCGAGCAGCGCGCCGAAAAGCG

CCGCGCCCGGTATCACGATGCGCTGA

>Bucl5_Bm_A193.seq Created: Tuesday, March 25, 2014 10:02 AM

ATGGGCTATGGGTGGACTATCGATGGGTTTTCGATGAGGCTCTCGATGGGTTCGGGTTCGGGTTCGGGTTCGATGAACT

CGATGAGTCAGATGAGTTCGATGGACTTGAGACTCGAAGGACTCGAAGGACTCGAAGGACTCGAAGGACTCGAAGGACT

CGAAGGACTCGAAGGACTCGAAGGACTCGAAGGACTCGAAGGACTCGAAGGACTCGAAGGACTCGAAGGACTCGAAGGA

CCCGAAGGACCCGAAGGACTCGATGGACTCGATGGATTCGATGAACTCGACGAACTCAACGCGAATCGACCACGCCGCT

CGCGAAACGGCGCCCACGCTCGCGTTCGGCATCGCGGCGGCGAGCGTGCCGATGCGGCGGCACGCTCGCCGCGCGCGAA

CGTCGTCGGCCTGTCCGGCAGGTCGGCGCCGAAGGCGCGTGCGCAACGTCATCCTCCCAGCGCGCCGCGCCGCTTCCAG

AGCAGCGCTAGAAAGAACGGCGCGCCGATCAGCGCGGTCAGGATGCCGAGCGGAATCTCGGCCGGCGCGGCGAGCGTGC

GCGCGGCGAGATCGGCGGCCACCGCGAGCAGCGCGCCGAAAAGCGCCGCGCCCGGTATCACGATGCGCTGA

>Bucl5_Bm_China_7.seq Created: Tuesday, March 25, 2014 10:42 AM

ATGGGCTATGGGTGGACTATCGATGGGTTTTCGATGAGGCTCTCGATGGGTTCGGGTTCGGGTTCGGGTTCGATGAACT

CGATGAGTCAGATGAGTTCGATGGACTTGAGACTCGAAGGACTCGAAGGACTCGAAGGACTCGAAGGACTCGAAGGACT

CGAAGGACTCGAAGGATTCGAAGGACTCGAAGGACCCGAAGGACCCGAAGGACTCGATGGACTCGATGGATTCGATGAA

CTCGACGAACTCAACGCGAATCGACCACGCCGCTCGCGAAACGGCGCCCACGCTCGCGTTCGGCATCGCGGCGGCGAGC

GTGCCGATGCGGCGGCACGCTCGCCGCGCGCGAACGTCGTCGGCCTGTCCGGCAGGTCGGCGCCGAAGGCGCGTGCGCA

ACGTCATCCTCCCAGCGCGCCGCGCCGCTTCCAGAGCAGCGCTAGAAAGAACGGCGCGCCGATCAGCGCGGTCAGGATG

CCGAGCGGAATCTCGGCCGGCGCGGCGAGCGTGCGCGCGGCGAGATCGGCGGCCACCGCGAGCAGCGCGCCGAAAAGCG

CCGCGCCCGGTATCACGATGCGCTGA

>Bucl5_Bm_PRL-20.seq Created: Tuesday, March 25, 2014 10:09 AM

ATGGGCTATGGGTGGACTATCGATGGGTTTTCGATGAGGCTCTCGATGGGTTCGGGTTCGGGTTCGATGAACTCGATGA

GTCAGATGAGTTCGATGGACTTGAGACTCGAAGGACTCGAAGGACTCGAAGGACTCGAAGGACTCGAAGGACTCGAAGG

ACTCGAAGGACTCGAAGGATTCGAAGGATTCGAAGGACTCGAAGGACCCGAAGGACCCGAAGGACCCGAAGGACTCGAT

GGACTCGATGGATTCGATGAACTCGACGAACTCAACGCGAATCGACCACGCCGCTCGCGAAACGGCGCCCACGCTCGCG

TTCGGCATCGCGGCGGCGAGCGTGCCGATGCGGCGGCACGCTCGCCGCGCGCGAACGTCGTCGGCCTGTCCGGCAGGTC

GGCGCCGAAGGCGCGTGCGCAACGTCATCCTCCCAGCGCGCCGCGCCGCTTCCAGAGCAGCGCTAGAAAGAACGGCGCG

CCGATCAGCGCGGTCAGGATGCCGAGCGGAATCTCGGCCGGCGCGGCGAGCGTGCGCGCGGCGAGATCGGCGGCCACCG

CGAGCAGCGCGCCGAAAAGCGCCGCGCCCGGTATCACGATGCGCTGA

>Bucl5_Bm_strain_6.seq Created: Tuesday, March 25, 2014 10:00 AM

ATGGGCTATGGGTGGACTATCGATGGGTTTTCGATGAGGCTCTCGATGGGTTCGGGTTCGGGTTCGGGTTCGATGAACT

CGATGAGTCAGATGAGTTCGATGGACTTGAGACTCGAAGGACTCGAAGGACTCGAAGGACTCGAAGGACTCGAAGGACT

CGAAGGACTCGAAGGACTCGAAGGACTCGAAGGACTCGAAGGACTCGAAGGACTCGAAGGACTCGAAGGACTCGAAGGA

CTCGAAGGACCCGAAGGACCCGAAGGACTCGATGGATTCGATGAACTCGACGAACTCAACGCGAATCGACCACGCCGCT

CGCGAAACGGCGCCCACGCTCGCGTTCGGCATCGCGGCGGCGAGCGTGCCGATGCGGCGGCACGCTCGCCGCGCGCGAA

CGTCGTCGGCCTGTCCGGCAGGTCGGCGCCGAAGGCGCGTGCGCAACGTCATCCTCCCAGCGCGCCGCGCCGCTTCCAG

AGCAGCGCTAGAAAGAACGGCGCGCCGATCAGCGCGGTCAGGATGCCGAGCGGAATCTCGGCCGGCGCGGCGAGCGTGC

GCGCGGCGAGATCGGCGGCCACCGCGAGCAGCGCGCCGAAAAGCGCCGCGCCCGGTATCACGATGCGCTGA

>Bucl5_Bm_strain_11.seq Created: Tuesday, March 25, 2014 10:36 AM

ATGGGCTATGGGTGGACTATCGATGGGTTTTCGATGAGGCTCTCGATGGGTTCGGGTTCGGGTTCGGGTTCGGGTTCGA

TGAACTCGATGAGTCAGATGAGTTCGATGGACTTGAGACTCGAAGGACTCGAAGGACTCGAAGGACTCGAAGGACTCGA

AGGACTCGAAGGACTCGAAGGACTCGAAGGACTCGAAGGACCCGAAGGACCCGAAGGACTCGATGGACTCGATGGATTC

GATGAACTCGACGAACTCAACGCGAATCGACCACGCCGCTCGCGAAACGGCGCCCACGCTCGCGTTCGGCATCGCGGCG

GCGAGCGTGCCGATGCGGCGGCACGCTCGCCGCGCGCGAACGTCGTCGGCCTGTCCGGCAGGTCGGCGCCGAAGGCGCG

TGCGCAACGTCATCCTCCCAGCGCGCCGCGCCGCTTCCAGAGCAGCGCTAGAAAGAACGGCGCGCCGATCAGCGCGGTC

AGGATGCCGAGCGGAATCTCGGCCGGCGCGGCGAGCGTGCGCGCGGCGAGATCGGCGGCCACCGCGAGCAGCGCGCCGA

AAAGCGCCGCGCCCGGTATCACGATGCGCTGA

>Bucl5_Bm_NCTC_10229.seq Created: Friday, January 03, 2014 3:26 PM

atgggctatgggtggactatcgatgggttttcgatgaggctctcgatgggttcgggttcgggttcgggttcgggttcga

tgaactcgatgagtcagatgagttcgatggacttgagactcgaaggactcgaaggactcgaaggactcgaaggactcga

aggactcgaaggactcgaaggactcgaaggactcgaaggactcgaaggactcgaaggactcgaaggactcgaaggactc

gaaggactcgaaggactcgaaggactcgaaggactcgaaggactcgaaggactcgaaggactcgaaggacccgaaggac

ccgaaggactcgatggactcgatggattcgatgaactcgacgaactcaacgcgaatcgaccacgccgctcgcgaaacgg

cgcccacgctcgcgttcggcatcgcggcggcgagcgtgccgatgcggcggcacgctcgccgcgcgcgaacgtcgtcggc

ctgtccggcaggtcggcgccgaaggcgcgtgcgcaacgtcatcctcccagcgcgccgcgccgcttccagagcagcgcta

gaaagaacggcgcgccgatcagcgcggtcaggatgccgagcggaatctcggccggcgcggcgagcgtgcgcgcggcgag

atcggcggccaccgcgagcagcgcgccgaaaagcgccgcgcccggtatcacgatgcgctga

>Bucl5_Bm_NCTC_10247.seq Reverse Complement DNA Sequence Untitled Seq #3(1,651)

ATGGGCTATGGGTGGACTATCGATGGGTTTTCGATGAGGCTCTCGATGGGTTCGGGTTCGGGTTCGGGTTCGATGAACT

CGATGAGTCAGATGAGTTCGATGGACTTGAGACTCGAAGGACTCGAAGGACTCGAAGGACTCGAAGGACTCGAAGGACT

CGAAGGACTCGAAGGACTCGAAGGACTCGAAGGACTCGAAGGACTCGAAGGACTCGAAGGACTCGAAGGACTCGAAGGA

CTCGAAGGACTCGAAGGACTCGAAGGACCCGAAGGACCCGAAGGACTCGATGGACTCGATGGATTCGATGAACTCGACG

AACTCAACGCGAATCGACCACGCCGCTCGCGAAACGGCGCCCACGCTCGCGTTCGGCATCGCGGCGGCGAGCGTGCCGA

TGCGGCGGCACGCTCGCCGCGCGCGAACGTCGTCGGCCTGTCCGGCAGGTCGGCGCCGAAGGCGCGTGCGCAACGTCAT

CCTCCCAGCGCGCCGCGCCGCTTCCAGAGCAGCGCTAGAAAGAACGGCGCGCCGATCAGCGCGGTCAGGATGCCGAGCG

GAATCTCGGCCGGCGCGGCGAGCGTGCGCGCGGCGAGATCGGCGGCCACCGCGAGCAGCGCGCCGAAAAGCGCCGCGCC

CGGTATCACGATGCGCTGA

>Bucl5_Bm_FMH.seq Reverse Complement DNA Sequence Untitled Seq #66(1,579)

ATGGGCTATGGGTGGACTATCGATGGGTTTTCGATGAGGCTCTCGATGGGTTCGGGTTCGGGTTCGGGTTCGATGAACT

CGATGAGTCAGATGAGTTCGATGGACTTGAGACTCGAAGGACTCGAAGGACTCGAAGGACTCGAAGGACTCGAAGGACT

CGAAGGACTCGAAGGATTCGAAGGACTCGAAGGACCCGAAGGACCCGAAGGACTCGATGGACTCGATGGATTCGATGAA

CTCGACGAACTCAACGCGAATCGACCACGCCGCTCGCGAAACGGCGCCCACGCTCGCGTTCGGCATCGCGGCGGCGAGC

GTGCCGATGCGGCGGCACGCTCGCCGCGCGCGAACGTCGTCGGCCTGTCCGGCAGGTCGGCGCCGAAGGCGCGTGCGCA

ACGTCATCCTCCCAGCGCGCCGCGCCGCTTCCAGAGCAGCGCTAGAAAGAACGGCGCGCCGATCAGCGCGGTCAGGATG

CCGAGCGGAATCTCGGCCGGCGCGGCGAGCGTGCGCGCGGCGAGATCGGCGGCCACCGCGAGCAGCGCGCCGAAAAGCG

CCGCGCCCGGTATCACGATGCGCTGA

>Bucl5_Bm_JHU.seq Reverse Complement DNA Sequence Untitled Seq #63(1,579)

ATGGGCTATGGGTGGACTATCGATGGGTTTTCGATGAGGCTCTCGATGGGTTCGGGTTCGGGTTCGGGTTCGATGAACT

CGATGAGTCAGATGAGTTCGATGGACTTGAGACTCGAAGGACTCGAAGGACTCGAAGGACTCGAAGGACTCGAAGGACT

CGAAGGACTCGAAGGATTCGAAGGACTCGAAGGACCCGAAGGACCCGAAGGACTCGATGGACTCGATGGATTCGATGAA

CTCGACGAACTCAACGCGAATCGACCACGCCGCTCGCGAAACGGCGCCCACGCTCGCGTTCGGCATCGCGGCGGCGAGC

GTGCCGATGCGGCGGCACGCTCGCCGCGCGCGAACGTCGTCGGCCTGTCCGGCAGGTCGGCGCCGAAGGCGCGTGCGCA

ACGTCATCCTCCCAGCGCGCCGCGCCGCTTCCAGAGCAGCGCTAGAAAGAACGGCGCGCCGATCAGCGCGGTCAGGATG

CCGAGCGGAATCTCGGCCGGCGCGGCGAGCGTGCGCGCGGCGAGATCGGCGGCCACCGCGAGCAGCGCGCCGAAAAGCG

CCGCGCCCGGTATCACGATGCGCTGA

>Bucl5_Bm_ATCC_10399.seq Created: Tuesday, March 25, 2014 10:05 AM

ATGGGCTATGGGTGGACTATCGATGGGTTTTCGATGAGGCTCTCGATGGGTTCGGGTTCGGGTTCGGGTTCGATGAACT

CGATGAGTCAGATGAGTTCGATGGACTTGAGACTCGAAGGACTCGAAGGACTCGAAGGACTCGAAGGACTCGAAGGACT

CGAAGGACTCGAAGGACTCGAAGGACTCGAAGGACTCGAAGGACTCGAAGGATTCGAAGGACTCGAAGGACCCGAAGGA

CCCGAAGGACTCGATGGACTCGATGGATTCGATGAACTCGACGAACTCAACGCGAATCGACCACGCCGCTCGCGAAACG

GCGCCCACGCTCGCGTTCGGCATCGCGGCGGCGAGCGTGCCGATGCGGCGGCACGCTCGCCGCGCGCGAACGTCGTCGG

CCTGTCCGGCAGGTCGGCGCCGAAGGCGCGTGCGCAACGTCATCCTCCCAGCGCGCCGCGCCGCTTCCAGAGCAGCGCT

AGAAAGAACGGCGCGCCGATCAGCGCGGTCAGGATGCCGAGCGGAATCTCGGCCGGCGCGGCGAGCGTGCGCGCGGCGA

GATCGGCGGCCACCGCGAGCAGCGCGCCGAAAAGCGCCGCGCCCGGTATCACGATGCGCTGA

>Bucl5_Bm_GB8_horse_4.seq Reverse Complement DNA Sequence Untitled Seq #69(1,579)

ATGGGCTATGGGTGGACTATCGATGGGTTTTCGATGAGGCTCTCGATGGGTTCGGGTTCGGGTTCGGGTTCGATGAACT

CGATGAGTCAGATGAGTTCGATGGACTTGAGACTCGAAGGACTCGAAGGACTCGAAGGACTCGAAGGACTCGAAGGACT

CGAAGGACTCGAAGGATTCGAAGGACTCGAAGGACCCGAAGGACCCGAAGGACTCGATGGACTCGATGGATTCGATGAA

CTCGACGAACTCAACGCGAATCGACCACGCCGCTCGCGAAACGGCGCCCACGCTCGCGTTCGGCATCGCGGCGGCGAGC

GTGCCGATGCGGCGGCACGCTCGCCGCGCGCGAACGTCGTCGGCCTGTCCGGCAGGTCGGCGCCGAAGGCGCGTGCGCA

ACGTCATCCTCCCAGCGCGCCGCGCCGCTTCCAGAGCAGCGCTAGAAAGAACGGCGCGCCGATCAGCGCGGTCAGGATG

CCGAGCGGAATCTCGGCCGGCGCGGCGAGCGTGCGCGCGGCGAGATCGGCGGCCACCGCGAGCAGCGCGCCGAAAAGCG

CCGCGCCCGGTATCACGATGCGCTGA

>Bucl5_Bm_ATCC_23344.seq Reverse Complement DNA Sequence Untitled Seq #24(1,579)

atgggctatgggtggactatcgatgggttttcgatgaggctctcgatgggttcgggttcgggttcgggttcgatgaact

cgatgagtcagatgagttcgatggacttgagactcgaaggactcgaaggactcgaaggactcgaaggactcgaaggact

cgaaggactcgaaggattcgaaggactcgaaggacccgaaggacccgaaggactcgatggactcgatggattcgatgaa

ctcgacgaactcaacgcgaatcgaccacgccgctcgcgaaacggcgcccacgctcgcgttcggcatcgcggcggcgagc

gtgccgatgcggcggcacgctcgccgcgcgcgaacgtcgtcggcctgtccggcaggtcggcgccgaaggcgcgtgcgca

acgtcatcctcccagcgcgccgcgccgcttccagagcagcgctagaaagaacggcgcgccgatcagcgcggtcaggatg

ccgagcggaatctcggccggcgcggcgagcgtgcgcgcggcgagatcggcggccaccgcgagcagcgcgccgaaaagcg

ccgcgcccggtatcacgatgcgctga

>Bucl5_Bm_SAVP1.seq Created: Saturday, January 04, 2014 12:56 PM

atgggctatgggtggactatcgatgggttttcgatgaggctctcgatgggttcgggttcgggttcgatgaactcgatga

gtcagatgagttcgatggacttgagactcgaaggactcgaaggactcgaaggactcgaaggactcgaaggactcgaagg

attcgaaggattcgaaggactcgaaggacccgaaggacccgaaggacccgaaggactcgatggactcgatggattcgat

gaactcgacgaactcaacgcgaatcgaccacgccgctcgcgaaacggcgcccacgctcgcgttcggcatcgcggcggcg

agcgtgccgatgcggcggcacgctcgccgcgcgcgaacgtcgtcggcctgtccggcaggtcggcgccgaaggcgcgtgc

gcaacgtcatcctcccagcgcgccgcgccgcttccagagcagcgctagaaagaacggcgcgccgatcagcgcggtcagg

atgccgagcggaatctcggccggcgcggcgagcgtgcgcgcggcgagatcggcggccaccgcgagcagcgcgccgaaaa

gcgccgcgcccggtatcacgatgcgctga

>Bucl6_Bps_K96243.seq Created: Tuesday, January 21, 2014 10:22 AM

ttgggcgcgttgggtgcgtcgggcgcggcgggtgcggaaagcgcgcccggcggccgcgcgcggctgcgtcggaccgtcg

ccgttccgaccgctcgcgcgacaaccgcgcacgaatcgctttaa

>Bucl6_Bps_MSHR_305.seq Created: Tuesday, January 21, 2014 10:27 AM

ttgggtgcgttgggtgcgttgggtgcgtcgggtgcggcgggtgcggcgggtgcggcgggtgcggcgggtgcggcgggtg

cggaaagcgcgcccggcggccgcgcgcggctgcgtcggaccgtcgccgttccgaccgctcgcgcgacaaccgcgcacga

atcgctttaa

>Bucl6_Bps_1106a.seq Created: Tuesday, January 21, 2014 10:08 AM

ttgggtgcgttgggtgcgtcgggtgcggcgggtgcggcgggtgcggaaagcgcgcccggcggccgcgcgcggctgcgtc

ggaccgtcgccgttccgaccgctcgcgcgacaaccgcgcacgaatcgctttaa

>Bucl6_Bps_MSHR_346.seq Reverse Complement DNA Sequence Untitled Seq #21(1,237)

TTGGGCGCGTTGGGCGCGTCGGGCGCGTCGGGCGCGTCGGGCGCGGCGGGTGCGTCGGGCGCGTCGGGTGCGTCGGGTG

CGTCGGGTGCGTCGGGTGCGTCGGGCGCGGCGGGTGCGGCGGGTGCGGAAAGCGCGCCCGGCGGCCGCGCGCGGCTGCG

TCGGACCGTCGCCGTTCCGACCGCTCGCGCGACAACCGCGCACGAATCGCTTTAA

>Bucl6_Bps_1026b.seq Created: Monday, December 30, 2013 4:11 PM

ttgggtgcgttgggtgcgtcgggtgcgtcgggtgcggcgggtgcggcgggtgcggcgggtgcggcgggtgcggcgggtg

cggcgggtgcggcgggtgcggcgggtgcggcgggtgcggcgggcgcgtcgggcgcggcgggcgcggcgggtgcggcggg

tgcggcgggcgcggcgggtgcggaaagcgcgcccggcggccgcgcgcggctgcgtcggaccgtcgccgttccgaccgct

cgcgcgacaaccgcgcacgaatcgctttaa

>Bucl6_Bps_1710b.seq Created: Tuesday, January 21, 2014 10:12 AM

ttgggtgcgttgggtgcgttgggtgcgtcgggtgcgtcgggtgcgtcgggtgcgtcgggtgcgtcgggtgcgtcgggtg

cgtcgggtgcgtcgggtgcgtcgggtgcgtcgggtgcgtcgggtgcggaaagcgcgcccggcggccgcgcgcggctgcg

tcggaccgtcgccgttccgaccgctcgcgcgacaaccgcgcacgaatcgctttaa

>Bucl6_Bps_BPC006.seq Created: Tuesday, January 21, 2014 10:18 AM

ttgggtgcgttgggtgcgtcgggtgcggcgggtgcggcgggtgcggaaagcgcgcccggcggccgcgcgcggctgcgtc

ggaccgtcgccgttccgaccgctcgcgcgacaaccgcgcacgaatcgctttaa

>A3N8K6_BURP6_Bucl6_Bp_668.seq Created: Tuesday, January 21, 2014 9:40 AM

ttgggcgcgtcgggcgcggcgggtgcgtcgggtgcgtcgggcgcggcgggtgcggcgggtgcggcgggtacgtcgggta

cgtcgggcgcggcgggtgcggcgggtgcggcgggtgcggcgggtgcggcgggtgcggcgggtgcggaaagcgcgcccgg

cggccgcgcgcggctgcgtcggaccgtcgccgttccgaccgctcgcgcgacaaccgcgcacgaatcgctttaa

>Bucl6_Bps_NCTC_13179.seq Created: Tuesday, January 21, 2014 10:32 AM

ttgggcgcgttgggcgcgttgggcgcgtcgggtgcgtcgggtgcgtcgggcgcggcgggtgcggcgggtgcggcgggtg

cggcggccgcgcgcggctgcgtcggaccgtcgccgttccgaccgctcgcgcgacaaccgcgcacgaatcgctttaa

>Bucl6_Bps_MSHR146.seq >gi|575433883|gb|CP004042.1|:2323872-2324018 Burkholderia pseudomallei MSHR146 chromosome 1, complete sequence

TTGGGTGCGTTGGGTGCGTCGGGTGCGTCGGGTGCGGAAAGCGCGCCCGGCGGCCGCGCGCGGCTGCGTCGGACCGTCG

CCGTTCCGACCGCTCGCGCGACAACCGCGCACGAATCGCTTTAA

>Bucl6_Bps_MSHR511.seq >gi|574600368|gb|CP004023.1|:2178363-2178509 Burkholderia pseudomallei MSHR511 chromosome 1, complete sequence

TTGGGTGCGTTGGGTGCGTCGGGTGCGTCGGGTGCGGAAAGCGCGCCCGGCGGCCGCGCGCGGCTGCGTCGGACCGTCG

CCGTTCCGACCGCTCGCGCGACAACCGCGCACGAATCGCTTTAA

>Bucl6_Bps_MSHR520.seq >gi|589299693|gb|CP004368.1|:2797685-2797885 Burkholderia pseudomallei MSHR520 chromosome 1, complete sequence

TTGGGTGCGTTGGGTGCGTTGGGTGCGTCGGGTGCGGCGGGTGCGGCGGGTGCGGCGGGTGCGGCGGGTGCGGCGGGTG

CGGAAAGCGCGCCCGGCGGCCGCGCGCGGCTGCGTCGGACCGTCGCCGTTCCGACCGCTCGCGCGACAACCGCGCACGA

ATCGCTTTAA

>Bucl6_Bps_NAU20B-16.seq >gi|568789317|gb|CP004003.1|:3988960-3989106 Burkholderia pseudomallei NAU20B-16 chromosome 1, complete sequence

TTGGGTGCGTTGGGTGCGTCGGGTGCGTCGGGTGCGGAAAGCGCGCCCGGCGGCCGCGCGCGGCTGCGTCGGACCGTCG

CCGTTCCGACCGCTCGCGCGACAACCGCGCACGAATCGCTTTAA

>Bucl6_Bps_NCTC_13178.seq >gi|568795288|gb|CP004001.1|:1660079-1660329 Burkholderia pseudomallei NCTC 13178 chromosome 1, complete sequence

TTGGGTGCGTTGGGTGCGTTGGGTGCGTCGGGTGCGTCGGGTGCGTCGGGTGCGTCGGGCGCGGCGGGTGCGGCGGGTG

CGTCGGGTGCGTCGGGTGCGTCGGGTGCGTCGGGTGCGTCGGGTGCGGCGGGTGCGGCGGGTGCGGCGGCCGCGCGCGG

CTGCGTCGGACCGTCGCCGTTCCGACCGCTCGCGCGACAACCGCGCACGAATCGCTTTAA

>Bucl6_Bm_2000031281.seq Created: Tuesday, March 25, 2014 8:47 PM

TTGGGTGCGTTGGGTGCGTTGGGTGCGTCGGGTGCGTCGGGTGCGTCGGGTGCGTCGGGTGCGTCGGGTGCGTCGGGTG

CGTCGGGTGCGTCGGGTGCGGCGGGCGCGGCGGGTGCGGCGGGTGCGGCGGGTGCGGCGGGTGCGGCGGGTGCGGCGGG

TGCGGAAAGCGCGCCCGGCGGCCGCGCGCGGCTGCGTCGGACCGTCGCCGTTCCGACCGCTCGCGCGACAACCGCGCAC

GAATCGCTTTAA

>Bucl6_Bm_2002721280.seq Created: Tuesday, March 25, 2014 9:15 PM

TTGGGTGCGTTGGGTGCGTTGGGTGCGTTGGGTGCGTCGGGTGCGTCGGGTGCGTCGGGTGCGTCGGGTGCGTCGGGTG

CGTCGGGTGCGTCGGGTGCGTCGGGTGCGGCGGGCGCGGCGGGTGCGGCGGGTGCGGCGGGTGCGGAAAGCGCGCCCGG

CGGCCGCGCGCGGCTGCGTCGGACCGTCGCCGTTCCGACCGCTCGCGCGACAACCGCGCACGAATCGCTTTAA

>Bucl6_Bm_A188.seq Reverse Complement DNA Sequence Untitled Seq #1(1,264)

TTGGGTGCGTTGGGTGCGTTGGGTGCGTTGGGTGCGTCGGGTGCGTCGGGTGCGTCGGGTGCGTCGGGTGCGTCGGGTG

CGTCGGGTGCGTCGGGTGCGTCGGGTGCGGCGGGCGCGGCGGGTGCGGCGGGTGCGGCGGGTGCGGAAAGCGCGCCCGG

CGGCCGCGCGCGGCTGCGTCGGACCGTCGCCGTTCCGACCGCTCGCGCGACAACCGCGCACGAATCGCTTTAA

>Bucl6_Bm_A193.seq Reverse Complement DNA Sequence Untitled Seq #23(1,273)

TTGGGTGCGTTGGGTGCGTTGGGTGCGTTGGGTGCGTCGGGTGCGTCGGGTGCGTCGGGTGCGTCGGGTGCGTCGGGTG

CGTCGGGTGCGTCGGGTGCGTCGGGTGCGGCGGGCGCGGCGGGTGCGGCGGGTGCGGCGGGTGCGGCGGGTGCGGAAAG

CGCGCCCGGCGGCCGCGCGCGGCTGCGTCGGACCGTCGCCGTTCCGACCGCTCGCGCGACAACCGCGCACGAATCGCTT

TAA

>Bucl6_Bm_China_7.seq Reverse Complement DNA Sequence Untitled Seq #10(1,282)

TTGGGTGCGTTGGGTGCGTTGGGTGCGTCGGGTGCGTCGGGTGCGTCGGGTGCGTCGGGTGCGTCGGGTGCGTCGGGTG

CGTCGGGTGCGTCGGGTGCGGCGGGCGCGGCGGGTGCGGCGGGTGCGGCGGGTGCGGCGGGTGCGGCGGGTGCGGCGGG

TGCGGAAAGCGCGCCCGGCGGCCGCGCGCGGCTGCGTCGGACCGTCGCCGTTCCGACCGCTCGCGCGACAACCGCGCAC

GAATCGCTTTAA

>Bucl6_Bm_PRL-20.seq Created: Tuesday, March 25, 2014 9:10 PM

TTGGGTGCGTTGGGTGCGTTGGGTGCGTCGGGTGCGTCGGGTGCGTCGGGTGCGTCGGGTGCGTCGGGTGCGTCGGGTG

CGGCGGGCGCGGCGGGTGCGGCGGGTGCGGCGGGTGCGGCGGGTGCGGCGGGTGCGGAAAGCGCGCCCGGCGGCCGCGC

GCGGCTGCGTCGGACCGTCGCCGTTCCGACCGCTCGCGCGACAACCGCGCACGAATCGCTTTAA

>Bucl6_Bm_strain_6.seq Reverse Complement DNA Sequence Untitled Seq #7(1,291)

TTGGGTGCGTTGGGTGCGTTGGGTGCGTCGGGTGCGTCGGGTGCGTCGGGTGCGTCGGGTGCGTCGGGTGCGTCGGGTG

CGTCGGGTGCGTCGGGTGCGTCGGGTGCGGCGGGCGCGGCGGGTGCGGCGGGTGCGGCGGGTGCGGCGGGTGCGGCGGG

TGCGGCGGGTGCGGAAAGCGCGCCCGGCGGCCGCGCGCGGCTGCGTCGGACCGTCGCCGTTCCGACCGCTCGCGCGACA

ACCGCGCACGAATCGCTTTAA

>Bucl6_Bm_strain_11.seq Created: Tuesday, March 25, 2014 8:51 PM

TTGGGTGCGTTGGGTGCGTTGGGTGCGTTGGGTGCGTTGGGTGCGTCGGGTGCGTCGGGTGCGTCGGGTGCGTCGGGTG

CGTCGGGTGCGTCGGGTGCGTCGGGTGCGTCGGGTGCGGCGGGCGCGGCGGGTGCGGCGGGTGCGGCGGGTGCGGCGGG

TGCGGCGGGTGCGGCGGGTGCGGAAAGCGCGCCCGGCGGCCGCGCGCGGCTGCGTCGGACCGTCGCCGTTCCGACCGCT

CGCGCGACAACCGCGCACGAATCGCTTTAA

>Bucl6_Bm_NCTC_10229.seq Created: Tuesday, January 21, 2014 9:57 AM

ttgggtgcgttgggtgcgttgggtgcgttgggtgcgtcgggtgcgtcgggtgcgtcgggtgcgtcgggtgcgtcgggtg

cgtcgggtgcgtcgggtgcgtcgggtgcgtcgggtgcggcgggcgcggcgggtgcggcgggtgcggcgggtgcggcggg

tgcggaaagcgcgcccggcggccgcgcgcggctgcgtcggaccgtcgccgttccgaccgctcgcgcgacaaccgcgcac

gaatcgctttaa

>Bucl6_Bm_NCTC_10247.seq Reverse Complement DNA Sequence Untitled Seq #17(1,274)

TTGGGTGCGTTGGGTGCGTTGGGTGCGTCGGGTGCGTCGGGTGCGTCGGGTGCGTCGGGTGCGTCGGGTGCGTCGGGTG

CGTCGGGTGCGTCGGGTGCGGCGGGCGCGGCGGGTGCGGCGGGTGCGGCGGGTGCGGCGGGTGCGGCGGGTGCGGAAAG

CGCGCCCGGCGGCCGCGCGCGGCTGCGTCGGACCGTCGCCGTTCCGACCGCTCGCGCGACAACCGCGCACGAATCGCTT

TAA

>Bucl6_Bm_FMH.seq Reverse Complement DNA Sequence Untitled Seq #18(1,282)

TTGGGTGCGTTGGGTGCGTTGGGTGCGTCGGGTGCGTCGGGTGCGTCGGGTGCGTCGGGTGCGTCGGGTGCGTCGGGTG

CGTCGGGTGCGTCGGGTGCGGCGGGCGCGGCGGGTGCGGCGGGTGCGGCGGGTGCGGCGGGTGCGGCGGGTGCGGCGGG

TGCGGAAAGCGCGCCCGGCGGCCGCGCGCGGCTGCGTCGGACCGTCGCCGTTCCGACCGCTCGCGCGACAACCGCGCAC

GAATCGCTTTAA

>Bucl6_Bm_JHU.seq Reverse Complement DNA Sequence Untitled Seq #16(1,282)

TTGGGTGCGTTGGGTGCGTTGGGTGCGTCGGGTGCGTCGGGTGCGTCGGGTGCGTCGGGTGCGTCGGGTGCGTCGGGTG

CGTCGGGTGCGTCGGGTGCGGCGGGCGCGGCGGGTGCGGCGGGTGCGGCGGGTGCGGCGGGTGCGGCGGGTGCGGCGGG

TGCGGAAAGCGCGCCCGGCGGCCGCGCGCGGCTGCGTCGGACCGTCGCCGTTCCGACCGCTCGCGCGACAACCGCGCAC

GAATCGCTTTAA

>Bucl6_Bm_ATCC_10399.seq Reverse Complement DNA Sequence Untitled Seq #27(1,246)

TTGGGTGCGTTGGGTGCGTTGGGTGCGTTGGGTGCGTCGGGTGCGTCGGGTGCGTCGGGTGCGTCGGGTGCGTCGGGTG

CGGCGGGCGCGGCGGGTGCGGCGGGTGCGGCGGGTGCGGCGGGTGCGGAAAGCGCGCCCGGCGGCCGCGCGCGGCTGCG

TCGGACCGTCGCCGTTCCGACCGCTCGCGCGACAACCGCGCACGAATCGCTTTAA

>Bucl6_Bm_GB8_horse_4.seq Reverse Complement DNA Sequence Untitled Seq #20(1,282)

TTGGGTGCGTTGGGTGCGTTGGGTGCGTCGGGTGCGTCGGGTGCGTCGGGTGCGTCGGGTGCGTCGGGTGCGTCGGGTG

CGTCGGGTGCGTCGGGTGCGGCGGGCGCGGCGGGTGCGGCGGGTGCGGCGGGTGCGGCGGGTGCGGCGGGTGCGGCGGG

TGCGGAAAGCGCGCCCGGCGGCCGCGCGCGGCTGCGTCGGACCGTCGCCGTTCCGACCGCTCGCGCGACAACCGCGCAC

GAATCGCTTTAA

>Bucl6_Bm_ATCC_23344.seq Created: Tuesday, January 21, 2014 9:47 AM

ttgggtgcgttgggtgcgttgggtgcgtcgggtgcgtcgggtgcgtcgggtgcgtcgggtgcgtcgggtgcgtcgggtg

cgtcgggtgcgtcgggtgcggcgggcgcggcgggtgcggcgggtgcggcgggtgcggcgggtgcggcgggtgcggcggg

tgcggaaagcgcgcccggcggccgcgcgcggctgcgtcggaccgtcgccgttccgaccgctcgcgcgacaaccgcgcac

gaatcgctttaa

>Bucl6_Bm_SAVP1.seq Created: Tuesday, January 21, 2014 10:02 AM

ttgggtgcgttgggtgcgttgggtgcgtcgggtgcgtcgggtgcgtcgggtgcgtcgggtgcgtcgggtgcgtcgggtg

cggcgggcgcggcgggtgcggcgggtgcggcgggtgcggcgggtgcggcgggtgcggaaagcgcgcccggcggccgcgc

gcggctgcgtcggaccgtcgccgttccgaccgctcgcgcgacaaccgcgcacgaatcgctttaa

>A3P4A7_BURP0_Bucl7_Bp_1106a.seq Created: Tuesday, October 29, 2013 6:45 PM

ATGGACACGACCACGCAGGACGGGAACCGGCCGCCGTTCGACGCGCCGCGAGGCGGGCGCGGAGACGGCGACAAGAACG

ACGACACGCGACTCCCCGCTTGCAACGAACGCGCGCGGCGGCGCGCCGGCGTCGCGGCGAAGCGCACGCGCCGCGCCCT

GAACGGCGCGGCGAGCCGCACCGGCCTGGACGCCGAATGCCGCGTTCGTCCGAAGAACGCGCGACGTCGCACGAGCGTT

TCGAAAGCGACGCGGGCCGTTCATTGGCGCGCCCGACGCCGCGCGATCGTCTCGATTCGCGCGGCGCAGCCGAAGGGCG

GACGTGGCGGTGCGCGAGCGAGCGCGCATCCCCCGATGCGTCCGATGCGCACGCGTGCGCAGATTCCGCGAGCCGATCG

CGAGCCTGGCTTATCCGGCTCGTCCGGCTCGTCCGGCTCGTCCGGCTCGTCCGGCTCGTCCGGCTCGTCCGGCTCGTCC

GGCTCGTCCGGCTCGTCCGGCTCGTCCGGCGCGTCCGGCGCGTCCGGCGTCGCGAACTTCGATCCGATTTTCTTCTTCT

ACCATCGCCTGCGTTCGCACGGCAGCCCGCAGCAGGCAAGCCCGACACAGGCCCGACGCACGAAGCGCGATCGCGCGGC

AGCGTAG

>Bucl7_Bps_BPC006.seq Created: Sunday, November 24, 2013 2:35 PM

ATGGACACGACCACGCAGGACGGGAACCGGCCGCCGTTCGACGCGCCGCGAGGCGGGCGCGGAGACGGCGACAAGAACG

ACGACACGCGACTCCCCGCTTGCAACGAACGCGCGCGGCGGCGCGCCGGCGTCGCGGCGAAGCGCACGCGCCGCGCCCT

GAACGGCGCGGCGAGCCGCACCGGCCTGGACGCCGAATGCCGCGTTCGTCCGAAGAACGCGCGACGTCGCACGAGCGTT

TCGAAAGCGACGCGGGCCGTTCATTGGCGCGCCCGACGCCGCGCGATCGTCTCGATTCGCGCGGCGCAGCCGAAGGGCG

GACGTGGCGGTGCGCGAGCGAGCGCGCATCCCCCGATGCGTCCGATGCGCACGCGTGCGCAGATTCCGCGAGCCGATCG

CGAGCCTGGCTTATCCGGCTCGTCCGGCTCGTCCGGCTCGTCCGGCTCGTCCGGCTCGTCCGGCTCGTCCGGCTCGTCC

GGCTCGTCCGGCTCGTCCGGCTCGTCCGGCGCGTCCGGCGCGTCCGGCGTCGCGAACTTCGATCCGATTTTCTTCTTCT

ACCATCGCCTGCGTTCGCACGGCAGCCCGCAGCAGGCAAGCCCGACACAGGCCCGACGCACGAAGCGCGATCGCGCGGC

AGCGTAG

>Bucl7_Bps_1710b.seq Created: Tuesday, December 31, 2013 1:07 PM

atggacacgaccacgcaggacgggaaccggccgccgttcgacgcgccgcgaggcgggcgcggagacggcgacaagaacg

acgacacgcgactccccgattgcaacgaacgcgcgcggcggcgcgccggcgtcgcggcgaagcgcacgcgccgcgccct

gaacggcgcggcgagccgcaccggcctggacgccgaatgccgcgttcgtccgaagaacgcgcgacgtcgcacgagcgtt

tcgaaagcgacgcgggccgttcattggcgcgcccgacgccgcgcggtcgtcccgattcgcgcggcgcagccgaagggcg

gacgtggcggtgcgcgagcgagcgcgcatcccccgatgcgtccgatgcgcacgcgtgcgcagattccgcgagccgatcg

cgagcctggcttatccggcttatccggcttatccggctcgtctggcgcgtccggcgcgtccggcgtcgcgaacttcgat

ccgattttcttcttctaccatcgcctgcgttcgcacggcagcccgcagcaggcaagcccgacacaggcccgacgcacga

agcgcgatcgcgcggcagcgtag

>Bucl7_Bps_668.seq Created: Monday, December 30, 2013 1:31 PM

atggacacgaccacgcaggacgggaaccggccgccgttcgacgcgccgcgaggcgggcgcggagacggcgacaagaacg

acgacacgcgactccccgattgcaacgaacgcgcgcggcggcgcgccggcgtcgcggcgaagcgcacgcgccgcgccct

gaacggcgcggcgagccgcaccggcctggacgccgaatgccgcgttcgtccgaagaacgcgcgacgtcgcacgagcgtt

tcgaaagcggcgcgggccgttcattggcgcgcccgacgccgcgcgatcgtcccgattcacgcggcccagccgaagggcg

gacgtggcggtgcgcgagcgagcgcgcatcccccgatgcgtccgatgcgcacgcgtgcgcagattccgcgagccgatcg

cgagcctggcttatccggcttatccggcttatccggctcgtccagcacgtccggcgtcgcgaacttcgatccgattttc

ttcttctaccatcgcctgcgttcgcacggcagcccgcagcaggcaagcccgacacaggcccgacgcacgaagcgcgatc

gcgcggcagcgtag

>Bucl7_Bps_1026b.seq Created: Monday, December 30, 2013 4:48 PM

atggacacgaccacgcaggacgggaaccggccgccgttcgacgcgccgcgaggcgggcgcggagacggcgacaagaacg

acgacacgcgactccccgattgcaacgaacgcgcgcggcggcgcgccggcgtcgcggcgaagcgcacgcgccgcgccct

gaacggcgcggcgagccgcaccggcctggacgccgaatgccgcgttcgtccgaagaacgcgcgacgtcgcacgagcgtt

tcgaaagcgacgcgggccgttcattggcgcgcccgacgccgcgcgatcgtcccgattcgcgcggcgcagccgaagggcg

gacgtggcggtgcgcgagcgagcgcgcatcccccgatgcgtccgatgcgcacgcgtgcgcagattccgcgagccgatcg

cgagcctggcttatccggcttatccggcttatccggctcgtccagctcgtccagctcgtccggcgcgtccggcgtcgcg

aacttcgatccgattttcttcttctaccatcgcctgcgttcgcacggcagcccgcagcaggcaagcccgacacaggccc

gacgcacgaagcgcgatcgcgcgacagcgtag

>Bucl7_Bps_MSHR_305.seq Created: Thursday, January 02, 2014 11:50 AM

atggacacgaccacgcaggacgggaaccggccgccgttcgacgcgccgcgaggcgggcgcggagacggcgacaagaacg

acgacacgcgattccccgattgcaacgaacgcgcgcggcggcgcgccggcgtcgcggcgaagcgcacgcgccgcgccct

gaacggcgcggcgagccgcaccggcctggacgccgaatgccgcgttcgtccgaagaacgcgcgacgtcgcacgagcgtt

tcgaaagcggcgcgggccgttcattggcgcgcccgacgccgcgcgatcgtcccgattcacgcggcccagccgaagggcg

gacgtggcggtgcgcgagcgagcgcgcatcccccgatgcgtccgatgcgcacgcgtgcgcagattccgcgagccgatcg

cgagcctggcttatccggcttatccggcttatccggcgcgtccggcgcgtccggcgcgtccggcgcgtccggcgtcgcg

aacttcgatccgattttcttcttctaccatcgcctgcgttcgcacggcagcccgcagcaagcaagcccgacacaggccc

gacgcacgaagcgcgatcgcgcgacagcgtag

>Bucl7_Bps_NCTC_13179.seq Reverse Complement DNA Sequence Untitled Seq #5(1,585)

atggacacgaccacgcaggacgggaaccggccgccgttcgacgcgccgcgaggcgggcgcggagacggcgacaagaacg

acgacacgcgactccccgattgcaacgaacgcgcgcggcggcgcgccggcgtcgcggcgaagcgcacgcgccgcgccct

gaacggcgcggcgagccgcaccggcctggacgccgaatgccgcgttcgtccgaagaacgcgcgacgtcgcacgagcgtt

tcgaaagcggcgcgggccgttcattggcgcgcccgacgccgcgcgatcgtcccgattcgcgcggcgcagccgaagggcg

gacgtggcggtgcgcgagcgagcgcgcatcccccgatgcgtccgatgcgcacgcgtgcgcagattccgcgagccgatcg

cgagcctggcttatccggcttatccggcttatccggctcgtccggctcgtccggctcgtccggcgcgtccggcgtcgcg

aacttcgatccgattttcttcttctaccatcgcctgcgttcgcacggcagcccgcagcaggcaagcccgacacaggccc

gacgcacgaagcgcgatcgcgcggcagcgtag

>Bucl7_Bm_ATCC_23344.seq Created: Friday, January 03, 2014 1:41 PM

atggacacgaccacgcaggacgggaatcggccgccgttcgacgcgccgcgaggcgggcgcggagacggcgacaagaacg

acgacacgcgactccccgattgcaacgaacgcgcgcggcggcgcgccggcgtcgcggcgaagcgcacgcgccgcgccct

gaacggcgcggcgagccgcaccggcctggacgccgaatgccgcgttcgtccgaagaacgcgcgacgtcgcacgagcgtt

tcgaaagcgacgcgggccgttcattggcgcgcccgacgccgcgcgatcgtcccgattcacgcggcccagccgaagggcg

gacgtggcggtgcgcgagcgagcgcgcatcccccgatgcgtccgatgcgcacgcgtgcgcagattccgcgagccgatcg

cgagcctggcttatccggcttatccggctcgtctggcgcgtccggcgcgtccggcgcgtccggcgtcgcgaacttcgat

ccgattttcttcttctaccatcgcctgcgttcgcacggcagcccgcagcaggcaagcccgacacaggcccgacgcacga

agcgcgatcgcgcggcagcgtag

>Bucl7_Bm_NCTC_10229.seq Reverse Complement DNA Sequence Untitled Seq #43(1,576)

atggacacgaccacgcaggacgggaatcggccgccgttcgacgcgccgcgaggcgggcgcggagacggcgacaagaacg

acgacacgcgactccccgattgcaacgaacgcgcgcggcggcgcgccggcgtcgcggcgaagcgcacgcgccgcgccct

gaacggcgcggcgagccgcaccggcctggacgccgaatgccgcgttcgtccgaagaacgcgcgacgtcgcacgagcgtt

tcgaaagcgacgcgggccgttcattggcgcgcccgacgccgcgcgatcgtcccgattcacgcggcccagccgaagggcg

gacgtggcggtgcgcgagcgagcgcgcatcccccgatgcgtccgatgcgcacgcgtgcgcagattccgcgagccgatcg

cgagcctggcttatccggcttatccggctcgtctggcgcgtccggcgcgtccggcgcgtccggcgtcgcgaacttcgat

ccgattttcttcttctaccatcgcctgcgttcgcacggcagcccgcagcaggcaagcccgacacaggcccgacgcacga

agcgcgatcgcgcggcagcgtag

>Bucl7_Bm_NCTC_10247.seq Created: Friday, January 03, 2014 5:59 PM

atggacacgaccacgcaggacgggaatcggccgccgttcgacgcgccgcgaggcgggcgcggagacggcgacaagaacg

acgacacgcgactccccgattgcaacgaacgcgcgcggcggcgcgccggcgtcgcggcgaagcgcacgcgccgcgccct

gaacggcgcggcgagccgcaccggcctggacgccgaatgccgcgttcgtccgaagaacgcgcgacgtcgcacgagcgtt

tcgaaagcgacgcgggccgttcattggcgcgcccgacgccgcgcgatcgtcccgattcacgcggcccagccgaagggcg

gacgtggcggtgcgcgagcgagcgcgcatcccccgatgcgtccgatgcgcacgcgtgcgcagattccgcgagccgatcg

cgagcctggcttatccggcttatccggctcgtctggcgcgtccggcgcgtccggcgcgtccggcgtcgcgaacttcgat

ccgattttcttcttctaccatcgcctgcgttcgcacggcagcccgcagcaggcaagcccgacacaggcccgacgcacga

agcgcgatcgcgcggcagcgtag

>Bucl7_Bps_K96243.seq Created: Monday, January 20, 2014 6:59 PM

atggacacgaccacgcaggacgggaaccggccgccgttcgacgcgccgcgaggcgggcgcggagacggcgacaagaacg

acgacacgcgactccccgattgcaacgaacgcgcgcggcggcgcgccggcgtcgcggcgaagcgcacgcgccgcgccct

gaacggcgcggcgagccgcaccggcctggacgccgaatgccgcgttcgtccgaagaacgcgcgacgtcgcacgagcgtt

tcgaaagcgacgcgggccgttcattggcgcgcccgacgccgcgcgatcgtcccgattcgcgcggcgcagccgaagggcg

gacgtggcggtgcgcgagcgagcgcgcatcccccgatgcgtccgatgcgcacgcgtgcgcagattccgcgagccgatcg

cgagcctggcttatccggcttatccggctcgtccagctcgtccggcgcgtccggcgtcgcgaacttcgatccgattttc

ttcttctaccatcgcctgcgttcgcacggcagcccgcagcaggcaagcccgacacaggcccgacgcacgaagcgcgatc

gcgcgacagcgtag

>Bucl7_Bps_MSHR146.seq >gi|575437442|gb|CP004043.1|:946056-946640 Burkholderia pseudomallei MSHR146 chromosome 2, complete sequence

ATGGACACGACCACGCAGGACGGGAACCGGCCGCCGTTCGACGCGCCGCGAGGCGGGCGCGGAGACGGCGACAAGAACG

ACGACACGCGACTCCCCGATTGCAACGAACGCGCGCGGCGGCGCGCCGGCGTCGCGGCGAAGCGCACGCGCCGCGCCCT

GAACGGCGCGGCGAGCCGCACCGGCCTGGACGCCGAATGCCGCGTTCGTCCGAAGAACGCGCGACGTCGCACGAGCGTT

TCGAAAGCGGCGCTGGCCGTTCATTGGCGCGCCCGACGCCGCGCGATCGTCCCGATTCACGCGGCCCAGCCGAAGGGCG

GACGTGGCGGTGCGCGAGCGAGCGCGCATCCCCCGATGCGTCCGATGCGCACGCGTGCGCAGATTCCGCGAGCCGATCG

CGAGCCTGGCTTATCCGGCTTATCCGGCTTATCCGGCTCGTCTGGCGCGTCCGGCGCGTCCGGCGCGTCCGGCGTCGCG

AACTTCGATCCGATTTTCTTCTTCTACCATCGCCTGCGTTCGCACGGCAGCCCGCAGCAGGCAAGCCCGACACAGGCCC

GACGCACGAAGCGCGATCGCGCGGCAGCGTAG

>Bucl7_Bps_MSHR511.seq Reverse Complement DNA Sequence CP004024.seq(1,585)

ATGGACACGACCACGCAGGACGGGAACCGGCCGCCGTTCGACGCGCCGCGAGGCGGGCGCGGAGACGGCGACAAGAACG

ACGACACGCGACTCCCCGATTGCAACGAACGCGCGCGGCGGCGCGCCGGCGTCGCGGCGAAGCGCACGCGCCGCGCCCT

GAACGGCGCGGCGAGCCGCACCGGCCTGGACGCCGAATGCCGCGTTCGTCCGAAGAACGCGCGACGTCGCACGAGCGTT

TCGAAAGCGGCGCTGGCCGTTCATTGGCGCGCCCGACGCCGCGCGATCGTCCCGATTCACGCGGCCCAGCCGAAGGGCG

GACGTGGCGGTGCGCGAGCGAGCGCGCATCCCCCGATGCGTCCGATGCGCACGCGTGCGCAGATTCCGCGAGCCGATCG

CGAGCCTGGCTTATCCGGCTTATCCGGCTTATCCGGCTCGTCTGGCGCGTCCGGCGCGTCCGGCGCGTCCGGCGTCGCG

AACTTCGATCCGATTTTCTTCTTCTACCATCGCCTGCGTTCGCACGGCAGCCCGCAGCAGGCAAGCCCGACACAGGCCC

GACGCACGAAGCGCGATCGCGCGGCAGCGTAG

>Bucl7_Bps_MSHR520.seq >gi|589303173|gb|CP004369.1|:3012374-3012958 Burkholderia pseudomallei MSHR520 chromosome 2, complete sequence

ATGGACACGACCACGCAGGACGGGAACCGGCCGCCGTTCGACGCGCCGCGAGGCGGGCGCGGAGACGGCGACAAGAACG

ACGACACGCGATTCCCCGATTGCAACGAACGCGCGCGGCGGCGCGCCGGCGTCGCGGCGAAGCGCACGCGCCGCGCCCT

GAACGGCGCGGCGAGCCGCACCGGCCTGGACGCCGAATGCCGCGTTCGTCCGAAGAACGCGCGACGTCGCACGAGCGTT

TCGAAAGCGGCGCGGGCCGTTCATTGGCGCGCCCGACGCCGCGCGATCGTCCCGATTCACGCGGCCCAGCCGAAGGGCG

GACGTGGCGGTGCGCGAGCGAGCGCGCATCCCCCGATGCGTCCGATGCGCACGCGTGCGCAGATTCCGCGAGCCGATCG

CGAGCCTGGCTTATCCGGCTTATCCGGCTTATCCGGCGCGTCCGGCGCGTCCGGCGCGTCCGGCGCGTCCGGCGTCGCG

AACTTCGATCCGATTTTCTTCTTCTACCATCGCCTGCGTTCGCACGGCAGCCCGCAGCAAGCAAGCCCGACACAGGCCC

GACGCACGAAGCGCGATCGCGCGACAGCGTAG

>Bucl7_Bps_NAU20B-16.seq Reverse Complement DNA Sequence CP004004.seq(1,585)

ATGGACACGACCACGCAGGACGGGAACCGGCCGCCGTTCGACGCGCCGCGAGGCGGGCGCGGAGACGGCGACAAGAACG

ACGACACGCGACTCCCCGATTGCAACGAACGCGCGCGGCGGCGCGCCGGCGTCGCGGCGAAGCGCACGCGCCGCGCCCT

GAACGGCGCGGCGAGCCGCACCGGCCTGGACGCCGAATGCCGCGTTCGTCCGAAGAACGCGCGACGTCGCACGAGCGTT

TCGAAAGCGGCGCTGGCCGTTCATTGGCGCGCCCGACGCCGCGCGATCGTCCCGATTCACGCGGCCCAGCCGAAGGGCG

GACGTGGCGGTGCGCGAGCGAGCGCGCATCCCCCGATGCGTCCGATGCGCACGCGTGCGCAGATTCCGCGAGCCGATCG

CGAGCCTGGCTTATCCGGCTTATCCGGCTTATCCGGCTCGTCTGGCGCGTCCGGCGCGTCCGGCGCGTCCGGCGTCGCG

AACTTCGATCCGATTTTCTTCTTCTACCATCGCCTGCGTTCGCACGGCAGCCCGCAGCAGGCAAGCCCGACACAGGCCC

GACGCACGAAGCGCGATCGCGCGGCAGCGTAG

>Bucl7_Bps_NCTC_13178.seq >gi|568798707|gb|CP004002.1|:1312091-1312684 Burkholderia pseudomallei NCTC 13178 chromosome 2, complete sequence

ATGGACACGACCACGCAGGACGGGAACCGGCCGCCGTTCGACGCGCCGCGAGGCGGGCGCGGAGACGGCGACAAGAACG

ACGACACGCGACTCCCCGATTGCAACGAACGCGCGCGGCGGCGCGCCGGCGTCGCGGCGAAGCGCACGCGCCGCGCCCT

GAACGGCGCGGCGAGCCGCACCGGCCTGGACGCCGAATGCCGCGTTCGTCCGAAGAACGCGCGACGTCGCACGAGCGTT

TCGAAAGCGGCGCGAGCCGTTCATTGGCGCGCCCGACGCCGCGCGATCGTCCCGATTCACGCGGCCCAGCCGAAGGGCG

GACGTGGCGGTGCGCGAGCGAGCGCGCATCCCCCGATGCGTCCGATGCGCACGCGTGCGCAGATTCCGCGAGCCGATCG

CGAGCCTGGCTTATCCGGCTTATCCGGCTCGTCCGGCTCGTCCGGCTCGTCCGGCGCGTCCGGCGCGTCCGGCGCGTCC

GGCGTCGCGAACTTCGATCCGATTTTCTTCTTCTACCATCGCCTGCGTTCGCACGGCAGCCCGCAGCAGGCAAGCCCGA

CACAGGCCCGACGCACGAAGCGCGATCGCGCGGCAGCGTAG

>Bucl7_Bm_2000031281.seq Created: Tuesday, March 25, 2014 10:42 PM

ATGGACACGACCACGCAGGACGGGAATCGGCCGCCGTTCGACGCGCCGCGAGGCGGGCGCGGAGACGGCGACAAGAACG

ACGACACGCGACTCCCCGATTGCAACGAACGCGCGCGGCGGCGCGCCGGCGTCGCGGCGAAGCGCACGCGCCGCGCCCT

GAACGGCGCGGCGAGCCGCACCGGCCTGGACGCCGAATGCCGCGTTCGTCCGAAGAACGCGCGACGTCGCACGAGCGTT

TCGAAAGCGACGCGGGCCGTTCATTGGCGCGCCCGACGCCGCGCGATCGTCCCGATTCACGCGGCCCAGCCGAAGGGCG

GACGTGGCGGTGCGCGAGCGAGCGCGCATCCCCCGATGCGTCCGATGCGCACGCGTGCGCAGATTCCGCGAGCCGATCG

CGAGCCTGGCTTATCCGGCTTATCCGGCTCGTCTGGCGCGTCCGGCGCGTCCGGCGCGTCCGGCGTCGCGAACTTCGAT

CCGATTTTCTTCTTCTACCATCGCCTGCGTTCGCACGGCAGCCCGCAGCAGGCAAGCCCGACACAGGCCCGACGCACGA

AGCGCGATCGCGCGGCAGCGTAG

>Bucl7_Bm_2002721280.seq Created: Tuesday, March 25, 2014 11:01 PM

ATGGACACGACCACGCAGGACGGGAATCGGCCGCCGTTCGACGCGCCGCGAGGCGGGCGCGGAGACGGCGACAAGAACG

ACGACACGCGACTCCCCGATTGCAACGAACGCGCGCGGCGGCGCGCCGGCGTCGCGGCGAAGCGCACGCGCCGCGCCCT

GAACGGCGCGGCGAGCCGCACCGGCCTGGACGCCGAATGCCGCGTTCGTCCGAAGAACGCGCGACGTCGCACGAGCGTT

TCGAAAGCGACGCGGGCCGTTCATTGGCGCGCCCGACGCCGCGCGATCGTCCCGATTCACGCGGCCCAGCCGAAGGGCG

GACGTGGCGGTGCGCGAGCGAGCGCGCATCCCCCGATGCGTCCGATGCGCACGCGTGCGCAGATTCCGCGAGCCGATCG

CGAGCCTGGCTTATCCGGCTTATCCGGCTCGTCTGGCGCGTCCGGCGCGTCCGGCGCGTCCGGCGTCGCGAACTTCGAT

CCGATTTTCTTCTTCTACCATCGCCTGCGTTCGCACGGCAGCCCGCAGCAGGCAAGCCCGACACAGGCCCGACGCACGA

AGCGCGATCGCGCGGCAGCGTAG

>Bucl7_Bm_A188.seq Created: Tuesday, March 25, 2014 10:39 PM

ATGGACACGACCACGCAGGACGGGAATCGGCCGCCGTTCGACGCGCCGCGAGGCGGGCGCGGAGACGGCGACAAGAACG

ACGACACGCGACTCCCCGATTGCAACGAACGCGCGCGGCGGCGCGCCGGCGTCGCGGCGAAGCGCACGCGCCGCGCCCT

GAACGGCGCGGCGAGCCGCACCGGCCTGGACGCCGAATGCCGCGTTCGTCCGAAGAACGCGCGACGTCGCACGAGCGTT

TCGAAAGCGACGCGGGCCGTTCATTGGCGCGCCCGACGCCGCGCGATCGTCCCGATTCACGCGGCCCAGCCGAAGGGCG

GACGTGGCGGTGCGCGAGCGAGCGCGCATCCCCCGATGCGTCCGATGCGCACGCGTGCGCAGATTCCGCGAGCCGATCG

CGAGCCTGGCTTATCCGGCTTATCCGGCTCGTCTGGCGCGTCCGGCGCGTCCGGCGCGTCCGGCGTCGCGAACTTCGAT

CCGATTTTCTTCTTCTACCATCGCCTGCGTTCGCACGGCAGCCCGCAGCAGGCAAGCCCGACACAGGCCCGACGCACGA

AGCGCGATCGCGCGGCAGCGTAG

>Bucl7_Bm_China_7.seq Created: Tuesday, March 25, 2014 10:50 PM

ATGGACACGACCACGCAGGACGGGAATCGGCCGCCGTTCGACGCGCCGCGAGGCGGGCGCGGAGACGGCGACAAGAACG

ACGACACGCGACTCCCCGATTGCAACGAACGCGCGCGGCGGCGCGCCGGCGTCGCGGCGAAGCGCACGCGCCGCGCCCT

GAACGGCGCGGCGAGCCGCACCGGCCTGGACGCCGAATGCCGCGTTCGTCCGAAGAACGCGCGACGTCGCACGAGCGTT

TCGAAAGCGACGCGGGCCGTTCATTGGCGCGCCCGACGCCGCGCGATCGTCCCGATTCACGCGGCCCAGCCGAAGGGCG

GACGTGGCGGTGCGCGAGCGAGCGCGCATCCCCCGATGCGTCCGATGCGCACGCGTGCGCAGATTCCGCGAGCCGATCG

CGAGCCTGGCTTATCCGGCTTATCCGGCTCGTCTGGCGCGTCCGGCGCGTCCGGCGCGTCCGGCGTCGCGAACTTCGAT

CCGATTTTCTTCTTCTACCATCGCCTGCGTTCGCACGGCAGCCCGCAGCAGGCAAGCCCGACACAGGCCCGACGCACGA

AGCGCGATCGCGCGGCAGCGTAG

>Bucl7_Bm_PRL-20.seq Created: Tuesday, March 25, 2014 10:59 PM

ATGGACACGACCACGCAGGACGGGAATCGGCCGCCGTTCGACGCGCCGCGAGGCGGGCGCGGAGACGGCGACAAGAACG

ACGACACGCGACTCCCCGATTGCAACGAACGCGCGCGGCGGCGCGCCGGCGTCGCGGCGAAGCGCACGCGCCGCGCCCT

GAACGGCGCGGCGAGCCGCACCGGCCTGGACGCCGAATGCCGCGTTCGTCCGAAGAACGCGCGACGTCGCACGAGCGTT

TCGAAAGCGACGCGGGCCGTTCATTGGCGCGCCCGACGCCGCGCGATCGTCCCGATTCACGCGGCCCAGCCGAAGGGCG

GACGTGGCGGTGCGCGAGCGAGCGCGCATCCCCCGATGCGTCCGATGCGCACGCGTGCGCAGATTCCGCGAGCCGATCG

CGAGCCTGGCTTATCCGGCTTATCCGGCTCGTCTGGCGCGTCCGGCGCGTCCGGCGCGTCCGGCGTCGCGAACTTCGAT

CCGATTTTCTTCTTCTACCATCGCCTGCGTTCGCACGGCAGCCCGCAGCAGGCAAGCCCGACACAGGCCCGACGCACGA

AGCGCGATCGCGCGGCAGCGTAG

>Bucl7_Bm_strain_6.seq Created: Tuesday, March 25, 2014 10:47 PM

ATGGACACGACCACGCAGGACGGGAATCGGCCGCCGTTCGACGCGCCGCGAGGCGGGCGCGGAGACGGCGACAAGAACG

ACGACACGCGACTCCCCGATTGCAACGAACGCGCGCGGCGGCGCGCCGGCGTCGCGGCGAAGCGCACGCGCCGCGCCCT

GAACGGCGCGGCGAGCCGCACCGGCCTGGACGCCGAATGCCGCGTTCGTCCGAAGAACGCGCGACGTCGCACGAGCGTT

TCGAAAGCGACGCGGGCCGTTCATTGGCGCGCCCGACGCCGCGCGATCGTCCCGATTCACGCGGCCCAGCCGAAGGGCG

GACGTGGCGGTGCGCGAGCGAGCGCGCATCCCCCGATGCGTCCGATGCGCACGCGTGCGCAGATTCCGCGAGCCGATCG

CGAGCCTGGCTTATCCGGCTTATCCGGCTCGTCTGGCGCGTCCGGCGCGTCCGGCGCGTCCGGCGTCGCGAACTTCGAT

CCGATTTTCTTCTTCTACCATCGCCTGCGTTCGCACGGCAGCCCGCAGCAGGCAAGCCCGACACAGGCCCGACGCACGA

AGCGCGATCGCGCGGCAGCGTAG

>Bucl7_Bm_strain_11.seq Reverse Complement DNA Sequence Untitled Seq #23(1,576)

ATGGACACGACCACGCAGGACGGGAATCGGCCGCCGTTCGACGCGCCGCGAGGCGGGCGCGGAGACGGCGACAAGAACG

ACGACACGCGACTCCCCGATTGCAACGAACGCGCGCGGCGGCGCGCCGGCGTCGCGGCGAAGCGCACGCGCCGCGCCCT

GAACGGCGCGGCGAGCCGCACCGGCCTGGACGCCGAATGCCGCGTTCGTCCGAAGAACGCGCGACGTCGCACGAGCGTT

TCGAAAGCGACGCGGGCCGTTCATTGGCGCGCCCGACGCCGCGCGATCGTCCCGATTCACGCGGCCCAGCCGAAGGGCG

GACGTGGCGGTGCGCGAGCGAGCGCGCATCCCCCGATGCGTCCGATGCGCACGCGTGCGCAGATTCCGCGAGCCGATCG

CGAGCCTGGCTTATCCGGCTTATCCGGCTCGTCTGGCGCGTCCGGCGCGTCCGGCGCGTCCGGCGTCGCGAACTTCGAT

CCGATTTTCTTCTTCTACCATCGCCTGCGTTCGCACGGCAGCCCGCAGCAGGCAAGCCCGACACAGGCCCGACGCACGA

AGCGCGATCGCGCGGCAGCGTAG

>Bucl7_Bm_FMH.seq Created: Tuesday, March 25, 2014 11:05 PM

ATGGACACGACCACGCAGGACGGGAATCGGCCGCCGTTCGACGCGCCGCGAGGCGGGCGCGGAGACGGCGACAAGAACG

ACGACACGCGACTCCCCGATTGCAACGAACGCGCGCGGCGGCGCGCCGGCGTCGCGGCGAAGCGCACGCGCCGCGCCCT

GAACGGCGCGGCGAGCCGCACCGGCCTGGACGCCGAATGCCGCGTTCGTCCGAAGAACGCGCGACGTCGCACGAGCGTT

TCGAAAGCGACGCGGGCCGTTCATTGGCGCGCCCGACGCCGCGCGATCGTCCCGATTCACGCGGCCCAGCCGAAGGGCG

GACGTGGCGGTGCGCGAGCGAGCGCGCATCCCCCGATGCGTCCGATGCGCACGCGTGCGCAGATTCCGCGAGCCGATCG

CGAGCCTGGCTTATCCGGCTTATCCGGCTCGTCTGGCGCGTCCGGCGCGTCCGGCGCGTCCGGCGTCGCGAACTTCGAT

CCGATTTTCTTCTTCTACCATCGCCTGCGTTCGCACGGCAGCCCGCAGCAGGCAAGCCCGACACAGGCCCGACGCACGA

AGCGCGATCGCGCGGCAGCGTAG

>Bucl7_Bm_JHU.seq Created: Tuesday, March 25, 2014 11:03 PM

ATGGACACGACCACGCAGGACGGGAATCGGCCGCCGTTCGACGCGCCGCGAGGCGGGCGCGGAGACGGCGACAAGAACG

ACGACACGCGACTCCCCGATTGCAACGAACGCGCGCGGCGGCGCGCCGGCGTCGCGGCGAAGCGCACGCGCCGCGCCCT

GAACGGCGCGGCGAGCCGCACCGGCCTGGACGCCGAATGCCGCGTTCGTCCGAAGAACGCGCGACGTCGCACGAGCGTT

TCGAAAGCGACGCGGGCCGTTCATTGGCGCGCCCGACGCCGCGCGATCGTCCCGATTCACGCGGCCCAGCCGAAGGGCG

GACGTGGCGGTGCGCGAGCGAGCGCGCATCCCCCGATGCGTCCGATGCGCACGCGTGCGCAGATTCCGCGAGCCGATCG

CGAGCCTGGCTTATCCGGCTTATCCGGCTCGTCTGGCGCGTCCGGCGCGTCCGGCGCGTCCGGCGTCGCGAACTTCGAT

CCGATTTTCTTCTTCTACCATCGCCTGCGTTCGCACGGCAGCCCGCAGCAGGCAAGCCCGACACAGGCCCGACGCACGA

AGCGCGATCGCGCGGCAGCGTAG

>Bucl7_Bm_ATCC_10399.seq Reverse Complement DNA Sequence Untitled Seq #48(1,585)

ATGGACACGACCACGCAGGACGGGAATCGGCCGCCGTTCGACGCGCCGCGAGGCGGGCGCGGAGACGGCGACAAGAACG

ACGACACGCGACTCCCCGATTGCAACGAACGCGCGCGGCGGCGCGCCGGCGTCGCGGCGAAGCGCACGCGCCGCGCCCT

GAACGGCGCGGCGAGCCGCACCGGCCTGGACGCCGAATGCCGCGTTCGTCCGAAGAACGCGCGACGTCGCACGAGCGTT

TCGAAAGCGACGCGGGCCGTTCATTGGCGCGCCCGACGCCGCGCGATCGTCCCGATTCACGCGGCCCAGCCGAAGGGCG

GACGTGGCGGTGCGCGAGCGAGCGCGCATCCCCCGATGCGTCCGATGCGCACGCGTGCGCAGATTCCGCGAGCCGATCG

CGAGCCTGGCTTATCCGGCTTATCCGGCTCGTCTGGCGCGTCCGGCGCGTCCGGCGCGTCCGGCGCGTCCGGCGTCGCG

AACTTCGATCCGATTTTCTTCTTCTACCATCGCCTGCGTTCGCACGGCAGCCCGCAGCAGGCAAGCCCGACACAGGCCC

GACGCACGAAGCGCGATCGCGCGGCAGCGTAG

>Bucl7_Bm_GB8_horse_4.seq Created: Tuesday, March 25, 2014 11:07 PM

ATGGACACGACCACGCAGGACGGGAATCGGCCGCCGTTCGACGCGCCGCGAGGCGGGCGCGGAGACGGCGACAAGAACG

ACGACACGCGACTCCCCGATTGCAACGAACGCGCGCGGCGGCGCGCCGGCGTCGCGGCGAAGCGCACGCGCCGCGCCCT

GAACGGCGCGGCGAGCCGCACCGGCCTGGACGCCGAATGCCGCGTTCGTCCGAAGAACGCGCGACGTCGCACGAGCGTT

TCGAAAGCGACGCGGGCCGTTCATTGGCGCGCCCGACGCCGCGCGATCGTCCCGATTCACGCGGCCCAGCCGAAGGGCG

GACGTGGCGGTGCGCGAGCGAGCGCGCATCCCCCGATGCGTCCGATGCGCACGCGTGCGCAGATTCCGCGAGCCGATCG

CGAGCCTGGCTTATCCGGCTTATCCGGCTCGTCTGGCGCGTCCGGCGCGTCCGGCGCGTCCGGCGTCGCGAACTTCGAT

CCGATTTTCTTCTTCTACCATCGCCTGCGTTCGCACGGCAGCCCGCAGCAGGCAAGCCCGACACAGGCCCGACGCACGA

AGCGCGATCGCGCGGCAGCGTAG

>Bucl8_Bps_K96243.seq Created: Monday, January 20, 2014 11:47 AM

ttgcaccattctcggtttattcggcaatccgcaaaaaaatataatcgcatcgactcatctttatccgaaagggagaaaa

tcgtgcagtctccggcaatgagagggacgcttgcactcgcggttcttgcaatctcattaataatggccggatgcgcaag

catgggcgatgttgcaccgcaagacaagcaggtcgatccggcgtcgctcgatgcgggcgcggcgatcgcggcggccgag

cgcgacgccggctggcccgcggccgactggtggcgcgcgtatcgcgatccgcagctcgattcgtggatcgccgcgtcgc

tcgccggcaacccttcactcgccgccgcgcaggcccgcgtgcgcgaggcgcagtcgctcgcgcgcatcgcgcacgccga

ggagctgccgcagttgaacggcaacctgtcgctgatgcgccagcactggccggacaacgtctattacggcccgggcccg

ctcgcgaacgccgacacgtggaacaacacgggcacgctgagcctgtcgtaccacctcgatctgtggggcaaggacaagc

acaacgccgagcgcgcgctcgacgcggcgcgcgcccgcgcggccgacgcgcgtgccgcgcagctcgagctcgaggcgaa

cgtcgtgcgcgcgtacgtcgatttcgcgaagaactacgcgctcctcgacatcgcgcacgcgacctacgagcgccagaac

gcgctcgccgaactggcgcgcaagcgcctgcgcgcgggcatcggcacgcaactcgaggtgagccaggccgaggcgccgc

tgcccgattattcgcggcagatcgactcgtatgaagaggcgatccagctcggccgtcaccagctcgccgcgctcgcggg

caaggggccgggcgcgggcgcctcgctcgcgcggccgaagctcgcgctcgacgcgaacgccgccctgccgtccgcgctg

cccgccgagctgctcggccgccggccggacatcgtcgccgcgcgctggatggtcgacgcgcaggcgcgcggcatcgacg

tcgcgaaggccgcgttctatccgaacgtcgacctgatcgcgtcgctcggcggcttcgcggtcagcgcgccgttcgcgac

gttcctgcgcgcgatgaacggcggctggtcggccggccccgcgctcacgctgccgatcttcgagggcggccggctgcgc

gcgcaactgggcgtcgcgtcggcgggctacgaccaggcggtcgagcactacaaccagacgatcgtcggcgcgctgaagg

acatcgccgacaacgtcgtgcgcctgcattcgctcgattcgcagcaaaaggacgcggcgcgcgcggtctcgctcacgcg

gcgctcgtacgatctgtcgcatgcgggcttcagccgcgggctcaccgactacgtgaacgtgctcatcgcgcagagccag

ttgctgaccgcgcaggagaaccagacccgcatcgaggccgcgcgcctcgccgcgcacgcgtcgctgatggtcgcgctcg

gcggcgggctcgaaaccgggcgcgacgcgccgcacgacgcgcccgcgggcgacgcgcggcggacgggtgcttcgggtgc

ttcgggtgcttcgcgtgcttcgcgtgcttcgcgtgcttcgggtgcttcgggtgcttcgggtgcctcgggtgcctcgggt

gcctcgggtgcctcgggtgcctcgggtgcctcgggtgcctcgggtgcctcgggtgcctcgggtgcctcgggtgcctcgg

gtgcctcgggtgcctcgggtgcctcgggtgcctcgggtgcctcgggtgcctcgggtgcctcgggtgcttcgagcaccgc

gggcgcttcggccaccgcatccgcgtcggccgccggccacgcgccggctggcgccgctgctccggcatcgcccgccggc

atccgcgcggccgccagcgctcgcgcgtcaatgccggctcccgccgcggcggcgaccgcgcccgccttcgcgtcgcccg

tcgccggcgcctcgacgccgatgcccgctgcgacagcggcggcccgcgccgcccgctaa

>A4LQJ4_BURPS_Bucl8_Bps_305.seq Created: Friday, August 02, 2013 11:10 AM

TTGCACCATTCTCGGTTTATTCGGCAATCCGCAAAAAAATATAATCGCATCGACTCATCTTTATCCGAAAGGGAGAAAA

TCGTGCAGTCTCCGGCAATGAGAGGGACGCTTGCACTCGCGGTTCTTGCAATCTCATTAATAATGGCCGGATGCGCAAG

CATGGGCGATGTTGCACCGCAAGACAAGCAGGTCGATCCGGCGTCGCTCGACGCGGGCGCGGCGATCGCGGCGGCCGAG

CGCGACGCCGGCTGGCCCGCGGCCGACTGGTGGCGCGCGTATCGCGATCCGCAGCTCGATTCGTGGATCGCCGCGTCGC

TCGCCGGCAACCCTTCGCTCGCCGCCGCGCAGGCCCGCGTGCGCGAGGCGCAGTCGCTCGCGCGCATCGCGCACGCCGA

GGAACTGCCGCAGTTGAACGGCAACCTGTCGCTGATGCGCCAGCACTGGCCGGACAACGTCTATTACGGCCCGGGCCCG

CTCGCGAACGCCGACACGTGGAACAACACGGGCACGCTGAGCCTGTCGTACCACCTCGATCTGTGGGGCAAGGACAAGC

ACAACGCCGAGCGCGCGCTCGACGCGGCGCGCGCCCGCGCGGCCGACGCGCGTGCCGCGCAGCTCGAGCTCGAGGCGAA

CGTCGTGCGCGCGTACGTCGATTTCGCGAAGAACTACGCGCTCCTCGACATCGCGCACGCGACCTACGAGCGCCAGAAC

GCGCTCGCCGAACTGGCGCGCAAGCGCCTGCGCGCGGGCATCGGCACGCAACTCGAGGTGAGCCAGGCCGAGGCGCCGC

TGCCCGATTATTCGCGGCAGATCGACTCGTATGAAGAGGCGATCCAGCTCGGCCGTCACCAGCTCGCCGCGCTCGCGGG

CAAGGGGCCGGGCGCGGGCGCCTCGCTCGCGCGGCCGAAGCTCGCGCTCGACGCGAACGCCGCCCTGCCGTCCGCGCTG

CCCGCCGAGCTGCTCGGCCGCCGGCCGGACATCGTCGCCGCGCGCTGGATGGTCGACGCGCAGGCGCGCGGCATCGACG

TCGCGAAGGCCGCGTTCTATCCGAACGTCGACCTGATCGCGTCGCTCGGCGGCTTCGCGGTCAGCGCGCCGTTCGCGAC

GTTCCTGCGCGCGATGAACGGCGGCTGGTCGGCCGGCCCCGCGCTCACGCTGCCGATCTTCGAGGGCGGCCGGCTGCGC

GCGCAACTGGGCGTCGCGTCGGCGGGCTACGACCAGGCGGTCGAGCACTACAACCAGACGATCGTCGGCGCGCTGAAGG

ACATCGCCGACAACGTCGTGCGCCTGCATTCGCTCGATTCGCAGCAAAAGGACGCGGCGCGCGCGGTCTCGCTCACGCG

GCGCTCGTACGATCTGTCGCATGCGGGCTTCAGCCGCGGGCTCACCGATTACGTGAACGTGCTCATCGCGCAGAGCCAG

TTGCTGACCGCGCAGGAGAACCAGACCCGCATCGAGGCCGCGCGCCTCGCCGCGCACGCGTCGCTGATGGTCGCGCTCG

GCGGCGGGCTCGAAACCGGGCACGACGCGCCCGCGGGCGACGCGCGGCGGACGGGTGCTTCGGGTGCTTCGGGTGCTTC

GGGTGCTTCGGGTGCTTCGGGTGCTTCGGGCGCTTCGGGCGCTTCGGGCGCTTCGGGTGCTTCAGGCGCTTCAGGCACC

GCGGGCGCTTCGGCCGCCGCATCCGCGTCGGCCGCCGGCCACGCGCCGGCTGGCGCCGCTGCTCCGGCATCGCCCGCCG

GCATCCGCGCGGCCGCCAGCGCTCGCGCGTCAATGCCGGCTCCCGCCGCGGCGGCGACCGCGCCCGCCTTCGCGTCGCC

CGTCGCCGGCGCCTCGACGCCGATGCCCGCTGCGACAGCGGCGGCCCGCGCCGCCCGCTAA

>Bucl8_Bps_1106a.seq Created: Monday, January 20, 2014 11:33 AM

ttgcaccattctcggtttattcggcaatccgcaaaaaaatataatcgcatcgactcatctttatccgaaagggagaaaa

tcgtgcagtctccggcaatgagagggacgcttgcactcgcggttcttgcaatctcattaataatggccggatgcgcaag

catgggcgatgttgcaccgcaagacaagcaggtcgatccggcgtcgctcgatgcgggcgcggcgatcgcggcggccgag

cgcgacgccggctggcccgcggccgactggtggcgcgcgtatcgcgatccgcagctcgattcgtggatcgccgcgtcgc

tcgccggcaacccttcgctcgccgccgcgcaggcccgcgtgcgcgaggcgcagtcgctcgcgcgcatcgcgcacgccga

ggagctgccgcagttgaacggcaacctgtcgctgatgcgccagcactggccggacaacgtctattacggcccgggcccg

ctcgcgaacgccgacacgtggaacaacacgggcacgctgagcctgtcgtaccacctcgatctgtggggcaaggacaagc

acaacgccgagcgcgcgctcgacgcggcgcgcgcccgcgcggccgacgcgcgtgccgcgcagctcgagctcgaggcgaa

cgtcgtgcgcgcgtacgtcgatttcgcgaagaactacgcgctcctcgacatcgcgcacgcgacctacgagcgccagaac

gcgctcgccgaactggcgcgcaagcgcctgcgcgcgggcatcggcacgcaactcgaggtgagccaggccgaggcgccgc

tgcccgattattcgcggcagatcgactcgtatgaagaggcgatccagctcggccgtcaccagctcgccgcgctcgcggg

caaggggccgggcgcgggcgcctcgctcgcgcggccgaagctcgcgctcgacgcgaacgccgccctgccgtccgcgctg

cccgccgagctgctcggccgccggccggacatcgtcgccgcgcgctggatggtcgacgcgcaggcgcgcggcatcgacg

tcgcgaaggccgcgttctatccgaacgtcgacctgatcgcgtcgctcggcggcttcgcggtcagcgcgccgttcgcgac

gttcctgcgcgcgatgaacggcggctggtcggccggccccgcgctcacgctgccgatcttcgagggcggccggctgcgc

gcgcaactgggcgtcgcgtcggcgggctacgaccaggcggtcgagcactacaaccagacgatcgtcggcgcgctgaagg

acatcgccgacaacgtcgtgcgcctgcattcgctcgattcgcagcaaaaggacgcggcgcgcgcggtctcgctcacgcg

gcgctcgtacgatctgtcgcatgcgggcttcagccgcgggctcaccgactacgtgaacgtgctcatcgcgcagagccag

ttgctgaccgcgcaggagaaccagacccgcatcgaggccgcgcgcctcgccgcgcacgcgtcgctgatggtcgcgctcg

gcggcgggctcgaaaccgggcgcgacgcgccgcacgacgcgcccgcgggcgacgcgcggcggacgggtgcttcgggtgc

ctcgggtgcctcgggtgcctcgggtgcctcgggtgcctcgggtgcttcgagcaccgcgggcgcttcggccaccgcatcc

gcgtcggccgccggccacgcgccggctggcgccgctgctccggcatcgcccgccggcatccgcgcggccgccagcgctc

gcgcgtcaatgccggctcccgccgcggcggcgaccgcgcccgccttcgcgtcgcccgtcgccggcgcctcgacgccgat

gcccgctgcgacagcggcggcccgcgccgcccgctaa

>Bucl8_Bps_MSHR_346.seq Reverse Complement DNA Sequence Untitled Seq #1(1,1860)

TTGCACCATTCTCGGTTTATTCGGCAATCCGCAAAAAAATATAATCGCATCGACTCATCTTTATCCGAAAGGGAGAAAA

TCGTGCAGTCTCCGGCAATGAGAGGGACGCTTGCACTCGCGGTTCTTGCAATCTCATTAATAATGGCCGGATGCGCAAG

CATGGGCGATGTTGCACCGCAAGACAAGCAGGTCGATCCGGCGTCGCTCGACGCGGGCGCGGCGATCGCGGCGGCCGAG

CGCGACGCCGGCTGGCCCGCGGCCGACTGGTGGCGCGCGTATCGCGATCCGCAGCTCGATTCGTGGATCGCCGCGTCGC

TCGCCGGCAACCCTTCGCTCGCCGCCGCGCAGGCCCGCGTGCGCGAGGCGCAGTCGCTCGCGCGCATCGCGCACGCCGA

GGAGCTGCCGCAGTTGAACGGCAACCTGTCGCTGATGCGCCAGCACTGGCCGGACAACGTCTATTACGGCCCGGGCCCG

CTCGCGAACGCCGACACGTGGAACAACACGGGCACGCTGAGCCTGTCGTACCACCTCGATCTGTGGGGCAAGGACAAGC

ACAACGCCGAGCGCGCGCTCGACGCGGCGCGCGCCCGCGCGGCCGACGCGCGTGCCGCGCAGCTCGAGCTCGAGGCGAA

CGTCGTGCGCGCGTACGTCGATTTCGCGAAGAACTACGCGCTCCTCGACATCGCGCACGCGACCTACGAGCGCCAGAAC

GCGCTCGCCGAACTGGCGCGCAAGCGCCTGCGCGCGGGCATCGGCACGCAACTCGAGGTGAGCCAGGCCGAGGCGCCGC

TGCCCGATTATTCGCGGCAGATCAACTCGTATGAAGAGGCGATCCAGCTCGGCCGTCACCAGCTCGCCGCGCTCGCGGG

CAAGGGGCCGGGCGCGGGCGCCTCGCTCGCGCGGCCGAAGCTCGCGCTCGACGCGAACGCCGCCCTGCCGTCCGCGCTG

CCCGCCGAGCTGCTCGGCCGCCGGCCGGACATCGTCGCCGCGCGCTGGATGGTCGACGCGCAGGCGCGCGGCATCGACG

TCGCGAAGGCCGCGTTCTATCCGAACGTCGACCTGATCGCGTCGCTCGGCGGCTTCGCGGTCAGCGCGCCGTTCGCGAC

GTTCCTGCGCGCGATGAACGGCGGCTGGTCGGCCGGCCCCGCGCTCACGCTGCCGATCTTCGAGGGCGGCCGGCTGCGC

GCGCAACTGGGCGTCGCGTCGGCGGGCTACGACCAGGCGGTCGAGCACTACAACCAGACGATCGTCGGCGCGCTGAAGG

ACATCGCCGACAACGTCGTGCGCCTGCATTCGCTCGATTCGCAGCAAAAGGACGCGGCGCGCGCGGTCTCGCTCACGCG

GCGCTCGTACGATCTGTCGCATGCGGGCTTCAGCCGCGGGCTCACCGATTACGTGAACGTGCTCATCGCGCAGAGCCAG

TTGCTGACCGCGCAGGAGAACCAGACACGCATCGAGGCCGCGCGCCTCGCCGCGCACGCGTCGCTGATGGTCGCGCTCG

GCGGCGGGCTCGAAACCGGGCACGACGCGCCCGCGGGCGACGCGCGGCGGACGGGTGCTTCGGGTGCTTCGGGTGCTTC

GGGTGCTTCGGGTGCTTCGGGCGCTTCGGGCGCTTCGGGTGCTTCAGGCGCTTCAGGCACCGCGGGCGCTTCGGCCACC

GCATCCGCGTCGGCCGCCGGCCACGCGCCGGCTGGCGCCGCTGCTCCGGCATCGCCCGCCGGCATCCGCGCGGCCGCCA

GCGCTCGCGCGTCAATGCCGGCTCCCGCCGCGGCGGCGACCGCGCCCGCCTTCGCGTCGCCCGTCGCCGGCGCCTCGAC

GCCGATGCCCGCTGCGACAGCGGCGGCCCGCGCCGCCCGCTAA

>Bucl8_Bps_1026b.seq Created: Monday, January 20, 2014 11:26 AM

ttgcaccattctcggtttattcggcaatccgcaaaaaaatataatcgcatcgactcatctttatccgaaagggagaaaa

tcgtgcagtctccggcaatgagagggacgcttgcactcgcggttcttgcaatctcattaataatggccggatgcgcaag

catgggcgatgttgcaccgcaagacaagcaggtcgatccggcgtcgctcgacgcgggcgcggcgatcgcggcggccgag

cgcgacgccggctggcccgcggccgactggtggcgcgcgtatcgcgatccgcagctcgattcgtggatcgccgcgtcgc

tcgccggcaacccttcgctcgccgccgcgcaggcccgcgtgcgcgaggcgcagtcgctcgcgcgcatcgcgcacgccga

ggagctgccgcagttgaacggcaacctgtcgctgatgcgccagcactggccggacaacgtctattacggcccgggcccg

ctcgcgaacgccgacacgtggaacaacacgggcacgctgagcctgtcgtaccacctcgatctgtggggcaaggacaagc

acaacgccgagcgcgcgctcgacgcggcgcgcgcccgcgcggccgacgcgcgtgccgcgcagctcgagctcgaagcgaa

cgtcgtgcgcgcgtacgtcgatttcgcgaagaactacgcgctcctcgacatcgcgcacgcgacctacgagcgccagaac

gcgctcgccgaactggcgcgcaagcgcctgcgcgcgggcatcggcacgcaactcgaggtgagccaggccgaggcgccgc

tgcccgattattcgcggcagatcgactcgtatgaagaggcgatccagctcggccgtcaccagctcgccgcgctcgcggg

caaggggccgggcgcgggcgcctcgctcgcgcggccgaagctcgcgctcgacgcgaacgccgccctgccgtccgcgctg

cccgccgagctgctcggccgccggccggacatcgtcgccgcgcgctggatggtcgacgcgcaggcgcgcggcatcgacg

tcgcgaaggccgcgttctatccgaacgtcgacctgatcgcgtcgctcggcggcttcgcggtcagcgcgccgttcgcgac

gttcctgcgcgcgatgaacggcggctggtcggccggccccgcgctcacgctgccgatcttcgagggcggccggctgcgc

gcgcaactgggcgtcgcgtcggcgggctacgaccaggcggtcgagcactacaaccagacgatcgtcggcgcgctgaagg

acatcgccgacaacgtcgtgcgcctgcattcgctcgattcgcagcaaaaggacgcggcgcgcgcggtctcgctcacgcg

gcgctcgtacgatctgtcgcatgcgggcttcagccgcgggctcaccgattacgtgaacgtgctcatcgcgcagagccag

ttgctgaccgcgcaggagaaccagacccgcatcgaggccgcgcgcctcgccgcgcacgcgtcgctgatggtcgcgctcg

gcggcgggctcgaaaccgggcgcgacgcgccgcacgacgcgcccgcgggcgacgcgcggcggacgggtgcttcgggtgc

ttcgggtgcttcgggtgcttcgggtgcttcgggtgcttcgggtgctgcttcgggtgcttcgagcaccgcgggcgcttcg

gccaccgcatccgcgtcggccgccggccacgcgccggctggcgccgctgctccggcatcgcccgccggcatccgcgcgg

ccgccagcgctcgcgcgtcaatgccggctcccgccgcggcggcgaccgcgcccgccttcgcgtcgcccgtcgccggcgc

ctcgacgccgatgcccgctgcgacagcggcggcccgcgccgcccgctaa

>Bucl8_Bps_1710b.seq Created: Monday, January 20, 2014 11:38 AM

ttgcaccattctcggtttattcggcaatccgcaaaaaaatataatcgcatcgactcatctttatccgaaagggagaaaa

tcgtgcagtctccggcaatgagagggacgcttgcactcgcggttcttgcaatctcattaataatggccggatgcgcaag

catgggcgatgttgcaccgcaagacaagcaggtcgatccggcgtcgctcgatgcgggcgcggcgatcgcggcggccgag

cgcgacgccggctggcccgcggccgactggtggcgcgcgtatcgcgatccgcagctcgattcgtggatcgccgcgtcgc

tcgccggcaacccttcgctcgccgccgcgcaggcccgcgtgcgcgaggcgcagtcgctcgcgcgcatcgcgcacgccga

ggagctgccgcagttgaacggcaacctgtcgctgatgcgccagcactggccggacaacgtctattacggcccgggcccg

ctcgcgaacgccgacacgtggaacaacacgggcacgctgagcctgtcgtaccacctcgatctgtggggcaaggacaagc

acaacgccgagcgcgcgctcgacgcggcgcgcgcccgcgcggccgacgcgcgtgccgcgcagctcgagctcgaggcgaa

cgtcgtgcgcgcgtacgtcgatttcgcgaagaactacgcgctcctcgacatcgcgcacgcgacctacgagcgccagaac

gcgctcgccgaactggcgcgcaagcgcctgcgcgcgggcatcggcacgcaactcgaggtaagccaggccgaagcgccgc

tgcccgattattcgcggcagatcgactcgtatgaagaggcgatccagctcggccgtcaccagctcgccgcgctcgcggg

caaggggccgggcgcgggcgcctcgctcgcgcggccgaagctcgcgctcgacgcgaacgccgccctgccgtccgcgctg

cccgccgagctgctcggccgccggccggacatcgtcgccgcgcgctggatggtcgacgcgcaggcgcgcggcatcgacg

tcgcgaaggccgcgttctatccgaacgtcgacctgatcgcgtcgctcggcggcttcgcggtcagcgcgccgttcgcgac

gttcctgcgcgcgatgaacggcggctggtcggccggccccgcgctcacgctgccgatcttcgagggcggccggctgcgc

gcgcaactgggcgtcgcgtcggcgggctacgaccaggcggtcgagcactacaaccagacgatcgtcggcgcgctgaagg

acatcgccgacaacgtcgtgcgcctgcattcgctcgattcgcagcaaaaggacgcggcgcgcgcggtctcgctcacgcg

gcgctcgtacgatctgtcgcatgcgggcttcagccgcgggctcaccgattacgtgaacgtgctcatcgcgcagagccag

ttgctgaccgcgcaggagaaccagacccgcatcgaggccgcgcgcctcgccgcgcacgcgtcgctgatggtcgcgctcg

gcggcgggctcgaaaccgggcgcgacgcgccgcacgacgcgcccgcgggcgacgcgcggcggacgggtgcttcgggtgc

ttcgggtgcttcgggtgcttcgcgtgcttcgcgtgcttcgggtgcttcgggtgcttcgggtgcttcgggtgcctcgggt

gcctcgggtgcctcgggtgcctcgggtgcctcgggtgcctcgggtgcctcgggtgcttcgagcaccgcgggcgcttcgg

ccaccgcatccgcgtcggccgccggccacgcgccggctggcgccgctgctccggcatcgcccgccggcatccgcgcggc

cgccagcgctcgcgcgtcaatgccggctcccgccgcggcggcgaccgcgcccgccttcgcgtcgcccgtcgccggcgcc

tcgacgccgatgcccgctgcgacagcggcagcccgcgccgcccgctaa

>Bucl8_Bps_BPC006.seq Created: Tuesday, December 31, 2013 3:22 PM

ttgcaccattctcggtttattcggcaatccgcaaaaaaatataatcgcatcgactcatctttatccgaaagggagaaaa

tcgtgcagtctccggcaatgagagggacgcttgcactcgcggttcttgcaatctcattaataatggccggatgcgcaag

catgggcgatgttgcaccgcaagacaagcaggtcgatccggcgtcgctcgatgcgggcgcggcgatcgcggcggccgag

cgcgacgccggctggcccgcggccgactggtggcgcgcgtatcgcgatccgcagctcgattcgtggatcgccgcgtcgc

tcgccggcaacccttcgctcgccgccgcgcaggcccgcgtgcgcgaggcgcagtcgctcgcgcgcatcgcgcacgccga

ggagctgccgcagttgaacggcaacctgtcgctgatgcgccagcactggccggacaacgtctattacggcccgggcccg

ctcgcgaacgccgacacgtggaacaacacgggcacgctgagcctgtcgtaccacctcgatctgtggggcaaggacaagc

acaacgccgagcgcgcgctcgacgcggcgcgcgcccgcgcggccgacgcgcgtgccgcgcagctcgagctcgaggcgaa

cgtcgtgcgcgcgtacgtcgatttcgcgaagaactacgcgctcctcgacatcgcgcacgcgacctacgagcgccagaac

gcgctcgccgaactggcgcgcaagcgcctgcgcgcgggcatcggcacgcaactcgaggtgagccaggccgaggcgccgc

tgcccgattattcgcggcagatcgactcgtatgaagaggcgatccagctcggccgtcaccagctcgccgcgctcgcggg

caaggggccgggcgcgggcgcctcgctcgcgcggccgaagctcgcgctcgacgcgaacgccgccctgccgtccgcgctg

cccgccgagctgctcggccgccggccggacatcgtcgccgcgcgctggatggtcgacgcgcaggcgcgcggcatcgacg

tcgcgaaggccgcgttctatccgaacgtcgacctgatcgcgtcgctcggcggcttcgcggtcagcgcgccgttcgcgac

gttcctgcgcgcgatgaacggcggctggtcggccggccccgcgctcacgctgccgatcttcgagggcggccggctgcgc

gcgcaactgggcgtcgcgtcggcgggctacgaccaggcggtcgagcactacaaccagacgatcgtcggcgcgctgaagg

acatcgccgacaacgtcgtgcgcctgcattcgctcgattcgcagcaaaaggacgcggcgcgcgcggtctcgctcacgcg

gcgctcgtacgatctgtcgcatgcgggcttcagccgcgggctcaccgactacgtgaacgtgctcatcgcgcagagccag

ttgctgaccgcgcaggagaaccagacccgcatcgaggccgcgcgcctcgccgcgcacgcgtcgctgatggtcgcgctcg

gcggcgggctcgaaaccgggcgcgacgcgccgcacgacgcgcccgcgggcgacgcgcggcggacgggtgcttcgggtgc

ttcgggtgcttcgggtgcctcgggtgcctcgggtgcctcgggtgcttcgagcaccgcgggcgcttcggccaccgcatcc

gcgtcggccgccggccacgcgccggctggcgccgctgctccggcatcgcccgccggcatccgcgcggccgccagcgctc

gcgcgtcaatgccggctcccgccgcggcggcgaccgcgcccgccttcgcgtcgcccgtcgccggcgcctcgacgccgat

gcccgctgcgacagcggcagcccgcgccgcccgctaa

>Bucl8_Bps_668.seq Created: Monday, January 20, 2014 11:13 AM

ttgcaccattctcggtttattcggcaatccgcaaaaaaatataatcgcatcgactcatctttatccgaaagggagaaaa

tcgtgcagtctccggcaatgagagggacgcttgcactcgcggttcttgcaatctcattaataatggccggatgcgcaag

catgggcgatgttgcaccgcaagacaagcaggtcgatccggcgtcgctcgacgcgggcgcggcgatcgcggcggccgag

cgcgacgccggctggcccgcggccgactggtggcgcgcgtatcgcgatccgcagctcgattcgtggatcgccgcgtcgc

tcgccggcaacccttcgctcgccgccgcgcaggcccgcgtgcgcgaggcgcagtcgctcgcgcgcatcgcgcacgccga

ggagctgccgcagttgaacggcaacctgtcgctgatgcgccagcactggccggacaacgtctattacggcccgggcccg

ctcgcgaacgccgacacgtggaacaacacgggcacgctgagcctgtcgtaccacctcgatctgtggggcaaggacaagc

acaacgccgagcgcgcgctcgacgcggcgcgcgcccgcgcggccgacgcgcgtgccgcgcagctcgagctcgaggcgaa

cgtcgtgcgcgcgtacgtcgatttcgcgaagaactacgcgctcctcgacatcgcgcacgcgacctacgagcgccagaac

gcgctcgccgaactggcgcgcaagcgcctgcgcgcgggcatcggcacgcaactcgaggtaagccaggccgaagcgccgc

tgcccgattattcgcggcagatcgactcgtatgaagaggcgatccagctcggccgtcaccagctcgccgcgctcgcggg

caaggggccgggcgcgggcgcctcgctcgcgcggccgaagctcgcgctcgacgcaaacgccgccctgccgtccgcgctg

cccgccgagctgctcggccgccggccggacatcgtcgccgcgcgctggatggtcgacgcgcaggcgcgcggcatcgacg

tcgcgaaggccgcgttctatccgaacgtcgacctgatcgcgtcgctcggcggcttcgcggtcagcgcgccgttcgcgac

gttcctgcgcgcgatgaacggcggctggtcggccggccccgcgctcacgctgccgatcttcgagggcggccggctgcgc

gcgcaactgggcgtcgcgtcggcgggctacgaccaggcggtcgagcactacaaccagacgatcgtcggcgcgttgaagg

acatcgccgacaacgtcgtgcgcctgcattcgctcgattcgcagcaaaaggacgcggcgcgcgcggtctcgctcacgcg

gcgctcgtacgatctgtcgcatgcgggcttcagccgcgggctcaccgattacgtgaacgtgctcatcgcgcagagccag

ttgctgaccgcgcaggagaaccagacccgcatcgaggccgcgcgcctcgccgcgcacgcgtcgctgatggtcgcgctcg

gcggcgggctcgaaaccggacgcgacgcgccgcacgacgcgcccgcgggcgacgcgcggcgaacgggtgcttcgggtgc

ttcgggtgcttcgggtgcttcgggtgcttcaggcgcttcaggcgcttcaggcgcttcaggcgcttcaggcgcttcaggc

accgcgggcgcttcggccaccgcatccgcgtcggccgccggccacgcgccggctggcgccgctgctccggcatcgtccg

ccggcatccgcgcggccgccagcgctcgcgcgtcaatgccggctcccgccgcggcggcgaccgcgcccgccttcgcgtc

gcccgtcgccggcgcctcgacgccgatgcccgctgcgacagcggcggcccgcgccgcccgctaa

>Bucl8_Bps_NCTC_13179.seq Created: Friday, January 03, 2014 10:39 AM

ttgcaccattctcggtttattcggcaatccgcaaaaaaatataatcgcatcgactcatctttatccgaaagggagaaaa

tcgtgcagtctccggcaatgagagggacgcttgcactcgcggttcttgcaatctcattaataatggccggatgcgcaag

catgggcgatgttgcaccgcaagacaagcaggtcgatccggcgtcgctcgacgcgggcgcggcgatcgcggcggccgag

cgcgacgccggctggcccgcggccgactggtggcgcgcgtatcgcgatccgcagctcgattcgtggatcgccgcgtcgc

tcgccggcaacccttcgctcgccgccgcgcaggcccgcgtgcgcgaggcgcagtcgctcgcgcgcatcgcgcacgccga

ggagctgccgcagttgaacggcaacctgtcgctgatgcgccagcactggccggacaacgtctattacggcccgggcccg

ctcgcgaacgccgacacgtggaacaacacgggcacgctgagcctgtcgtaccacctcgatctgtggggcaaggacaagc

acaacgccgagcgcgcgctcgacgcggcgcgcgcccgcgcggccgacgcgcgtgccgcgcagctcgagctcgaggcgaa

cgtcgtgcgcgcgtacgtcgatttcgcgaagaactacgcgctcctcgacatcgcgcacgcgacctacgagcgccagaac

gcgctcgccgaactggcgcgcaagcgcctgcgcgcgggcatcggcacgcaactcgaggtgagccaggccgaggcgccgc

tgcccgattattcgcggcagatcgactcgtatgaagaggcgatccagctcggccgtcaccagctcgccgcgctcgcggg

caaggggccgggcgcgggcgcctcgctcgcgcggccgaagctcgcgctcgacgcgaacgccgccctgccgtccgcgctg

cccgccgagctgctcggccgccggccggacatcgtcgccgcgcgctggatggtcgacgcgcaggcgcgcggcatcgacg

tcgcgaaggccgcgttctatccgaacgtcgacctgatcgcgtcgctcggcggcttcgcggtcagcgcgccgttcgcgac

gttcctgcgcgcgatgaacggcggctggtcggccggccccgcgctcacgctgccgatcttcgagggcggccggctgcgc

gcgcaactgggcgtcgcgtcggcgggctacgaccaggcggtcgagcactacaaccagacgatcgtcggcgcgctgaagg

acatcgccgacaacgtcgtgcgcctgcattcgctcgattcgcagcaaaaggacgcggcgcgcgcggtctcgctcacgcg

gcgctcgtacgatctgtcgcatgcgggcttcagccgcgggctcaccgattacgtgaacgtgctcatcgcgcagagccag

ttgctgaccgcgcaggagaaccagacccgcatcgaggccgcgcgcctcgccgcgcacgcatcgctgatggtcgcgctcg

gcggcgggctcgaaaccgggcgcgacgcgccgcacgacgcgcccgcgggcgacgcgcggcggacgggtgcttcgggtgc

ttcgggtgcttcgggtgcttcgggtgcctcgggtgcttcgagcaccgcgggcgcttcggccaccgcatccgcgtcggcc

gccggccacgcgccggctggcgccgctgctccggcatcgcccgccggcagccgcgcggccgccagcgctcgcgcgtcaa

tgccggctcccgccgcggcggcgaccgcgcccgccttcgcgtcgcccgtcgccggcgcctcgacgccgatgcccgctgc

gacagcggcggcccgcgccgcccgctaa

>Bucl8_Bps_MSHR146.seq >gi|575433883|gb|CP004042.1|:2272492-2274390 Burkholderia pseudomallei MSHR146 chromosome 1, complete sequence

TTGCACCATTCTCGGTTTATTCGGCAATCCGCAAAAAAATATAATCGCATCGACTCATCTTTATCCGAAAGGGAGAAAA

TCGTGCAGTCTCCGGCAATGAGAGGGACGCTTGCACTCGCGGTTCTTGCAATCTCATTAATAATGGCCGGATGCGCAAG

CATGGGCGATGTTGCACCGCAAGACAAGCAGGTCGATCCGGCGTCGCTCGACGCGGGCGCGGCGATCGCGGCGGCCGAG

CGCGACGCCGGCTGGCCCGCGGCCGACTGGTGGCGCGCGTATCGCGATCCGCAGCTCGATTCGTGGATCGCCGCGTCGC

TCGCCGGCAACCCTTCGCTCGCCGCCGCGCAGGCCCGCGTGCGCGAGGCGCAGTCGCTCGCGCGCATCGCGCACGCCGA

GGAGCTGCCGCAGTTGAACGGCAACCTGTCGCTGATGCGCCAGCACTGGCCGGACAACGTCTATTACGGCCCGGGCCCG

CTCGCGAACGCCGACACGTGGAACAACACGGGCACGCTGAGCCTGTCGTACCACCTCGATCTGTGGGGCAAGGACAAGC

ACAACGCCGAGCGCGCGCTCGACGCGGCGCGCGCCCGCGCGGCCGACGCGCGTGCCGCGCAGCTCGAGCTCGAGGCGAA

CGTCGTGCGCGCGTACGTCGATTTCGCGAAGAACTACGCGCTCCTCGACATCGCGCACGCGACCTACGAGCGCCAGAAC

GCGCTCGCCGAACTGGCGCGCAAGCGCCTGCGCGCGGGCATCGGCACGCAACTCGAGGTAAGCCAGGCCGAAGCGCCGC

TGCCCGATTATTCGCGGCAGATCGACTCGTATGAAGAGGCGATCCAGCTCGGCCGTCACCAGCTCGCCGCGCTCGCGGG

CAAGGGGCCGGGCGCGGGCGCCTCGCTCGCGCGGCCGAAGCTCGCGCTCGACGCGAACGCCGCCCTGCCGTCCGCGCTG

CCCGCCGAGCTGCTCGGCCGCCGGCCGGACGTCGTCGCCGCGCGCTGGATGGTCGACGCGCAGGCGCGCGGCATCGACG

TCGCGAAGGCCGCGTTCTATCCGAACGTCGACCTGATCGCGTCGCTCGGCGGCTTCGCGGTCAGCGCGCCGTTCGCGAC

GTTCCTGCGCGCGATGAACGGCGGCTGGTCGGCCGGCCCCGCGCTCACGCTGCCGATCTTCGAGGGCGGCCGGCTGCGC

GCGCAACTGGGCGTCGCGTCGGCGGACTACGACCAGGCGGTCGAGCACTACAACCAGACGATCGTCGGCGCGCTGAAGG

ACATCGCCGACAACGTCGTGCGCCTGCATTCGCTCGATTCGCAGCAAAAGGACGCGGCGCGCGCGGTCTCGCTCACGCG

GCGCTCGTACGATCTGTCGCATGCGGGCTTCAGCCGCGGGCTCACCGATTACGTGAACGTGCTCATCGCGCAGAGCCAG

TTGCTGACCGCGCAGGAGAACCAGACCCGCATCGAGGCAGCGCGCCTCGCCGCGCACGCGTCGCTGATGGTCGCGCTCG

GCGGCGGGCTCGAAACCGGGCGCGACGCGCCGCACGACGCGCCCGCGGGCGACGCGCGGCGGACGGGTGCTTCGGGTGC

TTCGGGTGCTTCGGGTGCCTCGGGTGCCTCGGGTGCTTCGGGTGCCTCGGGTGCTTCGGGTGCCTCGGGTGCTTCGAGC

ACCTCGGGTGCTTCGAGCACCTCGGGTACTTCGGCCACCGCATCCGCGTCGGCCGCCGGCCACGCGCCGGCTGGCGCCG

CTGCTCCGGCATCGCCCGCCGGCATCCGCGCGGCCGCCAGCGCTCGCGCGTCAATGCCGGCTCCCGCCGCGGCGGCGAC

CGCGCCCGCCTTCGCGTCGCCCGTCGCCGGCGCCTCGACGCCGATGCCCGCTGCGACAGCGGCGGCCCGCGCCGCCCGC

TAA

>Bucl8_Bps_MSHR511.seq >gi|574600368|gb|CP004023.1|:2127010-2128890 Burkholderia pseudomallei MSHR511 chromosome 1, complete sequence

TTGCACCATTCTCGGTTTATTCGGCAATCCGCAAAAAAATATAATCGCATCGACTCATCTTTATCCGAAAGGGAGAAAA

TCGTGCAGTCTCCGGCAATGAGAGGGACGCTTGCACTCGCGGTTCTTGCAATCTCATTAATAATGGCCGGATGCGCAAG

CATGGGCGATGTTGCACCGCAAGACAAGCAGGTCGATCCGGCGTCGCTCGACGCGGGCGCGGCGATCGCGGCGGCCGAG

CGCGACGCCGGCTGGCCCGCGGCCGACTGGTGGCGCGCGTATCGCGATCCGCAGCTCGATTCGTGGATCGCCGCGTCGC

TCGCCGGCAACCCTTCGCTCGCCGCCGCGCAGGCCCGCGTGCGCGAGGCGCAGTCGCTCGCGCGCATCGCGCACGCCGA

GGAGCTGCCGCAGTTGAACGGCAACCTGTCGCTGATGCGCCAGCACTGGCCGGACAACGTCTATTACGGCCCGGGCCCG

CTCGCGAACGCCGACACGTGGAACAACACGGGCACGCTGAGCCTGTCGTACCACCTCGATCTGTGGGGCAAGGACAAGC

ACAACGCCGAGCGCGCGCTCGACGCGGCGCGCGCCCGCGCGGCCGACGCGCGTGCCGCGCAGCTCGAGCTCGAGGCGAA

CGTCGTGCGCGCGTACGTCGATTTCGCGAAGAACTACGCGCTCCTCGACATCGCGCACGCGACCTACGAGCGCCAGAAC

GCGCTCGCCGAACTGGCGCGCAAGCGCCTGCGCGCGGGCATCGGCACGCAACTCGAGGTAAGCCAGGCCGAAGCGCCGC

TGCCCGATTATTCGCGGCAGATCGACTCGTATGAAGAGGCGATCCAGCTCGGCCGTCACCAGCTCGCCGCGCTCGCGGG

CAAGGGGCCGGGCGCGGGCGCCTCGCTCGCGCGGCCGAAGCTCGCGCTCGACGCGAACGCCGCCCTGCCGTCCGCGCTG

CCCGCCGAGCTGCTCGGCCGCCGGCCGGACGTCGTCGCCGCGCGCTGGATGGTCGACGCGCAGGCGCGCGGCATCGACG

TCGCGAAGGCCGCGTTCTATCCGAACGTCGACCTGATCGCGTCGCTCGGCGGCTTCGCGGTCAGCGCGCCGTTCGCGAC

GTTCCTGCGCGCGATGAACGGCGGCTGGTCGGCCGGCCCCGCGCTCACGCTGCCGATCTTCGAGGGCGGCCGGCTGCGC

GCGCAACTGGGCGTCGCGTCGGCGGACTACGACCAGGCGGTCGAGCACTACAACCAGACGATCGTCGGCGCGCTGAAGG

ACATCGCCGACAACGTCGTGCGCCTGCATTCGCTCGATTCGCAGCAAAAGGACGCGGCGCGCGCGGTCTCGCTCACGCG

GCGCTCGTACGATCTGTCGCATGCGGGCTTCAGCCGCGGGCTCACCGATTACGTGAACGTGCTCATCGCGCAGAGCCAG

TTGCTGACCGCGCAGGAGAACCAGACCCGCATCGAGGCAGCGCGCCTCGCCGCGCACGCGTCGCTGATGGTCGCGCTCG

GCGGCGGGCTCGAAACCGGGCGCGACGCGCCGCACGACGCGCCCGCGGGCGACGCGCGGCGGACGGGTGCTTCGGGTGC

TTCGGGTGCTTCGGGTGCCTCGGGTGCCTCGGGTGCTTCGGGTGCCTCGGGTGCTTCGGGTGCCTCGGGTGCTTCGAGC

ACCTCGGGTACTTCGGCCACCGCATCCGCGTCGGCCGCCGGCCACGCGCCGGCTGGCGCCGCTGCTCCGGCATCGCCCG

CCGGCATCCGCGCGGCCGCCAGCGCTCGCGCGTCAATGCCGGCTCCCGCCGCGGCGGCGACCGCGCCCGCCTTCGCGTC

GCCCGTCGCCGGCGCCTCGACGCCGATGCCCGCTGCGACAGCGGCGGCCCGCGCCGCCCGCTAA

>Bucl8_Bps_MSHR520.seq >gi|589299693|gb|CP004368.1|:2746149-2748026 Burkholderia pseudomallei MSHR520 chromosome 1, complete sequence

TTGCACCATTCTCGGTTTATTCGGCAATCCGCAAAAAAATATAATCGCATCGACTCATCTTTATCCGAAAGGGAGAAAA

TCGTGCAGTCTCCGGCAATGAGAGGGACGCTTGCACTCGCGGTTCTTGCAATCTCATTAATAATGGCCGGATGCGCAAG

CATGGGCGATGTTGCACCGCAAGACAAGCAGGTCGATCCGGCGTCGCTCGACGCGGGCGCGGCGATCGCGGCGGCCGAG

CGCGACGCCGGCTGGCCCGCGGCCGACTGGTGGCGCGCGTATCGCGATCCGCAGCTCGATTCGTGGATCGCCGCGTCGC

TCGCCGGCAACCCTTCGCTCGCCGCCGCGCAGGCCCGCGTGCGCGAGGCGCAGTCGCTCGCGCGCATCGCGCACGCCGA

GGAACTGCCGCAGTTGAACGGCAACCTGTCGCTGATGCGCCAGCACTGGCCGGACAACGTCTATTACGGCCCGGGCCCG

CTCGCGAACGCCGACACGTGGAACAACACGGGCACGCTGAGCCTGTCGTACCACCTCGATCTGTGGGGCAAGGACAAGC

ACAACGCCGAGCGCGCGCTCGACGCGGCGCGCGCCCGCGCGGCCGACGCGCGTGCCGCGCAGCTCGAGCTCGAGGCGAA

CGTCGTGCGCGCGTACGTCGATTTCGCGAAGAACTACGCGCTCCTCGACATCGCGCACGCGACCTACGAGCGCCAGAAC

GCGCTCGCCGAACTGGCGCGCAAGCGCCTGCGCGCGGGCATCGGCACGCAACTCGAGGTGAGCCAGGCCGAGGCGCCGC

TGCCCGATTATTCGCGGCAGATCGACTCGTATGAAGAGGCGATCCAGCTCGGCCGTCACCAGCTCGCCGCGCTCGCGGG

CAAGGGGCCGGGCGCGGGCGCCTCGCTCGCGCGGCCGAAGCTCGCGCTCGACGCGAACGCCGCCCTGCCGTCCGCGCTG

CCCGCCGAGCTGCTCGGCCGCCGGCCGGACATCGTCGCCGCGCGCTGGATGGTCGACGCGCAGGCGCGCGGCATCGACG

TCGCGAAGGCCGCGTTCTATCCGAACGTCGACCTGATCGCGTCGCTCGGCGGCTTCGCGGTCAGCGCGCCGTTCGCGAC

GTTCCTGCGCGCGATGAACGGCGGCTGGTCGGCCGGCCCCGCGCTCACGCTGCCGATCTTCGAGGGCGGCCGGCTGCGC

GCGCAACTGGGCGTCGCGTCGGCGGGCTACGACCAGGCGGTCGAGCACTACAACCAGACGATCGTCGGCGCGCTGAAGG

ACATCGCCGACAACGTCGTGCGCCTGCATTCGCTCGATTCGCAGCAAAAGGACGCGGCGCGCGCGGTCTCGCTCACGCG

GCGCTCGTACGATCTGTCGCATGCGGGCTTCAGCCGCGGGCTCACCGATTACGTGAACGTGCTCATCGCGCAGAGCCAG

TTGCTGACCGCGCAGGAGAACCAGACCCGCATCGAGGCCGCGCGCCTCGCCGCGCACGCGTCGCTGATGGTCGCGCTCG

GCGGCGGGCTCGAAACCGGGCACGACGCGCCCGCGGGCGACGCGCGGCGGACGGGTGCTTCGGGTGCTTCGGGTGCTTC

GGGTGCTTCGGGTGCTTCGGGTGCTTCGGGCGCTTCGGGCGCTTCGGGCGCTTCGGGTGCTTCAGGCGCTTCAGGCACC

GCGGGCGCTTCGGCCGCCGCATCCGCGTCGGCCGCCGGCCACGCGCCGGCTGGCGCCGCTGCTCCGGCATCGCCCGCCG

GCATCCGCGCGGCCGCCAGCGCTCGCGCGTCAATGCCGGCTCCCGCCGCGGCGGCGACCGCGCCCGCCTTCGCGTCGCC

CGTCGCCGGCGCCTCGACGCCGATGCCCGCTGCGACAGCGGCGGCCCGCGCCGCCCGCTAA

>Bucl8_Bps_NAU20B-16.seq >gi|568789317|gb|CP004003.1|:3937544-3939424 Burkholderia pseudomallei NAU20B-16 chromosome 1, complete sequence

TTGCACCATTCTCGGTTTATTCGGCAATCCGCAAAAAAATATAATCGCATCGACTCATCTTTATCCGAAAGGGAGAAAA

TCGTGCAGTCTCCGGCAATGAGAGGGACGCTTGCACTCGCGGTTCTTGCAATCTCATTAATAATGGCCGGATGCGCAAG

CATGGGCGATGTTGCACCGCAAGACAAGCAGGTCGATCCGGCGTCGCTCGACGCGGGCGCGGCGATCGCGGCGGCCGAG

CGCGACGCCGGCTGGCCCGCGGCCGACTGGTGGCGCGCGTATCGCGATCCGCAGCTCGATTCGTGGATCGCCGCGTCGC

TCGCCGGCAACCCTTCGCTCGCCGCCGCGCAGGCCCGCGTGCGCGAGGCGCAGTCGCTCGCGCGCATCGCGCACGCCGA

GGAGCTGCCGCAGTTGAACGGCAACCTGTCGCTGATGCGCCAGCACTGGCCGGACAACGTCTATTACGGCCCGGGCCCG

CTCGCGAACGCCGACACGTGGAACAACACGGGCACGCTGAGCCTGTCGTACCACCTCGATCTGTGGGGCAAGGACAAGC

ACAACGCCGAGCGCGCGCTCGACGCGGCGCGCGCCCGCGCGGCCGACGCGCGTGCCGCGCAGCTCGAGCTCGAGGCGAA

CGTCGTGCGCGCGTACGTCGATTTCGCGAAGAACTACGCGCTCCTCGACATCGCGCACGCGACCTACGAGCGCCAGAAC

GCGCTCGCCGAACTGGCGCGCAAGCGCCTGCGCGCGGGCATCGGCACGCAACTCGAGGTAAGCCAGGCCGAAGCGCCGC

TGCCCGATTATTCGCGGCAGATCGACTCGTATGAAGAGGCGATCCAGCTCGGCCGTCACCAGCTCGCCGCGCTCGCGGG

CAAGGGGCCGGGCGCGGGCGCCTCGCTCGCGCGGCCGAAGCTCGCGCTCGACGCGAACGCCGCCCTGCCGTCCGCGCTG

CCCGCCGAGCTGCTCGGCCGCCGGCCGGACGTCGTCGCCGCGCGCTGGATGGTCGACGCGCAGGCGCGCGGCATCGACG

TCGCGAAGGCCGCGTTCTATCCGAACGTCGACCTGATCGCGTCGCTCGGCGGCTTCGCGGTCAGCGCGCCGTTCGCGAC

GTTCCTGCGCGCGATGAACGGCGGCTGGTCGGCCGGCCCCGCGCTCACGCTGCCGATCTTCGAGGGCGGCCGGCTGCGC

GCGCAACTGGGCGTCGCGTCGGCGGACTACGACCAGGCGGTCGAGCACTACAACCAGACGATCGTCGGCGCGCTGAAGG

ACATCGCCGACAACGTCGTGCGCCTGCATTCGCTCGATTCGCAGCAAAAGGACGCGGCGCGCGCGGTCTCGCTCACGCG

GCGCTCGTACGATCTGTCGCATGCGGGCTTCAGCCGCGGGCTCACCGATTACGTGAACGTGCTCATCGCGCAGAGCCAG

TTGCTGACCGCGCAGGAGAACCAGACCCGCATCGAGGCAGCGCGCCTCGCCGCGCACGCGTCGCTGATGGTCGCGCTCG

GCGGCGGGCTCGAAACCGGGCGCGACGCGCCGCACGACGCGCCCGCGGGCGACGCGCGGCGGACGGGTGCTTCGGGTGC

TTCGGGTGCTTCGGGTGCCTCGGGTGCCTCGGGTGCTTCGGGTGCCTCGGGTGCTTCGGGTGCCTCGGGTGCTTCGAGC

ACCTCGGGTACTTCGGCCACCGCATCCGCGTCGGCCGCCGGCCACGCGCCGGCTGGCGCCGCTGCTCCGGCATCGCCCG

CCGGCATCCGCGCGGCCGCCAGCGCTCGCGCGTCAATGCCGGCTCCCGCCGCGGCGGCGACCGCGCCCGCCTTCGCGTC

GCCCGTCGCCGGCGCCTCGACGCCGATGCCCGCTGCGACAGCGGCGGCCCGCGCCGCCCGCTAA

>Bucl8_Bps_NCTC_13178.seq >gi|568795288|gb|CP004001.1|:1608848-1610743 Burkholderia pseudomallei NCTC 13178 chromosome 1, complete sequence

TTGCACCATTCTCGGTTTATTCGGCAATCCGCAAAAAAATATAATCGCATCGACTCATCTTTATCCGAAAGGGAGAAAA

TCGTGCAGTCTCCGGCAATGAGAGGGACGCTTGCACTCGCGGTTCTTGCAATCTCATTAATAATGGCCGGATGCGCAAG

CATGGGCGATGTTGCACCGCAAGACAAGCAGGTCGATCCGGCGTCGCTCGACGCGGGCGCGGCGATCGCGGCGGCCGAG

CGCGACGCCGGCTGGCCCGCGGCCGACTGGTGGCGCGCGTATCGCGATCCGCAGCTCGATTCGTGGATCGCCGCGTCGC

TCGCCGGCAACCCTTCGCTCGCCGCCGCGCAGGCCCGCGTGCGCGAGGCGCAGTCGCTCGCGCGCATCGCGCACGCCGA

GGAGCTGCCGCAGTTGAACGGCAACCTGTCGCTGATGCGCCAGCACTGGCCGGACAACGTCTATTACGGCCCGGGCCCG

CTCGCGAACGCCGACACGTGGAACAACACGGGCACGCTGAGCCTGTCGTACCACCTCGATCTGTGGGGCAAGGACAAGC

ACAACGCCGAGCGCGCGCTCGACGCGGCGCGCGCCCGCGCGGCCGACGCGCGTGCCGCGCAGCTCGAGCTCGAGGCGAA

CGTCGTGCGCGCGTACGTCGATTTCGCGAAGAACTACGCGCTCCTCGACATCGCGCACGCGACCTACGAGCGCCAGAAC

GCGCTCGCCGAACTGGCGCGCAAGCGCCTGCGCGCGGGCATCGGCACGCAACTCGAGGTGAGCCAGGCCGAAGCGCCGC

TGCCCGATTATTCGCGGCAGATCGACTCGTATGAAGAGGCGATCCAGCTCGGCCGTCACCAGCTCGCCGCGCTCGCGGG

CAAGGGGCCGGGCGCGGGCGCCTCGCTCGCGCGGCCGAAGCTCGCGCTCGACGCGAACGCCGCCCTGCCGTCCGCGCTG

CCCGCCGAGCTGCTCGGCCGCCGGCCGGACATCGTCGCCGCGCGCTGGATGGTCGACGCGCAGGCGCGCGGCATCGACG

TCGCGAAGGCCGCGTTCTATCCGAACGTCGACCTGATCGCGTCGCTCGGCGGCTTCGCGGTCAGCGCGCCGTTCGCGAC

GTTCCTGCGCGCGATGAACGGCGGCTGGTCGGCCGGCCCCGCGCTCACGCTGCCGATCTTCGAGGGCGGCCGGCTGCGC

GCGCAACTGGGCGTCGCGTCGGCGGGCTACGACCAGGCGGTCGAGCACTACAACCAGACGATCGTCGGCGCGCTGAAGG

ACATCGCCGACAACGTCGTGCGCCTGCATTCGCTCGATTCGCAGCAAAAGGACGCGGCGCGCGCGGTCTCGCTCACGCG

GCGCTCGTACGATCTGTCGCATGCGGGCTTCAGCCGCGGGCTCACCGACTACGTGAACGTGCTCATCGCGCAGAGCCAG

TTGCTGACCGCGCAGGAGAACCAGACCCGCATCGAGGCCGCGCGCCTCGCCGCGCACGCGTCGCTGATGGTCGCGCTCG

GCGGCGGGCTCGAAACCGGGCACGACGCGCCCGCGGGCGACGCGCGGCGGACGGGTGCTTCGGGTGCTTCGGGTGCTTC

GGGTGCTTCGGGTGCTTCGGGTGCTTCGGGTGCTTCGGGTGCTTCGGGTGCTTCGGGCGCTTCGGGCGCTTCGGGTGCT

TCAGGCGCTTCAGGCACCGCGGGCGCTTCGGCCACCGCATCCGCGTCGGCCGCCGGCCACGCGCCGGCTGGCGCCGCTG

CTCCGGCATCGCCCGCCGGCATCCGCGCGGCCGCCAGCGCTCGCGCGTCAATGCCGGCTCCCGCCGCGGCGGCGACCGC

GCCCGCCTTCGCGTCGCCCGTCGCCGGCGCCTCGACGCCGATGCCCGCTGCGACAGCGGCGGCCCGCGCCGCCCGCTAA

>B7CU27_BURPS_Bucl8_Bps_576.seq Created: Friday, August 02, 2013 11:21 AM

TTGCACCATTCTCGGTTTATTCGGCAATCCGCAAAAAAATATAATCGCATCGACTCATCTTTATCCGAAAGGGAGAAAA

TCGTGCAGTCTCCGGCAATGAGAGGGACGCTTGCACTCGCGGTTCTTGCAATCTCATTAATAATGGCCGGATGCGCAAG

CATGGGCGATGTTGCACCGCAAGACAAGCAGGTCGATCCGGCGTCGCTCGATGCGGGCGCGGCGATCGCGGCGGCCGAG

CGCGACGCCGGCTGGCCCGCGGCCGACTGGTGGCGCGCGTATCGCGATCCGCAGCTCGATTCGTGGATCGCCGCGTCGC

TCGCCGGCAACCCTTCGCTCGCCGCCGCGCAGGCCCGCGTGCGCGAGGCGCAGTCGCTCGCGCGCATCGCGCACGCCGA

GGAGCTGCCGCAGTTGAACGGCAACCTGTCGCTGATGCGCCAGCACTGGCCGGACAACGTCTATTACGGCCCGGGCCCG

CTCGCGAACGCCGACACGTGGAACAACACGGGCACGCTGAGCCTGTCGTACCACCTCGATCTGTGGGGCAAGGACAAGC

ACAACGCCGAGCGCGCGCTCGACGCGGCGCGCGCCCGCGCGGCCGACGCGCGTGCCGCGCAGCTCGAGCTCGAGGCGAA

CGTCGTGCGCGCGTACGTCGATTTCGCGAAGAACTACGCGCTCCTCGACATCGCGCACGCGACCTACGAGCGCCAGAAC

GCGCTCGCCGAACTGGCGCGCAAGCGCCTGCGCGCGGGCATCGGCACGCAACTCGAGGTGAGCCAGGCCGAGGCGCCGC

TGCCCGATTATTCGCGGCAGATCGACTCGTATGAAGAGGCGATCCAGCTCGGCCGTCACCAGCTCGCCGCGCTCGCGGG

CAAGGGGCCGGGCGCGGGCGCCTCGCTCGCGCGGCCGAAGCTCGCGCTCGACGCGAACGCCGCCCTGCCGTCCGCGCTG

CCCGCCGAGCTGCTCGGCCGCCGGCCGGACATCGTCGCCGCGCGCTGGATGGTCGACGCGCAGGCGCGCGGCATCGACG

TCGCGAAGGCCGCGTTCTATCCGAACGTCGACCTGATCGCGTCGCTCGGCGGCTTCGCGGTCAGCGCGCCGTTCGCGAC

GTTCCTGCGCGCGATGAACGGCGGCTGGTCGGCCGGCCCCGCGCTCACGCTGCCGATCTTCGAGGGCGGCCGGCTGCGC

GCGCAACTGGGCGTCGCGTCGGCGGGCTACGACCAGGCGGTCGAGCACTACAACCAGACGATCGTCGGCGCGCTGAAGG

ACATCGCCGACAACGTCGTGCGCCTGCATTCGCTCGATTCGCAGCAAAAGGACGCGGCGCGCGCGGTCTCGCTCACGCG

GCGCTCGTACGATCTGTCGCATGCGGGCTTCAGCCGCGGGCTCACCGACTACGTGAACGTGCTCATCGCGCAGAGCCAG

TTGCTGACCGCGCAGGAGAACCAGACCCGCATCGAGGCCGCGCGCCTCGCCGCGCACGCGTCGCTGATGGTCGCGCTCG

GCGGCGGGCTCGAAACCGGGCGCGACGCGCCGCACGACGCGCCCGCGGGCGACGCGCGGCGGACGGGTGCTTCGGGTGC

TTCGGGTGCTTCGCGTGCTTCGCGTGCTTCGCGTGCTTCGCGTGCTTCGGGTGCTTCGGGTGCTTCGGGTGCCTCGGGT

GCCTCGGGTGCCTCGGGTGCCTCGGGTGCCTCGGGTGCCTCGGGTGCCTCGGGTGCCTCGGGTGCCTCGGGTGCCTCGG

GTGCCTCGGGTGCTTCGAGCACCGCGGGCGCTTCGGCCACCGCATCCGCGTCGGCCGCCGGCCATGCGCCGGCTGGCGC

CGCTGCTCCGGCATCGCCCGCCGGCATCCGCGCGGCCGCCAGCGCTCGCGCGTCAATGCCGGCTCCCGCCGCGGCGGCG

ACCGCGCCCGCCTTCGCGTCGCCCGTCGCCGGCGCCTCGACGCCGATGCCCGCTGCGACAGCGGCAGCCCGCGCCGCCC

GCTAA

>C0XVP3_BURPS_Bucl8_Bps_Pakistan_9.seq Created: Monday, January 20, 2014 10:50 AM

TTGCACCATTCTCGGTTTATTCGGCAATCCGCAAAAAAATATAATCGCATCGACTCATCTTTATCCGAAAGGGAGAAAA

TCGTGCAGTCTCCGGCAATGAGAGGGACGCTTGCACTCGCGGTTCTTGCAATCTCATTAATAATGGCCGGATGCGCAAG

CATGGGCGATGTTGCACCGCAAGACAAGCAGGTCGATCCGGCGTCGCTCGATGCGGGCGCGGCGATCGCGGCGGCCGAG

CGCGACGCCGGCTGGCCCGCGGCCGACTGGTGGCGCGCGTATCGCGATCCGCAGCTCGATTCGTGGATCGCCGCGTCGC

TCGCCGGCAACCCTTCGCTCGCCGCCGCGCAGGCCCGCGTGCGCGAGGCGCAGTCGCTCGCGCGCATCGCGCACGCCGA

GGAGCTGCCGCAGTTGAACGGCAACCTGTCGCTGATGCGCCAGCACTGGCCGGACAACGTCTATTACGGCCCGGGCCCG

CTCGCGAACGCCGACACGTGGAACAACACGGGCACGCTGAGCCTGTCGTACCACCTCGATCTGTGGGGCAAGGACAAGC

ACAACGCCGAGCGCGCGCTCGACGCGGCGCGCGCCCGCGCGGCCGACGCGCGTGCCGCGCAGCTCGAGCTCGAGGCGAA

CGTCGTGCGCGCGTACGTCGATTTCGCGAAGAACTACGCGCTCCTCGACATCGCGCACGCGACCTACGAGCGCCAGAAC

GCGCTCGCCGAACTGGCGCGCAAGCGCCTGCGCGCGGGCATCGGCACGCAACTCGAGGTGAGCCAGGCCGAGGCGCCGC

TGCCCGATTATTCGCGGCAGATCGACTCGTATGAAGAGGCGATCCAGCTCGGCCGTCACCAGCTCGCCGCGCTCGCGGG

CAAGGGGCCGGGCGCGGGCGCCTCGCTCGCGCGGCCGAAGCTCGCGCTCGACGCGAACGCCGCCCTGCCGTCCGCGCTG

CCCGCCGAGCTGCTCGGCCGCCGGCCGGACATCGTCGCCGCGCGCTGGATGGTCGACGCGCAGGCGCGCGGCATCGACG

TCGCGAAGGCCGCGTTCTATCCGAACGTCGACCTGATCGCGTCGCTCGGCGGCTTCGCGGTCAGCGCGCCGTTCGCGAC

GTTCCTGCGCGCGATGAACGGCGGCTGGTCGGCCGGCCCCGCGCTCACGCTGCCGATCTTCGAGGGCGGCCGGCTGCGC

GCGCAACTGGGCGTCGCGTCGGCGGGCTACGACCAGGCGGTCGAGCACTACAACCAGACGATCGTCGGCGCGCTGAAGG

ACATCGCCGACAACGTCGTGCGCCTGCATTCGCTCGATTCGCAGCAAAAGGACGCGGCGCGCGCGGTCTCGCTCACGCG

GCGCTCGTACGATCTGTCGCATGCGGGCTTCAGCCGCGGGCTCACCGACTACGTGAACGTGCTCATCGCGCAGAGCCAG

TTGCTGACCGCGCAGGAGAACCAGACCCGCATCGAGGCCGCGCGCCTCGCCGCGCACGCGTCGCTGATGGTCGCGCTCG

GCGGCGGGCTCGAAACCGGGCGCGACGCGCCGCACGACGCGCCCGCGGGCGACGCGCGGCGGACGGGTGCTTCGGGTGC

TTCGGGTGCTTCGGGTGCTTCGGGTGCTTCGGGTGCTTCGGGTGCCTCGGGTGCCTCGGGTGCCTCGGGTGCCTCGGGT

GCCTCGGGTGCCTCGGGTGCCTCGGGTGCTTCGAGCACCGCGGGCGCTTCGGCCACCGCATCCGCGTCGGCCGCCGGCC

ATGCGCCGGCTGGCGCCGCTGCTCCGGCATCGCCCGCCGGCATCCGCGCGGCCGCCAGCGCTCGCGCGTCAATGCCGGC

TCCCGCCGCGGCGGCGACCGCGCCCGCCTTCGCGTCGCCCGTCGCCGGCGCCTCGACGCCGATGCCCGCTGCGACAGCG

GCGGCCCGCGCCGCCCGCTAA

>Bucl8_Bm_2000031281.seq Created: Wednesday, March 26, 2014 10:12 AM

TTGCACCATTCTCGGTTTATTCGGCAATCCGCAAAAAAATATAATCGCATCGACTCATCTTTATCCGAAAGGGAGAAAA

TCGTGCAGTCTCCGGCAATGAGAGGGACGCTTGCACTCGCGGTTCTTGCAATCTCATTAATAATGGCCGGATGCGCAAG

CATGGGCGATGTTGCACCGCAAGACAAGCAGGTCGATCCGGCGTCGCTCGACGCGGGCGCGGCGATCGCGGCGGCCGAG

CGCGACGCCGGCTGGCCCGCGGCCGACTGGTGGCGCGCGTATCGCGATCCGCAGCTCGATTCGTGGATCGTCGCGTCGC

TCGCCGGCAACCCTTCGCTCGCCGCCGCGCAGGCCCGCGTGCGCGAGGCGCAGTCGCTCGCGCGCATCGCGCACGCCGA

GGAGCTGCCGCAGTTGAACGGCAACCTGTCGCTGATGCGCCAGCACTGGCCGGACAACGTCTATTACGGCCCGGGCCCG

CTCGCGAACGCCGACACGTGGAACAACACGGGCACGCTGAGCCTGTCGTACCACCTCGATCTGTGGGGCAAGGACAAGC

ACAACGCCGAGCGCGCGCTCGACGCGGCGCGCGCCCGCGCGGCCGACGCGCGTGCCGCGCAGCTCGAGCTCGAGGCGAA

CGTCGTGCGCGCGTACGTCGATTTCGCGAAGAACTACGCGCTCCTCGACATCGCGCACGCGACCTACGAGCGCCAGAAC

GCGCTCGCCGAACTGGCGCGCAAGCGCCTGCGCGCGGGCATCGGCACGCAACTCGAGGTGAGCCAGGCCGAGGCGCCGC

TGCCCGATTATTCGCGGCAGATCGACTCGTATGAAGAGGCGATCCAGCTCGGCCGTCACCAGCTCGCCGCGCTCGCGGG

CAAGGGGCCGGGCGCGGGCGCCTCGCTCGCGCGGCCGAAGCTCGCGCTCGACGCGAACGCCGCCCTGCCGTCCGCGCTG

CCCGCCGAGCTGCTCGGCCGCCGGCCGGACATCGTCGCCGCGCGCTGGATGGTCGACGCGCAGGCGCGCGGCATCGACG

TCGCGAAGGCCGCGTTCTATCCGAACGTCGACCTGATCGCGTCGCTCGGCGGCTTCGCGGTCAGCGCGCCGTTCGCGAC

GTTCCTGCGCGCGATGAACGGCGGCTGGTCGGCCGGCCCCGCGCTCACGCTGCCGATCTTCGAGGGCGGCCGGCTGCGC

GCGCAACTGGGCGTCGCGTCGGCGGGCTACGACCAGGCGGTCGAGCACTACAACCAGACGATCGTCGGCGCGCTGAAGG

ACATCGCCGACAACGTCGTGCGCCTGCATTCGCTCGATTCGCAGCAAAAGGACGCGGCGCGCGCGGTCTCGCTCACGCG

GCGCTCGTACGATCTGTCGCATGCGGGCTTCAGCCGCGGGCTCACCGACTACGTGAACGTGCTCATCGCGCAGAGCCAG

TTGCTGACCGCGCAGGAGAACCAGACCCGCATCGAGGCCGCGCGCCTCGCCGCGCACGCGTCGCTGATGGTCGCGCTCG

GCGGCGGGCTCGAAACCGGGCGCGACGCGCCGCACGACGCGCCCGCGGGCGACGCGCGGCGGACGGGTGCTTCGGGTGC

TTCGGGTGCTTCGGGTGCTTCGGGTGCCTCGGGTGCCTCGGGTGCCTCGGGTGCTTCGAGCACCGCGGGCGCTTCGGCC

ACCGCATCCGCGTCGGCCGCCGGCCACGCGCCGGCTGGCGCCGCTGCTCCGGCATCGCCCGCCGGCATCCGCGCGGCCG

CCAGCGCTCGCGCGTCAATGCCGGCTCCCGCCGCGGCGGCGACCGCGCCCGCCTTCGCGTCGCCCGTCGCCGGCGCCTC

GACGCCGATGCCCGCTGCGACAGCGGCGGCCCGCGCCGCCCGCTAA

>Bucl8_Bm_2002721280.seq Created: Wednesday, March 26, 2014 11:16 AM

TTGCACCATTCTCGGTTTATTCGGCAATCCGCAAAAAAATATAATCGCATCGACTCATCTTTATCCGAAAGGGAGAAAA

TCGTGCAGTCTCCGGCAATGAGAGGGACGCTTGCACTCGCGGTTCTTGCAATCTCATTAATAATGGCCGGATGCGCAAG

CATGGGCGATGTTGCACCGCAAGACAAGCAGGTCGATCCGGCGTCGCTCGACGCGGGCGCGGCGATCGCGGCGGCCGAG

CGCGACGCCGGCTGGCCCGCGGCCGACTGGTGGCGCGCGTATCGCGATCCGCAGCTCGATTCGTGGATCGTCGCGTCGC

TCGCCGGCAACCCTTCGCTCGCCGCCGCGCAGGCCCGCGTGCGCGAGGCGCAGTCGCTCGCGCGCATCGCGCACGCCGA

GGAGCTGCCGCAGTTGAACGGCAACCTGTCGCTGATGCGCCAGCACTGGCCGGACAACGTCTATTACGGCCCGGGCCCG

CTCGCGAACGCCGACACGTGGAACAACACGGGCACGCTGAGCCTGTCGTACCACCTCGATCTGTGGGGCAAGGACAAGC

ACAACGCCGAGCGCGCGCTCGACGCGGCGCGCGCCCGCGCGGCCGACGCGCGTGCCGCGCAGCTCGAGCTCGAGGCGAA

CGTCGTGCGCGCGTACGTCGATTTCGCGAAGAACTACGCGCTCCTCGACATCGCGCACGCGACCTACGAGCGCCAGAAC

GCGCTCGCCGAACTGGCGCGCAAGCGCCTGCGCGCGGGCATCGGCACGCAACTCGAGGTGAGCCAGGCCGAGGCGCCGC

TGCCCGATTATTCGCGGCAGATCGACTCGTATGAAGAGGCGATCCAGCTCGGCCGTCACCAGCTCGCCGCGCTCGCGGG

CAAGGGGCCGGGCGCGGGCGCCTCGCTCGCGCGGCCGAAGCTCGCGCTCGACGCGAACGCCGCCCTGCCGTCCGCGCTG

CCCGCCGAGCTGCTCGGCCGCCGGCCGGACATCGTCGCCGCGCGCTGGATGGTCGACGCGCAGGCGCGCGGCATCGACG

TCGCGAAGGCCGCGTTCTATCCGAACGTCGACCTGATCGCGTCGCTCGGCGGCTTCGCGGTCAGCGCGCCGTTCGCGAC

GTTCCTGCGCGCGATGAACGGCGGCTGGTCGGCCGGCCCCGCGCTCACGCTGCCGATCTTCGAGGGCGGCCGGCTGCGC

GCGCAACTGGGCGTCGCGTCGGCGGGCTACGACCAGGCGGTCGAGCACTACAACCAGACGATCGTCGGCGCGCTGAAGG

ACATCGCCGACAACGTCGTGCGCCTGCATTCGCTCGATTCGCAGCAAAAGGACGCGGCGCGCGCGGTCTCGCTCACGCG

GCGCTCGTACGATCTGTCGCATGCGGGCTTCAGCCGCGGGCTCACCGACTACGTGAACGTGCTCATCGCGCAGAGCCAG

TTGCTGACCGCGCAGGAGAACCAGACCCGCATCGAGGCCGCGCGCCTCGCCGCGCACGCGTCGCTGATGGTCGCGCTCG

GCGGCGGGCTCGAAACCGGGCGCGACGCGCCGCACGACGCGCCCGCGGGCGACGCGCGGCGGACGGGTGCTTCGGGTGC

TTCGGGTGCTTCGGGTGCCTCGGGTGCCTCGGGTGCTTCGAGCACCGCGGGCGCTTCGGCCACCGCATCCGCGTCGGCC

GCCGGCCACGCGCCGGCTGGCGCCGCTGCTCCGGCATCGCCCGCCGGCATCCGCGCGGCCGCCAGCGCTCGCGCGTCAA

TGCCGGCTCCCGCCGCGGCGGCGACCGCGCCCGCCTTCGCGTCGCCCGTCGCCGGCGCCTCGACGCCGATGCCCGCTGC

GACAGCGGCGGCCCGCGCCGCCCGCTAA

>Bucl8_Bm_A188.seq Created: Wednesday, March 26, 2014 10:08 AM

TTGCACCATTCTCGGTTTATTCGGCAATCCGCAAAAAAATATAATCGCATCGACTCATCTTTATCCGAAAGGGAGAAAA

TCGTGCAGTCTCCGGCAATGAGAGGGACGCTTGCACTCGCGGTTCTTGCAATCTCATTAATAATGGCCGGATGCGCAAG

CATGGGCGATGTTGCACCGCAAGACAAGCAGGTCGATCCGGCGTCGCTCGACGCGGGCGCGGCGATCGCGGCGGCCGAG

CGCGACGCCGGCTGGCCCGCGGCCGACTGGTGGCGCGCGTATCGCGATCCGCAGCTCGATTCGTGGATCGTCGCGTCGC

TCGCCGGCAACCCTTCGCTCGCCGCCGCGCAGGCCCGCGTGCGCGAGGCGCAGTCGCTCGCGCGCATCGCGCACGCCGA

GGAGCTGCCGCAGTTGAACGGCAACCTGTCGCTGATGCGCCAGCACTGGCCGGACAACGTCTATTACGGCCCGGGCCCG

CTCGCGAACGCCGACACGTGGAACAACACGGGCACGCTGAGCCTGTCGTACCACCTCGATCTGTGGGGCAAGGACAAGC

ACAACGCCGAGCGCGCGCTCGACGCGGCGCGCGCCCGCGCGGCCGACGCGCGTGCCGCGCAGCTCGAGCTCGAGGCGAA

CGTCGTGCGCGCGTACGTCGATTTCGCGAAGAACTACGCGCTCCTCGACATCGCGCACGCGACCTACGAGCGCCAGAAC

GCGCTCGCCGAACTGGCGCGCAAGCGCCTGCGCGCGGGCATCGGCACGCAACTCGAGGTGAGCCAGGCCGAGGCGCCGC

TGCCCGATTATTCGCGGCAGATCGACTCGTATGAAGAGGCGATCCAGCTCGGCCGTCACCAGCTCGCCGCGCTCGCGGG

CAAGGGGCCGGGCGCGGGCGCCTCGCTCGCGCGGCCGAAGCTCGCGCTCGACGCGAACGCCGCCCTGCCGTCCGCGCTG

CCCGCCGAGCTGCTCGGCCGCCGGCCGGACATCGTCGCCGCGCGCTGGATGGTCGACGCGCAGGCGCGCGGCATCGACG

TCGCGAAGGCCGCGTTCTATCCGAACGTCGACCTGATCGCGTCGCTCGGCGGCTTCGCGGTCAGCGCGCCGTTCGCGAC

GTTCCTGCGCGCGATGAACGGCGGCTGGTCGGCCGGCCCCGCGCTCACGCTGCCGATCTTCGAGGGCGGCCGGCTGCGC

GCGCAACTGGGCGTCGCGTCGGCGGGCTACGACCAGGCGGTCGAGCACTACAACCAGACGATCGTCGGCGCGCTGAAGG

ACATCGCCGACAACGTCGTGCGCCTGCATTCGCTCGATTCGCAGCAAAAGGACGCGGCGCGCGCGGTCTCGCTCACGCG

GCGCTCGTACGATCTGTCGCATGCGGGCTTCAGCCGCGGGCTCACCGACTACGTGAACGTGCTCATCGCGCAGAGCCAG

TTGCTGACCGCGCAGGAGAACCAGACCCGCATCGAGGCCGCGCGCCTCGCCGCGCACGCGTCGCTGATGGTCGCGCTCG

GCGGCGGGCTCGAAACCGGGCGCGACGCGCCGCACGACGCGCCCGCGGGCGACGCGCGGCGGACGGGTGCTTCGGGTGC

TTCGGGTGCTTCGGGTGCTTCGGGTGCTTCGGGTGCCTCGGGTGCCTCGGGTGCCTCGGGTGCTTCGAGCACCGCGGGC

GCTTCGGCCACCGCATCCGCGTCGGCCGCCGGCCACGCGCCGGCTGGCGCCGCTGCTCCGGCATCGCCCGCCGGCATCC

GCGCGGCCGCCAGCGCTCGCGCGTCAATGCCGGCTCCCGCCGCGGCGGCGACCGCGCCCGCCTTCGCGTCGCCCGTCGC

CGGCGCCTCGACGCCGATGCCCGCTGCGACAGCGGCGGCCCGCGCCGCCCGCTAA

>Bucl8_Bm_A193.seq Reverse Complement DNA Sequence Untitled Seq #43(1,1845)

TTGCACCATTCTCGGTTTATTCGGCAATCCGCAAAAAAATATAATCGCATCGACTCATCTTTATCCGAAAGGGAGAAAA

TCGTGCAGTCTCCGGCAATGAGAGGGACGCTTGCACTCGCGGTTCTTGCAATCTCATTAATAATGGCCGGATGCGCAAG

CATGGGCGATGTTGCACCGCAAGACAAGCAGGTCGATCCGGCGTCGCTCGACGCGGGCGCGGCGATCGCGGCGGCCGAG

CGCGACGCCGGCTGGCCCGCGGCCGACTGGTGGCGCGCGTATCGCGATCCGCAGCTCGATTCGTGGATCGTCGCGTCGC

TCGCCGGCAACCCTTCGCTCGCCGCCGCGCAGGCCCGCGTGCGCGAGGCGCAGTCGCTCGCGCGCATCGCGCACGCCGA

GGAGCTGCCGCAGTTGAACGGCAACCTGTCGCTGATGCGCCAGCACTGGCCGGACAACGTCTATTACGGCCCGGGCCCG

CTCGCGAACGCCGACACGTGGAACAACACGGGCACGCTGAGCCTGTCGTACCACCTCGATCTGTGGGGCAAGGACAAGC

ACAACGCCGAGCGCGCGCTCGACGCGGCGCGCGCCCGCGCGGCCGACGCGCGTGCCGCGCAGCTCGAGCTCGAGGCGAA

CGTCGTGCGCGCGTACGTCGATTTCGCGAAGAACTACGCGCTCCTCGACATCGCGCACGCGACCTACGAGCGCCAGAAC

GCGCTCGCCGAACTGGCGCGCAAGCGCCTGCGCGCGGGCATCGGCACGCAACTCGAGGTGAGCCAGGCCGAGGCGCCGC

TGCCCGATTATTCGCGGCAGATCGACTCGTATGAAGAGGCGATCCAGCTCGGCCGTCACCAGCTCGCCGCGCTCGCGGG

CAAGGGGCCGGGCGCGGGCGCCTCGCTCGCGCGGCCGAAGCTCGCGCTCGACGCGAACGCCGCCCTGCCGTCCGCGCTG

CCCGCCGAGCTGCTCGGCCGCCGGCCGGACATCGTCGCCGCGCGCTGGATGGTCGACGCGCAGGCGCGCGGCATCGACG

TCGCGAAGGCCGCGTTCTATCCGAACGTCGACCTGATCGCGTCGCTCGGCGGCTTCGCGGTCAGCGCGCCGTTCGCGAC

GTTCCTGCGCGCGATGAACGGCGGCTGGTCGGCCGGCCCCGCGCTCACGCTGCCGATCTTCGAGGGCGGCCGGCTGCGC

GCGCAACTGGGCGTCGCGTCGGCGGGCTACGACCAGGCGGTCGAGCACTACAACCAGACGATCGTCGGCGCGCTGAAGG

ACATCGCCGACAACGTCGTGCGCCTGCATTCGCTCGATTCGCAGCAAAAGGACGCGGCGCGCGCGGTCTCGCTCACGCG

GCGCTCGTACGATCTGTCGCATGCGGGCTTCAGCCGCGGGCTCACCGACTACGTGAACGTGCTCATCGCGCAGAGCCAG

TTGCTGACCGCGCAGGAGAACCAGACCCGCATCGAGGCCGCGCGCCTCGCCGCGCACGCGTCGCTGATGGTCGCGCTCG

GCGGCGGGCTCGAAACCGGGCGCGACGCGCCGCACGACGCGCCCGCGGGCGACGCGCGGCGGACGGGTGCTTCGGGTGC

TTCGGGTGCTTCGGGTGCCTCGGGTGCCTCGGGTGCTTCGAGCACCGCGGGCGCTTCGGCCACCGCATCCGCGTCGGCC

GCCGGCCACGCGCCGGCTGGCGCCGCTGCTCCGGCATCGCCCGCCGGCATCCGCGCGGCCGCCAGCGCTCGCGCGTCAA

TGCCGGCTCCCGCCGCGGCGGCGACCGCGCCCGCCTTCGCGTCGCCCGTCGCCGGCGCCTCGACGCCGATGCCCGCTGC

GACAGCGGCGGCCCGCGCCGCCCGCTAA

>Bucl8_Bm_China_7.seq Reverse Complement DNA Sequence Untitled Seq #23(1,1863)

TTGCACCATTCTCGGTTTATTCGGCAATCCGCAAAAAAATATAATCGCATCGACTCATCTTTATCCGAAAGGGAGAAAA

TCGTGCAGTCTCCGGCAATGAGAGGGACGCTTGCACTCGCGGTTCTTGCAATCTCATTAATAATGGCCGGATGCGCAAG

CATGGGCGATGTTGCACCGCAAGACAAGCAGGTCGATCCGGCGTCGCTCGACGCGGGCGCGGCGATCGCGGCGGCCGAG

CGCGACGCCGGCTGGCCCGCGGCCGACTGGTGGCGCGCGTATCGCGATCCGCAGCTCGATTCGTGGATCGTCGCGTCGC

TCGCCGGCAACCCTTCGCTCGCCGCCGCGCAGGCCCGCGTGCGCGAGGCGCAGTCGCTCGCGCGCATCGCGCACGCCGA

GGAGCTGCCGCAGTTGAACGGCAACCTGTCGCTGATGCGCCAGCACTGGCCGGACAACGTCTATTACGGCCCGGGCCCG

CTCGCGAACGCCGACACGTGGAACAACACGGGCACGCTGAGCCTGTCGTACCACCTCGATCTGTGGGGCAAGGACAAGC

ACAACGCCGAGCGCGCGCTCGACGCGGCGCGCGCCCGCGCGGCCGACGCGCGTGCCGCGCAGCTCGAGCTCGAGGCGAA

CGTCGTGCGCGCGTACGTCGATTTCGCGAAGAACTACGCGCTCCTCGACATCGCGCACGCGACCTACGAGCGCCAGAAC

GCGCTCGCCGAACTGGCGCGCAAGCGCCTGCGCGCGGGCATCGGCACGCAACTCGAGGTGAGCCAGGCCGAGGCGCCGC

TGCCCGATTATTCGCGGCAGATCGACTCGTATGAAGAGGCGATCCAGCTCGGCCGTCACCAGCTCGCCGCGCTCGCGGG

CAAGGGGCCGGGCGCGGGCGCCTCGCTCGCGCGGCCGAAGCTCGCGCTCGACGCGAACGCCGCCCTGCCGTCCGCGCTG

CCCGCCGAGCTGCTCGGCCGCCGGCCGGACATCGTCGCCGCGCGCTGGATGGTCGACGCGCAGGCGCGCGGCATCGACG

TCGCGAAGGCCGCGTTCTATCCGAACGTCGACCTGATCGCGTCGCTCGGCGGCTTCGCGGTCAGCGCGCCGTTCGCGAC

GTTCCTGCGCGCGATGAACGGCGGCTGGTCGGCCGGCCCCGCGCTCACGCTGCCGATCTTCGAGGGCGGCCGGCTGCGC

GCGCAACTGGGCGTCGCGTCGGCGGGCTACGACCAGGCGGTCGAGCACTACAACCAGACGATCGTCGGCGCGCTGAAGG

ACATCGCCGACAACGTCGTGCGCCTGCATTCGCTCGATTCGCAGCAAAAGGACGCGGCGCGCGCGGTCTCGCTCACGCG

GCGCTCGTACGATCTGTCGCATGCGGGCTTCAGCCGCGGGCTCACCGACTACGTGAACGTGCTCATCGCGCAGAGCCAG

TTGCTGACCGCGCAGGAGAACCAGACCCGCATCGAGGCCGCGCGCCTCGCCGCGCACGCGTCGCTGATGGTCGCGCTCG

GCGGCGGGCTCGAAACCGGGCGCGACGCGCCGCACGACGCGCCCGCGGGCGACGCGCGGCGGACGGGTGCTTCGGGTGC

TTCGGGTGCTTCGGGTGCTTCGGGTGCCTCGGGTGCCTCGGGTGCCTCGGGTGCTTCGAGCACCGCGGGCGCTTCGGCC

ACCGCATCCGCGTCGGCCGCCGGCCACGCGCCGGCTGGCGCCGCTGCTCCGGCATCGCCCGCCGGCATCCGCGCGGCCG

CCAGCGCTCGCGCGTCAATGCCGGCTCCCGCCGCGGCGGCGACCGCGCCCGCCTTCGCGTCGCCCGTCGCCGGCGCCTC

GACGCCGATGCCCGCTGCGACAGCGGCGGCCCGCGCCGCCCGCTAA

>Bucl8_Bm_PRL-20.seq Created: Wednesday, March 26, 2014 11:51 AM

TTGCACCATTCTCGGTTTATTCGGCAATCCGCAAAAAAATATAATCGCATCGACTCATCTTTATCCGAAAGGGAGAAAA

TCGTGCAGTCTCCGGCAATGAGAGGGACGCTTGCACTCGCGGTTCTTGCAATCTCATTAATAATGGCCGGATGCGCAAG

CATGGGCGATGTTGCACCGCAAGACAAGCAGGTCGATCCGGCGTCGCTCGACGCGGGCGCGGCGATCGCGGCGGCCGAG

CGCGACGCCGGCTGGCCCGCGGCCGACTGGTGGCGCGCGTATCGCGATCCGCAGCTCGATTCGTGGATCGTCGCGTCGC

TCGCCGGCAACCCTTCGCTCGCCGCCGCGCAGGCCCGCGTGCGCGAGGCGCAGTCGCTCGCGCGCATCGCGCACGCCGA

GGAGCTGCCGCAGTTGAACGGCAACCTGTCGCTGATGCGCCAGCACTGGCCGGACAACGTCTATTACGGCCCGGGCCCG

CTCGCGAACGCCGACACGTGGAACAACACGGGCACGCTGAGCCTGTCGTACCACCTCGATCTGTGGGGCAAGGACAAGC

ACAACGCCGAGCGCGCGCTCGACGCGGCGCGCGCCCGCGCGGCCGACGCGCGTGCCGCGCAGCTCGAGCTCGAGGCGAA

CGTCGTGCGCGCGTACGTCGATTTCGCGAAGAACTACGCGCTCCTCGACATCGCGCACGCGACCTACGAGCGCCAGAAC

GCGCTCGCCGAACTGGCGCGCAAGCGCCTGCGCGCGGGCATCGGCACGCAACTCGAGGTGAGCCAGGCCGAGGCGCCGC

TGCCCGATTATTCGCGGCAGATCGACTCGTATGAAGAGGCGATCCAGCTCGGCCGTCACCAGCTCGCCGCGCTCGCGGG

CAAGGGGCCGGGCGCGGGCGCCTCGCTCGCGCGGCCGAAGCTCGCGCTCGACGCGAACGCCGCCCTGCCGTCCGCGCTG

CCCGCCGAGCTGCTCGGCCGCCGGCCGGACATCGTCGCCGCGCGCTGGATGGTCGACGCGCAGGCGCGCGGCATCGACG

TCGCGAAGGCCGCGTTCTATCCGAACGTCGACCTGATCGCGTCGCTCGGCGGCTTCGCGGTCAGCGCGCCGTTCGCGAC

GTTCCTGCGCGCGATGAACGGCGGCTGGTCGGCCGGCCCCGCGCTCACGCTGCCGATCTTCGAGGGCGGCCGGCTGCGC

GCGCAACTGGGCGTCGCGTCGGCGGGCTACGACCAGGCGGTCGAGCACTACAACCAGACGATCGTCGGCGCGCTGAAGG

ACATCGCCGACAACGTCGTGCGCCTGCATTCGCTCGATTCGCAGCAAAAGGACGCGGCGCGCGCGGTCTCGCTCACGCG

GCGCTCGTACGATCTGTCGCATGCGGGCTTCAGCCGCGGGCTCACCGACTACGTGAACGTGCTCATCGCGCAGAGCCAG

TTGCTGACCGCGCAGGAGAACCAGACCCGCATCGAGGCCGCGCGCCTCGCCGCGCACGCGTCGCTGATGGTCGCGCTCG

GCGGCGGGCTCGAAACCGGGCGCGACGCGCCGCACGACGCGCCCGCGGGCGACGCGCGGCGGACGGGTGCTTCGGGTGC

TTCGGGTGCCTCGGGTGCTTCGAGCACCGCGGGCGCTTCGGCCACCGCATCCGCGTCGGCCGCCGGCCACGCGCCGGCT

GGCGCCGCTGCTCCGGCATCGCCCGCCGGCATCCGCGCGGCCGCCAGCGCTCGCGCGTCAATGCCGGCTCCCGCCGCGG

CGGCGACCGCGCCCGCCTTCGCGTCGCCCGTCGCCGGCGCCTCGACGCCGATGCCCGCTGCGACAGCGGCGGCCCGCGC

CGCCCGCTAA

>Bucl8_Bm_strain_6.seq Created: Wednesday, March 26, 2014 11:45 AM

TTGCACCATTCTCGGTTTATTCGGCAATCCGCAAAAAAATATAATCGCATCGACTCATCTTTATCCGAAAGGGAGAAAA

TCGTGCAGTCTCCGGCAATGAGAGGGACGCTTGCACTCGCGGTTCTTGCAATCTCATTAATAATGGCCGGATGCGCAAG

CATGGGCGATGTTGCACCGCAAGACAAGCAGGTCGATCCGGCGTCGCTCGACGCGGGCGCGGCGATCGCGGCGGCCGAG

CGCGACGCCGGCTGGCCCGCGGCCGACTGGTGGCGCGCGTATCGCGATCCGCAGCTCGATTCGTGGATCGTCGCGTCGC

TCGCCGGCAACCCTTCGCTCGCCGCCGCGCAGGCCCGCGTGCGCGAGGCGCAGTCGCTCGCGCGCATCGCGCACGCCGA

GGAGCTGCCGCAGTTGAACGGCAACCTGTCGCTGATGCGCCAGCACTGGCCGGACAACGTCTATTACGGCCCGGGCCCG

CTCGCGAACGCCGACACGTGGAACAACACGGGCACGCTGAGCCTGTCGTACCACCTCGATCTGTGGGGCAAGGACAAGC

ACAACGCCGAGCGCGCGCTCGACGCGGCGCGCGCCCGCGCGGCCGACGCGCGTGCCGCGCAGCTCGAGCTCGAGGCGAA

CGTCGTGCGCGCGTACGTCGATTTCGCGAAGAACTACGCGCTCCTCGACATCGCGCACGCGACCTACGAGCGCCAGAAC

GCGCTCGCCGAACTGGCGCGCAAGCGCCTGCGCGCGGGCATCGGCACGCAACTCGAGGTGAGCCAGGCCGAGGCGCCGC

TGCCCGATTATTCGCGGCAGATCGACTCGTATGAAGAGGCGATCCAGCTCGGCCGTCACCAGCTCGCCGCGCTCGCGGG

CAAGGGGCCGGGCGCGGGCGCCTCGCTCGCGCGGCCGAAGCTCGCGCTCGACGCGAACGCCGCCCTGCCGTCCGCGCTG

CCCGCCGAGCTGCTCGGCCGCCGGCCGGACATCGTCGCCGCGCGCTGGATGGTCGACGCGCAGGCGCGCGGCATCGACG

TCGCGAAGGCCGCGTTCTATCCGAACGTCGACCTGATCGCGTCGCTCGGCGGCTTCGCGGTCAGCGCGCCGTTCGCGAC

GTTCCTGCGCGCGATGAACGGCGGCTGGTCGGCCGGCCCCGCGCTCACGCTGCCGATCTTCGAGGGCGGCCGGCTGCGC

GCGCAACTGGGCGTCGCGTCGGCGGGCTACGACCAGGCGGTCGAGCACTACAACCAGACGATCGTCGGCGCGCTGAAGG

ACATCGCCGACAACGTCGTGCGCCTGCATTCGCTCGATTCGCAGCAAAAGGACGCGGCGCGCGCGGTCTCGCTCACGCG

GCGCTCGTACGATCTGTCGCATGCTGGCTTCAGCCGCGGGCTCACCGACTACGTGAACGTGCTCATCGCGCAGAGCCAG

TTGCTGACCGCGCAGGAGAACCAGACCCGCATCGAGGCCGCGCGCCTCGCCGCGCACGCGTCGCTGATGGTCGCGCTCG

GCGGCGGGCTCGAAACCGGGCGCGACGCGCCGCACGACGCGCCCGCGGGCGACGCGCGGCGGACGGGTGCTTCGGGTGC

TTCGGGTGCCTCGGGTGCCTCGGGTGCCTCGGGTGCTTCGAGCACCGCGGGCGCTTCGGCCACCGCATCCGCGTCGGCC

GCCGGCCACGCGCCGGCTGGCGCCGCTGCTCCGGCATCGCCCGCCGGCATCCGCGCGGCCGCCAGCGCTCGCGCGTCAA

TGCCGGCTCCCGCCGCGGCGGCGACCGCGCCCGCCTTCGCGTCGCCCGTCGCCGGCGCCTCGACGCCGATGCCCGCTGC

GACAGCGGCGGCCCGCGCCGCCCGCTAA

>Bucl8_Bm_strain_11.seq Created: Wednesday, March 26, 2014 11:06 AM

TTGCACCATTCTCGGTTTATTCGGCAATCCGCAAAAAAATATAATCGCATCGACTCATCTTTATCCGAAAGGGAGAAAA

TCGTGCAGTCTCCGGCAATGAGAGGGACGCTTGCACTCGCGGTTCTTGCAATCTCATTAATAATGGCCGGATGCGCAAG

CATGGGCGATGTTGCACCGCAAGACAAGCAGGTCGATCCGGCGTCGCTCGACGCGGGCGCGGCGATCGCGGCGGCCGAG

CGCGACGCCGGCTGGCCCGCGGCCGACTGGTGGCGCGCGTATCGCGATCCGCAGCTCGATTCGTGGATCGTCGCGTCGC

TCGCCGGCAACCCTTCGCTCGCCGCCGCGCAGGCCCGCGTGCGCGAGGCGCAGTCGCTCGCGCGCATCGCGCACGCCGA

GGAGCTGCCGCAGTTGAACGGCAACCTGTCGCTGATGCGCCAGCACTGGCCGGACAACGTCTATTACGGCCCGGGCCCG

CTCGCGAACGCCGACACGTGGAACAACACGGGCACGCTGAGCCTGTCGTACCACCTCGATCTGTGGGGCAAGGACAAGC

ACAACGCCGAGCGCGCGCTCGACGCGGCGCGCGCCCGCGCGGCCGACGCGCGTGCCGCGCAGCTCGAGCTCGAGGCGAA

CGTCGTGCGCGCGTACGTCGATTTCGCGAAGAACTACGCGCTCCTCGACATCGCGCACGCGACCTACGAGCGCCAGAAC

GCGCTCGCCGAACTGGCGCGCAAGCGCCTGCGCGCGGGCATCGGCACGCAACTCGAGGTGAGCCAGGCCGAGGCGCCGC

TGCCCGATTATTCGCGGCAGATCGACTCGTATGAAGAGGCGATCCAGCTCGGCCGTCACCAGCTCGCCGCGCTCGCGGG

CAAGGGGCCGGGCGCGGGCGCCTCGCTCGCGCGGCCGAAGCTCGCGCTCGACGCGAACGCCGCCCTGCCGTCCGCGCTG

CCCGCCGAGCTGCTCGGCCGCCGGCCGGACATCGTCGCCGCGCGCTGGATGGTCGACGCGCAGGCGCGCGGCATCGACG

TCGCGAAGGCCGCGTTCTATCCGAACGTCGACCTGATCGCGTCGCTCGGCGGCTTCGCGGTCAGCGCGCCGTTCGCGAC

GTTCCTGCGCGCGATGAACGGCGGCTGGTCGGCCGGCCCCGCGCTCACGCTGCCGATCTTCGAGGGCGGCCGGCTGCGC

GCGCAACTGGGCGTCGCGTCGGCGGGCTACGACCAGGCGGTCGAGCACTACAACCAGACGATCGTCGGCGCGCTGAAGG

ACATCGCCGACAACGTCGTGCGCCTGCATTCGCTCGATTCGCAGCAAAAGGACGCGGCGCGCGCGGTCTCGCTCACGCG

GCGCTCGTACGATCTGTCGCATGCGGGCTTCAGCCGCGGGCTCACCGACTACGTGAACGTGCTCATCGCGCAGAGCCAG

TTGCTGACCGCGCAGGAGAACCAGACCCGCATCGAGGCCGCGCGCCTCGCCGCGCACGCGTCGCTGATGGTCGCGCTCG

GCGGCGGGCTCGAAACCGGGCGCGACGCGCCGCACGACGCGCCCGCGGGCGACGCGCGGCGGACGGGTGCTTCGGGTGC

TTCGGGTGCTTCGGGTGCCTCGGGTGCCTCGGGTGCTTCGAGCACCGCGGGCGCTTCGGCCACCGCATCCGCGTCGGCC

GCCGGCCACGCGCCGGCTGGCGCCGCTGCTCCGGCATCGCCCGCCGGCATCCGCGCGGCCGCCAGCGCTCGCGCGTCAA

TGCCGGCTCCCGCCGCGGCGGCGACCGCGCCCGCCTTCGCGTCGCCCGTCGCCGGCGCCTCGACGCCGATGCCCGCTGC

GACAGCGGCGGCCCGCGCCGCCCGCTAA

>Bucl8_Bm_NCTC_10229.seq Created: Friday, January 03, 2014 4:01 PM

ttgcaccattctcggtttattcggcaatccgcaaaaaaatataatcgcatcgactcatctttatccgaaagggagaaaa

tcgtgcagtctccggcaatgagagggacgcttgcactcgcggttcttgcaatctcattaataatggccggatgcgcaag

catgggcgatgttgcaccgcaagacaagcaggtcgatccggcgtcgctcgacgcgggcgcggcgatcgcggcggccgag

cgcgacgccggctggcccgcggccgactggtggcgcgcgtatcgcgatccgcagctcgattcgtggatcgtcgcgtcgc

tcgccggcaacccttcgctcgccgccgcgcaggcccgcgtgcgcgaggcgcagtcgctcgcgcgcatcgcgcacgccga

ggagctgccgcagttgaacggcaacctgtcgctgatgcgccagcactggccggacaacgtctattacggcccgggcccg

ctcgcgaacgccgacacgtggaacaacacgggcacgctgagcctgtcgtaccacctcgatctgtggggcaaggacaagc

acaacgccgagcgcgcgctcgacgcggcgcgcgcccgcgcggccgacgcgcgtgccgcgcagctcgagctcgaggcgaa

cgtcgtgcgcgcgtacgtcgatttcgcgaagaactacgcgctcctcgacatcgcgcacgcgacctacgagcgccagaac

gcgctcgccgaactggcgcgcaagcgcctgcgcgcgggcatcggcacgcaactcgaggtgagccaggccgaggcgccgc

tgcccgattattcgcggcagatcgactcgtatgaagaggcgatccagctcggccgtcaccagctcgccgcgctcgcggg

caaggggccgggcgcgggcgcctcgctcgcgcggccgaagctcgcgctcgacgcgaacgccgccctgccgtccgcgctg

cccgccgagctgctcggccgccggccggacatcgtcgccgcgcgctggatggtcgacgcgcaggcgcgcggcatcgacg

tcgcgaaggccgcgttctatccgaacgtcgacctgatcgcgtcgctcggcggcttcgcggtcagcgcgccgttcgcgac

gttcctgcgcgcgatgaacggcggctggtcggccggccccgcgctcacgctgccgatcttcgagggcggccggctgcgc

gcgcaactgggcgtcgcgtcggcgggctacgaccaggcggtcgagcactacaaccagacgatcgtcggcgcgctgaagg

acatcgccgacaacgtcgtgcgcctgcattcgctcgattcgcagcaaaaggacgcggcgcgcgcggtctcgctcacgcg

gcgctcgtacgatctgtcgcatgcgggcttcagccgcgggctcaccgactacgtgaacgtgctcatcgcgcagagccag

ttgctgaccgcgcaggagaaccagacccgcatcgaggccgcgcgcctcgccgcgcacgcgtcgctgatggtcgcgctcg

gcggcgggctcgaaaccgggcgcgacgcgccgcacgacgcgcccgcgggcgacgcgcggcggacgggtgcttcgggtgc

ttcgggtgcttcgggtgcctcgggtgcctcgggtgcttcgagcaccgcgggcgcttcggccaccgcatccgcgtcggcc

gccggccacgcgccggctggcgccgctgctccggcatcgcccgccggcatccgcgcggccgccagcgctcgcgcgtcaa

tgccggctcccgccgcggcggcgaccgcgcccgccttcgcgtcgcccgtcgccggcgcctcgacgccgatgcccgctgc

gacagcggcggcccgcgccgcccgctaa

>Bucl8_Bm_NCTC_10247.seq Created: Friday, January 03, 2014 6:07 PM

ttgcaccattctcggtttattcggcaatccgcaaaaaaatataatcgcatcgactcatctttatccgaaagggagaaaa

tcgtgcagtctccggcaatgagagggacgcttgcactcgcggttcttgcaatctcattaataatggccggatgcgcaag

catgggcgatgttgcaccgcaagacaagcaggtcgatccggcgtcgctcgacgcgggcgcggcgatcgcggcggccgag

cgcgacgccggctggcccgcggccgactggtggcgcgcgtatcgcgatccgcagctcgattcgtggatcgtcgcgtcgc

tcgccggcaacccttcgctcgccgccgcgcaggcccgcgtgcgcgaggcgcagtcgctcgcgcgcatcgcgcacgccga

ggagctgccgcagttgaacggcaacctgtcgctgatgcgccagcactggccggacaacgtctattacggcccgggcccg

ctcgcgaacgccgacacgtggaacaacacgggcacgctgagcctgtcgtaccacctcgatctgtggggcaaggacaagc

acaacgccgagcgcgcgctcgacgcggcgcgcgcccgcgcggccgacgcgcgtgccgcgcagctcgagctcgaggcgaa

cgtcgtgcgcgcgtacgtcgatttcgcgaagaactacgcgctcctcgacatcgcgcacgcgacctacgagcgccagaac

gcgctcgccgaactggcgcgcaagcgcctgcgcgcgggcatcggcacgcaactcgaggtgagccaggccgaggcgccgc

tgcccgattattcgcggcagatcgactcgtatgaagaggcgatccagctcggccgtcaccagctcgccgcgctcgcggg

caaggggccgggcgcgggcgcctcgctcgcgcggccgaagctcgcgctcgacgcgaacgccgccctgccgtccgcgctg

cccgccgagctgctcggccgccggccggacatcgtcgccgcgcgctggatggtcgacgcgcaggcgcgcggcatcgacg

tcgcgaaggccgcgttctatccgaacgtcgacctgatcgcgtcgctcggcggcttcgcggtcagcgcgccgttcgcgac

gttcctgcgcgcgatgaacggcggctggtcggccggccccgcgctcacgctgccgatcttcgagggcggccggctgcgc

gcgcaactgggcgtcgcgtcggcgggctacgaccaggcggtcgagcactacaaccagacgatcgtcggcgcgctgaagg

acatcgccgacaacgtcgtgcgcctgcattcgctcgattcgcagcaaaaggacgcggcgcgcgcggtctcgctcacgcg

gcgctcgtacgatctgtcgcatgcgggcttcagccgcgggctcaccgactacgtgaacgtgctcatcgcgcagagccag

ttgctgaccgcgcaggagaaccagacccgcatcgaggccgcgcgcctcgccgcgcacgcgtcgctgatggtcgcgctcg

gcggcgggctcgaaaccgggcgcgacgcgccgcacgacgcgcccgcgggcgacgcgcggcggacgggtgcttcgggtgc

ttcgggtgcttcgggtgcctcgggtgcctcgggtgcttcgagcaccgcgggcgcttcggccaccgcatccgcgtcggcc

gccggccacgcgccggctggcgccgctgctccggcatcgcccgccggcatccgcgcggccgccagcgctcgcgcgtcaa

tgccggctcccgccgcggcggcgaccgcgcccgccttcgcgtcgcccgtcgccggcgcctcgacgccgatgcccgctgc

gacagcggcggcccgcgccgcccgctaa

>Bucl8_Bm_FMH.seq Reverse Complement DNA Sequence Untitled Seq #29(1,1863)

TTGCACCATTCTCGGTTTATTCGGCAATCCGCAAAAAAATATAATCGCATCGACTCATCTTTATCCGAAAGGGAGAAAA

TCGTGCAGTCTCCGGCAATGAGAGGGACGCTTGCACTCGCGGTTCTTGCAATCTCATTAATAATGGCCGGATGCGCAAG

CATGGGCGATGTTGCACCGCAAGACAAGCAGGTCGATCCGGCGTCGCTCGACGCGGGCGCGGCGATCGCGGCGGCCGAG

CGCGACGCCGGCTGGCCCGCGGCCGACTGGTGGCGCGCGTATCGCGATCCGCAGCTCGATTCGTGGATCGTCGCGTCGC

TCGCCGGCAACCCTTCGCTCGCCGCCGCGCAGGCCCGCGTGCGCGAGGCGCAGTCGCTCGCGCGCATCGCGCACGCCGA

GGAGCTGCCGCAGTTGAACGGCAACCTGTCGCTGATGCGCCAGCACTGGCCGGACAACGTCTATTACGGCCCGGGCCCG

CTCGCGAACGCCGACACGTGGAACAACACGGGCACGCTGAGCCTGTCGTACCACCTCGATCTGTGGGGCAAGGACAAGC

ACAACGCCGAGCGCGCGCTCGACGCGGCGCGCGCCCGCGCGGCCGACGCGCGTGCCGCGCAGCTCGAGCTCGAGGCGAA

CGTCGTGCGCGCGTACGTCGATTTCGCGAAGAACTACGCGCTCCTCGACATCGCGCACGCGACCTACGAGCGCCAGAAC

GCGCTCGCCGAACTGGCGCGCAAGCGCCTGCGCGCGGGCATCGGCACGCAACTCGAGGTGAGCCAGGCCGAGGCGCCGC

TGCCCGATTATTCGCGGCAGATCGACTCGTATGAAGAGGCGATCCAGCTCGGCCGTCACCAGCTCGCCGCGCTCGCGGG

CAAGGGGCCGGGCGCGGGCGCCTCGCTCGCGCGGCCGAAGCTCGCGCTCGACGCGAACGCCGCCCTGCCGTCCGCGCTG

CCCGCCGAGCTGCTCGGCCGCCGGCCGGACATCGTCGCCGCGCGCTGGATGGTCGACGCGCAGGCGCGCGGCATCGACG

TCGCGAAGGCCGCGTTCTATCCGAACGTCGACCTGATCGCGTCGCTCGGCGGCTTCGCGGTCAGCGCGCCGTTCGCGAC

GTTCCTGCGCGCGATGAACGGCGGCTGGTCGGCCGGCCCCGCGCTCACGCTGCCGATCTTCGAGGGCGGCCGGCTGCGC

GCGCAACTGGGCGTCGCGTCGGCGGGCTACGACCAGGCGGTCGAGCACTACAACCAGACGATCGTCGGCGCGCTGAAGG

ACATCGCCGACAACGTCGTGCGCCTGCATTCGCTCGATTCGCAGCAAAAGGACGCGGCGCGCGCGGTCTCGCTCACGCG

GCGCTCGTACGATCTGTCGCATGCGGGCTTCAGCCGCGGGCTCACCGACTACGTGAACGTGCTCATCGCGCAGAGCCAG

TTGCTGACCGCGCAGGAGAACCAGACCCGCATCGAGGCCGCGCGCCTCGCCGCGCACGCGTCGCTGATGGTCGCGCTCG

GCGGCGGGCTCGAAACCGGGCGCGACGCGCCGCACGACGCGCCCGCGGGCGACGCGCGGCGGACGGGTGCTTCGGGTGC

TTCGGGTGCTTCGGGTGCTTCGGGTGCCTCGGGTGCCTCGGGTGCCTCGGGTGCTTCGAGCACCGCGGGCGCTTCGGCC

ACCGCATCCGCGTCGGCCGCCGGCCACGCGCCGGCTGGCGCCGCTGCTCCGGCATCGCCCGCCGGCATCCGCGCGGCCG

CCAGCGCTCGCGCGTCAATGCCGGCTCCCGCCGCGGCGGCGACCGCGCCCGCCTTCGCGTCGCCCGTCGCCGGCGCCTC

GACGCCGATGCCCGCTGCGACAGCGGCGGCCCGCGCCGCCCGCTAA

>Bucl8_Bm_JHU.seq Reverse Complement DNA Sequence Untitled Seq #26(1,1863)

TTGCACCATTCTCGGTTTATTCGGCAATCCGCAAAAAAATATAATCGCATCGACTCATCTTTATCCGAAAGGGAGAAAA

TCGTGCAGTCTCCGGCAATGAGAGGGACGCTTGCACTCGCGGTTCTTGCAATCTCATTAATAATGGCCGGATGCGCAAG

CATGGGCGATGTTGCACCGCAAGACAAGCAGGTCGATCCGGCGTCGCTCGACGCGGGCGCGGCGATCGCGGCGGCCGAG

CGCGACGCCGGCTGGCCCGCGGCCGACTGGTGGCGCGCGTATCGCGATCCGCAGCTCGATTCGTGGATCGTCGCGTCGC

TCGCCGGCAACCCTTCGCTCGCCGCCGCGCAGGCCCGCGTGCGCGAGGCGCAGTCGCTCGCGCGCATCGCGCACGCCGA

GGAGCTGCCGCAGTTGAACGGCAACCTGTCGCTGATGCGCCAGCACTGGCCGGACAACGTCTATTACGGCCCGGGCCCG

CTCGCGAACGCCGACACGTGGAACAACACGGGCACGCTGAGCCTGTCGTACCACCTCGATCTGTGGGGCAAGGACAAGC

ACAACGCCGAGCGCGCGCTCGACGCGGCGCGCGCCCGCGCGGCCGACGCGCGTGCCGCGCAGCTCGAGCTCGAGGCGAA

CGTCGTGCGCGCGTACGTCGATTTCGCGAAGAACTACGCGCTCCTCGACATCGCGCACGCGACCTACGAGCGCCAGAAC

GCGCTCGCCGAACTGGCGCGCAAGCGCCTGCGCGCGGGCATCGGCACGCAACTCGAGGTGAGCCAGGCCGAGGCGCCGC

TGCCCGATTATTCGCGGCAGATCGACTCGTATGAAGAGGCGATCCAGCTCGGCCGTCACCAGCTCGCCGCGCTCGCGGG

CAAGGGGCCGGGCGCGGGCGCCTCGCTCGCGCGGCCGAAGCTCGCGCTCGACGCGAACGCCGCCCTGCCGTCCGCGCTG

CCCGCCGAGCTGCTCGGCCGCCGGCCGGACATCGTCGCCGCGCGCTGGATGGTCGACGCGCAGGCGCGCGGCATCGACG

TCGCGAAGGCCGCGTTCTATCCGAACGTCGACCTGATCGCGTCGCTCGGCGGCTTCGCGGTCAGCGCGCCGTTCGCGAC

GTTCCTGCGCGCGATGAACGGCGGCTGGTCGGCCGGCCCCGCGCTCACGCTGCCGATCTTCGAGGGCGGCCGGCTGCGC

GCGCAACTGGGCGTCGCGTCGGCGGGCTACGACCAGGCGGTCGAGCACTACAACCAGACGATCGTCGGCGCGCTGAAGG

ACATCGCCGACAACGTCGTGCGCCTGCATTCGCTCGATTCGCAGCAAAAGGACGCGGCGCGCGCGGTCTCGCTCACGCG

GCGCTCGTACGATCTGTCGCATGCGGGCTTCAGCCGCGGGCTCACCGACTACGTGAACGTGCTCATCGCGCAGAGCCAG

TTGCTGACCGCGCAGGAGAACCAGACCCGCATCGAGGCCGCGCGCCTCGCCGCGCACGCGTCGCTGATGGTCGCGCTCG

GCGGCGGGCTCGAAACCGGGCGCGACGCGCCGCACGACGCGCCCGCGGGCGACGCGCGGCGGACGGGTGCTTCGGGTGC

TTCGGGTGCTTCGGGTGCTTCGGGTGCCTCGGGTGCCTCGGGTGCCTCGGGTGCTTCGAGCACCGCGGGCGCTTCGGCC

ACCGCATCCGCGTCGGCCGCCGGCCACGCGCCGGCTGGCGCCGCTGCTCCGGCATCGCCCGCCGGCATCCGCGCGGCCG

CCAGCGCTCGCGCGTCAATGCCGGCTCCCGCCGCGGCGGCGACCGCGCCCGCCTTCGCGTCGCCCGTCGCCGGCGCCTC

GACGCCGATGCCCGCTGCGACAGCGGCGGCCCGCGCCGCCCGCTAA

>Bucl8_Bm_ATCC_10399.seq Reverse Complement DNA Sequence Untitled Seq #52(1,1836)

TTGCACCATTCTCGGTTTATTCGGCAATCCGCAAAAAAATATAATCGCATCGACTCATCTTTATCCGAAAGGGAGAAAA

TCGTGCAGTCTCCGGCAATGAGAGGGACGCTTGCACTCGCGGTTCTTGCAATCTCATTAATAATGGCCGGATGCGCAAG

CATGGGCGATGTTGCACCGCAAGACAAGCAGGTCGATCCGGCGTCGCTCGACGCGGGCGCGGCGATCGCGGCGGCCGAG

CGCGACGCCGGCTGGCCCGCGGCCGACTGGTGGCGCGCGTATCGCGATCCGCAGCTCGATTCGTGGATCGTCGCGTCGC

TCGCCGGCAACCCTTCGCTCGCCGCCGCGCAGGCCCGCGTGCGCGAGGCGCAGTCGCTCGCGCGCATCGCGCACGCCGA

GGAGCTGCCGCAGTTGAACGGCAACCTGTCGCTGATGCGCCAGCACTGGCCGGACAACGTCTATTACGGCCCGGGCCCG

CTCGCGAACGCCGACACGTGGAACAACACGGGCACGCTGAGCCTGTCGTACCACCTCGATCTGTGGGGCAAGGACAAGC

ACAACGCCGAGCGCGCGCTCGACGCGGCGCGCGCCCGCGCGGCCGACGCGCGTGCCGCGCAGCTCGAGCTCGAGGCGAA

CGTCGTGCGCGCGTACGTCGATTTCGCGAAGAACTACGCGCTCCTCGACATCGCGCACGCGACCTACGAGCGCCAGAAC

GCGCTCGCCGAACTGGCGCGCAAGCGCCTGCGCGCGGGCATCGGCACGCAACTCGAGGTGAGCCAGGCCGAGGCGCCGC

TGCCCGATTATTCGCGGCAGATCGACTCGTATGAAGAGGCGATCCAGCTCGGCCGTCACCAGCTCGCCGCGCTCGCGGG

CAAGGGGCCGGGCGCGGGCGCCTCGCTCGCGCGGCCGAAGCTCGCGCTCGACGCGAACGCCGCCCTGCCGTCCGCGCTG

CCCGCCGAGCTGCTCGGCCGCCGGCCGGACATCGTCGCCGCGCGCTGGATGGTCGACGCGCAGGCGCGCGGCATCGACG

TCGCGAAGGCCGCGTTCTATCCGAACGTCGACCTGATCGCGTCGCTCGGCGGCTTCGCGGTCAGCGCGCCGTTCGCGAC

GTTCCTGCGCGCGATGAACGGCGGCTGGTCGGCCGGCCCCGCGCTCACGCTGCCGATCTTCGAGGGCGGCCGGCTGCGC

GCGCAACTGGGCGTCGCGTCGGCGGGCTACGACCAGGCGGTCGAGCACTACAACCAGACGATCGTCGGCGCGCTGAAGG

ACATCGCCGACAACGTCGTGCGCCTGCATTCGCTCGATTCGCAGCAAAAGGACGCGGCGCGCGCGGTCTCGCTCACGCG

GCGCTCGTACGATCTGTCGCATGCGGGCTTCAGCCGCGGGCTCACCGACTACGTGAACGTGCTCATCGCGCAGAGCCAG

TTGCTGACCGCGCAGGAGAACCAGACCCGCATCGAGGCCGCGCGCCTCGCCGCGCACGCGTCGCTGATGGTCGCGCTCG

GCGGCGGGCTCGAAACCGGGCGCGACGCGCCGCACGACGCGCCCGCGGGCGACGCGCGGCGGACGGGTGCTTCGGGTGC

TTCGGGTGCTTCGGGTGCTTCGGGTGCTTCGAGCACCGCGGGCGCTTCGGCCACCGCATCCGCGTCGGCCGCCGGCCAC

GCGCCGGCTGGCGCCGCTGCTCCGGCATCGCCCGCCGGCATCCGCGCGGCCGCCAGCGCTCGCGCGTCAATGCCGGCTC

CCGCCGCGGCGGCGACCGCGCCCGCCTTCGCGTCGCCCGTCGCCGGCGCCTCGACGCCGATGCCCGCTGCGACAGCGGC

GGCCCGCGCCGCCCGCTAA

>Bucl8_Bm_GB8_horse_4.seq Reverse Complement DNA Sequence Untitled Seq #32(1,1863)

TTGCACCATTCTCGGTTTATTCGGCAATCCGCAAAAAAATATAATCGCATCGACTCATCTTTATCCGAAAGGGAGAAAA

TCGTGCAGTCTCCGGCAATGAGAGGGACGCTTGCACTCGCGGTTCTTGCAATCTCATTAATAATGGCCGGATGCGCAAG

CATGGGCGATGTTGCACCGCAAGACAAGCAGGTCGATCCGGCGTCGCTCGACGCGGGCGCGGCGATCGCGGCGGCCGAG

CGCGACGCCGGCTGGCCCGCGGCCGACTGGTGGCGCGCGTATCGCGATCCGCAGCTCGATTCGTGGATCGTCGCGTCGC

TCGCCGGCAACCCTTCGCTCGCCGCCGCGCAGGCCCGCGTGCGCGAGGCGCAGTCGCTCGCGCGCATCGCGCACGCCGA

GGAGCTGCCGCAGTTGAACGGCAACCTGTCGCTGATGCGCCAGCACTGGCCGGACAACGTCTATTACGGCCCGGGCCCG

CTCGCGAACGCCGACACGTGGAACAACACGGGCACGCTGAGCCTGTCGTACCACCTCGATCTGTGGGGCAAGGACAAGC

ACAACGCCGAGCGCGCGCTCGACGCGGCGCGCGCCCGCGCGGCCGACGCGCGTGCCGCGCAGCTCGAGCTCGAGGCGAA

CGTCGTGCGCGCGTACGTCGATTTCGCGAAGAACTACGCGCTCCTCGACATCGCGCACGCGACCTACGAGCGCCAGAAC

GCGCTCGCCGAACTGGCGCGCAAGCGCCTGCGCGCGGGCATCGGCACGCAACTCGAGGTGAGCCAGGCCGAGGCGCCGC

TGCCCGATTATTCGCGGCAGATCGACTCGTATGAAGAGGCGATCCAGCTCGGCCGTCACCAGCTCGCCGCGCTCGCGGG

CAAGGGGCCGGGCGCGGGCGCCTCGCTCGCGCGGCCGAAGCTCGCGCTCGACGCGAACGCCGCCCTGCCGTCCGCGCTG

CCCGCCGAGCTGCTCGGCCGCCGGCCGGACATCGTCGCCGCGCGCTGGATGGTCGACGCGCAGGCGCGCGGCATCGACG

TCGCGAAGGCCGCGTTCTATCCGAACGTCGACCTGATCGCGTCGCTCGGCGGCTTCGCGGTCAGCGCGCCGTTCGCGAC

GTTCCTGCGCGCGATGAACGGCGGCTGGTCGGCCGGCCCCGCGCTCACGCTGCCGATCTTCGAGGGCGGCCGGCTGCGC

GCGCAACTGGGCGTCGCGTCGGCGGGCTACGACCAGGCGGTCGAGCACTACAACCAGACGATCGTCGGCGCGCTGAAGG

ACATCGCCGACAACGTCGTGCGCCTGCATTCGCTCGATTCGCAGCAAAAGGACGCGGCGCGCGCGGTCTCGCTCACGCG

GCGCTCGTACGATCTGTCGCATGCGGGCTTCAGCCGCGGGCTCACCGACTACGTGAACGTGCTCATCGCGCAGAGCCAG

TTGCTGACCGCGCAGGAGAACCAGACCCGCATCGAGGCCGCGCGCCTCGCCGCGCACGCGTCGCTGATGGTCGCGCTCG

GCGGCGGGCTCGAAACCGGGCGCGACGCGCCGCACGACGCGCCCGCGGGCGACGCGCGGCGGACGGGTGCTTCGGGTGC

TTCGGGTGCTTCGGGTGCTTCGGGTGCCTCGGGTGCCTCGGGTGCCTCGGGTGCTTCGAGCACCGCGGGCGCTTCGGCC

ACCGCATCCGCGTCGGCCGCCGGCCACGCGCCGGCTGGCGCCGCTGCTCCGGCATCGCCCGCCGGCATCCGCGCGGCCG

CCAGCGCTCGCGCGTCAATGCCGGCTCCCGCCGCGGCGGCGACCGCGCCCGCCTTCGCGTCGCCCGTCGCCGGCGCCTC

GACGCCGATGCCCGCTGCGACAGCGGCGGCCCGCGCCGCCCGCTAA

>Bucl8_Bm_ATCC_23344.seq Created: Friday, January 03, 2014 1:49 PM

ttgcaccattctcggtttattcggcaatccgcaaaaaaatataatcgcatcgactcatctttatccgaaagggagaaaa

tcgtgcagtctccggcaatgagagggacgcttgcactcgcggttcttgcaatctcattaataatggccggatgcgcaag

catgggcgatgttgcaccgcaagacaagcaggtcgatccggcgtcgctcgacgcgggcgcggcgatcgcggcggccgag

cgcgacgccggctggcccgcggccgactggtggcgcgcgtatcgcgatccgcagctcgattcgtggatcgtcgcgtcgc

tcgccggcaacccttcgctcgccgccgcgcaggcccgcgtgcgcgaggcgcagtcgctcgcgcgcatcgcgcacgccga

ggagctgccgcagttgaacggcaacctgtcgctgatgcgccagcactggccggacaacgtctattacggcccgggcccg

ctcgcgaacgccgacacgtggaacaacacgggcacgctgagcctgtcgtaccacctcgatctgtggggcaaggacaagc

acaacgccgagcgcgcgctcgacgcggcgcgcgcccgcgcggccgacgcgcgtgccgcgcagctcgagctcgaggcgaa

cgtcgtgcgcgcgtacgtcgatttcgcgaagaactacgcgctcctcgacatcgcgcacgcgacctacgagcgccagaac

gcgctcgccgaactggcgcgcaagcgcctgcgcgcgggcatcggcacgcaactcgaggtgagccaggccgaggcgccgc

tgcccgattattcgcggcagatcgactcgtatgaagaggcgatccagctcggccgtcaccagctcgccgcgctcgcggg

caaggggccgggcgcgggcgcctcgctcgcgcggccgaagctcgcgctcgacgcgaacgccgccctgccgtccgcgctg

cccgccgagctgctcggccgccggccggacatcgtcgccgcgcgctggatggtcgacgcgcaggcgcgcggcatcgacg

tcgcgaaggccgcgttctatccgaacgtcgacctgatcgcgtcgctcggcggcttcgcggtcagcgcgccgttcgcgac

gttcctgcgcgcgatgaacggcggctggtcggccggccccgcgctcacgctgccgatcttcgagggcggccggctgcgc

gcgcaactgggcgtcgcgtcggcgggctacgaccaggcggtcgagcactacaaccagacgatcgtcggcgcgctgaagg

acatcgccgacaacgtcgtgcgcctgcattcgctcgattcgcagcaaaaggacgcggcgcgcgcggtctcgctcacgcg

gcgctcgtacgatctgtcgcatgcgggcttcagccgcgggctcaccgactacgtgaacgtgctcatcgcgcagagccag

ttgctgaccgcgcaggagaaccagacccgcatcgaggccgcgcgcctcgccgcgcacgcgtcgctgatggtcgcgctcg

gcggcgggctcgaaaccgggcgcgacgcgccgcacgacgcgcccgcgggcgacgcgcggcggacgggtgcttcgggtgc

ttcgggtgcttcgggtgcttcgggtgcctcgggtgcctcgggtgcctcgggtgcttcgagcaccgcgggcgcttcggcc

accgcatccgcgtcggccgccggccacgcgccggctggcgccgctgctccggcatcgcccgccggcatccgcgcggccg

ccagcgctcgcgcgtcaatgccggctcccgccgcggcggcgaccgcgcccgccttcgcgtcgcccgtcgccggcgcctc

gacgccgatgcccgctgcgacagcggcggcccgcgccgcccgctaa

>Bucl8_Bm_SAVP1.seq Created: Saturday, January 04, 2014 1:10 PM

ttgcaccattctcggtttattcggcaatccgcaaaaaaatataatcgcatcgactcatctttatccgaaagggagaaaa

tcgtgcagtctccggcaatgagagggacgcttgcactcgcggttcttgcaatctcattaataatggccggatgcgcaag

catgggcgatgttgcaccgcaagacaagcaggtcgatccggcgtcgctcgacgcgggcgcggcgatcgcggcggccgag

cgcgacgccggctggcccgcggccgactggtggcgcgcgtatcgcgatccgcagctcgattcgtggatcgtcgcgtcgc

tcgccggcaacccttcgctcgccgccgcgcaggcccgcgtgcgcgaggcgcagtcgctcgcgcgcatcgcgcacgccga

ggagctgccgcagttgaacggcaacctgtcgctgatgcgccagcactggccggacaacgtctattacggcccgggcccg

ctcgcgaacgccgacacgtggaacaacacgggcacgctgagcctgtcgtaccacctcgatctgtggggcaaggacaagc

acaacgccgagcgcgcgctcgacgcggcgcgcgcccgcgcggccgacgcgcgtgccgcgcagctcgagctcgaggcgaa

cgtcgtgcgcgcgtacgtcgatttcgcgaagaactacgcgctcctcgacatcgcgcacgcgacctacgagcgccagaac

gcgctcgccgaactggcgcgcaagcgcctgcgcgcgggcatcggcacgcaactcgaggtgagccaggccgaggcgccgc

tgcccgattattcgcggcagatcgactcgtatgaagaggcgatccagctcggccgtcaccagctcgccgcgctcgcggg

caaggggccgggcgcgggcgcctcgctcgcgcggccgaagctcgcgctcgacgcgaacgccgccctgccgtccgcgctg

cccgccgagctgctcggccgccggccggacatcgtcgccgcgcgctggatggtcgacgcgcaggcgcgcggcatcgacg

tcgcgaaggccgcgttctatccgaacgtcgacctgatcgcgtcgctcggcggcttcgcggtcagcgcgccgttcgcgac

gttcctgcgcgcgatgaacggcggctggtcggccggccccgcgctcacgctgccgatcttcgagggcggccggctgcgc

gcgcaactgggcgtcgcgtcggcgggctacgaccaggcggtcgagcactacaaccagacgatcgtcggcgcgctgaagg

acatcgccgacaacgtcgtgcgcctgcattcgctcgattcgcagcaaaaggacgcggcgcgcgcggtctcgctcacgcg

gcgctcgtacgatctgtcgcatgcgggcttcagccgcgggctcaccgactacgtgaacgtgctcatcgcgcagagccag

ttgctgaccgcgcaggagaaccagacccgcatcgaggccgcgcgcctcgccgcgcacgcgtcgctgatggtcgcgctcg

gcggcgggctcgaaaccgggcgcgacgcgccgcacgacgcgcccgcgggcgacgcgcggcggacgggtgcttcgggtgc

ttcgggtgcctcgggtgcttcgagcaccgcgggcgcttcggccaccgcatccgcgtcggccgccggccacgcgccggct

ggcgccgctgctccggcatcgcccgccggcatccgcgcggccgccagcgctcgcgcgtcaatgccggctcccgccgcgg

cggcgaccgcgcccgccttcgcgtcgcccgtcgccggcgcctcgacgccgatgcccgctgcgacagcggcggcccgcgc

cgcccgctaa

>Bucl8_Bt_E264.seq Created: Sunday, September 22, 2013 6:18 PM

TTGCACCATTGTCGGTTTATTCGGCAATCCGCAAAAAAATATAATCGTACCCGACTCATCTTTATCCGAAAGGGAGAAA

ATCGTGCAGTCTCCGGCAACAAGAGGGACGTTTGCACTCGCGGTTCTTGCAATCTCATTAATAATGGCCGGATGTGCAA

GCATGGGCGATGTTGCACCGCAAGCAAAGCAGATCGATCCGGCGTCGCTCGATGCGGGCGCGGCGATCGCGGCGGCCGA

GCGCGACGCGGGCTGGCCCGCGGCCGACTGGTGGCGCGCGTATCGCGATCCGCAGCTCGACGCGTGGATCGCCGCGTCG

CTCGCCGGCAACCCGTCGCTTGCCGCCGCGCAGGCCCGCGTGCGCGAGGCGCAGTCGCTCGCGCGCGTCGCGCATGCCG

AGGAACTGCCGCAGTTGAACGGCAACCTGTCACTGATGCGCCAGCACTGGCCGGACAACGTCTACTACGGCCCAGGCCC

GCTCGCGAACGCCGACACCTGGAACAACACGGGCACGTTGAGCCTGTCGTACCACCTCGACCTCTGGGGCAAGGACAAG

AACAACGCGCAGCGCGCGCTCGACGCGGCGCGCGCCCGCGCGGCCGACGCGCGCGCCGCGCAGCTCGAGCTCGAAGCGA

ACGTGGTGCGCGCGTACATCGATTTCGCAAAGAACTACGCGCTCCTCGACATCGCGCACGCGACCTACGACCGCCAGAA

CGAGCTCGCCGAACTGGCGCGCAAGCGCCTGCGCGCGGGCATCGGCACGCAGCTCGAAGTGAGCCAGGCCGAGGCGCCG

CTGCCCGATTATTCGCGGCAGATCGATTCGTACGAAGAGGCGATCCAGTTGGGCCGCCATCAGCTCGCCGCGCTCGCGG

GCAAGGGGCCCGGCGCGGGCGCGTCGCTCGGGCGGCCGAAGCTCGCGCTCGACGCGAACGCCGGCCTGCCGTCCGCGCT

GCCCGCGGAGTTGATCGGCCGCCGGCCGGACGTCGTCGCCGCGCGCTGGACGGTCGACGCGCAGGCGCGCGGCATCGAC

GTCGCGAAGGCCGCGTTCTATCCGAACGTCGACCTGATCGCGTCGCTCGGCGGCTTCGCGGTCAGCGCGCCGTTCGCGA

CGTTCCTGCGCGCGATGAACGGCGGCTGGTCGGCGGGCCCCGCGCTCACGCTGCCGATCTTCGAGGGCGGCCGGCTGCG

CGCGCAACTGGGCGTCGCCGCGGCGGGCTACGACCAAGCGGTCGAGCACTACAACCAGACGATCGTCGGCGCGCTGAAG

GACATCGCCGACAACGTCGTGCGCCTGCGCTCGCTCGACTCGCAACAGAAGGACGCGGCGCGCGCGGTCTCGCTCACGC

GGCGATCGTACGATCTTTCGCACACGGGCTTCAGCCGCGGGCTCACCGACTACGTGAACGTGCTCATCGCGCAGAGCCA

GTTGCTGACCGCGCAGGAGAACCAGACGCGCGTCGAGGCCGCGCGTCTTGCCGCGCACGCGTCGCTGATGGCCGCGCTC

GGCGGCGGGCTCGAAACCGCGGGCGACGCACCGCATGACACGCCGGCGGGCGATGCGCGGCGCGCGGACGCTTCGGACG

CTTCGGCGGCCTCGTCCGCTTCGGCCGCCGCATCCGTGTCGGGCGCGCCGGCAGGAAGCCGTGCCGCCGGCATCGGCGC

TGCGGTCTCGGCCGGCGCCGACCGCGCAACCGACAATACGCGCGCATCGGCCACCACGCCCGCTGCAGCCCCCGCGCAC

GCCGCTCGCGCGCGGGCGCCGGCGAGCGCCGCCGCGCCCGCCATCCGCTAA

>Bucl8_Bt_2002721723.seq Reverse Complement DNA Sequence CP004097.seq(1,1789)

TTGCACCATTGTCGGTTTATTCGGCAATCCGCAAAAAAATATAATCGTACCCGACTCATCTTTATCCGAAAGGGAGAAA

ATCGTGCAGTCTCCGGCAACAAGAGGGACGTTTGCACTCGCGGTTCTTGCAATCTCATTAATAATGGCCGGATGTGCAA

GCATGGGCGATGTTGCACCGCAAGCAAAGCAGATCGATCCGGCGTCGCTCGATGCGGGCGCGGCGATCGCGGCGGCCGA

GCGCGACGCGGGCTGGCCCGCGGCCGACTGGTGGCGCGCGTATCGCGATCCGCAGCTCGACGCGTGGATCGCCGCGTCG

CTCGCCGGCAACCCGTCGCTTGCCGCCGCGCAGGCCCGCGTGCGCGAGGCGCAGTCGCTCGCGCGCGTCGCGCATGCCG

AGGAACTGCCGCAGTTGAACGGCAACCTGTCACTGATGCGCCAGCACTGGCCGGACAACGTCTACTACGGCCCAGGCCC

GCTCGCGAACGCCGACACCTGGAACAACACGGGCACGTTGAGCCTGTCGTACCACCTCGACCTCTGGGGCAAGGACAAG

AACAACGCGCAGCGCGCGCTCGACGCGGCGCGCGCCCGCGCGGCCGACGCGCGCGCCGCGCAGCTCGAGCTCGAAGCGA

ACGTGGTGCGCGCGTACATCGATTTCGCAAAGAACTACGCGCTCCTCGACATCGCGCACGCGACCTACGACCGCCAGAA

CGAGCTCGCCGAACTGGCGCGCAAGCGCCTGCGCGCGGGCATCGGCACGCAGCTCGAAGTGAGCCAGGCCGAGGCGCCG

CTGCCCGATTATTCGCGGCAGATCGATTCGTACGAAGAGGCGATCCAGTTGGGCCGCCATCAGCTCGCCGCGCTCGCGG

GCAAGGGGCCCGGCGCGGGCGCGTCGCTCGGGCGGCCGAAGCTCGCGCTCGACGCGAACGCCGGCCTGCCGTCCGCGCT

GCCCGCGGAGTTGATCGGCCGCCGGCCGGACGTCGTCGCCGCGCGCTGGACGGTCGACGCGCAGGCGCGCGGCATCGAC

GTCGCGAAGGCCGCGTTCTATCCGAACGTCGACCTGATCGCGTCGCTCGGCGGCTTCGCGGTCAGCGCGCCGTTCGCGA

CGTTCCTGCGCGCGATGAACGGCGGCTGGTCGGCGGGCCCCGCGCTCACGCTGCCGATCTTCGAGGGCGGCCGGCTGCG

CGCGCAACTGGGCGTCGCCGCGGCGGGCTACGACCAAGCGGTCGAGCACTACAACCAGACGATCGTCGGCGCGCTGAAG

GACATCGCCGACAACGTCGTGCGCCTGCGCTCGCTCGACTCGCAACAGAAGGACGCGGCGCGCGCGGTCTCGCTCACGC

GGCGATCGTACGATCTTTCGCACACGGGCTTCAGCCGCGGGCTCACCGACTACGTGAACGTGCTCATCGCGCAGAGCCA

GTTGCTGACCGCGCAGGAGAACCAGACGCGCGTCGAGGCCGCGCGTCTTGCCGCGCACGCGTCGCTGATGGCCGCGCTC

GGCGGCGGGCTCGAAACCGCGGGCGACGCACCGCATGACACGCCGGCGGGCGATGCGCGGCGCGCGGACGCTTCGGACG

CTTCGGCGGCCTCGTCCGCTTCGGCCGCCGCATCCGTGTCGGGCGCGCCGGCAGGAAGCCGTGCCGCCGGCATCGGCGC

TGCGGTCTCGGCCGGCGCCGACCGCGCAACCGACAATACGCGCGCATCGGCCACCACGCCCGCTGCAGCCCCCGCGCAC

GCCGCTCGCGCGCGGGCGCCGGCGAGCGCCGCCGCGCCCGCCATCCGCTAA

>Bucl8_Bt_E444.seq >gi|584105623|gb|CP004117.1|:1213834-1215622 Burkholderia thailandensis E444 chromosome 1, complete sequence

TTGCACCATTGTCGGTTTATTCGGCAATCCGCAAAAAAATATAATCGTACCCGACTCATCTTTATCCGAAAGGGAGAAA

ATCGTGCAGTCTCCGGCAACAAGAGGGACGTTTGCACTCGCGGTTCTTGCAATCTCATTAATAATGGCCGGATGTGCAA

GCATGGGCGATGTTGCACCGCAAGCAAAGCAGATCGATCCGGCGTCGCTCGATGCGGGCGCGGCGATCGCGGCGGCCGA

GCGCGACGCGGGCTGGCCCGCGGCCGACTGGTGGCGCGCGTATCGCGATCCGCAGCTCGACGCGTGGATCGCCGCGTCG

CTCGCCGGCAACCCGTCGCTTGCCGCCGCGCAGGCCCGCGTGCGCGAGGCGCAGTCGCTCGCGCGCGTCGCGCATGCCG

AGGAACTGCCGCAGTTGAACGGCAACCTGTCACTGATGCGCCAGCACTGGCCGGACAACGTCTACTACGGCCCAGGCCC

GCTCGCGAACGCCGACACCTGGAACAACACGGGCACGTTGAGCCTGTCGTACCACCTCGACCTCTGGGGCAAGGACAAG

AACAACGCGCAGCGCGCGCTCGACGCGGCGCGCGCCCGCGCGGCCGACGCGCGCGCCGCGCAGCTCGAGCTCGAAGCGA

ACGTGGTGCGCGCGTACATCGATTTCGCAAAGAACTACGCGCTCCTCGACATCGCGCACGCGACCTACGACCGCCAGAA

CGAGCTCGCCGAACTGGCGCGCAAGCGCCTGCGCGCGGGCATCGGCACGCAGCTCGAAGTGAGCCAGGCCGAGGCGCCG

CTGCCCGATTATTCGCGGCAGATCGATTCGTACGAAGAGGCGATCCAGTTGGGCCGCCATCAGCTCGCCGCGCTCGCGG

GCAAGGGGCCCGGCGCGGGCGCGTCGCTCGGGCGGCCGAAGCTCGCGCTCGACGCGAACGCCGGCCTGCCGTCCGCGCT

GCCCGCGGAGTTGATCGGCCGCCGGCCGGACGTCGTCGCCGCGCGCTGGACGGTCGACGCGCAGGCGCGCGGCATCGAC

GTCGCGAAGGCCGCGTTCTATCCGAACGTCGACCTGATCGCGTCGCTCGGCGGCTTCGCGGTCAGCGCGCCGTTCGCGA

CGTTCCTGCGCGCGATGAACGGCGGCTGGTCGGCGGGCCCCGCGCTCACGCTGCCGATCTTCGAGGGCGGCCGGCTGCG

CGCGCAACTGGGCGTCGCCGCGGCGGGCTACGACCAAGCGGTCGAGCACTACAACCAGACGATCGTCGGCGCGCTGAAG

GACATCGCCGACAACGTCGTGCGCCTGCGCTCGCTCGACTCGCAACAGAAGGACGCGGCGCGCGCGGTCTCGCTCACGC

GGCGATCGTACGATCTTTCGCACACGGGCTTCAGCCGCGGGCTCACCGACTACGTGAACGTGCTCATCGCGCAGAGCCA

GTTGCTGACCGCGCAGGAGAACCAGACGCGCGTCGAGGCCGCGCGTCTTGCCGCGCACGCGTCGCTGATGGCCGCGCTC

GGCGGCGGGCTCGAAACCGCGGGCGACGCACCGCATGACACGCCGGCGGGCGATGCGCGGCGCGCGGACGCTTCGGACG

CTTCGGCGGCCTCGTCCGCTTCGGCCGCCGCATCCGTGTCGGGCGCGCCGGCAGGAAGCCGTGCCGCCGGCATCGGCGC

TGCGGTCTCGGCCGGCGCCGACCGCGCAACCGACAATACGCGCGCATCGGCCACCACGCCCGCTGCAGCCCCCGCGCAC

GCCGCTCGCGCGCGGGCGCCGGCGAGCGCCGCCGCGCCCGCCATCCGCTAA

>Bucl8_Bt_E555.seq Created: Wednesday, March 26, 2014 12:19 PM

TTGCACCATTGTCGGTTTATTCGGCAATCCGCAAAAAAATATAATCGTACCCGACTCATCTTTATCCGAAAGGGAGAAA

ATCGTGCAGTCTCCGGCAACAAGAGGGACGTTTGCACTCGCGGTTCTTGCAATCTCATTAATAATGGCCGGATGCGCAA

GCATGGGCGATGTTGCACCGCAAGCAAAGCAGATCGATCCGGCGTCGCTCGATGCGGGCGCGGCGATCGCGGCGGCCGA

GCGCGACGCGGGCTGGCCCGCGGCCGACTGGTGGCGCGCGTATCGCGATCCGCAGCTCGACGCGTGGATCGCCGCGTCG

CTCGCCGGCAACCCGTCGCTCGCCGCCGCGCAGGCCCGCGTGCGCGAGGCGCAGTCGCTCGCGCGCGTCGCGCATGCCG

AGGAACTGCCGCAGTTGAACGGCAACCTGTCGCTGATGCGCCAGCACTGGCCGGACAACGTCTACTACGGCCCGGGCCC

GCTCGCGAACGCCGACACCTGGAACAACACGGGCACGTTGAGCCTGTCGTACCACCTCGACCTCTGGGGCAAGGACAAG

AACAACGCGCAGCGCGCGCTCGACGCGGCGCGCGCCCGCGCGGCCGACGCGCGCGCCGCGCAGCTCGAGCTCGAAGCGA

ACGTGGTGCGCGCGTACATCGATTTCGCAAAGAACTACGCGCTCCTCGACATCGCGCACGCGACCTACGACCGCCAGAA

CGAGCTCGCCGAACTGGCGCGCAAGCGCCTGCGCGCGGGCATCGGCACGCAGCTCGAAGTGAGCCAGGCCGAGGCGCCG

CTGCCCGACTATTCGCGGCAGATCGATTCGTACGAAGAGGCGATCCAGTTGGGCCGCCATCAGCTCGCCGCGCTCGCGG

GCAAGGGGCCCGGCGCGGGCGCGTCGCTCGGGCGGCCGAAGCTCGCGCTCGACGCAAACGCCGGCCTGCCGTCCGCGCT

GCCCGCGGAGTTGATCGGCCGCCGGCCGGACGTCGTCGCCGCGCGCTGGACGGTCGACGCGCAGGCGCGCGGCATCGAC

GTCGCGAAGGCCGCGTTCTATCCGAACGTCGACCTGATCGCGTCGCTCGGCGGCTTCGCGGTCAGCGCGCCGTTCGCGA

CGTTCCTGCGCGCGATGAACGGCGGCTGGTCGGCGGGCCCCGCGCTCACGCTGCCGATCTTCGAGGGCGGCCGGCTGCG

CGCGCAACTGGGCGTCGCCGCGGCGGGCTACGACCAGGCGGTCGAGCACTACAACCAGACGATCGTCGGCGCGCTGAAG

GACATCGCCGACAACGTCGTGCGCCTGCACTCGCTCGACTCGCAACAGAAGGACGCGGCGCGCGCGGTCTCGCTCACGC

GGCGATCGTACGATCTTTCGCACACGGGCTTCAGCCGCGGGCTCACCGACTACGTGAACGTGCTCATCGCGCAGAGCCA

GTTGCTGACCGCGCAGGAAAACCAGACGCGCGTCGAGGCCGCGCGTCTTGCCGCGCACGCGTCGCTGATGACCGCGCTC

GGCGGCGGGTTCGAAACCGCGGGCGACGCACCGCATGACACGCCGGCGGGCGATGCGCGGCGCGCGGGCGCTTCGGGCG

CTTCGGCGGCCTCGTCCGCTTCGGCCGCCGCATCCGTGTCGGGCGCGCCGGCAGGAAGCCGTGCCGCCGGCATCGGCGC

TGCGGCCTC

>Bucl8_Bt_H0587.seq >gi|584091436|gb|CP004089.1|:2588232-2590032 Burkholderia thailandensis H0587 chromosome 1, complete sequence

TTGCACCATTGTCGGTTTATTCGGCAATCCGCAAAAAAATATAATCGTACCCGACTCATCTTTATCCGAAAGGGAGAAA

ATCGTGCAGTCTCCGGCAACAAGAGGGACGTTTGCACTCGCGGTTCTTGCAATCTCATTAATAATGGCCGGATGCGCAA

GCATGGGCGATGTTGCACCGCAAGCAAAGCAGATCGATCCGGCGTCGCTCGATGCGGGCGCGGCGATCGCGGCGGCCGA

GCGCGACGCGGGCTGGCCCGCGGCCGACTGGTGGCGCGCGTATCGCGATCCGCAGCTCGACGCGTGGATCGCCGCGTCG

CTCGCCGGCAACCCGTCGCTCGCCGCCGCGCAGGCCCGCGTGCGCGAGGCGCAGTCGCTCGCGCGCGTCGCGCATGCCG

AGGAACTGCCGCAGTTGAACGGCAACCTGTCGCTGATGCGCCAGCACTGGCCGGACAACGTCTACTACGGCCCGGGCCC

GCTCGCGAACGCCGACACCTGGAACAACACGGGCACGTTGAGCCTGTCGTACCACCTCGACCTCTGGGGCAAGGACAAG

AACAACGCGCAGCGCGCGCTCGACGCGGCGCGCGCCCGCGCGGCCGACGCGCGCGCCGCGCAGCTCGAGCTCGAAGCGA

ACGTGGTGCGCGCGTACATCGATTTCGCAAAGAACTACGCGCTCCTCGACATCGCGCACGCGACCTACGACCGCCAGAA

CGAGCTCGCCGAACTGGCGCGCAAGCGCCTGCGCGCGGGCATCGGCACGCAGCTCGAAGTGAGCCAGGCCGAGGCGCCG

CTGCCCGATTATTCGCGGCAGATCGATTCGTACGAAGAGGCGATCCAGTTGGGCCGCCATCAGCTCGCCGCGCTCGCGG

GCAAGGGGCCCGGCGCGGGCGCGTCGCTCGGGCGGCCGAAGCTCGCGCTCGACGCGAACGCCGGCCTGCCGTCCGCGCT

GCCCGCGGAGTTGATCGGCCGCCGGCCGGACGTCGTCGCCGCGCGCTGGACGGTCGACGCGCAGGCGCGCGGCATCGAC

GTCGCGAAGGCCGCGTTCTATCCGAACGTCGACCTGATCGCGTCGCTCGGCGGCTTCGCGGTCAGCGCGCCGTTCGCGA

CGTTCCTGCGCGCGATGAACGGCGGCTGGTCGGCGGGCCCCGCGCTCACGCTGCCGATCTTCGAGGGCGGCCGGCTGCG

CGCGCAACTGGGCGTCGCCGCGGCGGGCTACGACCAGGCGGTCGAGCACTACAACCAGACGATCGTCGGCGCGCTGAAG

GACATCGCCGACAACGTCGTGCGCCTGCACTCGCTCGACTCGCAACAGAAGGACGCGGCGCGCGCGGTCTCGCTCACGC

GGCGATCGTACGATCTTTCGCACACGGGCTTCAGCCGCGGGCTCACCGACTACGTGAACGTGCTCATCGCGCAGAGCCA

GTTGCTGACCGCGCAGGAAAACCAGACGCGCGTCGAGGCCGCGCGTCTTGCCGCGCACGCGTCGCTGATGACCGCGCTC

GGCGGCGGGCTCGAAACCGCAGGCGACGCACCGCATGACGCGCCGGCGGGCGACGCGCGGCGCGCGGGCGCTTCGGGCG

CTTCGGCGGCCTCGCCCGCTTCGGCCGCCGCATCCGTGTCGGGCGCGCCGGCAGGAAGCCGTGCCGCCGGCATCGGCGC

TGCGGCCTCGGCCGGCGCCGACCGCGCAACCGACAATACGCGCGCATCGGCCACCACGCCCGCTGCAGCCGCCGGGGCA

GCCCCCGCGCACGCCGCTCGCGCGCGGGCGCCGGCGAGCGCCGCCGCGCCCGCCATCCGCTAA

>Bucl8_Bt_MSMB43.seq Reverse Complement DNA Sequence Untitled Seq #69(1,1621)

TTGCACCATTGTCGGTTTATTTGGCAATTCGCAAAAAAATATAATCGCATTCGACTCATCTTTATCCGAAAGGGAGAAA

ATCGTGCAATCTCCGGCAACAAGAGGGACGTTTGCACTCGCGGTTCTTGCAATCTCATCAATAATGGCCGGATGCGCAA

GCATGGGCGATGTTGCACCGCAAGCCAAGCAGATCGATCCGGCGTCGCTCGACGCGGGCGCGGCGATCGCAGCGGCCGA

CCGCGACGCGGGCTGGCCCGCGGCCGACTGGTGGCGCGCGTATCGCGATCCGCAGCTCGATGCGTGGATCGCCGCGTCG

CTCGCCGGCAACCCGTCGCTTGCCGCCGCGCAGGCCCGCGTGCGCGAGGCGCAATCGCTCGCGCGCATCGCGCACGCCG

AGGAGCTGCCGCAGGTGAACGGCAACCTGTCGCTGATGCGCCAGCACTGGCCGGACAACGTCTACTACGGCCCGGGCCC

GCTCGCCAACGCCGACACCTGGAACAACACGGGCACGCTGAGCCTGTCGTACCACCTCGACCTGTGGGGCAAGAACAAG

AACAACGCCGAGCGCGCGCTCGACGCGGCGCGCGCCCGCGCGGCCGACGCGCGCGCCGCGCAGCTCGAGCTCGAGGCGA

ACGTCGTGCGCGCATACATCGATTTCGCGAAGAATTACGCGCTCCTCGACATCGCGCACGCGACCTACGACCGCCAGAA

CGAGCTCGCCGAACTCGCGCGCAAGCGCTTGCGCGCGGGCATCGGCACGCAGCTCGAAGTCAGCCAGGCCGAGGCGCCT

CTGCCCGATTATTCGCAGCAGATCGATTCGTACGAAGAGGCGATCCAGCTCGGCCGGCATCAGCTCGCCGCGCTCGCCG

GCAAGGGGCCGGGCGCGGGCGCGTCGCTCGCGCGGCCGAAGCTCGCGCTCGACGCGAACGCCGGCCTGCCGTCCGCGCT

GCCCGCCGAGCTGATCGGCCGCCGGCCGGACATCGTCGCCGCGCGCTGGACGGTCGACGCGCAGACGCGCGGCATCGAC

GTCGCGAAGGCCGCGTTCTATCCGAACGTCGACCTGATCGCGTCGCTCGGCGGCTTCGCGGTCAGCGCGCCGTTCGCGA

CGTTCCTGCGCGCGATGAACGGCGGCTGGTCGGCGGGCCCCGCGCTCACGCTGCCGATCTTCGAGGGCGGCCGGCTGCG

CGCGCAACTGGGCGTCGCGTCGGCGGGCTACGACGAGGCGGTCGAGCACTACAACCAGACGATCGTCGGCGCGCTCAAG

GACATCGCCGACAACGTCGTGCGCCTGCACTCGCTCGACTCGCAGCAGAAGGACGCGGCGCGCGCGGTCTCGCTCACGC

GGCGCTCGTATGATCTGTCGCACACGGGCTTCAGCCGCGGGCTCACCGACTACGTGAACGTGCTCATCGCGCAGAGCCA

GTTGCTGAGCGCGCAGGAGAGCCAGACGCGCGTCGAGGCCGCGCGTCTCGCCGCGCATGCGTCGCTGATGGTCGCGCTC

GGCGGCGGGCTCGAAACCGCGGACGACGCGCCGCATGATGCGCCTGCAGGCGATGCGCGGCGTGCGGGTGCTTCGGGTG

CTTCGGGCGCTTCGCCCGCATCGGCGAGCGCCGTCCGCTAA

>Bucl8_Bt_4.seq Created: Wednesday, March 26, 2014 12:26 PM

TTGCACCATTGTCGGTTTATTCGGCAATCCGCAAAAAAATATAATCGTACCCGACTCATCTTTATCCGAAAGGGAGAAA

ATCGTGCAGTCTCCGGCAACAAGAGGGACGTTTGCACTCGCGGTTCTTGCAATCTCATTAATAATGGCCGGATGTGCAA

GCATGGGCGATGTTGCACCGCAAGCAAAGCAGATCGATCCGGCGTCGCTCGATGCGGGCGCGGCGATCGCGGCGGCCGA

GCGCGACGCGGGCTGGCCCGCGGCCGACTGGTGGCGCGCGTATCGCGATCCGCAGCTCGACGCGTGGATCGCCGCGTCG

CTCGCCGGCAACCCGTCGCTTGCCGCCGCGCAGGCCCGCGTGCGCGAGGCGCAGTCGCTCGCGCGCGTCGCGCATGCCG

AGGAACTGCCGCAGTTGAACGGCAACCTGTCACTGATGCGCCAGCACTGGCCGGACAACGTCTACTACGGCCCAGGCCC

GCTCGCGAACGCCGACACCTGGAACAACACGGGCACGTTGAGCCTGTCGTACCACCTCGACCTCTGGGGCAAGGACAAG

AACAACGCGCAGCGCGCGCTCGACGCGGCGCGCGCCCGCGCGGCCGACGCGCGCGCCGCGCAGCTCGAGCTCGAAGCGA

ACGTGGTGCGCGCGTACATCGATTTCGCAAAGAACTACGCGCTCCTCGACATCGCGCACGCGACCTACGACCGCCAGAA

CGAGCTCGCCGAACTGGCGCGCAAGCGCCTGCGCGCGGGCATCGGCACGCAGCTCGAAGTGAGCCAGGCCGAGGCGCCG

CTGCCCGATTATTCGCGGCAGATCGATTCGTACGAAGAGGCGATCCAGTTGGGCCGCCATCAGCTCGCCGCGCTCGCGG

GCAAGGGGCCCGGCGCGGGCGCGTCGCTCGGGCGGCCGAAGCTCGCGCTCGACGCGAACGCCGGCCTGCCGTCCGCGCT

GCCCGCGGAGTTGATCGGCCGCCGGCCGGACGTCGTCGCCGCGCGCTGGACGGTCGACGCGCAGGCGCGCGGCATCGAC

GTCGCGAAGGCCGCGTTCTATCCGAACGTCGACCTGATCGCGTCGCTCGGCGGCTTCGCGGTCAGCGCGCCGTTCGCGA

CGTTCCTGCGCGCGATGAACGGCGGCTGGTCGGCGGGCCCCGCGCTCACGCTGCCGATCTTCGAGGGCGGCCGGCTGCG

CGCGCAACTGGGCGTCGCCGCGGCGGGCTACGACCAAGCGGTCGAGCACTACAACCAGACGATCGTCGGCGCGCTGAAG

GACATCGCCGACAACGTCGTGCGCCTGCGCTCGCTCGACTCGCAACAGAAGGACGCGGCGCGCGCGGTCTCGCTCACGC

GGCGATCGTACGATCTTTCGCACACGGGCTTCAGCCGCGGGCTCACCGACTACGTGAACGTGCTCATCGCGCAGAGCCA

GTTGCTGACCGCGCAGGAGAACCAGACGCGCGTCGAGGCCGCGCGTCTTGCCGCGCACGCGTCGCTGATGGCCGCGCTC

GGCGGCGGGCTCGAAACCGCGGGCGACGCGCCGCATGACGCGCCGGCGGGCGATGCGCGGCGCGCGGACGCTTCGGACG

CTTCGGCCGCCGCATCCGTGTCGGGCGCGCCGGCAGGAGGCCGTGCCGCCGGCATCGGCGCTGCGGTCTCGGCCGGCG

>Bucl8_Bt_MSMB_121.seq Created: Thursday, May 22, 2014 11:19 AM

TTGCACCATTGTCGGTTTATTTGGCAATTCGCAAAAAAATATAATCGCATTCGACTCATCTTTATCCGAAAGGGAGAAA

ATCGTGCAGTCTCCGGCAACAAGAGGGACGTTTGCACTCGCGGTTCTTGCAATCTCATCAATAATGGCCGGATGCGCAA

GCATGGGCGATGTTGCACCGCAAGCCAAGCAGATCGATCCGGCGTCGCTCGACGCGGGCGCGGCGATCGCGGCGGCCGA

CCGCGACGCGGGCTGGCCCGCGGCCGACTGGTGGCGCGCGTATCGCGATCCGCAGCTCGATGCGTGGATCGCCGCGTCG

CTCGCCGGCAACCCGTCGCTTGCCGCCGCGCAGGCCCGCGTGCGCGAGGCGCAATCGCTCGCGCGCATCGCGCACGCCG

AGGAGCTGCCGCAGGTGAACGGCAACCTGTCGCTGATGCGCCAGCACTGGCCGGACAACGTCTACTACGGCCCGGGCCC

GCTCGCCAACGCCGACACCTGGAACAACACGGGCACGCTGAGCCTGTCGTACCACCTCGACCTGTGGGGCAAGGACAAG

AACAACGCCGAGCGCGCGCTCGACGCGGCGCGCGCCCGCGCGGCCGACGCGCGCGCCGCGCAGCTCGAGCTCGAGGCGA

ACGTCGTGCGCGCATACATCGATTTCGCGAAGAATTACGCGCTCCTCGACATCGCGCACGCGACCTACGACCGCCAGAA

CGAGCTCGCCGAACTCGCGCGCAAGCGCTTGCGCGCGGGCATCGGCACGCAGCTCGAAGTCAGCCAGGCCGAGGCGCCT

CTGCCCGATTATTCGCGGCAGATCGATTCGTACGAAGAGGCGATCCAGCTCGGCCGGCATCAGCTCGCCGCGCTCGCCG

GCAAGGGGCCGGGCGCGGGCGCGTCGCTCGCGCGGCCGAAGCTCGCGCTCGACGCGAACGCCGGCCTGCCGTCCGCGCT

GCCCGCCGAGCTGATCGGCCGCCGGCCGGACATCGTCGCCGCGCGCTGGACGGTCGACGCGCAGACGCGCGGCATCGAC

GTCGCGAAGGCCGCGTTCTATCCGAACGTCGACCTGATCGCGTCGCTCGGCGGCTTCGCGGTCAGCGCGCCGTTCGCGA

CGTTCCTGCGCGCGATGAACGGCGGCTGGTCGGCGGGCCCCGCGCTCACGCTGCCGATCTTCGAGGGCGGCCGGCTGCG

CGCGCAACTGGGCGTCGCGTCGGCGGGCTACGACGAGGCGGTCGAGCACTACAACCAGACGATCGTCGGCGCGCTCAAG

GACATCGCCGACAACGTCGTGCGCCTGCACTCGCTCGACTCGCAGCAGAAAGACGCGGCGCGCGCGGTCTCGCTCACAC

GGCGCTCGTACGATCTGTCGCACACGGGCTTCAGCCGCGGGCTCACCGACTACGTGAACGTGCTCATCGCGCAGAGCCA

GTTGCTGAGCGCGCAGGAGAGCCAGACGCGCGTCGAGGCCGCGCGTCTCGCCGCGCACGCGTCGCTGATGGTCGCGCTC

GGCGGCGGGCTCGAAACCGCGGGCGACGCGCCGCATGACGCGCCTGCGGGCGATGCGCGGCGTGCGGGCGCTTCGGGTG

CTTCGGGTGCTTCGTCCGCATCGGCGAGCGCCGTCCGCTAA

>B2GYG4_BURPS_Bucl10_Bps_1655.seq Created: Thursday, August 01, 2013 9:07 PM

ATGCACGGCATCCACGGCATCCACGGCATCCACGGCATCCACGGCATCCACGGCATCCACGGCATCCACGGCATCCACG

GCATCCACGGCATCCACGGCATCCACGGCATCCACGGCATCCACGGCATGCGTTGGACACGAGGCAGGCGAGGCAGTTC

GGCGCGGCACGATATGGCGACTCGCGCGGCCGCGCCCGCCGTCGATGCGGCGACGCGACGAGATGACGACGTTGCGCCG

ATGCGGCGTCAATGCGGCGCGCACGATATCGATATCGAAAATGCTTCGTTGTGCAGGGCGGCGACGCACCTTAATCTGG

ACCCGTAG

>COY5B0_BURPS_Bucl10_Bps_Pakistan_9.seq Created: Thursday, August 01, 2013 8:54 PM

ATGCACGGCATCCACGGCATCCACGGCATCCACGGCATCCACGGCATCCACGGCATCCACGGCATCCACGGCATCCACG

GCATCCACGGCATCCACGGCATCCACGGCATCCACGGCATGCGTTGGACACGAGGCAGGCGAGGCAGTTCGGCGCGGCA

CGATATGGCGACTCGCGCGGCCGCGCCCGCCGTCGATGCGGCGACGCGACGAGATGACGAGGTTGCGCCGATGCGGCGT

CAATGCGGCGCGCACGATATCGATATCGAAAATGCTTCGTTGTGCAGGGCGGCGACGCACCTTAATCTGGACCCGTAG

>Q3JSM5_BURP1_Bucl10_Bps_1710b.seq Created: Thursday, August 01, 2013 8:27 PM

ATGTGTTGGATGTGTTGGATGCACGGCATCCACGGCATCCACGGCATCCACGGCATCCACGGCATCCACGGCATCCACG

GCATCCACGGCATCCACGGCATCCACGGCATCCACGGCATCCACGGCATCCACGGCATCCACGGCATCCACGGCATCCA

CGGCATCCACGGCATCCACGGCATGCGTTGGACACGAGGCAGGCGAGGCAGTTCGGCGCGGCACGATATGGCGACTCGC

GCGGCCGCGCCCGCCGTCGATGCGGCGACGCGACGAGATGACGACGTTGCGCCGATGCGGCGTCAATGCGGCGCGCACG

ATATCGATATCGAAAATGCTTCGTTGTGCAGGGCGGCGACGCACCTTAATCTGGACCCGTAG

>Bucl10_Bps_NCTC_13179.seq Created: Thursday, January 02, 2014 3:01 PM

atgtgttggatgcacggcatccacggcatccacggcatccacggcatccacggcatccacggcatccacggcatccacg

gcatccacggcatccacggcatccacggcatccacggcatgcgttggacacgaggcaggcgaggcagttcggcgcggca

cgatatggcgactcgcgcggccgcgcccgccgtcgatgcggcgacgcgacgagatgacgacgttgcgccgatgcggcgt

caatgcggcgcgcacgatatcgatatcgaaaatgcttcgttgtgcagggcggcgacgcaccttaatctgaacccgtag

>Bucl10_Bps_K96243.seq Created: Monday, January 20, 2014 1:28 PM

atgtgttggatgcacggcatccacggcatccacggcatccacggcatccacggcatccacggcatccacggcatccacg

gcatccacggcatccacggcatccacggcatccacggcatccacggcatccacggcatccacggcatccacggcatcca

cggcatccacggcatccacggcatccacggcatccacggcatccacggcatccacggcatccacggcatgcgttggaca

cgaggcaggcgaggcagttcggcgcggcacgatatggcgactcgcgcggccgcgcccgccgtcgatgcggcgacgcgac

gagatgacgacgttgcgccgatgcggcgtcaatgcggcgcgcacgatatcgatatcgaaaatgcttcgttgtgcagggc

ggcgacgcaccttaatctggacccgtag

>Bucl10_Bps_1026b.seq Created: Monday, January 20, 2014 1:38 PM

atgtgttggatgcacggcatccacggcatccacggcatccacggcatccacggcatccacggcatccacggcatccacg

gcatccacggcatccacggcatccacggcatccacggcatccacggcatccacggcatccacggcatccacggcatgcg

ttggacacgaggcaggcgaggcagttcggcgcggcacgatatggcgactcgcgcggccgcgcccgccgtcgatgcggcg

acgcgacgagatgacgacgttgcgccgatgcggcgtcaatgcggcgcgcacgatatcgatatcgaaaatgcttcgttgt

gcagggcggcgacgcaccttaatctggacccgtag

>A1V459_BURMS_Bucl10_Bm_SAVP1.seq Created: Monday, January 20, 2014 1:47 PM

atgtgttggatgcacggcatgcacggcatgcacggcatgcgcggcatccacggcatccacggcatccacggcatccacg

gcatccacggcatccacggcatccacggcatccacggcatccacggcatccacggcatccacggcatccacggcatcca

cggcatccacggcatccacggcatccacggcatccacggcatccacggcatccacggcatgcgttggacacgaggcagg

cgaggcagttcggcgcggcacgatatggcgactcgcgcggccgcgcccgccgtcgatgcggcgacgcgacgagatgacg

acgttgcgccgatgcggcgtcaatgcggcgcgcacgatatcgatatcgaaaatgcttcgttgtgcagggcggcgacgca

ccttaatctggacccgtag

>A2S325_BURM9_Bucl10_Bm_NCTC_10229.seq Created: Monday, January 20, 2014 1:56 PM

atgtgttggatgcacggcatgcacggcatccacggcatccacggcatccacggcatccacggcatccacggcatccacg

gcatccacggcatccacggcatccacggcatccacggcatccacggcatccacggcatgcgttggacacgaggcaggcg

aggcagttcggcgcggcacgatatggcgactcgcgcggccgcgcccgccgtcgatgcggcgacgcgacgagatgacgac

gttgcgccgatgcggcgtcaatgcggcgcgcacgatatcgatatcgaaaatgcttcgttgtgcagggcggcgacgcacc

ttaatctggacccgtag

>A3MJE6_BURM7_Bucl10_Bm_NCTC_10247.seq Created: Monday, January 20, 2014 2:02 PM

atgtgttggatgcacggcatgcacggcatccacggcatccacggcatccacggcatccacggcatccacggcatccacg

gcatccacggcatccacggcatccacggcatccacggcatccacggcatccacggcatccacggcatccacggcatcca

cggcatccacggcatccacggcatccacggcatccacggcatccacggcatccacggcatgcgttggacacgaggcagg

cgaggcagttcggcgcggcacgatatggcgactcgcgcggccgcgcccgccgtcgatgcggcgacgcgacgagatgacg

acgttgcgccgatgcggcgtcaatgcggcgcgcacgatatcgatatcgaaaatgcttcgttgtgcagggcggcgacgca

ccttaatctggacccgtag

>A3NUX3_BURP0_Bucl10_Bps_1106a.seq Created: Monday, January 20, 2014 2:07 PM

atgtgttggatgcacggcatccacggcatccacggcatccacggcatccacggcatccacggcatccacggcatccacg

gcatccacggcatccacggcatccacggcatccacggcatccacggcatgcgttggacacgaggcaggcgaggcagttc

ggcgcggcacgatatggcgactcgcgcggccgcgcccgccgtcgatgcggcgacgcgacgagatgacgaggttgcgccg

atgcggcgtcaatgcggcgcgcacgatatcgatatcgaaaatgcttcgttgtgcagggcggcgacgcaccttaatctgg

acccgtag

>A5TLC5_BURMA_Bucl10_Bm_2002721280.seq Reverse Complement DNA Sequence Untitled Seq #31(1,405)

ATGTGTTGGATGCACGGCATGCACGGCATGCACGGCATCCACGGCATCCACGGCATCCACGGCATCCACGGCATCCACG

GCATCCACGGCATCCACGGCATCCACGGCATCCACGGCATCCACGGCATCCACGGCATCCACGGCATCCACGGCATCCA

CGGCATCCACGGCATCCACGGCATCCACGGCATCCACGGCATCCACGGCATGCGTTGGACACGAGGCAGGCGAGGCAGT

TCGGCGCGGCACGATATGGCGACTCGCGCGGCCGCGCCCGCCGTCGATGCGGCGACGCGACGAGATGACGACGTTGCGC

CGATGCGGCGTCAATGCGGCGCGCACGATATCGATATCGAAAATGCTTCGTTGTGCAGGGCGGCGACGCACCTTAATCT

GGACCCGTAG

>A9K814_BURMA_Bucl10_Bm_ATCC_10399.seq Created: Monday, January 20, 2014 2:39 PM

ATGTGTTGGATGCACGGCATGCACGGCATGCACGGCATGCGCGGCATCCACGGCATCCACGGCATCCACGGCATCCACG

GCATCCACGGCATCCACGGCATCCACGGCATCCACGGCATCCACGGCATCCACGGCATCCACGGCATCCACGGCATCCA

CGGCATCCACGGCATCCACGGCATCCACGGCATCCACGGCATCCACGGCATCCACGGCATCCACGGCATGCGTTGGACA

CGAGGCAGGCGAGGCAGTTCGGCGCGGCACGATATGGCGACTCGCGCGGCCGCGCCCGCCGTCGATGCGGCGACGCGAC

GAGATGACGACGTTGCGCCGATGCGGCGTCAATGCGGCGCGCACGATATCGATATCGAAAATGCTTCGTTGTGCAGGGC

GGCGACGCACCTTAATCTGGACCCGTAG

>B7CPT6_BURPS_Bucl10a_Bps_576.seq Reverse Complement DNA Sequence Untitled Seq #36(1,360)

ATGTGTTGGATGTGTTGGATGCACGGCATGCACGGCATGCACGGCATGCACGGCATGCACGGCATGCACGGCATCCACG

GCATCCACGGCATCCACGGCATCCACGGCATCCACGGCATCCACGGCATCCACGGCATCCACGGCATCCACGGCATCCA

CGGCATGCGTTGGACACGAGGCAGGCGAGGCAGTTCGGCGCGGCACGATATGGCGACTCGCGCGGCCGCGCCCGCCGTC

GATGCGGCGACGCGACGAGATGACGACGTTGCGCCGATGCGGCGTCAATGCGGCGCGCACGATATCGATATCGAAAATG

CTTCGTTGTGCAGGGCGGCGACGCACCTTAATCTGGACCCGTAG

>Bucl10_Bps_668.seq Created: Monday, January 20, 2014 3:20 PM

atgtgttggatgcacggcatccaaggcatccacggcatccacggcatccacggcatccacgacatccacgacatccacg

acatccacgacatccacgacatccacgacatgcgttggacacgaggcaggcgaggcagttcggcgcggcacgatatggc

gactcgcgcggccgcgcccgccgtcgatgcggcgacgcgacgagatgacgacgttgcgccgatgcggcgtcaatgcggc

gcgcacgatatcgatatcgaaaatgcttcgttgtgcagggcggcgacgcaccttaatctggacccgtag

>Bucl10_Bps_BPC006_2.seq Created: Monday, January 20, 2014 3:13 PM

atgtgttggatgacggcatccacggcatccacggcatccacggcatccacggcatccacggcatccacggcatccacgg

catccacggcatccacggcatccacggcatccacggcatccacggcatgcgttggacacgaggcaggcgaggcagttcg

gcgcggcacgatatggcgactcgcgcggccgcgcccgccgtcgatgcggcgacgcgacgagatgacgacgttgcgccga

tgcggcgtcaatgcggcgcgcacgatatcgatatcgaaaatgcttcgttgtgcagggcggcgacgcaccttaatctgga

cccgtag

>A8EDY0_BURPS_Bucl10_Bps_406e.seq Reverse Complement DNA Sequence Untitled Seq #2(1,360)

ATGTGTTGGATGCACGGCATCCACGGCATCCACGGCATCCACGGCATCCACGGCATCCACGGCATCCACGGCATCCACG

GCATCCACGGCATCCACGGCATCCACGGCATCCACGGCATCCACGGCATCCACGGCATCCACGGCATCCACGGCATCCA

CGGCATGCGTTGGACACGAGGCAGGCGAGGCAGTTCGGCGCGGCACGATATGGCGACTCGCGCGGCCGCGCCCGCCGTC

GATGCGGCGACGCGACGAGATGACGACGTTGCGCCGATGCGGCGTCAATGCGGCGCGCACGATATCGATATCGAAAATG

CTTCGTTGTGCAGGGCGGCGACGCACCTTAATCTGGACCCGTAG

>Bucl10_Bm_A188.seq Reverse Complement DNA Sequence AWGR01000154.seq(1,279)

ATGTGTTGGATGCACGGCATGCACGGCATCCACGGCATCCACGGCATCCACGGCATCCACGGCATCCACGGCATCCACG

GCATGCGTTGGACACGAGGCAGGCGAGGCAGTTCGGCGCGGCACGATATGGCGACTCGCGCGGCCGCGCCCGCCGTCGA

TGCGGCGACGCGACGAGATGACGACGTTGCGCCGATGCGGCGTCAATGCGGCGCGCACGATATCGATATCGAAAATGCT

TCGTTGTGCAGGGCGGCGACGCACCTTAATCTGGACCCGTAG

>Bucl10_Bm_A193.seq Reverse Complement DNA Sequence AWGS01000115.seq(1,297)

ATGTGTTGGATGCACGGCATGCACGGCATCCACGGCATCCACGGCATCCACGGCATCCACGGCATCCACGGCATCCACG

GCATCCACGGCATCCACGGCATGCGTTGGACACGAGGCAGGCGAGGCAGTTCGGCGCGGCACGATATGGCGACTCGCGC

GGCCGCGCCCGCCGTCGATGCGGCGACGCGACGAGATGACGACGTTGCGCCGATGCGGCGTCAATGCGGCGCGCACGAT

ATCGATATCGAAAATGCTTCGTTGTGCAGGGCGGCGACGCACCTTAATCTGGACCCGTAG

>Bucl10_Bm_PRL-20.seq Reverse Complement DNA Sequence Untitled Seq #86(1,360)

ATGTGTTGGATGCACGGCATGCACGGCATGCACGGCATGCGCGGCATCCACGGCATCCACGGCATCCACGGCATCCACG

GCATCCACGGCATCCACGGCATCCACGGCATCCACGGCATCCACGGCATCCACGGCATCCACGGCATCCACGGCATCCA

CGGCATGCGTTGGACACGAGGCAGGCGAGGCAGTTCGGCGCGGCACGATATGGCGACTCGCGCGGCCGCGCCCGCCGTC

GATGCGGCGACGCGACGAGATGACGACGTTGCGCCGATGCGGCGTCAATGCGGCGCGCACGATATCGATATCGAAAATG

CTTCGTTGTGCAGGGCGGCGACGCACCTTAATCTGGACCCGTAG

>Bucl10_Bps_MSHR146.seq Reverse Complement DNA Sequence CP004042.seq(1,387)

ATGTGTTGGATGCACGGCATCCACGGCATCCACGGCATCCACGGCATCCACGGCATCCACGGCATCCACGGCATCCACG

GCATCCACGGCATCCACGGCATCCACGGCATCCACGGCATCCACGGCATCCACGGCATCCACGGCATCCACGGCATCCA

CGGCATCCACGGCATCCACGGCATCCACGGCATGCGTTGGACACGAGGCAGGCGAGGCAGTTCGGCGCGGCACGATATG

GCGACTCGCGCGGCCGCGCCCGCCGTCGATGCGGCGACGCGACGAGATGACGACGTTGCGCCGATGCGGCGTCAATGCG

GCGCGCACGATATCGATATCGAAAATGCTTCGTTGTGCAGGGCGGCGACGCACCTTAATCTGGACCCGTAG

>Bucl10_Bps_MSHR511.seq Reverse Complement DNA Sequence CP004023.seq(1,432)

ATGTGTTGGATGCACGGCATCCACGGCATCCACGGCATCCACGGCATCCACGGCATCCACGGCATCCACGGCATCCACG

GCATCCACGGCATCCACGGCATCCACGGCATCCACGGCATCCACGGCATCCACGGCATCCACGGCATCCACGGCATCCA

CGGCATCCACGGCATCCACGGCATCCACGGCATCCACGGCATCCACGGCATCCACGGCATCCACGGCATCCACGGCATG

CGTTGGACACGAGGCAGGCGAGGCAGTTCGGCGCGGCACGATATGGCGACTCGCGCGGCCGCGCCCGCCGTCGATGCGG

CGACGCGACGAGATGACGACGTTGCGCCGATGCGGCGTCAATGCGGCGCGCACGATATCGATATCGAAAATGCTTCGTT

GTGCAGGGCGGCGACGCACCTTAATCTGGACCCGTAG

>Bucl10_Bps_MSHR520.seq Reverse Complement DNA Sequence CP004368.seq(1,396)

ATGTGTTGGATGCACGGCATGCACGGCATGCACGGCATGCACGGCATGCACGGCATGCACGGCATGCACGGCATCCACG

GCATCCACGGCATCCACGGCATCCACGGCATCCACGGCATCCACGGCATCCACGGCATCCACGGCATCCACGGCATCCA

CGGCATCCACGGCATCCACGGCATCCACGGCATCCACGGCATGCGTTGGACACGAGGCAGGCGAGGCAGTTCGGCGCGG

CACGATATGGCGACTCGCGCGGCCGCGCCCGCCGTCGATGCGGCGACGCGACGAGATGACGACGTTGCGCCGATGCGGC

GTCAATGCGGCGCGCACGATATCGATATCGAAAATGCTTCGTTGTGCAGGGCGGCGACGCACCTTAATCTGGACCCGTA

G

>Bucl10_Bps_NAU20B-16.seq Reverse Complement DNA Sequence CP004003.seq(1,360)

ATGTGTTGGATGCACGGCATCCACGGCATCCACGGCATCCACGGCATCCACGGCATCCACGGCATCCACGGCATCCACG

GCATCCACGGCATCCACGGCATCCACGGCATCCACGGCATCCACGGCATCCACGGCATCCACGGCATCCACGGCATCCA

CGGCATGCGTTGGACACGAGGCAGGCGAGGCAGTTCGGCGCGGCACGATATGGCGACTCGCGCGGCCGCGCCCGCCGTC

GATGCGGCGACGCGACGAGATGACGACGTTGCGCCGATGCGGCGTCAATGCGGCGCGCACGATATCGATATCGAAAATG

CTTCGTTGTGCAGGGCGGCGACGCACCTTAATCTGGACCCGTAG

>Bucl10_Bps_NCTC_13178.seq Reverse Complement DNA Sequence CP004001.seq(1,306)

ATGTGTTGGATGCACGGCATGCACGGCATCCACGGCATCCACGGCATCCACGGCATCCACGGCATCCACGGCATCCACG

GCATCCACGGCATCCACGGCATCCACGGCATGCGTTGGACACGAGGCAGGCGAGGCAGTTCGGCGCGGCACGATATGGC

GACTCGCGCGGCCGCGCCCGCCGTCGATGCGGCGACGCGACGAGATGACGACGTTGCGCCGATGCGGCGTCAATGCGGC

GCGCACGATATCGATATCGAAAATGCTTCGTTGTGCAGGGCGGCGACGCACCTTAATCTGGACCCGTAG

>C4KXC1_BURPS_Bucl10_MSHR_346.seq Created: Monday, January 20, 2014 3:00 PM

ATGTGTTGGATGCACGGCATCCACGGCATCCACGGCATCCACGGCATCCACGGCATCCACGGCATCCACGGCATCCACG

GCATCCACGGCATCCACGGCATCCACGGCATCCACGGCATCCACGGCATCCACGGCATGCGTTGGACACGAGGCAGGCG

AGGCAGTTCGGCGCGGCACGATATGGCGACTCGCGCGGCCGCGCCCGCCGTCGATGCGGCGACGCGACGAGATGACGAC

GTTGCGCCGATGCGGCGTCAATGCGGCGCGCACGATATCGATATCGAAAATGCTTCGTTGTGCAGGGCGGCGACGCACC

TTAATCTGGACCCGTAG

>A4LQ22_BURPS_Bucl10_Bps_305.seq Created: Monday, January 20, 2014 2:24 PM

atgtgttggatgcacggcatgcacggcatgcacggcatgcacggcatgcacggcatccacggcatccacggcatccacg

gcatccacggcatccacggcatgcgttggacacgaggcaggcgaggcagttcggcgcggcacgatatggcgactcgcgc

ggccgcgcccgccgtcgatgcggcgacgcgacgagatgacgacgttgcgccgatgcggcgtcaatgcggcgcgcacgat

atcgatatcgaaaatgcttcgttgtgcagggcggcgacgcaccttaatctggacccgtag

>Bucl10_Bm_strain_6.seq

ATGTGTTGGATGCACGGCATGCACGGCATGCACGGCATGCGCGGCATCCACGGCATCCACGGCATCCACGGCATCCACG

GCATCCACGGCATCCACGGCATGCGTTGGACACGAGGCAGGCGAGGCAGTTCGGCGCGGCACGATATGGCGACTCGCGC

GGCCGCGCCCGCCGTCGATGCGGCGACGCGACGAGATGACGACGTTGCGCCGATGCGGCGTCAATGCGGCGCGCACGAT

ATCGATATCGAAAATGCTTCGTTGTGCAGGGCGGCGACGCACCTTAATCTGGACCCGTAG

>Bucl10_Bm_strain_11.seq

ATGTGTTGGATGCACGGCATGCACGGCATGCACGGCATCCACGGCATCCACGGCATCCACGGCATCCACGGCATCCACG

GCATCCACGGCATCCACGGCATCCACGGCATCCACGGCATCCACGGCATCCACGGCATCCACGGCATCCACGGCATCCA

CGGCATCCACGGCATCCACGGCATCCACGGCATCCACGGCATCCACGGCATCCACGGCATCCACGGCATCCACGGCATC

CACGGCATCCACGGCATCCACGGCATCCACGGCATGCGTTGGACACGAGGCAGGCGAGGCAGTTCGGCGCGGCACGATA

TGGCGACTCGCGCGGCCGCGCCCGCCGTCGATGCGGCGACGCGACGAGATGACGACGTTGCGCCGATGCGGCGTCAATG

CGGCGCGCACGATATCGATATCGAAAATGCTTCGTTGTGCAGGGCGGCGACGCACCTTAATCTGGACCCGTAG

>A8EP72_BURPS_Bucl13_Bps_406e.seq Created: Sunday, August 04, 2013 8:47 PM

ATGTGTCGACGGCGTCGCGCAGTCGCGCGCGCTCGCATTGCGTCGCGCGGCGTCGATAGTGTCGATCATGTCGGAAGTG

GCGGAAGTGGCGGAAGTGGCGGAAGTGGCGGAAGCATCAGAAGCATCAGAAGCATCAGAAGCATCAGAAGCATCAGGGG

CATCAGGGGCATCAGGGGCATCAGGGGCATCAGGGGCATCAGAGGTATCAGAGGTATCAGAGGTATCAGAGGTATCAGA

GGCGTCAGAGGCGTCAGAGGCGTCAGCGGCATTAGAAGCATCGGAGGCACCGAAGCGATGACCGACCGACGCACATTCG

TCGGCGGCGCGGCGGCCTTCGCGGCCGCATGGGCCGCCGCGCCGTTCGGCGCGCGCGCCGGCATCGCGGTCGATCTCGA

CCCGCGTCAGGCGGGCCGCGTGCGCGCCGAACGCGATCCGGCCGCCGTCGACGCCGCGCGCCGCTACCGCTGGGTCGAC

GATCGCGCGTTCACCGTCGCGATCGCGCCGCATGCGCCACCCGTCGCGACGTTCGCGACCGACGCGCGCACCGTCGTCG

GCGCGGACCCGGACTACGCGCAACTCGTCGCCGACGCGCTCGGCCGGCGGCTCGCGCTCGTGCCCGTCGCCTGGGCCGA

CTGGCCGCTCGGGCTGAGCGCCGGCAAGTACGACGCGGTGATCTCGAACGTCGGCGTCACCGAGCAGCGCAAGCGGAAG

TTCGATTTCACGACGTACCGGCTCGGCCTGCACGGCTTCTATGTGCGGGCCGCGAGCCCGATCGCGCGGATCGCCGCGC

CGCGCGACGTGGCGGGCCTGCGGATCATCACCGCGTCGGGAACGAGCCAGGAGCGCATCCTGCTCGAATGGGACCGGCG

CAACGTCGCGCAGCGGCTCAAGCCGGTGGACGTGCTGTACTTCGACGACGACGCCGCATCGCGCGTCGCGCTGCTCTCG

GGGCGCGCCGACGCGGAGCTGAATCCGAACGCGACACTCGCGTACCAGGCGGCGCGCGACGGCAGGATTCGTTGCGTCG

GCAACGTGAACGCGGGCTGGCCGCTCAAGGCCGACGTCGCGATCGCGACGCGCAAGGGCAGCGGCCTCGCCGACGCGCT

CACGCTCGCGACCAACGGCCTGATCCGCAACGGCAAGTACCGGCAGGCGCTCGCGCGATGGGGGCTGCTGTCGGAGGCG

CTCGACCGGTCGGAGACGAATCCGGCGGGGCTGCCGTCGTTCTGA

>B2HC24_BURPS_Bucl13_Bps_1655.seq Created: Sunday, August 04, 2013 8:41 PM

ATGTGTCGACGGCGTCGCGCAGTCGCGCGCGCTCGCATTGCGTCGCGCGGCGTCGATAGTGTCGATCATGTCGGAAGTG

GCGGAAGTGGCGGAAGCATCAGGGGCATCAGGGGCATCAGGGGCATCAGGGGCATCAGAGGCATCAGAGGCATCAGAGG

CATCAGAGGTATCAGAGGTATCAGAGGTATCAGAGGTATCAGAGGTATCAGAGGTATCAGAGGCGTCAGAGGCGTCAGA

GGCATTAGAAGCATCAGCGGCATTAGAAGCATCGGAGGCACCGAAGCGATGACCGACCGACGCACATTCGTCGGCGGCG

CGGCGGCCTTCGCGGCCGCATGGGCCGCCGCGCCGTTCGGCGCGCGCGCCGGCATCGCGGTCGATCTCGACCCGCGTCA

GGCGGGCCGCGTGCGCGCCGAACGCGATCCGGCCGCCGTCGACGCCGCGCGCCGCTACCGCTGGGTCGACGATCGCGCG

TTCACCGTCGCGATCGCGCCGCATGCGCCGCCCGTCGCGACGTTCGCGACCGACGCGCGCACCGTCGTCGGCGCGGACC

CCGACTACGCGCAACTCGTCGCCGACGCGCTCGGCCGGCGGCTCGCGCTCGTGCCCGTCGCCTGGGCCGACTGGCCGCT

CGGGCTGAGCGCCGGCAAGTACGACGCGGTGATCTCGAACGTCGGCGTCACCGAGCAGCGCAAGCGGAAGTTCGATTTC

ACGACGTACCGGCTCGGCCTGCACGGCTTCTATGTGCGGGCCGCGAGCCCGATCGCGCGGATCGCCGCGCCGCGCGACG

TGGCGGGCCTGCGGATCATCACCGCGTCGGGAACGAGCCAGGAGCGCATCCTGCTCGAATGGGACCGGCGCAACGTCGC

GCAGCGGCTCAAGCCGGTGGACGTGCTGTACTTCGACGACGACGCCGCATCGCGCGTCGCGCTGCTCTCGGGGCGCGCC

GACGCGGAGCTGAATCCGAACGCGACACTCGCGTACCAGGCGGCGCGCGACGGCAGGATTCGTTGCGTCGGCAACGTGA

ACGCGGGCTGGCCGCTCAAGGCCGACGTCGCGATCGCGACGCGCAAGGGCAGCGGCCTCGCCGACGCGCTCACGCTCGC

GACCAACGGCCTGATCCGCAACGGCAAGTACCGGCAGGCGCTCGCGCGATGGGGGCTGCTGTCGGAGGCGCTCGACCGG

TCGGAGACGAATCCGGCGGGGCTGCCGTCGTTCTGA

>Bucl13_Bm_NCTC_10229.seq Created: Saturday, November 23, 2013 11:15 AM

ATGTGTCGACGGCGTCGCGCAGTCGCGCGCGCTCGCATTGCGTCGCGCGGCGTCGATAGTGTCGATCATGTCGGAAGTG

GCGGAAGTGGCGGAAGTGGCGGAAGCATCAGAAGCATCAGAAGCATCAGGGGCATCAGGGGCATCAGAGGTATCAGAGG

TATCAGAGGTATCAGAGGTATCAGAGGTATCAGAGGTATCAGAGGCGTCAGAGGCGTCAGAGGCGTCAGAGGCATTAGA

AGCATCGGAGGCACCGAAGCGATGACCGACCGACGCACATTCGTCGGCGGCGCGGCGGCCTTCGCGGCCGCATGGGCCG

CCGCGCCGTTCGGCGCGCGCGCCGGCATCGCGGTCGATCTCGACCCGCGTCAGGCGGGCCGCGTGCGCGCCGAACGCGA

TCCGGCCGCCGTCGACGCCGCGCGCCGCTACCGCTGGGTCGACGATCGCGCGTTCACCGTCGCGATCGCGCCGCATGCG

CCACCCGTCGCGACGTTCGCGACCGACGCGCGCACCGTCGTCGGCGCGGACCCGGACTACGCGCAACTCGTCGCCGACG

CGCTCGGCCGGCGGCTCGCGCTCGTGCCCGTCGCCTGGGCCGACTGGCCGCTCGGGCTGAGCGCCGGCAAGTACGACGC

GGTGATCTCGAACGTCGGCGTCACCGAGCAGCGCAAGCGGAAGTTCGATTTCACGACGTACCGGCTCGGCCTGCACGGC

TTCTATGTGCGGGCCGCGAGCCCGATCGCGCGGATCGCCGCGCCGCGCGACGTGGCGGGCCTGCGGATCATCACCGCGT

CGGGAACGAGCCAGGAGCGCATCCTGCTCGAATGGGACCGGCGCAACGTCGCGCAGCGGCTCAAGCCGGTGGACGTGCT

GTACTTCGACGACGACGCCGCATCGCGCGTCGCGCTGCTCTCGGGGCGCGCCGACGCGGAGCTGAATCCGAACGCGACA

CTCGCGTACCAGGCGGCGCGCGACGGCAGGATTCGTTGCGTCGGCAACGTGAACGCGGGCTGGCCGCTCAAGGCCGACG

TCGCGATCGCGACGCGCAAGGGCAGCGGCCTCGCCGACGCGCTCACGCTCGCGACCAACGGCCTGATCCGCAACGGCAA

GTACCGGCAGGCGCTCGCGCGATGGGGGCTGCTGTCGGAGGCGCTCGACCGGTCGGAGACGAATCCGGCGGGGCTGCCG

TCGTTCTGA

>Bucl13_Bm_NCTC_10247.seq Created: Saturday, November 23, 2013 11:19 AM

ATGTGTCGACGGCGTCGCGCAGTCGCGCGCGCTCGCATTGCGTCGCGCGGCGTCGATAGTGTCGATCATGTCGGAAGTG

GCGGAAGTGGCGGAAGTGGCGGAAGCATCAGAAGCATCAGAAGCATCAGGGGCATCAGGGGCATCAGAGGTATCAGAGG

TATCAGAGGTATCAGAGGTATCAGAGGTATCAGAGGTATCAGAGGCGTCAGAGGCGTCAGAGGCATTAGAAGCATCGGA

GGCACCGAAGCGATGACCGACCGACGCACATTCGTCGGCGGCGCGGCGGCCTTCGCGGCCGCATGGGCCGCCGCGCCGT

TCGGCGCGCGCGCCGGCATCGCGGTCGATCTCGACCCGCGTCAGGCGGGCCGCGTGCGCGCCGAACGCGATCCGGCCGC

CGTCGACGCCGCGCGCCGCTACCGCTGGGTCGACGATCGCGCGTTCACCGTCGCGATCGCGCCGCATGCGCCACCCGTC

GCGACGTTCGCGACCGACGCGCGCACCGTCGTCGGCGCGGACCCGGACTACGCGCAACTCGTCGCCGACGCGCTCGGCC

GGCGGCTCGCGCTCGTGCCCGTCGCCTGGGCCGACTGGCCGCTCGGGCTGAGCGCCGGCAAGTACGACGCGGTGATCTC

GAACGTCGGCGTCACCGAGCAGCGCAAGCGGAAGTTCGATTTCACGACGTACCGGCTCGGCCTGCACGGCTTCTATGTG

CGGGCCGCGAGCCCGATCGCGCGGATCGCCGCGCCGCGCGACGTGGCGGGCCTGCGGATCATCACCGCGTCGGGAACGA

GCCAGGAGCGCATCCTGCTCGAATGGGACCGGCGCAACGTCGCGCAGCGGCTCAAGCCGGTGGACGTGCTGTACTTCGA

CGACGACGCCGCATCGCGCGTCGCGCTGCTCTCGGGGCGCGCCGACGCGGAGCTGAATCCGAACGCGACACTCGCGTAC

CAGGCGGCGCGCGACGGCAGGATTCGTTGCGTCGGCAACGTGAACGCGGGCTGGCCGCTCAAGGCCGACGTCGCGATCG

CGACGCGCAAGGGCAGCGGCCTCGCCGACGCGCTCACGCTCGCGACCAACGGCCTGATCCGCAACGGCAAGTACCGGCA

GGCGCTCGCGCGATGGGGGCTGCTGTCGGAGGCGCTCGACCGGTCGGAGACGAATCCGGCGGGGCTGCCGTCGTTCTGA

>Bucl13_Bm_SAVP1.seq Created: Saturday, November 23, 2013 11:25 AM

ATGTGTCGACGGCGTCGCGCAGTCGCGCGCGCTCGCATTGCGTCGCGCGGCGTCGATAGTGTCGATCATGTCGGAAGTG

GCGGAAGTGGCGGAAGTGGCGGAAGCATCAGAAGCATCAGAAGCATCAGGGGCATCAGGGGCATCAGAGGTATCAGAGG

TATCAGAGGTATCAGAGGTATCAGAGGCGTCAGAGGCGTCAGAGGCGTCAGAGGCATTAGAAGCATCGGAGGCACCGAA

GCGATGACCGACCGACGCACATTCGTCGGCGGCGCGGCGGCCTTCGCGGCCGCATGGGCCGCCGCGCCGTTCGGCGCGC

GCGCCGGCATCGCGGTCGATCTCGACCCGCGTCAGGCGGGCCGCGTGCGCGCCGAACGCGATCCGGCCGCCGTCGACGC

CGCGCGCCGCTACCGCTGGGTCGACGATCGCGCGTTCACCGTCGCGATCGCGCCGCATGCGCCACCCGTCGCGACGTTC

GCGACCGACGCGCGCACCGTCGTCGGCGCGGACCCGGACTACGCGCAACTCGTCGCCGACGCGCTCGGCCGGCGGCTCG

CGCTCGTGCCCGTCGCCTGGGCCGACTGGCCGCTCGGGCTGAGCGCCGGCAAGTACGACGCGGTGATCTCGAACGTCGG

CGTCACCGAGCAGCGCAAGCGGAAGTTCGATTTCACGACGTACCGGCTCGGCCTGCACGGCTTCTATGTGCGGGCCGCG

AGCCCGATCGCGCGGATCGCCGCGCCGCGCGACGTGGCGGGCCTGCGGATCATCACCGCGTCGGGAACGAGCCAGGAGC

GCATCCTGCTCGAATGGGACCGGCGCAACGTCGCGCAGCGGCTCAAGCCGGTGGACGTGCTGTACTTCGACGACGACGC

CGCATCGCGCGTCGCGCTGCTCTCGGGGCGCGCCGACGCGGAGCTGAATCCGAACGCGACACTCGCGTACCAGGCGGCG

CGCGACGGCAGGATTCGTTGCGTCGGCAACGTGAACGCGGGCTGGCCGCTCAAGGCCGACGTCGCGATCGCGACGCGCA

AGGGCAGCGGCCTCGCCGACGCGCTCACGCTCGCGACCAACGGCCTGATCCGCAACGGCAAGTACCGGCAGGCGCTCGC

GCGATGGGGGCTGCTGTCGGAGGCGCTCGACCGGTCGGAGACGAATCCGGCGGGGCTGCCGTCGTTCTGA

>Bucl13_Bps_1026b.seq Reverse Complement DNA Sequence Untitled Seq #13(1,1194)

ATGTGTCGACGGCGTCGCGCAGTCGCGCGCGCTCGCATTGCGTCGCGCGGCGTCGATAGTGTCGATCATGTCGGAAGTG

GCGGAAGTGGCGGAAGCATCAGAAGCATCAGAAGCATCAGGGGCATCAGGGGCATCAGGGGCATCAGAGGCATCAGAGG

TATCAGAGGTATCAGAGGCGTCAGAGGCGTCAGAGGCATTAGAAGCGTCAGCGGCATTAGAAACGTCAGCGGCATTAGA

AGCATCGGAGGCACCGAAGCGATGACCGACCGACGCACATTCGTCGGCGGCGCGGCGGCCTTCGCGGCCGCATGGGCCG

CCGCGCCGTTCGGCGCGCGCGCCGGCATCGCGGTCGATCTCGACCCGCGTCAGGCGGGCCGCGTGCGCGCCGAACGCGA

TCCGGCCGTCGTCGACGCCGCGCGCCGCTACCGCTGGGTCGACGATCGCGCGTTCACCGTCGCGATCGCGCCGCATGCG

CCACCCGTCGCGACGTTCGCGACCGACGCGCGCACCGTCGTCGGCGCGGACCCGGACTACGCGCAACTCGTCGCCGACG

CGCTCGGCCGGCTGCTCGCGCTCGTGCCCGTCGCCTGGGCCGACTGGCCGCTCGGGCTGAGCGCCGGCAAGTACGACGC

GGTGATCTCGAACGTCGGCGTCACCGAGCAGCGCAAGCGGAAGTTCGATTTCACGACGTACCGGCTCGGCCTGCACGGC

TTCTATGTGCGGGCCGCGAGCCCGATCGCGCGGATCGCCGCGCCGCGCGACGTGGCGGGCCTGCGGATCATCACCGCGT

CGGGAACGAGCCAGGAGCGCATCCTGCTCGAATGGGACCGGCGCAACGTCGCGCAGCGGCTCAAGCCGGTGGACGTGCT

GTACTTCGACGACGACGCCGCATCGCGCGTCGCGCTGCTCTCGGGGCGCGCCGACGCGGAGCTGAATCCGAACGCGACA

CTCGCGTACCAGGCGGCGCGCGACGGCAGGATTCGTTGCGTCGGCAACGTGAACGCGGGCTGGCCGCTCAAGGCCGACG

TCGCGATCGCGACGCGCAAGGGCAGCGGCCTCGCCGACGCGCTCACGCTCGCGACCAACGGCCTGATCCGCAACGGCAA

GTACCGGCAGGCGCTCGCGCGATGGGGGCTGCTGTCGGAGGCGCTCGACCGGTCGGAGACGAATCCGGCGGGGCTGCCG

TCGTTCTGA

>Bucl13_Bps_1710b.seq Reverse Complement DNA Sequence Untitled Seq #7(1,1221)

ATGTGTCGACGGCGTCGCGCAGTCGCGCGCGCTCGCATTGCGTCGCGCGGCGTCGATAGTGTCGATCATGTCGGAAGTG

GCGGAAGCATCAGAAGCATCAGAAGCATCAGAAGCATCAGAAGCATCAGAAGCATCAGAAGCATCAGAAGCATCAGAAG

CATCAGAAGCATCAGAGGCATCAGAGGCGTCAGAGGCGTCAGAGGCGTCAGAGGCGTCAGAGGCATTAGAAGCGTCAGC

GGCATTAGAAACGTCAGCGGCATTAGAAGCATCGGAGGCACCGAAGCGATGACCGACCGACGCACATTCGTCGGCGGCG

CGGCGGCCTTCGCGGCCGCATGGGCCGCCGCGCCGTTCGGCGCGCGCGCCGGCATCGCGGTCGATCTCGACCCGCGTCA

GGCGGGCCGCGTGCGCGCCGAACGCGATCCGGCCGCCGTCGACGCCGCGCGCCGCTACCGCTGGGTCGACGATCGCGCG

TTCACCGTCGCGATCGCGCCGCATGCGCCACCCGTCGCGACGTTCGCGACCGACGCGCGCACCGTCGTCGGCGCGGACC

CGGACTACGCGCAACTCGTCGCCGACGCGCTCGGCCGGCTGCTCGCGCTCGTGCCCGTCGCCTGGGCCGACTGGCCGCT

CGGGCTGAGCGCCGGCAAGTACGACGCGGTGATCTCGAACGTCGGCGTCACCGAGCAGCGCAAGCGGAAGTTCGATTTC

ACGACGTACCGGCTCGGCCTGCACGGCTTCTATGTGCGGGCCGCGAGCCCGATCGCGCGGATCGCCGCGCCGCGCGACG

TGGCGGGCCTGCGGATCATCACCGCGTCGGGAACGAGCCAGGAGCGCATCCTGCTCGAATGGGACCGGCGCAACGTCGC

GCAGCGGCTCAAGCCGGTGGACGTGCTGTACTTCGACGACGACGCCGCATCGCGCGTCGCGCTGCTCTCGGGGCGCGCC

GACGCGGAGCTGAATCCGAACGCGACACTCGCGTACCAGGCGGCGCGCGACGGCAGGATTCGTTGCGTCGGCAACGTGA

ACGCGGGCTGGCCGCTCAAGGCCGACGTCGCGATCGCGACGCGCAAGGGCAGCGGCCTCGCCGACGCGCTCACGCTCGC

GACCAACGGCCTGATCCGCAACGGCAAGTACCGGCAGGCGCTCGCGCGATGGGGGCTGCTGTCGGAGGCGCTCGACCGG

TCGGAGACGAATCCGGCGGGGCTGCCGTCGTTCTGA

>Bucl13_Bps_668.seq Reverse Complement DNA Sequence Untitled Seq #1(1,1230)

ATGTGTCGATGGCGTCGCGCAGTCGCGCGCGCTCGCATTGCGTCGCGCGGCGTCGATAGTGTCGATCACGTCGGAAGTG

GCGGAAGCATCAGAAGCATCAGAAGCATCAGAGGTATCAGGGGTATCAGAGGTATCAGAGGTATCAGAGGTATCAGAGG

TATCAGAGGTATCAGAGGCATCAGAGGCATCAGAGGCATCAGAGGCATCAGAGGCATCAGAGGCGTCAGAGGCGTCAGA

GGCGTCAGAGGCATTAGAAGCGTCAGCGGCATTAGAAGCATCGGAGGCACCGACGCGATGACCGACCGACGCACATTCG

TCGGCGGCGCGGCGGCCTTCGCGGCCGCATGGGCCGCCGCGCCGTTCGGCGCGCGCGCCGGCATCGCGGTCGATCTCGA

CCCGCGTCAGGCGGGCCGCGTGCGCGCCGAACGCGATCCGGCCGCCGTCGACGCCGCGCGCCGCTACCGCTGGGTCGAC

GATCGCGCGTTCACCGTCGCGATCGCGCCGCATGCGCCACCCGTCGCGACGTTCGCGACCGACGCGCGCACCGTCGTCG

GCGCGGACCCCGACTACGCGCAACTCGTCGCCGACGCGCTCGGCCGGCGGCTCGCGCTCGTGCCCGTCGCCTGGGCCGA

CTGGCCGCTCGGGCTGAGCGCCGGCAAGTACGACGCGGTGATCTCGAACGTCGGCGTCACCGAGCAGCGCAAGCGGAAG

TTCGATTTCACGACGTACCGGCTCGGCCTGCACGGCTTCTATGTGCGGGCCGCGAGCCCGATCGCGCGGATCGCCGCGC

CGCGCGACGTGGCGGGCCTGCGGATCATCACCGCGTCGGGAACGAGCCAGGAGCGCATCCTGCTCGAATGGGACCGGCG

CAACGTCGCGCAGCGGCTCAAGCCGGTGGACGTGCTGTACTTCGACGACGACGCCGCATCGCGCGTCGCGCTGCTCTCG

GGGCGCGCCGACGCGGAGCTGAATCCGAACGCGACACTCGCGTACCAGGCGGCGCGCGACGGCAGGATTCGTTGCGTCG

GCAACGTGAACGCGGGCTGGCCGCTCAAGGCCGACGTCGCGATCGCGACGCGCAAGGGCAGCGGCCTCGCCGACGCGCT

CACGCTCGCGACCAACGGCCTGATCCGCAACGGCAAGTACCGGCAGGCGCTCGCGCGATGGGGGCTGCTGTCGGAGGCG

CTCGACCGGTCGGAGACGAATCCGGCGGGGCTGCCGTCGTTCTGA

>Bucl13_Bps_BPC006.seq Reverse Complement DNA Sequence Untitled Seq #4(1,1221)

ATGTGTCGACGGCGTCGCGCAGTCGCGCGCGCTCGCATTGCGTCGCGCGGCGTCGATAGTGTCGATCATGTCGGAAGTG

GCGGAAGCATCAGAAGCATCAGAAGCATCAGAAGCATCAGAAGCATCAGAAGCATCAGAAGCATCAGAAGCATCAGAAG

CATCAGAAGCATCAGAGGCATCAGAGGCGTCAGAGGCGTCAGAGGCGTCAGAGGCGTCAGAGGCATTAGAAGCGTCAGC

GGCATTAGAAACGTCAGCGGCATTAGAAGCATCGGAGGCACCGAAGCGATGACCGACCGACGCACATTCGTCGGCGGCG

CGGCGGCCTTCGCGGCCGCATGGGCCGCCGCGCCGTTCGGCGCGCGCGCCGGCATCGCGGTCGATCTCGACCCGCGTCA

GGCGGGCCGCGTGCGCGCCGAACGCGATCCGGCCGCCGTCGACGCCGCGCGCCGCTACCGCTGGGTCGACGATCGCGCG

TTCACCGTCGCGATCGCGCCGCATGCGCCACCCGTCGCGACGTTCGCGACCGACGCGCGCACCGTCGTCGGCGCGGACC

CGGACTACGCGCAACTCGTCGCCGACGCGCTCGGCCGGCGGCTCGCGCTCGTGCCCGTCGCCTGGGCCGACTGGCCGCT

CGGGCTGAGCGCCGGCAAGTACGACGCGGTGATCTCGAACGTCGGCGTCACCGAGCAGCGCAAGCGGAAGTTCGATTTC

ACGACGTACCGGCTCGGCCTGCACGGCTTCTATGTGCGGGCCGCGAGCCCGATCGCGCGGATCGTCGCGCCGCGCGACG

TGGCGGGCCTGCGGATCATCACCGCGTCGGGAACGAGCCAGGAGCGCATCCTGCTCGAATGGGACCGGCGCAACGTCGC

GCAGCGGCTCAAGCCGGTGGACGTGCTGTACTTCGACGACGACGCCGCATCGCGCGTCGCGCTGCTCTCGGGGCGCGCC

GACGCGGAGCTGAATCCGAACGCGACACTCGCGTACCAGGCGGCGCGCGACGGCAGGATTCGTTGCGTCGGCAACGTGA

ACGCGGGCTGGCCGCTCAAGGCCGACGTCGCGATCGCGACGCGCAAGGGCAGCGGCCTCGCCGACGCGCTCACGCTCGC

GACCAACGGCCTGATCCGCAACGGCAAGTACCGGCAGGCGCTCGCGCGATGGGGGCTGCTGTCGGAGGCGCTCGACCGG

TCGGAGACGAATCCGGCGGGGCTGCCGTCGTTCTGA

>Bucl13_Bps_K96243.seq Reverse Complement DNA Sequence Untitled Seq #10(1,1239)

ATGTGTCGACGGCGTCGCGCAGTCGCGCGCGCTCGCATTGCGTCGCGCGGCGTCGATAGTGTCGATCATGTCGGAAGTG

GCGGAAGTGGCGGAAGTGGCGGAAGCATCAGAAGCATCAGAAGCATCAGAAGCATCAGAAGCATCAGAAGCATCAGAAG

CATCAGAAGCATCAGAAGCATCAGAAGCATCAGGGGCATCAGGGGCATCAGGGGCATCAGAGGTATCAGAGGTATCAGA

GGCGTCAGAGGCGTCAGAGGCGTCAGAGGCGTCAGCGGCATTAGAAGCATCGGAGGCACCGAAGCGATGACCGACCGAC

GCACATTCGTCGGCGGCGCGGCGGCCTTCGCAGCCGCATGGGCCGCCGCGCCGTTCGGCGCGCGCGCCGGCATCGCGGT

CGATCTCGACCCGCGTCAGGCGGGCCGCGTGCGCGCCGAACGCGATCCGGCCGTCGTCGACGCCACGCGCCGCTACCGC

TGGGTCGACGATCGCGCGTTCACCGTCGCGATCGCGCCGCATGCGCCACCCGTCGCGACGTTCGCGACCGACGCGCGCA

CCGTCGTCGGCGCGGACCCGGACTACGCGCAACTCGTCGCCGACGCGCTCGGCCGGCGGCTCGCGCTCGTGCCCGTCGC

CTGGGCCGACTGGCCGCTCGGGCTGAGCGCCGGCAAGTACGACGCGGTGATCTCGAACGTCGGCGTCACCGAGCAGCGC

AAGCGGAAGTTCGATTTCACGACGTACCGGCTCGGCCTGCACGGCTTCTATGTGCGGGCCGCGAGCCCGATCGCGCGGA

TCGCCGCGCCGCGCGACGTGGCGGGCCTGCGGATCATCACCGCGTCGGGAACGAGCCAGGAGCGCATCCTGCTCGAATG

GGACCGGCGCAACGTCGCGCAGCGGCTCAAGCCGGTGGACGTGCTGTACTTCGACGACGACGCCGCATCGCGCGTCGCG

CTGCTCTCGGGGCGCGCCGACGCGGAGCTGAATCCGAACGCGACACTCGCGTACCAGGCGGCGCGCGACGGCAGGATTC

GTTGCGTCGGCAACGTGAACGCGGGCTGGCCGCTCAAGGCCGACGTCGCGATCGCGACGCGCAAGGGCAGCGGCCTCGC

CGACGCGCTCACGCTCGCGACCAACGGCCTGATCCGCAACGGCAAGTACCGGCAGGCGCTCGCGCGATGGGGGCTGCTG

TCGGAGGCGCTCGACCGGTCGGAGACGAATCCGGCGGGGCTGCCGTCGTTCTGA

>Bucl13_Bps_1106a.seq Created: Tuesday, December 31, 2013 9:55 AM

atgtgtcgacggcgtcgcgcagtcgcgcgcgctcgcattgcgtcgcgcggcgtcgatagtgtcgatcatgtcggaagtg

gcggaagcatcagaagcatcagaagcatcagaagcatcagaagcatcagaagcatcagaggcgtcagaggcgtcagagg

cgtcagaggcattagaagcgtcagcggcattagaagcgtcagcggcattagaaacgtcagcggcattagaagcatcgga

ggcaccgaagcgatgaccgaccgacgcacattcgtcggcggcgcggcggccttcgcggccgcatgggccgccgcgccgt

tcggcgcgcgcgccggcatcgcggtcgatctcgacccgcgtcaggcgggccgcgtgcgcgccgaacgcgatccggccgc

cgtcgacgccgcgcgccgctaccgctgggtcgacgatcgcgcgttcaccgtcgcgatcgcgccgcatgcgccacccgtc

gcgacgttcgcgaccgacgcgcgcaccgtcgtcggcgcggacccggactacgcgcaactcgtcgccgacgcgctcggcc

ggcggctcgcgctcgtgcccgtcgcctgggccgactggccgctcgggctgagcgccggcaagtacgacgcggtgatctc

gaacgtcggcgtcaccgagcagcgcaagcggaagttcgatttcacgacgtaccggctcggcctgcacggcttctatgtg

cgggccgcgagcccgatcgcgcggatcgtcgcgccgcgcgacgtggcgggcctgcggatcatcaccgcgtcgggaacga

gccaggagcgcatcctgctcgaatgggaccggcgcaacgtcgcgcagcggctcaagccggtggacgtgctgtacttcga

cgacgacgccgcatcgcgcgtcgcgctgctctcggggcgcgccgacgcggagctgaatccgaacgcgacactcgcgtac

caggcggcgcgcgacggcaggattcgttgcgtcggcaacgtgaacgcgggctggccgctcaaggccgacgtcgcgatcg

cgacgcgcaagggcagcggcctcgccgacgcgctcacgctcgcgaccaacggcctgatccgcaacggcaagtaccggca

ggcgctcgcgcgatgggggctgctgtcggaggcgctcgaccggtcggagacgaatccggcggggctgccgtcgttctga

>Bucl13_Bps_MSHR_305.seq Created: Thursday, January 02, 2014 11:19 AM

atgtgtcgacggcgtcgcgcagtcgcgcgcgctcgcattgcgtcgcgcggcgtcgatagtgtcgatcatgtcggaagtg

gcggaagcatcagaagcatcagaagcatcagaagcatcagaagcatcagaagcatcagaagcatcagaagcatcagaag

catcagaagcatcagaggtatcagaggcatcagaggcattagaagcgtcagcggcattagaagcatcggaggcaccgaa

gcgatgaccgaccgacgcacattcgtcggcggcgcggcggccttcgcggccgcatgggccgccgcgccgttcggcgcgc

gcgccggcatcgcggtcgatctcgacccgcgtcaggcgggccgcgtgcgcgccgaacgcgatccggccgccgtcgacgc

cgcgcgccgctaccgctgggtcgacgatcgcgcgttcaccgtcgcgatcgcgccgcatgcgccacccgtcgcgacgttc

gcgaccgacgcgcgcaccgtcgtcggcgcggacccggactacgcgcaactcgtcgccgacgcgctcggccggcggctcg

cgctcgtgcccgtcgcctgggccgactggccgctcgggctgagcgccggcaagtacgacgcggtgatctcgaacgtcgg

cgtcaccgagcagcgcaagcggaagttcgatttcacgacgtaccggctcggcctgcacggcttctatgtgcgggccgcg

agcccgatcgcgcggatcgccgcgccgcgcgacgtggcgggcctgcggatcatcaccgcgtcgggaacgagccaggagc

gcatcctgctcgaatgggaccggcgcaacgtcgcgcagcggctcaagccggtggacgtgctgtacttcgacgacgacgc

cgcatcgcgcgtcgcgctgctctcggggcgcgccgacgcggagctgaatccgaacgcgacactcgcgtaccaagcggcg

cgcgacggcaggattcgttgcgtcggcaacgtgaacgcgggctggccgctcaaggccgacgtcgcgatcgcgacgcgca

agggcagcggcctcgccgacgcgctcacgctcgcgaccaacggcctgatccgcaacggcaagtaccggcaggcgctcgc

gcgatgggggctgctgtcggaggcgctcgaccggtcggagacgaatccggcggggctgccgtcgttctga

>Bucl13_Bps_NCTC_13179.seq Created: Thursday, January 02, 2014 3:15 PM

atgtgtcgacggcgtcgcgcagtcgcgcgcgctcgcattgcgtcgcgcggcgtcgatagtgtcgatcatgtcggaagtg

gcggaagcatcaggggcatcaggggcatcaggggcatcaggggcatcaggggcatcaggggcatcagaggcatcagagg

catcagaggcatcagaggtatcagaggtatcagaggtatcagagatatcagaggtatcagaggtatcagaggcgtcaga

ggcgtcagaggcgtcagaggcgtcagaggcgtcagaggcgtcagaggcgtcagaggcgtcagaggcattagaagcgtca

gcggcattagaaacgtcagcggcattagaagcatcggaggcaccgaagcgatgaccgaccgacgcacattcgtcggcgg

cgcggcggccttcgcggccgcatgggccgccgcgccgttcggcgcgcgcgccggcatcgcggtcgatctcgacccgcgt

caggcgggccgcgtgcgcgccgaacgcgatccggccgccgtcgacgccgcgcgccgctaccgctgggtcgacgatcgcg

cgttcaccgtcgcgatcgcgccgcatgcgccacccgtcgcgacgttcgcgaccgacgcgcgcaccgtcgtcggcgcgga

cccggactacgcgcaactcgtcgccgacgcgctcggccggcggctcgcgctcgtgcccgtcgcctgggccgactggccg

ctcgggctgagcgccggcaagtacgacgcggtgatctcgaacgtcggcgtcaccgagcagcgcaagcggaagttcgatt

tcacgacgtaccggctcggcctgcacggcttctatgtgcgggccgcgagcccgatcgcgcggatcgccgcgccgcgcga

cgtggcgggcctgcggatcatcaccgcgtcgggaacgagccaggagcgcatcctgctcgaatgggaccggcgcaacgtc

gcgcagcggctcaagccggtggacgtgctgtacttcgacgacgacgccgcatcgcgcgtcgcgctgctctcggggcgcg

ccgacgcggagctgaatccgaacgcgacactcgcgtaccaggcggcgcgcgacggcaggattcgttgcgtcggcaacgt

gaacgcgggctggccgctcaaggccgacgtcgcgatcgcgacgcgcaagggcagcggcctcgccgacgcgctcacgctc

gcgaccaacggcctgatccgcaacggcaagtaccggcaggcgctcgcgcgatgggggctgctgtcggaggcgctcgacc

ggtcggagacgaatccggcggggctgccgtcgttctga

>Bucl13_Bm_ATCC_23344.seq Created: Friday, January 03, 2014 1:12 PM

atgtgtcgacggcgtcgcgcagtcgcgcgcgctcgcattgcgtcgcgcggcgtcgatagtgtcgatcatgtcggaagtg

gcggaagtggcggaagcatcagaagcatcagaagcatcaggggcatcaggggcatcagaggtatcagaggtatcagagg

tatcagaggcgtcagaggcgtcagaggcgtcagaggcattagaagcatcggaggcaccgaagcgatgaccgaccgacgc

acattcgtcggcggcgcggcggccttcgcggccgcatgggccgccgcgccgttcggcgcgcgcgccggcatcgcggtcg

atctcgacccgcgtcaggcgggccgcgtgcgcgccgaacgcgatccggccgccgtcgacgccgcgcgccgctaccgctg

ggtcgacgatcgcgcgttcaccgtcgcgatcgcgccgcatgcgccacccgtcgcgacgttcgcgaccgacgcgcgcacc

gtcgtcggcgcggacccggactacgcgcaactcgtcgccgacgcgctcggccggcggctcgcgctcgtgcccgtcgcct

gggccgactggccgctcgggctgagcgccggcaagtacgacgcggtgatctcgaacgtcggcgtcaccgagcagcgcaa

gcggaagttcgatttcacgacgtaccggctcggcctgcacggcttctatgtgcgggccgcgagcccgatcgcgcggatc

gccgcgccgcgcgacgtggcgggcctgcggatcatcaccgcgtcgggaacgagccaggagcgcatcctgctcgaatggg

accggcgcaacgtcgcgcagcggctcaagccggtggacgtgctgtacttcgacgacgacgccgcatcgcgcgtcgcgct

gctctcggggcgcgccgacgcggagctgaatccgaacgcgacactcgcgtaccaggcggcgcgcgacggcaggattcgt

tgcgtcggcaacgtgaacgcgggctggccgctcaaggccgacgtcgcgatcgcgacgcgcaagggcagcggcctcgccg

acgcgctcacgctcgcgaccaacggcctgatccgcaacggcaagtaccggcaggcgctcgcgcgatgggggctgctgtc

ggaggcgctcgaccggtcggagacgaatccggcggggctgccgtcgttctga

>Bucl13_Bm_2000031281.seq Reverse Complement DNA Sequence AWGQ01000104.seq(1,1158)

ATGTGTCGACGGCGTCGCGCAGTCGCGCGCGCTCGCATTGCGTCGCGCGGCGTCGATAGTGTCGATCATGTCGGAAGTG

GCGGAAGTGGCGGAAGCATCAGAAGCATCAGAAGCATCAGGGGCATCAGGGGCATCAGAGGTATCAGAGGTATCAGAGG

TATCAGAGGCGTCAGAGGCGTCAGAGGCGTCAGAGGCATTAGAAGCATCGGAGGCACCGAAGCGATGACCGACCGACGC

ACATTCGTCGGCGGCGCGGCGGCCTTCGCGGCCGCATGGGCCGCCGCGCCGTTCGGCGCGCGCGCCGGCATCGCGGTCG

ATCTCGACCCGCGTCAGGCGGGCCGCGTGCGCGCCGAACGCGATCCGGCCGCCGTCGACGCCGCGCGCCGCTACCGCTG

GGTCGACGATCGCGCGTTCACCGTCGCGATCGCGCCGCATGCGCCACCCGTCGCGACGTTCGCGACCGACGCGCGCACC

GTCGTCGGCGCGGACCCGGACTACGCGCAACTCGTCGCCGACGCGCTCGGCCGGCGGCTCGCGCTCGTGCCCGTCGCCT

GGGCCGACTGGCCGCTCGGGCTGAGCGCCGGCAAGTACGACGCGGTGATCTCGAACGTCGGCGTCACCGAGCAGCGCAA

GCGGAAGTTCGATTTCACGACGTACCGGCTCGGCCTGCACGGCTTCTATGTGCGGGCCGCGAGCCCGATCGCGCGGATC

GCCGCGCCGCGCGACGTGGCGGGCCTGCGGATCATCACCGCGTCGGGAACGAGCCAGGAGCGCATCCTGCTCGAATGGG

ACCGGCGCAACGTCGCGCAGCGGCTCAAGCCGGTGGACGTGCTGTACTTCGACGACGACGCCGCATCGCGCGTCGCGCT

GCTCTCGGGGCGCGCCGACGCGGAGCTGAATCCGAACGCGACACTCGCGTACCAGGCGGCGCGCGACGGCAGGATTCGT

TGCGTCGGCAACGTGAACGCGGGCTGGCCGCTCAAGGCCGACGTCGCGATCGCGACGCGCAAGGGCAGCGGCCTCGCCG

ACGCGCTCACGCTCGCGACCAACGGCCTGATCCGCAACGGCAAGTACCGGCAGGCGCTCGCGCGATGGGGGCTGCTGTC

GGAGGCGCTCGACCGGTCGGAGACGAATCCGGCGGGGCTGCCGTCGTTCTGA

>Bucl13_Bm_2002721280.seq Reverse Complement DNA Sequence AANX02000001.seq(1,1212)

ATGTGTCGACGGCGTCGCGCAGTCGCGCGCGCTCGCATTGCGTCGCGCGGCGTCGATAGTGTCGATCATGTCGGAAGTG

GCGGAAGTGGCGGAAGTGGCGGAAGCATCAGAAGCATCAGAAGCATCAGGGGCATCAGGGGCATCAGAGGTATCAGAGG

TATCAGAGGTATCAGAGGTATCAGAGGTATCAGAGGTATCAGAGGTATCAGAGGTATCAGAGGCGTCAGAGGCGTCAGA

GGCGTCAGAGGCATTAGAAGCATCGGAGGCACCGAAGCGATGACCGACCGACGCACATTCGTCGGCGGCGCGGCGGCCT

TCGCGGCCGCATGGGCCGCCGCGCCGTTCGGCGCGCGCGCCGGCATCGCGGTCGATCTCGACCCGCGTCAGGCGGGCCG

CGTGCGCGCCGAACGCGATCCGGCCGCCGTCGACGCCGCGCGCCGCTACCGCTGGGTCGACGATCGCGCGTTCACCGTC

GCGATCGCGCCGCATGCGCCACCCGTCGCGACGTTCGCGACCGACGCGCGCACCGTCGTCGGCGCGGACCCGGACTACG

CGCAACTCGTCGCCGACGCGCTCGGCCGGCGGCTCGCGCTCGTGCCCGTCGCCTGGGCCGACTGGCCGCTCGGGCTGAG

CGCCGGCAAGTACGACGCGGTGATCTCGAACGTCGGCGTCACCGAGCAGCGCAAGCGGAAGTTCGATTTCACGACGTAC

CGGCTCGGCCTGCACGGCTTCTATGTGCGGGCCGCGAGCCCGATCGCGCGGATCGCCGCGCCGCGCGACGTGGCGGGCC

TGCGGATCATCACCGCGTCGGGAACGAGCCAGGAGCGCATCCTGCTCGAATGGGACCGGCGCAACGTCGCGCAGCGGCT

CAAGCCGGTGGACGTGCTGTACTTCGACGACGACGCCGCATCGCGCGTCGCGCTGCTCTCGGGGCGCGCCGACGCGGAG

CTGAATCCGAACGCGACACTCGCGTACCAGGCGGCGCGCGACGGCAGGATTCGTTGCGTCGGCAACGTGAACGCGGGCT

GGCCGCTCAAGGCCGACGTCGCGATCGCGACGCGCAAGGGCAGCGGCCTCGCCGACGCGCTCACGCTCGCGACCAACGG

CCTGATCCGCAACGGCAAGTACCGGCAGGCGCTCGCGCGATGGGGGCTGCTGTCGGAGGCGCTCGACCGGTCGGAGACG

AATCCGGCGGGGCTGCCGTCGTTCTGA

>Bucl13_Bm_A188.seq Reverse Complement DNA Sequence AWGR01000148.seq(1,1167)

ATGTGTCGACGGCGTCGCGCAGTCGCGCGCGCTCGCATTGCGTCGCGCGGCGTCGATAGTGTCGATCATGTCGGAAGTG

GCGGAAGTGGCGGAAGCATCAGAAGCATCAGAAGCATCAGGGGCATCAGGGGCATCAGAGGTATCAGAGGTATCAGAGG

TATCAGAGGTATCAGAGGCGTCAGAGGCGTCAGAGGCGTCAGAGGCATTAGAAGCATCGGAGGCACCGAAGCGATGACC

GACCGACGCACATTCGTCGGCGGCGCGGCGGCCTTCGCGGCCGCATGGGCCGCCGCGCCGTTCGGCGCGCGCGCCGGCA

TCGCGGTCGATCTCGACCCGCGTCAGGCGGGCCGCGTGCGCGCCGAACGCGATCCGGCCGCCGTCGACGCCGCGCGCCG

CTACCGCTGGGTCGACGATCGCGCGTTCACCGTCGCGATCGCGCCGCATGCGCCACCCGTCGCGACGTTCGCGACCGAC

GCGCGCACCGTCGTCGGCGCGGACCCGGACTACGCGCAACTCGTCGCCGACGCGCTCGGCCGGCGGCTCGCGCTCGTGC

CCGTCGCCTGGGCCGACTGGCCGCTCGGGCTGAGCGCCGGCAAGTACGACGCGGTGATCTCGAACGTCGGCGTCACCGA

GCAGCGCAAGCGGAAGTTCGATTTCACGACGTACCGGCTCGGCCTGCACGGCTTCTATGTGCGGGCCGCGAGCCCGATC

GCGCGGATCGCCGCGCCGCGCGACGTGGCGGGCCTGCGGATCATCACCGCGTCGGGAACGAGCCAGGAGCGCATCCTGC

TCGAATGGGACCGGCGCAACGTCGCGCAGCGGCTCAAGCCGGTGGACGTGCTGTACTTCGACGACGACGCCGCATCGCG

CGTCGCGCTGCTCTCGGGGCGCGCCGACGCGGAGCTGAATCCGAACGCGACACTCGCGTACCAGGCGGCGCGCGACGGC

AGGATTCGTTGCGTCGGCAACGTGAACGCGGGCTGGCCGCTCAAGGCCGACGTCGCGATCGCGACGCGCAAGGGCAGCG

GCCTCGCCGACGCGCTCACGCTCGCGACCAACGGCCTGATCCGCAACGGCAAGTACCGGCAGGCGCTCGCGCGATGGGG

GCTGCTGTCGGAGGCGCTCGACCGGTCGGAGACGAATCCGGCGGGGCTGCCGTCGTTCTGA

>Bucl13_Bm_A193.seq Reverse Complement DNA Sequence AWGS01000151.seq(1,1194)

ATGTGTCGACGGCGTCGCGCAGTCGCGCGCGCTCGCATTGCGTCGCGCGGCGTCGATAGTGTCGATCATGTCGGAAGTG

GCGGAAGTGGCGGAAGTGGCGGAAGTGGCGGAAGTGGCGGAAGTGGCGGAAGCATCAGAAGCATCAGAAGCATCAGGGG

CATCAGGGGCATCAGAGGTATCAGAGGTATCAGAGGTATCAGAGGCGTCAGAGGCGTCAGAGGCGTCAGAGGCATTAGA

AGCATCGGAGGCACCGAAGCGATGACCGACCGACGCACATTCGTCGGCGGCGCGGCGGCCTTCGCGGCCGCATGGGCCG

CCGCGCCGTTCGGCGCGCGCGCCGGCATCGCGGTCGATCTCGACCCGCGTCAGGCGGGCCGCGTGCGCGCCGAACGCGA

TCCGGCCGCCGTCGACGCCGCGCGCCGCTACCGCTGGGTCGACGATCGCGCGTTCACCGTCGCGATCGCGCCGCATGCG

CCACCCGTCGCGACGTTCGCGACCGACGCGCGCACCGTCGTCGGCGCGGACCCGGACTACGCGCAACTCGTCGCCGACG

CGCTCGGCCGGCGGCTCGCGCTCGTGCCCGTCGCCTGGGCCGACTGGCCGCTCGGGCTGAGCGCCGGCAAGTACGACGC

GGTGATCTCGAACGTCGGCGTCACCGAGCAGCGCAAGCGGAAGTTCGATTTCACGACGTACCGGCTCGGCCTGCACGGC

TTCTATGTGCGGGCCGCGAGCCCGATCGCGCGGATCGCCGCGCCGCGCGACGTGGCGGGCCTGCGGATCATCACCGCGT

CGGGAACGAGCCAGGAGCGCATCCTGCTCGAATGGGACCGGCGCAACGTCGCGCAGCGGCTCAAGCCGGTGGACGTGCT

GTACTTCGACGACGACGCCGCATCGCGCGTCGCGCTGCTCTCGGGGCGCGCCGACGCGGAGCTGAATCCGAACGCGACA

CTCGCGTACCAGGCGGCGCGCGACGGCAGGATTCGTTGCGTCGGCAACGTGAACGCGGGCTGGCCGCTCAAGGCCGACG

TCGCGATCGCGACGCGCAAGGGCAGCGGCCTCGCCGACGCGCTCACGCTCGCGACCAACGGCCTGATCCGCAACGGCAA

GTACCGGCAGGCGCTCGCGCGATGGGGGCTGCTGTCGGAGGCGCTCGACCGGTCGGAGACGAATCCGGCGGGGCTGCCG

TCGTTCTGA

>Bucl13_Bm_ATCC_10399.seq >gi|99031410|gb|AAHN02000005.1|:176454-177620 Burkholderia mallei ATCC 10399 ctg_1047277000589, whole genome shotgun sequence

ATGTGTCGACGGCGTCGCGCAGTCGCGCGCGCTCGCATTGCGTCGCGCGGCGTCGATAGTGTCGATCATGTCGGAAGTG

GCGGAAGTGGCGGAAGTGGCGGAAGCATCAGAAGCATCAGAAGCATCAGGGGCATCAGGGGCATCAGAGGTATCAGAGG

TATCAGAGGTATCAGAGGCGTCAGAGGCGTCAGAGGCGTCAGAGGCATTAGAAGCATCGGAGGCACCGAAGCGATGACC

GACCGACGCACATTCGTCGGCGGCGCGGCGGCCTTCGCGGCCGCATGGGCCGCCGCGCCGTTCGGCGCGCGCGCCGGCA

TCGCGGTCGATCTCGACCCGCGTCAGGCGGGCCGCGTGCGCGCCGAACGCGATCCGGCCGCCGTCGACGCCGCGCGCCG

CTACCGCTGGGTCGACGATCGCGCGTTCACCGTCGCGATCGCGCCGCATGCGCCACCCGTCGCGACGTTCGCGACCGAC

GCGCGCACCGTCGTCGGCGCGGACCCGGACTACGCGCAACTCGTCGCCGACGCGCTCGGCCGGCGGCTCGCGCTCGTGC

CCGTCGCCTGGGCCGACTGGCCGCTCGGGCTGAGCGCCGGCAAGTACGACGCGGTGATCTCGAACGTCGGCGTCACCGA

GCAGCGCAAGCGGAAGTTCGATTTCACGACGTACCGGCTCGGCCTGCACGGCTTCTATGTGCGGGCCGCGAGCCCGATC

GCGCGGATCGCCGCGCCGCGCGACGTGGCGGGCCTGCGGATCATCACCGCGTCGGGAACGAGCCAGGAGCGCATCCTGC

TCGAATGGGACCGGCGCAACGTCGCGCAGCGGCTCAAGCCGGTGGACGTGCTGTACTTCGACGACGACGCCGCATCGCG

CGTCGCGCTGCTCTCGGGGCGCGCCGACGCGGAGCTGAATCCGAACGCGACACTCGCGTACCAGGCGGCGCGCGACGGC

AGGATTCGTTGCGTCGGCAACGTGAACGCGGGCTGGCCGCTCAAGGCCGACGTCGCGATCGCGACGCGCAAGGGCAGCG

GCCTCGCCGACGCGCTCACGCTCGCGACCAACGGCCTGATCCGCAACGGCAAGTACCGGCAGGCGCTCGCGCGATGGGG

GCTGCTGTCGGAGGCGCTCGACCGGTCGGAGACGAATCCGGCGGGGCTGCCGTCGTTCTGA

>Bucl13_Bm_JHU.seq Reverse Complement DNA Sequence AAIR02000014.seq(1,1158)

ATGTGTCGACGGCGTCGCGCAGTCGCGCGCGCTCGCATTGCGTCGCGCGGCGTCGATAGTGTCGATCATGTCGGAAGTG

GCGGAAGTGGCGGAAGCATCAGAAGCATCAGAAGCATCAGGGGCATCAGGGGCATCAGAGGTATCAGAGGTATCAGAGG

TATCAGAGGCGTCAGAGGCGTCAGAGGCGTCAGAGGCATTAGAAGCATCGGAGGCACCGAAGCGATGACCGACCGACGC

ACATTCGTCGGCGGCGCGGCGGCCTTCGCGGCCGCATGGGCCGCCGCGCCGTTCGGCGCGCGCGCCGGCATCGCGGTCG

ATCTCGACCCGCGTCAGGCGGGCCGCGTGCGCGCCGAACGCGATCCGGCCGCCGTCGACGCCGCGCGCCGCTACCGCTG

GGTCGACGATCGCGCGTTCACCGTCGCGATCGCGCCGCATGCGCCACCCGTCGCGACGTTCGCGACCGACGCGCGCACC

GTCGTCGGCGCGGACCCGGACTACGCGCAACTCGTCGCCGACGCGCTCGGCCGGCGGCTCGCGCTCGTGCCCGTCGCCT

GGGCCGACTGGCCGCTCGGGCTGAGCGCCGGCAAGTACGACGCGGTGATCTCGAACGTCGGCGTCACCGAGCAGCGCAA

GCGGAAGTTCGATTTCACGACGTACCGGCTCGGCCTGCACGGCTTCTATGTGCGGGCCGCGAGCCCGATCGCGCGGATC

GCCGCGCCGCGCGACGTGGCGGGCCTGCGGATCATCACCGCGTCGGGAACGAGCCAGGAGCGCATCCTGCTCGAATGGG

ACCGGCGCAACGTCGCGCAGCGGCTCAAGCCGGTGGACGTGCTGTACTTCGACGACGACGCCGCATCGCGCGTCGCGCT

GCTCTCGGGGCGCGCCGACGCGGAGCTGAATCCGAACGCGACACTCGCGTACCAGGCGGCGCGCGACGGCAGGATTCGT

TGCGTCGGCAACGTGAACGCGGGCTGGCCGCTCAAGGCCGACGTCGCGATCGCGACGCGCAAGGGCAGCGGCCTCGCCG

ACGCGCTCACGCTCGCGACCAACGGCCTGATCCGCAACGGCAAGTACCGGCAGGCGCTCGCGCGATGGGGGCTGCTGTC

GGAGGCGCTCGACCGGTCGGAGACGAATCCGGCGGGGCTGCCGTCGTTCTGA

>Bucl13_Bm_China_7.seq >gi|577031349|gb|AWGN01000011.1|:7368-8525 Burkholderia mallei China_7 Burkholderia-mallei-MCB-010_1293, whole genome shotgun sequence

ATGTGTCGACGGCGTCGCGCAGTCGCGCGCGCTCGCATTGCGTCGCGCGGCGTCGATAGTGTCGATCATGTCGGAAGTG

GCGGAAGTGGCGGAAGCATCAGAAGCATCAGAAGCATCAGGGGCATCAGGGGCATCAGAGGTATCAGAGGTATCAGAGG

TATCAGAGGCGTCAGAGGCGTCAGAGGCGTCAGAGGCATTAGAAGCATCGGAGGCACCGAAGCGATGACCGACCGACGC

ACATTCGTCGGCGGCGCGGCGGCCTTCGCGGCCGCATGGGCCGCCGCGCCGTTCGGCGCGCGCGCCGGCATCGCGGTCG

ATCTCGACCCGCGTCAGGCGGGCCGCGTGCGCGCCGAACGCGATCCGGCCGCCGTCGACGCCGCGCGCCGCTACCGCTG

GGTCGACGATCGCGCGTTCACCGTCGCGATCGCGCCGCATGCGCCACCCGTCGCGACGTTCGCGACCGACGCGCGCACC

GTCGTCGGCGCGGACCCGGACTACGCGCAACTCGTCGCCGACGCGCTCGGCCGGCGGCTCGCGCTCGTGCCCGTCGCCT

GGGCCGACTGGCCGCTCGGGCTGAGCGCCGGCAAGTACGACGCGGTGATCTCGAACGTCGGCGTCACCGAGCAGCGCAA

GCGGAAGTTCGATTTCACGACGTACCGGCTCGGCCTGCACGGCTTCTATGTGCGGGCCGCGAGCCCGATCGCGCGGATC

GCCGCGCCGCGCGACGTGGCGGGCCTGCGGATCATCACCGCGTCGGGAACGAGCCAGGAGCGCATCCTGCTCGAATGGG

ACCGGCGCAACGTCGCGCAGCGGCTCAAGCCGGTGGACGTGCTGTACTTCGACGACGACGCCGCATCGCGCGTCGCGCT

GCTCTCGGGGCGCGCCGACGCGGAGCTGAATCCGAACGCGACACTCGCGTACCAGGCGGCGCGCGACGGCAGGATTCGT

TGCGTCGGCAACGTGAACGCGGGCTGGCCGCTCAAGGCCGACGTCGCGATCGCGACGCGCAAGGGCAGCGGCCTCGCCG

ACGCGCTCACGCTCGCGACCAACGGCCTGATCCGCAACGGCAAGTACCGGCAGGCGCTCGCGCGATGGGGGCTGCTGTC

GGAGGCGCTCGACCGGTCGGAGACGAATCCGGCGGGGCTGCCGTCGTTCTGA

>Bucl13_Bm_FMH.seq Reverse Complement DNA Sequence AAIQ02000094.seq(1,1158)

ATGTGTCGACGGCGTCGCGCAGTCGCGCGCGCTCGCATTGCGTCGCGCGGCGTCGATAGTGTCGATCATGTCGGAAGTG

GCGGAAGTGGCGGAAGCATCAGAAGCATCAGAAGCATCAGGGGCATCAGGGGCATCAGAGGTATCAGAGGTATCAGAGG

TATCAGAGGCGTCAGAGGCGTCAGAGGCGTCAGAGGCATTAGAAGCATCGGAGGCACCGAAGCGATGACCGACCGACGC

ACATTCGTCGGCGGCGCGGCGGCCTTCGCGGCCGCATGGGCCGCCGCGCCGTTCGGCGCGCGCGCCGGCATCGCGGTCG

ATCTCGACCCGCGTCAGGCGGGCCGCGTGCGCGCCGAACGCGATCCGGCCGCCGTCGACGCCGCGCGCCGCTACCGCTG

GGTCGACGATCGCGCGTTCACCGTCGCGATCGCGCCGCATGCGCCACCCGTCGCGACGTTCGCGACCGACGCGCGCACC

GTCGTCGGCGCGGACCCGGACTACGCGCAACTCGTCGCCGACGCGCTCGGCCGGCGGCTCGCGCTCGTGCCCGTCGCCT

GGGCCGACTGGCCGCTCGGGCTGAGCGCCGGCAAGTACGACGCGGTGATCTCGAACGTCGGCGTCACCGAGCAGCGCAA

GCGGAAGTTCGATTTCACGACGTACCGGCTCGGCCTGCACGGCTTCTATGTGCGGGCCGCGAGCCCGATCGCGCGGATC

GCCGCGCCGCGCGACGTGGCGGGCCTGCGGATCATCACCGCGTCGGGAACGAGCCAGGAGCGCATCCTGCTCGAATGGG

ACCGGCGCAACGTCGCGCAGCGGCTCAAGCCGGTGGACGTGCTGTACTTCGACGACGACGCCGCATCGCGCGTCGCGCT

GCTCTCGGGGCGCGCCGACGCGGAGCTGAATCCGAACGCGACACTCGCGTACCAGGCGGCGCGCGACGGCAGGATTCGT

TGCGTCGGCAACGTGAACGCGGGCTGGCCGCTCAAGGCCGACGTCGCGATCGCGACGCGCAAGGGCAGCGGCCTCGCCG

ACGCGCTCACGCTCGCGACCAACGGCCTGATCCGCAACGGCAAGTACCGGCAGGCGCTCGCGCGATGGGGGCTGCTGTC

GGAGGCGCTCGACCGGTCGGAGACGAATCCGGCGGGGCTGCCGTCGTTCTGA

>Bucl13_Bm_GB8_horse_4.seq Reverse Complement DNA Sequence AAHO01000048.seq(1,1158)

ATGTGTCGACGGCGTCGCGCAGTCGCGCGCGCTCGCATTGCGTCGCGCGGCGTCGATAGTGTCGATCATGTCGGAAGTG

GCGGAAGTGGCGGAAGCATCAGAAGCATCAGAAGCATCAGGGGCATCAGGGGCATCAGAGGTATCAGAGGTATCAGAGG

TATCAGAGGCGTCAGAGGCGTCAGAGGCGTCAGAGGCATTAGAAGCATCGGAGGCACCGAAGCGATGACCGACCGACGC

ACATTCGTCGGCGGCGCGGCGGCCTTCGCGGCCGCATGGGCCGCCGCGCCGTTCGGCGCGCGCGCCGGCATCGCGGTCG

ATCTCGACCCGCGTCAGGCGGGCCGCGTGCGCGCCGAACGCGATCCGGCCGCCGTCGACGCCGCGCGCCGCTACCGCTG

GGTCGACGATCGCGCGTTCACCGTCGCGATCGCGCCGCATGCGCCACCCGTCGCGACGTTCGCGACCGACGCGCGCACC

GTCGTCGGCGCGGACCCGGACTACGCGCAACTCGTCGCCGACGCGCTCGGCCGGCGGCTCGCGCTCGTGCCCGTCGCCT

GGGCCGACTGGCCGCTCGGGCTGAGCGCCGGCAAGTACGACGCGGTGATCTCGAACGTCGGCGTCACCGAGCAGCGCAA

GCGGAAGTTCGATTTCACGACGTACCGGCTCGGCCTGCACGGCTTCTATGTGCGGGCCGCGAGCCCGATCGCGCGGATC

GCCGCGCCGCGCGACGTGGCGGGCCTGCGGATCATCACCGCGTCGGGAACGAGCCAGGAGCGCATCCTGCTCGAATGGG

ACCGGCGCAACGTCGCGCAGCGGCTCAAGCCGGTGGACGTGCTGTACTTCGACGACGACGCCGCATCGCGCGTCGCGCT

GCTCTCGGGGCGCGCCGACGCGGAGCTGAATCCGAACGCGACACTCGCGTACCAGGCGGCGCGCGACGGCAGGATTCGT

TGCGTCGGCAACGTGAACGCGGGCTGGCCGCTCAAGGCCGACGTCGCGATCGCGACGCGCAAGGGCAGCGGCCTCGCCG

ACGCGCTCACGCTCGCGACCAACGGCCTGATCCGCAACGGCAAGTACCGGCAGGCGCTCGCGCGATGGGGGCTGCTGTC

GGAGGCGCTCGACCGGTCGGAGACGAATCCGGCGGGGCTGCCGTCGTTCTGA

>Bucl13_Bm_PRL-20.seq Reverse Complement DNA Sequence AAZP01000008.seq(1,1176)

ATGTGTCGACGGCGTCGCGCAGTCGCGCGCGCTCGCATTGCGTCGCGCGGCGTCGATAGTGTCGATCATGTCGGAAGTG

GCGGAAGTGGCGGAAGTGGCGGAAGCATCAGAAGCATCAGAAGCATCAGGGGCATCAGGGGCATCAGAGGTATCAGAGG

TATCAGAGGTATCAGAGGTATCAGAGGCGTCAGAGGCGTCAGAGGCGTCAGAGGCATTAGAAGCATCGGAGGCACCGAA

GCGATGACCGACCGACGCACATTCGTCGGCGGCGCGGCGGCCTTCGCGGCCGCATGGGCCGCCGCGCCGTTCGGCGCGC

GCGCCGGCATCGCGGTCGATCTCGACCCGCGTCAGGCGGGCCGCGTGCGCGCCGAACGCGATCCGGCCGCCGTCGACGC

CGCGCGCCGCTACCGCTGGGTCGACGATCGCGCGTTCACCGTCGCGATCGCGCCGCATGCGCCACCCGTCGCGACGTTC

GCGACCGACGCGCGCACCGTCGTCGGCGCGGACCCGGACTACGCGCAACTCGTCGCCGACGCGCTCGGCCGGCGGCTCG

CGCTCGTGCCCGTCGCCTGGGCCGACTGGCCGCTCGGGCTGAGCGCCGGCAAGTACGACGCGGTGATCTCGAACGTCGG

CGTCACCGAGCAGCGCAAGCGGAAGTTCGATTTCACGACGTACCGGCTCGGCCTGCACGGCTTCTATGTGCGGGCCGCG

AGCCCGATCGCGCGGATCGCCGCGCCGCGCGACGTGGCGGGCCTGCGGATCATCACCGCGTCGGGAACGAGCCAGGAGC

GCATCCTGCTCGAATGGGACCGGCGCAACGTCGCGCAGCGGCTCAAGCCGGTGGACGTGCTGTACTTCGACGACGACGC

CGCATCGCGCGTCGCGCTGCTCTCGGGGCGCGCCGACGCGGAGCTGAATCCGAACGCGACACTCGCGTACCAGGCGGCG

CGCGACGGCAGGATTCGTTGCGTCGGCAACGTGAACGCGGGCTGGCCGCTCAAGGCCGACGTCGCGATCGCGACGCGCA

AGGGCAGCGGCCTCGCCGACGCGCTCACGCTCGCGACCAACGGCCTGATCCGCAACGGCAAGTACCGGCAGGCGCTCGC

GCGATGGGGGCTGCTGTCGGAGGCGCTCGACCGGTCGGAGACGAATCCGGCGGGGCTGCCGTCGTTCTGA

>Bucl13_Bm_strain_6.seq >gi|577030879|gb|AWGO01000153.1|:7094-8278 Burkholderia mallei strain_6 Burkholderia-mallei-MCB-011_1054, whole genome shotgun sequence

ATGTGTCGACGGCGTCGCGCAGTCGCGCGCGCTCGCATTGCGTCGCGCGGCGTCGATAGTGTCGATCATGTCGGAAGTG

GCGGAAGTGGCGGAAGTGGCGGAAGTGGCGGAAGTGGCGGAAGTGGCGGAAGCATCAGAAGCATCAGAAGCATCAGGGG

CATCAGGGGCATCAGAGGTATCAGAGGTATCAGAGGCGTCAGAGGCGTCAGAGGCGTCAGAGGCATTAGAAGCATCGGA

GGCACCGAAGCGATGACCGACCGACGCACATTCGTCGGCGGCGCGGCGGCCTTCGCGGCCGCATGGGCCGCCGCGCCGT

TCGGCGCGCGCGCCGGCATCGCGGTCGATCTCGACCCGCGTCAGGCGGGCCGCGTGCGCGCCGAACGCGATCCGGCCGC

CGTCGACGCCGCGCGCCGCTACCGCTGGGTCGACGATCGCGCGTTCACCGTCGCGATCGCGCCGCATGCGCCACCCGTC

GCGACGTTCGCGACCGACGCGCGCACCGTCGTCGGCGCGGACCCGGACTACGCGCAACTCGTCGCCGACGCGCTCGGCC

GGCGGCTCGCGCTCGTGCCCGTCGCCTGGGCCGACTGGCCGCTCGGGCTGAGCGCCGGCAAGTACGACGCGGTGATCTC

GAACGTCGGCGTCACCGAGCAGCGCAAGCGGAAGTTCGATTTCACGACGTACCGGCTCGGCCTGCACGGCTTCTATGTG

CGGGCCGCGAGCCCGATCGCGCGGATCGCCGCGCCGCGCGACGTGGCGGGCCTGCGGATCATCACCGCGTCGGGAACGA

GCCAGGAGCGCATCCTGCTCGAATGGGACCGGCGCAACGTCGCGCAGCGGCTCAAGCCGGTGGACGTGCTGTACTTCGA

CGACGACGCCGCATCGCGCGTCGCGCTGCTCTCGGGGCGCGCCGACGCGGAGCTGAATCCGAACGCGACACTCGCGTAC

CAGGCGGCGCGCGACGGCAGGATTCGTTGCGTCGGCAACGTGAACGCGGGCTGGCCGCTCAAGGCCGACGTCGCGATCG

CGACGCGCAAGGGCAGCGGCCTCGCCGACGCGCTCACGCTCGCGACCAACGGCCTGATCCGCAACGGCAAGTACCGGCA

GGCGCTCGCGCGATGGGGGCTGCTGTCGGAGGCGCTCGACCGGTCGGAGACGAATCCGGCGGGGCTGCCGTCGTTCTGA

>Bucl13_Bm_strain_11.seq >gi|577030871|gb|AWGP01000163.1|:7398-8600 Burkholderia mallei strain_11 Burkholderia-mallei-MCB-012_2213, whole genome shotgun sequence

ATGTGTCGACGGCGTCGCGCAGTCGCGCGCGCTCGCATTGCGTCGCGCGGCGTCGATAGTGTCGATCATGTCGGAAGTG

GCGGAAGTGGCGGAAGTGGCGGAAGCATCAGAAGCATCAGAAGCATCAGGGGCATCAGGGGCATCAGAGGTATCAGAGG

TATCAGAGGTATCAGAGGTATCAGAGGTATCAGAGGTATCAGAGGTATCAGAGGCGTCAGAGGCGTCAGAGGCGTCAGA

GGCATTAGAAGCATCGGAGGCACCGAAGCGATGACCGACCGACGCACATTCGTCGGCGGCGCGGCGGCCTTCGCGGCCG

CATGGGCCGCCGCGCCGTTCGGCGCGCGCGCCGGCATCGCGGTCGATCTCGACCCGCGTCAGGCGGGCCGCGTGCGCGC

CGAACGCGATCCGGCCGCCGTCGACGCCGCGCGCCGCTACCGCTGGGTCGACGATCGCGCGTTCACCGTCGCGATCGCG

CCGCATGCGCCACCCGTCGCGACGTTCGCGACCGACGCGCGCACCGTCGTCGGCGCGGACCCGGACTACGCGCAACTCG

TCGCCGACGCGCTCGGCCGGCGGCTCGCGCTCGTGCCCGTCGCCTGGGCCGACTGGCCGCTCGGGCTGAGCGCCGGCAA

GTACGACGCGGTGATCTCGAACGTCGGCGTCACCGAGCAGCGCAAGCGGAAGTTCGATTTCACGACGTACCGGCTCGGC

CTGCACGGCTTCTATGTGCGGGCCGCGAGCCCGATCGCGCGGATCGCCGCGCCGCGCGACGTGGCGGGCCTGCGGATCA

TCACCGCGTCGGGAACGAGCCAGGAGCGCATCCTGCTCGAATGGGACCGGCGCAACGTCGCGCAGCGGCTCAAGCCGGT

GGACGTGCTGTACTTCGACGACGACGCCGCATCGCGCGTCGCGCTGCTCTCGGGGCGCGCCGACGCGGAGCTGAATCCG

AACGCGACACTCGCGTACCAGGCGGCGCGCGACGGCAGGATTCGTTGCGTCGGCAACGTGAACGCGGGCTGGCCGCTCA

AGGCCGACGTCGCGATCGCGACGCGCAAGGGCAGCGGCCTCGCCGACGCGCTCACGCTCGCGACCAACGGCCTGATCCG

CAACGGCAAGTACCGGCAGGCGCTCGCGCGATGGGGGCTGCTGTCGGAGGCGCTCGACCGGTCGGAGACGAATCCGGCG

GGGCTGCCGTCGTTCTGA

>Bucl13_Bps_MSHR146.seq Reverse Complement DNA Sequence CP004043.seq(1,1221)

ATGTGTCGACGGCGTCGCGCAGTCGCGCGCGCTCGCATTGCGTCGCGCGGCGTCGATAGTGTCGATCATGTCGGAAGTG

GCGGAAGTGGCGGAAGCATCAGAAGCATCAGGGGCATCAGGGGCATCAGAGGTATCAGAGGTATCAGAGGTATCAGAGG

TATCAGAGGTATCAGAGGTATCAGAGGTATCAGAGGCGTCAGAGGCATTAGAAGCGTCAGCGGCATTAGAAACGTCAGC

GGCATTAGAAACGTCAGCGGCATTAGAAGCATCGGAGGCACCGAAGCGATGACCGACCGACGCACATTCGTCGGCGGCG

CGGCGGCCTTCGCGGCCGCATGGGCCGCCGCGCCGTTCGGCGCGCGCGCCGGCATCGCGGTCGATCTCGACCCGCGTCA

GGCGGGCCGCGTGCGCGCCGAACGCGATCCGGCCGTCGTCGACGCCACGCGCCGCTACCGCTGGGTCGACGATCGCGCG

TTCACCGTCGCGATCGCGCCGCATGCGCCACCCGTCGCGACGTTCGCGACCGACGCGCGCACCGTCGTCGGCGCGGACC

CCGACTACGCGCAACTCGTCGCCGACGCGCTCAGCCGGCGGCTCGCGCTCGTGCCCGTCGCCTGGGCCGACTGGCCGCT

CGGGCTGAGCGCCGGCAAGTACGACGCGGTGATCTCGAACGTCGGCGTCACCGAGCAGCGCAAGCGGAAGTTCGATTTC

ACGACGTACCGGCTCAGCCTGCACGGCTTCTATGTGCGGGCCGCGAGCCCGATCGCGCGGATCGCCGCGCCGCACGACG

TGGCGGGCCTGCGGATCATCACCGCGTCGGGAACGAGCCAGGAGCGCATCCTGCTCGAATGGGACCGGCGCAACGTCGC

GCAGCGGCTCAAGCCGGTGGACGTGCTGTACTTCGACGACGACGCCGCATCGCGCGTCGCGCTGCTCTCGGGGCGCGCC

GACGCGGAGCTGAATCCGAACGCGACACTCGCGTACCAGGCGGCGCGCGACGGCAGGATTCGTTGCGTCGGCAACGTGA

ACGCGGGCTGGCCGCTCAAGGCCGACGTCGCGATCGCGACGCGCAAGGGCAGCGGCCTCGCCGACGCGCTCACGCTCGC

GACCAACGGCCTGATCCGCAACGGCAAGTACCGGCAGGCGCTCGCGCGATGGGGGCTGCTGTCGGAGGCGCTCGACCGG

TCGGAGACGAATCCGGCGGGGCTGCCGTCGTTCTGA

>Bucl13_Bps_MSHR511.seq >gi|574604118|gb|CP004024.1|:1133861-1135090 Burkholderia pseudomallei MSHR511 chromosome 2, complete sequence

ATGTGTCGACGGCGTCGCGCAGTCGCGCGCGCTCGCATTGCGTCGCGCGGCGTCGATAGTGTCGATCATGTCGGAAGTG

GCGGAAGTGGCGGAAGCATCAGAAGCATCAGGGGCATCAGGGGCATCAGAGGTATCAGAGGTATCAGAGGTATCAGAGG

TATCAGAGGTATCAGAGGTATCAGAGGTATCAGAGGTATCAGAGGCGTCAGAGGCATTAGAAGCGTCAGCGGCATTAGA

AACGTCAGCGGCATTAGAAACGTCAGCGGCATTAGAAGCATCGGAGGCACCGAAGCGATGACCGACCGACGCACATTCG

TCGGCGGCGCGGCGGCCTTCGCGGCCGCATGGGCCGCCGCGCCGTTCGGCGCGCGCGCCGGCATCGCGGTCGATCTCGA

CCCGCGTCAGGCGGGCCGCGTGCGCGCCGAACGCGATCCGGCCGTCGTCGACGCCACGCGCCGCTACCGCTGGGTCGAC

GATCGCGCGTTCACCGTCGCGATCGCGCCGCATGCGCCACCCGTCGCGACGTTCGCGACCGACGCGCGCACCGTCGTCG

GCGCGGACCCCGACTACGCGCAACTCGTCGCCGACGCGCTCAGCCGGCGGCTCGCGCTCGTGCCCGTCGCCTGGGCCGA

CTGGCCGCTCGGGCTGAGCGCCGGCAAGTACGACGCGGTGATCTCGAACGTCGGCGTCACCGAGCAGCGCAAGCGGAAG

TTCGATTTCACGACGTACCGGCTCAGCCTGCACGGCTTCTATGTGCGGGCCGCGAGCCCGATCGCGCGGATCGCCGCGC

CGCACGACGTGGCGGGCCTGCGGATCATCACCGCGTCGGGAACGAGCCAGGAGCGCATCCTGCTCGAATGGGACCGGCG

CAACGTCGCGCAGCGGCTCAAGCCGGTGGACGTGCTGTACTTCGACGACGACGCCGCATCGCGCGTCGCGCTGCTCTCG

GGGCGCGCCGACGCGGAGCTGAATCCGAACGCGACACTCGCGTACCAGGCGGCGCGCGACGGCAGGATTCGTTGCGTCG

GCAACGTGAACGCGGGCTGGCCGCTCAAGGCCGACGTCGCGATCGCGACGCGCAAGGGCAGCGGCCTCGCCGACGCGCT

CACGCTCGCGACCAACGGCCTGATCCGCAACGGCAAGTACCGGCAGGCGCTCGCGCGATGGGGGCTGCTGTCGGAGGCG

CTCGACCGGTCGGAGACGAATCCGGCGGGGCTGCCGTCGTTCTGA

>Bucl13_Bps_MSHR520.seq Reverse Complement DNA Sequence CP004369.seq(1,1185)

ATGTGTCGACGGCGTCGCGCAGTCGCGCGCGCTCGCATTGCGTCGCGCGGCGTCGATAGTGTCGATCATGTCGGAAGTG

GCGGAAGCATCAGAAGCATCAGAAGCATCAGAAGCATCAGAAGCATCAGAAGCATCAGAAGCATCAGAAGCATCAGAAG

CATCAGAAGCATCAGAAGCATCAGAGGTATCAGAGGCATCAGAGGCATTAGAAGCGTCAGCGGCATTAGAAGCATCGGA

GGCACCGAAGCGATGACCGACCGACGCACATTCGTCGGCGGCGCGGCGGCCTTCGCGGCCGCATGGGCCGCCGCGCCGT

TCGGCGCGCGCGCCGGCATCGCGGTCGATCTCGACCCGCGTCAGGCGGGCCGCGTGCGCGCCGAACGCGATCCGGCCGC

CGTCGACGCCGCGCGCCGCTACCGCTGGGTCGACGATCGCGCGTTCACCGTCGCGATCGCGCCGCATGCGCCACCCGTC

GCGACGTTCGCGACCGACGCGCGCACCGTCGTCGGCGCGGACCCGGACTACGCGCAACTCGTCGCCGACGCGCTCGGCC

GGCGGCTCGCGCTCGTGCCCGTCGCCTGGGCCGACTGGCCGCTCGGGCTGAGCGCCGGCAAGTACGACGCGGTGATCTC

GAACGTCGGCGTCACCGAGCAGCGCAAGCGGAAGTTCGATTTCACGACGTACCGGCTCGGCCTGCACGGCTTCTATGTG

CGGGCCGCGAGCCCGATCGCGCGGATCGCCGCGCCGCGCGACGTGGCGGGCCTGCGGATCATCACCGCGTCGGGAACGA

GCCAGGAGCGCATCCTGCTCGAATGGGACCGGCGCAACGTCGCGCAGCGGCTCAAGCCGGTGGACGTGCTGTACTTCGA

CGACGACGCCGCATCGCGCGTCGCGCTGCTCTCGGGGCGCGCCGACGCGGAGCTGAATCCGAACGCGACACTCGCGTAC

CAAGCGGCGCGCGACGGCAGGATTCGTTGCGTCGGCAACGTGAACGCGGGCTGGCCGCTCAAGGCCGACGTCGCGATCG

CGACGCGCAAGGGCAGCGGCCTCGCCGACGCGCTCACGCTCGCGACCAACGGCCTGATCCGCAACGGCAAGTACCGGCA

GGCGCTCGCGCGATGGGGGCTGCTGTCGGAGGCGCTCGACCGGTCGGAGACGAATCCGGCGGGGCTGCCGTCGTTCTGA

>Bucl13_Bps_NAU20B-16.seq >gi|568792780|gb|CP004004.1|:1079697-1080926 Burkholderia pseudomallei NAU20B-16 chromosome 2, complete sequence

ATGTGTCGACGGCGTCGCGCAGTCGCGCGCGCTCGCATTGCGTCGCGCGGCGTCGATAGTGTCGATCATGTCGGAAGTG

GCGGAAGTGGCGGAAGCATCAGAAGCATCAGGGGCATCAGGGGCATCAGAGGTATCAGAGGTATCAGAGGTATCAGAGG

TATCAGAGGTATCAGAGGTATCAGAGGTATCAGAGGTATCAGAGGCGTCAGAGGCATTAGAAGCGTCAGCGGCATTAGA

AACGTCAGCGGCATTAGAAACGTCAGCGGCATTAGAAGCATCGGAGGCACCGAAGCGATGACCGACCGACGCACATTCG

TCGGCGGCGCGGCGGCCTTCGCGGCCGCATGGGCCGCCGCGCCGTTCGGCGCGCGCGCCGGCATCGCGGTCGATCTCGA

CCCGCGTCAGGCGGGCCGCGTGCGCGCCGAACGCGATCCGGCCGTCGTCGACGCCACGCGCCGCTACCGCTGGGTCGAC

GATCGCGCGTTCACCGTCGCGATCGCGCCGCATGCGCCACCCGTCGCGACGTTCGCGACCGACGCGCGCACCGTCGTCG

GCGCGGACCCCGACTACGCGCAACTCGTCGCCGACGCGCTCAGCCGGCGGCTCGCGCTCGTGCCCGTCGCCTGGGCCGA

CTGGCCGCTCGGGCTGAGCGCCGGCAAGTACGACGCGGTGATCTCGAACGTCGGCGTCACCGAGCAGCGCAAGCGGAAG

TTCGATTTCACGACGTACCGGCTCAGCCTGCACGGCTTCTATGTGCGGGCCGCGAGCCCGATCGCGCGGATCGCCGCGC

CGCACGACGTGGCGGGCCTGCGGATCATCACCGCGTCGGGAACGAGCCAGGAGCGCATCCTGCTCGAATGGGACCGGCG

CAACGTCGCGCAGCGGCTCAAGCCGGTGGACGTGCTGTACTTCGACGACGACGCCGCATCGCGCGTCGCGCTGCTCTCG

GGGCGCGCCGACGCGGAGCTGAATCCGAACGCGACACTCGCGTACCAGGCGGCGCGCGACGGCAGGATTCGTTGCGTCG

GCAACGTGAACGCGGGCTGGCCGCTCAAGGCCGACGTCGCGATCGCGACGCGCAAGGGCAGCGGCCTCGCCGACGCGCT

CACGCTCGCGACCAACGGCCTGATCCGCAACGGCAAGTACCGGCAGGCGCTCGCGCGATGGGGGCTGCTGTCGGAGGCG

CTCGACCGGTCGGAGACGAATCCGGCGGGGCTGCCGTCGTTCTGA

>Bucl13_Bps_NCTC_13178.seq Reverse Complement DNA Sequence CP004002.seq(1,1185)

ATGTGTCGACGGCGTCGCGCAGTCGCGCGCGCTCGCATTGCGTCGCGCGGCGTCGATAGTGTCGATCATGTCGGAAGTG

GCGGAAGCATCAGAAGCATCAGGGGCATCAGGGGCATCAGGGGCATCAGGGGCATCAGGGGCATCAGGGGCATCAGGGG

CATCAGAGGTATCAGAGGTATCAGAGGTATCAGAGGCGTCAGAGGCATTAGAAGCGTCAGCGGCATTAGAAGCATCGGA

GGCACCGAAGCGATGACCGACCGACGCACATTCGTCGGCGGCGCGGCGGCCTTCGCAGCCGCATGGGCCGCCGCGCCGT

TCGGCGCGCGCGCCGGCATCGCGGTCGATCTCGACCCGCGTCAGGCGGGCCGCGTGCGCGCCGAACGCCATCCGGCCGC

CGTCGACGCCGCGCGCCGCTACCGCTGGGTCGACGATCGCGCGTTCACCGTCGCGATCGCGCCGCATGCGCCACCCGTC

GCGACGTTCGCGACCGACGCGCGCACCGTCGTCGGCGCGGACCCGGACTACGCGCAACTCGTCGCCGACGCGCTCGGCC

GGCGGCTCGCGCTCGTGCCCGTCGCCTGGGCCGACTGGCCGCTCGGGCTGAGCGCCGGCAAGTACGACGCGGTGATCTC

GAACGTCGGCGTCACCGAGCAGCGCAAGCGGAAGTTCGATTTCACGACGTACCGGCTCGGCCTGCACGGCTTCTATGTG

CGGGCCGCGAGCCCGATCGCGCGGATCGCCGCGCCGCGCGACGTGGCGGGCCTGCGGATCATCACCGCGTCGGGAACGA

GCCAGGAGCGCATCCTGCTCGAATGGGACCGGCGCAACGTCGCGCAGCGGCTCAAGCCGGTGGACGTGCTGTACTTCGA

CGACGACGCCGCATCGCGCGTCGCGCTGCTCTCGGGGCGCGCCGACGCGGAGCTGAATCCGAACGCGACACTCGCGTAC

CAGGCGGCGCGCGACGGCAGGATTCGTTGCGTCGGCAACGTGAACGCGGGCTGGCCGCTCAAGGCCGACGTCGCGATCG

CGACGCGCAAGGGCAGCGGCCTCGCCGACGCGCTCACGCTCGCGACCAACGGCCTGATCCGCAACGGCAAGTACCGGCA

GGCGCTCGCGCGATGGGGGCTGCTGTCGGAGGCGCTCGACCGGTCGGAGACGAATCCGGCGGGGCTGCCGTCGTTCTGA

>A3N630_BURP6_Bucl14_Bps_668.seq Reverse Complement DNA Sequence Untitled Seq #18(1,576)

ATGCGGGGGACGCGCGTCTGGCGTTCTTGGTGTGGTTGGTGTGGTTGGTGTGGTTGGTGTGGTTGGTGTGGTCGGTGTG

GTCGGTGTGGTCGGTGTGGTCGGTGTGGTCGGTGTGGTTGGTGTGGTTGGTGTGGTCGGTGTGGTCGGTGTGGTCGGTG

TGGTCGGTGTGGTCGGTGTGGTCGGTGTGGTCGGTGTGGTCGGTGTGGTCGGTGTGGTCGGTGTGGTCGGTGTGGTCGG

TGTGGTCGGTGTGGTCGGTGTGGTCGGTGTGGTCGGTGTGGTCGGTGTGGTCGGTGTGGTCGGTGTGGTCGGTGTGGTC

GGCGTGGTCGGCGTGGTCGGCGTGGTCGGCGTGGTCGGCGTGGTCGGGCGTGGTCGGCACATCTGTCGCCGCGAACCCC

GCGGGCGTTTCCATCCGGACGTCCGGCCAAACGCCGCGCTCTTCCCCGGGCCCTCAGCCATCCCCCGGCGCGGCGGCCA

TGCCGTTCATGCGACGCGCGTCCCGTCTTCGCGTCGACGCGCGCCTCGCGCCGATCGACACGGCGGCCATACGCGCCGT

CGGTCAGCCCGTCGTCGGCATGA

>Bucl14_Bps_1026b.seq Created: Monday, December 30, 2013 5:09 PM

atgcgggggacgcgcgtctggcgttcttggtgtggtcggcgtggtcggcgtggtcggcacatctgtcgccgcgaacccc

gcgggcgtttccatccggacgtccggccaaacgccgcgctcttccccgggccctcagccatcccccggcgcggcggcca

tgccgttcatgcgacgcgcgtcccgtcttcgcgtcgacgcgcgcctcgcgccgatcgacacggcggccatacgcgccgt

cggtcagcccgtcgtcggcatga

>Bucl14_Bps_1106a.seq Reverse Complement DNA Sequence Untitled Seq #9(1,296)

atgcgggggacgcgcgtctggcgttcttggtgtggttggtgtggttggtgtggttggtgtggttggtgtggttggtgtg

gtcggcgtggtcggcacatctgtcgccgcgaaccccgcgggcgtttccatccggacgtccggccaaacgccgcgctctt

ccccgggccctcagccatcccccggcgcggcggccatgccgttcatgcgacgcgcgtcccgtcttcgcgtcgacgcgcg

cctcgcgccgatcgacacggcggccatacgcgccgtcggtcagcccgtcgtcggcatga

>Bucl14_Bps_BPC006.seq Reverse Complement DNA Sequence Untitled Seq #43(1,287)

atgcgggggacgcgcgtctggcgttcttggtgtggttggtgtggttggtgtggttggtgtggttggtgtggtcggcgtg

gtcggcacatctgtcgccgcgaaccccgcgggcgtttccatccggacgtccggccaaacgccgcgctcttccccgggcc

ctcagccatcccccggcgcggcggccatgccgttcatgcgacgcgcgtcccgtcttcgcgtcgacgcgcgcctcgcgcc

gatcgacacggcggccatacgcgccgtcggtcagcccgtcgtcggcatga

>Bucl14_Bps_MSHR_305.seq Created: Thursday, January 02, 2014 12:39 PM

atgcgggggacgcgcgtctggcgttcttggtgtggtcggcgtggtcggcacatctgtcgccgcgaaccccgcgggcgtt

tccatccggacgtccggccaaacgccgcgctcttccccgggccctcagccatcccccggcgcggcggccatgccgttca

tgcgacgcgcgtcccgtcttcgcgtcgacgcgcgcctcgcaccgatcgacacggcggccatacgcgccgtcggtcagcc

cgtcgtcggcatga

>Bucl14_Bps_NCTC_13179.seq Created: Friday, January 03, 2014 11:03 AM

atgcgggggacgcgcgtctggcgttcttggtgtggtcggtgtggtcggcgtggtcggcacatctgtcgccgcgaacccc

gcgggcgtttccatccggacgtccggccaaacgccgcgctcttccccgggccctcagccatcccccggcgcggcggcca

tgccgttcatgcgacgcgcgtcccgtcttcgcgtcgacgcgcgcctcgcaccgatcgacacggcggccatacgcgccgt

cggtcagcccgtcgtcggcatga

>Bucl14_Bm_ATCC_23344.seq Created: Friday, January 03, 2014 1:57 PM

atgcgggggacgcgcgtctggcgttcttggtgtggtcggtgtggtcggcgtggtcggcgtggtcggcgtggtcggcaca

tctgtcgccgcgaaccccgcgggcgtttccatccggacgtccggccaaacgccgcgctcttccccgggccctcagccat

cccccggcgcggcggccatgccgttcatgcgacgcgcgtcccgtcttcgcgtcgacgcgcgcctcgcgccgatcgacac

ggcggccatacgcgccgtcggtcagcccgtcgtcggcatga

>Bucl14_Bm_NCTC_10229.seq Reverse Complement DNA Sequence Untitled Seq #48(1,296)

atgcgggggacgcgcgtctggcgttcttggtgtggtcggtgtggtcggtgtggtcggtgtggtcggcgtggtcggcgtg

gtcggcgtggtcggcacatctgtcgccgcgaaccccgcgggcgtttccatccggacgtccggccaaacgccgcgctctt

ccccgggccctcagccatcccccggcgcggcggccatgccgttcatgcgacgcgcgtcccgtcttcgcgtcgacgcgcg

cctcgcgccgatcgacacggcggccatacgcgccgtcggtcagcccgtcgtcggcatga

>Bucl14_Bm_NCTC_10247.seq Created: Friday, January 03, 2014 6:13 PM

atgcgggggacgcgcgtctggcgttcttggtgtggtcggtgtggtcggtgtggtcggcgtggtcggcgtggtcggcgtg

gtcggcgtggtcggcacatctgtcgccgcgaaccccgcgggcgtttccatccggacgtccggccaaacgccgcgctctt

ccccgggccctcagccatcccccggcgcggcggccatgccgttcatgcgacgcgcgtcccgtcttcgcgtcgacgcgcg

cctcgcgccgatcgacacggcggccatacgcgccgtcggtcagcccgtcgtcggcatga

>Bucl14_Bm_SAVP1.seq Created: Saturday, January 04, 2014 1:18 PM

atgcgggggacgcgcgtctggcgttcttggtgtggtcggtgtggtcggcgtggtcggcgtggtcggcgtggtcggcaca

tctgtcgccgcgaaccccgcgggcgtttccatccggacgtccggccaaacgccgcgctcttccccgggccctcagccat

cccccggcgcggcggccatgccgttcatgcgacgcgcgtcccgtcttcgcgtcgacgcgcgcctcgcgccgatcgacac

ggcggccatacgcgccgtcggtcagcccgtcgtcggcatga

>Bucl14_Bm_2000031281.seq Reverse Complement DNA Sequence AWGQ01000095.seq(1,278)

ATGCGGGGGACGCGCGTCTGGCGTTCTTGGTGTGGTCGGTGTGGTCGGCGTGGTCGGCGTGGTCGGCGTGGTCGGCACA

TCTGTCGCCGCGAACCCCGCGGGCGTTTCCATCCGGACGTCCGGCCAAACGCCGCGCTCTTCCCCGGGCCCTCAGCCAT

CCCCCGGCGCGGCGGCCATGCCGTTCATGCGACGCGCGTCCCGTCTTCGCGTCGACGCGCGCCTCGCGCCGATCGACAC

GGCGGCCATACGCGCCGTCGGTCAGCCCGTCGTCGGCATGA

>Bucl14_Bm_2002721280.seq >gi|99033628|gb|AANX02000041.1|:11547-11833 Burkholderia mallei 2002721280 ctg2_1099945121895, whole genome shotgun sequence

ATGCGGGGGACGCGCGTCTGGCGTTCTTGGTGTGGTCGGTGTGGTCGGTGTGGTCGGCGTGGTCGGCGTGGTCGGCGTG

GTCGGCACATCTGTCGCCGCGAACCCCGCGGGCGTTTCCATCCGGACGTCCGGCCAAACGCCGCGCTCTTCCCCGGGCC

CTCAGCCATCCCCCGGCGCGGCGGCCATGCCGTTCATGCGACGCGCGTCCCGTCTTCGCGTCGACGCGCGCCTCGCGCC

GATCGACACGGCGGCCATACGCGCCGTCGGTCAGCCCGTCGTCGGCATGA

>Bucl14_Bm_A188.seq Reverse Complement DNA Sequence AWGR01000142.seq(1,287)

ATGCGGGGGACGCGCGTCTGGCGTTCTTGGTGTGGTCGGTGTGGTCGGTGTGGTCGGCGTGGTCGGCGTGGTCGGCGTG

GTCGGCACATCTGTCGCCGCGAACCCCGCGGGCGTTTCCATCCGGACGTCCGGCCAAACGCCGCGCTCTTCCCCGGGCC

CTCAGCCATCCCCCGGCGCGGCGGCCATGCCGTTCATGCGACGCGCGTCCCGTCTTCGCGTCGACGCGCGCCTCGCGCC

GATCGACACGGCGGCCATACGCGCCGTCGGTCAGCCCGTCGTCGGCATGA

>Bucl14_Bm_A193.seq >gi|577031902|gb|AWGS01000011.1|:62379-62663 Burkholderia mallei A193 Burkholderia-mallei-MCB-015_545, whole genome shotgun sequence

ATGCGGGGGACGCGCGTCTGGCGTTCTTGGTGTGGTCGGTGTGGTCGGTGTGGTCGGCGTGGTCGGCGTGGTCGGCGTG

GTCGGCACATCTGTCGCCGCGAACCCCGCGGGCGTTTCCATCCGGACGTCCGGCCAAACGCCGCGCTCTTCCCCGGGCC

CTCAGCCATCCCCCGGCGCGGCGGCCATGCCGTTCATGCGACGCGCGTCCCGTCTTCGCGTCGACGCGCGCCTCGCGCC

GATCGACACGGCGGCCATACGCGCCGTCGGTCAGCCCGTCGTCGGCATGA

>Bucl14_Bm_ATCC_10399.seq >gi|99031371|gb|AAHN02000044.1|:11517-11794 Burkholderia mallei ATCC 10399 ctg_1047276662049, whole genome shotgun sequence

ATGCGGGGGACGCGCGTCTGGCGTTCTTGGTGTGGTCGGTGTGGTCGGCGTGGTCGGCGTGGTCGGCGTGGTCGGCACA

TCTGTCGCCGCGAACCCCGCGGGCGTTTCCATCCGGACGTCCGGCCAAACGCCGCGCTCTTCCCCGGGCCCTCAGCCAT

CCCCCGGCGCGGCGGCCATGCCGTTCATGCGACGCGCGTCCCGTCTTCGCGTCGACGCGCGCCTCGCGCCGATCGACAC

GGCGGCCATACGCGCCGTCGGTCAGCCCGTCGTCGGCATGA

>Bucl14_Bm_China_7.seq >gi|577031103|gb|AWGN01000094.1|:62373-62650 Burkholderia mallei China_7 Burkholderia-mallei-MCB-010_1376, whole genome shotgun sequence

ATGCGGGGGACGCGCGTCTGGCGTTCTTGGTGTGGTCGGTGTGGTCGGCGTGGTCGGCGTGGTCGGCGTGGTCGGCACA

TCTGTCGCCGCGAACCCCGCGGGCGTTTCCATCCGGACGTCCGGCCAAACGCCGCGCTCTTCCCCGGGCCCTCAGCCAT

CCCCCGGCGCGGCGGCCATGCCGTTCATGCGACGCGCGTCCCGTCTTCGCGTCGACGCGCGCCTCGCGCCGATCGACAC

GGCGGCCATACGCGCCGTCGGTCAGCCCGTCGTCGGCATGA

>Bucl14_Bm_FMH.seq Reverse Complement DNA Sequence AAIQ02000042.seq(1,278)

ATGCGGGGGACGCGCGTCTGGCGTTCTTGGTGTGGTCGGTGTGGTCGGCGTGGTCGGCGTGGTCGGCGTGGTCGGCACA

TCTGTCGCCGCGAACCCCGCGGGCGTTTCCATCCGGACGTCCGGCCAAACGCCGCGCTCTTCCCCGGGCCCTCAGCCAT

CCCCCGGCGCGGCGGCCATGCCGTTCATGCGACGCGCGTCCCGTCTTCGCGTCGACGCGCGCCTCGCGCCGATCGACAC

GGCGGCCATACGCGCCGTCGGTCAGCCCGTCGTCGGCATGA

>Bucl14_Bm_GB8_horse_4.seq Reverse Complement DNA Sequence AAHO01000037.seq(1,278)

ATGCGGGGGACGCGCGTCTGGCGTTCTTGGTGTGGTCGGTGTGGTCGGCGTGGTCGGCGTGGTCGGCGTGGTCGGCACA

TCTGTCGCCGCGAACCCCGCGGGCGTTTCCATCCGGACGTCCGGCCAAACGCCGCGCTCTTCCCCGGGCCCTCAGCCAT

CCCCCGGCGCGGCGGCCATGCCGTTCATGCGACGCGCGTCCCGTCTTCGCGTCGACGCGCGCCTCGCGCCGATCGACAC

GGCGGCCATACGCGCCGTCGGTCAGCCCGTCGTCGGCATGA

>Bucl14_Bm_JHU.seq Reverse Complement DNA Sequence Untitled Seq #11(1,278)

ATGCGGGGGACGCGCGTCTGGCGTTCTTGGTGTGGTCGGTGTGGTSGGCGTGGTCGGCGTGGTCGGCGTGGTCGGCACA

TCTGTCGCCGCGAACCCCGCGGGCGTTTCCATCCGGACGTCCGGCCAAACGCCGCGCTCTTCCCCGGGCCCTCAGCCAT

CCCCCGGCGCGGCGGCCATGCCGTTCATGCGACGCGCGTCCCGTCTTCGCGTCGACGCGCGCCTCGCGCCGATCGACAC

GGCGGCCATACGCGCCGTCGGTCAGCCCGTCGTCGGCATGA

>Bucl14_Bm_PRL-20.seq >gi|145666995|gb|AAZP01000138.1|:9206-9483 Burkholderia mallei PRL-20 gcontig_1105338610854, whole genome shotgun sequence

ATGCGGGGGACGCGCGTCTGGCGTTCTTGGTGTGGTCGGTGTGGTCGGCGTGGTCGGCGTGGTCGGCGTGGTCGGCACA

TCTGTCGCCGCGAACCCCGCGGGCGTTTCCATCCGGACGTCCGGCCAAACGCCGCGCTCTTCCCCGGGCCCTCAGCCAT

CCCCCGGCGCGGCGGCCATGCCGTTCATGCGACGCGCGTCCCGTCTTCGCGTCGACGCGCGCCTCGCGCCGATCGACAC

GGCGGCCATACGCGCCGTCGGTCAGCCCGTCGTCGGCATGA

>Bucl14_Bm_strain_6.seq Reverse Complement DNA Sequence AWGO01000094.seq(1,305)

ATGCGGGGGACGCGCGTCTGGCGTTCTTGGTGTGGTCGGTGTGGTCGGTGTGGTCGGCGTGGTCGGCGTGGTCGGCGTG

GTCGGCGTGGTCGGCGTGGTCGGCACATCTGTCGCCGCGAACCCCGCGGGCGTTTCCATCCGGACGTCCGGCCAAACGC

CGCGCTCTTCCCCGGGCCCTCAGCCATCCCCCGGCGCGGCGGCCATGCCGTTCATGCGACGCGCGTCCCGTCTTCGCGT

CGACGCGCGCCTCGCGCCGATCGACACGGCGGCCATACGCGCCGTCGGTCAGCCCGTCGTCGGCATGA

>Bucl14_Bm_strain_11.seq Reverse Complement DNA Sequence AWGP01000139.seq(1,296)

ATGCGGGGGACGCGCGTCTGGCGTTCTTGGTGTGGTCGGTGTGGTCGGTGTGGTCGGCGTGGTCGGCGTGGTCGGCGTG

GTCGGCGTGGTCGGCACATCTGTCGCCGCGAACCCCGCGGGCGTTTCCATCCGGACGTCCGGCCAAACGCCGCGCTCTT

CCCCGGGCCCTCAGCCATCCCCCGGCGCGGCGGCCATGCCGTTCATGCGACGCGCGTCCCGTCTTCGCGTCGACGCGCG

CCTCGCGCCGATCGACACGGCGGCCATACGCGCCGTCGGTCAGCCCGTCGTCGGCATGA

>Bucl14_Bps_MSHR146.seq >gi|575433883|gb|CP004042.1|:3173614-3173918 Burkholderia pseudomallei MSHR146 chromosome 1, complete sequence

ATGCGGGGGACGCGCGTCTGGCGTTCTTGGTGTGGTTGGTGTGGTTGGTGTGGTTGGTGTGGTTGGTGTGGTTGGTGTG

GTTGGTGTGGTTGGTGTGGTCGGCACATCTGTCGCCGCGAACCCCGCGGGCGTTTCCATCCGGACGTCCGGCCAAACGC

CGCGCTCTTCCCCGGGCCCTCAGCCATCCCCCGGCGCGGCGGCCATGCCGTTCATGCGACGCGCGTCCCGTCTTCGCGT

CGACGCGCGCCTCGCGCCGATCGACACGGCGGCCATACGCGCCGTCGGTCAGCCCGTCGTCGGCATGA

>Bucl14_Bps_MSHR511.seq >gi|574600368|gb|CP004023.1|:3028369-3028673 Burkholderia pseudomallei MSHR511 chromosome 1, complete sequence

ATGCGGGGGACGCGCGTCTGGCGTTCTTGGTGTGGTTGGTGTGGTTGGTGTGGTTGGTGTGGTTGGTGTGGTTGGTGTG

GTTGGTGTGGTTGGTGTGGTCGGCACATCTGTCGCCGCGAACCCCGCGGGCGTTTCCATCCGGACGTCCGGCCAAACGC

CGCGCTCTTCCCCGGGCCCTCAGCCATCCCCCGGCGCGGCGGCCATGCCGTTCATGCGACGCGCGTCCCGTCTTCGCGT

CGACGCGCGCCTCGCGCCGATCGACACGGCGGCCATACGCGCCGTCGGTCAGCCCGTCGTCGGCATGA

>Bucl14_Bps_MSHR520.seq >gi|589299693|gb|CP004368.1|:3573295-3573545 Burkholderia pseudomallei MSHR520 chromosome 1, complete sequence

ATGCGGGGGACGCGCGTCTGGCGTTCTTGGTGTGGTCGGCGTGGTCGGCACATCTGTCGCCGCGAACCCCGCGGGCGTT

TCCATCCGGACGTCCGGCCAAACGCCGCGCTCTTCCCCGGGCCCTCAGCCATCCCCCGGCGCGGCGGCCATGCCGTTCA

TGCGACGCGCGTCCCGTCTTCGCGTCGACGCGCGCCTCGCACCGATCGACACGGCGGCCATACGCGCCGTCGGTCAGCC

CGTCGTCGGCATGA

>Bucl14_Bps_NAU20B-16.seq >gi|568789317|gb|CP004003.1|:771035-771339 Burkholderia pseudomallei NAU20B-16 chromosome 1, complete sequence

ATGCGGGGGACGCGCGTCTGGCGTTCTTGGTGTGGTTGGTGTGGTTGGTGTGGTTGGTGTGGTTGGTGTGGTTGGTGTG

GTTGGTGTGGTTGGTGTGGTCGGCACATCTGTCGCCGCGAACCCCGCGGGCGTTTCCATCCGGACGTCCGGCCAAACGC

CGCGCTCTTCCCCGGGCCCTCAGCCATCCCCCGGCGCGGCGGCCATGCCGTTCATGCGACGCGCGTCCCGTCTTCGCGT

CGACGCGCGCCTCGCGCCGATCGACACGGCGGCCATACGCGCCGTCGGTCAGCCCGTCGTCGGCATGA

>Bucl14_Bps_NCTC_13178.seq >gi|568795288|gb|CP004001.1|:2425477-2425736 Burkholderia pseudomallei NCTC 13178 chromosome 1, complete sequence

ATGCGGGGGACGCGCGTCTGGCGTTCTTGGTGTGATTGGTGTGGTTGGCGTGGTCGGCACGTCTGTCGCCGCGAACCCC

GCGGGCGTTTCCATCCGGACGTCCGGCCAAACGCCGCGCTCTTCCCCGGGCCCTCAGCCATCCCCCGGCGCGGCGGCCA

TGCCGTTCATGCGACGCGCGTCCCGTCTTCGCGTCGACGCGCGCCTCGCGCCGATCGACACGGCGGCCATACGCGCCGT

CGGTCAGCCCGTCGTCGGCATGA

>Bucl14_Bps_1710b.seq Reverse Complement DNA Sequence Untitled Seq #47(1,274)

ACGCGGGGGACGCGCGTCTGGCGTTCTTGGTGTGGTTGGTGTGGTCGGCGTGGTCGGCACATCTGTCGCCGCGAACCCC

GCGGGCGTTTCCATCCGGACGTCCGGCCAAACGCCGCGCTCTTCCCCGGGCCCTCAGCCATCCCCCGGCGCGGCGGCCA

TGCCGTTCATGCGACGCGCGTCCCGTCTTCGCGTCGACGCGCGCCTCGCGCCGATCGACACGGCGGCCATACGCGCCGT

CGGTCAGCCCGTCGTCGGCATGA

>Bucl14_Bps_K96243.seq Reverse Complement DNA Sequence Untitled Seq #52(1,274)

ATGCGGGGGACGCGCGTCTGGCGTTCTTGGTGTGGTCGGTGTGGTCGGCGTGGTCGGCACATCTGTCGCCGCGAACCCC

GCGGGCGTTTCCATCCGGACGTCCGGCCAAACGCCGCGCTCTTCCCCGGGCCCTCAGCCATCCCCCGGCGCGGCGGCCA

TGCCGTTCATGCGACGCGCGTCCCGTCTTCGCGTCGACGCGCGCCTCGCACCGATCGACACGGCGGCCATACGCGCCGT

CGGTCAGCCCGTCGTCGGCATGA

>Bucl14_Bps_MSHR_346.seq Reverse Complement DNA Sequence Untitled Seq #57(1,274)

ATGCGGGGGACGCGCGTCTGGCGTTCTTGGTGTGGTTGGTGTGGTCGGCACATCTGTCGCCGCGAACCCCGCGGGCGTT

TCCATCCGGACGTCCGGCCAAACGCCGCGCTCTTCCCCGGGCCCTCAGCCATCCCCCGGCGCGGCGGCCATGCCGTTCA

TGCGACGCGCGTCCCGTCTTCGCGTCGACGCGCGCCTCGCGCCGATCGACACGGCGGCCATACGCGCCGTCGATCAGCC

CGTCGTCGGCATGA

>Bucl15_Bps_K96243.seq

gtggctgtgcccacgaggggtttttttgcgtgcggcggcgcgccgggcgttttcggcgcggccacgcacggacgcacgg

atgcacggatgcacggatgcgcgatcgctcgacgcgcccggcgtgctcggtgcgcttgggatgctcggcgcgaccggcg

cgaccggtgcgattggcgcggcgcgccggcggttttag

>Bucl15_Bps_MSHR_305.seq

gtggctgcgctcacgaggggttttttgcgtgcggcggcgcgccgggcgttttcggcgcggccgcgcacggaagcacgga

tgcacggatgcgcgatcgctcgacgcgcccggcgtgctcggtgcgcttgggatgctcggcgcgaccggcgcgaccagcg

cgattggcgcggcacgccggcggttttag

>Bucl15_Bps_1106a.seq

gtggctgcgctcacgaggggttttttgcgtgcggcggcgcgccgggcgttttcggcgcggccgcgcacggacgcacgga

tgcgcgatcgctcgacgcgcccggcgtgctcggtgtgcttgggatgctcggggcgaccggcgcgattggcgcggcacgc

cggcggttttag

>Bucl15_Bps_1026b.seq

gtggctgtgcccacgaggggtttttttgcgtgcggcggcgcgccgggcgttttcggcgcggccacgcacggacgcacgg

atgcacggatgcacggatgcgcgatcgctcgacgcgcccggcgtgctcggtgcgcttgggatgctcggcgcgaccggcg

cgaccggtgcgattggcgcggcgcgccggcggttttag

>Bucl15_Bps_1710b.seq

gtggctgcgctcacgaggggttttttgcgtgcggcggcgcgccgggcgttttcgacgcggccgcgcacggacgcacgga

cgcacggatgcgcgatcgctcgacgcgcccggcgtgctcggtgcgcttgggatgctcggcccgaccggcgcggccggtg

cgattggcgcggcacgccggcggttttag

>Bucl15_Bps_BPC006.seq

gtggctgcgctcacgaggggttttttgcgtgcggcggcgcgccgggcgttttcggcgcggccgcgcacggacgcacgga

tgcgcgatcgctcgacgcgcccggcgtgctcggtgtgcttgggatgctcggggcgaccggcgcgattggcgcggcacgc

cggcggttttag

>A3NDA8_BURP6_Bucl15_Bps_668.seq

GTGGCTGCGCCCACGAGAGGTTTTTTTGCGTGCGGCGGCGCGCCGGGCGTTTTCGGCGCGGCCGCGCACGGAGGCACGG

ATGCGCGATCGCTCGACGCGCCCGGCGTGCTCGGTGTGCTTGGGATGCTCGGGGCGACCGGCGTGACCGCCCCGACCGG

CGCGACCGGCGCGACCGGCGCGATTGGCGCGATTGGCGCGATTGGCGCGATTGGCGCGATTGGCGCGATTGGCGCGGCG

CGCCGGCGGTTTTAG

>Bucl15_Bps_MSHR146.seq

GTGGCTGCGCTCACGAGGGGTTTTTTGCGTGCGGCGGCGCGCCGGGCGTTTTCGGCGCGGCCGCGCACGGACGCACGGA

CGCACGGACGCACGGACGCACGGACGCACGGACGCACGGACGCACGGACGCACGGACGCACGGATGCACGGACGCACGG

ATGCGCGATCGCTCGACGCGCCCGGCGTGCTCGGTGCGCTTGGGATGCTCGGCGCGACCGGTGCGATTGGCGCGGCGCG

CCGGCGGTTTTAG

>Bucl15_Bps_MSHR511.seq

GTGGCTGCGCTCACGAGGGGTTTTTTGCGTGCGGCGGCGCGCCGGGCGTTTTCGGCGCGGCCGCGCACGGACGCACGGA

CGCACGGACGCACGGACGCACGGACGCACGGACGCACGGACGCACGGACGCACGGACGCACGGACGCACGGACGCACGG

ACGCACGGATGCACGGACGCACGGATGCGCGATCGCTCGACGCGCCCGGCGTGCTCGGTGCGCTTGGGATGCTCGGCGC

GACCGGTGCGATTGGCGCGGCGCGCCGGCGGTTTTAG

>Bucl15_Bps_MSHR520.seq

GTGGCTGCGCTCACGAGGGGTTTTTTGCGTGCGGCGGCGCGCCGGGCGTTTTCGGCGCGGCCGCGCACGGAAGCACGGA

TGCACGGATGCGCGATCGCTCGACGCGCCCGGCGTGCTCGGTGCGCTTGGGATGCTCGGCGCGACCGGCGCGACCAGCG

CGATTGGCGCGGCACGCCGGCGGTTTTAG

>Bucl15_Bps_NAU20B-16.seq

GTGGCTGCGCTCACGAGGGGTTTTTTGCGTGCGGCGGCGCGCCGGGCGTTTTCGGCGCGGCCGCGCACGGACGCACGGA

CGCACGGACGCACGGACGCACGGACGCACGGACGCACGGACGCACGGACGCACGGACGCACGGACGCACGGACGCACGG

ATGCACGGACGCACGGATGCGCGATCGCTCGACGCGCCCGGCGTGCTCGGTGCGCTTGGGATGCTCGGCGCGACCGGTG

CGATTGGCGCGGCGCGCCGGCGGTTTTAG

>Bucl15_Bps_NCTC_13178.seq

GTGGCTGCGCCCACGAGGGGTTTTTTTGCGTGCGGCGGCGCGCCGGGCGTTTTCGGCGCGGCCGCGCACGGAAGCACGG

AAGCACGGAAGCACGGAAGCACGGAAGCACGGAAGCACGGAAGCACGGAAGCACGGAAGCACGGAAGCACGGAGGCACG

GAGGCACGGAGGCACGGAGGCGCGATCGCTCGACGCGCCCGACGTGCTCGGTGCGCTTGGGATGCTCGGCCCGACCGGC

GCGGCCGGTGCGATTGGCGCGGCACGCCGGCGGTTTTAG

>Bucl15_Bm_2000031281.seq

GTGGCTGTGCCCACGAGGGGTTTTTTTGCGTGCGGCGGCGCGCCGGGCGTTTTCGGCGCGGCCACGCACGGACGCATGG

ACGCACGGATGCACGGATGCACGGATGCGCGATCGCTCGACGCGCCCGGCGTGCTCGGTGCGCTTGGGATGCTCGGAGC

GACCGGCGCGACCGGTGCGACCGGTGCGATTGGCGCGGCGCGCCGGCGGTTTTAG

>Bucl15_Bm_2002721280.seq

GTGGCTGTGCCCACGAGGGGTTTTTTTGCGTGCGGCGGCGCGCCGGGCGTTTTCGGCGCGGCCACGCACGGACGCATGG

ACGCACGGATGCACGGATGCACGGATGCGCGATCGCTCGACGCGCCCGGCGTGCTCGGTGCGCTTGGGATGCTCGGAGC

GACCGGCGCGACCGGTGCGACCGGTGCGACCGGTGCGATTGGCGCGGCGCGCCGGCGGTTTTAG

>Bucl15_Bm_A188.seq

GTGGCTGTGCCCACGAGGGGTTTTTTTGCGTGCGGCGGCGCGCCGGGCGTTTTCGGCGCGGCCACGCACGGACGCATGG

ACGCACGGATGCACGGATGCACGGATGCGCGATCGCTCGACGCGCCCGGCGTGCTCGGTGCGCTTGGGATGCTCGGAGC

GACCGGCGCGACCGGTGCGACCGGTGCGATTGGCGCGGCGCGCCGGCGGTTTTAG

>Bucl15_Bm_A193.seq

GTGGCTGTGCCCACGAGGGGTTTTTTTGCGTGCGGCGGCGCGCCGGGCGTTTTCGGCGCGGCCACGCACGGACGCATGG

ACGCACGGATGCACGGATGCACGGATGCGCGATCGCTCGACGCGCCCGGCGTGCTCGGTGCGCTTGGGATGCTCGGAGC

GACCGGCGCGACCGGTGCGACCGGTGCGACCGGTGCGATTGGCGCGGCGCGCCGGCGGTTTTAG

>Bucl15_Bm_China_7.seq

GTGGCTGTGCCCACGAGGGGTTTTTTTGCGTGCGGCGGCGCGCCGGGCGTTTTCGGCGCGGCCACGCACGGACGCATGG

ACGCACGGATGCACGGATGCACGGATGCGCGATCGCTCGACGCGCCCGGCGTGCTCGGTGCGCTTGGGATGCTCGGAGC

GACCGGCGCGACCGGTGCGACCGGTGCGATTGGCGCGGCGCGCCGGCGGTTTTAG

>Bucl15_Bm_PRL-20.seq

GTGGCTGTGCCCACGAGGGGTTTTTTTGCGTGCGGCGGCGCGCCGGGCGTTTTCGGCGCGGCCACGCACGGACGCATGG

ACGCACGGATGCACGGATGCACGGATGCGCGATCGCTCGACGCGCCCGGCGTGCTCGGTGCGCTTGGGATGCTCGGAGC

GACCGGCGCGACCGGCGCGACCGGTGCGACCGGTGCGATTGGCGCGGCGCGCCGGCGGTTTTAG

>Bucl15_Bm_strain_6.seq

GTGGCTGTGCCCACGAGGGGTTTTTTTGCGTGCGGCGGCGCGCCGGGCGTTTTCGGCGCGGCCACGCACGGACGCATGG

ACGCACGGATGCACGGATGCACGGATGCACGGATGCGCGATCGCTCGACGCGCCCGGCGTGCTCGGTGCGCTTGGGATG

CTCGGAGCGACCGGCGCGACCGGCGCGACCGGTGCGATTGGCGCGGCGCGCCGGCGGTTTTAG

>Bucl15_Bm_strain_11.seq

GTGGCTGTGCCCACGAGGGGTTTTTTTGCGTGCGGCGGCGCGCCGGGCGTTTTCGGCGCGGCCACGCACGGACGCATGG

ACGCACGGATGCACGGATGCACGGATGCGCGATCGCTCGACGCGCCCGGCGTGCTCGGTGCGCTTGGGATGCTCGGAGC

GACCGGCGCGACCGGTGCGACCGGTGCGACCGGTGCGATTGGCGCGGCGCGCCGGCGGTTTTAG

>Bucl15_Bm_NCTC_10229.seq

gtggctgtgcccacgaggggtttttttgcgtgcggcggcgcgccgggcgttttcggcgcggccacgcacggacgcatgg

acgcacggatgcacggatgcacggatgcgcgatcgctcgacgcgcccggcgtgctcggtgcgcttgggatgctcggagc

gaccggcgcgaccggtgcgaccggtgcgaccggtgcgattggcgcggcgcgccggcggttttag

>Bucl15_Bm_NCTC_10247.seq

GTGGCTGTGCCCACGAGGGGTTTTTTTGCGTGCGGCGGCGCGCCGGGCGTTTTCGGCGCGGCCACGCACGGACGCATGG

ACGCACGGATGCACGGATGCACGGATGCGCGATCGCTCGACGCGCCCGGCGTGCTCGGTGCGCTTGGGATGCTCGGAGC

GACCGGCGCGACCGGTGCGACCGGTGCGACCGGTGCGATTGGCGCGGCGCGCCGGCGGTTTTAG

>Bucl15_Bm_JHU.seq

GTGGCTGTGCCCACGAGGGGTTTTTTTGCGTGCGGCGGCGCGCCGGGCGTTTTCGGCGCGGCCACGCACGGACGCATGG

ACGCACGGATGCACGGATGCACGGATGCGCGATCGCTCGACGCGCCCGGCGTGCTCGGTGCGCTTGGGATGCTCGGAGC

GACCGGCGCGACCGGTGCGACCGGTGCGATTGGCGCGGCGCGCCGGCGGTTTTAG

>Bucl15_Bm_FMH.seq

GTGGCTGTGCCCACGAGGGGTTTTTTTGCGTGCGGCGGCGCGCCGGGCGTTTTCGGCGCGGCCACGCACGGACGCATGG

ACGCACGGATGCACGGATGCACGGATGCGCGATCGCTCGACGCGCCCGGCGTGCTCGGTGCGCTTGGGATGCTCGGAGC

GACCGGCGCGACCGGTGCGACCGGTGCGATTGGCGCGGCGCGCCGGCGGTTTTAG

>Bucl15_Bm_ATCC_10399.seq

GTGGCTGTGCCCACGAGGGGTTTTTTTGCGTGCGGCGGCGCGCCGGGCGTTTTCGGCGCGGCCACGCACGGACGCATGG

ACGCACGGATGCACGGATGCACGGATGCGCGATCGCTCGACGCGCCCGGCGTGCTCGGTGCGCTTGGGATGCTCGGAGC

GACCGGCGCGACCGGCGCGACCGGTGCGACCGGTGCGATTGGCGCGGCGCGCCGGCGGTTTTAG

>Bucl15_Bm_GB8_horse_4.seq

GTGGCTGTGCCCACGAGGGGTTTTTTTGCGTGCGGCGGCGCGCCGGGCGTTTTCGGCGCGGCCACGCACGGACGCATGG

ACGCACGGATGCACGGATGCACGGATGCGCGATCGCTCGACGCGCCCGGCGTGCTCGGTGCGCTTGGGATGCTCGGAGC

GACCGGCGCGACCGGTGCGACCGGTGCGATTGGCGCGGCGCGCCGGCGGTTTTAG

>Bucl15_Bm_ATCC_23344.seq

gtggctgtgcccacgaggggtttttttgcgtgcggcggcgcgccgggcgttttcggcgcggccacgcacggacgcatgg

acgcacggatgcacggatgcacggatgcgcgatcgctcgacgcgcccggcgtgctcggtgcgcttgggatgctcggagc

gaccggcgcgaccggtgcgaccggtgcgattggcgcggcgcgccggcggttttag

>Bucl15_Bm_SAVP1.seq

GTGGCTGTGCCCACGAGGGGTTTTTTTGCGTGCGGCGGCGCGCCGGGCGTTTTCGGCGCGGCCACGCACGGACGCATGG

ACGCACGGATGCACGGATGCACGGATGCGCGATCGCTCGACGCGCCCGGCGTGCTCGGTGCGCTTGGGATGCTCGGAGC

GACCGGCGCGACCGGCGCGACCGGTGCGACCGGTGCGATTGGCGCGGCGCGCCGGCGGTTTTAG

>A3NSP4_BURP0_Bucl16_Bp_1106a.seq Created: Tuesday, December 31, 2013 11:21 AM

atgggtcacgcgaaaccgcgcgcgcctcgtcacgaggccgcgcggttttgtttggcgcgcgcgccgcgcggcggcgcgg

cggatacggcggtgcccgcgttcggggaggcgttcgcgcgcgcggtcgtgttcgccgcaggcgcgcggacggttttcgt

tcgtcgggaggcggctgtcgccgcgatctgcgtcgacggcttcggtggcttcggtggtgtcgacggcttcgacggcttc

gacggcttcgacggcttcgacggcttcgacggcttcgacggcttcgacggcttcgacggcttcgacggcttcgacggct

tcgacggcttcgacggcttcgacggcttcgacggcttcgacggcttcgacggcttcgacggcttcgacggcttcgacgg

cttcgacggcttcggtgccttcgacaatctcaccgtcttcgaagttttcgccgcctccgtcgccttcctttcctttggc

gcttgcgtcgcctcaccctcgtcagccggcgtgcccgcatcgagcgcctcggccgtcgcccgagtggcgcggcgaggag

atcgcggcgtcggcgtgcccgacgcccgttcggcgtcgacgcgcttcacgcgccccgtgacgggcgcgccctccctctg

tgccgccgccgcagcagctacctgggcccccgcatcgcgccccgccgccgcatcgcttcgcacgccggccgccgccggc

agcaccgactcgagcgtgcgcgtgcccgctgccagctcgcgcttctgcacggcggtgagccgcttcacccaatactcgg

cgcgcgacgcgctcgagcgatcggcgagcgcgaacgacgcgagcaccgcgcgcggcttgcgcgcgcgcgtgtag

>Bucl16_Bps_1710b.seq Created: Tuesday, December 31, 2013 1:48 PM

atgggtcacgcgaaaccgcgcgcgcctcgtcacgaggccgcgcggttttgtttggcgcgcgcgccgcgcggcggcgcgg

cggatacggcggtgcccgcgttcggggaggcgttcgcgcgcgcggtcgtgttcgccgcaggcgcgcggacggttttcgt

tcgttgggaggcggctgtcgccgcgatcggcttcggtggcttcgacggcttcggtggcttcgatgccttcggcggcttc

gatgccttcggtgccttcggtgccttcggtgccttcggtgccttcggtgccttcggtgccttcggtgccttcggtgcct

tcggtgccttcgacaatctcaccgtcttcgaagttttcgcagcctccgtcgccttcctttcctttggcgcttgcgtcgc

ctcaccctcgtcagccggcgtgcccgcatcgagcgcctcggccgtcgcccgagtggcgcggcgaggagatcgcggcgtc

ggcgtgcccgacgcccgttcggcgtcgacgcgcttcacgcgccccgtgacgggcgcgccctccctctgtgccgccgccg

cagcagctacctgggcccccgcatcgcgccccgccgccgcatcgcttcgcacgccggccgccgccggcagcaccgactc

gagcgtgcgcgtgcccgctgccagctcgcgcttctgcacggcggtgagccgcttcacccaatactcggcgcgcgacgcg

ctcgagcgatcggcgagcgcgaacgacgcgagcaccgcgcgcggcttgcgcgcgcgcgtgtag

>Bucl16_Bps_MSHR_305.seq Created: Thursday, January 02, 2014 1:02 PM

atgggtcacgcgaaaccgcgcgcgcctcgtcacgaggccgcgcggttttgtttggcgcgcgcgccgcgcggcggcgcgg

cggatacggcggtgcccgcgttcggggcggcgttcgcatgcgcggtcgtgttcgccgcaggcgcgcggacggttttcgt

tcgtcgggaggcggctgtcgccgcgatctgcgtcgacggcttcggtggcttcggtggtgtcgacggcttcgatggcttc

gatggcttcgatggcttcgatggcttcggtggcttcgatgccttcgatgccttcgatgccttcgatgccttcgatgcct

tcgatgccttcgatgccttcgatgccttcgatgccttcgacaatctcaccgtcttcgaagttttcgcagcctccgtcgc

cttcctttcctttggcgcttgcgtcgcctcaccctcgtcagccggcgtgcccgcatcgagcgcctcggccgtcgcccga

gtggcgcggcgaggagatcgcggcgtcggcgtgcccgacgcccgttcggcgtcgacgcgcttcacgcgccccgtgacgg

gcgcgccctccctctgtgccgccgcagcagcagctacctgggcccccgcatcgcgccccgccgccgcatcgcttcgcac

gccggccgccgccggcagcaccgactcgagcgtgcgcgtgcccgctgccagctcgcgcttctgcacggcggtgagccgc

ttcacccaatactcggcgcgcgacgcgctcgagcgatcggcgagcgcgaacgacgcgagcaccgcgcgcggcttgcgcg

cgcgcgtgtag

>Bucl16_Bps_NCTC_13179.seq Reverse Complement DNA Sequence Untitled Seq #14(1,774)

atgggtcacgcgaaaccgcgcgcgcctcgtcacgaggccgcgcggttttgtttggcgcgcgcgccgcgcggcggcgcgg

cggatacggcggtgcccgcgttcggggcggcgttcgcatgcgcatgcgcggtcgtgctcgccgcaggcgcgcggacggt

tttcgttcgtcgggaggcggctgtcgccgcgatctgcgtcgacggcttcggtggcttcggtggcttcgacggcttcgac

ggcttcgacggcttcgacggcttcgacggcttcgacggcttcgacggcttcgacggcttcgacggcttcgacggcttcg

gtgccttcgacaatctcaccgtcttcgaagttttcgcagcctccgtcgtcttcctttcctttggcgcttgcgtcgcctc

accctcgtcagccggcgtgcccgcatcgagcgcctcggccgtcgcccgattggcgcggcgacgagaccgcggcgtcggc

gtgcccgacgcccgttcggcgtcgacgcgcttcacgcgccccgtgacgggcgcgccctccctctgtgccgccgccgccg

cagcagctacctgggcccccgcatcgcgccccgccgccgcatcgcttcgcacgccggccgccgccggcagcaccgactc

gagcgtgcgcgtgcccgctgccagctcgcgcttctgcacggcggtgagccgcttcacccaatactcggcgcgcgacgcg

ctcgagcgatcggcgagcgcgaacgacgcgagcaccgcccgcggcttgcgcgcgcgcgtgtag

>Bucl16_Bm_ATCC_23344.seq Created: Friday, January 03, 2014 2:32 PM

atgggtcacgcgaaaccgcgcgcgcctcgtcacgaggccgcgcggttttgtttggcgcgcgcgccgcgcggcggcgcgg

cggatacggcggtgcccgcgttcggggcggcgttcgcatgcgcatgcgcggtcgtgctcgccgcaggcgcgcggacggt

tttcgttcgtcgggaggcggctgtcgccgcgatctgcgtcgacggcttcggtggcttcggtggtgtcgacggcttcgac

ggcttcgacggcttcgacggcttcgacggcttcgacggcttcgacggcttcgacggcttcgacggcttcgacggcttcg

acggcttcgacggcttcgacggcttcgacggcttcgacggcttcggtgccttcgacaatctcaccgtcttcgaagtttt

cgccgcctccgtcgccttcctttcctttggcgcttgcgtcgcctcaccctcgtcagccggcgtgcccgcatcgagcgcc

tcggccgtcgcccgagtggcgcggcgaggagatcgcggcgtcggcgtgcccgacgcccgttcggcgtcgacgcgcttca

cgcgccccgtgacgggcgcgccctccctctgtgccgccgccgcagcagctacctgggcccccgcatcgcgccccgccgc

cgcatcgcttcgcacgccggccgccgccggcagcaccgactcgagcgtgcgtgtgcccgctgccagctcgcgcttctgc

acggcggtgagccgcttcacccaatactcggcgcgcgacgcgctcgagcgatcggcgagctcgaacgacgcgagcaccg

cgcgcggcttgcgcgcgcgcgtgtag

>Bucl16_Bm_NCTC_10229.seq Created: Friday, January 03, 2014 4:33 PM

atgggtcacgcgaaaccgcgcgcgcctcgtcacgaggccgcgcggttttgtttggcgcgcgcgccgcgcggcggcgcgg

cggatacggcggtgcccgcgttcggggcggcgttcgcatgcgcatgcgcggtcgtgctcgccgcaggcgcgcggacggt

tttcgttcgtcgggaggcggctgtcgccgcgatctgcgtcgacggcttcggtggcttcggtggtgtcgacggcttcgac

ggcttcgacggcttcgacggcttcgacggcttcgacggcttcgacggcttcgacggcttcgacggcttcgacggcttcg

acggcttcgacggcttcgacggcttcgacggcttcgacggcttcgacggcttcgacggcttcgacggcttcgacggctt

cgacggcttcgacggcttcgacggcttcgacggcttcgacggcttcgacggcttcgacggcttcgacggcttcggtgcc

ttcgacaatctcaccgtcttcgaagttttcgccgcctccgtcgccttcctttcctttggcgcttgcgtcgcctcaccct

cgtcagccggcgtgcccgcatcgagcgcctcggccgtcgcccgagtggcgcggcgaggagatcgcggcgtcggcgtgcc

cgacgcccgttcggcgtcgacgcgcttcacgcgccccgtgacgggcgcgccctccctctgtgccgccgccgcagcagct

acctgggcccccgcatcgcgccccgccgccgcatcgcttcgcacgccggccgccgccggcagcaccgactcgagcgtgc

gtgtgcccgctgccagctcgcgcttctgcacggcggtgagccgcttcacccaatactcggcgcgcgacgcgctcgagcg

atcggcgagctcgaacgacgcgagcaccgcgcgcggcttgcgcgcgcgcgtgtag

>Bucl16_Bm_SAVP1.seq Created: Saturday, January 04, 2014 1:30 PM

atgggtcacgcgaaaccgcgcgcgcctcgtcacgaggccgcgcggttttgtttggcgcgcgcgccgcgcggcggcgcgg

cggatacggcggtgcccgcgttcggggcggcgttcgcatgcgcatgcgcggtcgtgctcgccgcaggcgcgcggacggt

tttcgttcgtcgggaggcggctgtcgccgcgatctgcgtcgacggcttcggtggcttcggtggtgtcgacggcttcgac

ggcttcgacggcttcgacggcttcgacggcttcgacggcttcgacggcttcgacggcttcgacggcttcgacggcttcg

acggcttcgacggcttcgacggcttcgacggcttcgacggcttcgacggcttcgacggcttcggtgccttcgacaatct

caccgtcttcgaagttttcgccgcctccgtcgccttcctttcctttggcgcttgcgtcgcctcaccctcgtcagccggc

gtgcccgcatcgagcgcctcggccgtcgcccgagtggcgcggcgaggagatcgcggcgtcggcgtgcccgacgcccgtt

cggcgtcgacgcgcttcacgcgccccgtgacgggcgcgccctccctctgtgccgccgccgcagcagctacctgggcccc

cgcatcgcgccccgccgccgcatcgcttcgcacgccggccgccgccggcagcaccgactcgagcgtgcgtgtgcccgct

gccagctcgcgcttctgcacggcggtgagccgcttcacccaatactcggcgcgcgacgcgctcgagcgatcggcgagct

cgaacgacgcgagcaccgcgcgcggcttgcgcgcgcgcgtgtag

>Bucl16_Bm_NCTC_10247.seq Created: Monday, January 20, 2014 8:44 PM

atgggtcacgcgaaaccgcgcgcgcctcgtcacgaggccgcgcggttttgtttggcgcgcgcgccgcgcggcggcgcgg

cggatacggcggtgcccgcgttcggggcggcgttcgcatgcgcatgcgcggtcgtgctcgccgcaggcgcgcggacggt

tttcgttcgtcgggaggcggctgtcgccgcgatctgcgtcgacggcttcggtggcttcggtggtgtcgacggcttcgac

ggcttcgacggcttcgacggcttcgacggcttcgacggcttcgacggcttcgacggcttcgacggcttcgacggcttcg

acggcttcgacggcttcgacggcttcgacggcttcgacggcttcgacggcttcgacggcttcgacggcttcgacggctt

cgacggcttcgacggcttcgacggcttcgacggcttcggtgccttcgacaatctcaccgtcttcgaagttttcgccgcc

tccgtcgccttcctttcctttggcgcttgcgtcgcctcaccctcgtcagccggcgtgcccgcatcgagcgcctcggccg

tcgcccgagtggcgcggcgaggagatcgcggcgtcggcgtgcccgacgcccgttcggcgtcgacgcgcttcacgcgccc

cgtgacgggcgcgccctccctctgtgccgccgccgcagcagctacctgggcccccgcatcgcgccccgccgccgcatcg

cttcgcacgccggccgccgccggcagcaccgactcgagcgtgcgtgtgcccgctgccagctcgcgcttctgcacggcgg

tgagccgcttcacccaatactcggcgcgcgacgcgctcgagcgatcggcgagctcgaacgacgcgagcaccgcgcgcgg

cttgcgcgcgcgcgtgtag

>Bucl16_Bps_1026b.seq Created: Monday, January 20, 2014 8:48 PM

atgggtcacgcgaaaccgcgcgcgcctcgtcacgaggccgcgcggttttgtttggcgcgcgcgccgcgcggcggcgcgg

cggatacggcggtgcccgcgttcggggaggcgttcgcgcgcgcggtcgtgttcgccgcaggcgcgcggacggttttcgt

tcgtcgggaggcggctgtcgccgcgatctgcgtcgacggcttcggtggcttcggtggtgtcgacggcttcgatggcttc

gatgccttcgatggcttcgacggcttcgacggcttcgacggcttcgacggcttcgacggcttcgacggcttcgacggct

tcgacggcttcgacggcttcgacggcttcgacggcttcgacggcttcgacggcttcgacggcttcgacggcttcggtgc

cttcgacaatctcaccgtcttcgaagttttcgcagcctccgtcgtcttcctttcctttggcgcttgcgtcgcctcaccc

tcgtcagccggcgtgcccgcatcgagcgcctcggccgtcgcccgagtggcgcggcgaggagagcgcggcgtcggcgtgc

ccgacgcccgttcggcgtcgacgcgcttcacgcgccccgtgacgggcgcgccctccctctgtgccgccgccgcagcagc

tacctgggcccccgcatcgcgccccgccgccgcatcgcttcgcacgccggccgccgccggcagcaccgactcgagcgtg

cgtgtgcccgctgccagctcgcgcttctgcacggcggtgagccgcttcacccaatactcggcgcgcgacgcgctcgagc

gatcggcgagctcgaacgacgcgagcaccgcgcgcggcttgcgcgcgcgcgtgtag

>Bucl16_Bps_BPC006.seq Created: Monday, January 20, 2014 8:53 PM

atgggtcacgcgaaaccgcgcgcgcctcgtcacgaggccgcgcggttttgtttggcgcgcgcgccgcgcggcggcgcgg

cggatacggcggtgcccgcgttcggggaggcgttcgcgcgcgcggtcgtgttcgccgcaggcgcgcggacggttttcgt

tcgtcgggaggcggctgtcgccgcgatctgcgtcgacggcttcggtggcttcggtggtgtcgacggcttcgacggcttc

gacggcttcgacggcttcgacggcttcgacggcttcgacggcttcgacggcttcgacggcttcgacggcttcgacggct

tcgacggcttcgacggcttcgacggcttcgacggcttcgacggcttcgacggcttcgacggcttcgacggcttcgacgg

cttcgacggcttcggtgccttcgacaatctcaccgtcttcgaagttttcgccgcctccgtcgccttcctttcctttggc

gcttgcgtcgcctcaccctcgtcagccggcgtgcccgcatcgagcgcctcggccgtcgcccgagtggcgcggcgaggag

atcgcggcgtcggcgtgcccgacgcccgttcggcgtcgacgcgcttcacgcgccccgtgacgggcgcgccctccctctg

tgccgccgccgcagcagctacctgggcccccgcatcgcgccccgccgccgcatcgcttcgcacgccggccgccgccggc

agcaccgactcgagcgtgcgcgtgcccgctgccagctcgcgcttctgcacggcggtgagccgcttcacccaatactcgg

cgcgcgacgcgctcgagcgatcggcgagcgcgaacgacgcgagcaccgcgcgcggcttgcgcgcgcgcgtgtag

>Bucl16_Bps_K96243.seq Created: Monday, January 20, 2014 8:57 PM

atgggtcacgcgaaaccgcgcgcgcctcgtcacgaggccgcgcggttttgtttggcgcgcgcgccgcgcggcggcgcgg

cggatacggcggtgcccgcgttcggggaggcgttcgcgcgcgcggtcgtgttcgccgcaggcgcgcggacggttttcgt

tcgtcgggaggcggctgtcgccgcgatctgcgtcgacggcttcggtggcttcggtggtgtcgacggcttcggtgccttc

gacaatctcaccgtcttcgaagttttcgcagcctccgtcgtcttcctttcctttggcgcttgcgtcgcctcaccctcgt

cagccggcgtgcccgcatcgagcgcctcggccgtcgcccgagtggcgcggcgaggagagcgcggcgtcggcgtgcccga

cgcccgttcggcgtcgacgcgcttcacgcgccccgtgacgggcgcgccctccctctgtgccgccgccgcagcagctacc

tgggcccccgcatcgcgccccgccgccgcatcgcttcgcacgccggccgccgccggcagcaccgactcgagcgtgcgtg

tgcccgctgccagctcgcgcttctgcacggcggtgagccgcttcacccaatactcggcgcgcgacgcgctcgagcgatc

ggcgagctcgaacgacgcgagcaccgcgcgcggcttgcgcgcgcgcgtgtag

>Bucl16_Bps_668.seq Created: Monday, January 20, 2014 9:01 PM

atgggtcacgcgaaaccgcgcgcgcctcgtcacgaggccgcgcggttttgtttggcgcgcgcgccgcgcggcggcgcgg

cggatacggcggtgcccgcgttcggggcggcgttcgcatgcgcatgcgcggtcgtgctcgccgcaggcgcgcggacggt

tttcgttcgtcgggaggcggctgtcgccgcgatcggcgtcgacggcttcggtggtgtcgacggcttcgatggcttcgat

gccttcggcggcttcggtgccttcggtggcttcggtggcttcggtggcttcggtggcttcggtggcttcgacaatctca

ccgtcttcgaagttttcgcagcctccgtcgccttcctttcctttggcgcttgcgtcgcctcaccctcgtcagccggcgt

gcccgcatcgagcgcctcggccgtcgcccgattggcgcggcgacgagaccgcggcgtcggcgtgcccgacgcccgttcg

gcgtcgacgcgcttcacgcgccccgtgacgggcgcgccctccctctgtgccgccgccgcatcgcttcgcacgccggccg

ccgccggcagcaccgactcgagcgtgcgcgtgcccgctgccagctcgcgcttctgcacggcggtgagccgcttcaccca

atactcggcgcgcgacgcgctcgagcgatcggcgagctcgaacgacgcgagcaccgcccgcggcttgcgtgcgcgcgtg

tag

>Bucl16_Bps_MSHR346.seq Created: Monday, January 20, 2014 9:06 PM

ATGGGTCACGCGAAACCGCGCGCGCCTCGTCACGAGGCCGCGCGGTTTTGTTTGGCGCGCGCGCCGCGCGGCGGCGCGG

CGGATACGGCGGTGCCCGCGTTCGGGGCGGCGTTCGCATGCGCATGCGCGGTCGTGCTCGCCGCAGGCGCGCGGACGGT

TTTCGTTCGTCGGGAGGCGGCTGTCGCCGCGATCGGCGTCGACGGCTTCGGTGGTGTCGACGGCTTCGATGGCTTCGAT

GCCTTCGGCGGCTTCGGTGCCTTCGGTGGCTTCGGTGGCTTCGGTGGCTTCGGTGGCTTCGGTGGCTTCGACAATCTCA

CCGTCTTCGAAGTTTTCGCAGCCTCCGTCGCCTTCCTTTCCTTTGGCGCTTGCGTCGCCTCACCCTCGTCAGCCGGCGT

GCCCGCATCGAGCGCCTCGGCCGTCGCCCGATTGGCGCGGCGACGAGACCGCGGCGTCGGCGTGCCCGACGCCCGTTCG

GCGTCGACGCGCTTCACGCGCCCCGTGACGGGCGCGCCCTCCCTCTGTGCCGCCGCCGCATCGCTTCGCACGCCGGCCG

CCGCCGGCAGCACCGACTCGAGCGTGCGCGTGCCCGCTGCCAGCTCGCGCTTCTGCACGGCGGTGAGCCGCTTCACCCA

ATACTCGGCGCGCGACGCGCTCGAGCGATCGGCGAGCTCGAACGACGCGAGCACCGCCCGCGGCTTGCGTGCGCGCGTG

TAG

>Bucl16_Bm_ATCC_10399.seq >gi|99031401|gb|AAHN02000014.1|:45919-46797 Burkholderia mallei ATCC 10399 ctg_1047277000600, whole genome shotgun sequence

ATGGGTCACGCGAAACCGCGCGCGCCTCGTCACGAGGCCGCGCGGTTTTGTTTGGCGCGCGCGCCGCGCGGCGGCGCGG

CGGATACGGCGGTGCCCGCGTTCGGGGCGGCGTTCGCATGCGCATGCGCGGTCGTGCTCGCCGCAGGCGCGCGGACGGT

TTTCGTTCGTCGGGAGGCGGCTGTCGCCGCGATCTGCGTCGACGGCTTCGGTGGTGTCGACGGCTTCGACGGCTTCGAC

GGCTTCGACGGCTTCGACGGCTTCGACGGCTTCGACGGCTTCGACGGCTTCGACGGCTTCGACGGCTTCGACGGCTTCG

ACGGCTTCGACGGCTTCGACGGCTTCGACGGCTTCGACGGCTTCGACGGCTTCGACGGCTTCGACGGCTTCGACGGCTT

CGACGGCTTCGACGGCTTCGACGGCTTCGGTGCCTTCGACAATCTCACCGTCTTCGAAGTTTTCGCCGCCTCCGTCGCC

TTCCTTTCCTTTGGCGCTTGCGTCGCCTCACCCTCGTCAGCCGGCGTGCCCGCATCGAGCGCCTCGGCCGTCGCCCGAG

TGGCGCGGCGAGGAGATCGCGGCGTCGGCGTGCCCGACGCCCGTTCGGCGTCGACGCGCTTCACGCGCCCCGTGACGGG

CGCGCCCTCCCTCTGTGCCGCCGCCGCAGCAGCTACCTGGGCCCCCGCATCGCGCCCCGCCGCCGCATCGCTTCGCACG

CCGGCCGCCGCCGGCAGCACCGACTCGAGCGTGCGTGTGCCCGCTGCCAGCTCGCGCTTCTGCACGGCGGTGAGCCGCT

TCACCCAATACTCGGCGCGCGACGCGCTCGAGCGATCGGCGAGCTCGAACGACGCGAGCACCGCGCGCGGCTTGCGCGC

GCGCGTGTAG

>Bucl16_Bm_2000031281.seq >gi|577031635|gb|AWGQ01000089.1|:12221-13027 Burkholderia mallei 2000031281 Burkholderia-mallei-MCB-013_1759, whole genome shotgun sequence

ATGGGTCACGCGAAACCGCGCGCGCCTCGTCACGAGGCCGCGCGGTTTTGTTTGGCGCGCGCGCCGCGCGGCGGCGCGG

CGGATACGGCGGTGCCCGCGTTCGGGGCGGCGTTCGCATGCGCATGCGCGGTCGTGCTCGCCGCAGGCGCGCGGACGGT

TTTCGTTCGTCGGGAGGCGGCTGTCGCCGCGATCTGCGTCGACGGCTTCGGTGGCTTCGGTGGTGTCGACGGCTTCGAC

GGCTTCGACGGCTTCGACGGCTTCGACGGCTTCGACGGCTTCGACGGCTTCGACGGCTTCGACGGCTTCGACGGCTTCG

ACGGCTTCGACGGCTTCGACGGCTTCGACGGCTTCGGTGCCTTCGACAATCTCACCGTCTTCGAAGTTTTCGCCGCCTC

CGTCGCCTTCCTTTCCTTTGGCGCTTGCGTCGCCTCACCCTCGTCAGCCGGCGTGCCCGCATCGAGCGCCTCGGCCGTC

GCCCGAGTGGCGCGGCGAGGAGATCGCGGCGTCGGCGTGCCCGACGCCCGTTCGGCGTCGACGCGCTTCACGCGCCCCG

TGACGGGCGCGCCCTCCCTCTGTGCCGCCGCCGCAGCAGCTACCTGGGCCCCCGCATCGCGCCCCGCCGCCGCATCGCT

TCGCACGCCGGCCGCCGCCGGCAGCACCGACTCGAGCGTGCGTGTGCCCGCTGCCAGCTCGCGCTTCTGCACGGCGGTG

AGCCGCTTCACCCAATACTCGGCGCGCGACGCGCTCGAGCGATCGGCGAGCTCGAACGACGCGAGCACCGCGCGCGGCT

TGCGCGCGCGCGTGTAG

>Bucl16_Bm_2002721280.seq >gi|99033648|gb|AANX02000021.1|:108256-109071 Burkholderia mallei 2002721280 ctg2_1099945121860, whole genome shotgun sequence

ATGGGTCACGCGAAACCGCGCGCGCCTCGTCACGAGGCCGCGCGGTTTTGTTTGGCGCGCGCGCCGCGCGGCGGCGCGG

CGGATACGGCGGTGCCCGCGTTCGGGGCGGCGTTCGCATGCGCATGCGCGGTCGTGCTCGCCGCAGGCGCGCGGACGGT

TTTCGTTCGTCGGGAGGCGGCTGTCGCCGCGATCTGCGTCGACGGCTTCGGTGGCTTCGGTGGTGTCGACGGCTTCGAC

GGCTTCGACGGCTTCGACGGCTTCGACGGCTTCGACGGCTTCGACGGCTTCGACGGCTTCGACGGCTTCGACGGCTTCG

ACGGCTTCGACGGCTTCGACGGCTTCGACGGCTTCGACGGCTTCGGTGCCTTCGACAATCTCACCGTCTTCGAAGTTTT

CGCCGCCTCCGTCGCCTTCCTTTCCTTTGGCGCTTGCGTCGCCTCACCCTCGTCAGCCGGCGTGCCCGCATCGAGCGCC

TCGGCCGTCGCCCGAGTGGCGCGGCGAGGAGATCGCGGCGTCGGCGTGCCCGACGCCCGTTCGGCGTCGACGCGCTTCA

CGCGCCCCGTGACGGGCGCGCCCTCCCTCTGTGCCGCCGCCGCAGCAGCTACCTGGGCCCCCGCATCGCGCCCCGCCGC

CGCATCGCTTCGCACGCCGGCCGCCGCCGGCAGCACCGACTCGAGCGTGCGTGTGCCCGCTGCCAGCTCGCGCTTCTGC

ACGGCGGTGAGCCGCTTCACCCAATACTCGGCGCGCGACGCGCTCGAGCGATCGGCGAGCTCGAACGACGCGAGCACCG

CGCGCGGCTTGCGCGCGCGCGTGTAG

>Bucl16_Bm_A188.seq >gi|577031707|gb|AWGR01000054.1|:12222-13028 Burkholderia mallei A188 Burkholderia-mallei-MCB-014_229, whole genome shotgun sequence

ATGGGTCACGCGAAACCGCGCGCGCCTCGTCACGAGGCCGCGCGGTTTTGTTTGGCGCGCGCGCCGCGCGGCGGCGCGG

CGGATACGGCGGTGCCCGCGTTCGGGGCGGCGTTCGCATGCGCATGCGCGGTCGTGCTCGCCGCAGGCGCGCGGACGGT

TTTCGTTCGTCGGGAGGCGGCTGTCGCCGCGATCTGCGTCGACGGCTTCGGTGGCTTCGGTGGTGTCGACGGCTTCGAC

GGCTTCGACGGCTTCGACGGCTTCGACGGCTTCGACGGCTTCGACGGCTTCGACGGCTTCGACGGCTTCGACGGCTTCG

ACGGCTTCGACGGCTTCGACGGCTTCGACGGCTTCGGTGCCTTCGACAATCTCACCGTCTTCGAAGTTTTCGCCGCCTC

CGTCGCCTTCCTTTCCTTTGGCGCTTGCGTCGCCTCACCCTCGTCAGCCGGCGTGCCCGCATCGAGCGCCTCGGCCGTC

GCCCGAGTGGCGCGGCGAGGAGATCGCGGCGTCGGCGTGCCCGACGCCCGTTCGGCGTCGACGCGCTTCACGCGCCCCG

TGACGGGCGCGCCCTCCCTCTGTGCCGCCGCCGCAGCAGCTACCTGGGCCCCCGCATCGCGCCCCGCCGCCGCATCGCT

TCGCACGCCGGCCGCCGCCGGCAGCACCGACTCGAGCGTGCGTGTGCCCGCTGCCAGCTCGCGCTTCTGCACGGCGGTG

AGCCGCTTCACCCAATACTCGGCGCGCGACGCGCTCGAGCGATCGGCGAGCTCGAACGACGCGAGCACCGCGCGCGGCT

TGCGCGCGCGCGTGTAG

>Bucl16_Bm_A193.seq Reverse Complement DNA Sequence AWGS01000111.seq(1,762)

ATGGGTCACGCGAAACCGCGCGCGCCTCGTCACGAGGCCGCGCGGTTTTGTTTGGCGCGCGCGCCGCGCGGCGGCGCGG

CGGATACGGCGGTGCCCGCGTTCGGGGCGGCGTTCGCATGCGCATGCGCGGTCGTGCTCGCCGCAGGCGCGCGGACGGT

TTTCGTTCGTCGGGAGGCGGCTGTCGCCGCGATCTGCGTCGACGGCTTCGGTGGCTTCGGTGGTGTCGACGGCTTCGAC

GGCTTCGACGGCTTCGACGGCTTCGACGGCTTCGACGGCTTCGACGGCTTCGACGGCTTCGACGGCTTCGGTGCCTTCG

ACAATCTCACCGTCTTCGAAGTTTTCGCCGCCTCCGTCGCCTTCCTTTCCTTTGGCGCTTGCGTCGCCTCACCCTCGTC

AGCCGGCGTGCCCGCATCGAGCGCCTCGGCCGTCGCCCGAGTGGCGCGGCGAGGAGATCGCGGCGTCGGCGTGCCCGAC

GCCCGTTCGGCGTCGACGCGCTTCACGCGCCCCGTGACGGGCGCGCCCTCCCTCTGTGCCGCCGCCGCAGCAGCTACCT

GGGCCCCCGCATCGCGCCCCGCCGCCGCATCGCTTCGCACGCCGGCCGCCGCCGGCAGCACCGACTCGAGCGTGCGTGT

GCCCGCTGCCAGCTCGCGCTTCTGCACGGCGGTGAGCCGCTTCACCCAATACTCGGCGCGCGACGCGCTCGAGCGATCG

GCGAGCTCGAACGACGCGAGCACCGCGCGCGGCTTGCGCGCGCGCGTGTAG

>Bucl16_Bm_JHU.seq >gi|83595687|gb|AAIR02000035.1|:47184-47999 Burkholderia mallei JHU ctg_1099471777643, whole genome shotgun sequence

ATGGGTCACGCGAAACCGCGCGCGCCTCGTCACGAGGCCGCGCGGTTTTGTTTGGCGCGCGCGCCGCGCGGCGGCGCGG

CGGATACGGCGGTGCCCGCGTTCGGGGCGGCGTTCGCATGCGCATGCGCGGTCGTGCTCGCCGCAGGCGCGCGGACGGT

TTTCGTTCGTCGGGAGGCGGCTGTCGCCGCGATCTGCGTCGACGGCTTCGGTGGCTTCGGTGGTGTCGACGGCTTCGAC

GGCTTCGACGGCTTCGACGGCTTCGACGGCTTCGACGGCTTCGACGGCTTCGACGGCTTCGACGGCTTCGACGGCTTCG

ACGGCTTCGACGGCTTCGACGGCTTCGACGGCTTCGACGGCTTCGGTGCCTTCGACAATCTCACCGTCTTCGAAGTTTT

CGCCGCCTCCGTCGCCTTCCTTTCCTTTGGCGCTTGCGTCGCCTCACCCTCGTCAGCCGGCGTGCCCGCATCGAGCGCC

TCGGCCGTCGCCCGAGTGGCGCGGCGAGGAGATCGCGGCGTCGGCGTGCCCGACGCCCGTTCGGCGTCGACGCGCTTCA

CGCGCCCCGTGACGGGCGCGCCCTCCCTCTGTGCCGCCGCCGCAGCAGCTACCTGGGCCCCCGCATCGCGCCCCGCCGC

CGCATCGCTTCGCACGCCGGCCGCCGCCGGCAGCACCGACTCGAGCGTGCGTGTGCCCGCTGCCAGCTCGCGCTTCTGC

ACGGCGGTGAGCCGCTTCACCCAATACTCGGCGCGCGACGCGCTCGAGCGATCGGCGAGCTCGAACGACGCGAGCACCG

CGCGCGGCTTGCGCGCGCGCGTGTAG

>Bucl16_Bm_China_7.seq >gi|577031115|gb|AWGN01000090.1|:12222-13028 Burkholderia mallei China_7 Burkholderia-mallei-MCB-010_1372, whole genome shotgun sequence

ATGGGTCACGCGAAACCGCGCGCGCCTCGTCACGAGGCCGCGCGGTTTTGTTTGGCGCGCGCGCCGCGCGGCGGCGCGG

CGGATACGGCGGTGCCCGCGTTCGGGGCGGCGTTCGCATGCGCATGCGCGGTCGTGCTCGCCGCAGGCGCGCGGACGGT

TTTCGTTCGTCGGGAGGCGGCTGTCGCCGCGATCTGCGTCGACGGCTTCGGTGGCTTCGGTGGTGTCGACGGCTTCGAC

GGCTTCGACGGCTTCGACGGCTTCGACGGCTTCGACGGCTTCGACGGCTTCGACGGCTTCGACGGCTTCGACGGCTTCG

ACGGCTTCGACGGCTTCGACGGCTTCGACGGCTTCGGTGCCTTCGACAATCTCACCGTCTTCGAAGTTTTCGCCGCCTC

CGTCGCCTTCCTTTCCTTTGGCGCTTGCGTCGCCTCACCCTCGTCAGCCGGCGTGCCCGCATCGAGCGCCTCGGCCGTC

GCCCGAGTGGCGCGGCGAGGAGATCGCGGCGTCGGCGTGCCCGACGCCCGTTCGGCGTCGACGCGCTTCACGCGCCCCG

TGACGGGCGCGCCCTCCCTCTGTGCCGCCGCCGCAGCAGCTACCTGGGCCCCCGCATCGCGCCCCGCCGCCGCATCGCT

TCGCACGCCGGCCGCCGCCGGCAGCACCGACTCGAGCGTGCGTGTGCCCGCTGCCAGCTCGCGCTTCTGCACGGCGGTG

AGCCGCTTCACCCAATACTCGGCGCGCGACGCGCTCGAGCGATCGGCGAGCTCGAACGACGCGAGCACCGCGCGCGGCT

TGCGCGCGCGCGTGTAG

>Bucl16_Bm_FMH.seq >gi|83595500|gb|AAIQ02000037.1|:46501-47316 Burkholderia mallei FMH ctg_1099464876501, whole genome shotgun sequence

ATGGGTCACGCGAAACCGCGCGCGCCTCGTCACGAGGCCGCGCGGTTTTGTTTGGCGCGCGCGCCGCGCGGCGGCGCGG

CGGATACGGCGGTGCCCGCGTTCGGGGCGGCGTTCGCATGCGCATGCGCGGTCGTGCTCGCCGCAGGCGCGCGGACGGT

TTTCGTTCGTCGGGAGGCGGCTGTCGCCGCGATCTGCGTCGACGGCTTCGGTGGCTTCGGTGGTGTCGACGGCTTCGAC

GGCTTCGACGGCTTCGACGGCTTCGACGGCTTCGACGGCTTCGACGGCTTCGACGGCTTCGACGGCTTCGACGGCTTCG

ACGGCTTCGACGGCTTCGACGGCTTCGACGGCTTCGACGGCTTCGGTGCCTTCGACAATCTCACCGTCTTCGAAGTTTT

CGCCGCCTCCGTCGCCTTCCTTTCCTTTGGCGCTTGCGTCGCCTCACCCTCGTCAGCCGGCGTGCCCGCATCGAGCGCC

TCGGCCGTCGCCCGAGTGGCGCGGCGAGGAGATCGCGGCGTCGGCGTGCCCGACGCCCGTTCGGCGTCGACGCGCTTCA

CGCGCCCCGTGACGGGCGCGCCCTCCCTCTGTGCCGCCGCCGCAGCAGCTACCTGGGCCCCCGCATCGCGCCCCGCCGC

CGCATCGCTTCGCACGCCGGCCGCCGCCGGCAGCACCGACTCGAGCGTGCGTGTGCCCGCTGCCAGCTCGCGCTTCTGC

ACGGCGGTGAGCCGCTTCACCCAATACTCGGCGCGCGACGCGCTCGAGCGATCGGCGAGCTCGAACGACGCGAGCACCG

CGCGCGGCTTGCGCGCGCGCGTGTAG

>Bucl16_Bm_GB8_horse_4.seq >gi|67105589|gb|AAHO01000072.1|:25452-26267 Burkholderia mallei GB8 horse 4 contig_371, whole genome shotgun sequence

ATGGGTCACGCGAAACCGCGCGCGCCTCGTCACGAGGCCGCGCGGTTTTGTTTGGCGCGCGCGCCGCGCGGCGGCGCGG

CGGATACGGCGGTGCCCGCGTTCGGGGCGGCGTTCGCATGCGCATGCGCGGTCGTGCTCGCCGCAGGCGCGCGGACGGT

TTTCGTTCGTCGGGAGGCGGCTGTCGCCGCGATCTGCGTCGACGGCTTCGGTGGCTTCGGTGGTGTCGACGGCTTCGAC

GGCTTCGACGGCTTCGACGGCTTCGACGGCTTCGACGGCTTCGACGGCTTCGACGGCTTCGACGGCTTCGACGGCTTCG

ACGGCTTCGACGGCTTCGACGGCTTCGACGGCTTCGACGGCTTCGGTGCCTTCGACAATCTCACCGTCTTCGAAGTTTT

CGCCGCCTCCGTCGCCTTCCTTTCCTTTGGCGCTTGCGTCGCCTCACCCTCGTCAGCCGGCGTGCCCGCATCGAGCGCC

TCGGCCGTCGCCCGAGTGGCGCGGCGAGGAGATCGCGGCGTCGGCGTGCCCGACGCCCGTTCGGCGTCGACGCGCTTCA

CGCGCCCCGTGACGGGCGCGCCCTCCCTCTGTGCCGCCGCCGCAGCAGCTACCTGGGCCCCCGCATCGCGCCCCGCCGC

CGCATCGCTTCGCACGCCGGCCGCCGCCGGCAGCACCGACTCGAGCGTGCGTGTGCCCGCTGCCAGCTCGCGCTTCTGC

ACGGCGGTGAGCCGCTTCACCCAATACTCGGCGCGCGACGCGCTCGAGCGATCGGCGAGCTCGAACGACGCGAGCACCG

CGCGCGGCTTGCGCGCGCGCGTGTAG

>Bucl16_Bm_PRL-20.seq >gi|145667041|gb|AAZP01000092.1|:18736-19560 Burkholderia mallei PRL-20 gcontig_1105338610794, whole genome shotgun sequence

ATGGGTCACGCGAAACCGCGCGCGCCTCGTCACGAGGCCGCGCGGTTTTGTTTGGCGCGCGCGCCGCGCGGCGGCGCGG

CGGATACGGCGGTGCCCGCGTTCGGGGCGGCGTTCGCATGCGCATGCGCGGTCGTGCTCGCCGCAGGCGCGCGGACGGT

TTTCGTTCGTCGGGAGGCGGCTGTCGCCGCGATCTGCGTCGACGGCTTCGGTGGCTTCGGTGGTGTCGACGGCTTCGAC

GGCTTCGACGGCTTCGACGGCTTCGACGGCTTCGACGGCTTCGACGGCTTCGACGGCTTCGACGGCTTCGACGGCTTCG

ACGGCTTCGACGGCTTCGACGGCTTCGACGGCTTCGACGGCTTCGACGGCTTCGGTGCCTTCGACAATCTCACCGTCTT

CGAAGTTTTCGCCGCCTCCGTCGCCTTCCTTTCCTTTGGCGCTTGCGTCGCCTCACCCTCGTCAGCCGGCGTGCCCGCA

TCGAGCGCCTCGGCCGTCGCCCGAGTGGCGCGGCGAGGAGATCGCGGCGTCGGCGTGCCCGACGCCCGTTCGGCGTCGA

CGCGCTTCACGCGCCCCGTGACGGGCGCGCCCTCCCTCTGTGCCGCCGCCGCAGCAGCTACCTGGGCCCCCGCATCGCG

CCCCGCCGCCGCATCGCTTCGCACGCCGGCCGCCGCCGGCAGCACCGACTCGAGCGTGCGTGTGCCCGCTGCCAGCTCG

CGCTTCTGCACGGCGGTGAGCCGCTTCACCCAATACTCGGCGCGCGACGCGCTCGAGCGATCGGCGAGCTCGAACGACG

CGAGCACCGCGCGCGGCTTGCGCGCGCGCGTGTAG

>Bucl16_Bm_strain_6.seq Reverse Complement DNA Sequence AWGO01000165.seq(1,807)

ATGGGTCACGCGAAACCGCGCGCGCCTCGTCACGAGGCCGCGCGGTTTTGTTTGGCGCGCGCGCCGCGCGGCGGCGCGG

CGGATACGGCGGTGCCCGCGTTCGGGGCGGCGTTCGCATGCGCATGCGCGGTCGTGCTCGCCGCAGGCGCGCGGACGGT

TTTCGTTCGTCGGGAGGCGGCTGTCGCCGCGATCTGCGTCGACGGCTTCGGTGGCTTCGGTGGTGTCGACGGCTTCGAC

GGCTTCGACGGCTTCGACGGCTTCGACGGCTTCGACGGCTTCGACGGCTTCGACGGCTTCGACGGCTTCGACGGCTTCG

ACGGCTTCGACGGCTTCGACGGCTTCGACGGCTTCGGTGCCTTCGACAATCTCACCGTCTTCGAAGTTTTCGCCGCCTC

CGTCGCCTTCCTTTCCTTTGGCGCTTGCGTCGCCTCACCCTCGTCAGCCGGCGTGCCCGCATCGAGCGCCTCGGCCGTC

GCCCGAGTGGCGCGGCGAGGAGATCGCGGCGTCGGCGTGCCCGACGCCCGTTCGGCGTCGACGCGCTTCACGCGCCCCG

TGACGGGCGCGCCCTCCCTCTGTGCCGCCGCCGCAGCAGCTACCTGGGCCCCCGCATCGCGCCCCGCCGCCGCATCGCT

TCGCACGCCGGCCGCCGCCGGCAGCACCGACTCGAGCGTGCGTGTGCCCGCTGCCAGCTCGCGCTTCTGCACGGCGGTG

AGCCGCTTCACCCAATACTCGGCGCGCGACGCGCTCGAGCGATCGGCGAGCTCGAACGACGCGAGCACCGCGCGCGGCT

TGCGCGCGCGCGTGTAG

>Bucl16_Bm_strain_11.seq >gi|577031286|gb|AWGP01000023.1|:12222-13028 Burkholderia mallei strain_11 Burkholderia-mallei-MCB-012_2073, whole genome shotgun sequence

ATGGGTCACGCGAAACCGCGCGCGCCTCGTCACGAGGCCGCGCGGTTTTGTTTGGCGCGCGCGCCGCGCGGCGGCGCGG

CGGATACGGCGGTGCCCGCGTTCGGGGCGGCGTTCGCATGCGCATGCGCGGTCGTGCTCGCCGCAGGCGCGCGGACGGT

TTTCGTTCGTCGGGAGGCGGCTGTCGCCGCGATCTGCGTCGACGGCTTCGGTGGCTTCGGTGGTGTCGACGGCTTCGAC

GGCTTCGACGGCTTCGACGGCTTCGACGGCTTCGACGGCTTCGACGGCTTCGACGGCTTCGACGGCTTCGACGGCTTCG

ACGGCTTCGACGGCTTCGACGGCTTCGACGGCTTCGGTGCCTTCGACAATCTCACCGTCTTCGAAGTTTTCGCCGCCTC

CGTCGCCTTCCTTTCCTTTGGCGCTTGCGTCGCCTCACCCTCGTCAGCCGGCGTGCCCGCATCGAGCGCCTCGGCCGTC

GCCCGAGTGGCGCGGCGAGGAGATCGCGGCGTCGGCGTGCCCGACGCCCGTTCGGCGTCGACGCGCTTCACGCGCCCCG

TGACGGGCGCGCCCTCCCTCTGTGCCGCCGCCGCAGCAGCTACCTGGGCCCCCGCATCGCGCCCCGCCGCCGCATCGCT

TCGCACGCCGGCCGCCGCCGGCAGCACCGACTCGAGCGTGCGTGTGCCCGCTGCCAGCTCGCGCTTCTGCACGGCGGTG

AGCCGCTTCACCCAATACTCGGCGCGCGACGCGCTCGAGCGATCGGCGAGCTCGAACGACGCGAGCACCGCGCGCGGCT

TGCGCGCGCGCGTGTAG

>Bucl16_Bps_MSHR511.seq Reverse Complement DNA Sequence CP004023.seq(1,819)

ATGGGTCACGCGAAACCGCGCGCGCCTCGTCACGAGGCCGCGCGGTTTTGTTTGGCGCGCGCGCCGCGCGGCGGCGCGG

CGGATACGGCGGTGCCCGCGTTCGGGGCGGCGTTCGCGCGCGCGGTCGTGTTCGCCGCAGGCGCGCGGACGGTTTTCGT

TCGTTGGGAGGCGGCCGTTGCCGCGATCGGCTTCGGTGGCTTCGATGCCTTCGGCGGCTTCGGTGGCTTCGATGCCTTC

GATGCCTTCGATGCCTTCGATGCCTTCGATGCCTTCGATGCCTTCGATGCCTTCGGTGCCTTCGGTGCCTTCGGTGCCT

TCGGTGCCTTCGGTGCCTTCGGTGCCTTCGGTGCCTTCGGTGCCTTCGGTGCCTTCGACAATCTCACCGTCTTCGAAGT

TTTCGCCGCCTCCGTCGTCTTCCTTTCCTTTGGCGCTTGCGTTGCCTCACCCTCGTCAGCCGGCGTGCCCGCATCGAGC

GCCTCGGCCGTCGCCCGAGTGGCGCGGCGAGGAGCGCGCGGCGTCGGCGTGCCCGACGCCCGTTCGGCGTCGACGCGCT

TCACGCGCCCCGTGACGGGCGCGCCCTCCCTCTGTGCCGCCGCCGCAGCAGCTACCTGGGCCCCCGCATCGCGCCCCGC

CGCCGCATCGCTTCGCACGCCGGCCGCCGCCGGCAGCACCGACTCGAGCGTGCGCGTGCCCGCTGCCAGCTCGCGCTTC

TGCACGGCGGTGAGCCGCTTCACCCAATACTCGGCGCGCGACGCGCTCGAGCGATCGGCGAGCTCGAACGACGCGAGCA

CCGCGCGCGGCTTGCGCGCGCGCGTGTAG

>Bucl16_Bps_NAU20B-16.seq Reverse Complement DNA Sequence CP004003.seq(1,801)

ATGGGTCACGCGAAACCGCGCGCGCCTCGTCACGAGGCCGCGCGGTTTTGTTTGGCGCGCGCGCCGCGCGGCGGCGCGG

CGGATACGGCGGTGCCCGCGTTCGGGGCGGCGTTCGCGCGCGCGGTCGTGTTCGCCGCAGGCGCGCGGACGGTTTTCGT

TCGTTGGGAGGCGGCCGTTGCCGCGATCGGCTTCGGTGGCTTCGATGCCTTCGGCGGCTTCGGTGGCTTCGATGCCTTC

GATGCCTTCGATGCCTTCGATGCCTTCGATGCCTTCGATGCCTTCGGTGCCTTCGGTGCCTTCGGTGCCTTCGGTGCCT

TCGGTGCCTTCGGTGCCTTCGGTGCCTTCGGTGCCTTCGACAATCTCACCGTCTTCGAAGTTTTCGCCGCCTCCGTCGT

CTTCCTTTCCTTTGGCGCTTGCGTTGCCTCACCCTCGTCAGCCGGCGTGCCCGCATCGAGCGCCTCGGCCGTCGCCCGA

GTGGCGCGGCGAGGAGCGCGCGGCGTCGGCGTGCCCGACGCCCGTTCGGCGTCGACGCGCTTCACGCGCCCCGTGACGG

GCGCGCCCTCCCTCTGTGCCGCCGCCGCAGCAGCTACCTGGGCCCCCGCATCGCGCCCCGCCGCCGCATCGCTTCGCAC

GCCGGCCGCCGCCGGCAGCACCGACTCGAGCGTGCGCGTGCCCGCTGCCAGCTCGCGCTTCTGCACGGCGGTGAGCCGC

TTCACCCAATACTCGGCGCGCGACGCGCTCGAGCGATCGGCGAGCTCGAACGACGCGAGCACCGCGCGCGGCTTGCGCG

CGCGCGTGTAG

>Bucl16_Bps_NCTC_13178.seq Reverse Complement DNA Sequence CP004001.seq(1,750)

ATGGGTCACGCGAAACCGCGCGCGCCTCGTCACGAGGCCGCGCGGTTTTGTTTGGCGCGCGCGCCGCGCGGCGGCGCGG

CGGATACGGCGGTGCCCGCGTTCGGGGCGGCGTTCGCATGCGCATGCGCGGTCGTGCTCGCCGCAGGCGCGCGGACGGT

TTTCGTTCGTCGGGAGGCGGCTGTCGCCGCGATCTGCGTCGACGGCTTCGGTGGCTTCGGTGGTGTCGACGGCTTCGAC

GGCTTCGACGGCTTCGACGGCTTCGACGGCTTCGACGGCTTCGACGGCTTCGACGGCTTCGGTGCCTTCGACAATCTCA

CCGTCTTCGAAGTTTTCGCAGCCTCCGTCGTCTTCCTTTCCTTTGGCGCTTGCGTCGCCTCACCCTCGTCAGCCGGCGT

GCCCGCATCGAGCGCCTCGGCCGTCGCCCGATTGGCGCGGCGAGGAGCGCGCGGCGTCGGCGTGCCCGACGCCCGTTCG

GCGTCGACGCGCTTCATGCGCCCCGTGACGGGCGCGCCCTCCCTCTGTGCCGCAGCAGCAGCTACCTGGGCCCCCGCAT

CGCGCCCCGCCGCCGCCTCGCTTCGCACGCCGGCCGCCGCCGGCAGCACCGACTCGAGCGTGCGCGTGCCCGCTGCCAG

CTCGCGCTTCTGCACGGCGGTGAGCCGCTTCACCCAATACTCGGCGCGCGACGCGCTCGAGCGATCGGCGAGCTCGAAC

GACGCGAGCACCGCGCGCGGCTTGCGCGCGCGCGTGTAG

>Bucl16_Bps_MSHR520.seq Reverse Complement DNA Sequence CP004368.seq(1,846)

ATGGGTCACGCGAAACCGCGCGCGCCTCGTCACGAGGCCGCGCGGTTTTGTTTGGCGCGCGCGCCGCGCGGCGGCGCGG

CGGATACGGCGGTGCCCGCGTTCGGGGCGGCGTTCGCATGCGCGGTCGTGTTCGCCGCAGGCGCGCGGACGGTTTTCGT

TCGTCGGGAGGCGGCTGTCGCCGCGATCTGCGTCGACGGCTTCGGTGGCTTCGGTGGTGTCGACGGCTTCGATGGCTTC

GATGGCTTCGATGGCTTCGATGGCTTCGATGGCTTCGGTGGCTTCGATGCCTTCGATGCCTTCGATGCCTTCGATGCCT

TCGATGCCTTCGATGCCTTCGATGCCTTCGATGCCTTCGATGCCTTCGATGCCTTCGATGCCTTCGATGCCTTCGATGC

CTTCGACAATCTCACCGTCTTCGAAGTTTTCGCAGCCTCCGTCGCCTTCCTTTCCTTTGGCGCTTGCGTCGCCTCACCC

TCGTCAGCCGGCGTGCCCGCATCGAGCGCCTCGGCCGTCGCCCGAGTGGCGCGGCGAGGAGATCGCGGCGTCGGCGTGC

CCGACGCCCGTTCGGCGTCGACGCGCTTCACGCGCCCCGTGACGGGCGCGCCCTCCCTCTGTGCCGCCGCAGCAGCAGC

TACCTGGGCCCCCGCATCGCGCCCCGCCGCCGCATCGCTTCGCACGCCGGCCGCCGCCGGCAGCACCGACTCGAGCGTG

CGCGTGCCCGCTGCCAGCTCGCGCTTCTGCACGGCGGTGAGCCGCTTCACCCAATACTCGGCGCGCGACGCGCTCGAGC

GATCGGCGAGCGCGAACGACGCGAGCACCGCGCGCGGCTTGCGCGCGCGCGTGTAG

>Bucl16_Bps_MSHR146.seq Reverse Complement DNA Sequence CP004042.seq(1,792)

ATGGGTCACGCGAAACCGCGCGCGCCTCGTCACGAGGCCGCGCGGTTTTGTTTGGCGCGCGCGCCGCGCGGCGGCGCGG

CGGATACGGCGGTGCCCGCGTTCGGGGCGGCGTTCGCGCGCGCGGTCGTGTTCGCCGCAGGCGCGCGGACGGTTTTCGT

TCGTTGGGAGGCGGCCGTTGCCGCGATCGGCTTCGGTGGCTTCGATGCCTTCGGCGGCTTCGGTGGCTTCGATGCCTTC

GATGCCTTCGATGCCTTCGATGCCTTCGATGCCTTCGATGCCTTCGGTGCCTTCGGTGCCTTCGGTGCCTTCGGTGCCT

TCGGTGCCTTCGGTGCCTTCGGTGCCTTCGACAATCTCACCGTCTTCGAAGTTTTCGCCGCCTCCGTCGTCTTCCTTTC

CTTTGGCGCTTGCGTTGCCTCACCCTCGTCAGCCGGCGTGCCCGCATCGAGCGCCTCGGCCGTCGCCCGAGTGGCGCGG

CGAGGAGCGCGCGGCGTCGGCGTGCCCGACGCCCGTTCGGCGTCGACGCGCTTCACGCGCCCCGTGACGGGCGCGCCCT

CCCTCTGTGCCGCCGCCGCAGCAGCTACCTGGGCCCCCGCATCGCGCCCCGCCGCCGCATCGCTTCGCACGCCGGCCGC

CGCCGGCAGCACCGACTCGAGCGTGCGCGTGCCCGCTGCCAGCTCGCGCTTCTGCACGGCGGTGAGCCGCTTCACCCAA

TACTCGGCGCGCGACGCGCTCGAGCGATCGGCGAGCTCGAACGACGCGAGCACCGCGCGCGGCTTGCGCGCGCGCGTGT

AG
