# Supplementary material for: A Unique Set of the Burkholderia Collagen-Like Proteins Provides Insight into Pathogenesis, Genome Evolution and Niche Adaptation, and Infection Detection
Source: PLoS One. 2015 Sep 10;10(9):e0137578. doi: 10.1371/journal.pone.0137578 (PMC4565658; doi:10.1371/journal.pone.0137578)
Supplement: S2 Dataset — Pairwise nucleotide sequence alignments were generated using the ClustalW algorithm in the DNAStar Megalign software and used to calculate percent identities and divergence. Each table contains percent identities (right side of black squares) and divergence values (left side of black squares) for each pairwise alignment. (PDF) [file pone.0137578.s002.pdf]

Percent Identity

	1	2	3	4	5	6	7	8	9	10	11	12	13	14	15	16	17	18	19	20	21	22	23	24	25	26	27	28	29		
1		90.2	90.7	87.7	93.6	96.8	90.4	87.5	86.5	89.6	93.8	93.3	90.9	96.6	89.2	84.7	89.2	90.6	89.2	91.9	89.2	86.2	96.8	86.2	89.2	89.2	93.4	89.2	89.2	1	Bucl1_Bps K96243.seq
2	2.6		98.1	91.4	94.6	89.1	97.8	80.3	78.8	81.8	86.4	94.9	83.2	88.9	95.8	90.9	96.0	99.0	95.8	96.3	95.6	92.8	91.8	92.8	95.8	95.8	94.8	95.8	95.8	2	A4LPM8_BURPS_Bucl1_Bps 305.seq
3	2.0	2.0		90.9	95.3	89.7	99.7	80.3	79.1	82.2	86.7	94.3	83.5	89.6	97.0	92.8	96.8	99.2	97.0	96.5	97.1	93.9	92.1	93.9	97.0	97.0	95.5	97.0	97.0	3	A3P738_BURP0_Bucl1_Bps 1106a.seq
4	2.3	3.1	3.7		91.4	86.2	90.6	86.2	85.0	79.3	83.3	92.1	80.6	86.0	89.6	85.0	89.7	91.4	89.6	94.4	89.4	86.5	89.2	86.5	89.6	89.6	91.6	89.6	89.6	4	C4I6P3_BURPS_Bucl1_Bps MSHR346.seq
5	2.2	2.6	1.9	3.2		92.4	94.9	83.2	82.0	85.0	89.6	98.7	86.4	92.3	93.4	89.2	93.6	95.3	93.4	97.0	93.6	90.4	94.8	90.4	93.4	93.4	99.8	93.4	93.4	5	Bucl1_Bps 1026b.seq
6	2.0	2.2	1.4	2.3	1.8		89.4	89.4	88.2	91.2	95.6	91.8	92.6	99.5	88.2	83.7	88.2	89.7	88.2	91.1	88.2	85.2	95.3	85.2	88.2	88.2	92.6	88.2	88.2	6	Bucl1_Bps 1710b.seq
7	2.0	2.0	0.0	3.8	1.9	1.4		80.0	78.8	81.8	86.4	93.9	83.2	89.2	96.6	92.4	96.5	98.8	96.6	96.1	96.8	93.6	91.8	93.6	96.6	96.6	95.1	96.6	96.6	7	Bucl1_Bps BPC006.seq
8	2.7	2.3	2.3	2.3	2.5	2.3	2.3		96.3	90.7	92.1	83.2	91.9	89.2	78.8	74.2	78.8	80.3	78.8	81.8	78.8	75.8	86.0	75.8	78.8	78.8	83.3	78.8	78.8	8	Bucl1_Bps 668.seq
9	2.1	2.3	1.8	1.8	2.1	1.8	1.8	3.0		92.4	91.1	81.8	94.9	88.0	77.6	73.1	77.6	79.1	77.6	80.6	77.6	74.6	85.0	74.6	77.6	77.6	82.2	77.6	77.6	9	Bucl1_Bps NCTC 13179.seq
10	1.8	2.0	1.5	1.6	1.8	1.5	1.5	2.6	2.3		94.4	84.8	97.1	91.1	80.6	76.1	80.6	82.2	80.6	83.7	80.6	77.6	88.2	77.6	80.6	80.6	85.2	80.6	80.6	10	Bucl1_Bps MSHR146.seq
11	2.1	1.9	1.5	2.2	1.7	1.7	1.5	2.7	2.1	1.3		88.7	95.8	95.6	85.2	80.6	85.2	86.7	85.2	88.2	85.2	82.2	92.3	82.2	85.2	85.2	89.7	85.2	85.2	11	Bucl1_Bps MSHR511.seq
12	2.6	2.3	3.0	2.4	1.5	2.6	3.0	2.5	2.3	2.0	2.7		86.2	91.6	92.4	88.2	92.6	94.3	92.4	96.0	92.6	89.4	95.1	89.4	92.4	92.4	98.8	92.4	92.4	12	Bucl1_Bps MSHR520.seq
13	2.0	2.2	1.7	1.8	2.0	1.7	1.7	3.0	0.9	1.8	1.5	2.2		92.4	82.0	77.4	82.0	83.5	82.0	85.0	82.0	79.0	89.4	79.0	82.0	82.0	86.5	82.0	82.0	13	Bucl1_Bps NAU20B-16.seq
14	2.2	2.4	1.6	2.6	2.0	0.6	1.6	2.5	2.1	1.8	1.7	2.8	2.0		88.0	83.5	88.0	89.6	88.0	90.9	88.0	85.0	95.1	85.0	88.0	88.0	92.4	88.0	88.0	14	Bucl1_Bps NCTC 13178.seq
15	2.0	2.9	1.6	3.5	2.3	1.4	1.6	2.3	1.8	1.5	1.5	3.4	1.7	1.6		93.8	99.8	96.8	100.0	95.1	99.8	95.6	90.6	95.6	100.0	100.0	93.6	100.0	100.0	15	Bucl1_Bm 2000031281.seq
16	2.0	3.3	1.3	3.5	1.9	1.4	1.3	2.3	1.8	1.5	1.5	3.0	1.7	1.6	1.8		93.6	91.9	93.8	90.6	93.9	96.8	86.0	96.8	93.8	93.8	89.4	93.8	93.8	16	Bucl1_Bm 2002721280.seq
17	2.0	2.7	1.8	3.3	2.1	1.4	1.8	2.3	1.8	1.5	1.5	3.2	1.7	1.6	0.2	2.0		97.0	99.8	95.3	99.7	95.5	90.4	95.5	99.8	99.8	93.8	99.8	99.8	17	Bucl1_Bm A188.seq
18	2.2	1.1	0.9	3.1	1.9	1.4	0.9	2.3	1.8	1.5	1.5	3.0	1.7	1.6	1.8	2.2	1.6		96.8	97.0	96.6	93.8	91.8	93.8	96.8	96.8	95.5	96.8	96.8	18	Bucl1_Bm A193.seq
19	2.0	2.9	1.6	3.5	2.3	1.4	1.6	2.3	1.8	1.5	1.5	3.4	1.7	1.6	0.0	1.8	0.2	1.8		95.1	99.8	95.6	90.6	95.6	100.0	100.0	93.6	100.0	100.0	19	Bucl1_Bm China 7.seq
20	2.4	2.4	2.2	1.4	1.7	1.6	2.2	2.3	1.8	1.5	1.5	2.8	1.7	1.8	2.0	2.0	1.8	1.7	2.0		94.9	92.1	93.4	92.1	95.1	95.1	97.1	95.1	95.1	20	Bucl1_Bm PRL-20.seq
21	2.0	3.1	1.4	3.8	2.1	1.4	1.5	2.3	1.8	1.5	1.5	3.2	1.7	1.6	0.2	1.6	0.4	2.0	0.2	2.2		95.5	90.6	95.5	99.8	99.8	93.8	99.8	99.8	21	Bucl1_Bm strain_6.seq
22	2.0	2.9	1.6	3.5	2.3	1.4	1.6	2.3	1.8	1.5	1.5	3.4	1.7	1.6	1.4	1.7	1.6	1.8	1.4	2.0	1.6		87.5	100.0	95.6	95.6	90.6	95.6	95.6	22	Bucl1_Bm strain_11.seq
23	2.0	2.5	2.1	2.3	2.5	2.0	2.2	2.7	2.1	1.5	2.1	2.1	2.0	2.2	2.1	2.1	2.3	2.5	2.1	2.3	2.1	2.1		87.5	90.6	90.6	94.9	90.6	90.6	23	A2S1L2_BURM9_Bucl1_Bm NCTC 10229.seq
24	2.0	2.9	1.6	3.5	2.3	1.4	1.6	2.3	1.8	1.5	1.5	3.4	1.7	1.6	1.4	1.7	1.6	1.8	1.4	2.0	1.6	0.0	2.1		95.6	95.6	90.6	95.6	95.6	24	A3MCC9_BURM7_Bucl1_Bm NCTC 10247.seq
25	2.0	2.9	1.6	3.5	2.3	1.4	1.6	2.3	1.8	1.5	1.5	3.4	1.7	1.6	0.0	1.8	0.2	1.8	0.0	2.0	0.2	1.4	2.1	1.4		100.0	93.6	100.0	100.0	25	A5J478_BURMA_Bucl1_Bm FMH.seq
26	2.0	2.9	1.6	3.5	2.3	1.4	1.6	2.3	1.8	1.5	1.5	3.4	1.7	1.6	0.0	1.8	0.2	1.8	0.0	2.0	0.2	1.4	2.1	1.4	0.0		93.6	100.0	100.0	26	A5XL93_BURMA_Bucl1_Bm JHU.seq
27	2.4	2.5	1.7	3.0	0.2	1.6	1.7	2.3	1.8	1.5	1.5	1.3	1.7	1.8	2.1	1.7	1.9	1.7	2.1	1.5	1.9	2.1	2.3	2.1	2.1	2.1		93.6	93.6	27	A9K4Y8_BURMA_Bucl1_Bm ATCC 10399.seq
28	2.0	2.9	1.6	3.5	2.3	1.4	1.6	2.3	1.8	1.5	1.5	3.4	1.7	1.6	0.0	1.8	0.2	1.8	0.0	2.0	0.2	1.4	2.1	1.4	0.0	0.0	2.1		100.0	28	C4AZF6_BURMA_Bucl1_Bm GB8 horse 4.seq
29	2.0	2.9	1.6	3.5	2.3	1.4	1.6	2.3	1.8	1.5	1.5	3.4	1.7	1.6	0.0	1.8	0.2	1.8	0.0	2.0	0.2	1.4	2.1	1.4	0.0	0.0	2.1	0.0		29	Q62AX7_BURMA_Bucl1_Bm ATCC 23344.seq
	1	2	3	4	5	6	7	8	9	10	11	12	13	14	15	16	17	18	19	20	21	22	23	24	25	26	27	28	29		

bucl1

Percent Identity

	1	2	3	4	5	6	7	8	9	10	11	12	13	14	15	16	17	18	19	20	21	22	23	24	25	26	27		
**1**		88.1	91.8	96.5	93.0	90.0	90.7	79.6	95.1	96.9	88.1	96.9	85.9	95.8	94.0	96.8	84.4	84.4	84.4	83.1	83.1	84.4	84.4	88.9	84.4	84.4	83.1	**1**	Bud2_Bps K96243.seq
**2**	4.7		95.6	87.2	93.7	93.9	81.4	86.9	91.4	89.2	100.0	89.2	95.3	85.9	84.9	87.5	91.8	91.8	91.8	91.4	91.4	91.8	91.8	95.1	91.8	91.8	91.4	**2**	Bud2_Bps MSHR305.seq
**3**	1.8	3.4		91.3	97.1	96.8	84.6	86.5	94.6	93.0	95.6	93.0	92.9	89.1	88.1	90.8	91.4	91.4	91.4	90.0	90.0	91.4	91.4	95.9	91.4	91.4	90.0	**3**	Bud2_Bps 1106a.seq
**4**	2.3	4.2	1.0		91.3	88.9	91.8	78.2	93.3	94.9	87.2	94.9	84.7	96.1	94.6	97.7	83.3	83.3	83.3	82.0	82.0	83.3	83.3	87.8	83.3	83.3	82.0	**4**	Bud2_Bps 1026b.seq
**5**	1.8	4.3	1.7	2.3		94.8	85.7	85.3	94.9	94.5	93.7	94.5	91.4	89.7	89.5	92.4	90.0	90.0	90.0	88.8	88.8	90.0	90.0	94.5	90.0	90.0	88.8	**5**	Bud2_Bps 1710b.seq
**6**	3.9	5.8	3.6	3.6	4.3		83.0	86.5	92.1	90.7	93.9	90.7	92.9	86.6	85.7	88.6	91.4	91.4	91.4	90.0	90.0	91.4	91.4	95.5	91.4	91.4	90.0	**6**	Bud2_Bps BPC006.seq
**7**	4.3	6.7	4.1	4.4	4.4	5.9		74.1	87.3	89.4	81.4	89.4	79.3	92.0	94.9	92.1	78.2	78.2	78.2	77.0	77.0	78.2	78.2	81.7	78.2	78.2	77.0	**7**	A3NN74_BURP6_Bucl2_Bp 668.seq
**8**	0.8	1.8	0.4	1.0	0.4	0.6	1.4		82.2	80.9	86.9	80.9	89.7	77.4	76.1	78.7	92.9	92.9	92.9	93.9	93.9	92.9	92.9	88.9	92.9	92.9	93.9	**8**	Bud2_Bps NCTC 13179.seq
**9**	2.4	4.0	1.5	2.9	2.8	4.3	5.5	1.0		96.2	91.4	96.2	89.1	92.3	91.4	93.9	87.6	87.6	87.6	85.7	85.7	87.6	87.6	92.3	87.6	87.6	85.7	**9**	Bud2_Bps MSHR146.seq
**10**	1.7	5.1	1.8	2.5	1.8	4.4	4.5	1.0	2.7		89.2	100.0	87.2	93.3	92.1	95.9	85.7	85.7	85.7	84.4	84.4	85.7	85.7	90.2	85.7	85.7	84.4	**10**	Bud2_Bps MSHR511.seq
**11**	4.7	0.0	3.4	4.2	4.3	5.8	6.7	1.8	4.0	5.1		89.2	95.3	85.9	84.9	87.5	91.8	91.8	91.8	91.4	91.4	91.8	91.8	95.1	91.8	91.8	91.4	**11**	Bud2_Bps MSHR520.seq
**12**	1.7	5.1	1.8	2.5	1.8	4.4	4.5	1.0	2.7	0.0	5.1		87.2	93.3	92.1	95.9	85.7	85.7	85.7	84.4	84.4	85.7	85.7	90.2	85.7	85.7	84.4	**12**	Bud2_Bps NAU20B-16.seq
**13**	4.3	2.5	3.7	4.1	4.1	3.9	6.2	1.6	3.7	4.5	2.5	4.5		84.4	82.4	85.2	95.9	95.9	95.9	94.9	94.9	95.9	95.9	95.8	95.9	95.9	94.9	**13**	Bud2_Bps NCTC 13178.seq
**14**	1.6	4.3	1.8	2.6	2.8	4.6	5.8	0.4	2.7	3.0	4.3	3.0	3.0		96.5	96.1	82.8	82.8	82.8	81.5	81.5	82.8	82.8	86.0	82.8	82.8	81.5	**14**	B7CQL7_BURPS_Bucl2_Bps 576.seq
**15**	2.0	4.0	1.5	2.8	1.5	4.1	4.0	0.4	2.3	2.9	4.0	2.9	3.9	2.3		95.5	80.9	80.9	80.9	79.6	79.6	80.9	80.9	84.9	80.9	80.9	79.6	**15**	C0Y6X0_BURPS_Bucl2_Bps Pakistan 9.seq
**16**	1.9	4.0	1.3	2.3	1.1	3.7	4.2	0.4	2.4	1.6	4.0	1.6	3.7	2.8	2.0		83.6	83.6	83.6	82.2	82.2	83.6	83.6	88.2	83.6	83.6	82.2	**16**	A8EN62_BURPS_Bucl2_Bp 406e.seq
**17**	2.9	3.7	2.4	2.7	2.7	2.5	4.4	0.8	2.4	3.1	3.7	3.1	1.8	1.8	2.5	2.5		100.0	100.0	97.2	97.2	100.0	100.0	94.8	100.0	100.0	97.2	**17**	Bud2_Bm 2000031281.seq
**18**	2.9	3.7	2.4	2.7	2.7	2.5	4.4	0.8	2.4	3.1	3.7	3.1	1.8	1.8	2.5	2.5	0.0		100.0	97.2	97.2	100.0	100.0	94.8	100.0	100.0	97.2	**18**	Bud2_Bm 2002721280.seq
**19**	2.9	3.7	2.4	2.7	2.7	2.5	4.4	0.8	2.4	3.1	3.7	3.1	1.8	1.8	2.5	2.5	0.0	0.0		97.2	97.2	100.0	100.0	94.8	100.0	100.0	97.2	**19**	Bud2_Bm China 7.seq
**20**	3.0	2.6	2.6	2.8	2.6	2.8	4.3	1.2	3.2	3.2	2.6	3.2	1.5	1.8	2.6	2.6	1.8	1.8	1.8		100.0	97.2	97.2	93.0	97.2	97.2	100.0	**20**	Bud2_Bm strain_11.seq
**21**	3.0	2.6	2.6	2.8	2.6	2.8	4.3	1.2	3.2	3.2	2.6	3.2	1.5	1.8	2.6	2.6	1.8	1.8	1.8	0.0		97.2	97.2	93.0	97.2	97.2	100.0	**21**	Bud2_Bm NCTC 10247.seq
**22**	2.9	3.7	2.4	2.7	2.7	2.5	4.4	0.8	2.4	3.1	3.7	3.1	1.8	1.8	2.5	2.5	0.0	0.0	0.0	1.8	1.8		100.0	94.8	100.0	100.0	97.2	**22**	Bud2_Bm FMH.seq
**23**	2.9	3.7	2.4	2.7	2.7	2.5	4.4	0.8	2.4	3.1	3.7	3.1	1.8	1.8	2.5	2.5	0.0	0.0	0.0	1.8	1.8	0.0		94.8	100.0	100.0	97.2	**23**	Bud2_Bm JHU.seq
**24**	2.1	4.4	1.6	1.9	1.9	2.3	4.8	0.8	1.4	2.2	4.4	2.2	3.5	2.6	2.4	1.5	1.6	1.6	1.6	2.2	2.2	1.6	1.6		94.8	94.8	93.0	**24**	Bud2_Bm ATCC 10399.seq
**25**	2.9	3.7	2.4	2.7	2.7	2.5	4.4	0.8	2.4	3.1	3.7	3.1	1.8	1.8	2.5	2.5	0.0	0.0	0.0	1.8	1.8	0.0	0.0	1.6		100.0	97.2	**25**	Bud2_Bm GB8 horse 4.seq
**26**	2.9	3.7	2.4	2.7	2.7	2.5	4.4	0.8	2.4	3.1	3.7	3.1	1.8	1.8	2.5	2.5	0.0	0.0	0.0	1.8	1.8	0.0	0.0	1.6	0.0		97.2	**26**	Bud2_Bm ATCC 23344.seq
**27**	3.0	2.6	2.6	2.8	2.6	2.8	4.3	1.2	3.2	3.2	2.6	3.2	1.5	1.8	2.6	2.6	1.8	1.8	1.8	0.0	0.0	1.8	1.8	2.2	1.8	1.8		**27**	Bud2_Bm SAVP1.seq
	1	2	3	4	5	6	7	8	9	10	11	12	13	14	15	16	17	18	19	20	21	22	23	24	25	26	27		

bucl2

Percent Identity

	1	2	3	4	5	6	7	8	9	10	11	12	13	14	15	16	17	18	19	20	21	22	23	24	25	26	27	28	29	30	31	32	33	34	35	36		
1		97.2	92.8	93.9	97.4	92.8	91.7	94.1	95.6	95.6	97.2	95.6	96.6	96.7	98.6	96.7	95.8	98.6	97.7	97.7	97.7	96.7	96.7	97.2	96.7	96.7	97.2	71.2	68.8	71.2	71.2	74.5	70.6	70.7	67.1	70.2	1	Q63IU2_BURPS_Bucl3_Bps K96243.seq
2	0.4		94.9	96.3	99.7	94.9	93.9	96.1	97.7	97.7	99.8	97.7	98.6	94.4	96.3	94.4	93.5	96.3	95.3	95.3	95.3	94.4	94.4	94.9	94.4	94.4	94.9	72.9	70.6	72.9	72.9	76.2	72.3	72.4	68.8	72.0	2	Bucl3_Bps MSHR 305.seq
3	0.0	0.3		91.6	95.0	99.8	95.8	98.3	97.0	97.0	94.9	97.0	94.2	94.4	91.6	94.4	95.3	91.6	93.5	93.5	93.5	94.4	94.4	93.9	94.4	94.4	93.9	70.7	67.9	70.7	70.7	72.1	69.2	68.1	69.2	70.7	3	Bucl3_Bps 1106a.seq
4	0.4	0.4	0.4		96.4	91.6	90.7	92.8	94.4	94.4	96.3	94.4	97.2	91.1	93.9	91.1	90.2	93.9	92.1	92.1	92.1	91.1	91.1	91.6	91.1	91.1	91.6	72.9	70.6	72.9	72.9	76.2	72.3	75.7	68.8	72.0	4	Bucl3_Bps 1026b.seq
5	0.2	0.2	0.2	0.2		95.0	94.1	96.3	97.8	97.8	99.7	97.8	98.8	94.5	96.4	94.5	93.6	96.4	95.5	95.5	95.5	94.5	94.5	95.0	94.5	94.5	95.0	73.1	70.7	73.1	73.1	76.3	72.4	72.6	69.0	72.1	5	Q3JJL4_BURP1_Bucl3_Bps 1710b.seq
6	0.0	0.3	0.0	0.4	0.2		95.8	98.3	97.0	97.0	94.9	97.0	94.2	94.4	91.6	94.4	95.3	91.6	93.5	93.5	93.5	94.4	94.4	93.9	94.4	94.4	93.9	70.7	67.9	70.7	70.7	72.1	69.2	68.1	69.2	70.7	6	Bucl3_BPC006.seq
7	0.2	0.3	0.3	0.4	0.2	0.3		97.0	95.8	95.8	93.9	95.8	93.0	93.6	90.8	93.6	94.5	90.8	92.7	92.7	92.7	93.6	93.6	93.1	93.6	93.6	93.1	69.8	67.0	69.8	69.8	71.2	72.7	67.1	71.3	69.8	7	Bucl3_Bps 668.seq
8	0.2	0.5	0.2	0.5	0.3	0.2	0.5		98.3	98.3	96.1	98.3	95.5	95.6	92.8	95.6	96.6	92.8	94.7	94.7	94.7	95.6	95.6	95.2	95.6	95.6	95.2	72.0	69.2	72.0	72.0	73.4	70.4	69.3	69.0	72.0	8	Bucl3_Bps NCTC 13179.seq
9	0.0	0.3	0.0	0.4	0.2	0.0	0.3	0.2		99.8	97.7	99.8	97.0	96.3	94.4	96.3	95.3	94.4	96.3	96.3	96.3	96.3	96.3	96.7	96.3	96.3	96.7	73.2	70.7	73.2	73.2	74.9	72.0	70.9	69.2	72.3	9	Bucl3_Bps MSHR146.seq
10	0.0	0.3	0.0	0.4	0.2	0.0	0.3	0.2	0.0		97.7	99.8	97.0	96.3	94.4	96.3	95.3	94.4	96.3	96.3	96.3	96.3	96.3	96.7	96.3	96.3	96.7	73.2	70.7	73.2	73.2	74.9	72.0	70.9	69.2	72.3	10	Bucl3_Bps MSHR511.seq
11	0.4	0.0	0.3	0.4	0.2	0.3	0.3	0.5	0.3	0.3		97.7	98.6	94.4	96.3	94.4	93.5	96.3	95.3	95.3	95.3	94.4	94.4	94.9	94.4	94.4	94.9	72.9	70.6	72.9	72.9	76.2	72.3	72.4	68.8	72.0	11	Bucl3_Bps MSHR520.seq
12	0.0	0.3	0.0	0.4	0.2	0.0	0.3	0.2	0.0	0.0	0.3		97.0	96.3	94.4	96.3	95.3	94.4	96.3	96.3	96.3	96.3	96.3	96.7	96.3	96.3	96.7	73.2	70.7	73.2	73.2	74.9	72.0	70.9	69.2	72.3	12	Bucl3_Bps NAU20B-16.seq
13	0.0	0.3	0.0	0.4	0.2	0.0	0.3	0.2	0.0	0.0	0.3	0.0		93.5	96.3	93.5	92.5	96.3	94.4	94.4	94.4	93.5	93.5	93.9	93.5	93.5	93.9	73.2	70.9	73.2	73.2	76.5	72.6	73.7	69.2	72.3	13	Bucl3_Bps NCTC 13178.seq
14	0.4	0.4	0.3	0.4	0.2	0.3	0.2	0.5	0.3	0.3	0.4	0.3	0.4		97.0	99.8	98.9	97.0	98.9	98.9	98.9	99.8	99.8	99.4	99.8	99.8	99.4	70.2	67.4	70.2	70.2	71.7	68.7	67.6	66.4	69.5	14	Bucl3_Bm 2000031281.seq
15	0.4	0.4	0.4	0.4	0.2	0.4	0.2	0.5	0.4	0.4	0.4	0.4	0.4	0.0		97.0	96.1	99.8	98.0	98.0	98.0	97.0	97.0	97.5	97.0	97.0	97.5	70.4	68.1	70.4	70.4	73.7	69.8	70.4	66.4	69.5	15	Bucl3_Bm 2002721280.seq
16	0.4	0.4	0.3	0.4	0.2	0.3	0.2	0.5	0.3	0.3	0.4	0.3	0.4	0.0	0.0		98.9	97.0	98.9	98.9	98.9	99.8	99.8	99.4	99.8	99.8	99.4	70.2	67.4	70.2	70.2	71.7	68.7	67.6	66.4	69.5	16	Bucl3_Bm China 7.seq
17	0.4	0.4	0.3	0.4	0.2	0.3	0.2	0.5	0.3	0.3	0.4	0.3	0.4	0.0	0.0	0.0		96.1	98.0	98.0	98.0	98.9	98.9	98.4	98.9	98.9	98.4	69.3	66.5	69.3	69.3	70.7	67.8	66.7	66.4	69.3	17	Bucl3_Bm PRL-20.seq
18	0.4	0.4	0.4	0.4	0.2	0.4	0.2	0.5	0.4	0.4	0.4	0.4	0.4	0.0	0.0	0.0	0.0		98.0	98.0	98.0	97.0	97.0	97.5	97.0	97.0	97.5	70.4	68.1	70.4	70.4	73.7	69.8	70.4	66.4	69.5	18	Bucl3_Bm strain_6.seq
19	0.4	0.4	0.3	0.4	0.2	0.3	0.2	0.5	0.3	0.3	0.4	0.3	0.4	0.0	0.0	0.0	0.0	0.0		99.8	99.8	98.9	98.9	99.4	98.9	98.9	99.4	70.4	68.1	70.4	70.4	72.6	69.6	68.5	66.4	69.5	19	Bucl3_Bm strain_11.seq
20	0.4	0.4	0.3	0.4	0.2	0.3	0.2	0.5	0.3	0.3	0.4	0.3	0.4	0.0	0.0	0.0	0.0	0.0	0.0		99.8	98.9	98.9	99.4	98.9	98.9	99.4	70.4	68.1	70.4	70.4	72.6	69.6	68.5	66.4	69.5	20	Bucl3_Bm NCTC 10229.seq
21	0.4	0.4	0.3	0.4	0.2	0.3	0.2	0.5	0.3	0.3	0.4	0.3	0.4	0.0	0.0	0.0	0.0	0.0	0.0	0.0		98.9	98.9	99.4	98.9	98.9	99.4	70.4	68.1	70.4	70.4	72.6	69.6	68.5	66.4	69.5	21	Bucl3_Bm NCTC 10247.seq
22	0.4	0.4	0.3	0.4	0.2	0.3	0.2	0.5	0.3	0.3	0.4	0.3	0.4	0.0	0.0	0.0	0.0	0.0	0.0	0.0	0.0		99.8	99.4	99.8	99.8	99.4	70.2	67.4	70.2	70.2	71.7	68.7	67.6	66.4	69.5	22	Bucl3_Bm FMH.seq
23	0.4	0.4	0.3	0.4	0.2	0.3	0.2	0.5	0.3	0.3	0.4	0.3	0.4	0.0	0.0	0.0	0.0	0.0	0.0	0.0	0.0	0.0		99.4	99.8	99.8	99.4	70.2	67.4	70.2	70.2	71.7	68.7	67.6	66.4	69.5	23	Bucl3_Bm JHU.seq
24	0.4	0.4	0.3	0.4	0.2	0.3	0.2	0.5	0.3	0.3	0.4	0.3	0.4	0.0	0.0	0.0	0.0	0.0	0.0	0.0	0.0	0.0	0.0		99.4	99.4	99.8	70.4	67.9	70.4	70.4	72.1	69.2	68.1	66.4	69.5	24	Bucl3_Bm ATCC 10399.seq
25	0.4	0.4	0.3	0.4	0.2	0.3	0.2	0.5	0.3	0.3	0.4	0.3	0.4	0.0	0.0	0.0	0.0	0.0	0.0	0.0	0.0	0.0	0.0	0.0		99.8	99.4	70.2	67.4	70.2	70.2	71.7	68.7	67.6	66.4	69.5	25	Bucl3_Bm GB8 horse 4.seq
26	0.4	0.4	0.3	0.4	0.2	0.3	0.2	0.5	0.3	0.3	0.4	0.3	0.4	0.0	0.0	0.0	0.0	0.0	0.0	0.0	0.0	0.0	0.0	0.0	0.0		99.4	70.2	67.4	70.2	70.2	71.7	68.7	67.6	66.4	69.5	26	Q62EE4_BURMA_Bucl3_ATCC 23344.seq
27	0.4	0.4	0.3	0.4	0.2	0.3	0.2	0.5	0.3	0.3	0.4	0.3	0.4	0.0	0.0	0.0	0.0	0.0	0.0	0.0	0.0	0.0	0.0	0.0	0.0	0.0		70.4	67.9	70.4	70.4	72.1	69.2	68.1	66.4	69.5	27	Bucl3_Bm SAVP1.seq
28	33.6	33.6	35.8	29.5	33.4	35.8	35.8	36.0	35.4	35.4	33.6	35.4	31.9	37.2	33.8	37.2	37.2	33.8	36.2	36.2	36.2	37.2	37.2	36.8	37.2	37.2	36.8		91.6	99.8	99.8	96.6	95.8	84.3	91.7	98.9	28	Bucl3_Bt E264.seq
29	38.4	38.2	39.8	34.1	38.0	39.8	39.8	40.1	39.8	39.8	38.2	39.8	36.5	41.4	38.6	41.4	41.4	38.6	41.0	41.0	41.0	41.4	41.4	41.4	41.4	41.4	41.4	8.7		91.6	91.6	89.6	88.8	91.7	83.8	90.7	29	Bucl3_Bt MSMB121.seq
30	33.6	33.6	35.8	29.5	33.4	35.8	35.8	36.0	35.4	35.4	33.6	35.4	31.9	37.2	33.8	37.2	37.2	33.8	36.2	36.2	36.2	37.2	37.2	36.8	37.2	37.2	36.8	0.0	8.7		99.8	96.6	95.8	84.3	91.7	98.9	30	Bucl3_Bt 2002721723.seq
31	33.6	33.6	35.8	29.5	33.4	35.8	35.8	36.0	35.4	35.4	33.6	35.4	31.9	37.2	33.8	37.2	37.2	33.8	36.2	36.2	36.2	37.2	37.2	36.8	37.2	37.2	36.8	0.0	8.7	0.0		96.6	95.8	84.3	91.7	98.9	31	Bucl3_Bt E444.seq
32	31.2	31.3	31.1	27.2	31.0	31.1	31.1	31.4	31.1	31.1	31.3	31.1	29.6	32.4	31.4	32.4	32.4	31.4	32.4	32.4	32.4	32.4	32.4	32.4	32.4	32.4	32.4	1.5	10.2	1.5	1.5		96.0	85.0	91.3	95.6	32	Bucl3_Bt E555.seq
33	32.3	32.3	33.3	28.2	32.1	33.3	32.6	33.6	33.3	33.3	32.3	33.3	30.6	34.6	32.4	34.6	34.6	32.4	34.6	34.6	34.6	34.6	34.6	34.6	34.6	34.6	34.6	0.8	9.3	0.8	0.8	0.7		82.4	93.9	94.9	33	Bucl3_Bt H0587.seq
34	30.5	30.6	30.0	30.6	30.3	30.0	30.0	30.3	30.0	30.0	30.6	30.0	30.0	31.3	31.3	31.3	31.3	31.3	31.3	31.3	31.3	31.3	31.3	31.3	31.3	31.3	31.3	9.3	0.7	9.3	9.3	10.8	9.9		76.5	83.3	34	Bucl3_Bt MSMB43.seq
35	30.2	30.3	35.4	26.2	30.0	35.4	32.9	34.0	32.0	32.0	30.3	32.0	28.6	33.8	30.3	33.8	35.0	30.3	32.7	32.7	32.7	33.8	33.8	33.3	33.8	33.8	33.3	2.2	10.8	2.2	2.2	0.7	1.3	11.6		92.7	35	Bucl3_Bt TXDOH.seq
36	33.6	33.6	36.9	29.5	33.4	36.9	36.9	37.2	35.4	35.4	33.6	35.4	31.9	37.4	33.8	37.4	38.4	33.8	36.2	36.2	36.2	37.4	37.4	36.8	37.4	37.4	36.8	0.0	8.7	0.0	0.0	1.5	0.8	9.3	2.2		36	Bucl3_Bt 4.seq
	1	2	3	4	5	6	7	8	9	10	11	12	13	14	15	16	17	18	19	20	21	22	23	24	25	26	27	28	29	30	31	32	33	34	35	36		

bucl3

Percent Identity

	1	2	3	4	5	6	7	8	9	10	11	12	13	14	15	16	17	18	19	20	21	22	23	24	25	26	27	28	29	30	31	32	33	34		
**1**		90.5	93.5	92.0	96.1	93.5	93.9	95.5	95.5	90.0	90.5	83.4	98.6	95.5	92.0	96.7	97.3	98.4	96.8	96.7	99.1	99.1	94.8	93.1	96.7	96.7	98.3	96.7	96.7	79.8	79.1	81.0	80.5	80.1	**1**	Q63JT5_BURPS_Bps_K96243.seq
**2**	1.1		96.0	97.6	93.6	96.0	95.8	93.7	87.3	80.8	100.0	80.1	89.4	86.4	83.3	93.0	92.2	89.7	88.1	93.0	90.5	90.5	94.8	85.0	93.0	93.0	91.3	93.0	93.0	77.6	88.0	80.4	82.0	84.8	**2**	Bucl4_Bps MSHR 305.seq
**3**	1.1	1.1		97.9	96.4	100.0	99.6	96.7	90.4	83.8	96.0	81.6	92.7	89.8	86.9	96.4	95.5	92.6	90.9	96.4	93.3	93.5	97.5	87.8	96.4	96.4	94.5	96.4	96.4	78.2	84.5	80.9	82.5	85.3	**3**	Bucl4_Bps 1106a.seq
**4**	1.1	1.0	0.6		95.0	97.9	97.6	95.6	89.2	82.5	97.6	81.2	91.3	88.5	85.4	94.8	93.9	91.1	89.5	94.8	91.9	91.9	96.2	86.4	94.8	94.8	92.8	94.8	94.8	77.9	86.2	80.7	82.2	85.0	**4**	Bucl4_Bps 1026b.seq
**5**	0.9	1.1	1.5	1.3		96.4	96.8	98.3	91.7	86.5	93.6	82.6	94.8	91.8	88.2	98.0	97.1	95.5	94.1	98.0	96.5	96.1	97.6	90.8	98.0	98.0	96.7	98.0	98.0	78.2	82.3	80.9	82.5	83.0	**5**	Bucl4_Bps 1710b.seq
**6**	1.1	1.1	0.0	0.6	1.5		99.6	96.7	90.4	83.8	96.0	81.6	92.7	89.8	86.9	96.4	95.5	92.6	90.9	96.4	93.3	93.5	97.5	87.8	96.4	96.4	94.5	96.4	96.4	78.2	84.5	80.9	82.5	85.3	**6**	Bucl4_Bps BPC006.seq
**7**	0.7	1.3	0.5	1.1	1.0	0.5		96.4	90.0	84.2	95.8	82.0	93.1	90.1	86.5	96.1	95.2	92.9	91.3	96.1	93.6	93.9	97.2	88.1	96.1	96.1	94.8	96.1	96.1	77.8	84.6	80.6	82.2	85.0	**7**	Bucl4_Bps 668.seq
**8**	1.5	1.0	1.1	0.5	1.9	1.1	1.5		92.8	85.7	93.7	82.2	94.6	91.8	88.6	98.2	97.4	94.5	92.7	98.2	95.2	95.5	98.4	89.2	98.2	98.2	96.4	98.2	98.2	79.4	82.5	81.9	83.5	84.1	**8**	Bucl4_Bps NCTC 13179.seq
**9**	1.6	1.1	1.1	0.6	2.3	1.1	1.6	1.0		92.5	87.3	83.6	96.2	98.1	95.5	93.2	93.7	95.6	97.0	93.2	94.8	94.9	91.9	94.8	93.2	93.2	94.4	93.2	93.2	81.3	76.0	78.7	78.1	77.8	**9**	Bucl4_Bps MSHR146.seq
**10**	0.6	1.4	1.5	1.2	1.0	1.5	1.0	2.0	1.3		80.8	83.9	90.5	93.8	96.9	86.7	87.3	90.5	91.8	86.7	89.6	89.4	84.9	94.1	86.7	86.7	88.5	86.7	86.7	80.0	69.4	71.3	70.8	70.4	**10**	Bucl4_Bps MSHR511.seq
**11**	1.1	0.0	1.1	1.0	1.1	1.1	1.3	1.0	1.1	1.4		80.1	89.4	86.4	83.3	93.0	92.2	89.7	88.1	93.0	90.5	90.5	94.8	85.0	93.0	93.0	91.3	93.0	93.0	77.6	88.0	80.4	82.0	84.8	**11**	Bucl4_Bps MSHR520.seq
**12**	10.0	3.7	5.7	4.3	7.3	5.7	5.2	7.8	13.3	16.7	3.7		83.9	84.3	84.8	82.7	82.9	83.6	84.1	82.7	83.4	83.6	81.8	84.8	82.7	82.7	83.4	82.7	82.7	82.1	70.6	73.9	73.2	72.6	**12**	Bucl4_Bps NAU20B-16.seq
**13**	0.7	1.4	1.1	1.1	1.4	1.1	0.7	1.7	1.7	1.0	1.4	10.2		96.1	92.7	95.7	96.4	98.8	97.2	95.7	98.1	98.1	94.0	93.2	95.7	95.7	97.4	95.7	95.7	80.2	78.1	80.1	79.7	79.4	**13**	Bucl4_Bps NCTC 13178.seq
**14**	0.6	1.3	0.9	0.6	1.3	0.9	0.5	1.3	1.2	0.7	1.3	13.3	0.9		96.2	92.6	93.1	95.7	97.0	92.6	94.8	95.0	90.6	96.4	92.6	92.6	94.5	92.6	92.6	80.5	75.0	77.3	76.7	76.4	**14**	B1H7D0_BURPS_Bps S13.seq
**15**	1.1	1.2	0.6	0.5	1.8	0.6	1.0	1.3	0.6	0.8	1.2	16.1	1.2	0.7		89.7	90.2	92.1	93.4	89.7	91.3	91.4	87.7	95.8	89.7	89.7	90.9	89.7	89.7	80.8	71.7	74.4	73.8	73.5	**15**	B2HC63_BURPS_Bps 1655.seq
**16**	1.1	0.9	0.5	0.6	1.4	0.5	0.9	1.2	1.5	1.7	0.9	8.1	1.3	1.3	1.0		99.0	96.0	94.2	100.0	96.7	96.8	97.8	90.6	100.0	100.0	97.7	100.0	100.0	79.3	81.5	81.8	83.1	82.9	**16**	Bucl4_Bm 2000031281.seq
**17**	1.3	0.9	0.6	0.7	1.5	0.6	1.0	1.2	1.8	1.9	0.9	8.7	1.4	1.6	1.3	0.2		96.7	94.9	99.0	97.3	97.6	97.2	91.2	99.0	99.0	98.5	99.0	99.0	79.3	80.7	81.8	82.2	82.1	**17**	A5TN55_BURMA_Bm_2002721280.seq
**18**	0.9	1.1	1.4	1.3	0.6	1.4	0.9	1.8	2.2	1.0	1.1	10.5	1.4	1.3	1.8	1.0	1.1		98.0	96.0	98.9	98.9	94.0	94.1	96.0	96.0	97.7	96.0	96.0	80.0	78.3	80.0	79.5	79.4	**18**	Bucl4_Bm A188.seq
**19**	1.0	1.1	1.5	1.3	0.5	1.5	1.0	2.0	2.4	1.2	1.1	11.8	1.4	1.5	2.0	1.2	1.3	0.5		94.2	97.5	97.1	92.3	95.8	94.2	94.2	96.0	94.2	94.2	79.9	76.7	78.2	77.9	77.6	**19**	Bucl4_Bm A193.seq
**20**	1.1	0.9	0.5	0.6	1.4	0.5	0.9	1.2	1.5	1.7	0.9	8.1	1.3	1.3	1.0	0.0	0.2	1.0	1.2		96.7	96.8	97.8	90.6	100.0	100.0	97.7	100.0	100.0	79.3	81.5	81.8	83.1	82.9	**20**	Bucl4_Bm China 7.seq
**21**	1.0	1.1	1.5	1.3	0.4	1.5	1.0	1.9	2.4	1.1	1.1	10.0	1.3	1.4	1.9	1.1	1.3	0.4	0.2	1.1		99.5	94.7	93.4	96.7	96.7	98.3	96.7	96.7	79.3	79.1	80.6	80.1	80.0	**21**	Bucl4_Bm PRL-20.seq
**22**	1.0	1.1	1.1	1.3	0.9	1.1	0.7	1.5	2.2	1.3	1.1	9.7	1.3	1.2	1.7	1.0	0.9	0.4	0.6	1.0	0.6		94.9	93.1	96.8	96.8	98.9	96.8	96.8	79.5	79.1	80.8	80.4	80.3	**22**	Bucl4_Bm strain_11.seq
**23**	1.4	0.6	1.1	0.8	2.0	1.1	1.5	0.9	1.2	2.1	0.6	7.3	1.5	1.7	1.5	0.7	0.5	1.5	1.6	0.7	1.6	1.3		89.0	97.8	97.8	95.9	97.8	97.8	79.0	83.4	81.5	83.1	84.7	**23**	A2S1C1_BURM9_Bm NCTC 10229.seq
**24**	1.6	1.1	1.5	1.3	0.6	1.5	1.0	2.5	3.1	2.1	1.1	14.4	2.3	2.2	2.8	1.8	2.0	1.4	1.1	1.8	1.2	1.6	1.8		90.6	90.6	92.2	90.6	90.6	79.2	73.6	74.5	74.1	74.3	**24**	Bucl4_Bm NCTC 10247.seq
**25**	1.1	0.9	0.5	0.6	1.4	0.5	0.9	1.2	1.5	1.7	0.9	8.1	1.3	1.3	1.0	0.0	0.2	1.0	1.2	0.0	1.1	1.0	0.7	1.8		100.0	97.7	100.0	100.0	79.3	81.5	81.8	83.1	82.9	**25**	Bucl4_Bm FMH.seq
**26**	1.1	0.9	0.5	0.6	1.4	0.5	0.9	1.2	1.5	1.7	0.9	8.1	1.3	1.3	1.0	0.0	0.2	1.0	1.2	0.0	1.1	1.0	0.7	1.8	0.0		97.7	100.0	100.0	79.3	81.5	81.8	83.1	82.9	**26**	A5XLG9_BURMA_Bm JHU.seq
**27**	1.1	1.1	0.9	1.1	1.1	0.9	0.5	1.4	1.9	1.4	1.1	9.0	1.2	0.9	1.4	0.8	0.8	0.8	1.0	0.8	1.0	0.4	1.1	1.7	0.8	0.8		97.7	97.7	79.5	79.9	81.7	81.4	81.2	**27**	A9K4R2_BURMA_Bm ATCC 10399.seq
**28**	1.1	0.9	0.5	0.6	1.4	0.5	0.9	1.2	1.5	1.7	0.9	8.1	1.3	1.3	1.0	0.0	0.2	1.0	1.2	0.0	1.1	1.0	0.7	1.8	0.0	0.0	0.8		100.0	79.3	81.5	81.8	83.1	82.9	**28**	C4AZL9_BURMA_Bm GB8 horse 4.seq
**29**	1.1	0.9	0.5	0.6	1.4	0.5	0.9	1.2	1.5	1.7	0.9	8.1	1.3	1.3	1.0	0.0	0.2	1.0	1.2	0.0	1.1	1.0	0.7	1.8	0.0	0.0	0.8	0.0		79.3	81.5	81.8	83.1	82.9	**29**	Q62AR6_BURMA_Bm ATCC 23344.seq
**30**	21.7	13.1	16.5	14.8	19.6	16.5	17.0	18.1	21.6	23.4	13.1	20.5	22.1	22.7	22.3	19.3	20.3	22.4	23.5	19.3	22.3	22.1	17.6	24.5	19.3	19.3	21.1	19.3	19.3		81.6	97.0	95.2	91.8	**30**	Bucl4_Bt E264.seq
**31**	12.3	11.9	12.4	12.3	12.1	12.4	12.3	11.9	12.1	12.7	11.9	12.7	12.5	12.5	12.7	12.3	12.3	12.3	12.3	12.3	12.3	12.3	11.7	12.3	12.3	12.3	12.3	12.3	12.3	6.5		86.3	87.8	90.8	**31**	Bucl4_Bt MSMB121.seq
**32**	20.5	13.2	16.6	14.9	19.6	16.6	17.1	18.1	19.3	20.9	13.2	18.8	20.7	20.3	19.9	19.3	20.3	20.9	21.2	19.3	21.2	20.9	17.7	22.1	19.3	19.3	20.7	19.3	19.3	0.0	6.8		98.4	95.5	**32**	Bucl4_Bt 2002721723.seq
**33**	19.3	13.2	16.6	14.9	19.6	16.6	17.1	18.1	18.2	19.7	13.2	17.9	19.3	19.1	18.7	18.9	19.2	19.6	19.7	18.9	19.9	19.5	17.7	20.7	18.9	18.9	19.2	18.9	18.9	0.0	6.8	0.0		97.0	**33**	Bucl4_Bt E444.seq
**34**	16.9	12.6	15.9	14.3	17.3	15.9	16.4	15.7	15.7	17.2	12.6	15.8	16.9	16.6	16.2	16.2	16.3	16.9	17.1	16.2	17.1	16.6	15.9	17.4	16.2	16.2	16.5	16.2	16.2	0.7	5.9	0.7	0.7		**34**	Bucl4_Bt H0587.seq
	**1**	**2**	**3**	**4**	**5**	**6**	**7**	**8**	**9**	**10**	**11**	**12**	**13**	**14**	**15**	**16**	**17**	**18**	**19**	**20**	**21**	**22**	**23**	**24**	**25**	**26**	**27**	**28**	**29**	**30**	**31**	**32**	**33**	**34**		

bucl4

Percent Identity

	1	2	3	4	5	6	7	8	9	10	11	12	13	14	15	16	17	18	19	20	21	22	23	24	25	26	27	28	29		
1		86.8	84.6	87.6	97.0	82.9	84.2	79.1	83.8	83.8	86.8	83.8	76.5	85.9	83.3	85.5	92.7	85.9	89.3	93.2	85.0	84.6	90.6	85.9	85.9	90.2	85.9	85.9	87.6	1	Bucl5_Bps K96243.seq
2	5.9		88.5	83.8	87.6	87.6	87.2	83.8	86.8	86.8	99.6	86.8	82.5	93.2	91.0	93.2	88.9	93.2	90.6	88.9	94.4	80.8	85.0	93.2	93.2	89.7	93.2	93.2	94.4	2	Bucl5_Bps MSHR305.seq
3	7.9	9.1		79.1	83.3	97.9	95.7	86.8	92.3	92.3	88.5	92.3	86.3	92.3	93.2	92.7	85.9	92.3	88.0	85.9	91.5	76.1	82.1	92.3	92.3	86.8	92.3	92.3	91.0	3	Bucl5_Bps 1106a.seq
4	8.8	3.8	9.2		89.3	79.9	79.5	73.9	78.2	78.2	83.8	78.2	77.8	82.5	78.2	82.5	87.6	82.5	85.5	87.2	82.1	85.9	92.7	82.5	82.5	86.8	82.5	82.5	84.6	4	Bucl5_Bps 1026b.seq
5	2.9	4.8	9.7	6.6		81.6	82.9	79.9	82.5	82.5	87.6	82.5	77.4	87.2	83.3	86.8	92.7	87.2	90.6	93.2	86.3	84.6	90.6	87.2	87.2	91.5	87.2	87.2	88.9	5	Q3JHM0_BURP1_Bucl5_Bp 1710b.seq
6	7.9	9.1	0.0	9.1	9.7		96.6	85.0	90.6	90.6	87.6	90.6	87.2	91.5	92.3	91.9	85.0	91.5	86.3	85.0	90.6	75.2	81.2	91.5	91.5	85.9	91.5	91.5	89.3	6	Bucl5_Bps BPC006.seq
7	6.7	9.1	3.3	10.3	8.5	3.2		85.0	90.2	90.2	87.2	90.2	86.3	89.3	89.3	89.7	82.9	89.3	85.0	82.9	88.5	73.1	79.1	89.3	89.3	83.8	89.3	89.3	88.0	7	Bucl5_Bps 668.seq
8	4.9	4.9	6.9	5.6	3.7	6.9	7.5		91.5	91.5	83.8	91.5	88.9	86.3	88.0	86.8	80.3	86.3	84.2	80.3	85.9	70.5	76.5	86.3	86.3	81.6	86.3	86.3	85.5	8	Bucl5_Bps NCTC 13179.seq
9	4.6	7.0	5.2	8.3	6.4	5.2	6.4	2.5		99.6	86.8	99.6	88.9	88.9	92.3	89.7	83.3	88.9	86.3	83.3	88.5	73.5	79.5	88.9	88.9	84.6	88.9	88.9	88.5	9	Bucl5_Bps MSHR146.seq
10	4.6	7.0	5.2	8.3	6.4	5.2	6.4	2.5	0.0		86.8	99.6	88.9	88.9	92.3	89.7	83.3	88.9	86.3	83.3	88.5	73.5	79.5	88.9	88.9	84.6	88.9	88.9	88.5	10	Bucl5_Bps MSHR511.seq
11	5.9	0.0	9.1	3.8	4.8	9.1	9.1	4.9	7.0	7.0		86.8	82.5	93.2	91.0	93.2	88.9	93.2	90.6	88.9	94.4	80.8	85.0	93.2	93.2	89.7	93.2	93.2	94.4	11	Bucl5_Bps MSHR520.seq
12	4.6	7.0	5.2	8.3	6.4	5.2	6.4	2.5	0.0	0.0	7.0		88.9	88.9	92.3	89.7	83.3	88.9	86.3	83.3	88.5	73.5	79.5	88.9	88.9	84.6	88.9	88.9	88.5	12	Bucl5_Bps NAU20B-16.seq
13	8.1	6.1	6.8	5.4	6.8	6.7	8.6	6.4	8.1	8.1	6.1	8.1		85.9	87.2	85.5	79.1	85.9	82.5	79.1	84.6	69.2	75.2	85.9	85.9	80.3	85.9	85.9	85.0	13	Bucl5_Bps NCTC 13178.seq
14	6.6	5.4	6.7	5.0	4.9	6.7	9.1	4.3	7.0	7.0	5.4	7.0	4.2		93.2	98.7	90.2	99.6	90.6	90.2	98.3	80.3	86.3	99.6	99.6	91.0	99.6	99.6	95.7	14	Bucl5_Bm 2000031281.seq
15	5.7	6.1	4.5	6.3	5.7	4.5	8.0	4.3	4.6	4.6	6.1	4.6	4.9	3.9		93.6	87.2	93.2	88.5	87.2	94.0	79.1	83.3	93.2	93.2	88.9	93.2	93.2	90.2	15	Bucl5_Bm 2002721280.seq
16	7.2	5.4	6.1	5.0	5.5	6.1	8.5	3.7	5.8	5.8	5.4	5.8	4.9	1.0	3.3		91.0	98.7	91.0	91.0	98.3	81.2	87.2	98.7	98.7	91.9	98.7	98.7	94.9	16	Bucl5_Bm A188.seq
17	5.6	5.9	6.7	6.2	5.6	6.7	9.1	3.7	5.8	5.8	5.9	5.8	4.9	3.7	3.3	2.7		90.2	91.9	98.3	89.7	87.2	92.7	90.2	90.2	96.2	90.2	90.2	89.7	17	Bucl5_Bm A193.seq
18	6.6	5.4	6.7	5.0	4.9	6.7	9.1	4.3	7.0	7.0	5.4	7.0	4.2	0.0	3.9	1.0	3.7		90.6	90.2	98.3	80.3	86.3	99.6	99.6	91.0	99.6	99.6	95.7	18	Bucl5_Bm China 7.seq
19	8.0	5.9	8.5	6.9	6.3	8.5	10.9	3.0	6.4	6.4	5.9	6.4	4.9	5.5	3.9	4.9	5.2	5.5		91.9	90.2	82.5	88.5	90.6	90.6	94.4	90.6	90.6	92.7	19	Bucl5_Bm PRL-20.seq
20	5.1	5.9	6.7	6.7	5.1	6.7	9.1	3.7	5.8	5.8	5.9	5.8	4.9	3.7	3.3	2.7	1.5	3.7	5.2		89.7	87.6	93.2	90.2	90.2	94.9	90.2	90.2	89.7	20	Bucl5_Bm strain_6.seq
21	6.6	4.8	6.7	4.4	4.9	6.7	9.1	3.7	6.4	6.4	4.8	6.4	4.9	0.5	3.8	0.5	3.2	0.5	4.9	3.2		81.6	85.9	98.3	98.3	90.6	98.3	98.3	94.4	21	Bucl5_Bm strain_11.seq
22	6.5	5.8	6.7	5.4	6.5	6.7	9.1	3.7	5.8	5.8	5.8	5.8	4.9	3.7	3.3	2.7	3.0	3.7	4.7	2.5	3.2		91.0	80.3	80.3	85.5	80.3	80.3	79.9	22	Bucl5_Bm NCTC 10229.seq
23	6.5	5.9	6.7	4.4	6.5	6.7	9.1	3.7	5.8	5.8	5.9	5.8	4.9	3.7	3.3	2.7	3.5	3.7	4.7	3.0	3.2	2.8		86.3	86.3	91.5	86.3	86.3	85.9	23	Bucl5_Bm NCTC 10247.seq
24	6.6	5.4	6.7	5.0	4.9	6.7	9.1	4.3	7.0	7.0	5.4	7.0	4.2	0.0	3.9	1.0	3.7	0.0	5.5	3.7	0.5	3.7	3.7		99.6	91.0	99.6	99.6	95.7	24	Bucl5_Bm FMH.seq
25	6.6	5.4	6.7	5.0	4.9	6.7	9.1	4.3	7.0	7.0	5.4	7.0	4.2	0.0	3.9	1.0	3.7	0.0	5.5	3.7	0.5	3.7	3.7	0.0		91.0	99.6	99.6	95.7	25	Bucl5_Bm JHU.seq
26	7.3	6.4	7.3	5.7	5.7	7.3	9.7	3.7	5.8	5.8	6.4	5.8	4.9	4.3	2.7	3.2	2.5	4.3	3.6	4.0	3.7	3.5	3.5	4.3	4.3		91.0	91.0	90.6	26	Bucl5_Bm ATCC 10399.seq
27	6.6	5.4	6.7	5.0	4.9	6.7	9.1	4.3	7.0	7.0	5.4	7.0	4.2	0.0	3.9	1.0	3.7	0.0	5.5	3.7	0.5	3.7	3.7	0.0	0.0	4.3		99.6	95.7	27	Bucl5_Bm GB8 horse 4.seq
28	6.6	5.4	6.7	5.0	4.9	6.7	9.1	4.3	7.0	7.0	5.4	7.0	4.2	0.0	3.9	1.0	3.7	0.0	5.5	3.7	0.5	3.7	3.7	0.0	0.0	4.3	0.0		95.7	28	Bucl5_Bm ATCC 23344.seq
29	7.1	4.3	7.9	4.9	5.4	7.9	10.3	4.9	7.0	7.0	4.3	7.0	4.9	2.1	5.1	3.2	4.8	2.1	5.4	4.8	2.7	4.8	4.8	2.1	2.1	5.4	2.1	2.1		29	Bucl5_Bm SAVP1.seq
	1	2	3	4	5	6	7	8	9	10	11	12	13	14	15	16	17	18	19	20	21	22	23	24	25	26	27	28	29		

bucl5

Percent Identity

	1	2	3	4	5	6	7	8	9	10	11	12	13	14	15	16	17	18	19	20	21	22	23	24	25	26	27	28	29	30		
**1**		80.9	95.1	64.0	44.2	63.7	95.1	56.6	76.0	98.9	98.9	80.9	98.9	52.1	50.2	56.6	56.6	53.2	50.2	60.3	46.8	43.1	49.8	53.6	50.2	50.2	63.3	50.2	50.2	60.3	**1**	Bucl6_Bps K96243.seq
**2**	5.1		85.0	77.9	61.8	80.1	85.0	71.5	92.1	82.0	82.0	100.0	82.0	68.9	66.7	73.0	73.0	69.7	66.7	77.2	63.3	59.2	66.3	70.0	66.7	66.7	80.1	66.7	66.7	77.2	**2**	Bucl6_Bps MSHR 305.seq
**3**	3.3	3.1		66.3	48.3	67.4	100.0	59.6	78.3	95.5	95.5	85.0	95.5	55.8	53.9	60.3	60.3	56.9	53.9	64.0	50.6	46.4	53.6	57.3	53.9	53.9	67.0	53.9	53.9	64.0	**3**	Bucl6_Bps 1106a.seq
**4**	5.0	8.9	7.2		73.0	95.9	66.3	87.6	74.5	63.7	63.7	77.9	63.7	83.1	82.4	88.4	88.4	85.0	82.4	91.0	78.7	74.2	81.3	85.8	82.4	82.4	94.8	82.4	82.4	91.0	**4**	Bucl6_Bps MSHR 346.seq
**5**	4.2	1.8	2.3	9.0		74.2	48.3	80.1	55.4	45.3	45.3	61.8	45.3	76.4	87.6	80.1	80.1	83.5	87.6	78.7	90.3	94.0	87.3	84.3	87.6	87.6	75.3	87.6	87.6	78.7	**5**	Bucl6_Bps 1026b.seq
**6**	5.9	4.9	4.7	5.4	7.4		67.4	86.5	75.3	64.8	64.8	80.1	64.8	86.5	84.3	91.0	91.0	87.6	84.3	93.6	81.3	77.5	84.6	87.6	84.3	84.3	97.4	84.3	84.3	93.6	**6**	Bucl6_Bps 1710b.seq
**7**	3.3	3.1	0.0	7.2	2.3	4.7		59.6	78.3	95.5	95.5	85.0	95.5	55.8	53.9	60.3	60.3	56.9	53.9	64.0	50.6	46.4	53.6	57.3	53.9	53.9	67.0	53.9	53.9	64.0	**7**	Bucl6_Bps BPC006.seq
**8**	6.8	8.2	7.2	7.4	7.8	9.0	7.2		67.4	56.9	56.9	71.5	56.9	88.0	87.3	94.0	94.0	89.9	87.3	91.4	83.5	79.0	86.1	90.6	87.3	87.3	87.6	87.3	87.3	91.4	**8**	A3N8K6_BURP6_Bucl6_Bp 668.seq
**9**	5.7	5.4	8.0	6.8	4.7	5.4	8.0	7.5		75.7	75.7	92.1	75.7	73.8	61.8	68.2	68.2	64.8	61.8	72.3	58.4	54.3	61.4	65.2	61.8	61.8	75.3	61.8	61.8	72.3	**9**	Bucl6_Bps NCTC 13179.seq
**10**	2.5	2.5	2.5	6.0	1.6	3.3	2.5	5.9	6.7		100.0	82.0	100.0	53.2	51.3	57.7	57.7	54.3	51.3	61.4	47.9	44.2	50.9	54.7	51.3	51.3	64.4	51.3	51.3	61.4	**10**	Bucl6_Bps MSHR146.seq
**11**	2.5	2.5	2.5	6.0	1.6	3.3	2.5	5.9	6.7	0.0		82.0	100.0	53.2	51.3	57.7	57.7	54.3	51.3	61.4	47.9	44.2	50.9	54.7	51.3	51.3	64.4	51.3	51.3	61.4	**11**	Bucl6_Bps MSHR511.seq
**12**	5.1	0.0	3.1	8.9	1.8	4.9	3.1	8.2	5.4	2.5	2.5		82.0	68.9	66.7	73.0	73.0	69.7	66.7	77.2	63.3	59.2	66.3	70.0	66.7	66.7	80.1	66.7	66.7	77.2	**12**	Bucl6_Bps MSHR520.seq
**13**	2.5	2.5	2.5	6.0	1.6	3.3	2.5	5.9	6.7	0.0	0.0	2.5		53.2	51.3	57.7	57.7	54.3	51.3	61.4	47.9	44.2	50.9	54.7	51.3	51.3	64.4	51.3	51.3	61.4	**13**	Bucl6_Bps NAU20B-16.seq
**14**	6.7	4.7	5.2	7.4	6.7	2.5	5.2	9.3	4.7	3.7	3.7	4.7	3.7		85.8	91.8	91.8	89.1	85.8	87.6	82.8	79.0	86.1	89.1	85.8	85.8	83.9	85.8	85.8	87.6	**14**	Bucl6_Bps NCTC 13178.seq
**15**	5.9	4.9	4.7	5.4	6.3	2.9	4.7	7.3	5.4	3.3	3.3	4.9	3.3	3.3		91.0	91.0	94.4	100.0	87.6	94.8	90.3	98.1	95.9	100.0	100.0	83.9	100.0	100.0	87.6	**15**	Bucl6_Bm 2000031281.seq
**16**	6.8	5.6	5.5	6.5	7.7	2.9	5.5	7.3	6.1	4.2	4.2	5.6	4.2	4.2	2.6		100.0	95.9	91.0	93.6	88.8	84.3	91.4	94.4	91.0	91.0	90.6	91.0	91.0	93.6	**16**	Bucl6_Bm 2002721280.seq
**17**	6.8	5.6	5.5	6.5	7.7	2.9	5.5	7.3	6.1	4.2	4.2	5.6	4.2	4.2	2.6	0.0		95.9	91.0	93.6	88.8	84.3	91.4	94.4	91.0	91.0	90.6	91.0	91.0	93.6	**17**	Bucl6_Bm A188.seq
**18**	6.8	5.6	5.5	6.5	7.4	2.9	5.5	8.3	6.1	4.2	4.2	5.6	4.2	3.3	2.5	0.9	0.9		94.4	90.3	92.1	87.6	94.8	98.5	94.4	94.4	87.3	94.4	94.4	90.3	**18**	Bucl6_Bm A193.seq
**19**	5.9	4.9	4.7	5.4	6.3	2.9	4.7	7.3	5.4	3.3	3.3	4.9	3.3	3.3	0.0	2.6	2.6	2.5		87.6	94.8	90.3	98.1	95.9	100.0	100.0	83.9	100.0	100.0	87.6	**19**	Bucl6_Bm China 7.seq
**20**	5.9	4.3	4.7	7.4	5.6	3.9	4.7	6.6	4.7	3.3	3.3	4.3	3.3	5.5	2.8	3.7	3.7	3.7	2.8		83.9	79.4	86.5	91.0	87.6	87.6	95.5	87.6	87.6	100.0	**20**	Bucl6_Bm PRL-20.seq
**21**	5.9	4.9	4.7	5.9	6.9	2.4	4.7	7.8	5.4	3.3	3.3	4.9	3.3	2.8	2.0	1.3	1.3	1.3	2.0	3.2		94.0	94.4	91.4	94.8	94.8	80.1	94.8	94.8	83.9	**21**	Bucl6_Bm strain_6.seq
**22**	6.8	6.2	6.3	7.5	6.2	2.9	6.3	9.3	6.8	4.2	4.2	6.2	4.2	3.3	3.3	2.6	2.6	2.5	3.3	4.7	2.8		92.1	86.9	90.3	90.3	76.4	90.3	90.3	79.4	**22**	Bucl6_Bm strain_11.seq
**23**	6.8	5.6	5.5	7.0	6.7	2.4	5.5	8.8	6.1	4.2	4.2	5.6	4.2	2.8	2.0	2.2	2.2	2.1	2.0	4.2	2.5	1.2		94.0	98.1	98.1	83.5	98.1	98.1	86.5	**23**	Bucl6_Bm NCTC 10229.seq
**24**	5.9	4.9	4.7	5.4	6.5	2.9	4.7	7.3	5.4	3.3	3.3	4.9	3.3	3.3	0.8	2.6	2.6	1.7	0.8	2.8	2.1	3.4	3.0		95.9	95.9	87.3	95.9	95.9	91.0	**24**	Bucl6_Bm NCTC 10247.seq
**25**	5.9	4.9	4.7	5.4	6.3	2.9	4.7	7.3	5.4	3.3	3.3	4.9	3.3	3.3	0.0	2.6	2.6	2.5	0.0	2.8	2.0	3.3	2.0	0.8		100.0	83.9	100.0	100.0	87.6	**25**	Bucl6_Bm FMH.seq
**26**	5.9	4.9	4.7	5.4	6.3	2.9	4.7	7.3	5.4	3.3	3.3	4.9	3.3	3.3	0.0	2.6	2.6	2.5	0.0	2.8	2.0	3.3	2.0	0.8	0.0		83.9	100.0	100.0	87.6	**26**	Bucl6_Bm JHU.seq
**27**	6.8	4.9	5.5	6.9	5.9	3.4	5.5	7.4	5.4	4.2	4.2	4.9	4.2	6.3	3.4	3.4	3.4	3.4	3.4	1.4	3.9	4.4	3.9	3.4	3.4	3.4		83.9	83.9	95.5	**27**	Bucl6_Bm ATCC 10399.seq
**28**	5.9	4.9	4.7	5.4	6.3	2.9	4.7	7.3	5.4	3.3	3.3	4.9	3.3	3.3	0.0	2.6	2.6	2.5	0.0	2.8	2.0	3.3	2.0	0.8	0.0	0.0	3.4		100.0	87.6	**28**	Bucl6_Bm GB8 horse 4.seq
**29**	5.9	4.9	4.7	5.4	6.3	2.9	4.7	7.3	5.4	3.3	3.3	4.9	3.3	3.3	0.0	2.6	2.6	2.5	0.0	2.8	2.0	3.3	2.0	0.8	0.0	0.0	3.4	0.0		87.6	**29**	Bucl6_Bm ATCC 23344.seq
**30**	5.9	4.3	4.7	7.4	5.6	3.9	4.7	6.6	4.7	3.3	3.3	4.3	3.3	5.5	2.8	3.7	3.7	3.7	2.8	0.0	3.2	4.7	4.2	2.8	2.8	2.8	1.4	2.8	2.8		**30**	Bucl6_Bm SAVP1.seq
	**1**	**2**	**3**	**4**	**5**	**6**	**7**	**8**	**9**	**10**	**11**	**12**	**13**	**14**	**15**	**16**	**17**	**18**	**19**	**20**	**21**	**22**	**23**	**24**	**25**	**26**	**27**	**28**	**29**	**30**		

bucl6

Percent Identity

	1	2	3	4	5	6	7	8	9	10	11	12	13	14	15	16	17	18	19	20	21	22	23	24	25	26	27		
1		93.0	84.5	93.4	95.3	84.5	96.2	93.4	93.0	93.0	93.0	93.0	92.0	95.3	95.3	95.3	95.3	95.3	95.3	95.3	95.3	95.3	95.3	95.3	93.9	95.3	95.3	1	Bud7_Bps K96243.seq
2	4.4		84.5	95.8	94.4	84.5	93.4	96.7	97.7	97.7	99.5	97.7	94.8	95.3	95.3	95.3	95.3	95.3	95.3	95.3	95.3	95.3	95.3	95.3	97.7	95.3	95.3	2	Bud7_Bps MSHR 305.seq
3	4.4	7.6		86.4	85.4	99.5	83.6	87.3	85.4	85.4	84.5	85.4	87.8	85.4	85.4	85.4	85.4	85.4	85.4	85.4	85.4	85.4	85.4	85.4	86.4	85.4	85.4	3	A3P4A7_BURP0_Bucl7_Bp 1106a.seq
4	3.8	4.2	5.3		94.4	86.4	93.4	97.7	95.8	95.8	95.8	95.8	93.9	93.4	93.4	93.4	93.4	93.4	93.4	93.4	93.4	93.4	93.4	93.4	95.8	93.4	93.4	4	Bud7_Bps 1026b.seq
5	3.3	4.3	4.9	4.3		85.4	94.8	95.3	95.3	95.3	94.4	95.3	93.4	97.7	97.7	97.7	97.7	97.7	97.7	97.7	97.7	97.7	97.7	97.7	95.3	97.7	97.7	5	Bud7_Bps 1710b.seq
6	4.4	7.6	0.0	5.3	4.9		83.6	87.3	85.4	85.4	84.5	85.4	87.8	85.4	85.4	85.4	85.4	85.4	85.4	85.4	85.4	85.4	85.4	85.4	86.4	85.4	85.4	6	Bud7_Bps BPC006.seq
7	3.8	3.8	5.5	3.8	3.8	5.5		94.4	94.4	94.4	93.4	94.4	93.0	94.8	94.8	94.8	94.8	94.8	94.8	94.8	94.8	94.8	94.8	94.8	93.4	94.8	94.8	7	Bud7_Bps 668.seq
8	3.8	3.2	4.2	2.1	3.2	4.2	2.7		97.7	97.7	96.7	97.7	95.8	94.4	94.4	94.4	94.4	94.4	94.4	94.4	94.4	94.4	94.4	94.4	96.7	94.4	94.4	8	Bud7_Bps NCTC 13179.seq
9	4.4	2.1	6.5	4.2	3.2	6.5	2.7	2.1		99.5	97.7	99.5	95.8	95.3	95.3	95.3	95.3	95.3	95.3	95.3	95.3	95.3	95.3	95.3	97.7	95.3	95.3	9	Bud7_Bps MSHR146.seq
10	4.4	2.1	6.5	4.2	3.2	6.5	2.7	2.1	0.0		97.7	99.5	95.8	95.3	95.3	95.3	95.3	95.3	95.3	95.3	95.3	95.3	95.3	95.3	97.7	95.3	95.3	10	Bud7_Bps MSHR511.seq
11	4.4	0.0	7.6	4.2	4.3	7.6	3.8	3.2	2.1	2.1		97.7	94.8	95.3	95.3	95.3	95.3	95.3	95.3	95.3	95.3	95.3	95.3	95.3	97.7	95.3	95.3	11	Bud7_Bps MSHR520.seq
12	4.4	2.1	6.5	4.2	3.2	6.5	2.7	2.1	0.0	0.0	2.1		95.8	95.3	95.3	95.3	95.3	95.3	95.3	95.3	95.3	95.3	95.3	95.3	97.7	95.3	95.3	12	Bud7_Bps NAU20B-16.seq
13	3.8	3.7	5.3	4.8	3.8	5.3	2.7	2.6	2.6	2.6	3.7	2.6		94.4	94.4	94.4	94.4	94.4	94.4	94.4	94.4	94.4	94.4	94.4	95.8	94.4	94.4	13	Bud7_Bps NCTC 13178.seq
14	3.3	3.2	4.9	5.4	2.1	4.9	3.8	4.3	3.2	3.2	3.2	3.2	2.7		99.5	99.5	99.5	99.5	99.5	99.5	99.5	99.5	99.5	99.5	97.2	99.5	99.5	14	Bud7_Bm 2000031281.seq
15	3.3	3.2	4.9	5.4	2.1	4.9	3.8	4.3	3.2	3.2	3.2	3.2	2.7	0.0		99.5	99.5	99.5	99.5	99.5	99.5	99.5	99.5	99.5	97.2	99.5	99.5	15	Bud7_Bm 2002721280.seq
16	3.3	3.2	4.9	5.4	2.1	4.9	3.8	4.3	3.2	3.2	3.2	3.2	2.7	0.0	0.0		99.5	99.5	99.5	99.5	99.5	99.5	99.5	99.5	97.2	99.5	99.5	16	Bud7_Bm A188.seq
17	3.3	3.2	4.9	5.4	2.1	4.9	3.8	4.3	3.2	3.2	3.2	3.2	2.7	0.0	0.0	0.0		99.5	99.5	99.5	99.5	99.5	99.5	99.5	97.2	99.5	99.5	17	Bud7_Bm China 7.seq
18	3.3	3.2	4.9	5.4	2.1	4.9	3.8	4.3	3.2	3.2	3.2	3.2	2.7	0.0	0.0	0.0	0.0		99.5	99.5	99.5	99.5	99.5	99.5	97.2	99.5	99.5	18	Bud7_Bm PRL-20.seq
19	3.3	3.2	4.9	5.4	2.1	4.9	3.8	4.3	3.2	3.2	3.2	3.2	2.7	0.0	0.0	0.0	0.0	0.0		99.5	99.5	99.5	99.5	99.5	97.2	99.5	99.5	19	Bud7_Bm strain_6.seq
20	3.3	3.2	4.9	5.4	2.1	4.9	3.8	4.3	3.2	3.2	3.2	3.2	2.7	0.0	0.0	0.0	0.0	0.0	0.0		99.5	99.5	99.5	99.5	97.2	99.5	99.5	20	Bud7_Bm strain_11.seq
21	3.3	3.2	4.9	5.4	2.1	4.9	3.8	4.3	3.2	3.2	3.2	3.2	2.7	0.0	0.0	0.0	0.0	0.0	0.0	0.0		99.5	99.5	99.5	97.2	99.5	99.5	21	Bud7_Bm NCTC 10229.seq
22	3.3	3.2	4.9	5.4	2.1	4.9	3.8	4.3	3.2	3.2	3.2	3.2	2.7	0.0	0.0	0.0	0.0	0.0	0.0	0.0	0.0		99.5	99.5	97.2	99.5	99.5	22	Bud7_Bm NCTC 10247.seq
23	3.3	3.2	4.9	5.4	2.1	4.9	3.8	4.3	3.2	3.2	3.2	3.2	2.7	0.0	0.0	0.0	0.0	0.0	0.0	0.0	0.0	0.0		99.5	97.2	99.5	99.5	23	Bud7_Bm FMH.seq
24	3.3	3.2	4.9	5.4	2.1	4.9	3.8	4.3	3.2	3.2	3.2	3.2	2.7	0.0	0.0	0.0	0.0	0.0	0.0	0.0	0.0	0.0	0.0		97.2	99.5	99.5	24	Bud7_Bm JHU.seq
25	3.3	2.1	5.3	4.2	3.2	5.3	3.8	3.2	2.1	2.1	2.1	2.1	2.6	1.1	1.1	1.1	1.1	1.1	1.1	1.1	1.1	1.1	1.1	1.1		97.2	97.2	25	Bud7_Bm ATCC 10399.seq
26	3.3	3.2	4.9	5.4	2.1	4.9	3.8	4.3	3.2	3.2	3.2	3.2	2.7	0.0	0.0	0.0	0.0	0.0	0.0	0.0	0.0	0.0	0.0	0.0	1.1		99.5	26	Bud7_Bm GB8 horse 4.seq
27	3.3	3.2	4.9	5.4	2.1	4.9	3.8	4.3	3.2	3.2	3.2	3.2	2.7	0.0	0.0	0.0	0.0	0.0	0.0	0.0	0.0	0.0	0.0	0.0	1.1	0.0		27	Bud7_Bm ATCC 23344.seq
	1	2	3	4	5	6	7	8	9	10	11	12	13	14	15	16	17	18	19	20	21	22	23	24	25	26	27		

bucl7

Percent Identity

	1	2	3	4	5	6	7	8	9	10	11	12	13	14	15	16	17	18	19	20	21	22	23	24	25	26	27	28	29	30	31	32	33	34	35	36	37	38	39	40		
1		91.4	90.7	90.6	90.9	95.2	90.8	91.2	90.3	92.3	91.6	91.4	91.6	92.3	97.1	93.9	91.2	90.3	91.5	90.3	91.2	89.6	90.2	90.3	90.3	90.3	91.2	91.2	90.0	91.2	91.2	89.6	78.3	78.3	78.3	80.3	79.2	81.5	80.9	82.0	1	Bucl8_Bps K96243.seq
2	1.1		97.0	98.8	97.3	95.8	97.0	98.1	96.8	97.7	97.9	100.0	97.9	98.7	94.0	97.1	97.4	96.7	97.8	96.7	97.4	95.9	96.6	96.7	96.7	96.7	97.4	97.4	96.4	97.4	97.4	95.9	84.9	84.9	84.9	87.3	85.0	88.7	87.8	89.1	2	A4LQJ4_BURPS_Bucl8_Bps 305.seq
3	0.5	0.8		97.8	99.0	95.0	99.9	97.7	99.2	97.1	98.0	97.0	98.0	96.2	93.3	96.6	99.3	99.3	98.8	99.3	99.3	98.5	99.3	99.3	99.3	99.3	99.3	99.3	98.8	99.3	99.3	98.5	87.2	87.2	87.2	89.7	87.3	91.3	90.4	91.7	3	Bucl8_Bps 1106a.seq
4	0.9	0.4	0.8		98.2	95.1	97.8	98.3	97.6	97.0	97.9	98.8	97.9	97.8	93.2	96.4	98.3	97.5	98.7	97.5	98.3	96.8	97.4	97.5	97.5	97.5	98.3	98.3	97.2	98.3	98.3	96.8	85.7	85.7	85.7	88.0	85.7	89.6	88.6	90.0	4	Bucl8_Bps MSHR 346.seq
5	0.9	1.0	0.5	1.0		95.3	99.0	98.1	98.8	97.3	98.2	97.3	98.2	96.5	93.5	96.7	99.2	98.7	98.9	98.7	99.2	97.9	98.6	98.7	98.7	98.7	99.2	99.2	98.4	99.2	99.2	97.9	86.6	86.6	86.6	89.0	86.6	90.6	89.7	91.0	5	Bucl8_Bps 1026b.seq
6	0.4	1.0	0.6	0.9	0.9		95.2	95.8	94.7	96.9	96.2	95.8	96.2	96.8	97.9	98.1	95.5	94.6	95.9	94.6	95.5	93.9	94.5	94.6	94.6	94.6	95.5	95.5	94.3	95.5	95.5	93.9	82.7	82.7	82.7	84.8	82.7	86.3	85.5	86.7	6	Bucl8_Bps 1710b.seq
7	0.4	0.7	0.2	0.8	0.4	0.4		97.7	99.2	97.1	98.0	97.0	98.0	96.3	93.4	96.6	99.3	99.4	98.8	99.4	99.3	98.4	99.3	99.4	99.4	99.4	99.3	99.3	98.9	99.3	99.3	98.4	87.2	87.2	87.2	89.7	87.3	91.3	90.4	91.7	7	Bucl8_Bps BPC006.seq
8	1.5	0.9	1.1	0.8	1.3	1.2	1.0		97.5	97.8	98.7	98.1	98.7	97.2	93.8	97.0	98.1	97.5	98.5	97.5	98.1	96.8	97.4	97.5	97.5	97.5	98.1	98.1	97.2	98.1	98.1	96.8	85.6	85.6	85.6	88.0	85.7	89.6	88.6	90.0	8	Bucl8_Bps 668.seq
9	0.5	0.5	0.4	0.5	0.2	0.5	0.4	0.8		96.8	97.7	96.8	97.7	95.9	92.9	96.2	98.9	99.8	98.4	99.8	98.9	98.9	99.7	99.8	99.8	99.8	98.9	98.9	99.3	98.9	98.9	98.9	87.5	87.5	87.5	90.0	87.6	91.7	90.7	92.1	9	Bucl8_Bps NCTC 13179.seq
10	1.2	1.3	0.8	1.2	1.2	1.0	0.7	1.5	0.6		99.1	97.7	99.1	98.0	94.8	98.2	97.6	96.9	97.9	96.9	97.6	95.9	96.8	96.9	96.9	96.9	97.6	97.6	96.3	97.6	97.6	95.9	84.7	84.7	84.7	87.0	84.8	88.7	87.7	89.1	10	Bucl8_Bps MSHR146.seq
11	1.0	1.1	0.8	1.2	1.2	0.8	0.7	1.5	0.6	0.0		97.9	100.0	97.2	94.1	97.5	98.5	97.7	98.8	97.7	98.5	96.8	97.6	97.7	97.7	97.7	98.5	98.5	97.2	98.5	98.5	96.8	85.6	85.6	85.6	87.9	85.7	89.6	88.6	90.1	11	Bucl8_Bps MSHR511.seq
12	1.1	0.0	0.8	0.4	1.0	1.0	0.7	0.9	0.5	1.3	1.1		97.9	98.7	94.0	97.1	97.4	96.7	97.8	96.7	97.4	95.9	96.6	96.7	96.7	96.7	97.4	97.4	96.4	97.4	97.4	95.9	84.9	84.9	84.9	87.3	85.0	88.7	87.8	89.1	12	Bucl8_Bps MSHR520.seq
13	1.0	1.1	0.8	1.2	1.2	0.8	0.7	1.5	0.6	0.0	0.0	1.1		97.2	94.1	97.5	98.5	97.7	98.8	97.7	98.5	96.8	97.6	97.7	97.7	97.7	98.5	98.5	97.2	98.5	98.5	96.8	85.6	85.6	85.6	87.9	85.7	89.6	88.6	90.1	13	Bucl8_Bps NAU20B-16.seq
14	1.0	0.5	0.6	0.4	0.9	1.0	0.5	0.9	0.5	1.1	0.9	0.5	0.9		94.9	98.1	96.8	95.9	97.2	95.9	96.8	95.1	95.8	95.9	95.9	95.9	96.8	96.8	95.6	96.8	96.8	95.1	83.9	83.9	83.9	86.2	84.0	87.8	86.8	88.1	14	Bucl8_Bps NCTC 13178.seq
15	0.3	1.1	0.6	1.0	1.0	0.4	0.4	1.5	0.5	1.3	1.1	1.1	1.1	1.1		96.5	93.7	92.9	94.2	92.9	93.7	92.2	92.8	92.9	92.9	92.9	93.7	93.7	92.6	93.7	93.7	92.2	81.0	81.0	81.0	83.1	81.0	84.4	83.7	84.9	15	B7CU27_BURPS_Bucl8_Bps 576.seq
16	0.4	1.0	0.4	0.9	0.9	0.6	0.3	1.4	0.3	1.0	0.8	1.0	0.8	1.0	0.5		97.1	96.2	97.5	96.2	97.1	95.4	96.1	96.2	96.2	96.2	97.1	97.1	95.9	97.1	97.1	95.4	84.1	84.1	84.1	86.4	84.1	87.8	87.1	88.3	16	C0XVP3_BURPS_Bucl8_Bps Pakistan 9.seq
17	0.5	0.7	0.3	0.7	0.7	0.6	0.3	1.1	0.3	0.6	0.6	0.7	0.6	0.5	0.6	0.3		99.0	99.5	99.0	100.0	98.2	98.9	99.0	99.0	99.0	100.0	100.0	98.6	100.0	100.0	98.2	86.7	86.7	86.7	89.1	86.7	90.8	89.8	91.2	17	Bucl8_Bm 2000031281.seq
18	0.4	0.5	0.3	0.6	0.3	0.5	0.2	0.8	0.3	0.5	0.5	0.5	0.5	0.4	0.5	0.3	0.1		98.5	100.0	99.0	99.0	99.9	100.0	100.0	100.0	99.0	99.0	99.5	99.0	99.0	99.0	87.6	87.6	87.6	90.1	87.6	91.7	90.7	92.2	18	Bucl8_Bm 2002721280.seq
19	0.5	0.8	0.4	0.8	0.9	0.6	0.3	1.2	0.3	0.8	0.8	0.8	0.8	0.5	0.6	0.3	0.1	0.2		98.5	99.5	97.7	98.4	98.5	98.5	98.5	99.5	99.5	98.2	99.5	99.5	97.7	86.2	86.2	86.2	88.7	86.3	90.3	89.3	90.7	19	Bucl8_Bm A188.seq
20	0.4	0.5	0.3	0.6	0.3	0.5	0.2	0.8	0.3	0.5	0.5	0.5	0.5	0.4	0.5	0.3	0.1	0.0	0.2		99.0	99.0	99.9	100.0	100.0	100.0	99.0	99.0	99.5	99.0	99.0	99.0	87.6	87.6	87.6	90.1	87.6	91.7	90.7	92.2	20	Bucl8_Bm A193.seq
21	0.5	0.7	0.3	0.7	0.7	0.6	0.3	1.1	0.3	0.6	0.6	0.7	0.6	0.5	0.6	0.3	0.0	0.1	0.1	0.1		98.2	98.9	99.0	99.0	99.0	100.0	100.0	98.6	100.0	100.0	98.2	86.7	86.7	86.7	89.1	86.7	90.8	89.8	91.2	21	Bucl8_Bm China 7.seq
22	0.3	0.5	0.2	0.5	0.2	0.4	0.3	0.7	0.3	0.7	0.7	0.5	0.7	0.4	0.3	0.2	0.1	0.1	0.1	0.1	0.1		99.0	99.0	99.0	99.0	98.2	98.2	99.5	98.2	98.2	100.0	88.5	88.5	88.5	91.0	88.5	92.7	91.7	93.1	22	Bucl8_Bm PRL-20.seq
23	0.5	0.7	0.3	0.7	0.4	0.7	0.3	0.9	0.4	0.7	0.7	0.7	0.7	0.5	0.6	0.4	0.2	0.1	0.3	0.1	0.2	0.1		99.9	99.9	99.9	98.9	98.9	99.4	98.9	98.9	99.0	87.5	87.5	87.5	90.0	87.6	91.7	90.7	92.1	23	Bucl8_Bm strain_6.seq
24	0.4	0.5	0.3	0.6	0.3	0.5	0.2	0.8	0.3	0.5	0.5	0.5	0.5	0.4	0.5	0.3	0.1	0.0	0.2	0.0	0.1	0.1	0.1		100.0	100.0	99.0	99.0	99.5	99.0	99.0	99.0	87.6	87.6	87.6	90.1	87.6	91.7	90.7	92.2	24	Bucl8_Bm strain_11.seq
25	0.4	0.5	0.3	0.6	0.3	0.5	0.2	0.8	0.3	0.5	0.5	0.5	0.5	0.4	0.5	0.3	0.1	0.0	0.2	0.0	0.1	0.1	0.1	0.0		100.0	99.0	99.0	99.5	99.0	99.0	99.0	87.6	87.6	87.6	90.1	87.6	91.7	90.7	92.2	25	Bucl8_Bm NCTC 10229.seq
26	0.4	0.5	0.3	0.6	0.3	0.5	0.2	0.8	0.3	0.5	0.5	0.5	0.5	0.4	0.5	0.3	0.1	0.0	0.2	0.0	0.1	0.1	0.1	0.0	0.0		99.0	99.0	99.5	99.0	99.0	99.0	87.6	87.6	87.6	90.1	87.6	91.7	90.7	92.2	26	Bucl8_Bm NCTC 10247.seq
27	0.5	0.7	0.3	0.7	0.7	0.6	0.3	1.1	0.3	0.6	0.6	0.7	0.6	0.5	0.6	0.3	0.0	0.1	0.1	0.1	0.0	0.1	0.2	0.1	0.1	0.1		100.0	98.6	100.0	100.0	98.2	86.7	86.7	86.7	89.1	86.7	90.8	89.8	91.2	27	Bucl8_Bm FMH.seq
28	0.5	0.7	0.3	0.7	0.7	0.6	0.3	1.1	0.3	0.6	0.6	0.7	0.6	0.5	0.6	0.3	0.0	0.1	0.1	0.1	0.0	0.1	0.2	0.1	0.1	0.1	0.0		98.6	100.0	100.0	98.2	86.7	86.7	86.7	89.1	86.7	90.8	89.8	91.2	28	Bucl8_Bm JHU.seq
29	0.3	0.4	0.3	0.4	0.2	0.4	0.3	0.6	0.3	0.7	0.7	0.4	0.7	0.3	0.3	0.2	0.1	0.1	0.0	0.1	0.1	0.1	0.2	0.1	0.1	0.1	0.1	0.1		98.6	98.6	99.5	88.0	88.0	88.0	90.5	88.1	92.2	91.2	92.7	29	Bucl8_Bm ATCC 10399.seq
30	0.5	0.7	0.3	0.7	0.7	0.6	0.3	1.1	0.3	0.6	0.6	0.7	0.6	0.5	0.6	0.3	0.0	0.1	0.1	0.1	0.0	0.1	0.2	0.1	0.1	0.1	0.0	0.0	0.1		100.0	98.2	86.7	86.7	86.7	89.1	86.7	90.8	89.8	91.2	30	Bucl8_Bm GB8 horse 4.seq
31	0.5	0.7	0.3	0.7	0.7	0.6	0.3	1.1	0.3	0.6	0.6	0.7	0.6	0.5	0.6	0.3	0.0	0.1	0.1	0.1	0.0	0.1	0.2	0.1	0.1	0.1	0.0	0.0	0.1	0.0		98.2	86.7	86.7	86.7	89.1	86.7	90.8	89.8	91.2	31	Bucl8_Bm ATCC 23344.seq
32	0.3	0.5	0.2	0.5	0.2	0.4	0.3	0.7	0.3	0.7	0.7	0.5	0.7	0.4	0.3	0.2	0.1	0.1	0.1	0.1	0.1	0.0	0.1	0.1	0.1	0.1	0.1	0.1	0.1	0.1	0.1		88.5	88.5	88.5	91.0	88.5	92.7	91.7	93.1	32	Bucl8_Bm SAVP1.seq
33	11.7	11.7	11.7	11.9	11.7	11.9	11.7	12.1	11.9	12.1	12.1	11.7	12.1	11.8	11.7	11.7	11.8	11.8	11.8	11.8	11.8	11.8	11.9	11.8	11.8	11.8	11.8	11.8	11.8	11.8	11.8	11.8		100.0	100.0	99.3	94.3	94.4	96.7	94.8	33	Bucl8_Bt E264.seq
34	11.7	11.7	11.7	11.9	11.7	11.9	11.7	12.1	11.9	12.1	12.1	11.7	12.1	11.8	11.7	11.7	11.8	11.8	11.8	11.8	11.8	11.8	11.9	11.8	11.8	11.8	11.8	11.8	11.8	11.8	11.8	11.8	0.0		100.0	99.3	94.3	94.4	96.7	94.8	34	Bucl8_Bt 2002721723.seq
35	11.7	11.7	11.7	11.9	11.7	11.9	11.7	12.1	11.9	12.1	12.1	11.7	12.1	11.8	11.7	11.7	11.8	11.8	11.8	11.8	11.8	11.8	11.9	11.8	11.8	11.8	11.8	11.8	11.8	11.8	11.8	11.8	0.0	0.0		99.3	94.3	94.4	96.7	94.8	35	Bucl8_Bt E444.seq
36	8.3	8.2	8.2	8.4	8.3	8.4	8.2	8.6	8.5	8.7	8.7	8.2	8.7	8.4	8.2	8.2	8.4	8.4	8.4	8.4	8.4	8.4	8.4	8.4	8.4	8.4	8.4	8.4	8.4	8.4	8.4	8.4	0.8	0.8	0.8		96.0	94.3	96.2	94.7	36	Bucl8_Bt E555.seq
37	11.3	11.1	11.2	11.3	11.2	11.4	11.2	11.5	11.4	11.5	11.5	11.1	11.5	11.3	11.2	11.2	11.3	11.3	11.3	11.3	11.3	11.3	11.4	11.3	11.3	11.3	11.3	11.3	11.3	11.3	11.3	11.3	6.1	6.1	6.1	3.9		94.6	96.5	95.0	37	Bucl8_Bt H0587.seq
38	6.5	6.4	6.4	6.4	6.5	6.5	6.4	6.7	6.5	6.6	6.6	6.4	6.6	6.4	6.5	6.5	6.4	6.4	6.4	6.4	6.4	6.4	6.5	6.4	6.4	6.4	6.4	6.4	6.4	6.4	6.4	6.4	6.6	6.6	6.6	6.7	5.8		95.1	99.2	38	Bucl8_Bt MSMB43.seq
39	7.6	7.6	7.6	7.8	7.6	7.8	7.6	8.0	7.7	8.0	8.0	7.6	8.0	7.8	7.6	7.6	7.7	7.7	7.7	7.7	7.7	7.7	7.8	7.7	7.7	7.7	7.7	7.7	7.7	7.7	7.7	7.7	3.7	3.7	3.7	4.4	3.6	5.9		95.5	39	Bucl8_Bt 4.seq
40	5.9	5.9	5.9	6.0	5.9	6.0	5.9	6.1	5.9	6.1	6.1	5.9	6.1	5.9	5.9	5.9	5.9	5.9	5.9	5.9	5.9	5.9	5.9	5.9	5.9	5.9	5.9	5.9	5.9	5.9	5.9	5.9	6.1	6.1	6.1	6.1	5.4	0.9	5.3		40	Bucl8_Bt MSMB 121.seq
	1	2	3	4	5	6	7	8	9	10	11	12	13	14	15	16	17	18	19	20	21	22	23	24	25	26	27	28	29	30	31	32	33	34	35	36	37	38	39	40		

bucl8

Percent Identity

	1	2	3	4	5	6	7	8	9	10	11	12	13	14	15	16	17	18	19	20	21	22	23	24	25	26	27	28		
**1**		98.0	88.7	93.8	79.8	94.0	81.5	94.0	81.5	95.6	83.3	79.8	81.5	87.5	96.0	92.3	83.3	86.9	92.3	86.9	78.0	85.1	92.3	88.7	97.6	76.2	79.8	70.8	**1**	B2GYG4_BURPS_Bucl10_Bps 1655.seq
**2**	0.3		86.7	95.4	77.8	92.1	79.6	95.6	79.6	97.6	81.3	77.8	83.1	89.1	97.6	90.3	84.9	88.5	90.3	84.9	76.0	83.1	90.3	90.3	95.6	77.8	81.3	68.8	**2**	COY5B0_BURPS_Bucl10_Bps Pakistan 9.seq
**3**	0.9	1.3		86.7	86.9	94.0	91.9	90.1	91.9	88.5	93.7	90.1	78.6	83.1	88.7	95.8	79.4	82.9	88.3	94.0	85.1	84.7	95.8	84.7	90.5	72.2	75.8	81.2	**3**	Q3JSM5_BURP1_Bucl10_Bps 1710b.seq
**4**	1.3	1.7	1.3		78.4	92.7	80.2	96.2	80.2	97.8	81.9	78.4	86.7	93.3	97.4	90.9	89.1	92.7	90.9	85.5	76.6	83.7	90.9	94.4	96.2	81.9	85.5	69.4	**4**	Bucl10_Bps NCTC 13179.seq
**5**	0.9	1.3	0.8	0.3		85.7	87.5	78.6	94.6	80.2	89.3	89.3	65.5	72.0	79.8	87.5	67.9	71.4	76.8	92.9	98.2	73.2	87.5	73.2	82.1	60.7	64.3	91.1	**5**	Bucl10_Bps K96243.seq
**6**	0.9	1.3	0.9	0.3	0.0		87.5	92.9	87.5	94.4	89.3	85.7	79.8	86.3	94.0	98.2	82.1	85.7	91.1	92.9	83.9	87.5	98.2	87.5	96.4	75.0	78.6	76.8	**6**	Bucl10_Bps 1026b.seq
**7**	0.9	1.3	1.3	0.3	0.0	0.0		83.9	92.9	81.9	98.2	98.2	77.4	77.0	81.5	89.3	73.2	76.8	89.3	94.6	85.7	85.5	89.3	78.6	83.9	73.0	76.8	85.7	**7**	A1V459_BURMS_Bucl10_Bm SAVP1.seq
**8**	1.0	1.3	1.5	0.3	0.0	0.0	0.0		83.9	98.0	85.7	82.1	86.5	93.1	97.6	91.1	89.3	92.9	94.6	85.7	76.8	87.5	91.1	94.6	96.4	82.1	85.7	73.2	**8**	A2S325_BURM9_Bucl10_Bm NCTC 10229.seq
**9**	0.9	1.3	1.3	0.3	0.0	0.0	0.0	0.0		81.9	94.6	94.6	70.4	77.0	81.5	89.3	73.2	76.8	82.1	94.6	92.9	78.6	89.3	78.6	83.9	66.1	69.6	89.3	**9**	A3MJE6_BURM7_Bucl10_Bm NCTC 10247.seq
**10**	1.3	1.0	1.2	0.6	0.3	0.3	0.3	0.3	0.3		83.7	80.2	84.9	91.5	99.2	92.7	87.3	90.9	92.7	87.3	78.4	85.5	92.7	92.7	98.0	80.2	83.7	71.2	**10**	A3NUX3_BURP0_Bucl10_Bps 1106a.seq
**11**	0.9	1.3	1.3	0.3	0.0	0.0	0.0	0.0	0.0	0.3		96.4	75.8	78.8	83.3	91.1	75.0	78.6	87.5	96.4	87.5	83.9	91.1	80.4	85.7	71.4	75.0	87.5	**11**	A5TLC5_BURMA_Bucl10_Bm 2002721280.seq
**12**	0.9	1.3	1.3	0.3	0.0	0.0	0.0	0.0	0.0	0.3	0.0		75.6	75.2	79.8	87.5	71.4	75.0	87.5	92.9	87.5	83.7	87.5	76.8	82.1	71.2	75.0	87.5	**12**	A9K814_BURMA_Bucl10_Bm ATCC 10399.seq
**13**	1.0	1.4	0.0	1.3	1.0	1.0	1.8	1.6	1.6	1.3	1.6	1.8		86.7	85.1	78.0	82.9	86.5	88.1	72.6	63.7	91.9	78.0	88.3	83.3	86.5	86.3	63.3	**13**	B7CPT6_BURPS_Bucl10a_Bps 576.seq
**14**	3.2	3.6	4.4	2.4	2.1	2.1	2.7	2.7	2.7	2.4	2.7	2.7	4.4		91.1	84.5	93.7	96.8	87.7	79.2	70.2	80.6	84.5	98.4	89.9	86.7	90.1	66.3	**14**	Bucl10_Bps 668.seq
**15**	1.0	1.3	0.6	1.0	0.6	0.6	0.6	0.6	0.6	0.9	0.6	0.6	0.7	2.8		92.3	86.9	90.5	92.3	86.9	78.0	85.1	92.3	92.3	97.6	79.8	83.3	70.8	**15**	Bucl10_Bps BPC006_2.seq
**16**	0.9	1.3	0.8	0.3	0.0	0.0	0.0	0.0	0.0	0.3	0.0	0.0	1.0	2.1	0.6		80.4	83.9	89.3	94.6	85.7	85.7	100.0	85.7	94.6	73.2	76.8	78.6	**16**	A8EDY0_BURPS_Bucl10_Bps 406e.seq
**17**	1.2	1.6	1.8	0.4	0.0	0.0	0.0	0.0	0.0	0.4	0.0	0.0	1.8	1.8	0.7	0.0		96.4	83.9	75.0	66.1	76.8	80.4	94.6	85.7	92.9	96.4	62.5	**17**	Bucl10_Bm A188.seq
**18**	1.1	1.5	1.7	0.3	0.0	0.0	0.0	0.0	0.0	0.3	0.0	0.0	1.7	2.4	0.7	0.0	0.0		87.5	78.6	69.6	80.4	83.9	98.2	89.3	89.3	92.9	66.1	**18**	Bucl10_Bm A193.seq
**19**	0.9	1.3	1.5	0.3	0.0	0.0	0.0	0.0	0.0	0.3	0.0	0.0	1.8	2.7	0.6	0.0	0.0	0.0		83.9	75.0	92.7	89.3	89.3	94.6	83.7	87.5	75.0	**19**	Bucl10_Bm PRL-20.seq
**20**	0.9	1.3	0.8	0.3	0.0	0.0	0.0	0.0	0.0	0.3	0.0	0.0	1.0	2.1	0.6	0.0	0.0	0.0	0.0		91.1	80.4	94.6	80.4	89.3	67.9	71.4	83.9	**20**	Bucl10_Bps MSHR146.seq
**21**	0.9	1.3	0.8	0.3	0.0	0.0	0.0	0.0	0.0	0.3	0.0	0.0	1.0	2.1	0.6	0.0	0.0	0.0	0.0	0.0		71.4	85.7	71.4	80.4	58.9	62.5	92.9	**21**	Bucl10_Bps MSHR511.seq
**22**	0.9	1.3	1.4	0.3	0.0	0.0	0.3	0.0	0.0	0.3	0.0	0.3	1.4	2.7	0.6	0.0	0.0	0.0	0.3	0.0	0.0		85.7	82.1	87.5	80.4	80.2	71.4	**22**	Bucl10_Bps MSHR520.seq
**23**	0.9	1.3	0.8	0.3	0.0	0.0	0.0	0.0	0.0	0.3	0.0	0.0	1.0	2.1	0.6	0.0	0.0	0.0	0.0	0.0	0.0	0.0		85.7	94.6	73.2	76.8	78.6	**23**	Bucl10_Bps NAU20B-16.seq
**24**	1.1	1.4	1.7	0.3	0.0	0.0	0.0	0.0	0.0	0.3	0.0	0.0	1.7	2.7	0.7	0.0	0.0	0.0	0.0	0.0	0.0	0.0	0.0		91.1	87.5	91.1	67.9	**24**	Bucl10_Bps NCTC 13178.seq
**25**	0.9	1.3	0.9	0.3	0.0	0.0	0.0	0.0	0.0	0.3	0.0	0.0	1.0	2.1	0.6	0.0	0.0	0.0	0.0	0.0	0.0	0.0	0.0	0.0		78.6	82.1	73.2	**25**	C4KXC1_BURPS_Bucl10_MSHR 346.seq
**26**	1.2	1.6	1.9	0.4	0.0	0.0	0.3	0.0	0.0	0.4	0.0	0.3	1.7	1.5	0.8	0.0	0.0	0.0	0.3	0.0	0.0	0.0	0.0	0.0	0.0		96.2	58.9	**26**	A4LQ22_BURPS_Bucl10_Bps 305.seq
**27**	1.2	1.6	1.8	0.4	0.0	0.0	0.0	0.0	0.0	0.4	0.0	0.0	2.1	1.8	0.7	0.0	0.0	0.0	0.0	0.0	0.0	0.3	0.0	0.0	0.0	0.3		62.5	**27**	Bucl10_Bm strain_6.seq
**28**	0.9	1.3	1.3	0.3	0.0	0.0	0.0	0.0	0.0	0.3	0.0	0.0	1.6	2.7	0.6	0.0	0.0	0.0	0.0	0.0	0.0	0.0	0.0	0.0	0.0	0.0	0.0		**28**	Bucl10_Bm strain_11.seq
	1	2	3	4	5	6	7	8	9	10	11	12	13	14	15	16	17	18	19	20	21	22	23	24	25	26	27	28		

bucl10

Percent Identity

	1	2	3	4	5	6	7	8	9	10	11	12	13	14	15	16	17	18	19	20	21	22	23	24	25	26	27	28	29	30	31		
**1**		92.4	90.3	89.6	90.1	90.1	89.6	90.1	86.6	87.3	91.2	87.3	88.7	96.5	93.5	90.1	94.9	90.8	92.9	90.1	92.2	92.2	94.0	93.5	93.1	90.1	90.1	91.5	90.1	90.1	92.2	**1**	Bucl13_Bps K96243.seq
**2**	2.9		94.5	95.6	94.9	94.9	92.9	87.3	90.8	91.9	96.8	91.9	94.7	93.1	93.3	95.9	94.2	95.9	94.9	95.9	96.8	95.4	94.7	95.6	96.3	95.9	95.9	95.6	95.9	95.9	96.8	**2**	Bucl13_Bps MSHR 305.seq
**3**	6.1	5.3		92.6	92.9	93.3	91.9	86.4	89.9	89.4	94.0	89.4	93.8	91.5	92.4	94.2	93.3	95.4	93.1	94.2	94.7	94.2	94.2	94.7	95.6	94.2	94.2	95.2	94.2	94.2	94.7	**3**	Bucl13_Bps 1106a.seq
**4**	4.5	3.1	5.9		94.5	94.0	94.0	88.7	93.5	94.7	95.2	94.7	95.9	90.8	92.9	96.3	93.3	96.3	92.6	96.3	96.3	93.1	93.8	94.7	95.4	96.3	96.3	95.6	96.3	96.3	96.3	**4**	Bucl13_Bps 1026b.seq
**5**	3.6	1.6	4.7	3.6		99.3	94.9	89.9	92.6	93.1	95.6	93.1	94.0	90.8	90.8	92.2	91.7	93.1	92.9	92.2	93.8	93.8	92.6	93.5	94.2	92.2	92.2	92.9	92.2	92.2	93.8	**5**	Bucl13_Bps 1710b.seq
**6**	3.6	1.6	4.2	4.1	0.5		94.9	89.9	92.6	93.1	95.6	93.1	94.0	90.8	90.8	92.2	91.7	93.1	92.9	92.2	93.8	93.8	92.6	93.5	94.2	92.2	92.2	92.9	92.2	92.2	93.8	**6**	Bucl13_Bps BPC006.seq
**7**	4.9	3.1	5.0	3.4	4.6	4.6		92.4	93.1	93.8	94.0	93.8	95.2	91.2	94.2	92.4	94.2	93.1	92.4	92.4	93.1	91.5	94.7	94.5	93.8	92.4	92.4	92.4	92.4	92.4	93.1	**7**	Bucl13_Bps 668.seq
**8**	5.3	3.1	5.0	3.1	4.1	4.1	2.0		90.3	91.0	88.0	91.0	89.6	90.3	91.2	86.9	90.3	87.3	86.4	86.9	87.3	85.9	89.4	88.5	88.2	86.9	86.9	86.6	86.9	86.9	87.3	**8**	Bucl13_Bps NCTC 13179.seq
**9**	4.0	4.8	5.1	3.1	4.4	4.4	3.1	3.5		98.6	93.1	98.6	94.7	87.3	89.9	92.6	89.9	93.8	88.9	92.6	92.4	89.9	90.6	91.0	91.7	92.6	92.6	93.1	92.6	92.6	92.4	**9**	Bucl13_Bps MSHR146.seq
**10**	3.9	4.2	6.4	2.6	4.6	4.6	3.0	3.5	0.5		92.4	99.8	93.5	88.0	90.6	92.2	90.6	92.6	89.9	92.2	93.1	90.3	91.0	91.9	92.6	92.2	92.2	91.9	92.2	92.2	93.1	**10**	Bucl13_Bps MSHR511.seq
**11**	1.8	1.0	3.4	4.4	1.5	1.5	2.6	3.1	4.4	4.4		92.4	97.2	91.5	91.7	94.9	92.6	95.6	93.1	94.9	94.7	93.5	93.3	93.8	94.5	94.9	94.9	95.4	94.9	94.9	94.7	**11**	Bucl13_Bps MSHR520.seq
**12**	3.9	4.2	6.4	2.6	4.6	4.6	3.0	3.5	0.5	0.0	4.4		93.5	88.0	90.6	92.2	90.6	92.6	89.9	92.2	93.1	90.3	91.0	91.9	92.6	92.2	92.2	91.9	92.2	92.2	93.1	**12**	Bucl13_Bps NAU20B-16.seq
**13**	4.8	3.4	3.7	3.6	3.4	3.4	1.3	1.3	2.6	3.1	2.8	3.1		90.3	93.3	95.6	92.9	96.8	91.9	95.6	95.4	92.9	93.5	94.0	94.7	95.6	95.6	96.1	95.6	95.6	95.4	**13**	Bucl13_Bps NCTC 13178.seq
**14**	2.7	2.9	5.5	3.9	3.6	3.6	3.9	4.3	4.0	3.9	2.4	3.9	3.7		94.9	91.7	96.5	92.4	95.4	91.7	93.8	94.7	95.6	95.2	94.7	91.7	91.7	93.1	91.7	91.7	93.8	**14**	A8EP72_BURPS_Bucl13_Bps 406e.seq
**15**	5.4	3.4	5.3	2.3	4.4	4.4	1.3	2.5	1.8	1.8	2.9	1.8	1.0	4.6		93.8	97.2	94.5	93.8	93.8	94.5	92.9	97.0	95.9	95.2	93.8	93.8	93.8	93.8	93.8	94.5	**15**	B2HC24_BURPS_Bucl13_Bps 1655.seq
**16**	4.0	2.9	4.0	0.8	3.2	3.2	2.1	2.1	2.6	2.4	3.2	2.4	2.4	2.9	1.3		94.2	98.6	93.5	99.8	97.0	94.0	94.9	95.6	96.3	99.8	99.8	97.9	99.8	99.8	97.0	**16**	Bucl13_Bm 2000031281.seq
**17**	3.0	3.1	5.0	2.6	4.2	4.2	2.0	2.8	2.6	2.6	2.6	2.6	2.4	2.0	2.0	1.6		94.9	95.6	94.2	96.3	94.7	98.6	97.7	97.0	94.2	94.2	95.6	94.2	94.2	96.3	**17**	Bucl13_Bm 2002721280.seq
**18**	4.0	3.7	3.4	1.6	2.9	2.9	2.1	2.4	2.1	2.6	3.2	2.6	1.8	2.9	1.3	0.5	1.6		94.0	98.6	97.9	94.9	95.6	96.1	96.8	98.6	98.6	99.1	98.6	98.6	97.9	**18**	Bucl13_Bm A188.seq
**19**	3.9	3.9	6.9	5.0	4.4	4.4	5.2	5.8	5.3	5.0	3.7	5.0	5.1	1.8	4.4	4.0	3.1	4.2		93.5	95.6	98.6	96.5	97.7	96.8	93.5	93.5	94.7	93.5	93.5	95.6	**19**	Bucl13_Bm A193.seq
**20**	4.0	2.9	4.0	0.8	3.2	3.2	2.1	2.1	2.6	2.4	3.2	2.4	2.4	2.9	1.3	0.0	1.6	0.5	4.0		97.0	94.0	94.9	95.6	96.3	99.8	99.8	97.9	99.8	99.8	97.0	**20**	Bucl13_Bm China 7.seq
**21**	3.1	3.4	5.0	2.3	2.9	2.9	2.9	3.1	2.9	2.9	3.4	2.9	2.6	2.1	2.1	1.6	0.8	1.3	3.1	1.6		96.5	96.8	97.7	98.4	97.0	97.0	98.6	97.0	97.0	99.8	**21**	Bucl13_Bm PRL-20.seq
**22**	3.9	4.2	6.4	5.3	3.6	3.6	5.5	5.5	5.1	5.3	4.0	5.3	4.8	1.8	4.7	4.3	3.4	4.0	0.5	4.3	2.9		95.6	96.5	97.5	94.0	94.0	95.6	94.0	94.0	96.5	**22**	Bucl13_Bm strain_6.seq
**23**	3.3	3.4	4.7	2.9	3.9	3.9	2.3	3.1	2.6	2.9	2.6	2.9	2.4	2.3	1.5	1.6	0.5	1.6	2.8	1.6	1.0	3.1		98.6	97.9	94.9	94.9	96.3	94.9	94.9	96.8	**23**	Bucl13_Bm strain_11.seq
**24**	3.1	3.1	5.0	2.6	3.6	3.6	2.8	3.4	2.9	2.6	2.9	2.6	2.6	2.0	2.0	1.6	0.8	1.8	2.3	1.6	0.8	2.8	0.5		98.8	95.6	95.6	96.8	95.6	95.6	97.7	**24**	Bucl13_Bm NCTC 10229.seq
**25**	2.8	3.1	4.7	2.6	3.1	3.1	2.8	2.8	2.9	2.6	2.9	2.6	2.6	1.8	2.1	1.6	0.8	1.8	2.6	1.6	0.8	2.6	0.5	0.3		96.3	96.3	97.5	96.3	96.3	98.4	**25**	Bucl13_Bm NCTC 10247.seq
**26**	4.0	2.9	4.0	0.8	3.2	3.2	2.1	2.1	2.6	2.4	3.2	2.4	2.4	2.9	1.3	0.0	1.6	0.5	4.0	0.0	1.6	4.3	1.6	1.6	1.6		99.8	97.9	99.8	99.8	97.0	**26**	Bucl13_Bm FMH.seq
**27**	4.0	2.9	4.0	0.8	3.2	3.2	2.1	2.1	2.6	2.4	3.2	2.4	2.4	2.9	1.3	0.0	1.6	0.5	4.0	0.0	1.6	4.3	1.6	1.6	1.6	0.0		97.9	99.8	99.8	97.0	**27**	Bucl13_Bm JHU.seq
**28**	3.2	4.0	3.7	2.4	3.2	3.2	2.9	3.2	2.9	3.4	3.4	3.4	2.6	2.1	2.1	1.3	0.8	0.8	3.4	1.3	0.5	3.2	0.8	1.0	1.0	1.3	1.3		97.9	97.9	98.6	**28**	Bucl13_Bm ATCC 10399.seq
**29**	4.0	2.9	4.0	0.8	3.2	3.2	2.1	2.1	2.6	2.4	3.2	2.4	2.4	2.9	1.3	0.0	1.6	0.5	4.0	0.0	1.6	4.3	1.6	1.6	1.6	0.0	0.0	1.3		99.8	97.0	**29**	Bucl13_Bm GB8 horse 4.seq
**30**	4.0	2.9	4.0	0.8	3.2	3.2	2.1	2.1	2.6	2.4	3.2	2.4	2.4	2.9	1.3	0.0	1.6	0.5	4.0	0.0	1.6	4.3	1.6	1.6	1.6	0.0	0.0	1.3	0.0		97.0	**30**	Bucl13_Bm ATCC 23344.seq
**31**	3.1	3.4	5.0	2.3	2.9	2.9	2.9	3.1	2.9	2.9	3.4	2.9	2.6	2.1	2.1	1.6	0.8	1.3	3.1	1.6	0.0	2.9	1.0	0.8	0.8	1.6	1.6	0.5	1.6	1.6		**31**	Bucl13_Bm SAVP1.seq
	**1**	**2**	**3**	**4**	**5**	**6**	**7**	**8**	**9**	**10**	**11**	**12**	**13**	**14**	**15**	**16**	**17**	**18**	**19**	**20**	**21**	**22**	**23**	**24**	**25**	**26**	**27**	**28**	**29**	**30**	**31**		

bucl13

Percent Identity

	1	2	3	4	5	6	7	8	9	10	11	12	13	14	15	16	17	18	19	20	21	22	23	24	25	26	27	28	29	30		
**1**		43.4	50.2	48.8	42.0	43.4	46.2	49.3	49.1	46.2	46.2	47.7	47.7	47.7	46.2	46.2	46.2	46.2	46.2	46.2	50.5	49.1	51.6	51.6	42.0	51.6	43.9	43.6	43.4	42.4	**1**	A3N630_BURP6_Bucl14_Bps 668.seq
**2**	4.0		92.0	93.6	97.6	99.7	96.0	92.5	92.7	96.0	96.0	94.3	94.3	94.3	96.0	96.0	96.0	96.0	95.8	96.0	91.1	92.7	90.5	90.5	97.6	90.5	99.1	99.5	99.7	97.2	**2**	Bucl14_Bps 1026b.seq
**3**	2.4	4.0		97.4	90.6	92.0	94.6	98.8	98.6	94.6	94.6	96.4	96.4	96.4	94.6	94.6	94.6	94.6	94.6	94.6	96.4	98.6	97.0	97.0	90.6	97.0	92.5	92.2	92.0	91.0	**3**	Bucl14_Bps 1106a.seq
**4**	2.1	4.0	2.1		92.2	93.6	96.5	96.9	96.7	96.5	96.5	99.0	99.0	99.0	96.5	96.5	96.5	96.5	96.5	96.5	95.1	96.7	95.5	95.5	92.2	95.5	94.1	93.8	93.6	92.5	**4**	Bucl14_Bps BPC006.seq
**5**	3.7	2.0	3.7	3.7		97.6	94.3	91.0	91.0	94.3	94.3	92.5	92.5	92.5	94.3	94.3	94.3	94.3	94.1	94.3	89.4	91.0	89.1	89.1	100.0	89.1	96.9	97.0	97.6	99.3	**5**	Bucl14_Bps MSHR 305.seq
**6**	4.0	0.8	4.0	4.0	2.0		96.0	92.5	92.7	96.0	96.0	94.3	94.3	94.3	96.0	96.0	96.0	96.0	95.8	96.0	91.1	92.7	90.5	90.5	97.6	90.5	99.1	99.5	100.0	97.2	**6**	Bucl14_Bps NCTC 13179.seq
**7**	4.5	2.0	4.9	4.1	2.4	2.0		95.8	96.0	100.0	100.0	97.6	97.6	97.6	100.0	100.0	100.0	100.0	99.8	100.0	94.4	96.0	93.1	93.1	94.3	93.1	95.8	95.8	96.0	94.3	**7**	Bucl14_Bm ATCC 23344.seq
**8**	4.2	2.8	2.4	3.2	2.9	2.8	2.2		99.8	95.8	95.8	97.6	97.6	97.6	95.8	95.8	95.8	95.8	95.7	95.8	97.6	99.8	95.8	95.8	91.0	95.8	92.4	92.4	92.5	91.0	**8**	Bucl14_Bm NCTC 10229.seq
**9**	4.6	2.4	2.8	3.6	2.9	2.4	1.8	0.3		96.0	96.0	97.7	97.7	97.7	96.0	96.0	96.0	96.0	95.8	96.0	97.7	100.0	95.7	95.7	91.0	95.7	92.5	92.5	92.7	91.0	**9**	Bucl14_Bm NCTC 10247.seq
**10**	4.5	2.0	4.9	4.1	2.4	2.0	0.0	2.2	1.8		100.0	97.6	97.6	97.6	100.0	100.0	100.0	100.0	99.8	100.0	94.4	96.0	93.1	93.1	94.3	93.1	95.8	95.8	96.0	94.3	**10**	Bucl14_Bm SAVP1.seq
**11**	4.5	2.0	4.9	4.1	2.4	2.0	0.0	2.2	1.8	0.0		97.6	97.6	97.6	100.0	100.0	100.0	100.0	99.8	100.0	94.4	96.0	93.1	93.1	94.3	93.1	95.8	95.8	96.0	94.3	**11**	Bucl14_Bm 2000031281.seq
**12**	4.4	2.4	4.4	2.1	2.9	2.4	1.8	1.8	1.4	1.8	1.8		100.0	100.0	97.6	97.6	97.6	97.6	97.4	97.6	96.2	97.7	94.4	94.4	92.5	94.4	94.1	94.1	94.3	92.5	**12**	Bucl14_Bm 2002721280.seq
**13**	4.4	2.4	4.4	2.1	2.9	2.4	1.8	1.8	1.4	1.8	1.8	0.0		100.0	97.6	97.6	97.6	97.6	97.4	97.6	96.2	97.7	94.4	94.4	92.5	94.4	94.1	94.1	94.3	92.5	**13**	Bucl14_Bm A188.seq
**14**	4.4	2.4	4.4	2.1	2.9	2.4	1.8	1.8	1.4	1.8	1.8	0.0	0.0		97.6	97.6	97.6	97.6	97.4	97.6	96.2	97.7	94.4	94.4	92.5	94.4	94.1	94.1	94.3	92.5	**14**	Bucl14_Bm A193.seq
**15**	4.5	2.0	4.9	4.1	2.4	2.0	0.0	2.2	1.8	0.0	0.0	1.8	1.8	1.8		100.0	100.0	100.0	99.8	100.0	94.4	96.0	93.1	93.1	94.3	93.1	95.8	95.8	96.0	94.3	**15**	Bucl14_Bm ATCC 10399.seq
**16**	4.5	2.0	4.9	4.1	2.4	2.0	0.0	2.2	1.8	0.0	0.0	1.8	1.8	1.8	0.0		100.0	100.0	99.8	100.0	94.4	96.0	93.1	93.1	94.3	93.1	95.8	95.8	96.0	94.3	**16**	Bucl14_Bm China 7.seq
**17**	4.5	2.0	4.9	4.1	2.4	2.0	0.0	2.2	1.8	0.0	0.0	1.8	1.8	1.8	0.0	0.0		100.0	99.8	100.0	94.4	96.0	93.1	93.1	94.3	93.1	95.8	95.8	96.0	94.3	**17**	Bucl14_Bm FMH.seq
**18**	4.5	2.0	4.9	4.1	2.4	2.0	0.0	2.2	1.8	0.0	0.0	1.8	1.8	1.8	0.0	0.0	0.0		99.8	100.0	94.4	96.0	93.1	93.1	94.3	93.1	95.8	95.8	96.0	94.3	**18**	Bucl14_Bm GB8 horse 4.seq
**19**	4.1	2.0	4.5	3.7	2.5	2.0	0.0	2.2	1.8	0.0	0.0	1.8	1.8	1.8	0.0	0.0	0.0	0.0		99.8	94.3	95.8	93.1	93.1	94.1	93.1	95.8	95.7	95.8	94.1	**19**	Bucl14_Bm JHU.seq
**20**	4.5	2.0	4.9	4.1	2.4	2.0	0.0	2.2	1.8	0.0	0.0	1.8	1.8	1.8	0.0	0.0	0.0	0.0	0.0		94.4	96.0	93.1	93.1	94.3	93.1	95.8	95.8	96.0	94.3	**20**	Bucl14_Bm PRL-20.seq
**21**	4.8	2.4	4.2	3.6	2.9	2.4	1.8	1.7	1.4	1.8	1.8	1.4	1.4	1.4	1.8	1.8	1.8	1.8	1.8	1.8		97.7	97.9	97.9	89.4	97.9	91.0	91.0	91.1	89.4	**21**	Bucl14_Bm strain_6.seq
**22**	4.6	2.4	2.8	3.6	2.9	2.4	1.8	0.3	0.0	1.8	1.8	1.4	1.4	1.4	1.8	1.8	1.8	1.8	1.8	1.8	1.4		95.7	95.7	91.0	95.7	92.5	92.5	92.7	91.0	**22**	Bucl14_Bm strain_11.seq
**23**	2.7	4.0	2.8	2.9	3.7	4.0	4.9	5.3	5.7	4.9	4.9	5.1	5.1	5.1	4.9	4.9	4.9	4.9	4.5	4.9	4.1	5.7		100.0	89.1	100.0	91.0	90.6	90.5	89.4	**23**	Bucl14_Bps MSHR146.seq
**24**	2.7	4.0	2.8	2.9	3.7	4.0	4.9	5.3	5.7	4.9	4.9	5.1	5.1	5.1	4.9	4.9	4.9	4.9	4.5	4.9	4.1	5.7	0.0		89.1	100.0	91.0	90.6	90.5	89.4	**24**	Bucl14_Bps MSHR511.seq
**25**	3.7	2.0	3.7	3.7	0.0	2.0	2.4	2.9	2.9	2.4	2.4	2.9	2.9	2.9	2.4	2.4	2.4	2.4	2.5	2.4	2.9	2.9	3.7	3.7		89.1	96.9	97.0	97.6	99.3	**25**	Bucl14_Bps MSHR520.seq
**26**	2.7	4.0	2.8	2.9	3.7	4.0	4.9	5.3	5.7	4.9	4.9	5.1	5.1	5.1	4.9	4.9	4.9	4.9	4.5	4.9	4.1	5.7	0.0	0.0	3.7		91.0	90.6	90.5	89.4	**26**	Bucl14_Bps NAU20B-16.seq
**27**	2.8	2.0	2.8	2.8	3.7	2.0	2.4	3.2	2.8	2.4	2.4	2.8	2.8	2.8	2.4	2.4	2.4	2.4	2.0	2.4	2.8	2.8	2.8	2.8	3.7	2.8		99.3	99.1	97.2	**27**	Bucl14_Bps NCTC 13178.seq
**28**	3.6	1.2	3.6	3.6	3.3	1.2	2.4	3.2	2.8	2.4	2.4	2.8	2.8	2.8	2.4	2.4	2.4	2.4	2.4	2.4	2.8	2.8	3.6	3.6	3.3	3.6	1.6		99.5	97.4	**28**	Bucl14_Bps 1710b.seq
**29**	4.0	0.8	4.0	4.0	2.0	0.0	2.0	2.8	2.4	2.0	2.0	2.4	2.4	2.4	2.0	2.0	2.0	2.0	2.0	2.0	2.4	2.4	4.0	4.0	2.0	4.0	2.0	1.2		97.2	**29**	Bucl14_Bps K96243.seq
**30**	2.9	2.9	2.9	2.9	1.6	2.9	2.4	2.9	2.9	2.4	2.4	2.9	2.9	2.9	2.4	2.4	2.4	2.4	2.5	2.4	2.9	2.9	2.9	2.9	1.6	2.9	2.9	2.4	2.9		**30**	Bucl14_Bps MSHR 346.seq
	**1**	**2**	**3**	**4**	**5**	**6**	**7**	**8**	**9**	**10**	**11**	**12**	**13**	**14**	**15**	**16**	**17**	**18**	**19**	**20**	**21**	**22**	**23**	**24**	**25**	**26**	**27**	**28**	**29**	**30**		

bucl14

Percent Identity

	1	2	3	4	5	6	7	8	9	10	11	12	13	14	15	16	17	18	19	20	21	22	23	24	25	26	27	28		
**1**		95.5	89.9	100.0	95.0	89.9	71.7	77.3	70.6	95.5	72.8	74.5	94.1	91.6	94.1	91.6	94.1	91.3	91.6	91.6	91.6	91.6	94.1	94.1	91.3	94.1	94.1	91.3	**1**	Bucl15_Bps_K96243.seq
**2**	3.9		93.6	95.5	98.0	93.6	73.7	75.6	68.9	100.0	71.1	72.3	89.9	87.4	89.9	87.4	89.9	87.7	87.7	87.4	87.4	87.4	89.9	89.9	87.7	89.9	89.9	87.7	**2**	Bucl15_Bps_MSHR_305.seq
**3**	6.2	3.6		89.9	93.3	100.0	75.1	76.2	69.5	93.6	71.7	67.2	85.2	82.6	85.2	82.6	85.2	82.6	82.9	82.6	82.6	82.6	85.2	85.2	82.6	85.2	85.2	82.6	**3**	Bucl15_Bps_1106a.seq
**4**	0.0	3.9	6.2		95.0	89.9	71.7	77.3	70.6	95.5	72.8	74.5	94.1	91.6	94.1	91.6	94.1	91.3	91.6	91.6	91.6	91.6	94.1	94.1	91.3	94.1	94.1	91.3	**4**	Bucl15_Bps_1026b.seq
**5**	5.0	3.9	4.3	5.0		93.3	73.1	75.1	68.3	98.0	70.6	73.1	89.4	86.8	89.4	86.8	89.4	86.6	86.6	86.8	86.8	86.8	89.4	89.4	86.6	89.4	89.4	86.6	**5**	Bucl15_Bps_1710b.seq
**6**	6.2	3.6	0.0	6.2	4.3		75.1	76.2	69.5	93.6	71.7	67.2	85.2	82.6	85.2	82.6	85.2	82.6	82.9	82.6	82.6	82.6	85.2	85.2	82.6	85.2	85.2	82.6	**6**	Bucl15_Bps_BPC006.seq
**7**	7.7	7.7	4.3	7.7	9.0	4.3		51.8	45.1	73.7	47.3	48.7	71.7	73.9	71.7	73.9	71.7	74.2	69.7	73.9	73.9	73.9	71.7	71.7	74.2	71.7	71.7	74.2	**7**	A3NDA8_BURP6_Bucl15_Bps_668.seq
**8**	3.9	3.5	3.0	3.9	4.7	3.0	6.2		93.3	75.6	95.5	86.8	76.5	73.9	76.5	73.9	76.5	73.9	78.4	73.9	73.9	73.9	76.5	76.5	73.9	76.5	76.5	73.9	**8**	Bucl15_Bps_MSHR146.seq
**9**	3.9	3.5	3.0	3.9	4.7	3.0	6.2	0.0		68.9	97.8	88.8	69.7	67.2	69.7	67.2	69.7	67.2	71.7	67.2	67.2	67.2	69.7	69.7	67.2	69.7	69.7	67.2	**9**	Bucl15_Bps_MSHR511.seq
**10**	3.9	0.0	3.6	3.9	3.9	3.6	7.7	3.5	3.5		71.1	72.3	89.9	87.4	89.9	87.4	89.9	87.7	87.7	87.4	87.4	87.4	89.9	89.9	87.7	89.9	89.9	87.7	**10**	Bucl15_Bps_MSHR520.seq
**11**	3.9	3.5	3.0	3.9	4.7	3.0	6.2	0.0	0.0	3.5		91.0	72.0	69.5	72.0	69.5	72.0	69.5	73.9	69.5	69.5	69.5	72.0	72.0	69.5	72.0	72.0	69.5	**11**	Bucl15_Bps_NAU20B-16.seq
**12**	5.8	5.6	6.8	5.8	3.8	6.8	8.9	8.9	8.8	5.6	8.8		72.8	70.3	72.8	70.3	72.8	70.0	74.5	70.3	70.3	70.3	72.8	72.8	70.0	72.8	72.8	70.0	**12**	Bucl15_Bps_NCTC_13178.seq
**13**	2.1	5.6	6.3	2.1	6.8	6.3	7.9	4.8	4.8	5.6	4.8	8.3		96.9	100.0	96.9	100.0	96.6	96.6	96.9	96.9	96.9	100.0	100.0	96.6	100.0	100.0	96.6	**13**	Bucl15_Bm_2000031281.seq
**14**	2.1	5.6	6.3	2.1	6.8	6.3	8.1	4.8	4.8	5.6	4.8	8.3	0.9		96.9	100.0	96.9	99.7	93.6	100.0	100.0	100.0	96.9	96.9	99.7	96.9	96.9	99.7	**14**	Bucl15_Bm_2002721280.seq
**15**	2.1	5.6	6.3	2.1	6.8	6.3	7.9	4.8	4.8	5.6	4.8	8.3	0.0	0.9		96.9	100.0	96.6	96.6	96.9	96.9	96.9	100.0	100.0	96.6	100.0	100.0	96.6	**15**	Bucl15_Bm_A188.seq
**16**	2.1	5.6	6.3	2.1	6.8	6.3	8.1	4.8	4.8	5.6	4.8	8.3	0.9	0.0	0.9		96.9	99.7	93.6	100.0	100.0	100.0	96.9	96.9	99.7	96.9	96.9	99.7	**16**	Bucl15_Bm_A193.seq
**17**	2.1	5.6	6.3	2.1	6.8	6.3	7.9	4.8	4.8	5.6	4.8	8.3	0.0	0.9	0.0	0.9		96.6	96.6	96.9	96.9	96.9	100.0	100.0	96.6	100.0	100.0	96.6	**17**	Bucl15_Bm_China_7.seq
**18**	2.6	5.0	6.3	2.6	7.4	6.3	7.5	4.8	4.8	5.0	4.8	8.9	1.4	0.5	1.4	0.5	1.4		93.8	99.7	99.7	99.7	96.6	96.6	100.0	96.6	96.6	100.0	**18**	Bucl15_Bm_PRL-20.seq
**19**	2.6	5.6	6.3	2.6	8.0	6.3	7.3	5.2	5.2	5.6	5.2	9.0	1.9	2.9	1.9	2.9	1.9	2.4		93.6	93.6	93.6	96.6	96.6	93.8	96.6	96.6	93.8	**19**	Bucl15_Bm_strain_6.seq
**20**	2.1	5.6	6.3	2.1	6.8	6.3	8.1	4.8	4.8	5.6	4.8	8.3	0.9	0.0	0.9	0.0	0.9	0.5	2.9		100.0	100.0	96.9	96.9	99.7	96.9	96.9	99.7	**20**	Bucl15_Bm_strain_11.seq
**21**	2.1	5.6	6.3	2.1	6.8	6.3	8.1	4.8	4.8	5.6	4.8	8.3	0.9	0.0	0.9	0.0	0.9	0.5	2.9	0.0		100.0	96.9	96.9	99.7	96.9	96.9	99.7	**21**	Bucl15_Bm_NCTC_10229.seq
**22**	2.1	5.6	6.3	2.1	6.8	6.3	8.1	4.8	4.8	5.6	4.8	8.3	0.9	0.0	0.9	0.0	0.9	0.5	2.9	0.0	0.0		96.9	96.9	99.7	96.9	96.9	99.7	**22**	Bucl15_Bm_NCTC_10247.seq
**23**	2.1	5.6	6.3	2.1	6.8	6.3	7.9	4.8	4.8	5.6	4.8	8.3	0.0	0.9	0.0	0.9	0.0	1.4	1.9	0.9	0.9	0.9		100.0	96.6	100.0	100.0	96.6	**23**	Bucl15_Bm_JHU.seq
**24**	2.1	5.6	6.3	2.1	6.8	6.3	7.9	4.8	4.8	5.6	4.8	8.3	0.0	0.9	0.0	0.9	0.0	1.4	1.9	0.9	0.9	0.9	0.0		96.6	100.0	100.0	96.6	**24**	Bucl15_Bm_FMH.seq
**25**	2.6	5.0	6.3	2.6	7.4	6.3	7.5	4.8	4.8	5.0	4.8	8.9	1.4	0.5	1.4	0.5	1.4	0.0	2.4	0.5	0.5	0.5	1.4	1.4		96.6	96.6	100.0	**25**	Bucl15_Bm_ATCC_10399.seq
**26**	2.1	5.6	6.3	2.1	6.8	6.3	7.9	4.8	4.8	5.6	4.8	8.3	0.0	0.9	0.0	0.9	0.0	1.4	1.9	0.9	0.9	0.9	0.0	0.0	1.4		100.0	96.6	**26**	Bucl15_Bm_GB8_horse_4.seq
**27**	2.1	5.6	6.3	2.1	6.8	6.3	7.9	4.8	4.8	5.6	4.8	8.3	0.0	0.9	0.0	0.9	0.0	1.4	1.9	0.9	0.9	0.9	0.0	0.0	1.4	0.0		96.6	**27**	Bucl15_Bm_ATCC_23344.seq
**28**	2.6	5.0	6.3	2.6	7.4	6.3	7.5	4.8	4.8	5.0	4.8	8.9	1.4	0.5	1.4	0.5	1.4	0.0	2.4	0.5	0.5	0.5	1.4	1.4	0.0	1.4	1.4		**28**	Bucl15_Bm_SAVP1.seq
	**1**	**2**	**3**	**4**	**5**	**6**	**7**	**8**	**9**	**10**	**11**	**12**	**13**	**14**	**15**	**16**	**17**	**18**	**19**	**20**	**21**	**22**	**23**	**24**	**25**	**26**	**27**	**28**		

bucl15

Percent Identity

	1	2	3	4	5	6	7	8	9	10	11	12	13	14	15	16	17	18	19	20	21	22	23	24	25	26	27	28	29	30		
**1**		86.3	79.7	86.2	82.3	88.5	79.7	86.2	88.5	86.2	83.3	81.4	85.2	90.6	85.7	84.7	85.7	90.5	85.7	83.7	85.7	85.7	73.0	76.9	84.7	84.7	77.2	84.7	84.7	82.7	**1**	Bucl16_Bps K96243.seq
**2**	1.5		90.1	86.3	91.9	94.0	90.1	86.3	92.3	95.1	94.2	94.8	96.0	90.9	96.3	95.3	96.3	92.6	96.3	94.3	96.3	96.3	83.6	87.5	95.3	95.3	87.8	95.3	95.3	93.3	**2**	Bucl16_Bps MSHR 305.seq
**3**	1.2	3.7		78.7	96.8	85.8	100.0	78.7	86.5	86.5	88.6	94.0	87.2	84.5	91.6	92.6	91.6	86.7	91.6	93.5	91.6	91.6	92.6	96.4	92.6	92.6	96.8	92.6	92.6	94.5	**3**	A3NSP4_BURP0_Bucl16_Bp 1106a.seq
**4**	3.2	4.1	5.1		80.7	88.8	78.7	100.0	90.8	86.4	83.4	81.6	85.4	92.6	86.6	85.7	86.6	91.5	86.6	84.7	86.6	86.6	74.0	77.9	85.7	85.7	79.5	85.7	85.7	83.7	**4**	Bucl16_Bps MSHR346.seq
**5**	0.3	3.8	1.4	5.1		87.6	96.8	80.7	88.0	89.0	91.0	95.9	89.6	86.3	93.0	94.0	93.0	88.1	93.0	94.9	93.0	93.0	90.1	94.0	94.0	94.0	94.3	94.0	94.0	95.9	**5**	Bucl16_Bps 1026b.seq
**6**	2.5	3.9	5.7	4.7	5.8		85.8	88.8	93.3	95.8	92.8	89.0	94.8	91.5	91.3	90.3	91.3	93.2	91.3	89.3	91.3	91.3	78.6	82.5	90.3	90.3	83.3	90.3	90.3	88.3	**6**	Bucl16_Bps 1710b.seq
**7**	1.2	3.7	0.0	5.1	1.4	5.7		78.7	86.5	86.5	88.6	94.0	87.2	84.5	91.6	92.6	91.6	86.7	91.6	93.5	91.6	91.6	92.6	96.4	92.6	92.6	96.8	92.6	92.6	94.5	**7**	Bucl16_Bps BPC006.seq
**8**	3.2	4.1	5.1	0.0	5.1	4.7	5.1		90.8	86.4	83.4	81.6	85.4	92.6	86.6	85.7	86.6	91.5	86.6	84.7	86.6	86.6	74.0	77.9	85.7	85.7	79.5	85.7	85.7	83.7	**8**	Bucl16_Bps 668.seq
**9**	2.5	3.5	2.3	3.6	2.8	6.0	2.3	3.6		91.2	88.3	87.6	90.2	95.7	94.2	93.2	94.2	97.1	94.2	92.2	94.2	94.2	81.6	85.4	93.2	93.2	86.2	93.2	93.2	91.3	**9**	Bucl16_Bps NCTC 13179.seq
**10**	3.0	4.7	7.0	5.3	6.3	2.8	7.0	5.3	6.3		97.0	90.2	99.0	89.9	92.4	91.5	92.4	91.2	92.4	90.5	92.4	92.4	79.8	83.7	91.5	91.5	84.7	91.5	91.5	89.5	**10**	Bucl16_Bps MSHR146.seq
**11**	3.0	4.6	7.9	5.4	7.2	2.9	7.9	5.4	6.1	0.1		92.9	98.0	87.1	91.6	92.2	91.6	88.3	91.6	92.9	91.6	91.6	81.9	85.8	92.2	92.2	86.7	92.2	92.2	91.6	**11**	Bucl16_Bps MSHR511.seq
**12**	1.5	0.4	4.6	3.9	4.6	4.0	4.6	3.9	3.3	4.8	4.9		91.0	86.2	91.6	92.3	91.6	87.8	91.6	93.1	91.6	91.6	87.5	91.4	92.3	92.3	91.7	92.3	92.3	93.9	**12**	Bucl16_Bps MSHR520.seq
**13**	3.0	4.8	7.4	5.3	6.7	2.8	7.4	5.3	6.3	0.0	0.1	4.9		88.9	93.4	92.1	93.4	90.2	93.4	91.2	93.4	93.4	80.5	84.4	92.1	92.1	85.3	92.1	92.1	90.2	**13**	Bucl16_Bps NAU20B-16.seq
**14**	2.2	2.9	2.5	4.8	2.6	6.0	2.5	4.8	2.2	5.6	5.4	2.7	5.6		92.3	91.4	92.3	97.2	92.3	90.4	92.3	92.3	79.7	83.6	91.4	91.4	83.9	91.4	91.4	89.4	**14**	Bucl16_Bps NCTC 13178.seq
**15**	1.5	3.6	1.1	4.5	1.8	6.5	1.1	4.5	2.0	7.3	7.1	3.5	7.2	1.9		98.7	100.0	94.8	100.0	97.7	100.0	100.0	87.1	90.9	98.7	98.7	91.3	98.7	98.7	96.8	**15**	Bucl16_Bm 2000031281.seq
**16**	1.5	3.7	1.1	4.5	1.8	6.5	1.1	4.5	2.0	7.3	7.4	3.7	7.7	1.9	0.4		98.7	93.9	98.7	98.7	98.7	98.7	88.0	91.9	100.0	100.0	92.2	100.0	100.0	97.7	**16**	Bucl16_Bm 2002721280.seq
**17**	1.5	3.6	1.1	4.5	1.8	6.5	1.1	4.5	2.0	7.3	7.1	3.5	7.2	1.9	0.0	0.4		94.8	100.0	97.7	100.0	100.0	87.1	90.9	98.7	98.7	91.3	98.7	98.7	96.8	**17**	Bucl16_Bm A188.seq
**18**	1.5	2.4	1.2	4.5	1.9	5.4	1.2	4.5	2.0	5.5	5.4	2.3	5.5	1.9	0.4	0.4	0.4		94.8	92.9	94.8	94.8	82.2	86.1	93.9	93.9	86.4	93.9	93.9	91.9	**18**	Bucl16_Bm A193.seq
**19**	1.5	3.6	1.1	4.5	1.8	6.5	1.1	4.5	2.0	7.3	7.1	3.5	7.2	1.9	0.0	0.4	0.0	0.4		97.7	100.0	100.0	87.1	90.9	98.7	98.7	91.3	98.7	98.7	96.8	**19**	Bucl16_Bm China 7.seq
**20**	1.5	3.7	1.1	4.5	1.7	6.5	1.1	4.5	2.0	7.3	7.8	3.9	7.7	1.9	0.4	0.4	0.4	0.4	0.4		97.7	97.7	89.0	92.9	98.7	98.7	93.2	98.7	98.7	98.7	**20**	Bucl16_Bm PRL-20.seq
**21**	1.5	3.6	1.1	4.5	1.8	6.5	1.1	4.5	2.0	7.3	7.1	3.5	7.2	1.9	0.0	0.4	0.0	0.4	0.0	0.4		100.0	87.1	90.9	98.7	98.7	91.3	98.7	98.7	96.8	**21**	Bucl16_Bm strain_6.seq
**22**	1.5	3.6	1.1	4.5	1.8	6.5	1.1	4.5	2.0	7.3	7.1	3.5	7.2	1.9	0.0	0.4	0.0	0.4	0.0	0.4	0.0		87.1	90.9	98.7	98.7	91.3	98.7	98.7	96.8	**22**	Bucl16_Bm strain_11.seq
**23**	1.5	3.7	1.0	4.5	1.7	6.5	1.0	4.5	2.0	7.3	8.2	4.6	7.7	1.9	0.4	0.4	0.4	0.4	0.4	0.4	0.4	0.4		95.8	88.0	88.0	94.2	88.0	88.0	90.0	**23**	Bucl16_Bm NCTC 10229.seq
**24**	1.5	3.7	1.0	4.5	1.7	6.5	1.0	4.5	2.0	7.3	8.2	4.6	7.7	1.9	0.4	0.4	0.4	0.4	0.4	0.4	0.4	0.4	0.3		91.9	91.9	98.1	91.9	91.9	93.9	**24**	Bucl16_Bm NCTC 10247.seq
**25**	1.5	3.7	1.1	4.5	1.8	6.5	1.1	4.5	2.0	7.3	7.4	3.7	7.7	1.9	0.4	0.0	0.4	0.4	0.4	0.4	0.4	0.4	0.4	0.4		100.0	92.2	100.0	100.0	97.7	**25**	Bucl16_Bm FMH.seq
**26**	1.5	3.7	1.1	4.5	1.8	6.5	1.1	4.5	2.0	7.3	7.4	3.7	7.7	1.9	0.4	0.0	0.4	0.4	0.4	0.4	0.4	0.4	0.4	0.4	0.0		92.2	100.0	100.0	97.7	**26**	Bucl16_Bm JHU.seq
**27**	2.4	4.5	1.8	3.6	2.4	6.8	1.8	3.6	2.2	7.3	8.2	5.4	7.7	2.7	1.1	1.1	1.1	1.2	1.1	1.1	1.1	1.1	1.0	1.0	1.1	1.1		92.2	92.2	94.2	**27**	Bucl16_Bm ATCC 10399.seq
**28**	1.5	3.7	1.1	4.5	1.8	6.5	1.1	4.5	2.0	7.3	7.4	3.7	7.7	1.9	0.4	0.0	0.4	0.4	0.4	0.4	0.4	0.4	0.4	0.4	0.0	0.0	1.1		100.0	97.7	**28**	Bucl16_Bm GB8 horse 4.seq
**29**	1.5	3.7	1.1	4.5	1.8	6.5	1.1	4.5	2.0	7.3	7.4	3.7	7.7	1.9	0.4	0.0	0.4	0.4	0.4	0.4	0.4	0.4	0.4	0.4	0.0	0.0	1.1	0.0		97.7	**29**	Bucl16_Bm ATCC 23344.seq
**30**	1.5	3.7	1.1	4.5	1.7	6.5	1.1	4.5	2.0	7.3	8.2	4.1	7.7	1.9	0.4	0.4	0.4	0.4	0.4	0.4	0.4	0.4	0.4	0.4	0.4	0.4	1.1	0.4	0.4		**30**	Bucl16_Bm SAVP1.seq
	**1**	**2**	**3**	**4**	**5**	**6**	**7**	**8**	**9**	**10**	**11**	**12**	**13**	**14**	**15**	**16**	**17**	**18**	**19**	**20**	**21**	**22**	**23**	**24**	**25**	**26**	**27**	**28**	**29**	**30**		

bucl16

Bucl No.	% identity range	Average % identity
1	73.1-100	90.37
2	74.1-100	89.96
3	66.4-99.8	85.95
4	70.4-100	89.88
5	69.2-99.6	88.36
6	43.1-100	76.45
7	83.6-99.5	94.91
8	78.3-100	94.14
10	58.9-100	84.63
13	86.9-99.8	93.87
14	42-100	92.17
15	45.1-100	86.36
16	73-100	91.17
