# Supplementary figures and images for: A Unique Set of the Burkholderia Collagen-Like Proteins Provides Insight into Pathogenesis, Genome Evolution and Niche Adaptation, and Infection Detection
Source: PLoS One. 2015 Sep 10;10(9):e0137578. doi: 10.1371/journal.pone.0137578 (PMC4565658; doi:10.1371/journal.pone.0137578)

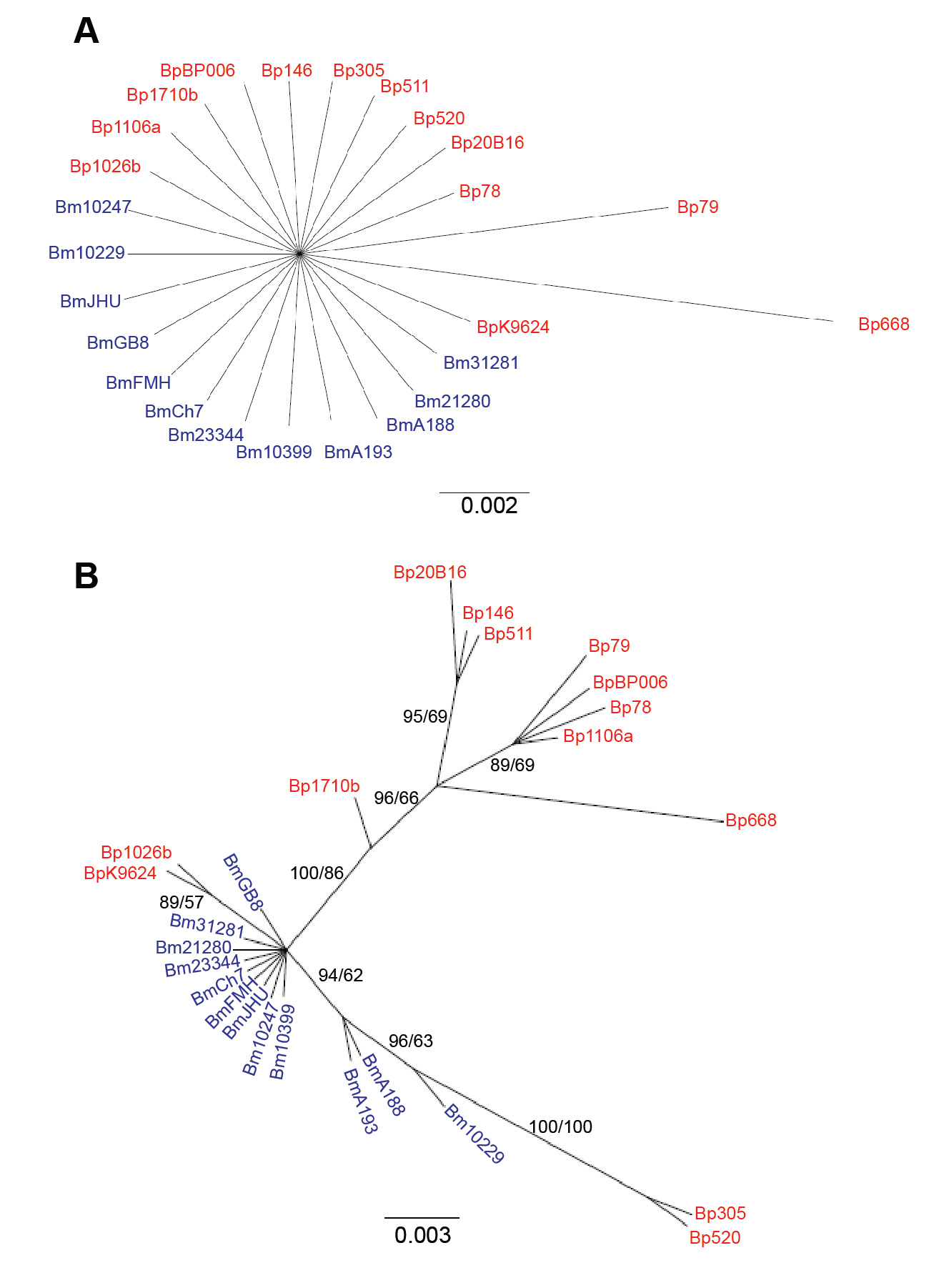

Supplement: S1 Fig — Nucleotide sequences encoding (A) the noncollagenous domain and (B) entire gene of bucl1 alleles were used. Support values for each branch are shown as posterior probability from Bayesian analysis and bootstrap values from maximum parsimony analysis, respectively (PP/MP). Scale bar is representative of evolutionary distance in substitutions per nucleotide. (JPG) [file pone.0137578.s003.jpg]

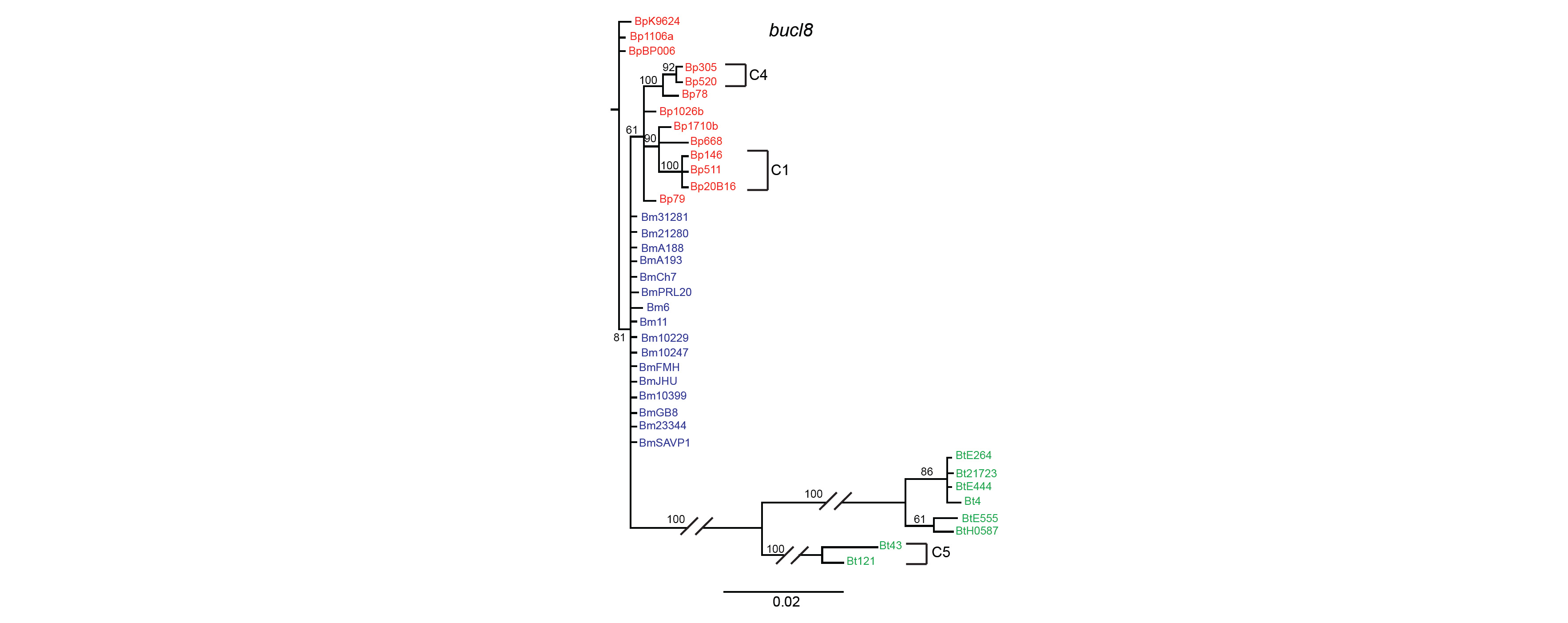

Supplement: S2 Fig — Bayesian analysis was performed on nucleotide sequences of bucl8 non-collagenous regions of a set of Burkholderia strains described in Table 3. Support values for each branch are shown as posterior probability from Bayesian analysis. Several clusters of strains, C1, C4, and C5, corresponding to those observed in the concatenated analysis were also observed. Scale bar is representative of evolutionary distance in substitutions per nucleotide. (JPG) [file pone.0137578.s004.jpg]

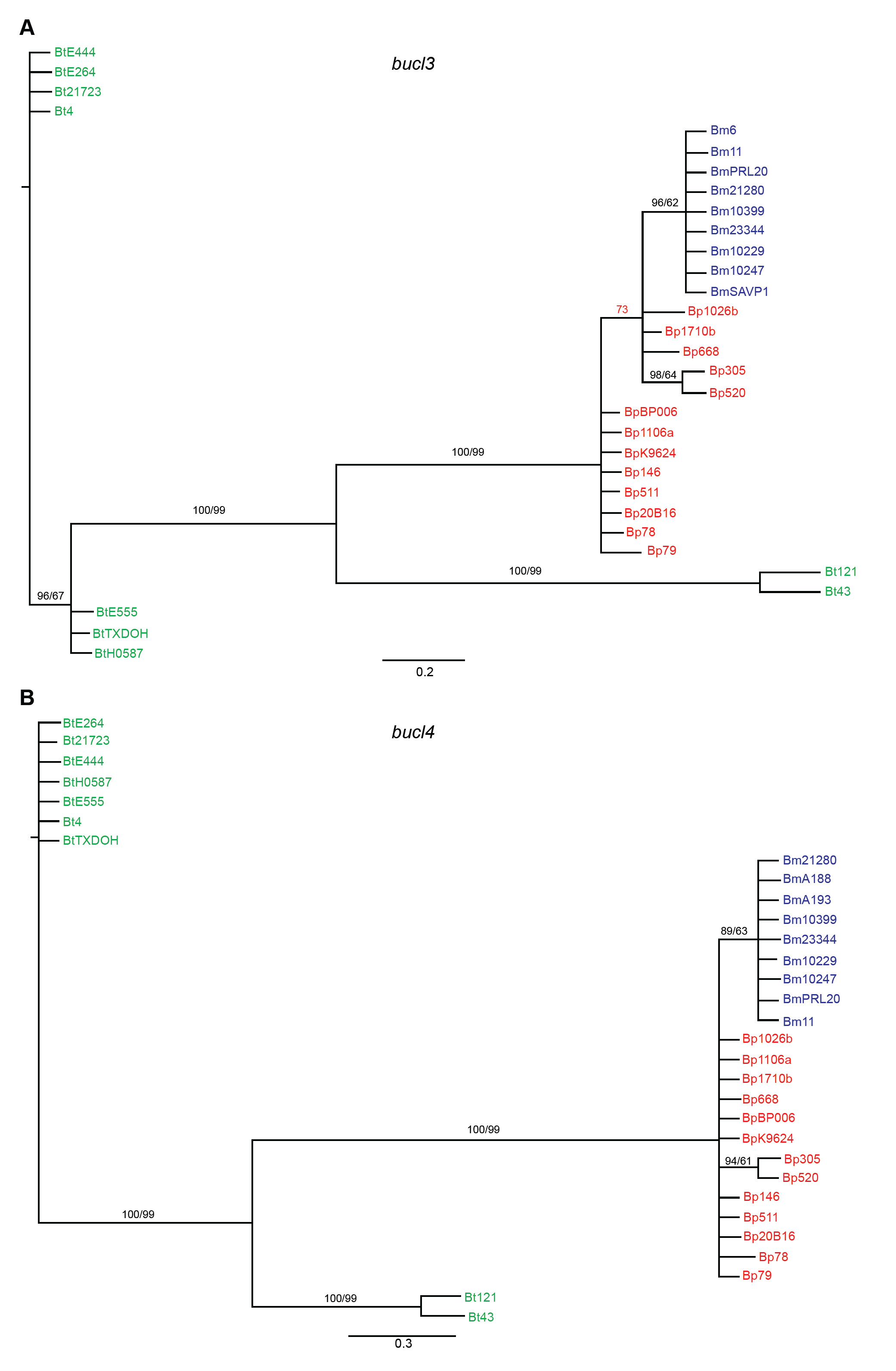

Supplement: S3 Fig — Bayesian analysis was performed on amino acid sequences of (A) Bucl3 and (B) Bucl4 non-collagenous regions of a set of Burkholderia strains described in Table 3. Support values for each branch are shown as posterior probability from Bayesian analysis and bootstrap values from maximum parsimony analysis, respectively (PP/MP). Posterior probability value, which was not supported by maximum parsimony analysis is shown in red. Scale bar is representative of evolutionary distance in substitutions per nucleotide. (JPG) [file pone.0137578.s005.jpg]

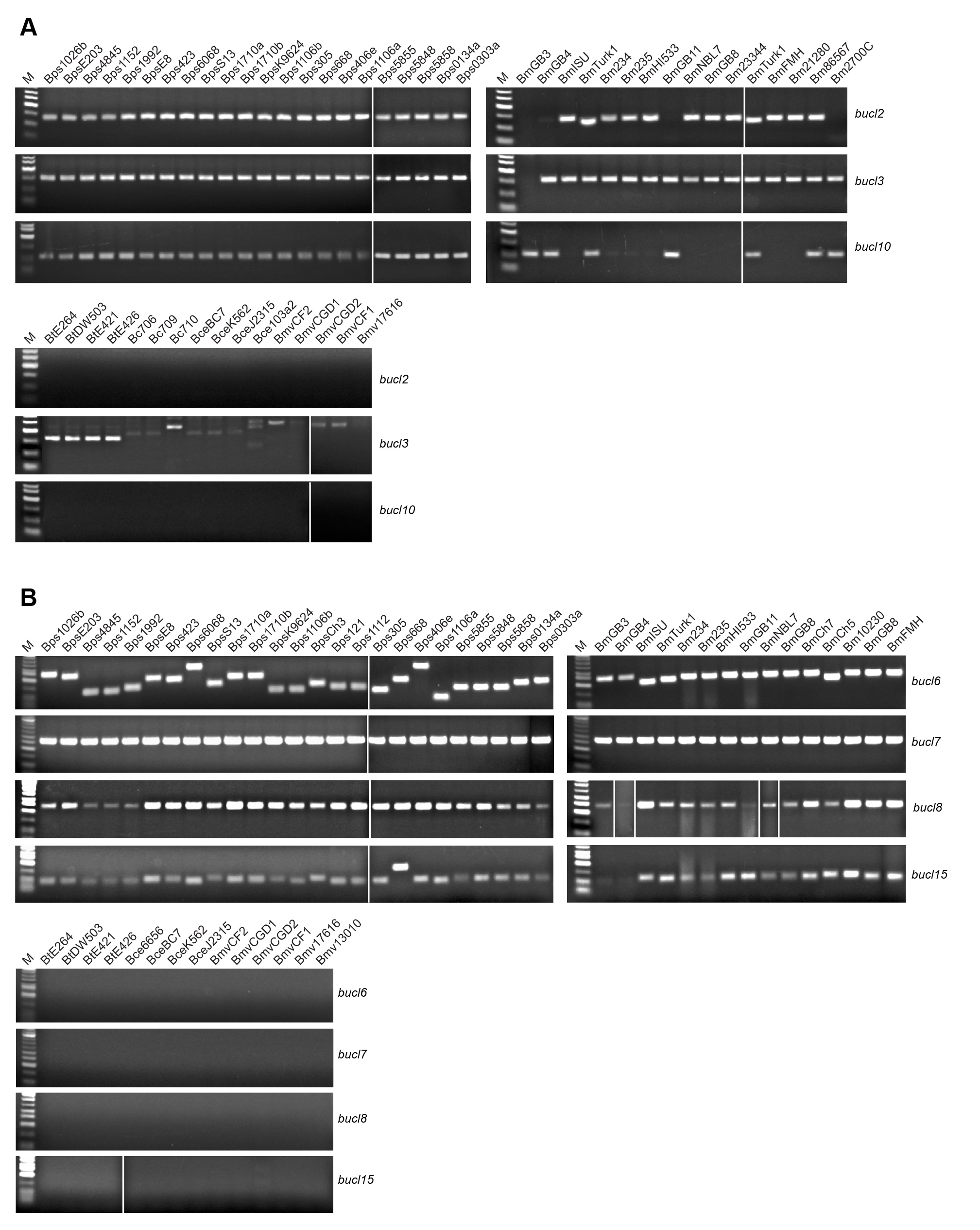

Supplement: S4 Fig — Presence of (A) bucl genes 2, 3, and 10 and (B) bucl genes 6, 7, 8, and 15, was assessed by PCR on a collection of genomic DNA from B. pseudomallei and B. mallei select agents (top panels), as well as in control strains of B. thailandensis, B. cepacia, B. cenocepacia, and B. multivorans (bottom panels). Amplicon sizes based on Bp K96243: In A) bucl2, 133 bp; bucl3, 166 bp; and bucl10, 109 bp; In B) bucl6, 115 bp; bucl7, 264 bp; bucl8, 243 bp; and bucl15, 95 bp.M, 50-bp DNA ladder. PCR data shown in panels A and B for 25 Bp strains come from two merged gel images. (JPG) [file pone.0137578.s006.jpg]
